# ISEV2021 Abstract Book

**DOI:** 10.1002/jev2.12083

**Published:** 2021-05-15

## About ISEV

The International Society for Extracellular Vesicles is the leading professional society for researchers and scientists involved in the study of microvesicles and exosomes. With nearly 1,000 members, ISEV continues to be the leader in advancing the study of extracellular vesicles. Founded in 2012 in Sweden, ISEV has since moved its Headquarters to the United States. Through its programs and services, ISEV provides essential training and research opportunities for those involved in exosome and microvesicle research.

## Mission Statement

Advancing extracellular vesicle research globally.

## Vision

Our vision is to be the leading advocate and guide of extracellular vesicle research and to advance the understanding of extracellular vesicle biology.

## ISEV2021 Annual Meeting

The International Society for Extracellular Vesicles is the is the premier international conference of extracellular vesicle research, covering the latest in exosomes, microvesicles and more. With an anticipated 1,000 attendees, ISEV2021 will feature presentations from the top researchers in the field, as well as providing opportunities for talks from students and early career researchers.

## ISEV2021 International Organizing Committee

IOC Chairs: Lorraine O'Driscoll (Ireland), Sophie Rome (France)

IOC Members: Antonella Bongiovanni (Italy), Dave Carter (United Kingdom), Vincent Hyenne (France), Soazig Le Lay (France), Andreas Möller (New Zealand), Eva Rohde (Austria), Tang‐Long Shen (Taiwan), Carolina Soekmadji (Australia), and Ken Witwer (USA)

## Journal of Extracellular Vesicles: Editors in Chief

Jan Lotvall (Sweden)


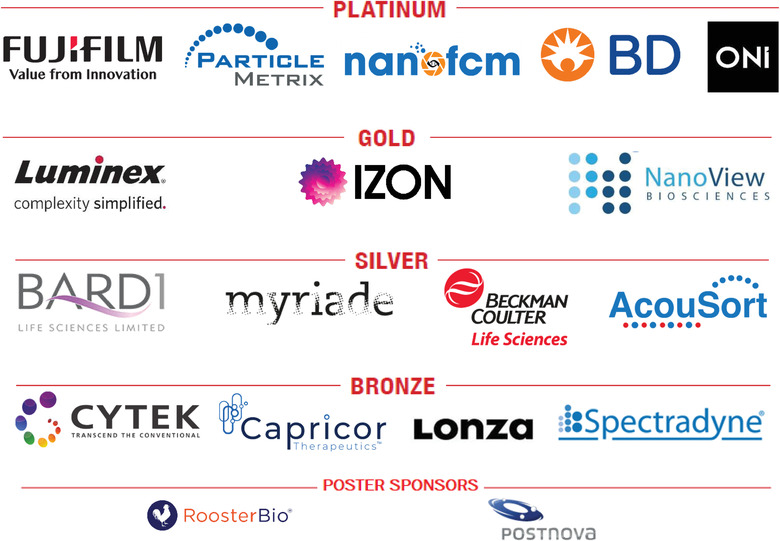


## PLEN1 Plenary 1 & Featured Abstract 1

Chair: Clotilde Thery, Institut Curie / INSERM U932, France

Chair: Kenneth Witwer, Johns Hopkins University School of Medicine, United States

### Plenary 1: EVs neurodegenerative diseases‐ Andrew Hill, Professor, La Trobe University

### FA01 Mitochondrial Dysfunction Alters the Number and Content of Mitovesicles, Newly Identified Mitochondria‐derived Extracellular Vesicles

Pasquale D'Acunzo, Nathan S. Kline Institute for Psychiatric Research

Efrat Levy, Center for Dementia Research, Nathan S. Kline Institute, Orangeburg, New York 10962, USA

**Introduction**: Mitochondrial damage is a well‐established player of neurodegenerative diseases, including Alzheimer's disease and Down syndrome (DS). We previously showed that the extracellular matrix of the brain contains a newly identified population of metabolically active extracellular vesicles (EVs) of mitochondrial origin that we have named ‘mitovesicles’. We investigated the effect of mitochondrial dysfunction in vivo on the number and content of mitovesicles in DS brains as compared with diploid controls and in vitro on secretion of mitovesicles by primary fibroblasts.

**Methods**: EVs were isolated from murine and human DS and control brains using a high‐resolution density step‐gradient that fractionates subtypes of EVs. EVs were analyzed by nanoparticle tracking analysis, Western blotting, mass spectrometry, and qPCR. EVs were also isolated from media of human fibroblasts following treatment with the electron transport chain inhibitor antimycin‐A and analyzed by Western blotting.

**Results**: The in vitro study revealed that mitochondrial damage enhances mitovesicle release in a mitophagy‐independent fashion. Consistently with these data, human and murine DS brains showed higher number of mitovesicles when compared to controls. Additionally, DS mitovesicles displayed perturbation in cargo loading, given that the amount of several mitochondrial proteins and mRNAs were lower in DS compared to controls when equal number of vesicles were considered. Quite the reverse, the amount of mitochondrial DNA, which is a strong pro‐inflammatory agent, was higher in DS mitovesicles compared to controls, consistent with the reported neuroinflammatory phenotype in DS.

**Summary/Conclusion**: Brain mitovesicle levels and cargo are modified in DS, suggesting that mitovesicles may be a previously unrecognized player of mitochondria quality control and may have a yet undiscovered role in the response to oxidative stress, neuroinflammation and synaptic regulation.

Supported by NIH grants AG017617, AG057517, AG056732, DA044489

## PLEN2 Plenary 2 & Featured Abstract 2

Chair: Susmita Sahoo, Department of Cardiology, Icahn School of Medicine at Mount Sinai, United States

Chair: Lei Zhang, Nanfang Hospital, China (People's Republic)

### Plenary 2: EVS in Cardiovascular Disorders‐ Chantal Boulanger, Research Director at the French Biomedical Research Agency (INSERM)

### FA02 Blood Flow Tunes Uptake and Fate of Extracellular Vesicles

Benjamin MARY , INSERM U1109 tumor biomechanis lab

Nandini ASOKAN, INSERM UMR_S1109, Tumor Biomechanics Lab; Université de Strasbourg, Fédération de Médecine Translationnelle de Strasbourg (FMTS), Strasbourg France

Olivier LEFEBVRE, INSERM UMR_S1109, Tumor Biomechanics Lab; Université de Strasbourg, Fédération de Médecine Translationnelle de Strasbourg (FMTS), Strasbourg France

Jacky GOETZ, INSERM UMR_S1109, Tumor Biomechanics Lab; Université de Strasbourg, Fédération de Médecine Translationnelle de Strasbourg (FMTS), Strasbourg France

Vincent Hyenne, INSERM / CNRS

**Introduction**: Circulating tumor EVs (ctEVs) are abundant in blood of cancer patients and favor metastasis by inducing the formation of pre‐metastatic niche in distant organs. Yet, how they react to the intravascular hemodynamic conditions remain poorly understood.

**Methods**: Here, we mimicked realistic bloodstream conditions in vitro and in vivo, using microfluidics and zebrafish respectively, to dissect the impact of blood flow parameters on the efficiency and endocytic route of ctEVs uptake.

**Results**: While moderate blood flow regimes (velocities around 400 μm/s) promote the uptake of ctEVs by the endothelium compared to a static condition, increasing shear and blood flow velocities cancel this positive effect. Adhesive properties of ctEVs is instrumental in their intravascular behavior notably via the adhesion molecule CD146 expressed on EV surface. Thus, it is likely that harsh hemodynamic constraints compete with their adhesive potential on the endothelium. Further and ongoing investigations will determine whether additional receptors (CD44, integrins) also contribute to ctEVs uptake.

Upon arrest, we identified clathrin‐independent endocytosis as a route of uptake of ctEVs and observed that hemodynamic forces affect the subcellular localization of ctEVs taken up by endothelial cells. We further observed that hemodynamic forces upregulate lysosomal pathways in endothelial cells. We are currently testing whether tuning blood flow impacts ctEVs cargo transfer and how this affects endothelial behavior using a combination of imaging and transcriptomic approaches.

**Summary/Conclusion**: Altogether, our work demonstrates that hemodynamics could tune the endothelial uptake of ctEVs. Since this is likely to control the function of ctEVs, we propose a novel route by which ctEVs can impact the establishment of pre‐metastatic niches.

## PLEN3 Plenary 3 & Featured Abstract 3

Chair: Andreas Moller, Group Leader, Tumour Microenvironment Laboratory, QIMR Berghofer Medical Research Institute, Associate Professor, University of Queensland and Queensland University of Technology, Australia

Chair: Lorraine O'Driscoll, Trinity College Dublin, Ireland

### Commensal gut bacteria‐derived extracellular vesicles: Mediators of gut microbe‐host crosstalk and vehicles for mucosal drug delivery‐ Simon Carding, Professor Mucosal Immunology

### FA03 Melanoma‐secreted exosomes prepare the formation of pre‐metastatic niches in sentinel lymph nodes

Susana Garcia‐Silva , Spanish National Cancer Research Center (CNIO), Madrid, Spain.

Alberto Benito‐Martín, Weill Cornell Medicine, New York, USA

Laura Nogues, Spanish National Cancer Research Center (CNIO)

Alberto Hernández‐Barranco, Spanish National Cancer Research Center (CNIO)

Vanesa Santos, Spanish National Cancer Research Center (CNIO)

Marina Mazariegos, Spanish National Cancer Research Center (CNIO)

Marta Hergueta, Spanish National Cancer Research Center (CNIO)

Raghu P. Kataru, Memorial Sloan Kettering Cancer Center

Sara Sanchez‐Redondo, Spanish National Cancer Research Center (CNIO)

Osvaldo Graña‐Castro,Spanish National Cancer Research Center (CNIO)

Irina Matei, Weill Cornell Medicine

Sagrario OrtegaSpanish National Cancer Research Center (CNIO)

Raúl Torres‐Ruiz, Spanish National Cancer Research Center (CNIO)

Sandra Rodríguez‐Perales,Spanish National Cancer Research Center (CNIO)

Lola Martínez,Spanish National Cancer Research Center (CNIO)

Manuel Pérez,Spanish National Cancer Research Center (CNIO)

Diego Megías,Spanish National Cancer Research Center (CNIO)

Babak J. Mehrara,Memorial Sloan Kettering Cancer Center

David Lyden, Weill Cornell Medicine

Hector Peinado,Spanish National Cancer Center

**Introduction**: Secreted EVs built a network of communication around primary tumors and distant organs favoring metastasis. Our previous works have demonstrated the role of tumor‐secreted EVs in pre‐metastatic niche formation and metastatic organotropism in distal organs. In this work we have analyzed the dynamics of tumor‐derived small EVs (sEVs) in the lymphatic system and their role establishing the pre‐metastatic niche formation in sentinel lymph nodes.

**Methods**: We have analyzed melanoma‐derived sEV biodistribution in the lymphatic system by in vivo imaging, confocal microscopy and flow cytometry. We performed RNA‐seq in lymphatic endothelial cells after sEV uptake combined with proteomic analysis of sEVs. We have analyzed the effect of sEV‐shed neurotrophin receptor lymphangiogenesis both in vitro and in vivo. In addition, we performed experimental and spontaneous metastasis as well as survival assays after education with melanoma‐derived sEVs in preclinical models. Finally, we have evaluated the expression of neurotrophin receptors in primary tumors and sentinel lymph nodes correlating with disease outcome.

**Results**: We found that sEVs derived from metastatic melanoma cell lines spread through the lymphatic system. We observed that lymphatic endothelial cells are the main and first cell type incorporating tumor‐derived sEVs followed by macrophages in the lymph nodes. Melanoma‐derived sEVs induced lymphangiogenic gene expression (e.g. LYVE‐1, VEGF‐C), tumor cell adhesion, and the activation of ERK and NF‐kB pathways in lymphatic endothelial cells. Mechanistically, we observed that neurotrophin receptors are secreted in melanoma‐derived sEVs and shuttled to lymphatic endothelial cells concomitant with the acquisition of a pro‐lymphangiogenic phenotype. Blocking sEVs‐induced signals reduced melanoma metastasis and improved survival in pre‐clinical models. Analysis of human samples showed that the expression of specific neurotrophin receptors in metastatic pioneering cells within the sentinel lymph node is correlated with poor survival.

**Summary/Conclusion**: Our data shows for the first time that both tumor intrinsic and extrinsic factors such as tumor‐secreted sEVs are involved in lymphangiogenesis and pre‐metastatic niche formation in sentinel lymph nodes through an neurotrophin receptor‐dependent mechanism favoring metastatic spread in melanoma.

## PLEN4 Plenary 4 & Featured Abstract 4

Chair: An Hendrix, Laboratory of Experimental Cancer Research, Department of Human Structure and Repair, Ghent University, Ghent, Belgium

Chair: Vincent Hyenne, INSERM / CNRS, France

### Biogenesis of the Multivesicular Endosome‐ Jean Gruenberg, Honorary Professor, Department of Biochemistry

### FA04 Specificity determinants for RNA release into extracellular vesicles

Marie Mosbach, Institute of Biochemistry, Justus Liebig University of Gießen

Christina Pfafenrot, Institute of Biochemistry, Justus Liebig University of Gießen

Elke Pogge von Strandmann, Institute for Tumorimmunology, Center for Tumor Biology and Immunology, Philipps University Marburg

Albrecht Bindereif, Institute of Biochemistry, Justus Liebig University of Gießen

Christian Preußer, Institute for Tumorimmunology, Center for Tumor Biology and Immunology, Philipps University Marburg

**Introduction**: Extracellular vesicles (EVs) are important for intercellular communication and act as vehicles for biological material such as various classes of coding and non‐coding RNAs, a few of which have been shown to be selectively targeted into vesicles. However, factors and mechanisms contributing to this specificity remain largely elusive, and only a few putative protein factors involved in packaging have been described.

**Methods**: Here we used reporter systems to decipher the loading efficacy of different RNA species into EVs, based on different expression constructs and transfection in mammalian cells: First, we studied RNA polymerase‐ (RNA pol) dependent effects at the 5' end of the RNA as well as different transcriptional terminators at the 3' end. Second, the size dependence of RNA loading was investigated, based on a series of reporter constructs. The relative and the absolute abundance of the different reporters as well as of some endogenous RNAs were determined.

**Results**: We could show that RNA pol III transcripts are more efficiently loaded into EVs than RNA pol II transcripts, and that even in these overexpression system, only relatively few RNA molecules per EV could be detected. This conclusion is further supported by the absolute quantification of endogenous EV‐associated RNAs. Regarding size distribution, we observed that shorter RNAs are more efficiently released into EVs than longer RNAs, indicating that size is an important determinant of RNA export.

**Summary/Conclusion**: Our results reflect the current debate on EV‐associated RNAs. The initial enthusiasm on the enrichment of various RNAs in EVs has been dampened by some reports and our own analysis: RNAs appear to be EV‐associated only at low copy numbers, and the question is still open whether this RNA association reflects internal EV encapsulation, or merely a less tightly bound state at the vesicle surface.

## Concurrent Sessions (CC)

## CC1 How are EVs Involved in Cancer Pathogenesis?

Chair: Hector Peinado, Spanish National Cancer Center, Spain

Chair: Lucia R. Languino, Thomas Jefferson University, United States

### CC1.1 Mitochondrial‐lysosomal crosstalk induces mitochondrial‐derived vesicle generation in cisplatin chemoresistance

Flora Guerra, Department of Biological and Environmental Sciences and Technologies (DiSTeBA), University of Salento

Sinforosa Gagliardi, Department of Biological and Environmental Sciences and Technologies (DiSTeBA), University of Salento

Silvia Caterina Resta, Department of Biological and Environmental Sciences and Technologies (DiSTeBA), University of Salento

Cecilia Bucci, Department of Biological and Environmental Sciences and Technologies (DiSTeBA), University of Salento

**Introduction**: Different studies suggest a key role of the crosstalk between mitochondria and lysosomes in cellular physiology and its dysregulation is present in cancer. Indeed, it is known that mitochondrial impairment can influence lysosomal function and viceversa. RAB7 is a small GTPase with multiple key roles in cellular physiology. RAB7 controls transport to late endocytic compartments and regulates late endocytic organelle biogenesis, lysosomal positioning and functions, trafficking and degradation of several signaling receptors and extracellular vesicle (EV) secretion. In recent works, RAB7 was described also as regulator of mitophagy, mitochondrial‐lysosomal contacts and mitochondrial dynamics. Moreover, it is known that RAB7 may determine fusion between mitochondrial derived vesicles (MDVs) with late endosome, but its role in MDVs biogenesis is poorly understood.

**Methods**: In this context, we have purified trough ultracentrifugation and immunoisolation EVs from cisplatin chemosensitive and chemoresistant ovarian cancer cell lines. We verified their endosomal biogenesis and mitochondrial content through western blotting. Moreover, we performed PCR to analyze the presence of mitochondrial DNA (mtDNA).

**Results**: We found that RAB7 downregulation and impairment of late endocytic functions characterize all chemoresistant cells. Moreover, in chemoresistant cells we observed increase of EV secretion compared to matched chemosensitive cells. Interestingly, we found that purified EVs contain several mitochondrial proteins and mtDNA.

**Summary/Conclusion**: Here, we concluded that cisplatin chemoresistance is associated with alteration of late endocytic pathway and with consequent increase of EV secretion. It is known that cisplatin treatment induces mitochondrial dysfunction. In this context, RAB7 is not able to induce autophagic degradation of dysfunctional mitochondria which are secreted becoming potential markers of chemoresistance.

### CC1.2 The role of Notch pathway in the pro‐tumorigenic activity of extracellular vesicles in multiple myeloma

Domenica Giannandrea , Università degli Studi di Milano

Michela Colombo, Università degli Studi di Milano

Natalia Platonova, Università degli Studi di Milano

Valentina Citro, Università degli Studi di Milano

Mara Mazzola, Università degli Studi di Milano

Anna Pistocchi, Università degli Studi di Milano

Raffaella Adami, Università degli Studi di Milano

Vincenza Dolo, Università degli Studi dell'Aquila

Ilaria Giusti, Università degli Studi dell'Aquila

Laura Cantone,Università degli Studi di Milano

Valentina Bollati, Università degli Studi di Milano

Mauro TurriniValduce Hospital

Raffaella Chiaramonte, Università degli Studi di Milano

**Introduction**: Multiple myeloma (MM) is characterized by the tight interaction between MM cells and bone barrow (BM) niche, resulting in tumor progression.

MM cells overexpressed Notch 2 and Jagged 1 and 2, triggering Notch pathway activation on BM population and their pro‐tumorigenic activity.

Extracellular vesicles (EV) represent novel pro‐tumorigenic players in the MM microenvironment.

In this work we assess the tumorigenic effect of MM‐derived EVs and the role played by the Notch pathway in EV‐mediated communication between MM cells and the BM cells.

**Methods**: EVs from MM cell lines (MM‐EVs) or MM cell lines constitutively inhibited for Jagged1/2 (MMJ1/2KD‐EVs) or Notch2 (MMN2KD‐EVs) were characterized for Notch2 and Jagged1 and 2 content by Western blot and for size and number by nanoparticle tracking analysis and electronic transmission microscopy. The transfer of HA‐tagged Notch2 via EVs was evaluated by an engineered system of HEK293 sending and receiving cells. Notch pathway activation was evaluated in vivo by injecting MM‐EVs in the duct of Cuvier of 2 days post fertilization Notch‐reporter Tg(T2KTp1bglob:hmgb1‐mCherry)jh transgenic zebrafish embryos. The pro‐tumorigenic effect of MM‐EVs, MMJ1/2KD‐EVs and MMN2KD‐EVs were assessed in vitro by measuring the osteoclastogenic potential, the ability to induce human endothelial cells to organize tubular structures and assessing changes in stromal cell‐mediated drug resistance.

**Results**: MM‐EVs carry Notch2 Jagged1 and 2 and transfer them to recipient cells; Notch members levels depend on their expression in MM cells.

The analysis of the functional effects indicates that MM‐EVs interact and activate Notch pathway in receiving cells in vitro and in vivo and display a pro‐tumorigenic effect. MM‐EVs show osteoclastogenic effect and angiogenic ability and boost drug resistance induced by the BM stromal cells HS5. All these effects are lost when EVs are produced by MMJ1/2KD and MMN2KD cells.

**Summary/Conclusion**: These results provide the first evidence that targeting the Notch pathway may be a valid therapeutic strategy to hamper the pro‐tumorigenic role of EV in MM progression.

### CC1.3 Extracellular vesicles from triple negative breast cancer promote differentiation of pro‐inflammatory macrophages associated with better clinical outcome

Jessie Thalmensi, Institut Curie / INSERM U932

Eleonora Timperi, Institut Curie / INSERM U932

Paul Gueguen, Institut Curie / INSERM U932

Nathalie Névo, Institut Curie / INSERM U932

Eleonora Grisard, Institut Curie U932

Philemon Sirven, Institut Curie / INSERM U932

Federico Cocozza, Institut Curie / INSERM U932

Alizée Gouronnec, Institut Curie / INSERM U932

Lorena Martin‐Jaular,Institut Curie / INSERM U932

Mabel Jouve, Institut Curie / CNRS UMR 3215

Coralie GuérinInstitut Curie / FlowCytométrie Platform

Vassili Soumelis, Université de Paris / INSERM U976

Emanuela Romano,Institut Curie / INSERM U932

Elodie Segura,Institut Curie / INSERM U932

Clotilde Thery, MD PhD,Institut Curie / INSERM U932

Clotilde Thery, MD PhD , Institut Curie / INSERM U932

**Introduction**: Tumor associated macrophages (TAMs) are highly abundant in human cancers, representing widely heterogeneous populations. The contribution of various tumor‐derived signals to differentiation of circulating monocytes into distinct TAM subsets is not well understood. In particular, tumors release both soluble factors and extracellular vesicles (EVs: exosomes, ectosomes and others) containing a complex set of signaling molecules, whose impact on TAM precursors may be different.

**Methods**: Here, we used Size‐exclusion chromatography (SEC) to separate EVs from soluble molecules in the secretome of triple negative breast cancer (TNBC) cell lines. We cultured human blood monocytes with EV‐rich or EV‐poor SEC fractions and analyzed the phenotype of differentiated monocytes in terms of cell surface marker expression and cytokine secretion, and by global transcriptomic analysis. We generated by CRISPR/Cas9 tumor cell lines KO for several genes that we identified as relevant to disclose the mechanisms mediating the observed effects (CSF1, Rab11, STING). We compared the in vitro obtained gene signatures with those of macrophages isolated from human breast tumor patients and analysed by single cell RNASeq.

**Results**: We show that both EVs and soluble secretome promote monocyte differentiation towards macrophages. However, EVs specifically promoted a subset of pro‐inflammatory macrophages bearing an IFN signature. CSF‐1 exposed on EVs was necessary for macrophage differentiation and the cGAS/STING axis was involved in the activation of the IFN‐response. Macrophages imprinted with an EV‐signature or with the soluble molecule signature were both found in patient's TAMs. Strikingly, EV‐induced macrophage signature positively correlated with T cell infiltration and patient survival.

**Summary/Conclusion**: Together these data suggest that TNBC‐released CSF‐1‐bearing EVs promote a tumor immune microenvironment associated with a favourable prognosis in TNBC patients.

### CC1.4 Cancer cells shuttle extracellular vesicles containing oncogenic mutant p53 proteins to the tumor microenvironment

Bibek Bhatta, Ben‐Gurion University of the Negev

Ishai Luz, Ben‐Gurion University of the Negev

Christian Krueger, Division of Cellular & Molecular Research, Humphrey Oei Institute of Cancer Research, National Cancer Centre Singapore

Fanny Xueting Teo, Division of Cellular & Molecular Research, Humphrey Oei Institute of Cancer Research, National Cancer Centre Singapore

David Lane, p53 Laboratory (p53Lab), Agency for Science, Technology, and Research (A*STAR)

kanaga sabapathy, Division of Cellular & Molecular Research, Humphrey Oei Institute of Cancer Research, National Cancer Centre Singapore

Tomer Cooks, Ben‐Gurion University of the Negev

**Introduction**: Mutations in the TP53 gene (encoding for the p53 tumor suppressor protein) are the most common molecular event in human cancer. Cancer cells harboring gain‐of‐function (GOF) mutant p53 are more aggressive than cancer cells harboring inactivating mutations or wild‐type (WT)‐p53. Notably, multiple studies have delineated the presence of GOF‐mutant p53 protein in untransformed cells or in stromal compartments of tumor microenvironment (TME). In recent years, the involvement of extracellular vesicles (EVs) in cell‐to‐cell communication has emerged as a major route by which cells can interact with each other. On the same note, cancer cells were shown to produce excessive amounts of EVs received by TME cells, thus recruiting the TME to become tumor‐supportive, therefore suggesting that EVs‐mediated communication between cancer cells and cells of TME imparts an aggressive trait to the underlying cancer. To this end, we hypothesize that mutant p53 protein can be shuttled via EVs to TME cells thus shedding light on a novel non‐cell autonomous role of mutant p53 cancers.

**Methods**: EVs were isolated from various cancer cell lines differing by their p53 status and the effect on neighboring cancer cells and TME cells was studied in vitro and in‐vivo. We used PANC‐1 pancreatic ductal adenocarcinoma cells, we knocked‐out the R273H endogenous mutant p53 using the CRISPR‐Cas9 system as well as with shRNA stable knockdown of the mutant p53 gene. In H358 lung carcinoma cells, we used Tetracycline‐controlled transcriptional activation cell system overexpressing several different p53 GOF mutants (V157F, R175H, R249S and R273H), simulating inactivated p53, compared with the WT form and an empty vector. We utilized the human colorectal Colo‐320DM cancer cell xenograft model, which expresses the R248W p53 mutant. FFPE sections of subcutaneous tumors derived from the Colo‐320DM xenografts were stained for p53 using the DO‐1 antibody that specifically recognizes human p53.

**Results**: Our data demonstrated that mutant p53 protein can be selectively sorted into EVs; that mutant p53 in EVs can be taken up by neighboring cancer cells and macrophages that do not harbor mutant p53 and that mutant p53 expression is found in non‐tumor cells in both human cancers, and in non‐human tissues in human xenografts.

**Summary/Conclusion**: In this report, we tested and corroborated the fundamental hypothesis suggesting the cancer cells that harbor GOF p53 mutants, can package these mutant proteins in EVs, and deliver them to neighboring cancer cells and to the TME.

### CC1.5 A comprehensive comparative analysis of extracellular vesicle release in non‐small cell lung cancer and its potential to drive cancer hallmarks in non‐cancerous lung epithelial cells

Humna Hasan, Purdue University

Ikjot Singh Sohal, Purdue University

Zulaida M. Soto‐Vargas, zsotovar, Purdue University

Anjali Byappanhalli, Purdue University

Sean Humphry, Purdue University

Hana Kubo, Northwestern University

Sarunya Kitdumrongthum, Mahidol University, Thailand

Arthit Chairoungdua, Mahidol University, Thailand

Andrea Kasinski, Purdue University

**Introduction**: Cancer‐derived extracellular vesicles (EVs) play a pivotal role in cancer progression by mediating bi‐directional communication between cancer cells and their environment. While multiple lines of evidence have shown how non‐small cell lung cancer (NSCLC) EVs promote cancer progression by evaluating distinct aspects of cancer, it remains unclear how EVs from different NSCLC cells differ in their secretion profile and in their potential to promote various cancer hallmarks.

**Methods**: To address this need, we performed a comparative analysis of (i) EV release from non‐cancerous bronchial epithelial cells (HBEC/BEAS‐2B) and several NSCLC cells (A549, H460, H358, SKMES and Calu6) as well as (ii) the potential of NSCLC EVs including EV‐encapsulated RNA in driving cancer hallmarks in HBEC/BEAS‐2B cells.

**Results**: The isolated EVs were in the 100–150nm size range and were enriched in CD9 and CD81 tetraspanins. While literature indicates that cancer cells generally have higher secretion rate, our secretion analysis indicated that only two (Calu6 and H358) out of 5 NSCLC cells had higher secretion rate compared to HBEC/BEAS‐2B cells. We observed differential uptake of NSCLC EVs by non‐cancerous cells with A549 and SKMES EVs showing the highest uptake. We showed that EVs derived from high secretion rate cells (H358 and Calu6) were able to disrupt BEAS‐2B epithelial barrier and significantly increase permeability, which was further confirmed by downregulation of E‐cadherin and ZO‐1 junctional complex proteins. Similarly, only H358 and Calu6 EVs dramatically enhanced invasive phenotype in HBEC/BEAS‐2B cells. On the other hand, EVs derived from low secretion rate cells neither impaired epithelial barrier (except SKMES) nor induced invasive phenotype to a large extent. Furthermore, EV‐encapsulated RNA was attributed as a contributing factor in mediating the above‐mentioned phenotypes for H358 and Calu6 EVs.

**Summary/Conclusion**: More nuanced analysis suggested that the NSCLC subtypes can be correlated with the potential of their distinct EVs to drive cancer hallmarks. In conclusion, the study lays the groundwork which will guide future studies to detail the role of EVs and their cargo in modulating the microenvironment at various stages of lung cancer progression.

## CC2 What's New with EVs in the Brain?

Chair: Andrew Hill, La Trobe University, Australia

Chair: Eva‐Maria Albers Kramer, Institut für Entwicklungs‐ und Neurobiologie Zelluläre Neurobiologie AG Extrazelluläre Vesikel, Germany

### CC2.1 Genome‐wide shRNA screening identifies factors required for exosome secretion from microglia

ZHI RUAN , Department of Pharmacology & Experimental Therapeutics, Boston University School of Medicine

Kayo Takamatsu‐Yukawa, Department of Pharmacology & Experimental Therapeutics, Boston University School of Medicine

Yuzhi Wang, Department of Pharmacology & Experimental Therapeutics, Boston University School of Medicine

Adam T. Labadorf, Department of Neurology, Bioinformatics Program, Boston University

Seiko Ikezu, Department of Pharmacology & Experimental Therapeutics, Boston University School of Medicine

Tsuneya Ikezu, MD, PhD, Department of Pharmacology & Experimental Therapeutics, Center for Systems Neuroscience,Boston University School of Medicine; Department of Neuroscience, Mayo Clinic Florida

**Introduction**: Microglia are the principal immune cells in the central nervous system, serving for functional and metabolic homeostasis as well as innate immune response to pathogen invasion and neuronal damage. After rapidly responding to noxious stimuli, activated microglia could release exosomes, the smallest extracellular vesicles, carrying various pro‐inflammatory cytokines including IL‐1β. The molecules critical for regulating the exosome production from microglia are yet to be understood.

**Methods**: Tetraspanin protein CD63 was constructed with tdTomato and packaged by VSV‐G pseudotyped lentivirus to establish murine microglial BV2 cell line stably expressing CD63‐tdTomato. The pooled shRNA library was constructed with the Dharmacon SMARTvector Lentiviral shRNA library expressing short hairpin RNAs (shRNAs), which target genome‐wide 21,745 genes with 8 clones per target and expressing TurboGFP under the control of murine EF1α promoter. After sorting of TurboGFP+ CD63‐tdTomato+ BV2 cell library, cells were exposed to 5mM ATP stimulation for exocytosis of CD63‐tdTomato+ exosomes, and sorted for TurboGFP+ CD63‐tdTomatohigh and CD63‐tdTomatolow cells. The sorted cells were subjected to genomic DNA extraction, PCR amplification of barcoded shRNA region and next generation sequencing. The enrichment of shRNA clones in CD63‐tdTomatohigh and CD63‐tdTomatolow cells were ranked by the Z‐score to identify the “Hit” candidates.

**Results**: ATP‐induced exosome secretion from CD63‐tdTomato+ BV2 cells and reduction of the tdTomato signal in cells, showing that CD63‐tdTomatohigh and CD63‐tdTomatolow cells show suppression or enhancement of CD63‐tdTomato+ exosomes, respectively. By using a barcoded lentivirus‐based pooled short‐hairpin RNA (shRNA) library combined with next generation sequencing, we identified 1353 silenced host genes highly enriched in cells resistant to the ATP induced exosome secretion. The majority of these genes are the integral component of membrane and plasma membrane and participate in the cell‐to‐cell communication as determined by the DAVID gene ontology analysis and Metascape. Validation experiments were performed on several top hits, and determined essential for the exosome secretion from microglia as assessed by nanoparticle tracking analysis and CD63 ELISA. Finally, silencing of these three genes by siRNAs suppressed the lipopolysaccharide‐ and ATP‐induced IL‐1β release from murine primary cultured microglia.

**Summary/Conclusion**: These findings provided novel candidate genes for microglial exosome secretion, which is a therapeutic target of neuroinflammatory and neurodegenerative disorders.

### CC2.2 Novel method for isolating extracellular vesicles from hippocampal interstitial fluid in Alzheimer's disease

Morgan Pait, Wake Forest School of Medicine

Sarah Kaye, Wake Forest School of Medicine

Yixin Su, Wake Forest School of Medicine

Andy Snipes, Wake Forest School of Medicine

Jingyun Lee, PhD, Wake Forest School of Medicine

Cristina Furdui, PhD, Wake Forest School of Medicine

Gagan Deep, PhD, Wake Forest School of Medicine

Shannon Macauley, PhD, Wake Forest School of Medicine

**Introduction**: Abnormal protein aggregation is a hallmark of Alzheimer's disease (AD). Exosomes, endosome‐derived, small extracellular vesicles (EVs), can transport AD‐related proteins amyloid‐beta (Aβ) and tau, making them potential AD blood‐based biomarkers. However, it is unclear how peripheral exosomes compare to exosomes in the brain's interstitial fluid (ISF) as AD pathology progresses in vivo. Here we describe a novel method for collecting exosome‐enriched EVs from hippocampal ISF using in vivo microdialysis in the presence and absence of Aβ pathology.

**Methods**: In vivo microdialysis was used to collect hippocampal ISF from 3‐and 9‐month‐old, unanaesthetized, unrestrained, APPswe/PS1″E9 (APP/PS1), a mouse model of Aβ overexpression, and B6C3 wildtype (WT) mice. Exosome‐enriched ISF EVs were isolated via ultracentrifugation then underwent nanoparticle tracking analysis and immunogold labeling. Mass spec and proteomic analysis were performed on both EV surface and cargo proteins.

**Results**: ISF EVs from APP/PS1 and WT mice were 40–150nm and CD63‐ and CD9‐positive. Of ExoCarta's Top 100 exosome proteins, 59 were found in the 3mo, WT ISF EVs. EV concentration increased with age in males, but not females, and was lower in APP/PS1 vs. WT. In 3mo APP/PS1 mice, fewer endothelial‐related proteins were on EV surface compared to WT. At 9mo, following Aβ plaque formation, astrocyte, microglia, neuronal and oligodendrocyte‐related proteins were altered on the surface and in the core of exosome‐enriched EVs. EV surface and core proteins changed with age, with 612 proteins unique to 3mo, 161 unique to 9mo, and 333 proteins shared across ages. At 3mo, WT and APP/PS1 shared 325 proteins, WT had 495 unique proteins and APP/PS1 had 125 unique proteins. At 9mo, WT and APP/PS1 shared 372 proteins, while 101 were specific to WT but only 21 to APP/PS1.

**Summary/Conclusion**: In vivo microdialysis is a novel method for brain ISF EV collection. Exosome‐enriched, ISF EVs are altered with age and Aβ pathology. Aβ plaques may impair release, clearance and/or uptake of EVs sex‐dependently. Furthermore, cell‐type specific (astrocyte, neuron, etc.) ISF EVs and their contents are altered in APP/PS1 mice. In vivo ISF exosome‐enriched EVs offer a unique opportunity to identify novel AD biomarkers and validate them in peripheral EVs.

### CC2.3 Outreach of striatum derived EVs in the mouse brain

David Rufino‐Ramos, CNC ‐ Center for Neuroscience and Cell Biology, University of Coimbra, Coimbra, Portugal

Koen Breyne, PhD, Molecular Neurogenetics Unit, Department of Neurology and Center for Molecular Imaging Research, Department of Radiology, Massachusetts General Hospital and Program in Neuroscience, Harvard Medical School, Boston, MA, USA

Killian O'Brien, PhD, Molecular Neurogenetics Unit, Department of Neurology and Center for Molecular Imaging Research, Department of Radiology, Massachusetts General Hospital and Program in Neuroscience, Harvard Medical School, Boston, MA, USA

Kevin Leandro, CNC ‐ Center for Neuroscience and Cell Biology, University of Coimbra, Coimbra, Portugal

Thomas S Van Solinge, Molecular Neurogenetics Unit, Department of Neurology and Center for Molecular Imaging Research, Department of Radiology, Massachusetts General Hospital and Program in Neuroscience, Harvard Medical School, Boston, MA, USA

Shadi Mahjoum, Molecular Neurogenetics Unit, Department of Neurology and Center for Molecular Imaging Research, Department of Radiology, Massachusetts General Hospital and Program in Neuroscience, Harvard Medical School, Boston, MA, USA

Sevda Lule, Molecular Neurogenetics Unit, Department of Neurology and Center for Molecular Imaging Research, Department of Radiology, Massachusetts General Hospital and Program in Neuroscience, Harvard Medical School, Boston, MA, USA

Shilpa Prabhakar, Molecular Neurogenetics Unit, Department of Neurology and Center for Molecular Imaging Research, Department of Radiology, Massachusetts General Hospital and Program in Neuroscience, Harvard Medical School, Boston, MA, USA

Luís Pereira De Almeida, CNC ‐ Center for Neuroscience and Cell Biology, University of Coimbra, Coimbra, Portugal;

Xandra O Breakefield, Molecular Neurogenetics Unit, Department of Neurology and Center for Molecular Imaging Research, Department of Radiology, Massachusetts General Hospital and Program in Neuroscience, Harvard Medical School, Boston, MA, USA

**Introduction**: Extracellular vesicles (EVs) are known as mediators of intercellular communication, primarily due to their capacity to transfer functional cargo and ultimately modulate cellular function. Transfer of functional cargo in the brain can be demonstrated using the Cre‐LoxP reporter system.

The aim of this work is to evaluate the biodistribution of functional striatum‐derived EVs in a mouse brain over time by exploiting the genome modifications ilicited by EV‐encapsulated Cre‐recombinase (Cre) in the recipient cells of a floxed reporter mice.

**Methods**: HEK293T cells were transduced with lentivirus encoding Cre and Fluc under the PGK and UBC promoter, respectively. EVs were isolated by Size Exclusion Cromatography (SEC) and the presence of Cre encoding mRNA was demonstrated by qPCR. In vitro transfer was determined by direct co‐culture and incubation of CRE‐EVs with both Ai9 and Nanoluc reporter cell lines. Fluorescence and luminescence was measured and RT‐PCR was used to confirm editing at the DNA level.

The lentivirus was injected into the right striatum of Ai9 reporter mice. Mice were monitored by IVIS imaging and were sacrified at designated time‐points. Whole brain coronal sections were analyzed for tdTomato‐positive signal by immunohistochemistry and ddPCR. Moreover, the level of DNA editing in other brain regions was analysed by correlating floxed and unfloxed DNA.

**Results**: Stably transduced cells were able to produce EVs encapsulating functional Cre mRNA that was delivered to Ai9 and Nanoluc reporter cell lines, resulting in induction of tdTomato and luciferase in a time‐dependent manner. Ai9 mice intracranially injected with lentivirus encoding Cre and Fluc express functional Cre and tdTomato signal not only at the injection site, but also in other brain regions.

**Summary/Conclusion**: This study emphasizes the ability of striatum‐derived EVs to distribute throughout the brain and transfer functional cargo.

### CC2.4 Interneuronal exchange and functional integration of synaptobrevin via extracellular vesicles

Natali L. Chanaday , Department of Pharmacology ‐ Vanderbilt University

Alejandro Vilcaes, CIQUIBIC‐CONICET Depto. Quimica Biologica Fac. de Ciencias Quimicas Pabellon Argentina Universidad Nacional de Cordoba

Ege T. Kavalai, Professor & Acting Chair, Department of Pharmacology, William Stokes Chair In Experimental Therapeutics, Vanderbilt University Department of Pharmacology

**Introduction**: In the past few years the role of extracellular vesicles (EVs) as short and long distance messengers has grown considerably. We now know that these secreted vesicles mediate the interchange of genomic materials, proteins and lipids in numerous tissues and can change the phenotype of the target cells. However, the functions of EVs in the nervous system and the underlying molecular mechanisms are only starting to be comprehended. The goal of the present work was to understand the putative role of EVs in neurotransmission from a molecular neuroscience perspective.

**Methods**: We isolated EVs from glia‐free dissociated rat and mouse hippocampal neurons. EVs were characterized by proteomics, electron microscopy and nanoparticle tracking analysis. We then studied the impact of EVs on neuron physiology using a combination of live fluorescence imaging and electrophysiology. Studies were complemented with Western blots, dot blots and localization analysis via confocal microscopy.

**Results**: We found that neuronal EVs contain several neuron‐specific proteins, including synaptic vesicle proteins that regulate the release of neurotransmitters, among them synaptobrevin‐2 (syb2). Syb2 from EVs can be functionally incorporated into synaptic vesicles in the target neurons, leading to a selective, calcium‐dependent increase in inhibitory neurotransmission. Syb2 recruitment to EVs is dependent on the tetraspanin CD81. Moreover, EVs containing syb2 can partially rescue spontaneous neurotransmission in syb2 knock‐out neurons. Conversely, EVs from hippocampal astrocytes, which lack syb2, have no effect on spontaneous neurotransmission.

**Summary/Conclusion**: These findings shed light on the molecular underpinnings of a novel form of interneuronal communication mediated by EVs and suggest the trafficking routes of EVs and synaptic vesicles may be interconnected.

### CC2.5 Monitoring of the effect neutral sphingomyelinase 2 inhibition on neural extracellular vesicles release by surface plasmon resonance imaging

Silvia Picciolini , IRCCS Fondazione Don Carlo Gnocchi

Carolyn Tallon, PhD, Johns Hopkins University School of Medicine

Cristiano Carlomagno, IRCCS Fondazione Don Carlo Gnocchi

Alice Gualerzi, PhD, IRCCS Fondazione Don Carlo Gnocchi ONLUS

Seung‐Wan Yoo, Johns Hopkins University School of Medicine

Ajit G. Thomas, Johns Hopkins University School of Medicine

Arindom Pal, Johns Hopkins University School of Medicine

Jesse Alt, Johns Hopkins University School of Medicine

Norman J. Haughey, Johns Hopkins University School of Medicine

Rana Rais,Johns Hopkins University School of Medicine

Barbara S. Slusher, Johns Hopkins University School of Medicine

Marzia BedoniIRCCS Fondazione Don Carlo Gnocchi

**Introduction**: Considering the implication of EVs in the spread of pathogenic proteins, inhibitors of Neutral Sphingomyelinase 2 (nSMase2), an enzyme critical in EV biogenesis, have been studied as therapeutic agents for several diseases. We recently discovered phenyl(R)‐(1‐(3‐(3,4‐dimethoxyphenyl)‐2,6‐dimethylimidazo[1,2‐b]pyridazin‐8‐yl)pyrrolidin‐3‐yl)carbamate (PDDC), the first potent, selective, orally‐available, and brain penetrable nSMase2 inhibitor, capable of reducing EV release in vitro and in vivo. Herein, we used Surface Plasmon Resonance imaging (SPRi) to evaluate which specific brain cell‐derived EVs were affected by PDDC in response to a neuroinflammatory insult.

**Methods**: Mice fed vehicle or PDDC‐containing chow were administered an intra‐striatal IL‐1β injection and 2h later their plasma were collected. EVs were isolated from plasma by size‐exclusion chromatography and different brain‐derived EVs were detected on a SPRi‐based biosensor by probing specific EV membrane molecules.

**Results**: IL‐1β‐induced injury selectively increased the levels of plasma EVs derived from CD171+ neurons and PLP1+ oligodendroglia that were normalized by PDDC, while GLAST+ astrocyte‐derived EVs were unchanged. IL‐1β injection increased the amount of EVs released from activated, CD11b+ microglia compared to EVs released from non‐activated microglia in plasma. The increase in EVs from activated microglia was normalized with PDDC.

**Summary/Conclusion**: We found that PDDC was able to normalize the increased release of neuronal‐, oligodendrocyte‐ and activated microglial‐derived EVs into plasma following an acute brain injury. These data support nSMase inhibition as a therapeutic strategy for acute brain injury, the use of circulating brain‐derived EVs as indicators of neuroinflammation status and the use of SPRi to evaluate the efficacy of therapeutics.

## CC3 Advances in EV Engineering and Characterization

Chair: Carolina Soekmadji, QIMR Berghofer Medical Research Institute, Australia

Chair: Navneet Dogra, Department of Genetics and Genomics Sciences, Icahn School of Medicine at Mount Sinai, United States

### CC3.1 Crosslinking mass spectrometry reveals the EV‐surface protein composition, structural terrain and interactome

Julia Bauzá‐Martinez, Biomolecular Mass Spectrometry and Proteomics, Bijvoet Center for Biomolecular Research and Utrecht Institute for Pharmaceutical Sciences, Utrecht University, The Netherlands

Gadi Armony, Utrecht University

Albert J.R. Heck, Utrecht University

Wei Wu, Biomolecular Mass Spectrometry and Proteomics, Bijvoet Center for Biomolecular Research and Utrecht Institute for Pharmaceutical Sciences, Utrecht University, The Netherlands

**Introduction**: EVs mediate key processes like intercellular communication and immune activation. EVs can directly trigger T‐cell activation by forming immunological synapses with the TCR, or indirectly after uptake by APCs. Although membrane proteins are involved in most of EV‐mediated processes, the study of such proteins remains challenged by technical limitations. In crosslinking mass spectrometry (XL‐MS), a chemical crosslinker is used to covalently link surface residues of proteins in close‐proximity, i.e., < 30Å. Yet, XL‐MS relies on using lots of material, a key limitation when working with EVs. Here, we overcome this limitation by using a highly efficient crosslinker (disuccinimidyl suberate; DSS) and a fractionation strategy to enrich for crosslinked peptides. We apply this to study the surface interactome of intact B‐cell derived EVs.

**Methods**: EVs from JY cells were isolated by ultracentrifugation (UC). Intact EVs were reconstituted in PBS and crosslinked with DSS for 10 min. Crosslinked EVs were lysed and proteins were trypsin‐digested. Crosslinked peptides were enriched by strong cationic exchange fractionation before LC‐MS analysis. Data was searched against a tailored database containing abundant proteins of the EV proteome. EV purity and composition were characterized by multiple techniques, including NTA, TEM and bottom‐up proteomics.

**Results**: Isolated EVs ranged between 30 ‐ 200 nm as shown by NTA and TEM, and appeared intact after UC. Compared to source cells, EVs were highly enriched in EV markers and membrane proteins, including CD81 and MHC‐I. By XL‐MS we identified ∼1,000 crosslinks from EVs, simultaneously mapping several membrane and EV‐specific protein interactions. The large matrix of distance restrains generated not only describes the EV‐surface terrain but can also be used to make structural models. Finally, we contrasted EV‐specific interactions to the JY cell surface interactome, with focus on supramolecular MHC complexes.

**Summary/Conclusion**: Here, we demonstrate the feasibility of using XL‐MS to study the EV‐interactome. By selecting a suitable crosslinker and enriching for crosslinked species, we boosted sensitivity overcoming one of the main limitations of working with EVs. We envision this approach will be highly suitable for EV‐surface interactome profiling, to aid in rationalizing membrane fusion and uptake processes in recipient cells.

### CC3.2 A novel enzymatic surface functionalization approach for generating engineered extracellular vesicles for targeted drug delivery

Migara K. Jayasinghe , Department of Pharmacology, Yong Loo Lin School of Medicine, National University of Singapore

Tin C. Pham, Department of Biomedical Sciences, College of Veterinary Medicine and Life Sciences, City University of Hong Kong

Thach T. Pham, Department of Pharmacology, Yong Loo Lin School of Medicine, National University of Singapore

Yuqi Yang, Department of Biomedical Sciences, College of Veterinary Medicine and Life Sciences, City University of Hong Kong

Marco Pirisinu, Department of Biomedical Sciences, College of Veterinary Medicine and Life Sciences, City University of Hong Kong

Boya Peng, Department of Pharmacology, Yong Loo Lin School of Medicine, National University of Singapore

Jiahai Shi, Department of Biomedical Sciences, College of Veterinary Medicine and Life Sciences, City University of Hong Kong

Minh T. Le, Department of Pharmacology, Yong Loo Lin School of Medicine, National University of Singapore

**Introduction**: Given their propensity for intercellular communication and their excellent biocompatible profile, extracellular vesicles (EVs) have been viewed for many years as promising vectors for drug delivery. However, endogenous EVs are often lacking in their target specificity leading to the incorporation of artificial modifications that elevate their therapeutic potential. Thus far, most studies on EV surface engineering have relied on slight variations of four principle approaches ‐ genetic engineering of cells, lipid insertion, affinity interactions and chemical conjugation. While demonstrating impressive functionality in some cases, they tend to be tedious and inefficient due to their reliance on methods such as genetic manipulation, transient interactions and harsh chemical treatments. Here we describe a novel approach for the surface functionalization of EVs based on enzymatic ligation.

**Methods**: Via the use of protein ligases, we are able to efficiently and site‐specifically conjugate a desired targeting molecule of choice at high copy number onto pre‐existing EV membrane proteins via the formation of covalent peptide bonds. The approach is biocompatible, requires no genetic manipulation and has no effect on EV integrity or physicochemical characteristics.

**Results**: Conjugation of targeting peptides and antibodies onto the EV surface enables efficient and specific delivery of encapsulated drugs to target cells expressing corresponding receptors such as EGFR, EpCAM and CXCR4. Furthermore, we demonstrate tumor specific delivery of EV‐encapsulated chemotherapeutics, peptides and RNA drugs upon systemic administration in multiple mouse models of cancer, including a lung cancer xenograft, leukemia and breast cancer. Targeted delivery of encapsulated therapeutics leads to significantly better tumor suppression and improved treatment outcomes in vivo. We also demonstrate the ability to target other receptors such as SIRP‐α to enhance anti‐phagocytic properties of EVs.

**Summary/Conclusion**: We present here a novel method of surface functionalization that improves upon the efficiency and versatility of existing methods and preserves the endogenous biocompatible profile of EVs while concurrently utilizing a more clinically translatable approach by virtue of the non‐immunogenic, non‐mutagenic and stable nature of enzymatic ligation.

### CC3.3 On‐chip magnetic immuno‐extraction of small extracellular vesicles from human plasma

Monica Araya‐Farias , Curie Institute

Dario Brambilla, CNR ‐ SCITEC

Lucile Alexandre, McGill University

Laura Trapiella‐Alfonso, Chimie Paris Tech‐PSL University

Giacomo Gropplero, Curie Institute

Marine Verhursel, Fluigent‐Smart Microfluidics

William Cesar, Fluigent‐Smart Microfluidics

Marcella Chiari, National Research Council of Italy ‐ Institute of Chemical Sciences and Technologies (CNR ‐ SCITEC)

Than‐Duc Mai, Université Paris‐Saclay

Marco Morani,Université Paris‐Saclay

Stéphanie Descroix, Curie Institute

**Introduction**: Extracellular vesicles (EVs) have emerged over the past years as the new biomarkers of the future for non‐invasive disease diagnostics, sometimes called as the new Hermes biomarkers as they will provide a large quantity of information. However, to exploit their potential in human diagnostics, a fast, high‐throughput and reproducible method is needed for their extraction and analysis. Here, we present an original microfluidic approach that combines immuno‐extraction and fluidized bed (FB) technology to isolate EVs directly from human plasma allowing the capture and release of EVs in a single device.

**Methods**: We have developed a miniaturized and microfluidic fluidized bed in which magnetic and drag forces are balanced to keep in suspension antibody‐functionalized magnetic beads in a triangular shaped‐chamber during sample perfusion. Beads were functionalized with EVs‐specific antibodies (AntiCD63 or AntiCD9) by using a novel DNA‐directed immobilization (DDI) strategy. A low"cost FB microfluidic platform was developed to carry out the experiments in a completely automated manner.

**Results**: We evaluated the effect of the flow rate ranging from 0.5 to 5μL/min to tune the residence time of the sample in the chip in order to achieve the highest efficiency. EVs were extracted from human plasma with around 80% of capture efficiency. We demonstrated thanks to Nano Tracking Analysis (NTA) that our microfluidic strategy allowed to capture and to release significantly a large number of EVs (40% higher than an experiment performed in tube) with a size between 100 to 200 nm. This integrated and automated approach enabled the release of intact vesicles which was successfully confirmed by imaging techniques such as Transmission Electron Microscopy (TEM) and Cryo‐Electron Microscopy as well as Capillary Electrophoresis Coupled Laser Induced Fluorescent Detection (CE‐LIF).

**Summary/Conclusion**: We demonstrated the feasibility of our fluidized bed approach to efficiently extract small EVs from a complex biological matrix. Further experiments will be performed to assess how these fluidized bed devices can be sequentially coupled to extract different EVs subpopulations.

### CC3.4 AFM‐based high throughput mechanical characterization of single EVs from different natural sources

Andrea Ridolfi, Dipartimento di Chimica “Ugo Schiff”, Università degli Studi di Firenze, 50019 Firenze, Italy

Marco Brucale, Consiglio Nazionale delle Ricerche, Istituto per lo Studio dei Materiali Nanostrutturati, 40129 Bologna, Italy

Costanza Montis, Dipartimento di Chimica “Ugo Schiff”, Universitàdegli Studi di Firenze, 50019 Firenze, Italy

Lucrezia Caselli, Dipartimento di Chimica “Ugo Schiff”, Università degli Studi di Firenze, 50019 Firenze, Italy

Lucia Paolini, Dipartimento di Medicina Molecolare e Traslazionale, Università degli Studi di Brescia, 25123 Brescia, Italy

Anne Borup, Department of Clinical Medicine, Faculty of Health, Aarhus University, 8200 Aarhus, Denmark

Anders Toftegaard Boysen, Department of Clinical Medicine, Faculty of Health, Aarhus University, 8200 Aarhus, Denmark

Francesca Loria, HansaBioMed Life Sciences OÜ, Mäealuse 2/1, 12618 Tallinn, Estonia

Martijn van Herwijnen, Department of Biomolecular Health Sciences, Faculty of Veterinary Medicine, Utrecht University, The Netherlands.

Marije Kleinjan,Department of Biomolecular Health Sciences, Utrecht University, The Netherlands

Peter Lindberg Nejsum, Department of Clinical Medicine, Faculty of Health, Aarhus University, 8200 Aarhus, Denmark

Natasa ZarovniHansaBiomed Life Sciences, 12618 Tallinn, Estonia

Marca H.M. H.M. Wauben, Department of Biomolecular Health Sciences, Utrecht University, The Netherlands

Debora Berti,Dipartimento di Chimica “Ugo Schiff”, Università degli Studi di Firenze, 50019 Firenze, Italy

Paolo Bergese,Dipartimento di Medicina Molecolare e Traslazionale, Università degli Studi di Brescia, 25123 Brescia, Italy

Francesco Valle,Consiglio Nazionale delle Ricerche, Istituto per lo Studio dei Materiali Nanostrutturati, 40129 Bologna, Italy

Francesco Valle,Consiglio Nazionale delle Ricerche, Istituto per lo Studio dei Materiali Nanostrutturati, 40129 Bologna, Italy

**Introduction**: The mechanical properties of extracellular vesicles (EVs) are known to influence their biological function in terms of e.g. cellular adhesion, endo/ exocytosis, cellular uptake, and mechanosensing. Moreover, these characteristics provide a significant contribution to the heterogeneity that affects different EV subtypes, which is one of the main issues hindering the application of EVs in multiple biomedical fields. EVs nanomechanics can be studied by Atomic Force Microscopy‐based Force Spectroscopy (AFM‐FS); however, the low throughput and the need for dedicated instrumentation usually limit this approach to specialists, making it unable to address the demand of the EV community for a simple and rapid mechanical characterization.

**Methods**: In the attempt of providing a solution to this open issue, we herein present a simple AFM‐based high throughput characterization method that allows for the simultaneous nanomechanical and morphological analysis of several hundred individual nanosized EVs within the hour time scale (Ridolfi A. et al, 2020, Anal. Chem. 92:10274"10282). The procedure is based on the measurement of the contact angle displayed by each vesicle, upon adsorption on a surface.

**Results**: Results suggest that the contact angle can be regarded as a mechanical fingerprint of the typical “vesicle‐like” behavior, allowing for the detection of contaminants within an EV sample and for rapidly assessing the presence of EVs even in samples for which no established assays and/or commercial kits are available. Moreover, we show that a vesicle's contact angle is directly related to its stiffness as measured by AFM‐FS, and can hence be used to obtain a high throughput mechanical characterization of single EVs from different populations.

**Summary/Conclusion**: In this framework, we present our latest results regarding the mechanical characterization of multiple EV samples coming from distinct natural sources, thereby demonstrating the applicability of the method to a wide variety of EV subtypes and its ability to map EV samples’ heterogeneities, which remain inaccessible to most of the currently used bulk characterization techniques.

### CC3.5 Novel strategies to increase efficacy and to overcome drug resistance in BRAF‐driven tumours

Adrián Varela. Varela‐Vázquez , CellCOM Research Group. Research Biomedical Institute of A Coruña (INIBIC)

Amanda Guitián‐Caamaño, CellCOM Group, Instituto de Investigación Biomédica de A Coruña (INIBIC), Servizo Galego de Saúde (SERGAS), Universidade da Coruña (UDC)

Paula Carpintero‐Fernández, CellCOM Group, Instituto de Investigación Biomédica de A Coruña (INIBIC), Servizo Galego de Saúde (SERGAS), Universidade da Coruña (UDC).

Marta Varela‐Eirín, European Research Institute for the Biology of Ageing (ERIBA);, University Medical Center Groningen (UMCG), University of Groningen.

Susana B. Bravo‐López, Instituto de Investigación Sanitaria de Santiago de Compostela IDIS, Proteomics laboratory.

Teresa Calleja‐Chuclá, Hospital Pharmacy Service. CH‐Universitario A Coruña (XXIAC). Servizo Galego de Saúde (SERGAS). Universidade da Coruña (UDC).

María Quindós‐Varela, Translational Cancer Research Group, Instituto de Investigación Biomédica de A Coruña (INIBIC). CH‐Universitario A Coruña (XXIAC). Servizo Galego de Saúde (SERGAS). Universidade da Coruña.

David Santamaría, University of Bordeaux, INSERM U1218, ACTION Laboratory, IECB.

Eduardo Fonseca, Dermatology Deparment; CellCOM Group, Instituto de Investigación Biomédica de A Coruña (INIBIC), Servizo Galego de Saúde (SERGAS), Universidade da Coruña (UDC).

María D. Mayán,CellCOM Group, Instituto de Investigación Biomédica de A Coruña (INIBIC), Servizo Galego de Saúde (SERGAS), Universidade da Coruña (UDC).

**Introduction**: Connexin43 (Cx43) has been described as a tumor suppressor in primary melanoma, but its role in disease progression is still under debate. Small extracellular vesicles (sEVs) containing Cx43 allow the exchange of small molecules such as small RNAs (sRNAs) via gap junction channels. BRAF/MEK inhibitors (BRAF/MEKi) have become the standard therapy in patients with BRAF‐mutated melanoma. However, resistance to therapy often develops within 12 months. Drug resistance continues to be a major problem for totally efficient to this therapy.

**Methods**: Expression vectors and sEVs‐enriched in Cx43 were used to increase Cx43 activity in tumor cells. sEVs were isolated by ultracentrifugation and characterized by NTA, electron microscopy and WB. IP was performed to study protein interactions and analyzed by LC‐MS/MS. RNA‐seq was used to identified sRNAs. Standard methods were used to study cellular senescence and apoptosis.

**Results**: Cx43 overactivity decreases proliferation and increases senescence and apoptosis in BRAF mutant cells in absence/presence of BRAF/MEKi. sEVs‐positive for Cx43 radically changes their function and the content of proteins and smallRNA, indicating that Cx43 may participate in the recruitment of proteins and sRNA. Also, restoration of Cx43 using sEVs, in BRAF‐mutated tumors, increases the efficacy of the BRAF/MEKi, and prevents drug resistance by reinforcing senescence and enhancing apoptosis alone and in combination with the senolytic drug navitoclax.

**Summary/Conclusion**: We have identified a new therapeutic target for the treatment of BRAF‐mutated tumors and to overcome drug resistance to BRAF/MEKi based on the combination of sEVs containing Cx43 along with BRAF/MEKi which increases in more than 80% inhibitors efficacy. We have demonstrated that sEVs can be used as drug carrier to transport transmembrane proteins such as Cx43. Our results could impact in the manage and treatment of metastatic tumors with a potential clinical benefit in patients with a metastatic disease.

## CC4 At the Heart of EVs

Chair: Antonella Bongiovanni, Institute for Biomedical Research and Innovation, National Research Council of Italy, Italy

Chair: Michael Davis, Georgia Institute of Technology and Emory University, United States

### CC4.1 Determining the Timing and Mechanisms of Cardiac Recovery by Extracellular Vesicles secreted by Induced Pluripotent Stem Cell Derived Cardiomyocytes

Bryan Z. Wang, M.S., Columbia University

Bohao Liu, Columbia University

Trevor Nash, Columbia University

Xiaokan Zhang, Columbia University

Lori luo, Columbia University

Manny Tamargo, Columbia University

Sharon Fleischer, Columbia University

Roberta Lock, Columbia University

Gordana Vunjak‐Novakovic, Columbia University

**Introduction**: Extracellular vesicles from induced pluripotent stem cell derived cardiomyocytes (iCM‐EV) have potential for treating myocardial infarction (MI). The efficacy of subacute treatment with iCM‐EV has not been studied. Also, the mechanisms of their action in human tissue are unknown.

**Methods**: Studies were done in a rat model of MI, and in human cell and tissue models of ischemia. To induce MI, rats underwent left anterior descending artery ligation. iCM‐EV laden patches with sustained release of EVs were implanted on the infarct zone immediately after MI induction, or 24 hours post‐MI. Echocardiography and histological analysis of hearts were performed after four weeks. In vitro, iCM were injured with hypoxia, treated with EVs, and evaluated by RNA sequencing. Western Blot and qPCR were used to evaluate iCMs. Physiologically matured human engineered cardiac tissues (ECT) were generated using our previously established methods, injured with hypoxia, treated with EVs, and evaluated by calcium imaging (for function) and qPCR.

**Results**: iCM‐EV significantly reduced the infarct size and increased cardiac function when implanted immediately after ligation. Delaying treatment by 24h reduced therapeutic benefit. RNA sequencing and protein analysis showed increased anti‐apoptotic and pro‐inflammatory signatures in EV‐treated cardiomyocytes. Activation of epidermal growth factor receptor (EGFR) signaling was identified as a key pathway affected by EV treatment. Analysis of iCM‐EV cargo showed an abundance of EGFR in EVs. iCM‐EVs upregulated EGFR phosphorylation, concomitant with increased pro‐survival ERK1/2 activity. In ECT, iCM‐EV significantly altered tissue calcium handling. qPCR confirmed increases in pro‐inflammatory and anti‐apoptotic gene expression.

**Summary/Conclusion**: Acute, but not subacute, treatment with iCM‐EVs was effective in treating MI, underscoring their proinflammatory and anti‐apoptotic effects which are potentially mediated through activation of EGFR signaling.

### CC4.2 MicroRNA‐30 is a potential circulating biomarker for dysregulated microvascular endothelial cell function and metabolism in heart failure with preserved ejection fraction

Shawn C. Veitch , University Health Network / University of Toronto

Makon‐Sebastien Njock2Makon‐Sebastien Njock, University Health Network

Mark Chandy, University Health Network

M. Ahsan Siraj, University Health Network

Zhiqi Chen, University Health Network

Lijun Chi, The Hospital for Sick Children

Faisal Alibhai, University Health Network

Dakota D. Gustafson, Toronto General Hospital Research Institute

Dorrin Zarrin Khat, University Health Network / University of Toronto

Patrick Meagher,St. Michael's Hospital

Henry S. Cheng, University Health Network / University of Toronto

Kim ConnellySt. Michael's Hospital

Paul Delgado‐Olguin, The Hospital for Sick Children

Mansoor Husain,University Health Network

Jason E. Fish, PhD,Toronto General Hospital Research Institute

**Introduction**: Type 2 diabetes (T2D) is associated with cardiac microvascular dysfunction, which can contribute to the development of diastolic dysfunction and heart failure with preserved ejection fraction (HFpEF). The molecular mechanisms responsible for HFpEF remain unclear, and no effective diagnostics or treatments are available.

**Methods**: T2D mouse (db/db) and rat (Goto‐Kakizaki) models were used in this study. The presence of HFpEF was assessed using echocardiography and pressure‐volume loop analysis. The microRNA content of circulating extracellular vesicles (EVs) during the pathogenesis T2D‐associated HFpEF was measured using a RT‐qPCR microRNA array. EV concentration and size was measured using NanoSight NS300 Nanoparticle Tracking Analysis (NTA). Finally, RT‐qPCR was used to assess the role of miR‐30 in endothelial cells (ECs) at a molecular level. Seahorse and fluorometric assays were used to assess the role of miR‐30 in EC metabolism.

**Results**: We found that miR‐30d and miR‐30e were increased in EVs prior to echocardiographic evidence of diastolic dysfunction. These microRNAs may serve as biomarkers of microvascular dysfunction as they are upregulated in the endothelial cells (ECs) of the left ventricle of the heart, but not other organs. Furthermore, the induction of the miR‐30 family in ECs is regulated by senescence, a characteristic feature of diabetic ECs. Assessment of pathways regulated by miR‐30d/e revealed a large number of target genes involved in fatty acid metabolism. Importantly, over‐expression of miR‐30e in ECs increased exogenous fatty acid oxidation and the production of reactive oxygen species, while inhibiting the miR‐30 family had the opposite effect. Additionally, miR‐30e over‐expression synergized with fatty acid exposure to dramatically down‐regulate the expression of eNOS, an important regulator of microvascular function.

**Summary/Conclusion**: Circulating miR‐30d/e may represent early biomarkers of diastolic dysfunction that reflect altered fatty acid metabolism and microvascular dysfunction in the heart. Furthermore, the pathways regulated by miR‐30 may represent therapeutic targets for diabetes‐associated HFpEF.

### CC4.3 Pluripotency‐associated miRNAs underlie the superior in vitro bioactivity of human induced pluripotent stem cell (hiPSC)‐derived extracellular vesicles in mediating cardiac repair

Ana Filipa F. Louro, iBET; ITQB‐NOVA

Marta Paiva, iBET; ITQB‐NOVA

Marta Oliveira, iBET

Patrícia Gomes‐Alves, iBET; ITQB‐NOVA

Paula Alves, iBET; ITQB‐NOVA

Margarida Serra, iBET; ITQB‐NOVA

**Introduction**: Studies on the cardiac repair potential of Extracellular Vesicles (EV) traditionally use mesenchymal or cardiac progenitor cell derived EV. In this study, we isolated EV from key stages of the hiPSC‐cardiomyocyte (hiPSC‐CM) differentiation and maturation, i.e. from hiPSC (hiPSC‐EV), cardiac progenitors (CPC‐EV), immature (CMi‐EV) and mature (CMm‐EV) cardiomyocytes, with the aim of identifying an efficient cell biofactory for therapeutic EV production, and selecting miRNA candidates for cardiac regeneration.

**Methods**: hiPSC were differentiated into hiPSC‐CM and matured in 3D culture, according to in‐house protocols. EV were separated by density, and characterized in terms of expression of specific EV‐associated markers, yield, particle size and particle size distribution. Bioactivity was assessed in human umbilical vein endothelial cells (HUVEC) and hiPSC‐CM based on EV uptake and its effect on angiogenesis, cell migration and proliferation. Small RNA‐Seq was performed to identify differentially expressed miRNA in the four EV groups.

**Results**: Bioactivity assays showed increased tube formation and migration in HUVEC treated with hiPSC‐EV compared to EV from committed cell populations (p < 0.0001). hiPSC‐EV also increased hiPSC‐CM proliferation (p < 0.01). Small RNA‐Seq analysis identified 15 miRNAs differentially expressed along hiPSC‐CM differentiation and maturation. A cluster involved in stemness maintenance was highly expressed in hiPSC, and gradually repressed along CM differentiation. These miRNAs were found to target the PI3/AKT pathway and were transfected into HUVEC and hiPSC‐CM to further investigate their role in cardiac repair.

**Summary/Conclusion**: Our findings suggest a superior in vitro bioactivity for hiPSC‐derived EV possibly mediated by pluripotency associated miRNA, intrinsically involved in cell proliferation processes.

### CC4.4 Coronary artery disease ameliorates extracellular vesicle lncRNA PUNISHER regulates angiogenic response and endothelial cells function via NFkB‐dependent mechanism

Mohammed Rabiul Hosen , University of Bonn

**Introduction**: Augmenting evidence indicates that long noncoding RNAs are playing a crucial role in diverse cellular/pathological processes. Intercellular transfer of extracellular vesicles transmitted lncRNA regulates vascular health and diseases. However, whether lncRNA expression in EVs is regulated in patients with coronary artery disease, is unknown.

**Methods**: A PCR‐based lncRNA array analysis revealed that EV‐PUNISHER was significantly upregulated in patients with CAD (n = 221) compared to healthy subjects. To examine the specific role of PUNISHER in EC phenotypic regulation, siRNA‐mediated silencing in ECs revealed that depletion of PUNISHER suppresses the migration and proliferation of ECs. Depletion of PUNISHER decreases cell survival by reducing cell viability and proliferation through increased apoptosis and cytotoxicity. To investigate EC function in PUNISHER depleted cells, sprouting and tube formation assay revealed that PUNISHER is an important mediator EC functions. In vitro atherosclerotic stimuli (OxLDL, TNF‐α, IL‐6) increased PUNISHER expression in EV/EC in a dose‐dependent way. Microarray analysis identified a series of pro‐ and anti‐angiogenic genes as well genes directly involved in cell viability that are differentially regulated.

**Results**: We confirmed that PUNISHER is incorporated into endothelial microvesicles (EMVs) and transferred to recipient cells by using different experiments. By using lncRNA‐FISH and vesicle degradation assays, we showed that PUNISHER is incorporated into EMVs, augmented recipient EC function in vitro upon transfer via EMVs. To examine whether EMV‐PUNISHER promotes the EC function, different in vitro functional experiments with ECs (tube formation, angiogenic sprouting, migration, proliferation, apoptosis, etc.) confirmed that PUNISHER is an important regulator. Mass spectrometry analysis has revealed EMV contains numerous proteins, including RNA binding proteins such as hnRNPU, hnRNPK, hnRNPA2/B1, etc. By using RNA‐pulldown and RNA immunoprecipitation (RIP), we confirmed that PUNISHER interacts with hnRNPU, which facilitates packaging into EMV prior to transfer to recipient EC. The interaction acts as an important positive regulator of cell viability and survival of recipient cells, identified using functional assay (migration, viability, proliferation, and angiogenesis). The transcription factor array has shown that PUNISHER regulates NFkB to control cellular viability and apoptosis. A murine re‐endothelialization model after injection of EVs or ncRNAs revealed that EV‐PUNISHER promotes reendothelialization.

**Summary/Conclusion**: Our study unveiled that EV‐incorporated PUNISHER exerts its function in ECs which might be beneficial in cardiovascular pathologies

### CC4.5 Engineering small extracellular vesicle‐like vehicles carrying pro‐reparative microRNA for cardiac repair after myocardial infarction

Sruti Bheri , Georgia Institute of Technology and Emory University

Michael E. Davis, Ph.D., Georgia Institute of Technology and Emory University

**Introduction**: Small extracellular vesicles (sEVs) are released by ckit+ progenitor cells (CPCs) for cardiac repair after myocardial infarction (MI). These sEVs carry microRNA (miR) cargo and have a complex lipid membrane which is associated with efficient uptake. However, as sEVs are secreted by cells, we have little control over sEV yield, the cargo loaded and its concentration. To address this, synthetic sEV mimics allowing custom cargo loading have been developed. However, they do not have the sEV's complex membrane, often having high toxicity and lower uptake. Here, we've designed sEV‐like vehicles (ELVs) to combine the benefits of both sEVs and mimics, by containing an sEV‐derived membrane and allowing tailored cargo. Further, to test ELV functionality, we investigated its ability to deliver pro‐reparative miR cargo to cardiac cells.

**Methods**: sEVs were isolated from CPC conditioned media using differential ultracentrifugation. They were ruptured using freeze‐thaw cycling and sonication to remove inherent cargo. Thin film hydration was then used to create a uniform lipid film. Finally, ELVs were formed by rehydrating the film in the presence of pro‐reparative miR‐126 or ‐133 cargo. ELVs were then post‐processed to remove unbound miR and form sEV‐sized vehicles. ELV size and concentration were assessed with nanoparticle tracking analysis. For testing ELV functional response, miR‐126 ELVs were dosed to cardiac endothelial cells (CECs) and angiogenesis and proliferation were quantified. Finally, the anti‐fibrotic effects of miR‐133 ELVs were studied on rat cardiac fibroblasts (RCFs).

**Results**: ELVs were successfully created with selective miR encapsulated, although there was some sample loss during synthesis. Treatment of CECs with miR‐126 ELVs increased angiogenesis and proliferation over 48hr treatment, compared to sEV alone. Similarly, ELVs containing anti‐fibrotic miR‐133 reduced RCF fibrotic response in a dose dependent manner.

**Summary/Conclusion**: This study provides the groundwork for a potent and tailored cardiac miR carrier for MI, which mitigates the challenges with sEVs and sEV mimics. Moreover, given the extensive role of miRs as therapeutics, ELVs have scope to be used for cargo delivery in other cardiac diseases and beyond.

## CC5 EV Loading and Release

Chair: Alissa Weaver, Department of Cell and Developmental Biology, Vanderbilt University School of Medicine, United States

Chair: Guillaume Van Niel, IPNP INSERM U1266, France

### CC5.1 Intracellular sorting and EV‐mediated release of Y‐RNA and Y‐RNA binding proteins

Tom Driedonks, PhD , Johns Hopkins Medical School / Utrecht University

Sarah Ressel, Institute of Immunology & Infection Research, School of Biological Sciences, University of Edinburgh, Edinburgh, United Kingdom

Thi Tran, Dept. Biomolecular Health Sciences, Fact. Veterinary Medicine, Utrecht University, Utrecht, The Netherlands

Amy H. Buck, Institute of Immunology & Infection Research, School of Biological Sciences, University of Edinburgh, Edinburgh, United Kingdom

Esther N.M Nolte – ‘t Hoen, Dr., Utrecht University

**Introduction**: Y‐RNA is a non‐coding RNA that is abundantly present in EV from various cell types and biofluids such as plasma. EV‐associated Y‐RNA has been associated with immune‐regulatory functions. We previously showed that Toll‐like receptor (TLR) activation of primary immune cells alters the abundance of Y‐RNA in EV, but not in the cytoplasm, suggesting that Y‐RNA shuttling is a regulated process. Y‐RNA interacts with various RNA‐binding proteins (RBP) in cells. Using subcellular fractionation of THP1 macrophages, we investigated how activation‐induced changes in intracellular sorting of Y‐RNA and their RBP relate to enhanced release of Y‐RNA in EV.

**Methods**: Post‐nuclear cytosol of TLR2/1‐stimulated and unstimulated THP1 was separated on Optiprep gradients into fractions enriched in ER, Golgi and endo/lysosomes. Y‐RNA and RBP in subcellular fractions and EV were analyzed in parallel by Western blot, RT‐qPCR and Northern blot.

**Results**: Full‐length Y‐RNA was mainly detected in subcellular fractions containing endo/lysosomal markers Lamp‐1, CD63 and Tsg101. Y‐RNA binding proteins Ro60, La, HuR and hnRNP K, but not YBX1, co‐localized with Y‐RNA in these fractions. This supported the idea that Y‐RNA can be sorted into EV at multivesicular endosomes (MVE). Of the tested RBP, only Ro60 was detected in Y‐RNA containing EV. While TLR stimulation did not affect cytoplasmic Y‐RNA levels, we observed an increase in the local Y‐RNA concentration in MVE. This was paralleled with a TLR stimulation‐induced increase in Y‐RNA and Ro60 incorporation into EV.

**Summary/Conclusion**: Our data exemplify how parallel analysis of subcellular compartments and EV can be used to study cell stimulation‐induced changes in the sorting of EV‐associated RNAs and their interacting proteins. We demonstrate that full‐length Y‐RNA and a subset of Y‐RNA binding proteins are enriched at EV biogenesis sites. Our results suggest that TLR‐activation changes the incorporation of Y‐RNA into EV by altering its local concentration at EV‐biogenesis sites.

### CC5.2 Unconventional protein trafficking for extracellular vesicle packaging and function

Petra Parać, Metabolic Research Laboratories, Wellcome Trust‐Medical Research Council Institute of Metabolic Science, University of Cambridge, Cambridge, UK

Anna Albecka, Medical Research Council Laboratory of Molecular Biology, Cambridge, UK

Stephanie J. Popa, Metabolic Research Laboratories, Wellcome Trust‐Medical Research Council Institute of Metabolic Science, University of Cambridge, Cambridge, UK

Julien Villeneuve, Cambridge Institute for Medical Research, University of Cambridge, Cambridge, UK

Suresh Mathivanan, Department of Biochemistry and Genetics, La Trobe Institute for Molecular Science, La Trobe University, Melbourne, VIC 3083, Australia

Sarah E. Stewart, Department of Biochemistry and Genetics, La Trobe Institute for Molecular Science, La Trobe University, Melbourne, VIC 3083, Australia

Australia

**Introduction**: The current model for EV biogenesis dictates that the subcellular location of proteins is reflected in the EV architecture, with cytosolic proteins in the lumen and cell surface proteins on the surface of EVs. This is irrespective of whether the EVs originate from the plasma membrane or are formed in the multivesicular body. However, myself and others have reported that cytosolic proteins, such as annexin A2, are reside on the surface of EVs. This is not entirely surprising, we have also shown that annexins are primarily secreted from cells via type I unconventional protein secretion, directly crossing the plasma membrane. This suggests that cytosolic proteins, including annexin A2, reside on the cell surface and hence the surface of EVs.

**Methods**: To build on these observations, we are investigating EV architecture and protein packaging using a proteomic approach to identify proteins known to be unconventionally secreted on the surface of EVs by tryptic digest. Candidates of interest are further investigated for roles in EV biogenesis and extracellular function using gene knockdown and CRISPR/Cas9 knockout, imaging and biochemical approaches.

**Results**: Several proteins known to be secreted by unconventional protein secretion are identified on the surface of EVs. Surprisingly other cytosolic proteins, not known to be secreted by cells are also detected on the surface of EVs. Proteins of interest were assessed for their role in EV function, such as binding and uptake in recipient cells. At least one EV surface protein was found potentially mediate EV uptake by recipient cells. Additionally, we investigated the role of several candidates in the regulation of EV biogenesis. Again, we identified one surface protein appears to regulate EV biogenesis or secretion.

**Summary/Conclusion**: The concept of cytosolic proteins residing on the surface of EVs is important for understanding the extracellular functions of EVs, including uptake and fusion which is likely to be mediated by proteins on the surface of EVs and cells. This data adds to the body of knowledge by which proteins are secreted through unconventional protein secretion and may identify new molecular pathways for packaging proteins onto the surface of EVs.

### CC5.3 Biogenesis of RNA‐containing extracellular vesicles at VAP‐A directed endoplasmic reticulum membrane contact sites

Bahnisikha Barman , Vanderbilt University

Jie Ping, Vanderbilt University Medical Center

Evan Krystofiak, Vanderbilt University

Ryan Allen, Vanderbilt University Medical Center

Nripesh Prasad, HudsonAlpha Institute for Biotechnology

Kasey Vickers, Vanderbilt University Medical Center

James G. Patton, PhD, Vanderbilt University

Qi Liu, Vanderbilt University

Alissa Weaver, Department of Cell and Developmental Biology, Vanderbilt University School of Medicine

**Introduction**: Promising data suggest that extracellular RNAs (exRNAs) can affect gene expression, function, and phenotypes of recipient cells. While several RNA binding proteins (RBPs) are known to carry RNAs into extracellular vesicles (EVs), where and how in the cell this occurs is unclear. Here, we identify VAP‐A positioned endoplasmic reticulum membrane contact sites (ER MCS) as key locations for the biogenesis of RNA‐containing EVs.

**Methods**: **Methods**: We used bioinformatics, RNA‐sequencing, lipidomic, confocal and transmission electron microscopy, tumor xenograft and various biochemical techniques to analyze EV biogenesis and cargo content in colon cancer cell lines molecularly engineered for molecules that control ER MCS (VAP‐A‐KD, VAP‐A OE, CERT‐KD, ORP1L‐KD).

**Results**:RNA‐Seq analysis revealed a number of small RNAs that are altered in VAP‐A KD small and large EVs compared to control cells. Density gradient fractionation revealed that VAP‐A regulates a select subpopulation of small EVs that are enriched with RNA. Furthermore, this VAP‐A‐controlled small EV population is critical for transfer of miR‐100 to recipient cells and for growth of xenograft mouse tumors. Lipidomics analysis of small and large EVs revealed that VAP‐A controls levels of ceramide and cholesterol, two lipids involved in EV biogenesis. Furthermore, KD of the VAP‐A binding ceramide and cholesterol transporters CERT and ORP1L led to similar defects in EV biogenesis.

**Summary/Conclusion**: **Summary/Conclusion**: We uncovered a novel pathway of EV biogenesis that takes place at ER MCS. These data suggest a model in which lipid transfer at ER MCS drives biogenesis of a select subpopulation of EVs containing RNA‐RBP complexes. Beyond improving our understanding of EV biogenesis, we anticipate that these findings may be useful for future engineering of therapeutic EVs as well as exploring the functions of RNA‐containing EVs.

### CC5.4 High‐throughput screening for the identification of drugs affecting secretion of two types of Extracellular Vesicles in breast cancer cells

Eleonora Grisard , Institut Curie U932

Aurianne Lescure, Istitut Curie Biophenics Laboratory

Maxime Corbé, Insitut Curie Biophenics Laboratory

Mercedes Tkach, Institut Curie / INSERM U932

Lorena Martin‐Jaular, Institut Curie / INSERM U932

Mathilde Mathieu, Insitut Curie U932

Grégory Lavieu, PhD, Université de Paris, inserm, umr7057/cnrs

Mabel Jouve, Institut Curie / CNRS UMR 3215

Elaine del Nery, Institut Curie Biophenics Laboratory

Clotilde Thery, MD PhD,Institut Curie / INSERM U932

**Introduction**: Eukaryotic cells, including cancer cells, secrete many different types of extracellular vesicles (EVs) as mediators of inter‐cellular communication. Secreted EVs can originate in different subcellular locations, resulting in populations highly heterogeneous in size, composition and function. Despite the most commonly used EV isolation techniques co‐isolate mixtures of these heterogeneous EVs, the specific mechanisms of secretion of the different EV subtypes are still largely unknown and tools to specifically modulate them are still lacking.

**Methods**: To analyze different EV subtypes, we first engineered MDA‐MB‐231 triple‐negative breast cancer cells to express constructs encoding for either CD63 or CD9 EV markers tagged with the Nanoluciferase (Nluc) enzyme. We then set up a high‐content screening assay to detect the secretion of Nluc‐CD63 or Nluc‐CD9 tagged EVs by quantifying Nluc activity in the supernatant of cells. We used a compound library of 1,280 FDA, EMA approved drugs (Prestwick Chemicals V3) to identify drugs able to modulate the secretion of Nluc‐CD63 or Nluc‐CD9 positive EVs. Finally, a panel of selected drugs depicted from the screening were used to further quantify EV release upon classical techniques of EV isolation and characterization (Size exclusion chromatography, Nanoparticle tracking analysis, Western blot, Electron microscopy).

**Results**: Our screening allowed the identification of several drugs modulating either Nluc‐CD63 cells or Nluc‐CD9 cells, or both. The hit validation step highlighted several potential misinterpretations due to unpredictable interaction between drugs and Nluc enzyme. After the establishment of stringent criteria to select drugs, we identified a new drug which robustly increases EV secretion in both Nluc‐CD63 and Nluc‐CD9 cells and we validated it also in the parental MDA‐MB‐231. Interestingly, this drug increases a EV subpopulation enriched in a novel combination of surface markers.

**Summary/Conclusion**: A novel EVs secretion multi‐drug high‐content screening allowed the identification of a never described drug able to increase the secretion of a specific EV subpopulation.

### CC5.5 Identification of the Wnt Signal Peptide that directs secretion in Extracellular Vesicles

Uxia Gurriaran , Ottawa Hospital Research Institute

David Datzkiw, 1 Regenerative Medicine Program, Ottawa Hospital Research Institute, Ottawa, Ontario, Canada. 2 Department of Cellular and Molecular Medicine, Faculty of Medicine, University of Ottawa, Ottawa, Ontario, Canada.

Leandro G. Radusky, Centre for Genomic Regulation (CRG), The Barcelona Institute for Science and Technology, Barcelona, Spain

Solomon Fisher, 1 Regenerative Medicine Program, Ottawa Hospital Research Institute, Ottawa, Ontario, Canada. 2 Department of Cellular and Molecular Medicine, Faculty of Medicine, University of Ottawa, Ottawa, Ontario, Canada.

Fan Xiao, 1 Regenerative Medicine Program, Ottawa Hospital Research Institute, Ottawa, Ontario, Canada. 2 Department of Cellular and Molecular Medicine, Faculty of Medicine, University of Ottawa, Ottawa, Ontario, Canada.

Hong Ming, 1 Regenerative Medicine Program, Ottawa Hospital Research Institute, Ottawa, Ontario, Canada. 2 Department of Cellular and Molecular Medicine, Faculty of Medicine, University of Ottawa, Ottawa, Ontario, Canada.

Yves De Repentigny, 1 Regenerative Medicine Program, Ottawa Hospital Research Institute, Ottawa, Ontario, Canada. 2 Department of Cellular and Molecular Medicine, Faculty of Medicine, University of Ottawa, Ottawa, Ontario, Canada.

Rashmy Kothary, 1 Regenerative Medicine Program, Ottawa Hospital Research Institute, Ottawa, Ontario, Canada. 2 Department of Cellular and Molecular Medicine, Faculty of Medicine, University of Ottawa, Ottawa, Ontario, Canada.

Luis Serrano, 3 Centre for Genomic Regulation (CRG), The Barcelona Institute for Science and Technology, Barcelona, Spain 4 Institució Catalana de Recerca i Estudis Avançats (ICREA), Barcelona, Spain

Michael Rudnicki,1 Regenerative Medicine Program, Ottawa Hospital Research Institute, Ottawa, Ontario, Canada. 2 Department of Cellular and Molecular Medicine, Faculty of Medicine, University of Ottawa, Ottawa, Ontario, Canada.

**Introduction**: Wnt proteins are a secreted family of hydrophobic glycoproteins that govern essential developmental, growth, and regenerative processes, as well as pathological conditions. Recently, our group discovered that upon a muscle injury there is an upregulation of Wnt7a expression on new regenerating myofibers. Moreover, Wnt7a local‐muscle injection into dystrophic mice restores muscle function. However, Wnt7a is highly hydrophobic impairing its systemic delivery to treat all muscles. Despite their relative insolubility due to the palmitoylation required for specific Frizzled receptor biding, Wnt proteins actively participate in long‐range paracrine signaling between Wnt‐producing cells and distal recipient cells. These observations raise the unanswered controversial question of how long‐range Wnt signals are regulated. Recently, we discovered that Wnt7a is secreted in vitro through small Extracellular Vesicles (EVs). Our goal is to decipher the specific mechanism of Wnt7a‐EVs secretion to manufacture highly efficient Wnt7a‐EVs for systemic Duchene Muscular Dystrophy (DMD) treatment.

**Methods**: Using Tangential Flow Filtration we have standardized a Wnt purification protocol that allows the isolation of Wnt EVs without cross‐contamination with other sources of Wnt secretion. Structure‐function analysis, BioID experiments and in silico modeling interaction experiments were performed to elucidate the specific mechanism that regulates Wnt‐EVs secretion.

**Results**: We discovered that Wnt7a is secreted at high levels on exosomes following muscle injury to stimulate regeneration. Structure‐function analysis identified the signal sequence in Wnt7a, that we termed Extracellular Vesicle Signal Peptide (ESP), which directs EVs secretion, and revealed that palmitoylation is not required. This peptide binds specifically to Coatomer proteins through a positively charged motif to direct trafficking of Wnt to EVs. The positively charged motif and mechanism is conserved among Wnts.

**Summary/Conclusion**: Our studies identify a novel signal peptide that traffics Wnt cargo to EVs surface and elucidate the mechanisms that facilitate long‐range Wnt signaling. Our experiments suggest that systemic delivery of Wnt7a loaded on EVs represents a potential therapy for DMD. Moreover, the use of the ESP to direct the display of cargo proteins on the surface of EVs opens the door for multiple therapeutic applications. We anticipate that our discovery will be a starting point for more sophisticated delivery systems as well as establish the fundamental knowledge for Wnt secretion in pathological contexts.

## CC6 The Road to the Clinic: EV Therapeutics and Drug Delivery

Chair: Andreas Moller, Group Leader, Tumour Microenvironment Laboratory, QIMR Berghofer Medical Research Institute, Associate Professor, University of Queensland and Queensland University of Technology, Australia

Chair: Tang‐Long Shen, National Taiwan University, Taiwan (Republic of China)

### CC6.1 Cardiotropic Mechanisms of EV‐Encapsulated AAVs for Gene Therapy in Heart Failure

Sabrina La Salvia, SL , Icahn School of Medicine at Mount Sinai

Yaxuan Liang, YL, Cardiovascular Research Center Icahn School of Medicine at Mount Sinai

Marta Adamiak, MA, Cardiovascular Research Center Icahn School of Medicine at Mount Sinai

Shweta Lodha, SL, Research Center Icahn School of Medicine at Mount Sinai

Samantha Osinki, SO, Research Center Icahn School of Medicine at Mount Sinai

Mihir Parikh, MP, Cardiovascular Research Center Icahn School of Medicine at Mount Sinai

Kimberly Okoli, KO, Icahn School of Medicine at Mount Sinai

Tzu‐Yi Chen, Icahn School of Medicine at Mount Sinai, Department of Department of Genetics and Genomic Sciences

Nicole C. Dubois, NCD, Cardiovascular Research Center Icahn School of Medicine at Mount Sinai

Navneet Dogra, PhD,Department of Genetics and Genomics Sciences, Icahn School of Medicine at Mount Sinai

Susmita Sahoo, SS, Department of Cardiology, Icahn School of Medicine at Mount Sinai

**Introduction**: Adeno‐associated viruses (AAVs) are one of the most commonly used viral vectors in cardiac gene therapy. However, preexisting neutralizing antibodies (NAb) bind to free to AAVs and impair their clinical effect. Here, we describe extracellular vesicles (EV)‐encapsulated AAVs (evAAVs) as a superior cardiac gene delivery system offering high NAb resistance.

**Methods**: We characterized the ultracentrifuge‐isolated evAAVs using TEM, qPCR, Western blot, DLS, qNano, imaging flow cytometry and ExoView technologies. We determined the gene delivery efficacy both in presence and absence of NAbs using human iPS‐derived cardiomyocytes (CM) and human left‐ventricular CM‐AC16 cell line in vitro, and in mice in vivo, via flow cytometry and IVIS bioluminescence imaging. We determined the therapeutic efficacy of SERCA2a (a gene that regulates cardiomyocyte contraction) delivery using either evAAVs or free AAVs in a mouse model of myocardial infarction, in presence of NAbs. We investigated the mechanisms of evAAV uptake and trafficking both in vitro and in vivo using fluorescence‐labeled evAAVs.

**Results**: evAAVs outperformed free AAV in delivering genes in the presence of NAbs, both in vitro and in vivo. Intramyocardially injected evAAVs carrying SERCA2a significantly improved cardiac function (ejection fraction, %EF, ∼60%) compared to free AAVs (∼40%) and saline control (∼20%), in mice with NAb. evAAVs colocoalized with endosomes (Rab5, 7) and delivered significantly higher amounts of Luciferase to human CMs compared to free AAVs. We postulate that evAAVs deliver higher amounts of viruses to the nucleus via their acidification in endosomal compartments, and Bafilomycin A1, which inhibits the proton pump in endosomes, will inhibit this process. In addition, fluorescence‐tagged evAAVs injected to mouse hearts were internalized into all cell types (CMs and non‐CMs). However, evAAV‐mCherry was primarily expressed in CMs (8.3%), but not in non‐CMs (1.2%) showing evAAV‐mediated gene delivery to the heart is cardiotropic

**Summary/Conclusion**: Our study demonstrated that evAAV‐mediated gene delivery can circumvent neutralizing antibody (NAb) issue for gene therapy. We show a complex mechanism of evAAV biology, trafficking and cardiotropism. evAAV is a superior gene delivery vector and a new platform to treat heart failure.

### CC6.2 Nasal administration of MSC‐derived small extracellular vesicles reverses cisplatin‐induced cognitive impairments, changes in white matter and neuronal mitochondrial function in mice

Bojana Milutinovic , The University of Texas MD Anderson Cancer Center

Luis Arroyo, The University of Texas MD Anderson Cancer Center

Mayela Mendt, The University of Texas MD Anderson Cancer Center

Faiza Hancock, The University of Texas MD Anderson Cancer Center

Alex Seua, The University of Texas MD Anderson Cancer Center

Shruti Dharmaraj, The University of Texas MD Anderson Cancer Center

Elizabeth Shpall, The University of Texas MD Anderson Cancer Center

Annemieke Kavelaars, The University of Texas MD Anderson Cancer Center

Cobi j. Heijnen, The University of Texas MD Anderson Cancer Center

**Introduction**: Cognitive deficits including impaired attention, memory and executive functioning are common neurotoxic side effects of chemotherapy. Up to 75% of chemotherapy patients experience cognitive decline and in 30% of the patients it persists after treatment. There are no FDA‐approved therapeutic options. We recently showed that neuronal mitochondrial dysfunction is the underlying mechanism of cisplatin‐induced cognitive impairment (CICI) and is accompanied by changes in white matter organization and hippocampal synaptic loss. Based on our successful use of mesenchymal stem cells (MSCs) to reverse CICI, we proposed that hBM MSC‐derived small extracellular vesicles (sEVs) are also capable of restoring cognition.

**Methods**: sEVs were isolated by differential centrifugation of hBM MSC cell culture medium. Animals were injected with cisplatin according to Chiu et al, 2017. sEV were administered nasally 48 and 96 h after the last cisplatin injection. Cognition was tested in the Puzzle box and Novel Object Place Recognition Test (NOPRT). Distribution of sEVs was monitored using fluorescent DiR. Synaptosomal mitochondrial morphology was analyzed by transmission electron microscopy and mitochondrial oxygen consumption using Seahorse technology. Synaptophysin and Black Gold II staining were used to assess synaptic loss and white matter integrity.

**Results**: Nasal sEV administration reversed the deficits in working and spatial memory as well as executive functioning in a dose‐dependent manner. Damage to mitochondrial morphology and the decrease in mitochondrial oxygen consumption was restored as well as the cisplatin‐induced changes in white matter and synaptic integrity. sEV entered the brain within 30 minutes. Clearance began 24 h after treatment but sEV remained in the brain for at least 48 h.

**Summary/Conclusion**: Nasal administration of MSC‐derived sEV is a potentially effective therapeutic approach to reverse the debilitating effects of CICI.

### CC6.3 Exploring the crosstalk between endothelial autophagy and extracellular vesicle biology: potential role in atherosclerosis

Pierre‐Michaël Coly, Université de Paris, INSERM U970, Paris Cardiovascular Research Centre, Paris, France

Shruti Chatterjee, Université de Paris, INSERM U970, Paris Cardiovascular Research Centre, Paris, France

Fariza Mezine, Université de Paris, INSERM U970, Paris Cardiovascular Research Centre, Paris, France

Florent Dingli, Curie Institute, PSL Research University, Laboratoire de Spectrométrie de masse Protéomique, Paris, France

Damarys Loew, Université de Paris, INSERM U970, Paris Cardiovascular Research Centre, Paris, France

Xavier LOYER, INSERM U970‐PARCC

Chantal Boulanger, Inserm U970‐ Paris Cardiovascular Research Center

**Introduction**: Atherosclerotic lesions mainly form in arterial areas exposed to low shear stress (LSS), where endothelial cells express a senescent and inflammatory phenotype. Our team has recently demonstrated that endothelial autophagy is a protective process, stimulated under conditions of high shear stress (HSS) when compared to LSS, and hampers the development of atherosclerotic lesions. Endothelial EVs have been shown to regulate inflammation, senescence and angiogenesis and might therefore play a crucial role in vascular homeostasis and disease. While previous studies have shown links between autophagy and extracellular vesicle formation, the exact role of the autophagic machinery in the release and uptake of EVs remains elusive. Our aim is therefore to decipher the interplay between these processes in endothelial cells exposed to atheroprone or atheroprotective shear stress.

**Methods**: Confluent human umbilical vein endothelial cells (HUVEC) were exposed to either LSS (2 dyn/cm2) or HSS (20 dyn/cm2) for 24 hours in culture medium containing 2% EV‐free FCS. Large (>200 nm) and small EVs were isolated from conditioned medium by sequential centrifugation and size exclusion chromatography. They were characterized by Western blot analysis of EV markers (CD9, CD63 and HSC70), tunable resistive pulse sensing, flow cytometry and proteomics. Uptake experiments were performed using Membright or Vybrant‐DID‐labelled EVs and differences between groups were assessed by flow cytometry and confocal microscopy.

**Results**: Levels of large and small EVs were fifty and five times higher in HSS than in LSS conditions, respectively. In vivo and in vitro uptake experiments revealed greater EV incorporation by cells exposed to LSS conditions compared to HSS. Interestingly, silencing ATG5 in HUVECs increased EV uptake by cells exposed to HSS. Additionally, endothelial LSS‐EVs appeared to have a greater affinity for HUVECs than HSS‐EVs or EVs derived from platelets, red blood cells, granulocytes and peripheral blood mononuclear cells. Proteomic analysis revealed that LSS‐EVs were enriched in adhesion proteins such as PECAM1, MCAM and integrins. We found that neutralizing PECAM1 and MCAM on EVs significantly reduced their uptake by HUVECs. Finally, inhibiting autophagy using pharmacological or genetic approaches elevated levels of adhesion proteins in HSS‐EVs and caused them to be more readily taken up by HUVECs

**Summary/Conclusion**: These findings suggest that endothelial shear stress and autophagy may have an important function during EV biogenesis and uptake. Given the major role of EVs and autophagy in vascular health, deciphering the relation between these processes may yield innovative strategies for the early detection and treatment of endothelial dysfunction.

### CC6.4 High performance of α‐galactosidase A lysosomal enzyme loaded in extracellular vesicles through recombinant protein overexpression

Joaquin Seras‐Franzoso , Drug Delivery & Targeting (DDT), CIBBIM‐Nanomedicine, Vall d'Hebron Institut de Recerca (VHIR)

Zamira V. Díaz‐Riascos, Drug Delivery & Targeting (DDT), Functional Validation & Preclinical Research, CIBBIM‐Nanomedicine, Vall d'Hebron Institut de Recerca (VHIR)/Networking Research Center on Bioengineering, Biomaterials and Nanomedicine (CIBER‐BBN)

José Luis Corchero, Institut de Biotecnologia i de Biomedicina (IBB) and Department of Genetics and Microbiology, Universitat Autònoma de Barcelona (UAB)/Networking Research Center on Bioengineering, Biomaterials and Nanomedicine (CIBER‐BBN)

Patricia González, Drug Delivery & Targeting (DDT), CIBBIM‐Nanomedicine, Vall d'Hebron Institut de Recerca (VHIR)

Natalia García‐Aranda, Drug Delivery & Targeting (DDT), Functional Validation & Preclinical Research, CIBBIM‐Nanomedicine, Vall d'Hebron Institut de Recerca (VHIR)/Networking Research Center on Bioengineering, Biomaterials and Nanomedicine (CIBER‐BBN)

Mònica Mandaña, Drug Delivery & Targeting (DDT), Functional Validation & Preclinical Research, CIBBIM‐Nanomedicine, Vall d'Hebron Institut de Recerca (VHIR)/Networking Research Center on Bioengineering, Biomaterials and Nanomedicine (CIBER‐BBN)

Roger Riera, Nanoscopy for Nanomedicine Group, Institute for Bioengineering of Catalonia (IBEC)

Ana Boullosa, Drug Delivery & Targeting (DDT), Functional Validation & Preclinical Research, CIBBIM‐Nanomedicine, Vall d'Hebron Institut de Recerca (VHIR)/Networking Research Center on Bioengineering, Biomaterials and Nanomedicine (CIBER‐BBN)

Sandra Mancilla, Drug Delivery & Targeting (DDT), Functional Validation & Preclinical Research, CIBBIM‐Nanomedicine, Vall d'Hebron Institut de Recerca (VHIR)/Networking Research Center on Bioengineering, Biomaterials and Nanomedicine (CIBER‐BBN)

Alba Grayston,Neurovascular Research Laboratory, Vall d'Hebron Institut de Recerca (VHIR)

Marc Moltó‐Abad, Drug Delivery & Targeting (DDT), CIBBIM‐Nanomedicine, Vall d'Hebron Institut de Recerca (VHIR)/Division of Rare Diseases, Reference Center for Hereditary Metabolic Disorders (CSUR, XUEC, MetabERN, and CIBER‐ER), Vall d'Hebron University Hospital

Elena Garcia‐FruitósInstitut de Biotecnologia i de Biomedicina (IBB) and Department of Genetics and Microbiology, Universitat Autònoma de Barcelona (UAB)/Networking Research Center on Bioengineering, Biomaterials and Nanomedicine (CIBER‐BBN)

Rosa Mendoza, Institut de Biotecnologia i de Biomedicina (IBB) and Department of Genetics and Microbiology, Universitat Autònoma de Barcelona (UAB)/Networking Research Center on Bioengineering, Biomaterials and Nanomedicine (CIBER‐BBN)

Guillem Pintos‐Morell,Drug Delivery & Targeting (DDT), CIBBIM‐Nanomedicine, Vall d'Hebron Institut de Recerca (VHIR)/Division of Rare Diseases, Reference Center for Hereditary Metabolic Disorders (CSUR, XUEC, MetabERN, and CIBER‐ER), Vall d'Hebron University Hospital

Lorenzo Albertazzi,Nanoscopy for Nanomedicine Group, Institute for Bioengineering of Catalonia (IBEC)

Anna Rosell,Neurovascular Research Laboratory, Vall d'Hebron Institut de Recerca (VHIR)

Josefina Casas,RUBAM, Biological Chemistry, Institute of Advanced Chemistry of Catalonia (IQAC‐CSIC) / Networking Research Center on Hepatic and Digestive Diseases (CIBEREHD)

Antonio Villaverde,Institut de Biotecnologia i de Biomedicina (IBB) and Department of Genetics and Microbiology, Universitat Autònoma de Barcelona (UAB)/Networking Research Center on Bioengineering, Biomaterials and Nanomedicine (CIBER‐BBN)

Simó Schwartz JrDrug Delivery & Targeting (DDT), CIBBIM‐Nanomedicine, Vall d'Hebron Institut de Recerca (VHIR) / Networking Research Center on Bioengineering, Biomaterials and Nanomedicine (CIBER‐BBN)

Ibane Abasolo,Drug Delivery & Targeting (DDT), Functional Validation & Preclinical Research, CIBBIM‐Nanomedicine, Vall d'Hebron Institut de Recerca (VHIR)/Networking Research Center on Bioengineering, Biomaterials and Nanomedicine (CIBER‐BBN)

**Introduction**: Fabry disease (FD), is a lysosomal storage disorder (LSD) caused by the mutation of α‐galactosidase A (GLA) and enzymatic replacement therapy (ERT) is its preferred therapeutic option. However, ERT requires life‐long administration of large amounts of recombinant enzyme and depends highly on mannose‐6‐phospate (M6P) mediated cell internalization. We propose an alternative system to deliver lysosomal enzymes in EVs simplifying the enzyme downstream processing while protecting the enzyme's activity and improving its bioavailability.

**Methods**: EVs loaded with GLA (EV‐GLA) were produced by protein overexpression in CHO cells. EVs were routinely isolated by water exclusion precipitation and further validated by tangential flow filtration and iodixanol gradient ultracentrifugation. EV‐GLA activity in vitro was assessed by specific enzymatic activity (EA) assay, GLA substrate (Gb3) accumulation and MP6 competition assays. FD mice models were used to evaluate EV‐GLA biodistribution and therapeutic potential.

**Results**: EV‐GLA loaded significant amounts of GLA exhibiting 6 times higher EA than free enzyme. In FD in vitro cultures, EV‐GLA restored WT levels of Gb3 more efficiently than GLA. In vivo, DiR labeled EV‐GLA was detected in liver, lungs and kidneys by ex‐vivo imaging but also in difficult‐to‐target organs such as kidneys and brain. Consequently, single intravenous administration of EV‐GLA restored basal Gb3 levels in highly accessible organs like liver or lungs but also reduced Gb3 in kidneys and brain. Cellular uptake revealed that EV internalization was independent on the MP6 route, fact that could partially explain the higher efficacy and bioavailability of EV‐GLA compared to the free enzyme.

**Summary/Conclusion**: Our data picture EVs loaded by recombinant lysosomal enzymes as natural drug delivery systems increasing the stability, bioavailability and efficacy of the cargo

### CC6.5 Pro‐inflammatory cytokines primed mesenchymal stromal cell‐derived exosomes contribute to tissue regeneration in experimental inflammatory bowel disease

Anna Maria Tolomeo , L.i.f.e.L.a.b. Program, Consorzio per la Ricerca Sanitaria (CORIS), Veneto Region, Padua, Italy

Ignazio Castagliuolo, Department of Molecular Medicine, University of Padova, Padua, Italy

Martina Piccoli, Laboratory of Tissue Engineering, Fondazione Istituto di Ricerca Pediatrica Città della Speranza, Padua, Italy

Michele Grassi, Department of Women's and Children's Health, University of Padova, Padua, Italy

Fabio Magarotto, Department of Women's and Children's Health, University of Padova, Padua, Italy

Giada De Lazzari, Department of Women's and Children's Health, University of Padova, Padua, Italy

Ricardo Malvicini, Instituto de Medicina Traslacional, Trasplante y Bioingenieria (IMeTTyB‐CONICET)

Federico Caicci, Department of Biology,University of Padua, Italy

Chiara Franzin, Fondazione Istituto di Ricerca Pediatrica Città della Speranza, Padua, Italy;

Melania Scarpa, Department of Women's and Children's Health, University of Padova, Padua, Italy

Antonella Viola, Fondazione Istituto di Ricerca Pediatrica Città della Speranza, Padua, Italy;

Andrea PorzionatoDepartment of Neurosciences, University of Padova, Padua, Italy

Michela Pozzobon, Department of Women's and Children's Health, University of Padova, Padua, Italy

Maurizio Muraca, Department of Women's and Children's Health, University of Padova, Padua, Italy

**Introduction**: Several reports have described a beneficial effect of Mesenchymal Stromal Cells (MSCs) and of their secreted extracellular vesicles (EVs) in mice with experimental colitis. However, the effects of the two treatments have not been thoroughly compared in this model.

**Methods**: Here, we compared the effects of MSCs and of MSC‐EV administration in mice with colitis induced by dextran sulfate sodium (DSS) treatment. Since cytokine conditioning was reported to enhance the immunomodulatory activity of MSCs, the cells were kept either under standard culture conditions (naïve, nMSCs) or primed with pro‐inflammatory cytokines (IL1b, IL6 and TNFa; induced, iMSCs). Colitis was induced in C57BL/6N mice by 3% dextran sulfate sodium (DSS) in drinking water for 6 days followed by 3 days on plain water. Healthy controls received plain water only. Mice with colitis received an intraperitoneal injection (IP) of MSCs (4.00E+6 nMSCs, 4.00E+6 iMSCs) on days 4 and 8 or of EVs (1.00E+9 nMSC‐EVs and 1.00E+9 iMSC‐EVs) on days 4, 6 and 8. Control mice received PBS only. Animals were sacrificed on day 10.

**Results**: In our experimental conditions, nMSCs and iMSCs administration resulted in both clinical and histological worsening and was associate with pro‐inflammatory polarization of intestinal macrophages. However, mice treated with iEVs showed clinico‐pathological improvement, decreased intestinal fibrosis and angiogenesis and a striking increase in intestinal expression of Mucin5ac, suggesting improved epithelial function. Moreover, treatment with iEVs resulted in the polarization of intestinal macrophages towards and anti‐inflammatory phenotype and in an increased Treg/Teff ratio at the level of the intestinal lymph node.

**Summary/Conclusion**: Collectively, these data support the concept that MSCs can behave either as anti‐ or as pro‐inflammatory agents depending on the host environment. In contrast, MSC‐EVs showed a beneficial effect, suggesting a more predictable behavior. Moreover, we show for the first time that MSC‐EV administration is associated with enhanced epithelial protection mediated by Mucin5ac hypersecretion.

## CC7 EVs in Translational Medicine

Chair: Edit Buzás, Semmelweis University, Department of Genetics, Cell‐ and Immunobiology, Hungary

Chair: Uta Erdbrugger, University of Virginia School of Medicine, United States

### CC7.1 Apoptotic bodies mediate mesenchymal stem cell‐based therapy for type 2 diabetes

Chenxi Zheng , Center for Tissue Engineering, The Fourth Military Medical University

Bingdong Sui, Center for Tissue Engineering, The Fourth Military Medical University

Jiachen Hu, Center for Tissue Engineering, The Fourth Military Medical University

Shiyu Liu, Center for Tissue Engineering, The Fourth Military Medical University

Yan Jin, Center for Tissue Engineering, The Fourth Military Medical University

**Introduction**: Mesenchymal stem cells (MSCs) have been identified as a promising cell source for translational application in multiple diseases, including type 2 diabetes (T2D). Specifically, MSCs undergo apoptosis and release apoptotic bodies (ABs) during therapeutic application. Nevertheless, the feature, fate and function of MSC‐derived ABs remain largely unknown.

**Methods**: We established high‐fat diet (HFD)‐induced and genetic Db/db T2D mouse models. We explored the therapeutic efficacy of MSCs with apoptosis inhibition. Then, we isolated MSC‐derived ABs and identified their features. The therapeutic potency of ABs was evaluated in two T2D models. Next, the in vivo fate of ABs and the effects of ABs on T2D liver macrophages were analyzed. Finally, the molecular mechanisms underlying the uptake of ABs were evaluated.

**Results**: We firstly found that inhibition of apoptosis impaired the therapeutic efficacy of MSCs in T2D. Next, we found that infusion of isolated ABs was able to alleviate both diet‐induced and genetic T2D models. We further revealed that ABs were efferocytosed by liver macrophages and functionally modulated them, leading to inhibition of macrophage infiltration and transformation of macrophages towards anti‐inflammation phenotype. At the molecular level, we showed that calreticulin was exposed on the surface of ABs which acted as a critical “eat me” signal mediating the functional efferocytosis and therapeutic potency of ABs.

**Summary/Conclusion**: This study revealed the therapeutic mechanism that MSCs undergo apoptosis and release ABs to treat T2D, clarified ABs’ in vivo fate of targeting macrophages and the underlying molecular mechanism, and verified the modulatory effects and therapeutic potential of ABs, thus promoting the establishment of novel T2D treatment strategies.

### CC7.2 Unraveling EV‐mediated cardioprotection: EV‐dependent and ‐independent mechanisms?

Marieke T. T. Roefs, Department of Experimental Cardiology, University Medical Center Utrecht, Utrecht University, The Netherlands

Pieter Vader, CDL Research, University Medical Center Utrecht, The Netherlands

Joost P.G. Sluijter, J.P.G., Department of Experimental Cardiology, University Medical Center Utrecht, Utrecht University, The Netherlands

Joost P.G. Sluijter, J.P.G., Department of Experimental Cardiology, University Medical Center Utrecht, Utrecht University, The Netherlands

**Introduction**: Cardiac progenitor cell (CPC)‐derived extracellular vesicles (EVs) have been shown to protect the myocardium against ischemia/reperfusion injury. However, the underlying mechanisms for CPC‐EV‐mediated cardioprotection remain elusive. By exploring protein‐mediated effects of CPC‐EVs, we discovered that crude EV preparations activate recipient endothelial cells through EV‐dependent and "independent pathways.

**Methods**: CPCs were stimulated with calcium ionophore (ca ion‐EVs), previously shown to influence EV release, or vehicle (control‐EVs) for 24 hours and crude EVs were isolated using size exclusion chromatography (SEC). EV concentration and size was assessed using NTA and proteomic composition was profiled using mass spectrometry. Following SEC, Optiprep gradient ultracentrifugation was used to separate EVs from free proteins. EV‐ and protein fractions were functionally characterized based on endothelial cell activation assays.

**Results**: Endothelial cells displayed enhanced phosphorylation of ERK1/2 and AKT and increased wound closure after stimulation with control‐EVs, but not with ca ion‐EVs. Proteomic analysis identified multiple proteins uniquely expressed or enriched in control‐EVs compared with ca ion‐EVs. Surprisingly, when investigating the contribution of individual candidate proteins, the extent of endothelial cell activation was found to be influenced by the purity of the EV preparations. EVs isolated using Optiprep gradients lost part of their ability to activate endothelial cells compared to crude EV preparations. In addition, several candidate proteins were found to be present in the free protein fraction instead of the EV fraction. This hints towards a co‐stimulatory role of co‐isolated proteins in recipient cell activation.

**Summary/Conclusion**: A specific set of EV proteins is identified that may be functionally responsible for the activation of endothelial cells upon exposure to CPC‐EVs. It is important to identify if these proteins are EV‐associated or represent co‐isolated factors that contribute to endothelial cell activation. This may lead to a better mechanistic understanding of CPC‐EV‐mediated cell activation and translation of EV‐mediated therapeutics.

### CC7.3 Administration of Extracellular Vesicles Derived from Amniotic Fluid Stem Cells Rescues Autophagy in Underdeveloped Fetal Lungs

Kasra Khalaj, PhD, MSc, The Hospital for Sick Children

Lina Antounians, MSc, The Hospital for Sick Children

Rebeca Figueira, PhD, MSc, The Hospital for Sick Children

Martin Post, PhD, The Hospital for Sick Children

Augusto Zani, MD, PhD, FACS, FAAP, The Hospital for Sick Children

**Introduction**: We previously showed that administration of extracellular vesicles derived from amniotic fluid stem cells (AFSC‐EVs) promote lung growth and maturation in fetuses with pulmonary hypoplasia (PH) via a miRNA‐mediated mechanism. It has been recently reported that autophagy is crucial for normal lung development. Herein, we aimed to assess whether PH lungs have abnormal autophagy and if AFSC‐EV treatment can restore normal levels of autophagy.

**Methods**: EVs were separated from rat AFSC conditioned medium by ultracentrifugation (100,000g/14hrs), and characterized for size (nanoparticle tracking analysis), morphology (transmission electron microscopy), and Western blot (WB; EV‐specific markers: CD63, CD81, TSG101). Following our established PH model in fetal rats (nitrofen administration to rat dams at E9.5), lungs were harvested at E14.5 and E17.5 and explants were established. PH explants were treated with AFSC‐EVs (10%v/v AFSC‐EVs; NA) or medium alone (N) for 72h. Fetal lungs from untreated dams served as control. Gene and protein levels of autophagy markers (activator: ATG5,BECN1; repressor: SQSTM1) were analyzed via RT‐qPCR and WB. Purified EV‐RNA was sequenced using NEB‐Next. We compared N with NA treated samples for miRNAs associated with autophagy. Statistics: One‐way (qPCR and WB) and Two‐way (RNA‐seq) ANOVAs.

**Results**: PH explants had lower Atg5 and Becn1 and higher levels of impaired autophagy marker, SQSTM1 at both time points. AFSC‐EV treatment to hypoplastic lungs restored expression levels of all markers back to normal control levels. RNA‐sequencing identified 3 miRNAs (miRs‐17,‐20a,‐93) known to be associated with autophagy (in relation to Sqstm1) and were enriched in the AFSC‐EV‐treated group.

**Summary/Conclusion**: This is the first study demonstrating impaired autophagy in PH, which can be rescued by the administration of AFSC‐EVs. miRNA cargo contained in AFSC‐EVs targets autophagy mechanism in developing lungs and represents a promising approach to treat PH.

### CC7.4 Angio‐modulatory role of amniotic fluid derived extracellular vesicles in preeclampsia

Natalia Gebara , University of Turin

Renata Skovronova, University of Turin

Julia Scheel, Department of systems biology and bioinformatics

Luca Marozio, Department of Surgical Sciences,Obstetrics and Gynecology

Benedetta Bussolati, University of Turin, Department of Molecular Biotechnology and Health Sciences, Italy

**Introduction**: Preeclampsia is a hypertensive pregnancy disorder that affects up to 8% of pregnancies worldwide. Many metabolically active cell types of the placenta or fetus secrete within the amniotic fluid bio‐factors, such as VEGF, endoglin, PGF‐1 as well as extracellular vesicles (EVs). In pathological conditions, such as hypoxia, these factors may affect the physiology of pregnancy and cause multiple pathologies such as preeclampsia. This study aims to fully characterize, test, and compare the bio‐function of amniotic fluid‐derived EVs from normal and preeclamptic pregnancies.

**Methods**: The amniotic fluid was obtained from gestationally matched normal and preeclamptic pregnancies, during cesarean sections. Samples were immediately processed for EV isolation. The physical parameters were tested by Nanosight technology. EVs were further characterized by a super‐resolution microscopy. The identification of exosomal markers was tested by Macsplex exosome kit and NanoView. Angiogenic effects of EVs were tested using tube formation assay. A comparative bioinformatic analysis of 754 miRNAs, between, normal and pre‐eclamptic patients, was performed.

**Results**: We set up a method for EV isolation from the amniotic fluid using differential centrifugation and filtration steps. EVs from 22 normal pregnancies and 7 preeclamptic samples were analyzed. The EV size and concentration were 222.8 +/‐ 6nm and 2.8 × 1010 /ml. In a comparison of 171.5+/‐7nm and 2.7 × 10 9 /ml for 7 preeclamptic pregnancies samples. For miRNA and NanoView experiments, we further purified the EV samples using size exclusion columns. Size and tetraspanin expression were confirmed by dSTORM analysis using the superresolution NanoImager microscope. NanoView and Mascplex analysis showed differences between the angiogenic marker (CD105 (endoglin)), progenitor markers (CD24 and SSEA‐4), and the MHC‐1 (HLA‐ABC). Tube formation assay showed pre‐eclampsia derived EVs to significantly downregulate formation of tubules in comparison with controls and normal pregnancy derived‐EVs. Several miRNAs were detected to be differentially expressed in EVs derived from preeclamptic pregnancies

**Summary/Conclusion**: Our results show that pre‐eclamptic EVs differ in their content and angiogenic ability, as well as in their size in comparison with normal pregnancy amniotic fluid‐derived EVs. Our data support the importance of EV's role as cell‐cell communicators and their significant function in the development of pre‐eclampsia.

### CC7.5 Pharmacokinetics and biodistribution of EV administered intravenously versus intranasally in mice and macaque models

Tom Driedonks, PhD , Johns Hopkins Medical School / Utrecht University

Bess Carlson, Dept. Molecular and Comparative Pathobiology, Johns Hopkins School of Medicine, Baltimore, MD

Suzanne Queen, Dept. Molecular and Comparative Pathobiology, Johns Hopkins School of Medicine, Baltimore, MD

Olesia Gololobova, Johns Hopkins University School of Medicine

Zheng Han, Russell H. Morgan Department of Radiology, Johns Hopkins University School of Medicine, Baltimore, Maryland, USA

Guanshu Liu, Russell H. Morgan Department of Radiology, Johns Hopkins University School of Medicine, Baltimore, Maryland, USA

Lyle Nyberg, Dept. Molecular and Comparative Pathobiology, Johns Hopkins School of Medicine, Baltimore, MD

Gabriela Lima, Dept. Molecular and Comparative Pathobiology, Johns Hopkins School of Medicine, Baltimore, MD

Kayla Schonvisky, Dept. Molecular and Comparative Pathobiology, Johns Hopkins School of Medicine, Baltimore, MD

Natalie Castell,Dept. Molecular and Comparative Pathobiology, Johns Hopkins School of Medicine, Baltimore, MD

Barbara Smith, Dept. Cell Biology, Johns Hopkins School of Medicine, Baltimore, MD

Charles Lai, PhD, Institute of Atomic and Molecular Sciences, Academia Sinica, Taipei, Taiwan

Jessica Izzi, Dept. Molecular and Comparative Pathobiology, Johns Hopkins School of Medicine, Baltimore, MD

Eric Hutchinson,Dept. Molecular and Comparative Pathobiology, Johns Hopkins School of Medicine, Baltimore, MD

Kelly Pate,Dept. Molecular and Comparative Pathobiology, Johns Hopkins School of Medicine, Baltimore, MD

Kenneth W. Witwer,Johns Hopkins University School of Medicine

**Introduction**: Extracellular vesicles (EVs) have potential to deliver therapeutic cargo to tissues including brain. It was previously shown in mice that intravenously administered EVs have a half‐life of minutes and are taken up mostly by the liver and spleen. Alternative administration routes may result in different distributions. Although mice are a valuable preclinical model, physiologic differences between mice and humans may limit the translational value of findings. Here, we compared the blood and cerebrospinal fluid (CSF) pharmacokinetics of EVs administered intravenously (i.v.), intranasally (i.n.), and intrathecally (ith.) to macaques. We also compared organ biodistribution of i.v. and i.n. EVs in mice and macaques.

**Methods**: EVs containing a palmitoylated GFP‐Nanoluciferase (palmBRET) dual reporter were produced in Expi293F cells, concentrated by TFF, labelled with MemGlow (700 nm) lipid dye, and purified by SEC. EVs were characterized by Western blot, NTA, electron microscopy, SP‐IRIS, and imaging flow cytometry. 3E10^10 " 7E10^10 EVs were administered i.v., i.n., or ith. into macaques, and plasma and CSF were sampled at regular intervals for 24h. 6E10^9 EVs were administered i.v. or i.n. into mice, and organs were harvested after 60 min. Presence of the EV reporter in tissue lysates and biofluids was measured by NanoGlo assay.

**Results**: PalmBRET bioluminescence was present in EV‐containing SEC fractions, colocalized with CD9, CD63 and CD81 by SP‐IRIS, and was protease resistant in the absence of detergent. PalmBRET EVs were detectable over a 10^5‐fold dilution series in macaque plasma. The route of administration affected EV levels in biofluids. After ith. administration, EVs were cleared rapidly (t1/2 = 2 min) from CSF. Intravenously injected EV were rapidly cleared from plasma (t1/2 = 2 min). At the administered dose, i.n. EVs were not detected in plasma. EVs administered i.n. and i.v. accumulated in CSF between 1h and 24h. In mice, the administration route affected EV biodistribution, with i.v. EVs accumulating predominantly in the liver, spleen, and kidney, and i.n. EVs accumulating in lung and brain.

**Summary/Conclusion**: Highly sensitive reporters such as palmBRET enable pharmacokinetic studies of EVs in large animal models. Our data reveal that EV administration route affects the uptake and biodistribution of EVs, which may inform future therapeutic applications of EVs in humans. Studies of higher EV doses and distribution in macaques are ongoing.

## CC8 EVs and Viruses: Partnerships in the Pandemic

Chair: Lynn Pulliam, University of California, San Francisco, United States

Chair: Metka Lenassi, Institute of Biochemistry and Molecular Genetics, Faculty of Medicine, University of Ljubljana, Slovenia, EU

### CC8.1 ACE2‐containing extracellular vesicles and exomeres bind the SARS‐CoV‐2 spike protein

Qin Zhang , Vanderbilt University Medical Center

Dennis Jeppesen, Vanderbilt University Medical Center

James Higginbotham, Vanderbilt University Medical Center

Jeffrey Franklin, Vanderbilt University Medical Center

James Crowe, Vanderbilt University Medical Center

Robert Coffey, Vanderbilt University Medical Center

**Introduction**: Small extracellular vesicles (sEVs) and exomeres (a recently discovered nanoparticle) are increasingly implicated in both physiological and pathophysiological conditions. The COVID‐19 pandemic is due to SARS‐CoV‐2 that via its spike (S) protein binds the host cell receptor angiotensin‐converting enzyme 2 (ACE2) to enter cells. Host serine protease TMPRSS2 primes the spike (S) protein of SARS‐CoV‐2 for cellular entry. Recently, human recombinant (r) soluble ACE2 has been shown to attenuate SARS‐CoV‐2 infection in vitro and in patients, presumably acting as a viral decoy. TMPRSS2 and the metalloprotease TACE cleave and release the ectodomain of ACE2, raising the possibility that extracellular ACE2 also may act as a viral decoy.

**Methods**: sEV pellets (sEV‐Ps) and exomeres were isolated by differential high‐speed ultracentrifugation from the conditioned medium of human colorectal cancer cell lines (DiFi, DKO‐1, LIM1215, Caco‐2, LS174T). The binding of ACE2 in sEVs and exomeres to the receptor binding domain (RBD) of the S1 subunit of S protein was examined by flow cytometry and co‐immunoprecipitation. α2,6‐sialylated proteins were pelleted with the SNA lectin. For cytokine treatment, CRC or Calu‐3 lung cancer cells were treated with 200 U/ml or 1000 U/ml of rhuman IFN‐γ and/or with 50 ng/ml of rhuman TNF‐α.

**Results**: Full‐length ACE2 is released in sEVs from CRC cells and two ectodomain fragments of ACE2 are enriched in exomeres. TMPRSS2 and TACE are expressed in CRC cells and secreted in sEVs. ACE2 in cells, sEVs and exomeres is α2,6‐sialylated by ST6Gal‐1. ACE2‐containing sEVs and exomeres bind the RBD of the SARS‐CoV‐2 S protein S1 subunit. Intriguingly, IFN‐γ induces inflammation and reduces ACE2 expression in these CRC cells and in Calu‐3 cells.

**Summary/Conclusion**: CRC cell‐derived sEVs contain full‐length ACE2 and TMPRSS2 that primes the S protein of SARS‐CoV‐2 for entry. Exomeres contain ectodomain‐shed ACE2. ACE2 in sEVs and exomeres undergoes 2,6 sialylation by ST6Gal‐1. Studies are underway to determine 1) the relative efficiency of ACE2‐containing sEVs and exomeres to bind S protein compared to recombinant human ACE2 and 2) if inflammatory modulation of cellular ACE2 expression results in altered secretion of ACE2 in sEVs and exomeres. Our findings support the hypothesis that ACE2‐containing sEVs and exomeres may act as viral decoys for SARS‐CoV‐2 and have potential important implications for the treatment COVID‐19.

### CC8.2 Extracellular Vesicle Capture by AnTibody of CHoice and Enzymatic Release (EV‐CATCHER): A customizable purification assay designed for small‐RNA biomarker identification and functional evaluation of extracellular vesicles

Megan I. Mitchell , Center for Discovery and Innovation, Hackensack Meridian Health

Iddo Ben‐Dov, Hadassah‐Hebrew University Medical Center

Kenny Ye, Albert Einstein College of Medicine

Christina Liu, Center for Discovery and Innovation, Hackensack Meridian Health

Kar Chow, Hackensack University Medical Center

Yael Kramer, Hackensack University Medical Center

Anju Gangadharan, Hackensack University Medical Center

Steven Park, Center for Discovery and Innovation, Hackensack Meridian Health

Sean Fitzgerald, Center for Discovery and Innovation, Hackensack Meridian Health

Andrew Ramnauth,Weill Cornell Medicine

David Perlin, Center for Discovery and Innovation, Hackensack Meridian Health

Michele Donato,Hackensack University Medical Center

Emily Bhoy, Center for Discovery and Innovation, Hackensack Meridian Health

Ehsan Manouchehri Doulabi,Uppsala University

Masood Kamali‐Moghaddam,University of Uppsala

Olivier Loudig,Center for Discovery and Innovation, Hackensack Meridian Health

**Introduction**: Circulating nucleic acids in extracellular vesicles (EVs) provide a stable source of disease biomarkers. However, the selective isolation of disease‐associated EVs from whole biofluid is critical. Many available purification assays rely on the physical properties of extracellular vesicles, rather than inherent cellular characteristics. We established a highly selective purification assay, termed EV Capture by AnTibody of CHoice and Enzymatic Release: EV‐CATCHER, designed for high‐throughput analysis of low‐abundance miRNAs and the evaluation of EV functional characteristics.

**Methods**: Evaluation of EV‐CATCHER sensitivity by specific capture and small‐RNA sequencing of mouse EVs spiked into human plasma. Western blotting, nanoparticle tracking, and TEM were used to evaluate EV purification between EV‐CATCHER and commercial assays. miRNA sequencing was performed on EV‐CATCHER purified EVs from sera of consented, hospitalized patients with either mild or severe Covid‐19 disease. Finally, In vitro functional evaluation of Covid‐19 convalescent EVs isolated from sera using EV‐CATCHER was performed.

**Results**: Our miRNA data demonstrates the purity and sensitivity of EV‐CATCHER in isolating mouse EVs spiked in human plasma, using a mouse‐specific CD63 antibody. Small‐RNA sequencing of EVs isolated from sera of mildly and severely ill Covid‐19 patients identified hsa‐miR‐146a and hsa‐miR‐126‐3p to be significantly downregulated with severity, two miRNAs associated with inflammation and endothelial cell repair inhibition, not significantly detectable in whole sera. Finally, using EV‐CATCHER we identified neutralizing properties against SARS‐CoV‐2 for EVs isolated from high anti‐spike IgG convalescent sera, demonstrating that our assay allows for release of intact and functional EVs.

**Summary/Conclusion**: EV‐CATCHER represents a versatile molecular assay for the highly specific purification of EVs from all biofluids, with unique properties to identify circulating biomarkers and functionality associated with disease.

### CC8.3 The progression of a Covid‐19 pneumonia to Sars‐CoV‐2 acute respiratory distress syndrome (ARDS) is regulated by serum cell‐free miRNAs in precipitated extracellular vesicles

Agnes S. Meidert , University Hospital, Ludwig‐Maximilians‐University Munich

Stefanie Hermann, Technical University of Munich

Florian Brandes, Ludwig‐Maximilians‐University Munich

Benedikt Kirchner, MSc, Technical University of Munich

Dominik Buschmann, Technical University of Munich

Anja Lindemann, University Hospital, Ludwig‐Maximilians‐Universität München

Marlene Reithmair, University Hospital, Ludwig‐Maximilians‐Universität München

Gustav Schelling, University Hospital, Ludwig‐Maximilians‐Universität München

Michael W. Pfaffl, PhD, Chair of Animal Physiology & Immunology

**Introduction**: Extracellular vesicles (EVs) and their biologically active molecules regulate the intercellular communication during inflammatory lung response. EVs and enveloped viruses share several molecular and structural characteristics, and some viruses can hijack EV biogenesis to promote their dissemination. We assume that serum circulating EVs released during Sars‐CoV‐2 infection support inflammatory processes and might be associated with the progression of Covid‐19 pneumonia (CoP) to severe Covid‐19 associated pulmonary failure (ARDS).

**Methods**: We studied 20 symptomatic patients with confirmed CoP (age = 63.5±14.8 years, CURB‐65 score = 1.2±1.2), 20 mechanically ventilated patients with Covid‐19 ARDS (age = 62.5±10.9, paO2/FiO2 ratio = 139.9±57.9) and 20 healthy controls (age = 40±12.9). EVs were purified from serum by precipitation, total RNA was isolated and small‐RNA was profiled by NGS. Differential gene expression (DGE) was performed, and differentially regulated miRNAs were analyzed by Ingenuity Pathway Analysis (IPA) to characterize signaling pathways in CoP and the progression to ARDS with healthy individuals serving as controls.

**Results**: DGE revealed 42 differentially expressed miRNAs in CoP compared to the healthy state, and 19 regulated miRNAs in patients with CoP in comparison to ARDS. IPA revealed differentially regulated signaling networks between CoP and healthy individuals with miR‐542‐3p targeting TNF, and miR‐3168 or miR‐338‐5p targeting IL‐6. miR‐197‐3p and miR‐338‐5p also targeted OR52N2, an olfactory receptor in the nose that triggers the perception of smell. The olfactory pathway is currently regarded as a major route for neuroinvasiveness of the Sars‐CoV‐2 virus. Comparing CoP to ARDS resulted in a fully activated network of cytokines and transcription factors controlled by downregulated miR‐4433b‐5p, miR‐4433b‐3p and miR‐3168.

**Summary/Conclusion**: Our data indicate a role for cell‐free miRNAs as mediators of pathophysiologic changes in CoP and the associated progression to ARDS. EVs could also serve as carrier vehicles for future RNA based vaccines against SARS‐CoV‐2.

### CC8.4 Single‐particle detection and analysis of SARS‐CoV‐2 protein‐carrying extracellular vesicles

Linglei Jiang, Johns Hopkins University

Tom Driedonks, PhD, Johns Hopkins Medical School / Utrecht University

Wouter Jong, Abera Bioscience AB, Stockholm, Sweden

Zhaohao Liao, Johns Hopkins University

Olesia Gololobova, Johns Hopkins University School of Medicine

Fengying Li, Johns Hopkins University

Joen Luirink, Vrije Universiteit Amsterdam

Kenneth W. Witwer, Johns Hopkins University School of Medicine

**Introduction**: Severe acute respiratory syndrome coronavirus 2 (SARS‐CoV‐2) is the causative agent of COVID‐19, with a death toll reaching two million in just over a year after the first reported case. Despite rapid development and deployment of vaccines, continuing spread, vaccine availability, unknown longevity of immune responses, and viral mutations suggest that SARS‐CoV‐2 may be a problem for years to come. Here, we used several single‐particle analyses to determine if viral proteins could be detected on the surface of extracellular vesicles as virus mimetics.

**Methods**: The receptor binding domain (RBD) of SARS‐CoV‐2 Spike protein was expressed in mammalian cell culture and purified. Extracellular vesicles were collected from an engineered bacterial strain. RBD was then conjugated to the EV surface using affinity tag technology. EVs were then characterized in bulk by Western blot, zeta potential measurement, and particle tracking. Single‐particle analyses were done with immunogold EM and single particle interferometric reflectance imaging sensing (SP‐IRIS).

**Results**: Highly efficient conjugation of Spike RBD to bacterial EVs was observed by densitometry and confirmed by immunogold labelling. The diameter range of conjugated particles was from about 30 nm to 200 nm. Particles were successfully detected with custom SP‐IRIS capture chips printed with affinity reagents to Spike epitopes and bacterial LPS. Furthermore, SP‐IRIS in fluorescence mode confirmed co‐localization of Spike and bacterial LPS.

**Summary/Conclusion**: Our results show proof of principle that SARS‐CoV‐2 protein produced in mammalian cell culture can be conjugated to bacterial EVs, and also that viral protein‐containing particles can be detected and characterized at the single‐particle level by technologies including SP‐IRIS. These findings may have implications for diagnosis and monitoring of infection as well as mobilization of highly tractable bacterial systems for future vaccines.

### CC8.5 Deciphering the biogenesis mechanisms of JC polyomavirus associated extracellular vesicles

Jenna Morris‐Love, Brown University

Bethany O'Hara, MS, Brown University

Gretchen V. Gee, PhD, University of Massachusetts Medical School, MassBiologics

Aisling S. Dugan, PhD, Assumption University

Brandon Armstead, MS, Brown University

Ryan O'Rourke, Brown University

Benedetta Assetta, PhD, Brown University

Sheila A. Haley, PhD, Brown University

Walter J. Atwood, PhD, Brown University

**Introduction**: JC polyomavirus (JCPyV) is a small non‐enveloped virus that causes the neurodegenerative disease Progressive Multifocal Leukoencephalopathy (PML). JCPyV establishes productive infections using either receptor‐dependent or receptor‐independent mechanisms. In the receptor‐dependent mechanism naked JCPyV requires attachment to a sialic acid containing receptor followed by interaction with a member of the 5‐hydroxytryptamine type 2 serotonin receptor family that triggers clathrin‐mediated endocytosis. In the receptor‐independent mechanism JCPyV is associated with extracellular vesicles that are taken up by target cells via clathrin‐mediated endocytosis or macropinocytosis regardless of the presence of either attachment or entry receptors. The biogenesis of extracellular vesicles involves several non‐mutually exclusive pathways with a myriad of proteins. Here, we begin to characterize the general pathways and individual proteins crucial to the production of JCPyV associated extracellular vesicles (EVs).

**Methods**: Using chemical and/or genetic depletion techniques we targeted neutral sphingomyelinase 2 (nSMase2), endosomal sorting complexes required for transport (ESCRT) proteins, common EV associated tetraspanins, and factors involved in secretory autophagy.

**Results**: We found the use of drug treatments against nSMase2 decreased the spread of purified JCPyV over time. Genetic depletion of nSMase2 caused an increased in EVs produced per cell yet decreased the quantity of protected viral genomes associated with EVs and reduced infectious EV production. Knockdown of seven different ESCRT‐related proteins“HRS, ALIX, TSG101, VPS25, VPS20, CHMP4a, and VPS4a”did not significantly affect JCPyV‐EV infectivity or production, whereas knockdown of the tetraspanins CD9 and CD81 or the secretory autophagy related proteins Rab8a, Rab27a, and GRASP65 all significantly reduced the spread of purified JCPyV and decreased production of infectious EVs.

**Summary/Conclusion**: These findings point to a general role for exosomes and secretory autophagy in the release of JCPyV associated EVs with a specific role for nSMase2, CD9, CD81, Rab8a, Rab27a, and GRASP65 proteins.

## On Demand Oral Sessions (OD)

## OD01 EV Lipids in Pathogenesis

Chair: Juan Manuel Falcón‐Pérez, CIC bioGUNE, Spain

Chair: Soazig Le Lay, Institut du Thorax INSERM 1087, France

### OD01.01 The specific enrichment in lipids, proteins and miRNAs of exosomes released from skeletal muscle explants is altered in obese mice and modulated lipid storage in adipocytes

AUDREY JALABERT, INRAe

Laura Reininger, CeeD

EMMANUELLE BERGER, INRAe

YOHAN COUTE, Univ. Grenoble Alpes, CEA, INSERM, IRIG, BGE, Grenoble, France

EMMANUELLE MEUGNIER, INRAe

Alexis FORTERRE, DIATECH, UMR 7294, Centre européen d’étude du Diabète

ELIZABETH ERRAZURIZ‐CERDA, UNIVERSITY OF LYON

ALAIN GELOEN, CNRS

Karim Bouzakri, CEED

JENNIFER RIEUSSET, INSERM

SOPHIE ROME, INRAE

**Introduction**: We have determined for the first time, the specific enrichment in lipids, proteins and miRNAs of exosome‐like vesicles (EXO) released from skeletal muscle (SkM) explants from mice. Then we have determined the impact of obesity‐induced insulin resistance on SkM‐EXO composition and biological functions

**Methods**: Ob/ob and C57BL/6 WT mice were fed for 12 weeks (standard chow diet). At sacrifice quadricep (Quad) and gastrocnemus (Gast) were excised and cut into small pieces to remove all contaminant tissues and incubated for 24h in serum‐free DMEM. EXO were extracted by differential centrifugations/filtrations/ultracentrifugations. EXO were labeled with anti‐CD81and visualized by TEM. TSG101 and ALIX were detected by WB. EXO proteins were quantified by using a mass spectrometry‐based quantitative proteomic approach. Individual phospholipid classes and and cholesterol were quantified from Gast or from EXO, released from the same Gast muscle to calculate specific lipid enrichment. MiRNAs were quantified by TaqMan(R) Low Density Arrays and compared with their expressions in Quad explants.

**Results**: ob/ob‐Quad released significantly less EXO than WT‐Quad, in agreement with the decrease in RAB35 and with the increase in cholesterol concentration vs WT‐Quad. WT‐EXO contained 798 proteins involved in glucose metabolism, signalling pathways and inflammation. They were located in intracellular organelles (also mitochondria), but not in plasma membrane validating the endosomal origin of EXO. 49 proteins were differentially abundant between WT‐EXO and ob/ob‐EXO, involved in lipid oxidation and with catalytic activities. Compared with Gast, Gast‐EXO accumulated sphingomyelin and cholesterol. The levels of specific subspecies of ceramides, sphingomyelin and phosphatidylcholine were modified in ob/ob‐EXO vs WT‐EXO but these variations did not mirror those between ob/ob‐Gast and WT‐Gast. The WT‐EXO miRNA population was surprising as many miRNAs had a nuclear addressing sequence and computer predictions indicated that their target genes were enriched in genes encoding proteins with nuclear activities. On the 7 miRNAs differentially expressed between WT‐EXO and ob/ob‐EXO, 4 had been found expressed in nucleus of various cell types and 2 had a nuclear motif in their 3’ regions. Ob/ob‐EXO induced lipid storage whereas WT‐EXO prevent lipid accumulation in adipocytes, suggesting for the first time, the existence of a cross‐talk between SkM and adipose tissue in favor of the adipose tissue expansion in obesity.

**Summary/Conclusion**: During obesity, all SkM‐EXO components are altered and can individually participate in the spread of a deleterious endocrine signal from SkM to adipose tissue, by targeting different intracellular organelles in these recipient cells, including the nucleus.

### OD01.02 Characterization of Brain Derived Extracellular Vesicle Lipids in Alzheimer's Disease

Huaqi Su, The University of Melbourne

Yepy Rustam, The University of Melbourne

Colin Masters, The University of Melbourne

Enes Makalic, The University of Melbourne

Catriona McLean, The Florey Institute of Neuroscience and Mental Health

Andrew Hill, La Trobe University

Kevin Barnham, The Florey Institute of Neuroscience and Mental Health

Gavin Reid, The University of Melbourne

Laura Vella, The Florey Institute of Neuroscience and Mental Health

**Introduction**: Lipid dysregulation is associated with Alzheimer's disease (AD) pathogenesis, however the application of lipids as blood‐based biomarkers of AD, has proven difficult. Most biomarker studies have focused on examining the entire lipidome, without focusing on brain‐specific lipids. However, the small extracellular vesicles (EV), can pass the blood‐brain barrier (BBB) and enter the periphery, while carrying a subset of lipids which make them uniquely suited for biomarker exploration.

**Methods**: To determine the potential of EV lipids as biomarkers, using the protocol developed for brain‐derived exosome isolation and characterization (Vella, et al. JEV. 2017) and quantitative mass spectrometry based lipidome analysis (Rustam and Reid. Anal. Chem. 2018), we revealed the lipidomic characterization of post‐mortem frontal cortices and the brain derived EV (BDEV) from AD subjects versus controls.

**Results**: Western blot result showed enrichment of syntenin and TSG101 in the isolated BDEV preparations with little calnexin, indicating minimum cell lysis. Transmission electron microscopy revealed the typical round and cup‐shaped EV morphology with vesicle size being 50–150nm. A total of 692 lipids were identified and quantified. Enrichment of glycerophosphatidylserine (PS) lipids, especially the ether PS lipids, alkyl‐ and alkenyl‐, were observed in BDEV. Remodeling in phosphatidylethanoamine (PE) lipid class and polyunsaturated fatty acyl containing lipids (PUFA‐) was observed in AD BDEV in comparison to control BDEV.

**Summary/Conclusion**: We, for the first time, characterized the lipid composition of EVs in human frontal cortex. BDEVs offered improved detection of dysregulated lipids in AD over global lipid profiling of the brain region. Many of these changes have previously been reported to play key roles in AD pathogenesis, suggesting BDEV provide a readout of lipid dysregulation in AD and highlighting the potential use of these lipids as disease biomarkers in the periphery.

### OD01.03 Glycerophospholipid and sphingolipid composition of equine synovial fluid derived extracellular vesicles obtained pre‐ and post LPS‐induced acute synovitis

Laura Varela , Division of Equine Sciences, Department of Clinical Sciences, Faculty of Veterinary Medicine, Utrecht University, Utrecht, The Netherlands

Chris H.A. van de Lest, Division of Equine Sciences, Department of Clinical Sciences, Faculty of Veterinary Medicine, Utrecht University, Utrecht, The Netherlands

René P.R. van Weeren, Division of Equine Sciences, Department of Clinical Sciences, Faculty of Veterinary Medicine, Utrecht University, Utrecht, The Netherlands

Marca H.M. H.M. Wauben, Department of Biomolecular Health Sciences, Utrecht University, The Netherlands

**Introduction**: Inflammation is the hallmark of many diseases and most joint disorders have inflammation as their common denominator, though causes of inflammation may differ. Extracellular vesicles (EVs) are known to play a role in intercellular communication in both health and disease states. The aim of this study was to investigate the lipidome profile of synovial fluid‐derived EVs in healthy equine joints and joints with lipopolysaccharide (LPS)‐induced synovitis, a model of acute, however transient and fully reversible, synovitis, characterized by an acute inflammation phase 5–8h after injection with the height of the resolution phase at around 24h post‐injection. Focus was on non‐neutral lipids, including the glycerophospholipids and sphingolipids.

**Methods**: Equine synovial fluid (SF) was harvested prior to LPS‐injection at 0 hours, and at 5 hours and 24 hours post‐injection. SF was centrifugated at 3000g and stored at ‐80°C. Thawed cell‐free SF samples were treated with hyaluronidase, DNAse I, and sodium citrate. Extracellular vesicles were isolated by differential ultracentrifugation with final steps at 10,000g and 200,000g, followed by EV purification with sucrose density gradients. From each fraction, lipids were extracted by the Bligh & Dyer method and analyzed using a Fusion orbitrap mass spectrometer. The subsequent data processing, as well as data visualization and statistical analyses, were performed using R packages and/or GraphPad software.

**Results**: We identified more than 250 lipid species within 14 lipid classes. Total lipid levels were significantly higher (p < 0.001) in EVs from the 5h (7‐fold) and 24h (2.6‐fold) collected‐SF when comparing to the 0h samples. Moreover, there was an increase of hexosylceramide (HexCer), phosphatidylserine (PS), phosphatidylcholine (PC), and sphingomyelin (SM) in the composition of EVs from both the peak and the resolutions phases. On the other hand, the amount of phosphatidylethanolamine (PE) relatively decreased in the inflamed samples compared to healthy SF.

**Summary/Conclusion**: Our results show that inflammation has clearly a profound effect on the lipidome composition of SF‐derived EVs.

### OD01.04 Extracellular vesicle ‐mediated crosstalk between adipose tissue and liver in the development of non‐alcoholic fatty liver disease

Johanna Matilainen , University of Eastern Finland, Institute of Biomedicine

Ville Männistö, University of Eastern Finland, Institute of Public Health and Clinical Nutrition; Kuopio University Hospital

Natalia Rosso, Fondazione Italiana Fegato ONLUS, The Italian Liver Foundation

Jussi Pihlajamäki, University of Eastern Finland, Institute of Public Health and Clinical Nutrition

Uma Arasu, University of Eastern Finland, A.I. Virtanen Institute for Molecular Sciences

Ashik Deen, University of Eastern Finland, A.I. Virtanen Institute for Molecular Sciences

Petteri Nieminen, University of Eastern Finland, Institute of Biomedicine

Anne‐Mari Mustonen, University of Eastern Finland, Institute of Biomedicine

Reijo Käkelä, University of Helsinki, Molecular and Integrative Biosciences Research Programme

Kai Härkönen,Finnish Red Cross Blood Service

Kirsi Rilla, University of Eastern Finland, Institute of Biomedicine

**Introduction**: Recent studies have produced evidence that extracellular vesicles (EV) released from adipocytes and visceral adipose tissue (VAT) could have important roles in inter‐organ communication leading to ectopic fat accumulation in the liver (i.e., non‐alcoholic fatty liver disease, NAFLD). However, despite the promising results based on the analyses of the contents of AT‐EV, the predicted EV‐mediated effects in the liver have been rarely studied in detail. In our experiments, we investigate how obesity affects the secretion and contents of AT‐EV. EV will be isolated from human adipocyte cell line and from ex vivo AT cultures of obese mice as well as bariatric surgery patients. In addition, we aim to provide the first, detailed mechanistic data of how AT‐EV promote NAFLD development and progression. To examine this, EV from patient AT ex vivo cultures and adipocyte cell line will be added into hepatocyte cultures in vitro, after which EV‐mediated changes, particularly in fatty acid metabolism, were studied.

**Methods**: EV were isolated from human Simpson Golabi Behmel Syndrome (SGBS) adipocyte cells and NAFLD patient ex vivo AT cultures by ultracentrifugation, and EV counts were measured by Nanoparticle Tracking Analysis. EV were transferred to hepatocyte (immortalized human hepatocyte (IHH), human hepatoma cell line HuH7) cultures. The effects of EV and VAT on hepatocyte fatty acid metabolism and signaling promoting NAFLD were studied by qPCR and RNAseq. The amount of fatty acids were analyzed by the fluorescent labeling of intracellular lipids and confocal microscopy. Fatty acid profiles of hepatocytes and EV were determined by mass spectrometry" gas chromatography.

**Results**: Our preliminary results suggest that patient VAT secretes more EV than subcutaneous AT. Furthermore, our analyses revealed that EV from mature SGBS cells contained high proportions of saturated fatty acids and, particularly, palmitic acid. SGBS‐EV interacted with IHH cells, and caused increased expression of sterol regulatory element‐binding protein 1c and acetyl coenzyme A carboxylase enzymes, together with increased number of lipid droplets.

**Summary/Conclusion**: Our results suggest that inflammation drives the EV secretion from AT, and that AT‐EV may induce NAFLD‐promoting effects.

### OD01.05 Lipidomic analysis of adipose‐derived extracellular vesicles reveals their potential as lipid mediators of obesity‐associated metabolic complications

Alexia BLANDIN , Institut du Thorax INSERM 1087

Grégory Hilairet, University of Angers

Maharajah Ponnaiah, ICANalytics

Marie Lhomme, ICANalytics

Soazig Le Lay, Institut du Thorax INSERM 1087

**Introduction**: Obesity‐related disorders are commonly associated with perturbations of lipid metabolism. Recent data show the ability of adipose extracellular vesicles (AdEV) to transport lipids that could participate in the development of metabolic dysfunctions. We aim to characterize the lipid content of AdEV in an healthy or obesity context in order to define their lipid signature and predict their role as mediators of metabolic disorders.

**Methods**: AdEV were purified from conditioned media of visceral adipose tissue (VAT) from control (Ob/+) or obese (Ob/Ob) mice by differential centrifugation to isolate large AdEV (13K) and small AdEV (100K). An untargeted mass spectrometry lipidomic approach was conducted to identify the whole sphingolipidome and phospholipidome as well as neutral lipids of AdEV, in comparison to secretory VAT.

**Results**: Lipidomic analysis of VAT reveals that lean tissue significantly differs from obese one at the level of both membrane lipid classes and molecular species. The most consistent changes in the obese VAT were the decrease in Lysophosphatidylethanolamines (LPE) and in PE plasmalogens (PEP) and the increase of Phosphatidylinositols (PI) and mitochondrial‐derived Phosphatidylglycerols (PG) in comparison to lean VAT. The PCA using lipid content data of AdEV and VAT shows a significant separation between both groups. Comprehensive lipidome analysis indeed reveals a specific EV enrichment of Ceramides (Cer) and its derivatives as well as in PG. EV lipidome was nonetheless more dependent of cell origin than on EV subtype, since obesity impacted AdEV lipid content of large and small AdEV in similar ways. For instance, obesity drives AdEV subtype enrichment in Triacylglycerols (TG) suggesting AdEV neutral lipid sorting independent of the canonical lipolytic pathway, and sphingomyelin (SM) enrichment at the expense of Cer illustrating dysregulated sphingolipid synthesis with obesity. Of interest, obese AdEV are particularly enriched in PG and arachidonic subspecies which might, respectively, contribute to mitochondrial dysfunction and chronic inflammation associated with obesity.

**Summary/Conclusion**: This study establishs for the first time the lipid fingerprint of VAT and their derived AdEV in a healthy and obesity context and highlights the AdEV potential as lipid mediators of obesity‐associated metabolic complications.

### OD01.06 Inhibition of neutral sphingomyelinase 2 reverses depression‐ and cognitive‐associated behaviors in EcoHIV‐infected mice

XIAOLEI ZHU , JHU

Kristen Hollinger, JHU

Yiyao Huang, MD, Johns Hopkins University School of Medicine

Tanina Arab, JHU

Alejandra Borjabad, Icahn School of Medicine at Mount Sinai

Boe‐Hyun Kim, Icahn School of Medicine at Mount Sinai

Ajit G. Thomas, Johns Hopkins University School of Medicine

Mohammed Moniruzzaman, JHU

Lyndah Lovell, JHU

Camilo Rojas,JHU

Atsushi Kamiya, JHU

Kenneth W. WitwerJohns Hopkins University School of Medicine

David Volsky, Icahn School of Medicine at Mount Sinai

Norman J. Haughey,Johns Hopkins University School of Medicine

Barbara S. Slusher,Johns Hopkins University School of Medicine

**Introduction**: To determine if depressive‐like behaviors are present in the EcoHIV mouse model of HIV‐induced neurocognitive disorders, and to evaluate the behavioral and biochemical effect of administering phenyl(R)‐(1‐(3‐(3,4‐dimethoxyphenyl)‐2,6‐dimethylimidazo[1,2‐b]pyridazin‐8‐yl)pyrrolidin‐3‐yl)‐carbamate (PDDC). PDDC is an orally bioavailable, selective and brain penetrable small molecule inhibitor of neutral sphingomyelinase 2 (nSMase2), an enzyme involved in the production of ceramide and extracellular vesicle (EV) biogenesis.

**Methods**: Mice were infected with EcoHIV and treated daily with either vehicle or PDDC starting at 3 weeks post‐infection. After two weeks of treatment, depressive‐like behaviors were evaluated using three‐chamber social approach test and forced swim testing. In parallel a separate cohort of mice was treated similarly and evaluated for cognitive function using the radial arm water maze. Subsequently, mice were sacrificed and brain tissues and plasma were collected to determine the effect of PDDC on nSMase2 enzymatic activity and sphingolipids. EVs were also isolated from brain tissue to determine the effect of PDDC on EV number and cargo.

**Results**: EcoHIV‐infected mice exhibited depressive‐like behaviors and cognitive impairment, both of which were reversed by PDDC. Brain nSMase2 activity was significantly elevated in EcoHIV infected mice, resulting in decreased sphingomyelin levels and increased ceramide levels. PDDC treatment restored these levels to baseline. EcoHIV infected mice also exhibited changes in brain‐derived EV levels, along with altered miRNA and protein cargo; all were normalized by PDDC.

**Summary/Conclusion**: Inhibition of nSMase2 represents a new therapeutic strategy for the treatment of HIV‐associated cognitive impairment and depression.

## OD02 EVs and Immunity

Chair: Marca H.M. Wauben, Department of Biomolecular Health Sciences, Utrecht Universit, The Netherlands

Chair: Phil Askenase, Yale University School of Medicine, United States

### OD02.01 Allergic sensitization of the mother influences the miRNA cargo and T cell modulatory properties of milk‐derived extracellular vesicles

Alberta Giovanazzi , Department of Biomolecular Health Sciences, Utrecht University, The Netherlands

Martijn van Herwijnen, Department of Biomolecular Health Sciences, Faculty of Veterinary Medicine, Utrecht University, The Netherlands.

Marijke I. Zonneveld, Postdoctoral researcher, GROW‐School for Oncology and Developmental Biology, Maastricht University, The Netherlands

Joaquín Jurado Maqueda, BIOINF2BIO, Porto, Portugal; i3S, Universidade do Porto, Portugal

Tom Driedonks, PhD, Johns Hopkins Medical School / Utrecht University

Marije Kleinjan, Department of Biomolecular Health Sciences, Utrecht University, The Netherlands

Gerbrich N. van der Meulen, Department of Paediatric Allergy, Martini Hospital, Groningen, The Netherlands

Johan Garssen, Division of Pharmacology, Department of Pharmaceutical Sciences, Utrecht University, The Netherlands

Ruurd M. van Elburg, Department of Pediatrics, Emma Children's Hospital/Academic Medical Center, Amsterdam, The Netherlands

Carla Oliveira,BIOINF2BIO, Porto, Portugal; i3S, Universidade do Porto, Portugal

Frank A. A. Redegeld, Division of Pharmacology, Department of Pharmaceutical Sciences, Utrecht University, The Netherlands

Esther N.M Nolte – ‘t Hoen, Dr.Utrecht University

Marca H.M. H.M. Wauben, Department of Biomolecular Health Sciences, Utrecht University, The Netherlands

**Introduction**: Allergic diseases can alter the physiological milk composition, but it is still unknown whether EV function and cargo are affected. Recently, we compared the functionality of EVs derived from non‐allergic and allergic mothers and we found a reduced T cell modulatory capacity in the latter group. In this study, we analyzed the miRNA cargo of milk EVs and used T cell signaling network analysis to predict possible relations between altered miRNA cargo and changed T cell modulatory activity.

**Methods**: EVs were purified from human milk of non‐allergic and allergic mothers (total serum IgE ‐ 50 kU/ml and/or positive Phadiatop assay for specific IgE) by differential centrifugation, density gradient floatation and size exclusion chromatography. Small RNA sequencing was performed to pinpoint qualitative and quantitative differences in milk EV miRNA cargo. A T cell signalling network model based on miRNA‐target interactions was built to predict hotspots of milk EV regulation.

**Results**: Transcriptomic analysis of milk EVs revealed that some miRNAs were differentially expressed among non‐allergic and allergic samples. Our prediction model shows that miRNAs overrepresented in non‐allergic samples favour the attenuation of T cell activation‐downstream processes such as cell cycle progression, STAT3/IL6 pathway and glucose metabolism, while miRNAs overrepresented in allergic samples target pro‐apoptotic pathway and negative regulators of cyclins.

**Summary/Conclusion**: Milk EVs from non‐allergic mothers are stronger inhibitors of T cell activation and this might be linked to quantitative differences in milk EV miRNA cargo between allergic and non‐allergic mothers.

### OD02.02 Umbilical cord blood‐derived small extracellular vesicles: immune‐modulating properties and regenerative potential for psoriatic lesions

Patricia Freire , Exogenus Therapeutics, S.A.

Silvia Rodrigues, Exogenus Therapeutics, S.A.

Patricia Freire, Exogenus Therapeutics, S.A.

Renato Cardoso, Exogenus Therapeutics, S.A.

Cláudia Gomes, Exogenus Therapeutics, S.A.

Ricardo Neves, Exogenus Therapeutics, S.A.; Center for Neurosciences and Cell Biology (CNC), University of Coimbra, Coimbra, Portugal; Institute for Interdisciplinary Research (3Is), University of Coimbra, Coimbra, Portugal

Joana Correia, Exogenus Therapeutics, S.A.; Center for Neurosciences and Cell Biology (CNC), University of Coimbra, Coimbra, Portugal

**Introduction**: Umbilical cord blood (UCB) has long been seen as a rich source of naïve cells with strong regenerative potential, likely mediated by the secretion of small extracellular vesicles (sEV). More recently, the immune‐modulating properties of stem‐cell‐derived sEV have attracted attention as a possible treatment for auto‐immune conditions. Despite their widely accepted use for transplantation into patients with blood disorders, UCB mononuclear cells (MNC) are seldomly studied for their regenerative and anti‐inflammatory potential.

**Methods**: With this work, we aimed to characterize the effect of UCB‐MNC‐sEV in different immune populations and determine their therapeutic effect in in vitro and in vivo models of psoriasis.

**Results**: In vitro, sEV were capable of shifting the profile of THP‐1 derived macrophages towards an anti inflammatory M2 phenotype, including in the presence of a pro‐inflammatory stimulus (LPS). In fresh PBMC, incubation with sEV resulted in a significant inhibition of total CD4+ and CD8+ T cell proliferation and pro‐inflammatory cytokine release (IFNg, TNFa, CCL20). Moreover, sEV were shown to influence the expression of transcriptional regulators T‐bet, RORgt and Foxp3, thereby supporting the development of regulatory T‐cells (Treg), while hindering Th1 and Th17 differentiation. In a 3D model of psoriatic epidermis, sEV strongly decreased the expression of antimicrobial peptides S100A7 and DEFB4, as well as of pro‐inflammatory mediators IL‐6, IL‐8, CXCL10 and COX‐2. Furthermore, in vivo, sEV significantly prevented or reversed epidermal thickening (acanthosis) in a model of imiquimod‐induced psoriasis, and tendentially increased the number of Treg in affected skin, while having no overall effect in macroscopic disease score.

**Summary/Conclusion**: In conclusion, this work provides evidence for the immune‐modulating effect of UCB‐MNC‐sEV, opening the door to the exploitation of this largely discarded material which may prove effective in the treatment of inflammatory skin diseases with high unmet needs, such as psoriasis.

### OD02.03 Molecular and functional signatures of distinct subpopulations of extracellular vesicles provide a rationale for beta‐cell mediated immune disease

Grégoire Mignot , IECM laboratory USC 1383 INRAE

Khem Giri, IECM, ONIRIS, INRAE, USC1383, Nantes, France

Laurence de Beaurepaire, IECM, ONIRIS, INRAE, USC1383, Nantes, France

Dominique Jegou, IECM, ONIRIS, INRAE, USC1383, Nantes, France

Margot Lavy, IECM, ONIRIS, INRAE, USC1383, Nantes, France

Mathilde Mosser, IECM, ONIRIS, INRAE, USC1383, Nantes, France

Aurélien Dupont, MRic, Biosit, UMS3480 CNRS, University of Rennes 1, Rennes, France

Romain Fleurisson, PAnTher, INRAE, Oniris, Université Bretagne Loire, Nantes, France

Laurence Dubreuil, PAnTher, INRAE, Oniris, Université Bretagne Loire, Nantes, France

Julien Pichon,PAnTher, INRAE, Oniris, Université Bretagne Loire, Nantes, France

mayeul Collot, Laboratoire de Biophotonique et Pharmacologie, UMR CNRS 7213, Université de Strasbourg, Illkirch, France

Peter van EndertINSERM, U1151, Institut Necker‐Enfants Malades, Paris, France

Jean‐Marie Bach, IECM, ONIRIS, INRAE, USC1383, Nantes, France

Steffi Bosch,IECM, ONIRIS, INRAE, USC1383, Nantes, France

**Introduction**: Evidences accumulates for an active role of the insulin‐producing pancreatic beta cell in diabetes development. As metabolically highly active cells, beta cells readily undergo cellular stress responses imposed by environmental changes. Beta cells release self‐antigens and microRNA inside extracellular vesicles (EV), fostering the idea that EV act as mediators in communication with immune effectors in diabetes development. While evidence accumulates on specific immune functions of subtypes of EV, the complete beta vesiculome has not been explored yet.

**Methods**: Here, we make quantitative and qualitative side‐by‐side comparisons of the phenotype and function of large apoptotic bodies (AB), microvesicles (MV) and small EV (sEV) isolated from an equal amount of insulin‐producing beta cells in normal and inflammatory settings.

**Results**: Under normal conditions, AB and MV represent 93% of the volume and 90% of the vesiculome's insulin load. None of the EV present detectable amounts of cytokines. Under inflammatory conditions, a consistently higher release of all EV subtypes is observed, commensurate with EV‐associated export of the autoantigen insulin, cytokines/chemokines and immune‐stimulatory microRNA. While the concentration of insulin inside the vesicles remains unchanged, the concentration of specific microRNA sequences raises specifically in sEV. Enrichment of MCP‐1 in all EV subtypes and of interleukin‐27 solely in AB suggests selective sorting inside EV subpopulations. Functional assays in mouse macrophage and dendritic cell cultures revealed differences in the aptitude of EV subtypes to modulate expression of cytokines and maturation markers.

**Summary/Conclusion**: Our findings highlight the different imprints of environmental changes in subpopulations of EV, whose relative contributions should provide new insights into the development of the global immune response.

### OD02.04 Small Extracellular Vesicles Propagate the Inflammatory Response After Trauma

Tanja Seibold , University Hospital Ulm

Jonathan Schönfelder, University Hospital Ulm

Florian Weeber, University Hospital Ulm

Milena Armacki, University Hospital Ulm

Andre Lechel, University Hospital Ulm

Markus Huber‐Lang, University Hospital Ulm

Miriam Kalbitz, University Hospital Ulm

Thomas Seufferlein, University Hospital Ulm

Tim Eiseler, University Hospital Ulm

**Introduction**: Trauma is the leading cause of death in individuals under 44 years of age. Thorax trauma (TxT) is strongly associated with trauma‐related death, an unbalanced innate immune response, sepsis, acute‐respiratory‐distress‐syndrome (ARDS), and multiple‐organ‐dysfunction (MODS), but also marked endothelial activation and inflammation has been described.

**Methods**: To investigate sEV secretion in response to trauma we used nanoparticle tracking analysis to characterize serum sEVs from mice after TxT, polytrauma (PT) and hemorrhagic shock (HS). Effects of sEVs on endothelial inflammation were elucidated by an in‐vitro trauma model using an inflammatory polytrauma cocktail (PTC). We assessed the role of sEVs for the post‐traumatic response in mice by injecting the sEV‐biogenesis inhibitor GW4869 10 min after TxT. Vice versa, TxT‐plasma‐sEVs were injected into healthy animals to investigate regulation of inflammation and endothelial barrier stability. Results were validated by analyzing PT‐patient plasma‐sEV.

**Results**: We show that different in‐vivo traumata, or PTC in‐vitro trigger secretion of sEVs from endothelial cells with pro‐inflammatory cargo. These sEVs transfer transcripts for adhesion molecules and cytokines, but also miRNAs to both propagate inflammation and destabilize endothelial barriers, respectively. Inhibition of sEV‐release after TxT in mice ameliorated local as well as systemic inflammation, neutrophil infiltration, and secondary acute kidney injury (AKI). In turn, injection of TxT‐plasma‐sEVs into healthy animals was sufficient to trigger pulmonary and systemic inflammation as well as AKI. Moreover, increased sEV concentrations and transfer of similar cargos was observed in polytrauma patients.

**Summary/Conclusion**: We show that diverse traumatic insults significantly increase sEV secretion and identify endothelial cells as major source. In summary, we here described a so‐far neglected role for endothelial‐derived sEVs in the transmission of local and systemic post‐traumatic inflammation.

### OD02.05 Cytosolic dsDNA and extracellular vesicles participate to antitumor immunity induced by external and targeted radiotherapy

Julie Constanzo , IRCM, Institut de Recherche en Cancérologie de Montpellier, INSERM U1194, Université de Montpellier, Institut régional du Cancer de Montpellier

Jihad Karam, Institut de Recherche en Cancérologie de Montpellier

Alexandre Pichard, IRCM, Institut de Recherche en Cancérologie de Montpellier, INSERM U1194, Université de Montpellier, Institut régional du Cancer de Montpellier

Julien Faget, IRCM, Institut de Recherche en Cancérologie de Montpellier, INSERM U1194, Université de Montpellier, Institut régional du Cancer de Montpellier

Frank Bruchertseifer, European Commission ‐ Joint Research Centre

Alfred Morgenstern, European Commission ‐ Joint Research Centre

Isabelle Villa, IGH, Institut de Génétique Humaine, CNRS, Université de Montpellier, Molecular Basis of Inflammation Laboratory

Nadine Laguette, IGH, Institut de Génétique Humaine, CNRS, Université de Montpellier, Molecular Basis of Inflammation Laboratory, Montpellier

Nathalie Bonnefoy, IRCM, Institut de Recherche en Cancérologie de Montpellier, INSERM U1194, Université de Montpellier, Institut régional du Cancer de Montpellier

Jean‐Pierre Pouget,IRCM, Institut de Recherche en Cancérologie de Montpellier, INSERM U1194, Université de Montpellier, Institut régional du Cancer de Montpellier

**Introduction**: Beside conventional external beam radiotherapy (EBRT) using X‐rays dedicated to localized tumors, targeted radionuclide therapy (TRT) allows to specifically irradiate diffuse and metastatic tumors. TRT consists of the administration of radiopharmaceuticals made of monoclonal antibodies or peptides coupled to a radionuclide emitting alpha, beta and Auger particles. Here, we investigate the role of X‐rays, alpha, Auger and beta particles in triggering systemic effects through the activation of cGAS‐STING pathway in tumor and host immune cells and assessed the role of extracellular vesicles (EVs) in intercellular communications.

**Methods**: B16F10 melanoma cells were subcutaneously injected in C57BL/6J and athymic mice. Mice received intraperitoneal injections of TA99 mAb targeting TYRP‐1/gp75 tumor antigen radiolabeled either with 225Ac (1‐ 9.25 kBq; 74MBq/mg, alpha‐TRT) or 125I (2‐ 27 MBq; 37MBq/mg, Auger‐TRT), or with EVs purified from non‐treated cells or cells exposed to 2MBq/ml 177Lu‐TA99 (200 MBq/mg, beta‐TRT).

**Results**: In vivo, alpha‐ and Auger‐TRT efficacy was shown to require T‐cells for adaptive immunity. Median survival was 16 days and 29 days for alpha and Auger TRT, respectively versus 11 and 15 days for controls (**p = 0.0035). No difference with controls was observed in athymic nude mice. In vitro, B16F10 cells exposed to Auger‐TRT, demonstrated a protracted accumulation of cytosolic dsDNA over 48h as compared with beta‐TRT or X‐rays. In addition, an early and persistent activation of cGAS‐STING pathway was observed, from 1h to 48h following the beginning of TRT. However, knocked‐out cGAS genes in host immune cells did not affect Auger‐TRT response in vivo, while knocked‐out STING genes did, suggesting that dsDNA is not the only extracellular messenger involved in activation of immune cells. We therefore focused on extracellular vesicles as a second messenger released by cancer cells that may activate an antitumor immune response through the STING pathway. EVs purified from B16F10 cells exposed to beta‐TRT contained 3.5.10‐3 fg of dsDNA. Compared to EVs purified from non‐treated cells, 4 intratumoral injections of EVs‐Beta demonstrated a strong tumor growth delay and survival (***p = 0.0007) in C57Bl/6J mice, while no difference were observed in athymic nude mice.

**Summary/Conclusion**: Radiation‐induced cytosolic dsDNA and EVs mediate radiation‐induced systemic response in vivo.

### OD02.06 The effects of mesenchymal stromal cells‐derived small extracellular vesicles on dendritic cells via IL‐10 in patients with allergic rhinitis

Ya‐Qi Peng, The First Affiliated Hospital, Sun Yat‐sen University

Qing‐Ling Fu , The First Affiliated Hospital, Sun Yat‐sen University

**Introduction**: Mesenchymal stromal cells (MSCs) are well known as their immunoregulatory roles on allergic inflammation particularly by acting on T cells, B cells, and dendritic cells (DCs). MSC‐derived small extracellular vesicles (MSC‐sEV) are increasingly considered as one of the main factors for the effects of MSCs on immune responses. However, the effects of MSC‐sEV on DCs in allergic diseases remain unclear.

**Methods**: MSC‐sEV were prepared from the induced pluripotent stem cells (iPSC)‐MSCs by anion‐exchange chromatography, and were characterized with the size, morphology, and specific markers. Human monocyte‐derived DCs were generated and cultured in the presence of MSC‐sEV to differentiate the so‐called sEV‐immature DCs (sEV‐iDCs) and sEV‐mature DCs (sEV‐mDCs), respectively. The phenotypes and the phagocytic ability of sEV‐iDCs were analyzed by flow cytometry. sEV‐mDCs were co‐cultured with isolated CD4+T cells or peripheral blood mononuclear cells (PBMCs) from patients with allergic rhinitis (AR). The levels of Th1 and Th2 cytokines produced by T cells were examined by ELISA and intracellular flow staining, and the following mechanisms were further investigated.

**Results**: We demonstrated that MSC‐sEV inhibited the differentiation of human monocytes to iDCs with down‐regulation of the expression of CD40, CD80, CD86, and HLA‐DR, but had no effects on the surface markers of mDCs. However, sEV treatment enhanced the antigen uptake of mDC. In addition, sEV‐mDCs suppressed the Th2 immune response by reducing the production of IL‐4, IL‐9, and IL‐13. Moreover, the treatment of neutralizing anti‐IL‐10 antibodies led to a significant reversal in levels of IL‐13+CD4+T cells, IL‐9 and IL‐13 production from T cells compared to the sEV‐mDCs alone. Additionally, the administration of sEV‐mDCs upregulated the levels of IL‐10+CD4+T cells and CD4+CD25+Foxp3+ Treg cells.

**Summary/Conclusion**: Our study identified that mDCs treated with MSC‐sEV exhibited the inhibition of Th2 responses, providing a novel evidence of the potential cell‐free therapy of on DCs in allergic airway diseases.

### OD02.07 Red blood cells‐derived extracellular vesicles decrease Neutrophils survival, phagocytosis, and ROS production

Getulio P. Oliveira, Ph.D., Beth Israel Deaconess Medical Center

Brandy Pinckney, Beth Israel Deaconess Medical Center

Shulin Lu, Beth Israel Deaconess Medical Center

Alan Zimmerman, Northeastern University

John Tigges, Beth Israel Deaconess Medical Center

Alexander Ivanov, Northeastern University

Ionita C. Ghiran, MD, Beth Israel Deaconess Medical Center

**Introduction**: Extracellular vesicles (EVs) are membrane‐bound entities released by cells and tissues into biofluids involved in cell‐cell communication. Circulating red blood cells (RBCs), the most numerous cell‐type in the body, generate large numbers of EVs daily. The fate of the RBC‐EVs is currently unknown.

**Methods**: RBC plasma membranes were labeled with a red fluorescent dye, and EVs were generated by either Ionomycin 10 μM or A23187 10 μM for 1h. RBC‐EVs were purified by size exclusion chromatography (SEC), and characterized using microscopy (fluorescence and electron (EM)), nanoflow cytometry, western blotting, tunable pulse sensing, and LC‐MS‐based proteomics. EV uptake was measured by flow cytometry, fluorescence microscopy and cryo‐EM. The biological function of RBC‐EVs on neutrophil was assessed by survival assay at 24h (Annexin 5‐PI staining), phagocytosis of fluorescent E. coli BioParticles after 2h, and ROS production after 4h.

**Results**: The purity of RBCs‐EVs was confirmed by western blots and immuno‐EM, using anti‐CD235a and anti‐Band3 antibodies. Quantitative proteomic profiling revealed the enrichment of sorcin, stomatin, annexin A7, and RAB proteins into RBC‐EVs. Immuno‐EM showed two distinct populations of RBC‐EVs (positive and negative) for CD235a. Furthermore, we detect two mechanisms of RBC‐EVs uptake by neutrophils: fusion of the plasma membrane and complete internalization. RBC‐EVs were rapidly uptaken by neutrophils, even after 10 min of incubation, increasing the number of late apoptotic cells (Annexin 5‐ and PI‐positive cells) after 24h. The neutrophils' ability to phagocyte E. coli was slightly decreased after incubating with RBC‐EVs for 2h. Furthermore, the neutrophils production of reactive oxygen species (ROS) was impaired after 4h of incubation.

**Summary/Conclusion**: This work brings new insights into the communication among RBC‐EVs to PBMCs, and neutrophils in circulation.

## OD03 Brain and Aging

Chair: Laura Vella, The Florey Institute of Neuroscience and Mental Health, Australia

Chair: Sowmya Yelamanchili, Department of Anesthesiology, United States

### OD03.01 Astrocyte‐derived small extracellular vesicles promote synapse formation via fibulin‐2 mediated TGF‐beta signaling

Mikin Patel, Vanderbilt University

Alissa Weaver, Department of Cell and Developmental Biology, Vanderbilt University School of Medicine

**Introduction**: Synapses are specialized neuronal structures that are critical for neuronal communication. Dendritic spines are postsynaptic membrane specializations that are critical for synapse formation and function. Previous studies have shown that astrocyte conditioned media can enhance neuronal spine and synapse formation, suggesting the importance of molecules secreted by astrocytes. Here, we examine the role of astrocyte‐derived small extracellular vesicles in the formation of neuronal dendritic spines and synapses.

**Methods**: Small extracellular vesicles (SEVs) were isolated from primary cortical neurons (CNSEVs), primary astrocytes (ADSEVs) and C6 glioma cells (C6SEVs) using differential ultracentrifugation. Purified SEVs were characterized for their size, morphology and common markers using NTA, TEM and Western blot. Day in vitro 10 cortical neurons were treated for 48 h with increasing doses of purified SEVs to analyze their effect on dendritic spines and synapses. iTRAQ proteomics analysis was performed to identify unique synaptogenic cargo present in ADSEVs. Phosphorylated Smad2 (pSmad2) was measured to examine activation of TGF‐beta signaling.

**Results**: Here, we find that ADSEVs, but not CNSEVs or C6SEVs, enhance dendritic spine and synapse formation. iTRAQ proteomics analysis revealed that ADSEVs are enriched in proteins distinct from CNSEVs or C6SEVs, including fibulin‐2, an extracellular matrix protein known to activate TGF‐beta. We find that treatment with fibulin‐2‐knockdown ADSEVs does not induce synaptogenesis, whereas treatment with recombinant fibulin‐2 induces synaptogenesis. Treatment of neurons with recombinant fibulin‐2 or ADSEVs leads to an increase in pSmad2, suggesting activation of TGF‐beta signaling. Also, the synaptogenic effects of fibulin‐2 or ADSEVs are reversed by inhibiting TGF‐beta signaling. Finally, the increase in pSmad2 level by ADSEVs is only slightly diminished by the presence of the endocytosis inhibitor Dynasore, suggesting that activation of TGF‐beta signaling by ADSEVS is likely to occur at the cell surface.

**Summary/Conclusion**: These results show that fibulin‐2 carried by astrocyte‐derived SEVs promotes formation of neuronal dendritic spines and synapses by activating TGF‐beta signaling.

### OD03.02 Phenotypic and functional analysis of nigrostriatal astrocyte‐derived extracellular vesicles reveals an intrinsic brain area‐dependent neuroprotective potential in Parkinson's disease models

Loredana Leggio, Department of Biomedical and Biotechnological Sciences, University of Catania, Italy

Francesca L'Episcopo, Neuropharmacology Section, OASI Research Institute‐IRCCS, 94018 Troina (EN), Italy

Andrea Magrì, Department of Biological, Geological and Environmental Sciences, University of Catania, Italy

María José Ulloa‐Navas, Institute Cavanilles, University of Valencia, Valencia, Spain

Greta Paternò, Department of Biomedical and Biotechnological Sciences, University of Catania, Italy

Silvia Vivarelli, Department of Biomedical and Biotechnological Sciences, University of Catania, Italy

Cataldo tirolo, Neuropharmacology Section, OASI Research Institute‐IRCCS, 94018 Troina (EN), Italy

Nunzio Testa, Neuropharmacology Section, OASI Research Institute‐IRCCS, 94018 Troina (EN), Italy

Salvatore Caniglia, Neuropharmacology Section, OASI Research Institute‐IRCCS, 94018 Troina (EN), Italy

Carlos Bastos, University of Cambridge, Department of Veterinary Medicine, Cambridge, United Kingdom

Pierpaolo Risiglione, Department of Biomedical and Biotechnological Sciences, University of Catania, Italy

Nuno FariaUniversity of Cambridge, Department of Veterinary Medicine, Cambridge, United Kingdom

Stefano Pluchino, University of Cambridge, Department of Clinical Neurosciences, Cambridge, United Kingdom

Jose Manuel Garcia‐Verdugo, Institute Cavanilles, University of Valencia, Valencia, Spain

Angela Messina, Department of Biological, Geological and Environmental Sciences, University of Catania, Italy

Bianca Marchetti, Department of Biomedical and Biotechnological Sciences, University of Catania, Italy

Nunzio Iraci, Department of Biomedical and Biotechnological Sciences, University of Catania, Italy

**Introduction**: Parkinson's disease (PD) is characterized by the progressive degeneration of dopaminergic neuronal cell bodies in the ventral midbrain (VMB) and their terminals the striatum (STR), with consequent dopamine depletion. In this context, astrocytes (AS) play either destructive or beneficial roles and, when activated by the chemokine CCL3, they exert a robust neuroreparative action both in cellular and pre‐clinical PD models. We herein isolated EVs derived from VMB‐ and STR‐ AS, both in basal and CCL3 conditions, to evaluate their possible involvement in the complex cross‐talk between AS and neurons.

**Methods**: EVs were purified from AS supernatants by ultracentrifugation and characterized by nanoparticle tracking analysis, immunogold‐transmission electron microscopy and western blotting. AS‐EV functional effects were evaluated on differentiated SH‐SY5Y cells under neurodegenerative conditions using immunofluorescence and high resolution respirometry (HRR).

**Results**: AS secrete vesicles with a dimension of "100 nm, positive for CD63, CD9 and Alix markers. In basal conditions, VMB‐AS release more EVs than STR‐AS, and only VMB‐AS respond to CCL3 by producing more EVs. Following internalization by SH‐SY5Y cells, we tested AS‐EV effects under H2O2 treatment. We found that both basal but mostly CCL3 AS‐EVs induce a significant reduction of Caspase 3 activation in oxidative conditions. Moreover, we tested EV effects in the presence of MPP+ PD neurotoxin and measured the mitochondrial functionality by HRR. We observed an important recover of complex I activity in the presence of AS‐EVs. Interestingly, during ATP production, the reduction of O2 flux and ADP phosphorylation caused by MPP+ was specifically restored by VMB‐AS‐EVs.

**Summary/Conclusion**: For the first time, to our knowledge, our study shows the existence of specific brain region‐linked mechanism(s) for AS‐EV secretion with possible functional implications in the intercellular communication within the nigrostriatal area, in the context of PD.

### OD03.03 Cell type‐specific extracellular vesicles define disease‐related protein networks associated with astrocyte activation in Alzheimer's disease

Yang YOU, Boston university

Satoshi Muraoka, Department of Pharmacology & Experimental Therapeutics, Boston University School of Medicine, Boston, MA, USA

Mark P. Jedrychowski, Department of Cell Biology, Harvard Medical School, Boston, MA, USA

Shuiqiao Hu, Department of Pharmacology & Experimental Therapeutics, Boston University School of Medicine, Boston, MA, USA

Amanda K. McQuade, Department of Neurobiology and Behavior, Institute for Memory Impairments and Neurological Disorders, University of California, Irvine, CA, USA

Tracy Young‐Pearse, Department of Neurology, Brigham and Women's Hospital, Harvard Medical School, Boston, MA, USA

Roshanak Aslebagh, Department of Biochemistry and Molecular Pharmacology, University of Massachusetts Medical School, Worcester, MA, USA

Mohammad Abdullah, Department of Pharmacology & Experimental Therapeutics, Boston University School of Medicine, Boston, MA, USA

Scott A. Shaffer, PhD, Department of Biochemistry and Molecular Pharmacology, University of Massachusetts Medical School, Worcester, MA, USA

Mathew Blurton‐Jones,Department of Neurobiology and Behavior, Institute for Memory Impairments and Neurological Disorders, University of California, Irvine, CA, USA

Wayne W. Poon, Department of Neurobiology and Behavior, Institute for Memory Impairments and Neurological Disorders, University of California, Irvine, CA, USA

Steven P. GygiDepartment of Cell Biology, Harvard Medical School, Boston, MA, USA

Tsuneya Ikezu, MD, PhD, Department of Pharmacology & Experimental Therapeutics, Center for Systems Neuroscience,Boston University School of Medicine; Department of Neuroscience, Mayo Clinic Florida

**Introduction**: Extracellular vesicles (EVs) have gathered great interest in studying neurodegenerative diseases including Alzheimer's disease (AD) with the capability of transferring pathogenic molecules. Almost every cell type in the central nervous system (CNS) including neurons and glia are known to shed EVs. Capturing cell type‐specific EVs from patient‐derived samples and profiling their contents by transcriptomic or proteomic analyses provide a useful method to study the pathophysiology of AD. Indeed, recent studies immunoprecipitated CNS‐specific EVs from AD samples via specific antibodies and obtained promising results. However, a consensus on cell type‐specific EV markers is lacking due to the limited evidence of cell type‐specific EV proteomic datasets from human samples. To address these concerns, we sought to define human CNS cell type‐specific EV protein signatures that could be employed for cell type EV isolation, and investigate their potential roles in AD pathology.

**Methods**: We performed combined label‐free and TMT‐labeling based quantitative mass‐spectrometry of EVs isolated from human induced pluripotent stem cells (hiPSCs) and AD brain tissues to conduct a comprehensive EV proteomics study. The weighted protein co‐expression network analysis (WGCNA) was used to generate AD‐associated and cell type‐specific EV protein modules. The disease‐related proteins were further validated by purifying cell type EVs from AD brain using an independent cohort.

**Results**: Novel cell type‐specific EV protein markers were identified from hiPSC‐derived excitatory neurons (e.g., NCAM1, ATP1A3), astrocytes (e.g., LRP1, ITGA6), microglia‐like cells (e.g., CD300A, ITGAM) and oligodendrocytes (e.g., LAMP2, FTH1). WGCNA of brain‐derived EV proteomics from 11 healthy controls, 8 mild cognitive impairment and 11 AD patients identified a protein module, which were enriched with astrocyte‐derived EV markers and plasma membrane molecules, was most significantly associated with AD pathology and cognitive function. These proteins are biased towards the EV profile of activated astrocytes and significantly involved in inflammatory processes. We validated the elevated expression of ITGB1, a hub protein within the module, in AD astrocyte‐specific EVs by using an independent cohort.

**Summary/Conclusion**: Our study presents novel human CNS cell type‐specific EV markers, highlights the key role of astrocyte‐derived EVs in AD pathogenesis, and provides a featured framework for future EV studies on neurodegenerative diseases.

### OD03.04 Measuring biomarkers for Parkinson's Disease using neuronal‐origin extracellular vesicles

Joseph M. Blommer, National Institute on Aging

Toni Pitcher, New Zealand Brain Research Institute

Wassilios Meissner, Institute of Neurodegenerative Disorders, University Bordeaux

Maja Mustapic, PhD, National Institute on Aging

Tim Anderson, New Zealand Brain Research Institute

Dimitrios Kapogiannis, National Institute on Aging

**Introduction**: Parkinson's Disease (PD) pathogenesis involves intraneuronal a‐synuclein accumulation, but also impaired insulin sensitivity, which is characterized by imbalance in Tyr and Ser insulin receptor substrate‐1 (IRS‐1) phosphorylations. Besides motor symptoms, some PD patients develop mild cognitive impairment (PD‐MCI) or dementia (PD‐D), perhaps as a result of Alzheimer's disease (AD) pathology (amyloid‐beta plaques and hyperphosphorylated tau tangles) developing concurrently with a‐synuclein pathology. Given its importance for disease prognosis, there is a need to develop biomarkers for distinguishing PD with normal cognition (PD‐N) from PD‐MCI/D. Neuronal‐origin extracellular vesicles (NEVs) contain cell signaling and pathogenic proteins that may serve as biomarkers for Alzheimer's disease and PD.

**Methods**: From 104 PD‐N, 83 PD‐MCI, and 39 PD‐D patients and 48 age and sex‐matched Controls, we immunocaptured plasma NEVs using anti‐L1CAM antibody. We measured biomarkers by electrochemiluminescence immunoassays for: 1) PD and AD hallmark pathogenic proteins (a‐synuclein, amyloid‐beta42, total and p181‐Tau 2) pTyr20 and pSer312‐IRS‐1 and 3) synaptic proteins reflecting synaptic degeneration. Particle concentration was measured in all samples by Nanoparticle Tracking Analysis and immunoblots were used to characterize NEV preparations.

**Results**: A‐synuclein was lower in PD compared to Controls (p < 0.01) and stepwise in PD‐MCI and PD‐D compared to PD‐N (p < 0.01) and tended to decrease with increasing motor symptom severity by MDS‐UPDRS III (p = 0.06). Amyloid‐beta42 trended towards being higher in PD‐MCI and PD‐D groups compared to PD‐N (p = 0.06). pTau181 was higher in PD patients compared to Controls (p < 0.005) and in PD‐MCI compared to PD‐N (p < 0.05). Total Tau was not different between groups. pTyr20‐IRS‐1 was lower in PD compared to Controls and in PD‐MCI compared to PD‐N (p < 0.05) and decreased with increasing motor symptom severity by MDS‐UPDRS III (p < 0.01). The ratio pSer312/pTyr20 IRS‐1 (indicating insulin resistance) was higher in PD patients compared to Controls (p < 0.05) and in PD‐MCI and PD‐D compared to PD‐N (p < 0.05). Synaptophysin and synaptopodin were not different between groups.

**Summary/Conclusion**: PD patients with cognitive impairment exhibited lower NEV levels of a‐synuclein and pTyr20‐IRS‐1and higher levels of pTau181 than cognitively intact PD patients. Additionally, a‐synuclein and IRS‐1pTyr20 were associated with PD motor symptom severity. Plasma NEVs are a valuable tool for discovering biomarkers in PD and investigating aspects of disease progression.

### OD03.05 Flow cytometry detection of membrane nanoparticles with size and cargo characteristics of spontaneously released EVs from brain

Carlos J. Nogueras‐Ortiz, NIH/NIA/LCI

Christopher Dunn, NIH/NIA/LCI

Ana P. Amorim Gomes, University of Minho

Ioannis Sotiropoulus, University of Minho

Dimitrios Kapogiannis, National Institute on Aging

**Introduction**: We sought to determine the relative abundance of tetraspanins known to be enriched in distinct EV subpopulations and hence used as EV markers, in intact spontaneously‐released EVs from perfused mouse brain tissue, using high sensitivity nanoscale flow cytometry analysis combining side scatter‐ and fluorescence‐based particle detection.

**Methods**: Side scatter of particles isolated via ultracentrifugation (3,000g, then 100,000g) was detected using a 405 nm (violet) wavelength laser (vSSC) which has been demonstrated to improve EV resolution and to be a better indicator of particle size compared to 488 nm (blue) wavelength lasers.

**Results**: Simultaneous labelling of EVs with APC‐tagged anti‐CD9, CD63 and CD81 antibodies resulted in the detection of APC+ events with a vSSC range within that of fluorescent beads and fluorescent EVs isolated from YFP+ HEK cells, with sizes ranging from 100–1300 nm, whereas negative controls, including water, antibody‐alone and EVs labelled with isotype controls, showed low electronic noise and no signs of particle detection. APC+ nanoparticles were not detected after treatment with NP40 detergent, thus confirming their membrane composition.

To explore the relative abundance of tetraspanins, we assessed EV subpopulations positive for either CD9 or CD81, and both. First, we confirmed the capacity of our flow cytometry analysis to distinguish between single‐ and double‐positive events by comparing mixed EVs individually labelled with APC‐CD9 and PE‐CD81, and simultaneously labelled EVs. APC/PE double‐positive events were only detected when simultaneously labelling EVs, and not when individually labelled EVs were mixed prior to analysis, indicating the detection of double‐positive single nanoparticles in the absence of coincidental events due to swarming. Further analysis of simultaneously labelled EVs showed that 98% of APC‐CD9+ events are PE‐CD81+, whereas 42% of PE‐CD81+ events are APC‐CD9+.

**Summary/Conclusion**: These results concur with previous observations suggesting that CD81 is a specific marker of small EVs, whereas CD9 and CD63 are present in multiple EV subpopulations and validate the isolation of membrane nanoparticles with size and cargo characteristics of spontaneously released EVs from mouse brain tissue.

### OD03.06 Plasma extracellular vesicle‐associated mitochondrial DNA declines with age

Nicole Noren Hooten, National Institute on Aging, National Institutes of Health

Stephanie Lazo, National Institute on Aging, National Institutes of Health

Jamal Green, National Institute on Aging, National Institutes of Health

Erez Eitan, National Institute on Aging, National Institutes of Health

Nicolle Mode, National Institute on Aging, National Institutes of Health

Qing‐Rong Liu, National Institute on Aging, National Institutes of Health

Alan Zonderman, National Institute on Aging, National Institutes of Health

Ngozi Ezike, National Institute on Aging, National Institutes of Health

Mark Mattson, National Institute on Aging, National Institutes of Health

Paritosh Ghosh,National Institute on Aging, National Institutes of Health

Michele Evans, National Institute on Aging, National Institutes of Health

**Introduction**: Aging is associated with the progressive decline in organ and tissue function over the lifetime leading to age‐associated diseases. Mitochondrial dysfunction is a factor that drives the aging process. Cellular mitochondrial DNA (mtDNA) can be released outside of the cell as circulating cell‐free mtDNA (ccf‐mtDNA) and can be measured from blood. Higher levels of ccf‐mtDNA have been detected in cancer and inflammatory diseases, suggesting that they may be indicators of health and disease. Mitochondrial components may be encapsulated in EVs, yet little is known about whether ccf‐mtDNA can be detected in EVs from the circulation and whether there are changes in ccf‐mtDNA in the context of normal physiological processes such as aging.

**Methods**: Here we examined ccf‐mtDNA in plasma‐derived EVs from a cross‐sectional and longitudinal cohort of individuals across the lifespan. DNA was isolated from plasma EVs and the EV‐depleted fractions and analyzed by quantitative real‐time PCR using four primer sets that were designed against different regions of the mitochondrial genome. EVs from young and old individuals were used to treat cells in vitro to determine the effects on mitochondrial energetics.

**Results**: We report that plasma‐derived ccf‐mtDNA can be encapsulated in EVs. Furthermore, we examined EV‐mtDNA levels across the lifespan and found that EV‐mtDNA levels significantly decline with age in both our cross‐sectional and longitudinal analyses. We tested whether EV‐age altered mitochondrial function. Basal and maximal respiration were higher in cells treated with young EVs compared to old EVs.

**Summary/Conclusion**: Our results indicate that plasma EVs can carry ccf‐mtDNA as cargo. Furthermore, we found that plasma EV‐derived mtDNA declines with advancing age, which may impact cellular mitochondrial function. These data shed new light on the relationship between EVs, EV cargo and age that may help guide the usage of EVs and their content as biomarkers of health.

### OD03.07 ESC‐derived sEVs Rejuvenate Aging Hippocampal NSCs by Transferring SMADs to Regulate the MYT1‐Egln3‐Sirt1 Axis

Yuguo Xia, Department of Neurosurgery, Shanghai Jiao Tong University Affiliated Sixth People's Hospital

Zhifeng Deng, Department of Neurosurgery, Shanghai Jiao Tong University Affiliated Sixth People's Hospital

Guowen Hu, Department of Neurosurgery, Shanghai Jiao Tong University Affiliated Sixth People's Hospital

Yuguo Xia, Department of Neurosurgery, Shanghai Jiao Tong University Affiliated Sixth People's Hospital

Qing Li, Institute of Microsurgery on Extremities, Shanghai Jiao Tong University Affiliated Sixth People's Hospital

Yang Wang, Institute of Microsurgery on Extremities, Shanghai Jiao Tong University Affiliated Sixth People's Hospital

Zhifeng Deng, Department of Neurosurgery, Shanghai Jiao Tong University Affiliated Sixth People's Hospital

**Introduction**: Tissue stem cell senescence leads to stem cell exhaustion, which results in tissue homeostasis imbalance and a decline in regeneration capacity. However, whether neural stem cell (NSC) senescence occurs and causes neurogenesis reduction during aging is unknown. In this study, we aimed to investigate NSC senescence during aging and the effect of embryonic stem cell‐derived small extracellular vesicles (ESC‐sEVs) on rejuvenating NSC senescence as well as the underlying mechanism.

**Methods**: In this study, mice at different ages were used to detect age‐related hippocampal NSC (H‐NSC) senescence, as well as the function and mechanism of ESC‐sEVs in rejuvenating H‐NSC senescence.

**Results**: We found a progressive cognitive impairment, as well as age‐related H‐NSC senescence, in mice. ESC‐sEVs treatment significantly alleviated H‐NSC senescence, recovered compromised self‐renewal and neurogenesis capacities, and reversed cognitive impairment. Transcriptome analysis revealed that myelin transcription factor 1 (MYT1) is downregulated in senescent H‐NSCs but upregulated by ESC‐sEVs treatment. In addition, knockdown of MYT1 in young H‐NSCs accelerated age‐related phenotypes and impaired proliferation and differentiation capacities. Mechanistically, ESC‐sEVs rejuvenated senescent H‐NSCs partly by transferring SMAD family members 4 (SMAD4) and 5 (SMAD5) to activate MYT1, which downregulated egl‐9 family hypoxia‐inducible factor 3 (Egln3), followed by activation of hypoxia‐inducible factor 2 subunit a (HIF‐2a), nicotinamide phosphoribosyltransferase (NAMPT), and sirtuin 1 (Sirt1) successively.

**Summary/Conclusion**: Taken together, our results indicated that H‐NSC senescence caused cellular exhaustion, neurogenesis reduction, and cognitive impairment during aging, which can be reversed by ESC‐sEVs. Thus, ESC‐sEVs may be promising therapeutic candidates for age‐related diseases.

## OD04 EVs in Cancer Pathogenesis I

Chair: Fabrice Lucien, Mayo Clinic, United States

Chair: Takahiro Ochiya, Department of Molecular and Cellular Medicine, Tokyo Medical University

### OD04.01 Stress‐induced extracellular vesicles enriched in Rab11a‐exosomes promote cetuximab resistance in colorectal cancer cells through activation of EGFR signalling

John D. Mason, University of Oxford

Ewan Marks, Department of Physiology, Anatomy & Genetics, University of Oxford

Shih‐Jung Fan, MD PhD, Department of Physiology, Anatomy & Genetics, University of Oxford

Clive Wilson, Department of Physiology, Anatomy & Genetics, University of Oxford

Freddie Hamdy, Nuffield Department of Surgical Sciences, University of Oxford

Adrian Harris, Department of Oncology, Weatherall Institute of Molecular Medicine, University of Oxford

Chris Cunningham, Nuffield Department of Surgical Sciences, University of Oxford

Deborah C I Goberdhan, PhD, Department of Physiology, Anatomy & Genetics, University of Oxford

**Introduction**: We recently demonstrated that metabolic stress leads to the preferential release of extracellular vesicles (EVs) containing exosomes made in Rab11a‐labelled recycling endosomes from a panel of colorectal cancer cell lines. These vesicles carry increased levels of Amphiregulin (AREG), which promote EGFR‐ERK dependent growth in recipient cells. Here, we test whether these properties are transferable to other colorectal cancer cell lines and their role in promoting drug resistance in KRAS‐wild type colorectal cancer cells.

**Methods**: EVs were isolated using size exclusion chromatography from HCT116 cells (mutant KRAS) grown under glutamine‐replete and ‐depleted conditions, and their effect on the growth of Caco2 recipient cells (KRAS¬‐wild type) was measured. In addition, we tested whether the inhibitory effects of the clinically used anti‐EGFR antibody, cetuximab, and an intracellular EGFR kinase inhibitor, could be reversed by co‐treatment with switched EVs.

**Results**: Switched EVs isolated from HCT116 secreting cells strongly promoted AREG‐dependent growth of Caco2 recipient cells. Caco2, but not HCT116, cell growth was inhibited by increasing doses of cetuximab which could be partially reversed by co‐treatment of switched EVs. Consistent, with this being an AREG‐dependent mechanism, this property was lost by treating EVs with an AREG neutralising antibody. Furthermore, switched EVs were unable to rescue Caco2 cells treated with downstream EGFR kinase domain inhibitors, providing evidence that the EV‐induced drug resistance is mediated by binding of EV‐associated AREG to the EGFR, despite the low levels of AREG on these vesicles.

**Summary/Conclusion**: We conclude that switched EVs are important mediators of colorectal cancer cell growth and also cetuximab resistance via a competition or displacement mechanism, and could serve as useful novel biomarkers to predict response to treatment.

### OD04.02 Altered cargo of EVs from mTORC1‐driven tumours enhances pro‐tumoral signalling in recipient fibroblasts of the tumour microenvironment

Muireann Ní Bhaoighill, Cardiff University

Andrew R. Tee, Cardiff University

Jason P. Webber, Swansea University

Elaine Dunlop, Cardiff University

**Introduction**: Loss‐of‐function of TSC1/2 growth suppressor genes enables hyperactivation of mammalian Target Of Rapamycin Complex 1 (mTORC1), which causes tumour growth. Subsequent inhibition of mTORC1 in cancer has limited impact, suggesting that feedback loops or other signalling mechanisms must facilitate tumour growth. We have previously shown that tumour‐derived extracellular vesicles (EVs) can facilitate tumour growth by activating stromal cells in the prostate microenvironment. However, little is known about how mTORC1‐active cells interact with their microenvironment, and the pathogenic role of EVs from these cells is unclear. Therefore, we investigated the characteristics and tumour‐promoting capacity of EVs secreted from mTORC1‐active tumour cells.

**Methods**: We explored characteristics, cargo, and functionality of EVs from TSC2‐expressing (control) and TSC2‐deficient/mTORC1‐active (disease) cells. EVs were isolated by ultracentrifugation‐based floatation within 30% sucrose, and characterised according to MISEV guidelines. We conducted a comprehensive screen of mRNA and protein cargo, with selected targets validated by qPCR and ELISA. EV‐mediated signalling activation in stromal fibroblasts was assessed and functional assays were used to determine EV contribution to disease phenotypes.

**Results**: We show increased EV secretion from mTORC1‐active cells compared to control cells. EVs from mTORC1‐active cells have distinct transcriptomic and proteomic profiles, enriched for cancer‐ and mTORC1 signalling‐associated markers. We show that such EVs can mediate tumour‐promoting signalling downstream of mTORC1 in recipient fibroblasts.

**Summary/Conclusion**: EVs from mTORC1‐active cells have altered characteristics and cargo, enabling these EVs to mediate signalling in the microenvironment to promote tumour growth. This furthers our understanding of mTORC1‐driven tumour development and reveals candidate biomarkers.

### OD04.03 Exploring the role of extracellular vesicles in the cross‐talk between adipocytes and prostate cancer cells

Fabrizio Fontana, University of Milan

Monica Marzagalli, University of Milan

Michela Raimondi, University of Milan

Emanuela Carollo, Department of Biological and Medical Sciences, Oxford Brookes University

Patrizia Sartori, University of Milan

Patrizia Procacci, University of Milan

David R F. Carter, Department of Biological and Medical Sciences, Oxford Brookes University

Patrizia Limonta, University of Milan

**Introduction**: It is known that an association exists between obesity and risk of prostate cancer (PCa). A crosstalk between adipocytes and PCa has been demonstrated; however, the study of this dialog has been limited to metabolites and adipokines, despite emerging evidence points to a key role of extracellular vesicles (EVs) in the control of tumor progression.

**Methods**: After isolation by SEC, EVs were characterized by NTA, TEM and Western blot analysis (TSG101, Hsc70, Alix, calnexin and cytochrome c). In PC3 and DU145 PCa cells, the effect of 3T3‐L1 adipocyte conditioned media and EVs on cell proliferation was evaluated by Trypan blue exclusion assay; AKT phosphorylation was analyzed by Western Blot. After adipocyte media/EV conditioning, PC3 and DU145 metastatic potential was assessed by scratch test and Boyden chamber assay, and matrix metalloproteinase and epithelial‐to‐mesenchymal transition marker levels were analyzed by Western Blot, while changes in mitochondrial activity, ATP synthesis, lipid content and glucose consumption were assessed by flowcytometry. The effect of adipocyte conditioned media/EVs on PCa cell resistance to docetaxel was evaluated by Trypan blue exclusion assay and annexin V/PI assay; Western Blot analysis of caspase 3 and PARP levels as well as of CD44 expression was also performed.

**Results**: We demonstrated that 3T3‐L1 adipocyte conditioned media can affect PC3 and DU145 cell features, inducing increased proliferation, associated with AKT phosphorylation, and invasion, correlated with MMP2 and 9 activation, E/N‐cadherin switch and Snail upregulation. Moreover, PCa cells were found to accumulate lipid droplets and, more importantly, to undergo a neuroendocrine differentiation, accompanied by CD44 enhanced expression and docetaxel resistance. Notably, these results were confirmed in 3T3‐L1 EV‐treated PC3 and DU145 cells, where an increase in the glucose consumption, mitochondrial activity, ATP production and ROS generation was also observed, suggesting that adipocyte EVs can reprogram PCa metabolism.

**Summary/Conclusion**: These results highlight that an EV‐mediated crosstalk exists between adipocytes and PCa, driving tumor aggressiveness. Further studies will be performed to identify the adipocyte EV molecular cargo responsible for the modulation of this dialog.

### OD04.04 Intra‐tumoral microniche critically regulates extracellular vesicle release in lung adenocarcinoma

Gyöngyvér Orsolya Sándor, Semmelweis University, Department of Genetics, Cell and Immunobiology

András Áron Á Soós, Semmelweis University, Department of Genetics, Cell‐ and Immunobiology

Judit Moldvay, Department of Tumor Biology, National Korányi Institute of Pulmonology, Semmelweis University

Zoltán Wiener, Semmelweis University, Department of Genetics, Cell and Immunobiology, Budapest, Hungary

**Introduction**: Lung adenocarcinoma (LUAD), one subtype of lung cancer, is a frequent disease with a poor survival. Wnt production by some tumor cells establishes a special microenvironment for highly proliferating LUAD cells in mouse models and this micro‐niche may critically influence the clinical outcome. The 3D organoid technology maintains the heterogeneity of in vivo epithelial tissues and has proved to be so far the best ex vivo model of human cancers. In our studies we set out i) to prove the presence of this micro‐niche in human LUAD and ii) to study its importance in EV secretion.

**Methods**: We used organoids of different origin. The Medical Research Council of Hungary approved the experiments and informed consent was obtained from patients. EVs were detected by antibody‐coated beads, NTA and TEM. Intra‐tumor heterogeneity was studied by RT‐qPCR and immunostaining.

**Results**: We found that only a subpopulation of mouse lung, normal lung bronchiolar (NL) and LUAD organoid cells produced active Wnt with a partial overlap with proliferating cells, thus, providing evidence for the presence of a Wnt producing microniche both in the normal lung and in human LUAD. Inhibiting Wnt secretion in NL or LUAD organoids critically changed not only cell proliferation, but EV secretion as well. In addition, fibroblast‐derived EVs contributed to the establishment of this intra‐tumor microniche via EVs.

**Summary/Conclusion**: We show here the presence of lung cancer cell subpopulations with different EV release. Thus, our findings may be of high importance when considering EVs as diagnostic tools.

### OD04.05 Extracellular vesicles from retinal pigment epithelial cells expressing misfolded proteins induce epithelial‐mesenchymal transition in recipient cells

Mi Zhou, Department of Ophthalmology, Penn State Hershey College of Medicine, Hershey, PA, USA.

Yuanjun Zhao, Department of Ophthalmology, Penn State Hershey College of Medicine, Hershey, PA, USA.

Sarah Weber, Department of Ophthalmology, Penn State Hershey College of Medicine, Hershey, PA, USA.

Han Chen, TEM Core Facility, Penn State College of Medicine, Hershey, PA, USA.

Alistair Barber, Department of Ophthalmology, Penn State Hershey College of Medicine, Hershey, PA, USA.

Stephanie Grillo, Department of Ophthalmology, Penn State Hershey College of Medicine, Hershey, PA, USA.

Jeffrey Sundstrom, Department of Ophthalmology, Penn State Hershey College of Medicine, Hershey, PA, USA.

**Introduction**: Previous studies in our lab found that the expression of the misfolded protein, R345W‐Fibulin‐3, induces retinal pigment epithelial (RPE) cells to undergo epithelial‐mesenchymal transition (EMT). The purpose of the current study was to investigate the size, cargo and function of extracellular vesicles (EVs) derived from RPE cells expressing the R345W‐Fibulin‐3 mutation, and to determine the role of these EVs in regulating RPE cell dysfunction.

**Methods**: Transmission electron microscopy (TEM) and cryogenic electron microscopy (cryo‐EM) were performed to study EV morphology. The amount and size distribution of EVs were determined by Nanoparticle Tracking Analysis (NTA). EV protein concentrations were quantified using the DCTM Protein Assay (Bio‐Rad). EV cargo were analyzed by unbiased proteomics using LC‐MS/MS with subsequent pathway analysis (Advaita). The EV‐associated transforming growth factor beta 1 (TGF‐β1) protein was measured by enzyme‐linked immunosorbent assay (ELISA). The migration ability of ARPE‐19 cells, in the absence and presence of mutant EVs, was evaluated by using scratch assays.

**Results**: TEM imaging revealed concave‐appearing vesicles, while cryo‐EM imaging showed spherical vesicles with two subpopulations of EVs: a small group with diameters around 30nm and a large group with diameters around 100nm. Imaging also revealed a greater number of small EVs (∼30 nm) in the mutant group compared to the WT group. This result was further confirmed by NTA showing that, in the mutant group, the particle size distributions were smaller than those of the WT EVs. The protein concentration per EV in the mutant group was not significantly different from that of the WT group. Proteomics identified critical members of sonic hedgehog (SHH) signaling and ciliary tip components in the EVs derived from WT ARPE‐19 cells, whereas EVs derived from mutant ARPE19 cells contained EMT mediators, including TGF‐β‐induced protein (TGFBI), vimentin, and mothers against decapentaplegic homolog 4 (SMAD4). ELISA confirmed the elevated TGF‐β1 associated with mutant EVs compared to WT EVs. Critically, EV transplant studies showed that treatment of recipient cells with EVs derived from mutant cells was sufficient to increase migration and elevate EMT markers in RPE cells after scratch‐injury.

**Summary/Conclusion**: The protein cargo of EVs is determined by the phenotype of their parental cells. Expression of R345W‐Fibulin‐3 mutation also alters the size and autocrine function of EVs. Notably, EVs derived from RPE cells expressing R345W‐Fibulin‐3 are sufficient to induce EMT in wild‐type RPE cells.

### OD04.06 Tumors regulate their nutrient demand in response to hypoxia via autocrine EV signaling

Marijke I. Zonneveld, Postdoctoral researcher, GROW‐School for Oncology and Developmental Biology, Maastricht University, The Netherlands

Joël E.J. Beaumont, Department of Radiotherapy, GROW‐School for Oncology and Developmental Biology, Maastricht University

Tom G.H. Keulers, Department of Radiotherapy

Kim Savelkouls, Department of Radiotherapy, GROW‐School for Oncology and Developmental Biology, Maastricht University

Kasper W. Derks, Clinical genetics, Maastricht University medical center, The Netherlands

Marca H.M. H.M. Wauben, Department of Biomolecular Health Sciences, Utrecht University, The Netherlands

Kasper M.A. Rouschop, Department of Radiotherapy, GROW‐School for Oncology and Developmental Biology, Maastricht University

**Introduction**: Hypoxia is an important component of the tumor microenvironment (TME), associated with increased angiogenesis, migration and treatment resistance. In a process known as phenocopying, extracellular vesicles (EV) have been shown to transfer specific traits, such metastatic potential and drug resistance, from one cell to another. We were interested whether EV could phenocopy hypoxia‐tolerance between tumor cells.

**Methods**: HT‐29 and MDA‐MD‐231 cells were exposed to moderate (0.2% O2) or severe (0.02% O2) hypoxia. EV were isolated using a density gradient or size‐exclusion chromatography and characterized by high resolution flow cytometry (HR‐FC) and western blot. Autocrine effects of EV on hypoxia‐naïve tumor cells were assessed via next generation sequencing, qPCR, and glucose/lactate uptake assays. The effects of EV pre‐conditioning on cell viability was assessed through clonogenic survival assay.

**Results**: Hypoxia did not alter the total number of EV secreted, nor were gross protein and RNA content affected. However, HR‐FC showed different scatter patterns and immunoblotting showed distinct patterns for CD9, CD63 and Flotillin‐1, indicative of different subpopulations being secreted. Ingenuitiy pathway analysis of target cell transcriptomes indicated that their metabolism changed in response to EV. qPCR showed a slight increase in GLUT1 (glucose transporter) mRNA levels in target cells exposed to EV from normoxic and moderately hypoxic cells, but not from severely hypoxic cells. To assess whether this was functionally significant, glucose uptake and lactate secretion was assessed. Indeed, EV from normoxic and hypoxic EV stimulated additional uptake of glucose, while EV from severely hypoxic cells did not. Lactate secretion was elevated in all EV‐exposed cells compared to controls. The overall survival of these cells was not significantly impacted.

**Summary/Conclusion**: Hypoxia alters the composition of EV, leading to adaptations in GLUT1 transcription and glucose uptake in target cells. These results show that the tumor regulates nutrient demand in response to hypoxia via EV in an autocrine fashion.

### OD04.07 Carcinoma‐associated fibroblasts secrete small extracellular vesicles carrying Periostin and LOX to promote collagen cross‐linking

Xue Liu, Department of Basic Science of Stomatology, Shanghai Stomatological Hospital, Fudan University, Shanghai, China

Tingjiao Liu, Department of Basic Science of Stomatology, Shanghai Stomatological Hospital, Fudan University, Shanghai, China

**Introduction**: Extracellular matrix (ECM) stiffening is an important feature of tumor stroma and is related to tumor invasion, metastasis, drug resistance and prognosis. Recently, small extracellular vesicles (sEVs) play an important role in mediating cell communication. However, the interaction between EVs and ECM is rarely reported. It attracts researchers’ attention whether EVs produced by carcinoma‐associated fibroblast (CAF) can mediate cell‐ECM communication. In this study, we investigated the role of the CAF sEVs promoting collagen cross‐linking.

**Methods**: sEVs were isolated from the conditioned medium of four CAFs and NF by differential ultracentrifugation. CAF sEVs was added to NF for 12 hours, ELISA determined the contents of Pyridinoline, dihydroxylysinonorleucine (DHLNL) and hydroxylysinonorleucine (HLNL) to evaluate collagen crosslinking in vitro.

**Results**: Transmission electron microscopy (TEM) photos showed CAF sEVs were typical cup‐shaped contains lipid bilayer membrane. Western blot (WB) showed that sEVs expressed typical exosome markers, such as CD63, CD9 and Hsp70. Nanoparticle tracking analysis (NTA) results showed that the average particle size distribution of CAF sEVs mostly diameter in 150 nm. Periostin (POSTN) expression in CAFs was significantly higher than NF, and OSCC cell lines were not expressed. Similar to the cellular results, POSTN was expressed in CAF sEVs, but not in NF and OSCC cell lines derived sEVs. POSTN was located on the surface of CAF sEVs. POSTN content increased with the increase of CAF sEVs concentration, and POSTN content decreased significantly after CAF sEVs was added with POSTN blocking antibody. In addition, POSTN combines with BMP1 and αLOX in CAF sEVs. POSTN and αLOX were co‐located on the surface of CAF sEVs membrane. Content of LOX in CAF sEVs increased with the concentration of CAF sEVs, and the content of LOX in CAF sEVs decreased significantly after blocking with LOX antibody. ELISA showed that the contents of Pyridinoline, DHLNL and HLNL in CAF sEVs was significantly higher than CAF sEVs + LOX Antibody and CAF sEVs + BAPN.

**Summary/Conclusion**: Our data indicate that POSTN may be a biomarker that distinguishes CAF sEVs from OSCC cell‐derived sEVs, POSTN is located on the membrane surface of CAF sEVs. CAF sEVs directly promote collagen crosslinking in vitro carrying POSTN‐BMP1‐αLOX with bioactive LOX.

### OD04.08 The role of the autophagy adaptor protein PLEKHM1 and exosome release in the leukaemic bone marrow microenvironment

Christina Karantanou, Georg‐Speyer‐Haus, Institute for Tumor Biology and Experimental Therapy

Valentina R. Minciacchi, Georg‐Speyer‐Haus, Institute for Tumor Biology and Experimental Therapy

Rahul Kumar, Georg‐Speyer‐Haus, Institute for Tumor Biology and Experimental Therapy

Costanza Zanetti, Georg‐Speyer‐Haus, Institute for Tumor Biology and Experimental Therapy

Georg Tascher, Institute of Biochemistry II, Medical Faculty, Goethe‐University

Christian Münch, Institute of Biochemistry II, Medical Faculty, Goethe‐University

Tobias Tertel, Institute for Transfusion Medicine, University Hospital Essen, Germany

Bernd Giebel, Prof, Institute for Transfusion Medicine, University Hospital Essen, Germany

David G. McEwan, Cancer Research UK Beatson Institute

Ivan Dikic,Institute of Biochemistry II, Medical Faculty, Goethe‐University

Daniela S. Krause, Georg‐Speyer‐Haus, Institute for Tumor Biology and Experimental Therapy

**Introduction**: Bone marrow microenvironment (BMM)‐derived mesenchymal stromal cells (MSC) play a role in the maintenance of normal haematopoiesis and leukaemia progression. Pleckstrin homology domain‐containing family M member 1 (PLEKHM1) serves as a hub for the fusion of intracellular vesicles and their secretion. We hypothesized that PLEKHM1 may have a role in the regulation of extracellular vesicle (EV) biogenesis and that EV secreted by BMM‐derived MSC may modulate leukaemia progression.

**Methods**: Retroviral transduction/transplantation mouse model of B‐cell acute lymphoblastic leukaemia (B‐ALL); flow cytometry; imaging; differential centrifugation; AMNIS analysis; proteomic and Western blot (WB) analysis.

**Results**: Induction of B‐ALL in WT or Plekhm1 KO primary recipient or in WT secondary recipient mice revealed an increase of B‐ALL aggressiveness and B‐ALL‐initiating cell number and/or function, respectively. Small EV (sEV) from Plekhm1 KO MSC did not differ in number, but contained increased levels of syndecan‐binding protein 1 (syntenin) and its binding partner, syndecan‐1, compared to WT. Treatment of B‐ALL cells with KO sEV led to increased levels of syntenin and syndecan‐1 and higher pAkt, a sign of pro‐survival and growth in target cells. This effect was reversed upon inhibition of EV uptake. Furthermore, pretreatment of leukaemia cells with KO sEV increased their aggressiveness in vivo. TNFα secretion by leukaemia cells may lead to PLEKHM1 downregulation with simultaneous upregulation of syntenin and syndecan‐1 in MSC.

**Summary/Conclusion**: Our data suggest that reduced PLEKHM1 levels in MSC may affect the cargo of EV. Uptake of these EV by leukaemia cells may increase their aggressiveness, thereby, leading to the establishment of a novel mechanism for the delivery of pro‐survival signals to leukaemia cells, triggered by the release of TNFα from leukaemia cells.

## OD05 EV Separation and Concentration

Chair: Antonio Marcilla, Area de Parasitologia, Departamento de Farmacia y Tecnología Farmacéutica y Parasitología, Universitat de València, Burjassot Valencia, Spain; Joint Research Unit on Endocrinology, Nutrition and Clinical Dietetics, Health Research Institute La Fe, Universitat de Valéncia,Valencia, Spain

Chair: Rienk Nieuwland, Department of Clinical Chemistry, Amsterdam UMC, University of Amsterdam, Amsterdam, the Netherlands, Vesicle Observation Center, Amsterdam UMC, University of Amsterdam, Amsterdam, the Netherlands

### OD05.01 Optimization of the pre‐treatment and evaluation of different separation techniques to isolate EV subpopulations from a complex biofluid like synovial fluid

Daniele D ' Arrigo, Regenerative Medicine Technologies Lab, Ospedale Regionale di Lugano

Chiara Arrigoni, Regenerative Medicine Technologies Lab, Ospedale Regionale di Lugano, Lugano (CH)

Marco Vanoni, Department of Biotechnology and Bioscience, Università degli studi di Milano‐Bicocca, Piazza della Scienza 2, Milan (IT)

Christian Candrian, Regenerative Medicine Technologies Lab, Ospedale Regionale di Lugano, Lugano (CH)

Matteo Moretti, Regenerative Medicine Technologies Lab, Ospedale Regionale di Lugano, Lugano (CH)

**Introduction**: Extracellular vesicles (EVs) isolated with liquid biopsies from biofluids emerged as a promising diagnostic tool for several diseases, including the osteoarthritis. Most of the studies were focused on small size EVs, but evidences demonstrated that also larger EVs have a pathophysiological role. However, the development of effective isolation and separation methods to obtain different EV subpopulations is not straightforward. This is made even more difficult due to the complexity of biofluids like the synovial fluid (SF). Aiming at developing a protocol to isolate and separate EV subpopulations with different size from SF, we optimized its pre‐treatment and compared different separation protocols.

**Methods**: We collected SF from the knee joints of 5 end‐stage arthritic patients underwent joint replacement. To optimize the pretreatment, we evaluated the effect of hyaluronidase digestion. Then, to remove from the SF the cell contaminants without affecting the yield of the EVs, we evaluated the effect of different centrifugation regimens on the SF samples. Finally, we tested and compared the effectiveness of different methods, such as ultracentrifugation, size‐exclusion chromatography also with HPLC and others, to isolate and separate different‐sized EV subpopulations.

**Results**: The hyaluronidase digestion decreased SF viscosity, increasing the total number of EVs isolated with ultracentrifugation by about 40%. We also found the best centrifugation regimen to remove the highest quantity of cell contaminants while minimizing the loss of larger EVs. On the other hand, the isolation techniques differed in the capability to separate the different‐sized EV subpopulations. Finally, we characterized the EVs belonging to each subpopulation by different methods among which light scattering, western blot (e.g. CD63, TSG101, Albumin, and others) and protein content quantification.

**Summary/Conclusion**: We optimized the pre‐treatment of the SF to facilitate the EV isolation process. We also demonstrated that various separation methods have a different capability in isolating EV subpopulations with different size. This could be useful for future studies aiming to isolate different‐sized EV subpopulations from complex biofluids like SF.

### OD05.02 DNA‐directed immobilization of antibodies and its application in extracellular vesicles purification and fabrication of EV‐based diagnostics

Dario Brambilla, CNR ‐ SCITEC

Laura Sola, National Research Council of Italy ‐ Institute of Chemical Sciences and Technologies (CNR ‐ SCITEC)

Marcella Chiari, National Research Council of Italy ‐ Institute of Chemical Sciences and Technologies (CNR ‐ SCITEC)

**Introduction**: Extracellular vesicles (EVs) are a powerful source of novel biomarkers in the diagnosis and prognosis of disease. In spite of their potential in human diagnostics, the choice of the most appropriate purification and characterization strategies is often troublesome. Immunoaffinity approaches offer and unmatched selectivity allowing the analysis of specific subpopulation of EVs. Unfortunately strategies involving antibodies suffer from disadvantages that limit their application. We present DNA‐directed immobilization (DDI) of antibodies to overcome some of the limitations. The use of DDI in immunoaffinity separation enhances the antibody affinity towards the target. We have exploited DDI in the immunoaffinity separation of EVs to enable the recovery of intact vesicles. In the proposed approach EVs are immunocaptured on magnetic beads and finally released using enzymatic cleavage of DNA linker mediated by DNase I.

**Methods**: Antibodies were tagged with different sequences of ssDNA exploting strain promoted azide‐alkyne cycloaddition. DNA‐antibody conjugates were used to generate antibody mciroarrays via DDI. The microarrays were used to characterize EVs derived by HEK‐293 cell culture media. Magnetic beads, coated with streptavidin, were also functionlized with DNA‐directed antibodies, incubated with EVs for 2.5 h at room temperature to capture EVs, and then treated with DNAse I for 1 h at 37°C. The released EVs were analyzed by Nanoparticle Tracking Analysis, nanoFlow Cytometry and TEM microscopy.

**Results**: Antibodies immobilized via DDI showed a greater affinity in comparison with antibodies conventionally immobilized on sensors, and this feature was exploited to develop an analytical platform and an immunoaffinity separation kit. The microarray sensor showed consistency with commercial kits, while DDI‐based immunoaffinity experiments allowed the separation of intact EVs, as confirmed by NTA, nanoFCM and TEM analysis.

**Summary/Conclusion**: We present an unprecedented application of DNA‐directed immobilization of antibodies for EVs separation and characterization. DDI‐based tools potentially pave the way towards the implementation of EV analysis in diagnostics.

### OD05.03 Assessing the biomolecular landscape and dynamics of systemically circulating lipid‐carrying particles

Cláudio A. Pinheiro, Laboratory of Experimental Cancer Research, Department of Human Structure and Repair, Ghent University, Cancer Research Institute Ghent, Ghent, Belgium

Glenn Vergauwen, Department of Gynecology, Ghent University Hospital, Ghent, Belgium

Joeri Tulkens, Laboratory of Experimental Cancer Research, Department of Human Structure and Repair, Ghent University, Ghent, Belgium

Cláudio A. Pinheiro, Laboratory of Experimental Cancer Research, Department of Human Structure and Repair, Ghent University, Cancer Research Institute Ghent, Ghent, Belgium

Francisco Avila Cobos, Cancer Research Institute Ghent, OncoRNALab, Department of Biomolecular Medicine, Ghent University, Ghent, Belgium

Sándor Dedeyne, Laboratory of Experimental Cancer Research, Department of Human Structure and Repair, Ghent University, Cancer Research Institute Ghent, Ghent, Belgium

Marie‐Angélique De Scheerder, Department of General Internal Medicine, Ghent University Hospital, Ghent, Belgium

Linos Vandekerckhove, Department of General Internal Medicine, Ghent University Hospital, Ghent, Belgium

Francis Impens, VIB Center for Medical Biotechnology, Department of Biomolecular Medicine, VIB Proteomics Core, Ghent, Belgium

Ilkka Miinalainen, Biocenter Oulu, University of Oulu, Oulu, Finland

Geert Braems, Cancer Research Institute Ghent, OncoRNALab, Department of Biomolecular Medicine, Ghent University, Department of Gynecology, Ghent University Hospital, Ghent, Belgium

Kris GevaertVIB Center for Medical Biotechnology, Department of Biomolecular Medicine, Ghent University, Ghent, Belgium

Pieter Mestdagh, Cancer Research Institute Ghent, OncoRNALab, Department of Biomolecular Medicine, Ghent University, Ghent, Belgium

Jo Vandesompele, Cancer Research Institute Ghent, OncoRNALab, Department of Biomolecular Medicine, Ghent University, Ghent, Belgium

Hannelore Denys, Department of Medical Oncology, Ghent University Hospital, Ghent, Belgium

Olivier De Wever, Laboratory of Experimental Cancer Research, Department of Human Structure and Repair, Ghent University, Ghent, Belgium

An Hendrix, Laboratory of Experimental Cancer Research, Department of Human Structure and Repair, Ghent University, Ghent, Belgium

**Introduction**: Separating lipid‐carrying particles from blood is challenging and complicates the biological understanding and biomarker development of extracellular vesicles (EV) and lipoprotein particles (LPP). In this study, we fractionate blood plasma samples (n = 36) with the aim to assess the biomolecular composition and dynamics of EV and LPP in multiple disease conditions.

**Methods**: Size‐exclusion chromatography (SEC) followed by density gradient (DG) centrifugation fractionates blood plasma in two dimensions, namely size and density. EV and LPP extracts are characterized by EM, western blot, ELISA, NTA, LC‐MS/MS and small RNAseq. To validate the repeatability of the protocol, we analyze pooled blood plasma from healthy donors and breast cancer patients. We investigate longitudinal samples from ovarian cancer patients and HIV patients to test the variability of EV cargo composition during treatment.

**Results**: SEC prepares crude extracts from blood plasma retaining 1.4%, 9.67% and 73.0% of APOA1‐containing LPP, APOB‐containing LPP and CD9‐containing EV. DG centrifugation further fractionates crude extracts with high specificity into LPP and EV extracts. LPP extracts (1.04 " 1.07 g/mL) retain 85.2% and 95.4% of respectively APOA1 and APOB‐containing LPP while EV extracts (1.09‐1.10 g/mL) hold 30% of EV. Multi‐omics analysis of technical replicates confirms high methodological repeatability. Differential analysis of the protein composition of EV versus LPP extracts identifies the compositional nature of the EV protein corona and provides a catalog of proteins associated with systemically circulating EV versus LPP. Finally, we reveal that EV carry a unique, dynamic, context‐dependent protein composition, and miRNA and tRNA profile, which are not directly measurable in LPP extracts and total blood plasma, respectively.

**Summary/Conclusion**: The implementation of blood plasma fractionation substantially advances the biological understanding and biomarker development of systemically circulating lipid‐carrying particles.

### OD05.04 Automated liquid handling for highly specific and reproducible density‐based separation of extracellular vesicles from human body fluids

Sofie Van Dorpe, Laboratory of Experimental Cancer Research, Department of Human Structure and Repair, Ghent University, Ghent, Belgium

Lien Lippens, PhD studen, Laboratory of Experimental Cancer Research, Department of Human Structure and Repair, Ghent University, Ghent, Belgium

Robin Boiy, Laboratory of Experimental Cancer Research, Department of Human Structure and Repair, Ghent University, Ghent, Belgium

Hannelore Denys, Department of Medical Oncology, Ghent University Hospital, Ghent, Belgium

Olivier De Wever, Laboratory of Experimental Cancer Research, Department of Human Structure and Repair, Ghent University, Ghent, Belgium

An Hendrix, Laboratory of Experimental Cancer Research, Department of Human Structure and Repair, Ghent University, Ghent, Belgium

**Introduction**: Extracellular vesicles (EV) in body fluids are extensively studied as potential biomarkers for numerous diseases. Major impediments of EV‐based biomarker discovery include the specificity and reproducibility of EV sample preparation. To tackle this, we present an automated liquid handling workstation for the density‐based separation of EV from human body fluids and compare its performance to manual handling by (in)experienced researchers.

**Methods**: Variability in density‐based EV separation using manual versus automated liquid handling is first evaluated by spiking PBS with trackable recombinant extracellular vesicles (rEV) followed by the quantification of rEV recovery efficiency using fluorescent NTA and ELISA (Geeurickx et al., Nature Comm, 2019). Next, EV are separated from blood plasma and urine through the orthogonal implementation of size‐exclusion chromatography and manual or automated density‐gradient centrifugation as previously described (Dhondt et al., STAR Protoc, 2020; Tulkens et al., Nature Protoc, 2020). Variability, EV yield and purity are assessed by MS‐based proteomics and transmission electron microscopy.

**Results**: Automated versus manual liquid handling significantly reduces variability in rEV recovery after density‐based separation (CVauto 11.6% vs CVman 26.4%), and significantly decreases variability in EV preparations obtained from blood plasma and urine. Indeed, the median CVauto for protein group quantification are between 17.2% (plasma) and 20.0% (urine), CVman,exp between 38.5% (plasma) and 22.6% (urine), and CVman,inexp between 43.2% (plasma) and 27.6% (urine). While retaining an equal EV yield compared to manual liquid handling, automation significantly diminishes the presence of contaminating abundant proteins in EV preparations, including lipoproteins in plasma (2‐fold decrease in ApoB) and uromodulin in urine (2.6‐fold decrease in THP).

**Summary/Conclusion**: Automated liquid handling ensures EV separation from body fluids with high reproducibility and specificity.

### OD05.05 Capillary‐channeled polymer (C‐CP) fiber solid‐phase extraction micropipette tips for the isolation of extracellular vesicles (EVs) from complex biofluid matrices

Kaylan K. Jackson, Clemson University

Rhonda R. Powell, Clemson University

Terri F. Bruce, MDPhD, Clemson University

R. Kenneth Marcus, FRSC, FAAAS, FSAS, FNAI, Clemson University

**Introduction**: Available extracellular vesicle (EV) isolation methods are time‐consuming, costly, and result in low recoveries and purity. Typically, EVs are contaminated with protein/lipoprotein aggregates. This creates difficulties in the quantification and characterization of recovered EVs, hindering fundamental research and downstream applications. A method capable of producing pure EVs, repeatedly, across diverse size scales is of critical importance. Capillary‐channeled polymer (C‐CP) fiber micropipette tips are employed here in a solid‐phase extraction (SPE) workflow for the efficient isolation of EVs.

**Methods**: Polyester (PET) C‐CP fibers are employed here in a spin‐down micropipette tip format for the SPE of EVs, allowing processing using simple table‐top centrifugation of sample volumes down to 10s of microliters. Demonstration matrices have included urine, saliva, cervical mucus, blood serum and plasma, milk, and cell culture media. EV purity is assessed based on the removal of free lipoproteins and using an ELISA to apolipoprotein B100 and tetraspanin proteins (CD81, CD63). Electron and confocal microscopy confirm the presence of EVs, with concentration and size distributions determined via absorbance (scattering) detection and nanoparticle tracking analysis.

**Results**: Quantitative recoveries of up to 1E10 EVs are readily obtained from 100 μL of biofluids using the C‐CP fiber tip method. TEM analysis confirms the retention of the characteristic EV cup‐shape following isolation. The HIC tip isolation workflow removes up to 89% of biofluid‐originating proteins. A >60% reduction of spiked lipoproteins is demonstrated by ELISA detection of the ApoB‐100 LDL‐marker in the EV fractions, with MS proteomics suggesting even higher purities.

**Summary/Conclusion**: The C‐CP fiber tip EV isolation technique is beneficial in terms of time (< 15 min), cost (< $1 per tip), ease of use, and clinical tailor‐ability in terms of capture and labeling. The C‐CP tip workflow produces clean and concentrated EV recoveries. This new isolation method introduces a simple capture mode based on vesicle hydrophobicity, with EV imaging techniques performed directly on the fiber surface. The long‐term goal is to create an efficient, practical EV isolation method for fundamental and clinical applications.

### OD05.06 A defected metal‐organic framework featuring cleavable lipid probe for efficient and non‐destructive isolation of extracellular vesicles

Weilun Pan, Nanfang hostipital

Bo Li, Department of Laboratory Medicine, Nanfang Hospital, Southern Medical University

Feng Jun Jie, Department of Laboratory Medicine, Nanfang Hospital, Southern Medical University

**Introduction**: Extracellular vesicles (EVs) are phospholipid bilayer surrounded particles ranging from 30 nm‐1 μm released by all types of cells in an evolutionally conserved manner. Increasing evidence suggests that EVs have potential as clinical biomarkers and therapeutic agents. However, because EVs with heterogeneous sizes are present in biological fluids such as plasma, serum, urine, saliva, and cell supernatant, in which also non‐EV biomolecules are abundant. Thus, it is urgent to develop an efficient platform for the isolation of high pure EVs.

**Methods**: Herein, we constructed a defected nanoscale Metal‐organic frameworks (MOF) UiO‐66‐NH2 as a supporter, a cleavable lipid probe PO43–Spacer‐DNA‐Cholesterol (PSDC) connected to the MOF as cpaturer to isolate EV. With the strong affinity between PO43‐ on PSDC and Zr(IV) in the MOF, the MOF@PSDC platform was constructed easily. Next, due to the high binding efficacy between cholesterol and phospholipid bilayer of EVs, a system of MOF@PSDC@EVs was developed to separate EVs at 12 800 g within 10 minutes ascribe to the high specific gravity of MOF. After trapping of EVs, the MOF@PSDC@EVs was further incubated with deoxyribonuclease I (DNase I) to hydrolyze DNA, resulting in detachment of EVs from the MOF. With the second centrifugation to remove the MOF, isolated EVs were obtained rapidly and efficiently.

**Results**: The MOF@PSDC was constructed successfully by the confirmation of a series of characterization. Model EVs derived from breast cancer cells MDA‐MB‐231 were applied for identifying of the feasibility of the MOF@PSDC in isolating EVs with high efficiency and purity. This MOF@PSDC platform was further used to isoalted EVs from plasmas samples from patients with breast cancer in different clincal stages, benign lesions and healthy individuals and sujected to the detection of three biomarkers glycipan‐1 (GPC‐1), CD63 and human epidermalgrowth factor receptor‐2 (HER‐2). The resultes indicated that by using the established platform MOF@PDC, EVs from plasmas could be isolated in a quick and high quality maner for downstream analysis.

**Summary/Conclusion**: In summary, a universal EVs isolation platform based on a defected MOF featuring cleavable lipid probe (MOF@PSDC) has been constructed successfully. Such a novel defected MOF platform featuring the cleavable lipid probe shows merits over existing EVs isolation methods in terms of its advantages that include fast, efficient, non‐destructive, and contamination‐free without requirement of the expensive equipment. It could be further studied to apply for harvesting EVs from complex biological fluids to facilitate EVs‐based research in clinical theranostics. Furthermore, this study also provides new insights into the design of functional materials for isolating subcellular structures or biomacromolecules.

## OD06 EVs in Cancer Immunity

Chair: Hang Hubert Yin, Tsinghua University School of Pharmaceutical Sciences, China (People's Republic)

Chair: Susanne Gabrielsson, Karolinska Institutet, Sweden

### OD06.01 NK‐cell derived EVs target and kill resistant cancer cell‐derived spheroids

Miriam Aarsund Larsen, University of Oslo

Marit Inngjerdingen, Oslo University Hospital

**Introduction**: Natural killer (NK) cells are innate lymphocytes that recognize and kill cancer cells. NK cell‐based therapies has shown promise for hematological tumors, but their use for solid cancers is hampered by their poor ability to infiltrate the tumor. EVs secreted from NK cells (NK cell‐derived EVs; NK‐EVs) are shown to contain the cytolytic material necessary for targeting and killing cancer cells, indicating that NK‐EVs may be ideal therapeutic agents.

**Methods**: EVs were isolated from primary NK cells or the NK cell lines NK92 or KHYG‐1 upon culture in either IL‐15 (resting) or a cocktail of IL‐12, IL‐15, and IL‐18 (activation). EVs were precipitated, and successful isolation confirmed by Western blotting, NTA, and electron microscopy. Phenotypic characterization was done by Western blotting and mass spectrometry. Function were tested in cultures and in spheroids from a total of seven different solid cancer cell lines (HCT116 and HCT 15 (colon), DU145 (prostate), OVCAR (ovarian), SKRB3 and T4D7 (breast), WM9 (melanoma)). Tumor growth and kinetics of apoptosis upon treatment with NK‐EV was obtained by continuous monitoring for 7 days using InCuCyte technology. Evaluation of EV penetration into spheroids was evaluated by histology.

**Results**: Apoptosis and tumor shrinkage was observed upon treatment with primary NK cell‐ or NK92‐derived EVs in colon, melanoma, prostate and ovarian spheroids, but not breast cancer‐derived. We found that activating NK cells with IL‐12, IL‐15, and IL‐18 generated EVs with enhanced killing capacity, although they produced similar amounts of EVs as resting cells. The enhanced capacity of IL‐12/15/18‐activated NK cell‐derived EVs was linked to enhanced protein levels of cytolytic proteins. We further observed differential expression of ligands for the activating receptors NKG2D by the cancer cell lines, that matched their susceptibility to NK‐EVs. The killing activity was conversely abrogated using an anti‐NKG2D antibody. Finally, we found that NK‐EVs were able to penetrate into the spheroid core.

**Summary/Conclusion**: We have in this study systematically analyzed NK‐EV targeting of cancer cells derived from solid tumors, and demonstrated potent killing activity of spheroids.

### OD06.02 Separating tumor EV subtypes by Asymmetric Flow field flow fractionation (AF4) unravels differential functional capacities

Federico Cocozza, Institut Curie / INSERM U932

Lien Lippens, PhD studen, Laboratory of Experimental Cancer Research, Department of Human Structure and Repair, Ghent University, Ghent, Belgium

Mabel Jouve, Institut Curie / CNRS UMR 3215

Aurélie Di Cicco, Institut Curie/UMR CNRS168

Bruno G. De Geest, Department of Pharmaceutics, Ghent University, Ottergemsesteenweg 460, 9000, Ghent, Belgium

Daniel Levy, Institut Curie/UMR CNRS168

An Hendrix, Laboratory of Experimental Cancer Research, Department of Human Structure and Repair, Ghent University, Ghent, Belgium

Clotilde Thery, MD PhD, Institut Curie / INSERM U932

Mercedes Tkach, Institut Curie / INSERM U932

**Introduction**: Extracellular vesicles (EVs) represent a heterogenous population of vesicles of different sizes, densities and compositions, whose common and distinct functional properties are still ill defined. The asymmetric flow field‐flow fractionation (AF4) technology has recently been shown to efficiently separate EV subtypes based on their size, and the use of this technology led to the identification of a distinct very small nanoparticle, named exomere (Zhang et al., 2018, Nat Cell Biol 20: 332). The goal of our study was to establish the AF4 technology to separate tumor‐derived EV subtypes, and compare their efficiency to be captured by target phagocytic immune cells (dendritic cells).

**Methods**: Mouse mammary tumor E0771 cells were transfected with a general membrane‐binding tag (myr/palm) fused to mCherry to target the fluorescent protein towards lipid membranes and therefore a broad set of secreted EVs.

EVs obtained by differential ultracentrifugation (dUC) and AF4 fractionation were characterized by Cryo Electron Microscopy and Western Blot. Flow cytometry and immuno‐fluorescence microscopy were used to assess the uptake of mCherry by mouse dendritic cells.

**Results**: A protocol with a good resolution to separate EVs from 40nm to 180nm from the myr/palm ‐mCherry expressing cells was set. Western blot and electron microscopy confirmed the presence of exomeres and at least two types of EVs including one with viral particle features. mCherry was present together with other EV markers, in all the types of particles. Exomere markers recovery was sensitive to an ultracentrifugation washing step.

Our results show that all types of particles can be uptaken but with different efficacies.

**Summary/Conclusion**: The size distributions of EVs secreted by E0771 cells show the existence of two populations of sEVs (below and above 100nm in diameter) plus exomeres (smaller than 50nm), which can all be uptaken by dendritic cells. The functional consequences of this uptake (i.e. degradation or delivery to cytosol) must now be evaluated.

### OD06.03 The αvβ6 Integrin Regulates IFIT3 Protein Levels in PrCa Cells and their EVs

Nicole M. Naranjo, Thomas Jefferson University

Israa Salem, Thomas Jefferson University

Shiv R. Krishn, Thomas Jefferson University

Larry Harshyne, Thomas Jefferson University

D. Craig Hooper, Thomas Jefferson University

Lucia R. R. Languino, Thomas Jefferson University

**Introduction**: The αvβ6 integrin (αvβ6), a transmembrane cell adhesion receptor, is a known biomarker of distinct cancers, including prostate cancer (PrCa). We have shown that transfer of αvβ6 via sEVs to recipient cells, including monocytes, affects their phenotype and function. Therefore, we aimed to evaluate if αvβ6 regulates the expression of specific downstream effectors of the adaptive immune response, specifically the Interferon Induced Proteins with Tetratricopeptide Repeats family (IFIT) in PrCa and their EVs.

**Methods**: To investigate αvβ6 effect on the IFIT protein expression in cells and/or EVs, we have isolated LEVs and sEVs derived from PrCa cells treated with siRNA to downregulate αvβ6 expression. We have characterized LEVs and sEVs by using immunoblotting analysis as well as assessed their size and concentration via Nanoparticle tracking analysis (NTA). Moreover, to isolate and characterize LEVs and sEVs devoid of αvβ6 or IFIT3, we have generated homozygous αvβ6 or IFIT3 knock out PC3 PrCa cells using CRISPR‐Cas9 strategies.

**Results**: Our analysis shows that among other downstream effectors, the IFIT3 protein, which is an antiviral protein induced by type I and II interferon pathways, is upregulated in both LEVs and sEVs derived from αvβ6 siRNA treated PrCa cells. Furthermore, IFIT3 protein expression is increased in LEVs and sEVs derived from CRISPR‐αvβ6 deleted PrCa cells compared to PrCa cells containing αvβ6. NTA analysis shows that the presence or absence of αvβ6 does not influence the size or concentration of LEVs and sEVs released by PrCa cells. Moreover, αvβ6 protein levels are not affected in LEVs or sEVs derived from CRISPR‐IFIT3 deleted PrCa cells.

**Summary/Conclusion**: Overall, these results show that the IFIT3 protein expression in PrCa and their EVs is regulated by the αvβ6 integrin. Furthermore, they suggest that transfer of PrCa EVs, enriched in IFIT3 and devoid of αvβ6, may affect recipient cell phenotype and functions.

### OD06.04 Release of Immunosuppressive Tumor‐Derived Extracellular Vesicles Impairs with CD8+‐T cell Activity in Response to Radiotherapy

Yohan Kim, Mayo Clinic

Roxane R Lavoie, Mayo Clinic

Haidong Dong, Mayo Clinic

Sean Park, Mayo Clinic

Fabrice Lucien, MD PhD, Mayo Clinic

**Introduction**: Stereotactic ablative radiotherapy (SABR) is the effective treatment for prostate cancer (PCa) patients with few metastatic lesions (oligometastasis). SABR induces immunogenic cell deaths and elicits a systemic antitumor immune response at non‐irradiated distant metastatic sites (abscopal effect). Despite clinical observations of abscopal effect, cases remain scarce as most patients develop wide‐spread metastasis. There is an unmet need to elucidate the underlying mechanisms that impair with SABR‐mediated abscopal effect and develop more effective combination therapies. Extracellular vesicles (EVs) have emerged as key players of antitumor immunity locally and systemically. However, their functional roles in radiotherapy‐associated antitumor immune response have not been fully investigated yet.

**Methods**: To examine the cross‐talk between PCa‐derived EVs (PCEVs) and CD8+‐T cell in response to radiotherapy, nanoscale flow cytometry was used to analyze plasma samples from oligometastatic castration‐resistant prostate cancer (omCRPC) patients treated with SABR. Co‐culture of human CD8+‐T cells with PCEVs was used to examine the molecular and cellular mechanisms involved in EV‐mediated CD8+‐T cell activity. Finally, integrated transcriptomic and proteomic profiling was employed to identify molecular determinants responsible for the immunomodulatory activity of PCEVs.

**Results**: By using clinical samples, we have observed that SABR induces release of PCEVs into bloodstream and high PCEV levels were associated with higher risk of recurrence. High levels of PCEVs negatively correlated with levels of effector memory CD8+‐T cells. We also observed that EVs released from irradiated PC3 and DU145 cells inhibit CD8+‐T cell proliferation and cytotoxic function in vitro. Profiling of immunomodulatory cell‐surface proteins on PCa cells identified PD‐L1 and B7‐H3 as key players of EV‐mediated immunosuppression in response to radiotherapy. Blockade of PD‐L1 and B7‐H3+ EV release led to improved CD8+‐T cell function.

**Summary/Conclusion**: This study reveals a novel cellular mechanism that compromises radiation‐mediated antitumor immune response through the release immunosuppressive tumor‐derived EVs. Targeting these EVs in combination with SABR may represent a promising therapeutic approach for the treatment of omCRPC patients.

### OD06.05 Identification of miRNAs released in vivo by extracellular vesicles upon BRAFV600E induction in thyrocytes; implication for immune cell recruitment

Ophélie Delcorte, DDUV institute‐ Cell Unit

Catherine Spourquet, DDUV Institute‐CELL Unit

Pascale Lemoine, DDUV Institute‐CELL Unit

Christophe E. Pierreux, DDUV Institute‐CELL Unit

**Introduction**: Papillary thyroid carcinoma (PTC) carrying BRAFV600E mutation is the most frequent subtype of thyroid cancers. Despite a very good prognosis in most cases, postsurgery recurrences and metastases occur in 10–15% of patients. Moreover, differential diagnosis between benign and malignant nodules is still challenging. Knowledge about extracellular vesicles (EVs) in PTC is rather weak when it could rise a double interest: a better understanding of the biology of PTC and its clinical behavior, and an improvement of differential diagnosis between thyroid cancer types.

The goal of this project is to (i) identify miRNAs actors and markers, released via EVs by the tumor, and (ii) decipher the mechanisms by which they impact on thyroid cancer and its microenvironment.

**Methods**: Using a mouse model in which BRAFV600E is selectively expressed in thyrocytes upon doxycycline injections, we developed a protocol to isolate control and PTC‐EVs from dissociated tissue by differential ultracentrifugations. Vesicles in the high‐speed pellet displayed a size and some specific markers that were consistent with exosomal characteristics. Sequencing was performed to identify miRNAs with a differential abundance in EVs isolated from control and BRAFV600E PTC tissue. In silico and in vitro analyses were carried out to elucidate their role within tumor microenvironment.

**Results**: We identified miRNAs that were progessively deregulated during PTC development in tissue and EVs. To study the role of those EV‐miRNAs, we focused on upregulated candidates, as those were the first deregulated and the most susceptible to be involved in intercellular communications within the tumour microenvironment. The in silico analysis of upregulated EV‐miRNAs and downregulated tissue‐mRNAs identified by mRNA sequencing was performed. It revealed a network organized around 6 miRNAs with many common mRNA targets, that were enriched in immune‐related pathways.

**Summary/Conclusion**: We provide a gradual tissue‐ and EV‐miRNAs profiling along BRAFV600E‐driven PTC development and thyrocyte dedifferentiation, and identify miRNAs candidates which could have an impact on tumorigenesis and communication within the tumor microenvironment, especially on the immune microenvironment.

### OD06.06 Tumor secreted extracellular vesicles regulate T‐cell costimulation and can be engineered to induce tumor specific T‐cell responses

Subbaya Subramanian, Department of Surgery, University of Minnesota

Ce Yuan, Department of Surgery, University of Minnesota

Dechen Wangmo, Department of Surgery, University of Minnesota

Xianda Zhao, Department of Surgery, University of Minnesota

Xianda Zhao, Department of Surgery, University of Minnesota

**Introduction**: Colorectal Cancer (CRC) is a major cause of cancer‐related deaths world‐wide. Immune checkpoint blockade therapy (ICBT) is effective in 30–60% of the microsatellite instable‐High (MSI‐H) subtype. Unfortunately, most CRC patients (>85%) have microsatellite stable (MSS) tumors, that do not respond to ICBT. In this study, we aim to decipher the underlying tumor intrinsic mechanisms critical for improving immunotherapy in colorectal cancer.

**Methods**: We used human and mouse tumor samples, cell lines, and various syngeneic orthotopic mouse models of late‐stage CRC to define the effects of tumor cell‐secreted extracellular vesicles (EVs) on the antitumor immune response. T cell priming and functional assays were carried out using primary human CRC organoids and cell lines and preclinical mouse models.

**Results**: Our analyses of human CRC immune profiles and tumor‐immune cell interactions revealed that TEVs containing microRNA miR‐424 suppressed the CD28‐CD80/86 costimulatory pathway in tumor‐infiltrating T cells and dendritic cells, leading to immune checkpoint blockade therapies (ICBT) resistance. Modified TEVs with miR‐424 knocked down enhanced T‐cell mediated antitumor immune response in CRC tumor models and increased the ICBT response. Intravenous injections of modified TEVs induced tumor antigen‐specific immune responses and boosted the ICBT efficacy in CRC models that mimic aggressively progressing late‐stage disease.

**Summary/Conclusion**: Collectively, we demonstrate a critical role for TEVs in antitumor immune regulation and immunotherapy response, which could be developed as a novel treatment for immune checkpoint blockade therapy resistant colorectal cancer.

### OD06.07 Metabolomic, proteomic, transcriptomic and fatty acid profiling of glioblastoma‐stem cells and their small extracellular vesicles

Tolga Lokumcu, DKFZ Heidelberg

Lisa Schlicker, KDFZ

Klinke Glynis Fiona, Centre for Organismal Studies Heidelberg

Frederik Bethke Bethke, DKFZ

Kendra Maass, DKFZ

Karsten Richter, DKFZ

Almut Schulze, DKFZ

Violaine Goidts, DKFZ

**Introduction**: Glioblastoma is the most common and aggressive brain tumor with the median survival is about 12–15 months after diagnosis. It is an extremely aggressive tumor showing high degree of intra‐ and inter‐tumoral heterogeneity and the exceedingly heterogeneous nature of glioblastoma creates a major challenge to implement better treatment strategies. It is now known that small sub‐population of cancer cells, known as glioblastoma stem‐like cells (GSCs), are responsible for the therapy resistance of glioblastoma, resulting in low overall survival in patients. Different population of glioblastoma cells have the ability to communicate with each other and with various different cell types in the microenvironment by exchanging proteins, nucleic acids, lipids and metabolites through extracellular vesicles. The extracellular vesicles taken up by neighboring tumor cells and normal cells in the microenvironment can change the phenotype of recipient cells, which results in increased angiogenesis, immune suppression, promoted cell invasion and metabolic deregulation. The goal of our work was to identify the factors that might be responsible for the glioblastoma subtype plasticity.

**Methods**: Proneural (PN) GSCs were treated with the different fractions of conditioned medium of mesenchymal (MES) GSCs and the changes in the expression of MES/PN markers (CD44 and CD133, respectively) were determined by flow cytometry. The metabolites and fatty acids of GSCs/small extracellular vesicles were identified by GC‐MS and the proteomic profiling of GSCs/sEV was assessed by LC‐MS. Finally, the RNA content of GSCs/sEV was detected by total RNA seq.

**Results**: PN cells increased the expression of mesenchymal marker CD44 when they were treated with the conditioned medium of MES cells. Whereas PN cells treated with 2.000g and 10.000g depleted conditioned medium increased the expression of CD44, as shown with complete CM transfer, cells treated with 100.000g depleted CM lost their high CD44‐expressing phenotype. Given that exosomes are depleted by 100.000g centrifugation, our data suggest that exosomes secreted by MES cells change the expression/abundance of CD44 in recipient cells. Importantly, metabolomic, proteomic, transcriptomic and fatty acid profiling of glioblastoma‐stem cells and their small extracellular vesicles showed that GSC‐derived sEVs harbor a diverse repertoire of biomolecules, which makes them critical mediators of glioblastoma heterogeneity and cell‐to‐cell communication.

**Summary/Conclusion**: Our data show that GSC‐derived sEVs carry various biomolecules including metabolites, proteins, RNAs and fatty acids, making them crucial biological structures for the cell‐to‐cell communication and the maintenance of heterogeneity in glioblastoma.

## OD07 EVs in Tissue Protection and Repair

Chair: Benedetta Bussolati, Department of Molecular Biotechnology and Health Sciences, University of Turin, Italy

Chair: Eva Rohde, GMP Unit, Spinal Cord Injury and Tissue Regeneration Center Salzburg (SCI‐TReCS) and University Institute for Transfusion Medicine, Paracelsus Medical University Salzburg, Austria

### OD07.01 Extracellular vesicles rejuvenate aged tissues by activating the glutathione pathway

Juan Antonio Fafian‐Labora, Queen Mary University of London

Jorge Pascual‐Guerra, Hospital Ramon y Cajal

Marta Posada, Hospital Ramon y Cajal

Jesus Alarcon, Hospital Ramon y Cajal

Jose Antonio Rodríguez‐Navarro, Hospital Ramon y Cajal

Ana O'Loghlen, Queen Mary University of London

**Introduction**: Ageing is a major risk factor for many human diseases. It is a complex process that progressively compromises most of the biological functions of the organisms, resulting in an increased susceptibility to disease and death. It is characterised by different hallmarks, one of which is cellular senescence. In fact, senescent cells accumulate in different organs during ageing. Senescence is a cellular phenotype characterized by a stable cell cycle arrest and a particular secretome denominated senescence‐associated secretory phenotype (SASP). The SASP is compromised by soluble factors and extracellular vesicles (EV) but the role the latter play are understudied.

**Methods**: Functionality experiments were performed with extracellular vesicles of small size (sEV). sEV were isolated by either serial ultracentrifugation and/or size exclusion chromatography. A variety of technique involved to identify the activation of senescence or the presence of ageing‐related markers were used. In addition, different techniques involved in determining the activation of the glutathione pathway were also used both using cell culture models and tissues from old and young mice.

**Results**: Here, we have evaluated the role that EV of small size (sEV) play in ageing and cellular senescence. Previous data from our lab show that sEV from senescent cells are mediators of senescence in a non‐cell autonomous fashion. We further identified proteins within the interferon pathway as partial mediators of sEV‐mediated paracrine senescence. However, as during ageing, the organs are formed by a mixture of proliferating and senescent cells here we determined the influence of proliferating cells on senescent and ageing cells. Interestingly, we found that sEV from proliferating cells ameliorated several senescence and ageing features both in vivo and in vivo. Furthermore, we identified that sEV from proliferating cells have intrinsic glutathione‐S‐transferase (GST) activity due to their enrichment in the GST‐related protein, GSTM2. Transfection of recombinant GSTM2 into sEVs derived from old fibroblasts restores their GST and antioxidant capacity. Thus, sEVs from proliferating cells increase the levels of reduced glutathione and decrease oxidative stress including the GSTM2‐specific downstream signalling, lipid peroxidation, both in vivo and in vitro.

**Summary/Conclusion**: In conclusion, we found that sEV have an important role in the intercellular communication mediated through sEV during cellular senescence, ageing and rejuvenation processes respectively.

### OD07.02 Clinical grade MSC‐EVs promote human cartilage recovery in vitro

Maria Elisabetta Federica Palamà, Department of Experimental Medicine (DIMES), University of Genoa, Italy

Simona Coco, IRCCS Policlinico San Martino, Genoa, Italy

Daniele Reverberi, U.O. Molecular Pathology, IRCCS Policlinico San Martino, Genoa, Italy

Georgina Margaret Shaw, Regenerative Medicine Institute (REMEDI), National University of Ireland Galway (NUI Galway), Galway, Ireland,

Dario Pisignano, Nanoscience Institute CNR‐NANO (NEST), Pisa, Italy

Katia Cortese, Department of Experimental Medicine (DIMES), University of Genoa, Italy

Frances Peter Barry, Regenerative Medicine Institute (REMEDI), National University of Ireland Galway (NUI Galway), Galway, Ireland

Mary Murphy, Regenerative Medicine Institute (REMEDI), National University of Ireland Galway (NUI Galway), Galway, Ireland

Chiara Gentili, Department of Experimental Medicine (DIMES), University of Genoa, Italy

**Introduction**: Osteoarthritis (OA) is a disabling joint disorder causing articular cartilage degeneration. Currently, treatments are mainly pain‐ and symptom‐modifying, rather than disease‐modifying. Human bone marrow stromal cells (hBMSCs) have emerged as a promising paracrine mechanism‐based approach for the treatment of OA.

**Methods**: We cultured hBMSCs in a novel xeno‐free culture system (XFS). We characterized extracellular vesicles (EVs) derived from hBMSCs grown in XFS compared to a conventional fetal bovine serum (FBS) culture system, in normoxic and hypoxic culture setting. We investigated also the therapeutic potential of EVs in an in vitro model of OA. Also, miRNA content of EVs in different culture setting was investigated, to select putative miRNA that could be involved in a biological function.

**Results**: The biological effects of XFS‐ and FBS‐cultured hBMSCs was tested on IL‐1α treated human articular chondrocytes (hACs), in an experiment designed to mimic the OA environment. We observed that hBMSC‐derived EVs counteract the inflammatory state of hACs, promoting the homeostasis maintenance. This effect was streghtened by XFS culture, both in normoxia and hypoxia. Analysis of miRNA content showed the upregulation in XFS‐hBMSC‐derived EVs of miRNA known to have a chondroprotective role, such as miR‐17, miR‐140, miR‐145, miR‐30a, miR‐29a, miR‐130a, miR‐199a. Interestingly, most of the miRNA found in our preparations seem to be involved in cartilage homeostasis and they affect TGF‐beta signaling.

**Summary/Conclusion**: In conclusion, the XFS medium was found to be suitable for isolation and expansion of hBMSCs, which secrete EVs with high therapeutic function. The application of cells cultured exclusively in XFS overcomes issues of safety associated with serum‐containing media and makes ready‐to‐use clinical therapies more accessible.

### OD07.03 Mesenchymal stem cell extracellular vesicles as therapy for acute and chronic lung diseases: A systematic review and meta‐analysis

Alvin Tieu, Ottawa Hospital Research Institute

Kevin Hu, Ottawa Hospital Research Institute

Catherine Gnyra, Ottawa Hospital Research Institute

Joshua Montroy, MSc, Ottawa Hospital Research Institute

Dean Fergusson, PhD, Ottawa Hospital Research Institute

Duncan Stewart, MD, Ottawa Hospital Research Institute

David Allan, MD, Ottawa Hospital Research Institute

Manoj Lalu, MD, MDPhD, Ottawa Hospital Research Institute

**Introduction**: Mesenchymal stem cell EVs (MSC‐EVs) are reported to reduce inflammation and improve organ function in preclinical lung diseases. Prior to translation, an objective analysis of all available data is needed. Moreover, identifying EV characteristics associated with greater efficacy may help refine EV therapy. This systematic review aims to determine the efficacy of MSC‐EVs for lung diseases.

**Methods**: A protocol was registered a priori (PROSPERO CRD42020145334). MEDLINE and Embase were searched for in vivo studies of MSC‐EVs as therapy for acute lung injury (ALI), bronchopulmonary dysplasia (BPD) and pulmonary arterial hypertension (PAH). A random effects meta‐analysis was conducted to measure efficacy. Subgroup analysis identified EV methods/characteristics associated with improved efficacy. Data is presented as standardized mean differences (SMD) or risk ratios (RR) with 95% confidence intervals (CI).

**Results**: After screening 1167 reports, 52 studies met our eligibility criteria. For ALI, MSC‐EVs markedly reduced lung injury (SMD 4.33, CI 2.92‐5.73), vascular permeability (SMD 2.43, CI 1.82‐3.05), and mortality (RR 0.39, CI 0.22‐0.68). No differences were seen between MSC tissue sources, immunocompatibility or isolation techniques. However, small EVs were more effective than large EVs. For BPD, alveolarization was improved by EVs (SMD 1.45, CI 0.82‐2.08) with small EVs being more consistently beneficial then small/large EVs. In PAH, right ventricular systolic pressure (SMD 4.16, CI 2.64‐5.68) and hypertrophy (SMD 2.80, CI 1.91‐3.68) were attenuated by EVs. Allogeneic EVs were more beneficial than xenogeneic. In both BPD and PAH studies, EVs from tangential flow filtration (TFF) displayed no efficacy, whereas ultracentrifugation alone or paired with other techniques resulted in improved therapeutic benefit.

**Summary/Conclusion**: All outcomes were significantly improved by MSC‐EVs demonstrating the potential of EV therapy for treating acute and chronic lung diseases. MSC tissue source or route of administration did not alter efficacy, whereas ultracentrifugation and small EVs were more consistently beneficial. More direct comparisons of isolation techniques and EV subtypes are needed to optimize EV therapy for clinical translation.

### OD07.04 Honeybee Royal Jelly extracellular vesicles display promising antibacterial and pro‐regenerative properties for wound healing

Sebastian Aguayo, Pontificia Universidad Católica de Chile, Faculty of Medicine, School of Dentistry

Pamina Contreras, Clínica Alemana‐Universidad del Desarrollo, Facultad de Medicina, Centro de Medicina Regenerativa

Simón Alvarez, Clínica Alemana‐Universidad del Desarrollo, Facultad de Medicina, Centro de Medicina Regenerativa

Orlando Ramirez, Clínica Alemana‐Universidad del Desarrollo, Facultad de Medicina, Centro de Medicina Regenerativa

Christina M.A.P. Schuh, Clínica Alemana‐Universidad del Desarrollo, Facultad de Medicina, Centro de Medicina Regenerativa

**Introduction**: Honeybee Apis mellifera Royal Jelly (RJ), has been used in medicinal treatments by many cultures for centuries. RJ is a honeybee hypopharyngeal gland secretion known to be antibacterial and to exert beneficial effects on wound healing. However, the underlying mechanisms have not been fully elucidated yet. In recent years microvesicles have been identified as key players in cellular communication and are upcoming as promising therapeutic vehicles. Since glandular secretions present a rich source of active extracellular vesicles (EVs), we hypothesized that EVs are present in RJ and participate in its known antibacterial and pro‐regenerative effects.

**Methods**: EVs were isolated from RJ using ultracentrifugation and analyzed for size distribution by Nanoparticle Tracking Analysis (NTA) and Transmission Electron Microscopy (TEM). Furthermore, vesicles were tested for presence of exosomal markers as well as cargo proteins with Western Blot. Antibacterial effects were tested in microplate biofilm assays using wound‐associated strain Staphylococcus aureus ATCC 29213. The effect of RJ‐EVs on human mesenchymal stem cells (MSCs) and fibroblasts was assessed in cell cycle‐ and migration assays.

**Results**: Presence of EVs was verified with NTA and TEM, and exosomal origin was confirmed by Western Blot (Syntenin, CD63, due to apis origin). Major Royal Jelly Protein 1 (MRJP1), Defensin‐1 and Jellein‐3 were identified as relevant EV cargo. RJ‐EVs displayed strong bactericidal and biofilm‐disrupting effects on both bacterial strains (concentration ∼10:1 RJ‐EVS to CFU). Both MSCs and fibroblasts internalized bee‐derived RJ‐EVs resulting in a pro‐migratory effect for MSCs and increased fibroblast proliferation.

**Summary/Conclusion**: RJ‐EVs displayed promising properties for application in wound healing. On the one hand, they are strongly antibacterial and biofilm‐disruptive; on the other hand, RJ‐EVs are not limited to intra‐species communication: their internalization by mammalian cells promotes either migration or proliferation of cells associated to wound healing. The underlying mechanisms can be associated to the cargo proteins identified, as Defensin‐1, Jellein‐3 and MRJP1 are known to be antibacterial, and the latter to furthermore act as a growth factor for several cell types. Summarizing, we were the first to identify EVs in RJ and link known RJ proteins to active EV cargo. Being encased by vesicles, these proteins are delivered into mammalian cells in a directional manner, increasing their therapeutic potential. Thus, RJ‐EVs could be used as a novel vesicle‐based therapy for the treatment of chronic wounds, especially when associated to infection and biofilm development.

### OD07.05 Enhanced angiogenesis and wound healing in vivo induced by extracellular vesicles from therapeutic grade allogeneic human placental stromal cells

Martin Wolf, Cell Therapy Institute, Spinal Cord Injury and Tissue Regeneration Center Salzburg (SCI‐TReCS), Paracelsus Medical University (PMU), Salzburg, Austria

Rodolphe Poupardin, Cell Therapy Institute, Spinal Cord Injury and Tissue Regeneration Center Salzburg

Constantin Blöchl, Dept. of Biosciences, Paris Lodron University Salzburg

André Cronemberger Andrade, Paracelsus Medical University

Fausto Gueths Gomes, Cell Therapy Institute, Spinal Cord Injury and Tissue Regeneration Center Salzburg

Patricia Ebner, Cell Therapy Institute, Spinal Cord Injury and Tissue Regeneration Center Salzburg

Balazs Vari, Cell Therapy Institute, Spinal Cord Injury and Tissue Regeneration Center Salzburg

Essi Eminger, Cell Therapy Institute, Spinal Cord Injury and Tissue Regeneration Center Salzburg (SCI‐TReCS), Paracelsus Medical University (PMU), Salzburg, Austria

Heide Marie Binder, Cell Therapy Institute, Spinal Cord Injury and Tissue Regeneration Center Salzburg

Anna M Raninger,Cell Therapy Institute, Spinal Cord Injury and Tissue Regeneration Center Salzburg

Gabriele Brachtl, Cell Therapy Institute, Spinal Cord Injury and Tissue Regeneration Center Salzburg

Andreas SpittlerCore Facility Flow Cytometry & Surgical Research Laboratories, Medical University of Vienna

Thomas Heuser, Vienna Biocenter Core Facilities, Medical University, Vienna

Astrid Obermayer,Dept. of Biosciences, Paris Lodron University Salzburg

Christian G Huber,Dept. of Biosciences, Paris Lodron University Salzburg

Katarina Schallmoser,Department of Transfusion Medicine and SCI‐TReCS, PMU, Salzburg, Austria

Hans‐ Dieter Volf,BCRT & Institute of Medical Immunology, Charite ‐ Univeritätsmedizin Berlin, Germany

Strunk Dirk,Cell Therapy Institute, Spinal Cord Injury and Tissue Regeneration Center Salzburg (SCI‐TReCS), Paracelsus Medical University (PMU), Salzburg, Austria

**Introduction**: Cell therapy approaches using of the shelf allogeneic products have shown surprising results despite lack of engraftment of the transplanted cells. Their efficacy was so far considered to be mostly mediated by secreted trophic factors. We tested if extracellular vesicles (EVs) contribute to their mode of action. Here we provide evidence that EVs derived from therapeutic placental‐expanded (PLX) stromal cells are potent inducers of angiogenesis and modulate immune cell proliferation in a dose‐dependent manner.

**Methods**: Crude EVs were enriched >100‐fold from large volume PLX conditioned media via tangential flow filtration (TFF) as determined by tuneable resistive pulse sensing (TRPS). Additional TFF purification was devised to separate EVs from cell‐secreted soluble factors. EV identity was confirmed by western blot, electron microscopy and super resolution microscopy. To identify the mode of action we compared isolated EV preparations with corresponding soluble factors via proteomic analysis, in vitro angiogenesis assay, stimulation capacity of Immune cells, and in vivo wound healing mouse model.

**Results**: Surface marker profiling of tetraspanin‐positive EVs identified expression of cell‐ and matrix‐interacting adhesion molecules. Differential tandem mass tag proteomics comparing PLX‐EVs to PLX‐derived soluble factors revealed significant differential enrichment of 258 proteins in purified PLX‐EVs involved in angiogenesis, cell movement and immune system signalling. At the functional level, PLX‐EVs and cells in contrast to soluble factors inhibited T cell mitogenesis and showed different activation patterns of proliferation pathways in Monocytes and T cells. PLX‐EVs and soluble factors displayed dose‐dependent proangiogenic potential by enhancing tube‐like structure formation in vitro as well as enhanced vessel density and wound healing in an in vivo mouse model.

**Summary/Conclusion**: Our findings indicate that the mode of PLX action involves an EV‐mediated proangiogenic function and immune response modulation that may help explaining clinical efficacy beyond presence of the transplanted allogeneic cells.

### OD07.06 In vivo administration of extracellular vesicles derived from amniotic fluid stem cells improves lung development in experimental pulmonary hypoplasia

Rebeca Figueira, PhD, MSc, The Hospital for Sick Children

Noor Ramy, The Hospital for Sick Children

Lina Antounians, MSc, The Hospital for Sick Children

Kasra Khalaj, PhD, MSc, The Hospital for Sick Children

Sree Gandhi, The Hospital for Sick Children

Augusto Zani, MD, PhD, FACS, FAAP, The Hospital for Sick Children

**Introduction**: We previously reported that administration of extracellular vesicles derived from amniotic fluid stem cells (AFSC‐EVs) to in vitro rat lung models of pulmonary hypoplasia (PH) restores lung growth and maturation. We also reported that intra‐amniotically injected ExoGlow+ AFSC‐EVs reach the fetal lungs. Herein, we investigated whether AFSC‐EV administration could rescue normal lung growth also in an in vivo rat model of PH.

**Methods**: EVs were isolated from pre‐cleared conditioned medium of cKit+rat AFSCs using ultracentrifugation (100,000g, 14h), and characterized for size (nanoparticle tracking analysis), morphology (transmission electron microscopy), and expression of canonical EV protein markers CD63, Hsp70, Flo‐1, and TSG101 (Western blot). To reproduce fetal PH, nitrofen was administered to pregnant rats at embryonic day (E) 9.5. During the canalicular stage of lung development (E18.5‐19.5), dams were anaesthetized, uterine horns were exposed, and 100uL of either AFSC‐EVs (n = 9) or saline (n = 9) were injected into the amniotic sac. Fetal lungs were harvested at E21.5. Groups were compared for: 1) airway branch density (mean linear intercept with H&E); 2) number of vessels per mm2 (immunofluorescence: von Willebrand factor for endothelial cells + smooth muscle actin for muscle cells); 3) mean wall thickness of pulmonary arteries with 10–60um diameter (H&E). Statistics: t‐test.

**Results**: Compared to saline injection alone, intra‐amniotic administration of AFSC‐EVs to nitrofen‐exposed fetal lungs restored airway branch density (p = 0.0029), rescued the number of vessels per mm2 (p = 0.04), and decreased the mean wall thickness of pulmonary arterioles (p < 0.0001).

**Summary/Conclusion**: Antenatal in vivo administration of AFSC‐EVs improves fetal lung development by restoring airway branching and vascularization levels in the rat model of PH. AFSC‐EV antenatal administration represents a promising therapy for fetuses with PH.

### OD07.07 Therapeutic Potential of Pericyte‐derived EVs for Skeletal Muscle Recovery Following Hindlimb Immobilization in Aged Mice

Yu‐Fu Wu, M.S., University of Illinois at Urbana‐Champaign

Noah Kim, University of Illinois at Urbana‐Champaign

Rebecca Jung, University of Illinois at Urbana‐Champaign

Marni D. Boppart, Sc.D., University of Illinois at Urbana‐Champaign

**Introduction**: Skeletal muscle disuse atrophy is the decline of muscle mass that results from physical unloading. Recovery via physical rehabilitation is often incomplete in older adults, which can lead to disability and loss of independence. Our laboratory previously demonstrated that perivascular stem cells (CD146+ pericyte) can effectively recover muscle mass in young adult mice following a period of immobilization (IM). The purpose of this study was to determine the extent to which pericytes and pericyte‐derived extracellular vesicles (EVs) could effectively improve muscle mass in aged mice.

**Methods**: Four‐month‐old and 24‐month‐old C57BL/6 mice were randomly separated into cell or EV treatment groups by age (n = 3‐5/group). One hindlimb was immobilized in full dorsiflexion via a surgical staple. After 2 weeks of IM, staples were removed, and either PBS (control), pericytes, or pericyte‐derived EVs (unprimed or hydrogen peroxide (H2O2) primed) were injected into the tibialis anterior muscle. Muscles were excised after 2 weeks of remobilization, and recovery was assessed by immunofluorescence. One‐way ANOVA was used to compare the extent of improvement between treatments within each age group.

**Results**: No significant improvement was observed with pericyte transplantation in aged mice, likely due to cell viability issues associated with the aged microenvironment. However, significant improvements in myofiber CSA and collagen remodeling were observed in both young (p = 0.011 and p = 0.002) and aged (p = 0.011 and p = 0.036) mice receiving primed EVs as compared to age‐matched PBS groups. Unprimed EVs resulted in variable responses in young and old compared to primed EVs.

**Summary/Conclusion**: Pericyte‐derived EVs represent an important new therapy that can potentially recover and rebuild muscle mass after a period of disuse in both young and older adults.

### OD07.08 Pregnancy Resembles Heterochronic Parabiosis And Amniotic Fluid Extracellular Vesicles Transfer Regenerative Potential

Pascal Goldschmidt‐Clermont, M.D., Alzady International, LLC

Corinne Hubinont, M.D., Saint Luc University Hospital

Bruna Turnes, Ph.D., Harvard Medical School

Ian A. White, PhD, Neobiosis, LLC

**Introduction**: Aging is an evolutionary conserved mechanism driving genetic diversity in all eukaryotic organisms. This process of “timing out” individuals allows resources to be made available to “novel” genetic variants (offspring) potentially better suited to take advantage of local environments or to inhabit new. Aging progresses due to a gradual, but continuous, depletion of endogenous stem cells, which are capable of maintaining homeostasis through tissue repair. As a consequence, aged individuals experience progressively slower and less complete tissue repair. This process has been interrupted in murine laboratory experiments referred to as heterochronic parabiosis, where an old individual regains youthful repair potential when connected to a young individual's blood supply though a common dermal patch. This phenomenon is thought to happen by boosting the regenerative capacity of endogenous tissue stem cells. A natural counterpart exists and that is pregnancy.

**Methods**: Pregnancy is an unusual form of heterochronic parabiosis, as the placenta prevents most blood cells to be exchanged between the young and the older parabionts. Instead, plasma, including small extracellular vesicles (EVs), can readily cross the placental barrier. These nanosized EVs, which accumulate in the amniotic fluid, are essential for fetal organogenesis and growth and also impact the mother, as they are essential for maternal physiological changes in response to the stresses of pregnancy. Using an array‐based multiplex ELISA approach we have identified over 200 bioactive cytokines, proteases and soluble receptors related to inflammation and tissue repair from purified EVs derived from human amniotic fluid (hAFEVs). We tested the hypothesis that these EVs are capable of improving age‐related pathology by employing a murine model of chronic inflammatory mono‐arthritis of the knee.

**Results**: Through intra‐articular injection of hAFEVs we learned that hAFEVs were immunologically tolerated and reduced knee‐inflammation and promoted endogenous tissue repair in treated animals (n = 12) compared to control animals (n = 8) up to 21 days post injury.

**Summary/Conclusion**: These data suggest a potential strategy for accessing benefits of heterochronic parabiosis to boost the regenerative capacity of endogenous tissue stem cells in humans and regain the homeostatic repair potential of the young, using a readily available tissue source.

## OD08 EV Characterization

Chair: Joshua Welsh, Translational Nanobiology Section, Laboratory of Pathology, National Cancer Institute, National Institutes of Health, United States

Chair: Randy Carney, UC Davis, United States

### OD08.01 Immunophenotyping of single extracellular vesicles via nano‐flow cytometry for nasopharyngeal carcinoma diagnosis

Yunyun Hu, Department of Chemical Biology, College of Chemistry and Chemical Engineering, Xiamen University

Bin Hu, The First Affiliated Hospital of Xiamen University

Ye Tian, Department of Chemical Biology, College of Chemistry and Chemical Engineering, Xiamen University

Qin Lin, The First Affiliated Hospital of Xiamen University

Xiaomei Yan, PhD, Department of Chemical Biology, College of Chemistry and Chemical Engineering, Xiamen University

**Introduction**: Nasopharyngeal carcinoma (NPC) is one of the most common malignant epithelial tumors which presents a major public health problem worldwide, especially in South China. Epstein Barr Virus (EBV) infection is a vital factor that contributes to NPC pathogenesis. Although no viral particles are detected in the tumor, the EBV genome is present in virtually all NPC cells, encoding numbers of latent gene products including membrane proteins (LMP1 and LMP2). The traditional clinical diagnostics of NPC relies on invasive tissue biopsy and less‐sensitive medical imaging technology. Extracellular vesicles (EVs, ∼40'1000 nm) are emerging as a promising substitute of liquid biopsy for disease diagnosis. Herein, we performed protein analysis on single EVs from both the NPC cells and NPC patients by using a laboratory‐built nano‐flow cytometer (nFCM) that enables multiparameter analysis of single EVs as small as 40 nm.

**Methods**: EVs derived from NP69 (a normal nasopharyngeal epithelial cell line), C666‐1 (an NPC cell line consistently harboring EBV), CNE1 (an EBV‐negative NPC cell line) and NPC patient plasma were isolated by differential ultracentrifugation. Upon immunofluorescent staining, the expression levels of five markers (LMP2A, LMP1, EpCAM, PD‐L1, and EGFR) for isolated EVs were analyzed and quantified.

**Results**: Upon screening of a series of cancer biomarkers, the expression levels of LMP2A and LMP1 for EVs derived from C666‐1 were elevated compared to those of NP69 and CNE1, which indicated their potential for NPC diagnosis. When plasma EVs were analyzed by nFCM, the concentration of LMP2A+ EVs showed a great performance in distinguishing clinical patients with stage II‐IV NPC from healthy donors (AUC = 0.97). Of note, when concurrent analysis of other cancer biomarkers was conducted, the combined signature offered up to 100% sensitivity, specificity, and accuracy for early‐stage NPC detection.

**Summary/Conclusion**: nFCM provides a straightforward and non‐invasive approach for immunophenotyping of EVs derived from NPC patients. In particular, multiple protein profiling facilitates diagnosis of early‐stage NPC with 100% accuracy. To further expand the application of the identified marker set, we will enroll more clinical samples to evaluate the diagnostic potential in distinguishing NPC from other cancer types.

### OD08.02 Characterisation of EVs separated from plasma and BALF of patients diagnosed with lung lesions including NSCLC

Magdalena Dlugolecka, Chair and Department of Biochemistry, Doctoral School, Medical University of Warsaw

Jacek Szymanski, Chair and Department of Biochemistry, Medical University of Warsaw

Lukasz Zareba, Chair and Department of Biochemistry, Medical University of Warsaw

Zuzanna Homoncik, Chair and Department of Biochemistry, Medical University of Warsaw

Joanna Domagala‐Kulawik, Department of Internal Diseases, Pneumonology and Allergology, Medical University of Warsaw

Malgorzata Polubiec‐Kownacka, Department of Surgery, Institute of Tuberculosis and Lung Diseases

Malgorzata Czystowska‐Kuzmicz, Chair and Department of Biochemistry, Medical University of Warsaw

**Introduction**: The molecular characterisation of tumor‐derived extracellular vesicles (EVs) can be beneficial for diagnostic and prognostic purposes. In lung diseases like non‐small cell lung cancer (NSCLC), bronchopulmonary lavage fluid (BALF) seems to be a respectable source of tumour‐derived EVs. Using modern analytic methods we phenotyped and compared EV populations from patients’ BALF and plasma.

**Methods**: Plasma EVs were separated using size‐exclusion chromatography (SEC). BALF from the lung affected by lung cancer or another lesion (cBALF) and BALF from the not affected lung (hBALF) were collected and EVs were separated by differential ultracentrifugation. EV enriched samples were characterised by Western blot, Cryo‐Transmission electron microscopy (Cryo‐TEM), bead‐assisted flow cytometry (using a mix of magnetic beads with antibodies against CD63, CD9 and CD81) and FL‐NTA, where EV enriched samples were labelled with a membrane dye (CMDR) and PE dyed antibodies against typical EV‐markers (CD9, CD81, CD63).

**Results**: Cryo‐TEM imaging showed that BALF EVs consists exclusively of double‐membrane vesicles, whereas plasma EVs have much more complex morphology than both BALF EV types, containing mostly, besides typical exosomal vesicles, single‐membrane liposomes. Exosomal markers were present in different amounts in all EV types analysed by Western blot. In contrast, flow cytometry showed that BALF EVs bound to magnetic beads were positive for CD63, CD9 and CD81 whereas in case of plasma EVs detectable was only CD63 in a small EV subpopulation. FL‐NTA showed a higher particle concentration in plasma than in both BALF types in respect to one ml of each biological fluid. The particles from plasma were only positive for CMDR, whereas both BALF EV types were positive not only for membrane‐labelling but also for exosomal markers.

**Summary/Conclusion**: Deep FL‐NTA analysis allowed us to highlight the significant differences between EVs from plasma and BALF. We have shown that there were no significant differences between cBALF and hBALF EVs.

### OD08.03 In situ imaging of bacterial membranous extensions and their associated protein complexes using electron cryo‐tomography

Mohammed Kaplan, California Institute of technology

Grant Jensen, California Institute of technology

**Introduction**: The ability to produce membranous extensions (MEs) in the form of membrane vesicles and tubes is a widespread phenomenon amongst bacteria. Despite this, our knowledge of the molecular ultrastructure of these extensions and their associated protein complexes (PCs) in different species remains limited.

**Methods**: Here, we used electron cryo‐tomography (cryo‐ET) to survey the ultrastructure and formation of MEs and their associated PCs in numerous bacterial species.

**Results**: We describe the ultrastructure of MEs in the form of nanotubes with a uniform diameter (with or without an internal scaffold) or irregular diameter, pearling nanotubes, connected chains of vesicles (with or without neck‐like connectors), budding vesicles and nanopods. These forms were present either exclusively or combined in a species‐specific manner. Furthermore, we identified various PCs associated with the MEs and were located either randomly or exclusively at the tip of the MEs including a secretin like complex and a crown‐like complex associated with cell‐lysis.

**Summary/Conclusion**: In total, our results show that the molecular architecture of MEs and their associated PCs are variable even amongst closely‐related species.

### OD08.04 Application of extracellular vesicle surface activity in the presence of gas‐liquid and liquid‐liquid interfaces for their characterization by using the dynamic surface/interfacial tension probe

Ekaterina Tsydenzhapova, Moscow Institute of Physics & Technology

Roman Chuprov‐Netochin, Moscow Institute of Physics & Technology

Sergei German, Skolkovo Institute of Science and Technology

Anastasiia Merdalimova, Skolkovo Institute of Science and Technology

Alexey Yashchenok, Skolkovo Institute of Science and Technology

Sergey Leonov, Moscow Institute of Physics & Technology

Dmitry Gorin, Skolkovo Institute of Science and Technology

Mikhail Skliar,University of Utah

Vasiliy S. Chernyshev, Skolkovo Institute of Science and Technology

**Introduction**: Surface activity is a dynamic phenomenon where molecules (e.g. proteins) or nanoparticles migrate towards and adsorb to a surface (liquid‐air) or interface (liquid‐liquid) to reach a more energetically favorable state, causing a change in surface tension (ST) or interfacial tension (IT). Despite the high potential of extracellular vesicles (EVs) in medicine, there is a lack of information about their potential surface activity at the liquid‐air and liquid‐liquid interfaces which are regularly encountered in research, development of diagnostic platforms and drugs. Since EVs are known to contain membrane macromolecules (e.g. proteins) that are exposed to surrounding fluid, we hypothesized their potential surface activity.

**Methods**: EVs were isolated from MCF7, MCF10a, MDA‐MB‐231 and SKOV3 cell culture media by ultrafiltration followed by SEC. SEC fractions containing EVs were analyzed by NTA, DLS, Scanning‐EM, BCA and WB. For surface activity analysis, dynamic ST and IT of each SEC fraction containing EVs in 1x PBS was measured for at least 4 hrs (n = 3) in the presence of air, isopropyl myristate, toluene and cyclohexane by using a custom‐made real‐time pendant‐drop tensiometer at room temperature. Statistical analysis was done by using Matlab software.

**Results**: SEC sample characterization confirmed presence of EVs with high purity in 3 fractions for each cell line which were used for ST and IT measurements. At early age of the pendant drop ST and IT values agreed with values reported for pure fluid‐air and fluid‐fluid systems. As time progressed the ST and IT values decreased and after 4 hours the value was 5–30% lower than at early age of the pendant drop (p < 0.05) due to EV diffusion to the surface/interface and adsorption. A distinct correlation between EV quantity and tension values was found at specific time points.

**Summary/Conclusion**: Dynamic ST and IT measurements allowed to determine that EVs are surface active, diffuse and adsorb at the liquid/air and liquid/liquid interfaces. This EV property not only can play a critical role in their biological function but also opened doors to a new approach for EV characterization, especially their quantitation by using the highly informative dynamic ST/IT probe.

### OD08.05 Multi‐Objective Calibration and Standardization of EV Measurement Using Nanoscale Flow Cytometry

Edwin van der pol, Amsterdam University Medical Centers

Fabrice Lucien, MD PhD, Mayo Clinic

**Introduction**: Nanoscale flow cytometry (nFC) is a powerful method that combines fluorescence and light‐scattering detection to enumerate a large number of extracellular vesicles (EVs) within minutes. nFC also offers the opportunity to evaluate the clinical utility of EVs as prognostic markers in human diseases. However, single particle detection can be challenged by variances in pre‐analytical conditions and instrumentation settings. To overcome this, impressive collaborative efforts resulted in a framework for standardized reporting of EV flow cytometry (FC) experiments (MIFlowCyt‐EV). By building on previous milestones, we further evaluated the performance and limitations of nFC in EV enumeration from biofluids. We also provide novel insights on pre‐analytical conditions and acquisition parameters to reliably enumerate specific EV subpopulations.

**Methods**: Biofluid samples (blood and urine) of healthy donors and prostate cancer patients were prepared according to the recommendations of MISEV 2018. The Apogee A60‐MicroPlus nanoscale flow cytometer was used for all FC experiments. Gag‐GFP+HEK293T EVs, spiked in biofluids were used as biological reference. Different antibody labeling methods were also assessed for detection of EV subsets with differential abundance in biofluids. Light‐scatter and fluorescence detection of EVs was assessed with more than 12 different acquisition settings. By using Mie Theory, optical configurations, calibrations, and all EV data were converted into standardized units.

**Results**: The optimal concentration ranges of biofluid samples for reproducible EV enumeration after serial dilution were determined and most samples could meet median concentrations within the ranges. For EV immunophenotyping, the number of dyes per antibody and choice of fluorophores were critical factors for fluorescence detection of EVs. Multiplexing with up to 3 antibodies targeting different EV subpopulations did not affect concentration measurements. However, double staining of same EV subset may result in underestimation of EV concentrations from potential steric hindrance of antibodies on surface of EVs.

**Summary/Conclusion**: In conclusion, this study provides optimal settings and recommendations for reproducible EV enumeration using nFC, and ultimately help other research groups strive for developing EV‐based liquid biopsies.

### OD08.06 Quantitative analysis of 2D and 3D cell culture‐isolated EV populations by ILM, TRPS and NTA technologies

Liliia Paniushkina, Medical Center Freiburg, Exosomes and Tumour biology group

Martin Wolf, Cell Therapy Institute, Spinal Cord Injury and Tissue Regeneration Center Salzburg (SCI‐TReCS), Paracelsus Medical University (PMU), Salzburg, Austria

Krisztina V. Vukman, Department of Genetics, Cell‐ and Immunobiology, Semmelweis University, Budapest, Hungary

Strunk Dirk, Cell Therapy Institute, Spinal Cord Injury and Tissue Regeneration Center Salzburg (SCI‐TReCS), Paracelsus Medical University (PMU), Salzburg, Austria

Edit I. Buzás, Department of Genetics, Cell‐ and Immunobiology, Semmelweis University, Budapest, Hungary

Irina Nazarenko, Medical Center Freiburg, Germany

**Introduction**: Different methods are used to measure particle number for characterisation of isolated EVs. These methods bring internal discrepancies when measuring the same EV sample. This study aimed to quantify four EV populations of different size isolated from 2D and 3D cell culture models by three technologies. We introduce an application of Interferometric Light Microscopy (ILM) in comparison with TRPS and NTA.

**Methods**: Noticed a growing interest in physiological models, we used 2D and 3D prostate cancer (22Rv1) cell cultures to analyse surface molecular signatures and EV cargo. We compared the particle number and size of four EV populations: EV5, EV12, EV120, sEV designated according to centrifugation speed and purified by density gradient centrifugation resulting in 10 fractions per population. The fractions were analysed using ILM, TRPS and NTA technologies following by the characterization of tissue and EV‐specific markers.

**Results**: We observed a highly significant difference in particle number measured by all three technologies in EV5. The TRPS gives the lowest concentration of particles (∼107 particles/ml), while ILM ‐ ∼109 particles/ml, and NTA ‐ ∼1011 particles/ml. When comparing the measured particle size, NTA was able to detect only small particles (30‐200nm), while TRPS using different membranes distinguished between small and large particles, showing two populations: 50–150nm and 200–600nm. Only ILM was able simultaneously estimated small and large particles in a size range: 80–720nm.

**Summary/Conclusion**: All three techniques ILM, TRPS and NTA detect and evaluate the particles at 80–200nm. The TRPS and ILM estimated large particles, while NTA did not. Our results show that ILM might simultaneously evaluate a polydisperse sample detecting small and large particles in one preparation. However, using TRPS, a broader size range of particles 50–2000 nm can be seen, while ILM is limited by 80nm for small. Our result demonstrated the importance of applying different methods of particle quantification to characterize different EV populations.

### OD08.07 Association between infrared spectra and the lipidomic profile of human milk exosomes

Victoria Ramos‐Garcia, Neonatal Research Unit, Health Research Institute Hospital La Fe, Avda Fernando Abril Martorell 106, 46026 Valencia, Spain

Isabel Ten‐Domenech, Neonatal Research Unit, Health Research Institute Hospital La Fe, Avda Fernando Abril Martorell 106, 46026 Valencia, Spain

Abel Albiach‐Delgado, Neonatal Research Unit, Health Research Institute Hospital La Fe, Avda Fernando Abril Martorell 106, 46026 Valencia, Spain

Alba Moreno‐Giménez, Neonatal Research Unit, Health Research Institute Hospital La Fe, Avda Fernando Abril Martorell 106, 46026 Valencia, Spain

María Gormaz, Division of Neonatology, University & Polytechnic Hospital La Fe, Avda Fernando Abril Martorell 106, 46026 Valencia, Spain

Anna Parra‐Llorca, Division of Neonatology, University & Polytechnic Hospital La Fe, Avda Fernando Abril Martorell 106, 46026 Valencia, Spain

María Círia, Regenerative Medicine and Heart Transplantation Unit, Health Research Institute Hospital La Fe, Avda Fernando Abril Martorell 106, 46026 Valencia, Spain

Pilar Sepúlveda, Regenerative Medicine and Heart Transplantation Unit, Health Research Institute Hospital La Fe, Avda Fernando Abril Martorell 106, 46026 Valencia, Spain

David Pérez‐Guaita, Department of Analytical Chemistry, University of Valencia, 50 Dr. Moliner Street, research building, 46100 Burjassot, Valencia, Spain

Bernhard Lendl, Institute of Chemical Technologies and Analytics, Technische Universität Wien, Getreidemarkt 9/164. A 1060 Vienna, Austria

Guillermo Quintás, Health and Biomedicine, Leitat Technological Center, Carrer de la Innovació, 2, 08225 Terrassa, Spain

Julia Kuligowski, Neonatal Research Unit, Health Research Institute Hospital La Fe, Avda Fernando Abril Martorell 106, 46026 Valencia, Spain

**Introduction**: Exosomes are nanosized (50‐100 nm) membrane vesicles released by fusion of the multivesicular body with the plasma membrane. The main objective of this study was to determine the feasibility of a rapid characterization of the lipidomic profile of exosomes by infrared spectroscopy using exosomes isolated from human milk (HM) as a model example.

**Methods**: Exosomes were isolated employing a multi‐stage ultracentrifugation procedure. After a single‐phase extraction, lipidomic fingerprinting was carried out using ultra‐high performance liquid chromatography quadrupole‐time‐of‐flight mass spectrometry (UPLC"qTOF‐MS) operating in positive and negative ionization modes. Automated MSMS‐based annotation of metabolites was carried out using HMDB, METLIN, in silico LipidBlast and MSDIAL MS/MS databases. Then, dry films of 2 μL of HM exosomes suspended in PBS were directly analysed by Attenuated Total Reflectance ‐ Fourier Transform Infrared (ATR‐FTIR) spectroscopy.

**Results**: A total of 693 LC‐MS features detected in HM exosomes were successfully annotated. The classes with the most annotated features were glycerolipids (230), glycerophospholipids (217), sphingolipids (173), and fatty acyls (20). Multivariate analysis showed significant associations between specific regions of the ATR‐FTIR spectra and the concentrations of different lipid classes. Besides, the UPLC‐MS and ATR‐FTIR datasets were analysed by principal component analysis. Using the distances among samples in the PC1‐PC2 score space as criteria, the significance of the similarity between the trends observed in both scores plots was assessed by the Mantel test (p‐value < 0.005).

**Summary/Conclusion**: A correlation between the lipidomic profile of HM exosomes and their ATR‐FTIR spectra has been obtained, indicating that the latter technique can be used for a rapid evaluation of the composition of HM exosomes, thus supporting the development of a new tool for a direct and fast quality control of the exosome isolation procedure.

### OD08.08 Determination of the kinetics of circulating small extracellular vesicles with an in situ membrane biotinylation strategy

Zili Yu, School and Hospital of Stomatology, Wuhan University

Yi Zhao, School and Hospital of Stomatology, Wuhan University

Gang Chen, School and Hospital of Stomatology, Wuhan University

**Introduction**: The in vivo kinetics of circulating small extracellular vesicles (sEVs), which possess great potential to serve as biomarkers for disease diagnosis or delivery vectors for personalized therapy, remain elusive.

**Methods**: The in vivo biosafety, kinetics and biodistribution of intravenously injected DSPE‐PEG‐Biotin were systematically evaluated in mice. Biotinylation of circulating sEVs were characterized by immuno‐electron microscopy using streptavidin‐conjugated gold nanoparticles. The level of biotinylated circulating sEVs at serial time points were monitored with flow cytometry. The in vivo kinetics of each subtype of circulating sEVs using in situ biotinylation strategy were evaluated with the antibodies against different cell markers.

**Results**: The concentration of biotin in the circulation of mice was rapidly decreased and almost undetectable at 12 h after injection. The in situ membrane biotinylation strategy is universal to label circulating sEVs with biotin biofriendly and efficiently in different animal models. The level of biotinylated circulating sEVs rapidly decreased with time, and finally fallen to the baseline level at 3 days after injection of DSPE‐PEG‐Biotin. The lifetime (halftime) was 1 (0.14), 2 (0.40), 2 (0.45), 2 (0.50), 2 (0.54) and 5 (0.95) days for endothelium‐derived sEVs, macrophage derived sEVs, platelet‐derived sEVs, leukocyte‐derived sEVs, lymphocyte‐derived sEVs and erythrocyte‐derived sEVs, respectively. The lifetime of tumor‐derived PD‐L1+ sEVs in circulation was around 2 days (halftime: 0.99 days). The circulating sEVs were biotinylated by the intravenously injected DSPE‐PEG‐Biotin mainly through the direct way other than donor cell‐assisted manner.

**Summary/Conclusion**: This study realized the first reliable in situ biotinylation strategy and revealed the dynamics kinetics of circulating sEVs, which will undoubtedly beneficial to the understanding of fundamental aspects of circulating sEVs physiology in vivo and have implications for the design and feasibility of circulating sEVs‐based therapeutics.

## OD09 All Aboard! EV Loading and Release

Chair: Anindya Mukhopadhya, School of Pharmacy and Pharmaceutical Sciences & Trinity Biomedical Sciences Institute, Trinity College Dublin and Trinity St. James's Cancer Institute, Dublin 2, Ireland

Chair: Irina Nazarenko, Medical Center Freiburg, Germany

### OD09.01 Drug‐induced lysosomal impairment drives the release of extracellular vesicles carrying autophagy‐ssociated markers

Lorena Urbanelli, Università di Perugia

Krizia Sagini, Department of Surgery, Division of Cancer Biology and Therapeutics, Cedars‐Sinai Medical Center

Sandra Buratta, Università di Perugia

Federica Delo, Università di Perugia

Roberto Maria Pellegrino, Università di Perugia

Carla Emiliani, Università di Perugia

**Introduction**: Amiodarone (AM) is a cationic amphiphilic drug used as antiarrhythmic agent. It is known to induce phospholipidosis (PLD), i.e. the accumulation of phospholipids into multilamellar structures within organelles of the endo‐lysosomal system. Extracellular vesicles (EVs) are now considered an additional manner to transmit intercellular signals, but they have been initially identified as a system to dispose extracellularly unnecessary cell material. Because of the important role played by the endolysosomal system in the biogenesis and secretion of EVs, we investigated their role in PLD.

**Methods**: AM‐treated HEK‐293 cells were engineered to produce fluorescently labelled vesicles by expression of the mCherry‐CD63 fusion protein. EVs were isolated from cell media by differential ultracentrifugation and both medium/large and small florescent EVs were retrieved in the 10K and 100K faction, respectively.

**Results**: AM induces the release of a higher number of EVs, mostly of medium/large size. Although EVs released upon AM treatment do not display significant morphological changes by EM or altered size distribution by NTA, they show a dose dependent increase of autophagy associated markers by IB. Proteolytic digestion shows that at least one of these markers, LC3B, is localized within EVs. Determination of EV phospholipid content by LC/MS showed that medium/large EVs released upon AM treatment contain more phospholipids than those released by untreated cells, indicating that AM‐treated cells release EVs enriched in phospholipids to possibly alleviate their intracellular accumulation. Drugs commonly used to block autophagy such as chloroquine or bafilomycin A also induce a higher release of EVs enriched in autophagic markers.

**Summary/Conclusion**: Our findings indicates that EVs enriched in autophagy markers may be related to lysosomal impairment in general and not to the specific type of accumulated substrate. Improving lysosomal function and autophagy by transfection of TFEB transcription factor, a master gene regulating lysosomal biogenesis, prevents AM‐induced EV release. This result confirms that the degradative cell capability is a key factor in determining undigested material fate (intracellularly degraded or extracellularly released) and suggests that this could be a feasible target to attenuate PLD‐induced abnormalities.

### OD09.02 Drug repositioning screening for an inhibitor of EV secretion in ovarian cancer cells

Yusuke Yoshioka, Tokyo Medical University

Akira Yokoi, Nagoya University

Takahiro Ochiya, PhD, Department of Molecular and Cellular Medicine, Tokyo Medical University

**Introduction**: Cancer‐related EVs with their ability to act within the tumor microenvironment and distally and thus play a major role in tumor progression and metastasis. For example, we found that EVs derived from highly metastatic ovarian cancer cells promote peritoneal dissemination in vivo (Yokoi A et al., Nat Commun, 2017). Therefore, inhibition of EV secretion from cancer cells can serve as a novel therapeutic tool to inhibit cancer metastasis. This study focused on the screening of small‐molecule inhibitors for EV secretion in ovarian cancer cells.

**Methods**: We used an original screening system based on ExoScreen assay for monitoring CD9 positive EV secretion (Yoshioka Y et al., Nat Commun, 2014). After screening, we used ExoView to measure EV secretion as a validation of our screening results. To observe the influence of small molecules on cell growth, a proliferation assay was undertaken using IncuCyte. The EV secretion rate of cells was normalized to the cell growth rate. Using this screening system and a chemical compound library containing 1271 small molecules, inhibitors for EV secretion were identified in the ovarian cancer cell line ES‐2.

**Results**: Based on the first screening result, 45 small molecules were selected as putative inhibitors for EV secretion. These small molecules were further validated by ExoScreen. As a result of the validation, 8 small molecules were found to inhibit EV secretion in ES‐2 cells. To confirm the screening result, 4 cell lines, including 2 non‐cancer cell lines, were treated these 8 molecules and measured EV secretion by ExoView. Some molecules inhibited EV secretion in a cancer cell‐specific manner. These 8 molecules did not affect cell proliferation compared to 0.1% DMSO treated cells.

**Summary/Conclusion**: Here, we identify inhibitors for EV secretion in ovarian cancer cells. Based on these results, we are now analyzing the effects of these candidate molecules on gene expression in cancer cells, and we plan to test their therapeutic effects using a mouse model of ovarian cancer transplantation.

### OD09.03 Controlling cell‐material interactions to tune therapeutic extracellular vesicle production

Stephen B. Lenzini, University of Illinois at Chicago

Singwan Wong, University of Illinois at Chicago

Angela Song, University of Illinois at Chicago

Raymond Bargi, University of Illinois at Chicago

Dolly Mehta, University of Illinois at Chicago

Jae‐Won Shin, University of Illinois at Chicago

**Introduction**: Success in biomanufacturing of therapeutic EVs from mesenchymal stromal cells (MSCs) depends on a fundamental understanding of how EVs are produced and transported in physiologically relevant environments. We recently show that both matrix stress relaxation properties and water transport through aquaporin‐1 enable EVs to deform and travel through the dense mesh of the extracellular matrix (Lenzini et al., Nat Nano 15: 217–223, 2020). However, it remains unclear how matrix properties impact EV production by MSCs. Since mechanical forces regulate membrane trafficking and mechanosensing, we hypothesized that substrate mechanics regulate EV production.

**Methods**: Alginate polymer was conjugated with an integrin binding peptide Arg‐Gly‐Asp (RGD). Alginate hydrogels with tunable stiffness were formed by adipic acid dihydrazide crosslinking. Human bone marrow MSCs (Lonza) were seeded on hydrogels followed by washing to remove unattached cells. To measure EV number, Nanoparticle Tracking Analysis (NTA) via NanoSight NS300 (Malvern) was used.

**Results**: MSCs produce ∼10 times more EVs on a soft hydrogel than on a rigid polystyrene surface, and ∼2.5 times more than on a stiffer hydrogel. Treatment with blebbistatin does not impact stiffness dependent EV production, suggesting this effect is independent of myosin‐II. EVs are produced more rapidly when MSCs are adhered to soft substrates for 4 hours versus 24 hours. Consistent with this result, activating integrins with Mn2+ decreases EV production, suggesting that soft substrates may enhance EV production by decreasing activation of integrins. Importantly, we show that EVs from MSCs on different substrates resolve a lipopolysaccharide‐induced model of acute lung injury in mice.

**Summary/Conclusion**: The results propose an optimal substrate stiffness and cell adhesion time to enhance EV production from MSCs. Production strategies designed based on these results will have a profound impact on advancing biomanufacturing of EVs from MSCs.

### OD09.04 Extracellular vesicles containing I‐BAR proteins are released from the cell plasma membrane in an Arp2/3 dependent manner

Delphine M. Muriaux, CNRS & University of Montpellier

**Introduction**: Extracellular vesicles are nanometric membrane vesicles produced by cells and involved in cell‐cell communication. Extracellular vesicles (EVs) formation can occur in endosomal compartments (exosomes) or at the cell plasma membrane (microvesicles). How these cellular vesicles bud from the cell plasma membrane is not completely understood. I‐BAR proteins are cytosolic proteins, when activated, bind to the plasma membrane and are involved in plasma membrane protrusion formation including filopodia and lamellipodia. These proteins contain a conserved I‐BAR domain which sense and induce negative membrane curvatures at the plasma membrane. I‐BAR proteins also interact with actin co‐factors to induce membrane protrusions. Here we explore if ectopic I‐BAR proteins, such as IRSp53, IRTKS and Pinkbar, are in EVs.

**Methods**: To explore these hypotheses, we purified EVs from productive human 293T cells and characterize plasma membrane I‐BAR EVs using immunoblots, interferometric microscopy, Atomic Force microscopy and immuno‐fluorescence coupled to TIRF‐Microscopy on EVs, several exosomal markers, siRNA and actin drugs.

**Results**: We found that the I‐BAR EVs are small vesicles of 200nm diameter in average mainly associated with CD81, CD9, ALIX and less CD63 but a subtype of these I‐BAR EVs are CD81 negative, wich are produced from the cell plasma membrane in a TSG101 independent manner and in an Arp2/3 dependent manner.

**Summary/Conclusion**: Our results thus reveal that these previously undescribed I‐BAR EVs represent a subset of plasma membrane microvesicles whose production depends on branched actin.

### OD09.05 Characterising treatment‐induced exosome release reveals novel insights into biogenesis pathways and the pathogenesis of ovarian cancer

Elise H. Padbury, Department of Biological and Medical Sciences, Oxford Brookes University

Štefan Bálint, Kennedy Institute of Rheumatology, University of Oxford

Emanuela Carollo, Department of Biological and Medical Sciences, Oxford Brookes University

Esther Becker, Department of Physiology, Anatomy and Genetics, University of Oxford

David R F. Carter, Department of Biological and Medical Sciences, Oxford Brookes University

**Introduction**: Due to the high level of heterogeneity within Extracellular Vesicle (EV) subtypes, key questions in EV biogenesis remain unanswered. By analysing how external cues modulate the release of multivesicular body (MVB) derived EVs, we can begin to classify exosome subpopulations and dissect the pathways which underly their biogenesis. Interestingly, we have identified the transient receptor potential channel 3 (TRPC3) as a potential facilitator of Ca2+‐mediated exosome release by ovarian cancer cells, but its role in pathogenesis is unclear.

**Methods**: Using a pH‐sensitive CD81 live‐cell reporter, we directly visualised MVBs fusing with the plasma membrane (PM) in SKOV3 ovarian cancer cells by total internal reflection fluorescence (TIRF) microscopy. Basal MVB‐PM fusion was characterised and compared with fusion following stimulation with histamine, ionomycin or the TRPC3 activator GSK 1702934A. Protein content of basal and induced EVs was determined using label‐free quantitative mass spectrometry. Proliferation, migration and invasion assays were used to evaluate the phenotypic effect of TRPC3 activation on SKOV3 cells.

**Results**: Stimulation with TRPC3 activator, histamine and ionomycin increased the rate of MVB‐PM fusion in SKOV3 cells. Treatment‐induced differences were also apparent in the size, fluorescence and duration of fusion events, and may be suggestive of distinct exosome biogenesis pathways. We also report novel fusion dynamics including localised, synchronised MVB‐PM fusion. Proteomic profiling of treatment‐induced EVs revealed distinct protein compositions which provide a mechanistic insight into their biogenesis pathways. Finally, modulating the activity of TRPC3 altered the growth and behaviour of SKOV3 cells.

**Summary/Conclusion**: Our work provides novel insights into the dynamics of exosome biogenesis and the molecular factors which facilitate the release of exosome subpopulations, potentially linking exosome biogenesis with the pathogenesis of ovarian cancer.

### OD09.06 CD47 interactions with exportin‐1 regulate targeting of m7G‐capped RNAs to extracellular vesicles

Sukhbir Kaur, Laboratory of Pathology, Center for Cancer Research, National Cancer Institute, National Institutes of Health, Bethesda, USA

Alejandra Cavazos Saldana, Laboratory of Pathology, Center for Cancer Research, National Cancer Institute, National Institutes of Health, Bethesda, USA

Jennifer D. D. Petersen, PhD, Section on Integrative Biophysics, Division of Basic and Translational Biophysics, Eunice‐Kennedy‐Shriver National Institute of Child Health and Human Development, National Institutes of Health, Bethesda, Maryland, USA

Anush Arakelyan, Section on Intercellular Interactions, Division of Basic and Translational Biophysics, Eunice Kennedy‐Shriver National Institute of Child Health and Human Development, National Institutes of Health, Bethesda, USA

Abdel G. Elkahloun, Cancer Genetics Branch, National Human Genome Research Institute, National Institutes of Health, Bethesda, USA.

Leonid Margolis, Section on Intercellular Interactions, Division of Basic and Translational Biophysics, Eunice Kennedy‐Shriver National Institute of Child Health and Human Development, National Institutes of Health, Bethesda, USA

Joshua Zimmerberg, Section on Integrative Biophysics, Division of Basic and Translational Biophysics, Eunice‐Kennedy‐Shriver National Institute of Child Health and Human Development, National Institutes of Health, Bethesda, Maryland, USA

Lisa M Jenkins, Laboratory of Cell Biology, Center for Cancer Research, National Cancer Institute, National Institutes of Health, Bethesda, USA

David G Jordan, Laboratory of Pathology, Center for Cancer Research, National Cancer Institute, National Institutes of Health, Bethesda, USA

Andy D Tran, Confocal Microscopy Core Facility, Center for Cancer Research, National Cancer Institute, National Institutes of Health, Bethesda, USA

David D. Roberts, Laboratory of Pathology, Center for Cancer Research, National Cancer Institute, National Institutes of Health, Bethesda, USA

**Introduction**: CD47 is a ubiquitously expressed membrane protein that binds the ligands thrombospondin‐1 (TSP‐1) and signal‐regulatory protein alpha. Previously, we have shown that CD47+ EVs contain distinct non‐coding RNAs, including miRNAs, relative to CD63+ and MHC1+ EVs released by the same cells suggesting that the packaging of noncoding RNAs into specific subpopulations of EVs is directed by CD47 (PMID: 29416092), The mechanisms by which CD47 directly or indirectly regulates which RNAs are packaged into EV remain unknown.

**Methods**: EVs released from WT and CD47‐ T cells were evaluated using miRNA sequencing, real‐time PCR and RNA‐immunoprecipitation. Interactions between CD47 and exportin‐1/Ran complex was identified by mass spectrometry and confirmed by using co‐immunoprecipitation, subcellular localization, flow cytometry, and confocal and electron microscopy.

**Results**: EV released from human CD47‐ T cells and in cd47‐/‐ mouse plasma were enriched in 5’‐7‐methylguanosine (m7G)‐capped miRNAs and mRNAs that depend on the exportin‐1/RanGTP pathway. Globally, more precursor/seed miRNAs than mature miRNAs exhibited shared CD47‐dependence between EVs and cells or uniquely differed in WT versus CD47‐ EVs. Therefore, CD47 may preferentially regulate trafficking of precursor rather than mature miRNAs into EVs. Knockdown of CD47 in WT cells or TSP1‐1 treatment correspondingly enhanced levels of capped‐RNAs released in EV and re‐expressing CD47 in null cells decreased their levels. Mass spectrometry and co‐immunoprecipitation identified specific interactions of CD47 with components of the exportin‐1/Ran nuclear export complex and its known cargos and between the CD47 cytoplasmic adapter ubiquilin‐1 and the exportin‐1/Ran complex. Interaction of CD47 with exportin‐1 was inhibited by leptomycin B, which inactivates exportin‐1 and increased levels of cap‐dependent RNAs in EV released from wild type but not CD47‐ T cells. We have further identified CD47 in a subset of vesicles within MVBs visualized by electron microscopy with immunogold labeling in WT cells. Treatment with LMB did not alter the number of MVBs or the abundance of CD47‐expressing vesicles therein. Therefore, some release of CD47‐expressing EVs occurs via the MVB pathway, but exportin‐1 does not regulate the release of CD47‐expressing EVs at the level of MVB biogenesis. CD47 generally limits RNA export in EVs rather than their cellular expression.

**Summary/Conclusion**: These findings indicate that CD47‐dependent TSP1‐1 signaling regulates levels of cap‐dependent pre‐miRNAs and mRNAs released in EVs at least in part through ubiquilin‐1‐ and GTP‐dependent physical interactions of CD47 with the exportin‐1/Ran transport complex.

### OD09.07 Single‐cell extracellular vesicle secretion detection with a home‐use scanner

YAO LU, Dalian Institute of Chemical Physics, Chinese Academy of Sciences

Fengjiao Zhu, Dalian Institute of Chemical Physics, Chinese Academy of Sciences

**Introduction**: We reported a high‐throughput single‐cell EV secretion analysis method based on a domestic home use scanner without cell counting, which combines gold nanoparticle enhanced silver staining and Poisson distribution.

**Methods**: The hard silicon mold was prepared by photolithography, and PDMS was poured on the mold to prepared a high throughput microwell array chip for capturing single cells. By optimizing the cell density, the Poisson distribution can be used to count the number of single cells in the microchip. To visualize EVs, standard ELISA procedures were implemented. A desktop scanner was used to record and read the results.

**Results**: By combining gold nanoparticle enhanced silver staining with Poisson distribution, overcoming the dependence on large and expensive instruments and making this platform an ideal choice for single‐cell EV secretion analysis in resource‐limited environments. The platform consists of an antibody‐coated poly‐L‐lysine glass slide used to capture EVs and a high‐throughput microwell array chip used to capture single cells. The distribution of cells on the microwell chip obeys Poisson distribution, and ∼1000 single cells were reliably captured (1213±354, n = 9). CD9+CD63+EV and CD63+EV secreted from OSCC cell line, and OSCC primary cells were analyzed, which revealed the cell heterogeneity at the single‐cell level.

**Summary/Conclusion**: We successfully developed a high‐throughput single‐cell EV secretion analysis method based on a domestic home use scanner without cell counting. OSCC cells have significant heterogeneity in the number and secretion rate of EV secretion.

### OD09.08 Unravelling intercellular communication between keratinocytes and melanocytes in the skin: extracellular vesicles and their role in the regulation of melanocyte function

Cécile Giordano, Institut Curie CNRS UMR144 Structure and membrane compartments

Ilse Hurbain, Institut Curie, CNRS UMR144, Structure et Compartiments Membranaires, Université Paris Sciences et Lettres, Paris, France. Institut Curie, CNRS UMR144, Plateforme d'imagerie cellulaire et tissulaire (PICT‐IBiSA), Université Paris Sciences et Lettres, Pari

Graça Raposo, PhD, Institut Curie, CNRS UMR144, Structure et Compartiments Membranaires, Université Paris Sciences et Lettres, Paris, France. Institut Curie, CNRS UMR144, Plateforme d'imagerie cellulaire et tissulaire (PICT‐IBiSA), Université Paris Sciences et Lettres, Pari

Gisela d'Angelo, Institut Curie, CNRS UMR144, Structure et Compartiments Membranaires, Université Paris Sciences et Lettres, Paris, France

**Introduction**: Extracellular vesicles (EVs), which facilitate the transfer of proteins, lipids and genetic material molecules between cells, are recognized as an additional mechanism for intercellular communication. In the epidermis, the communication between melanocytes and keratinocytes is tightly regulated to maintain skin homeostasis. Melanocytes synthetize the melanin pigment in melanosomes that are transferred to keratinocytes via an intricate dendritic network in order to color and photo‐protect the skin against UV‐B radiations. We have recently shown that EVs secreted by keratinocytes modulate pigment synthesis in melanocytes, the first step of melanogenesis. However, whether keratinocyte‐EVs play additional roles in melanogenesis is not known. We hypothesize that keratinocyte‐EV contribute to melanogenesis by promoting melanocyte dendricity for an efficient pigment transfer.

**Methods**: We combined here cell biology, optical and electron microscopy, biochemical, and molecular biology approaches.

**Results**: We show that keratinocyte‐EVs induce morphological changes of melanocytes, and increase their dendricity. Exploiting our previously identified keratinocyte‐EV content, we also show that keratinocyte‐EV depleted for Rac1 protein, a member of the Rho family of GTPases, do not affect melanocyte morphology, and fail to promote melanocyte dendricity. Finally, by electron microscopy, we observe an accumulation of mature melanosomes at the tip of the dendrites. We are currently investigating the mechanisms underlying such an effect.

**Summary/Conclusion**: Altogether our results support the view that Rac1 containing‐EVs released by keratinocytes provide a means for modulating melanocyte dendricity and morphology. They also put forward a new function of keratinocyte‐EVs in programing and instructing melanocytes so that they coordinate their functions: melanosome biogenesis, dendricity, and accumulation of mature melanosomes that will ultimately by transfer by melanocytes to keratinocytes. Importantly, dysregulation of these pathways could underlie pigmentary disorders like melanoma and skin carcinoma.

## OD10 Pre‐clinical Studies: New Insights

Chair: Carlos Salomon, The University of Queensland, Australia

### OD10.01 Extracellular vesicles from mesenchymal stromal cells combined with tissue engineering improve cardiac function, reduce fibrosis and modulate immune response in acute myocardial infarcted pigs

Marta Monguió‐Tortajada, Health Science Research Institute Germans Trias i Pujol (IGTP), Can Ruti Campus, Badalona, Spain

Cristina Prat‐Vidal, ICREC, Health Science Research Institute Germans Trias i Pujol (IGTP), Can Ruti Campus, Badalona, Institut d'Investigació Biomèdica de Bellvitge‐IDIBELL, CIBERCV, Instituto de Salud Carlos III, Spain

Daina Martínez‐Falguera, ICREC, Health Science Research Institute Germans Trias i Pujol (IGTP), Can Ruti Campus, Badalona, Department of Medicine, Universitat de Barcelona (UB),CIBERCV, Instituto de Salud Carlos III, Spain

Micaela Munizaga‐Larroudé, ICREC, Health Science Research Institute Germans Trias i Pujol (IGTP), Can Ruti Campus, Badalona, Department of Medicine, Universitat Autònoma de Barcelona (UAB), CIBERCV, Instituto de Salud Carlos III, Spain

Carolina Soler‐Botija, ICREC, Health Science Research Institute Germans Trias i Pujol (IGTP), Can Ruti Campus, Badalona, CIBERCV, Instituto de Salud Carlos III, Spain

Miriam Moron‐Font, REMAR‐IVECAT Group, Health Science Research Institute Germans Trias i Pujol (IGTP), Can Ruti Campus, Badalona, Spain

Adriana Cserkoova, ICREC, Health Science Research Institute Germans Trias i Pujol (IGTP), Can Ruti Campus, Badalona, Spain

Antoni Bayes‐Genis, ICREC, Health Science Research Institute Germans Trias i Pujol (IGTP), Can Ruti Campus, Badalona, CIBERCV, Instituto de Salud Carlos III; Cardiology Service, Germans Trias i Pujol University Hospital; Department of Medicine, UAB, Spain

Francesc E. Borràs, REMAR‐IVECAT Group, Health Science Research Institute Germans Trias i Pujol (IGTP), Can Ruti Campus; Nephrology Service, Germans Trias i Pujol University Hospital, Badalona, Spain

Santiago Roura,ICREC, Health Science Research Institute Germans Trias i Pujol (IGTP), Can Ruti Campus, Badalona, CIBERCV, Instituto de Salud Carlos III, Spain

Carolina Gálvez‐Montón, ICREC, Health Science Research Institute Germans Trias i Pujol (IGTP), Can Ruti Campus, Badalona, CIBERCV, Instituto de Salud Carlos III, Spain

**Introduction**: Accumulating evidence supports the potential of extracellular vesicles (EVs) from mesenchymal stromal cell (MSC) as a therapy for cardiac healing after myocardial infarction (MI). Nevertheless, neither their efficient administration nor their therapeutic mechanisms are fully elucidated. Here, we evaluate the preclinical efficacy of a tissue engineering approach to locally deliver porcine cardiac adipose tissue MSCs (cATMSC‐EV) in an acute MI pig model.

**Methods**: Pigs (n = 24) were subjected to permanent ligation of the coronary artery. After 30 min, animals were randomized to Untreated or treated groups with a tissue engineered graft composed of a decellularized pericardial scaffold filled with peptide hydrogel and cATMSC‐EV purified by size exclusion chromatography (EV‐treated group) or buffer (Control group) placed over the post‐MI myocardium. Cardiac troponin levels and cardiac MRI revealed consistent myocardial damage and infarct size in all animals.

**Results**: After 30 days, cardiac function was significantly improved with less ventricle dilatation in the EV‐treated group, indicating less myocardial remodelling. MRI showed reduced scar size in EV‐treated animals, correlating with a decrease of fibrosis in the distal zone and increased vascular density in the infarct core. Less macrophage infiltration and more anti‐inflammatory phenotype (CD163+CD73+) were found in the infarct of treated animals. Surprisingly, local delivery of cATMSC‐EV also triggered a systemic effect, reducing PBMC increase 2‐days post‐MI and modulating systemic CD73+ and CCR2+ monocytes, related to immunomodulation and fibrosis modulation.

**Summary/Conclusion**: These results highlight the clinical potential of cATMSC‐EV in modulating key features of ischemic injury and promoting cardiac repair after MI.

### OD10.03 MSC exosomes promote cartilage and subchondral bone repair in a porcine osteochondral defect model

Wei Seong Toh, PhD, Faculty of Dentistry, National University of Singapore

Shipin Zhang, PhD, Faculty of Dentistry, National University of Singapore

Keng Lin Wong, MD, Department of Orthopaedic Surgery, Yong Loo Lin School of Medicine, National University of Singapore

Xiafei Ren, MD, Department of Orthopaedic Surgery, Yong Loo Lin School of Medicine, National University of Singapore

Ruenn Chai Lai, PhD, Institute of Molecular and Cell Biology, Agency for Science, Technology and Research, Singapore

Sai Kiang Lim, PhD, Institute of Molecular and Cell Biology, Agency for Science, Technology and Research, Singapore

James Hoi Po Hui, MD, Department of Orthopaedic Surgery, Yong Loo Lin School of Medicine, National University of Singapore

**Introduction**: We had previously reported the efficacy of human mesenchymal stromal cell (MSC) exosomes in repair of critical‐size osteochondral defects in both rats and rabbits. However, small animals unlike humans have inherent tendency for spontaneous healing of cartilage defects in addition to differences in size and biomechanics. To enable clinical translation of MSC exosomes, we therefore proposed a validation of the efficacy of MSC exosomes in a large animal model.

**Methods**: Bilateral osteochondral defects measuring 6mm diameter and 1mm depth were surgically created on the weight‐bearing area of the medial femoral condyles of 24 knees in 12 micropigs. Immediately after surgery and at days 8 and 15 post‐surgery, 6 micropigs in exosome/HA group received sequential administration of 1mg exosomes in 1ml phosphate‐buffered saline (PBS) followed by 1ml hyaluronic acid (HA; Synvisc(R)) in both knees, whereas the other 6 micropigs in the HA group received 1ml of PBS followed by 1ml HA in both knees. Except for magnetic resonance imaging (MRI) performed on day 15, 2 and 4 months, macroscopic, histological and micro‐computed tomography (micro‐CT) assessments were performed at 4 months.

**Results**: As early as day 15 post‐surgery, exosome/HA treated defects had a better MRI score of 4.46 than the score of 3.63 by HA treated defects (P = 0.017). The MRI scores for exosome/HA treated defects continued to improve, and were consistently higher than that for HA treated defects at both 2 months (7.83 vs 5.79; P = 0.023) and 4 months (9.25 vs 6.71; P = 0.024). At 4 months, exosome/HA treated defects had significantly better ICRS macroscopic score (9.22 vs 7.25; P = 0.008) and histological score (79.71 vs 65.10; P = 0.032) than HA treated defects. Micro‐CT analysis further revealed structural improvements in the subchondral bone with significantly higher BV/TV (49.38% vs 39.73%; P = 0.046) and Tb.Th (0.18mm vs 0.13mm; P = 0.009), but not Tb.N and Tb.Sp in exosome/HA treated defects, compared to HA treated defects.

**Summary/Conclusion**: Our results show that MSC exosomes and HA combination administered at a clinically acceptable frequency of three intra‐articular injections can promote osteochondral repair with significantly improved morphological and histological outcomes in a clinically relevant porcine model. Our study highlights a clinically translatable protocol utilizing MSC exosomes as an off‐the‐shelf and cell‐free therapeutic for patients with osteochondral injuries and potentially osteoarthritis.

### OD10.04 Anti‐fibrotic effects of Membrane Particles from mesenchymal stromal cells in renal ischemia reperfusion injury mouse model

Ana Merino, Nephrology and Transplantation, Department of Internal Medicine, Erasmus MC, University Medical Center Rotterdam, The Netherlands

Zhaoyu Du, Internal medicine department, Erasmus MC

Anusha Shankar, Internal Medicine department, Erasmus MC

Sander Korevaar, Internal Medicine department, Erasmus MC

Derek Reijerkerk, Internal Medicine department, Erasmus MC

Carla Baan, Nephrology and Transplantation, Department of Internal Medicine, Erasmus MC, University Medical Center Rotterdam, The Netherlands

Marlies Reinders, Internal Medicine Department, Erasmus MC

Martin Hoogduijn, Nephrology and Transplantation, Department of Internal Medicine, Erasmus MC, University Medical Center Rotterdam, The Netherlands

**Introduction**: Membrane particles (MP) are nanovesicles artificially generated by extrusion of the mesenchymal stromal cell (MSC) membranes. MP were designed to circumvent the risks of MSC therapy such as a poor biodistribution due to their large size and unknown mechanistic behaviour after infusion, while keeping the reparative and immunomodulatory properties of MSC. We have demonstrated earlier that MP have immunomodulatory properties, endothelial regenerative capacity, and antifibrotic effect on lung fibroblasts in vitro. In this study, the aim is to demonstrate the efficacy of MP as an antifibrotic treatment on a renal ischemia reperfusion injury (IRI) mouse model.

**Methods**: Ischemia injury was performed by clamping the right kidney of the mice for 37 minutes. The MP were intravenously infused 3–5 hours after ischemia. Animals were sacrificed and the kidney harvested 3 days after renal IRI. Four groups of mice were analysed: Sham, IRI, IRI+MP derived from 1 million of MSC, and IRI+MP derived from half million of MSC. Gene expression of proinflammatory cytokines was measured in the kidneys such as IL6, and TNFa; kidney injury marker KIM1, infiltration of monocytes and lymphocytes; and profibrotic markers such as TGFb, PAI‐1, fibronectin, tenascin C, collagen I, and III.

**Results**: We found no difference between IRI mice treated with MP and IRI untreated mice respect to the proinflammatory markers IL6, TNFa, injury marker KIM‐1 or infiltration of monocytes and lymphocytes. IRI induced an upregulation of the gene expression of profibrotic markers such as TGFb and PAI‐1, and proteins from the extracellular matrix. Interestingly, both doses of MP significantly decreased the expression of the TGFb, PAI‐1 and the main extracellular matrix proteins involved in fibrogenesis.

**Summary/Conclusion**: Our findings show that MP have antifibrotic effects on renal IRI, opening a new potential avenue for treatment of organ fibrosis.

## OD11 Interplay of Viruses and EVs

Chair: Linglei Jiang, Johns Hopkins University, United States

Chair: Shilpa Buch, University of Nebraska Medical Center, United States

### OD11.01 Extracellular vesicles are involved in circulation of Hepatitis B Virus RNA in infected cells’ supernatant and patients’ serum

Delphine Bousquet, INSERM U1052‐ Cancer Research Center of Lyon (CRCL), Lyon, France

Doohyun Kim, INSERM U1052‐ Cancer Research Center of Lyon (CRCL), Lyon, France

Annie Adrait, Univ. Grenoble Alpes, CEA, INSERM, IRIG, BGE, Grenoble, France

YOHAN COUTE, Univ. Grenoble Alpes, CEA, INSERM, IRIG, BGE, Grenoble, France

Maria Guadalupe Martinez, INSERM U1052‐ Cancer Research Center of Lyon (CRCL), Lyon, France

Alexia Paturel, INSERM U1052‐ Cancer Research Center of Lyon (CRCL), Lyon, France

Aaron Hamilton, Roche Molecular Diagnostics, Pleasanton, CA

Marintha Heil, Roche Molecular Diagnostics, Pleasanton, CA

Massimo Levrero, INSERM U1052‐ Cancer Research Center of Lyon (CRCL), Lyon, France

Barbara Testoni,INSERM U1052‐ Cancer Research Center of Lyon (CRCL), Lyon, France

Fabien Zoulim, INSERM U1052‐ Cancer Research Center of Lyon (CRCL), Lyon, France

**Introduction**: Despite the availability of effective vaccine, chronic Hepatitis B virus (HBV) infection remains a global health burden. Current antiviral strategies (nucleos(t)ide analogues, NUCs) are unable to eliminate the virus from infected hepatocytes and, thus, achieve a complete cure. Relevant and non‐invasive biomarkers are necessary to ameliorate patients’ management and the evaluation of new therapies. In this study, we aim at better characterizing the compartments containing extracellular HBV RNAs, which were recently proposed as a new surrogate marker of intrahepatic viral activity.

**Methods**: Supernatant from HBV‐infected HepG2‐NTCP cells, treated or not with NUCs, was collected and processed through sucrose/iodixanol gradient separation, to allow physical separation of extracellular vesicles (EVs) and viral particles according to their buoyant density. Viral and EVs‐associated proteins were analyzed by Western Blotting and Elisa, while HBV RNAs were detected by specific digital droplet (dd)PCR. NTA and mass spectrometry‐based proteomic analyses were used to further characterize the EVs components.

**Results**: Elisa assays for viral surface proteins after gradient separation showed that virions were found in fractions corresponding to a density of 1,21‐1,25 g/ml. Western Blotting for CD9 and CD63, markers of exosomes/EVs were detected only in lower density fractions (1,13"1,19 g/m), which were deprived of viral proteins. Interestingly, HBV RNAs were detected not only in virion‐like particles but also in lighter gradient fractions, suggesting that EVs could contribute to carry the circulating HBV RNA pool. No significant difference was found in NUC‐treated vs untreated samples. To further investigate the nature of EVs detected in light density fractions, NTA analysis was performed, showing that these fractions were indeed containing EVs in size spanning from 30 to 150 nm. Finally, proteomic analyses of the same fractions revealed the presence of specific markers of exosomes (CD63, CD9, TSG101 or HSC70).

**Summary/Conclusion**: Our study will shed light on the molecular biology of serum HBV RNA secretion and will aid the development of serum HBV RNA as a novel biomarker for chronic HBV infection.

### OD11.02 Human cytomegalovirus infection modifies trophoblastic small extracellular vesicles secretion and composition, facilitating viral dissemination in recipient cells

Mathilde Bergamelli, Institut Toulousain des Maladies Infectieuses et Inflammatoires (Infinity), Université de Toulouse, INSERM, CNRS, UPS, Toulouse, France

Hélène Martin, Institut Toulousain des Maladies Infectieuses et Inflammatoires (Infinity), Université de Toulouse, INSERM, CNRS, UPS, Toulouse, France

Jean‐Michel Mansuy, CHU Toulouse, Hôpital Purpan, Laboratoire de Virologie, Toulouse, France

Ilse Hurbain, Institut Curie, CNRS UMR144, Structure et Compartiments Membranaires, Université Paris Sciences et Lettres, Paris, France. Institut Curie, CNRS UMR144, Plateforme d'imagerie cellulaire et tissulaire (PICT‐IBiSA), Université Paris Sciences et Lettres, Pari

Jacques Izopet, CHU Toulouse, Hôpital Purpan, Laboratoire de Virologie, Toulouse, France.

Graça Raposo, PhD, Institut Curie, CNRS UMR144, Structure et Compartiments Membranaires, Université Paris Sciences et Lettres, Paris, France. Institut Curie, CNRS UMR144, Plateforme d'imagerie cellulaire et tissulaire (PICT‐IBiSA), Université Paris Sciences et Lettres, Pari

Daniel Gonzalez‐Dunia, Institut Toulousain des Maladies Infectieuses et Inflammatoires (Infinity), Université de Toulouse, INSERM, CNRS, UPS, Toulouse, France

Gisela d'Angelo, Institut Curie, CNRS UMR144, Structure et Compartiments Membranaires, Université Paris Sciences et Lettres, Paris, France

Cécile Malnou, Institut Toulousain des Maladies Infectieuses et Inflammatoires (Infinity), Université de Toulouse, INSERM, CNRS, UPS, Toulouse, France

**Introduction**: Congenital infection by human Cytomegalovirus (hCMV) is a major public health issue because of its high incidence and the variety of induced neurological sequelae in neonates but despite intense research, pathophysiology of hCMV infection is not yet fully understood. As they participate in mother‐fetus communication, we examined the hypothesis that placental small extracellular vesicles (sEVs) could contribute to placental and fetal injury.

**Methods**: sEV from trophoblastic cells infected or not by hCMV were purified by differential ultracentrifugation and density gradient. sEV structure and composition were further analyzed by flow cytometry, nanoparticle tracking analysis, immune‐electron microscopy and proteomics. Finally, impact of trophoblastic sEV on hCMV permissiveness in recipient cells was examined.

**Results**: We observed that hCMV infection increased secretion of trophoblastic sEV that presented smaller size compared to non‐infected condition. Protein content of trophoblastic sEV was modified by hCMV infection, with the presence of viral proteins and with modification in cellular protein composition, suggesting that they could play a role in “priming” infection in recipient cells and thus facilitate further viral infection. Trophoblastic sEV were internalized in fetal cells with time and dose effect. Incubation of fetal cells with sEV from trophoblastic cells infected by hCMV increased significantly the infection rate compared to fetal cell incubated with sEV prepared from non‐infected cells.

**Summary/Conclusion**: In conclusion, we showed that hCMV infection modifies both trophoblastic sEV secretion and protein content, therefore priming fetal recipient cells for a future infection. Hence sEVs may be crucial mediators that could play an important role in maternal‐fetal transmission of hCMV by facilitating viral dissemination towards the fetus.

### OD11.03 Administration of Amniotic Fluid derived Extracellular Vesicles in COVID‐19 Long Hauler Patients

Maria Ines Mitrani, M.D., Ph.D., Organicell Regenerative Medicine

Michael A. Bellio, Ph.D., Organicell Regenerative Medicine

Gwendolyn Haskell, Pharma D, Organicell Regenerative Medicine

George C. Shapiro, M.D., Organicell Regenerative Medicine

**Introduction**: Post‐COVID‐19 infection symptoms such as mental fog, tachycardia, and extreme fatigue are just a few of the symptoms wreaking havoc on patients’ lives. Patients with long‐term sequelae following COVID‐19 are being called long‐haulers. To date, long‐haulers are receiving little to no guidance from physicians on their lingering COVID‐19 symptoms with no treatment options available. Zofin is an acellular biologic that contains the extracellular vesicle (EV) fraction of human amniotic fluid and is under investigation for use as a COVID‐19 therapeutic. We have recently completed 4 single patient emergency/compassionate use eINDs investigating amniotic‐fluid derived EVs in COVID‐19 long haulers under our approved parent IND 19881 to demonstrate safety and feasibility.

**Methods**: FDA and IRB approval were obtained for these single patient cases investigating Zofin treatment in an outpatient setting. IND approval numbers were: eIND 25888, eIND 26560, eIND 26561, IND 26821. The therapeutic intervention, Zofin, is an allogenic, acellular biologic derived from human amniotic fluid containing 2.3 × 10^11 particles/mL with 70–80% positive expression of exosome markers CD63 and CD81. Zofin was administered intravenous as 1mL doses on baseline, day 4 and day 8 (3 doses). The approved clinical protocol included patient follow up with biomarker testing and chest X Rays (CXR) on Day 0, 4, 8, 14, 21, 28, and 60. The primary objective of these studies was to demonstrate the safety of Zofin. All patients tested positive for COVID‐19 a minimum of 2 months prior to treatment.

**Results**: Administration of the EV product was shown to be safe in all patients. One patient had detectable bilateral pneumonia at baseline treatment that was present 2 months after discharge from the hospital. On Day 14, repeated CXR showed improvement of the patchy peripheral pulmonary opacities. Then, the CXR report on Day 21 noted that the patient's lungs were clear. Furthermore, this patient experienced extreme shortness of breath prior to treatment with baseline pulse oximetry readings were 95% when seated and 93–94% when supine on room air. Improvements in fatigue were noted soon after the second dose and the patient was able to exercise to fatigue, at which time, he did not desaturate and remained at 97–98% on room air.

**Summary/Conclusion**: The single patient IND studies were completed without any reported adverse events or safety concerns. Furthermore, these completed studies demonstrate the feasibility and a therapeutic potential of amniotic fluid‐derived EVs for COVID‐19 long hauler intervention.

## OD12 Therapeutics

Chair: Janusz Rak, Professor, Canada

Chair: Shin‐ichi Kano, Department of Psychiatry and Behavioral Neurobiology, The University of Alabama at Birmingham School of Medicine, United States

### OD12.01 Tunability of platelet‐derived extracellular vesicles

Mari Palviainen, PhD, University of Helsinki

Puutio Johanna, University of Helsinki

Johannes A. Eble, University of Munster

Masood Kamali‐Moghaddam, University of Uppsala

Pia Siljander, University of Helsinki

**Introduction**: The proteome of anuclear platelets comprises >5000 proteins and is impacted e.g. by age and disease. The platelet secretome of over 300 proteins contains e.g. growth factors and immunomodulatory proteins and therefore, platelet products, such as platelet rich plasma are used in regenerative medicine. Platelets also release extracellular vesicles (EVs), both constitutively and upon activation. We studied 1) the tunability of platelet EVs by agonists engaging different critical platelet signaling pathways, 2) the characteristics of these EVs, and 3) macrophage responses to these EVs.

**Methods**: Isolated human platelets were activated by CRP (engaging GPVI), rhodocytin (CLEC‐2), and by thrombin and collagen co‐stimulus (all thrombin and collagen receptors). Agonist concentrations and the time of activation were optimized for maximal EV yield in the shortest possible time. EVs were isolated by ultracentrifugation using cushioned density‐gradient, and then characterized by particle concentration, size distribution (NTA) and marker protein expression (Exoview R100). The inflammation‐linked proteome of EVs was analyzed in a targeted array using Olink technology. Macrophages differentiated from THP‐1 cells were treated with equal numbers of EVs for 6 and 24 hours, and the secretome of macrophages was analyzed with Luminex technology targeted for cytokines and chemokines

**Results**: Although more CD63+/CD9+ EVs were generated from activated platelets when compared to non‐activated platelets, activation by rhodocytin resulted in a markedly lower EV yield compared to CRP or TC co‐stimulus. The signaling pathways engaged during activation significantly impacted on the inflammation‐related molecular cargo. These findings were further supported by the variability of the agonist‐, but also time‐dependent changes in the secretome of the EV‐treated macrophages reflecting the tunability of platelet‐derived EVs

**Summary/Conclusion**: Upon activating platelets, agonists have distinct impacts on the characteristics of secreted EVs, their proteome and functionality. These results imply that by differential activation it is possible to create tunable EVs e.g. for immunomodulatory purposes.

### OD12.02 microRNA Enrichment of Extracellular Vesicle Content for Diabetic Wound Treatment

Ricardo C. de Abreu, Department of Molecular Genetics, Faculty of Sciences and Engineering, Maastricht University, Maastricht, Netherlands

Cristiana Ramos, Coimbra Chemistry Centre, Chemistry Department, Faculty of Science and Technology, University of Coimbra, Portugal

Clarissa Becher, Biomaterials and Stem‐Cell Based Therapeutics group, CNC‐ Center for Neuroscience and Cell Biology, University of Coimbra, Portugal

Miguel Lino, Biomaterials and Stem‐Cell Based Therapeutics group, CNC‐ Center for Neuroscience and Cell Biology, University of Coimbra, Portugal

Carlos Jesus, Biomaterials and Stem‐Cell Based Therapeutics group, CNC‐ Center for Neuroscience and Cell Biology, University of Coimbra, Portugal

Patrícia Martins, Biomaterials and Stem‐Cell Based Therapeutics group, CNC‐ Center for Neuroscience and Cell Biology, University of Coimbra, Portugal

Inês Albino, Biomaterials and Stem‐Cell Based Therapeutics group, CNC‐ Center for Neuroscience and Cell Biology, University of Coimbra, Portugal

Marta Barão, Biomaterials and Stem‐Cell Based Therapeutics group, CNC‐ Center for Neuroscience and Cell Biology, University of Coimbra, Portugal

Maria João Moreno, Coimbra Chemistry Centre, Chemistry Department, Faculty of Science and Technology, University of Coimbra, Portugal

Hugo Fernandes,Biomaterials and Stem‐Cell Based Therapeutics group, CNC‐ Center for Neuroscience and Cell Biology, University of Coimbra, Portugal

Paula da Costa martins, Department of Molecular Genetics, Faculty of Sciences and Engineering, Maastricht University, Maastricht, Netherlands

Lino FerreiraBiomaterials and Stem‐Cell Based Therapeutics group, CNC‐ Center for Neuroscience and Cell Biology, University of Coimbra, Portugal

**Introduction**: Extracellular vesicles (EVs) have been used for tissue regeneration but their native cargo may be insufficient to elicit a therapeutic effect. Here we describe a method to load small EVs with therapeutically active miRNAs and their application in a diabetic wound healing mouse model.

**Methods**: Electroporation, saponin, cholesterol, freeze‐thaw and Exo‐Fect™ were tested for their capacity to load EVs with a fluorescently‐labelled miRNA. Loading efficiency was calculated based on the percentage of total fluorescence in the EV fraction and confirmed by quantitative PCR. To test the functionality of miRNA‐transfected EVs we used a HEK‐293T cell line was transfected with miRNA‐loaded EVs. This line constitutively expresses mCherry, which is inhibited by the presence of functional miRNA. The internalization and intracellular kinetics of these EVs were analysed in endothelial cells. Finally, EVs were loaded with a pro‐survival miRNA and topically administered (2x/day for 10 days) on wounds of diabetic mice.

**Results**: Our data shows that Exo‐Fect™ is the most effective strategy to load EVs with >50% loading efficiency. This was then shown to be functional in a reporter cell line, where transfection with microRNA‐loaded EVs decreased reporter signal by 30%. Furthermore, modulated EVs were shown to be less signalled for lysosomal degradation than their native counterparts. In vivo, miRNA‐loaded EVs were able to improve wound healing in diabetic mice, partially by enhancing wound vascularization.

**Summary/Conclusion**: Our results show that Exo‐Fect™ is an efficient and functional way of loading microRNAs into EVs. Further, these modulated EVs can be used in vitro and in vivo as effective delivery agents.

### OD12.03 Exploration of blood‐cerebrospinal fluid barrier targeted extracellular vesicles in brain drug delivery

Marie Pauwels, VIB‐UGent

Adam Ceroi, VIB‐UGent

Nele Plehiers, VIB‐UGent

Caroline Van Cauwenberghe, VIB‐UGent

Sophie Steeland, VIB‐UGent

Elien Van wonterghem, VIB‐UGent

Griet Van Imschoot, VIB‐UGent

Sriram Balusu, PhD, VIB‐UGent

Florencia Linero, VIB‐UGent

Imre Mäger,Department of Paediatrics, University of Oxford, Oxford, United Kingdom.

Lien Van Hoecke, VIB‐UGent

Roosmarijn E. VandenbrouckeVIB ‐ Ghent University

**Introduction**: Successful treatment of neurological diseases is hampered by the presence of tightly regulated nervous system (CNS) barriers that restrict drug delivery to the brain. Up until now, most delivery strategies have focused on the blood‐brain barrier, while targeting the blood‐cerebrospinal fluid barrier (BCSFB) remains largely unexplored. However, the presence of transporting mechanisms, extensive secretory activity and strong vesicular trafficking indicate that BCSFB targeting strategies might have great potential. Interestingly, an increasing amount of data suggest that extracellular vesicles (EVs) (i.e. membrane derived vesicles that transfer biological cargoes between cells) naturally cross the CNS barriers. The low immunogenicity, the relatively high stability; and the cell targeting capacities of EVs, urged us to explore the BCSFB crossing capacity of EVs and their potential as brain delivery system.

**Methods**: Here, we genetically decorated HEK293T cell‐derived EVs with new BSCFB targeting ligands. Additionally, BCSFB epithelial cell‐derived EV characteristics as well as their homing capacity and protein content are currently under investigation using biodistribution assay, flow cytometry analyses, and in vitro setups.

**Results**: Our in vivo biodistribution studies indicate that BSCFB targeted EVs are a promising strategy for EV mediated brain delivery upon systemic injection. Moreover, specific EV enrichment at the BCSFB was observed by flow cytometry and in vitro BCSFB assays were used to further study the barrier crossing capacity.

**Summary/Conclusion**: In conclusion, our results indicate that BSCFB targeted EVs have great potential as brain drug delivery system. Further research is ongoing to characterize the underlying molecular mechanisms and possible loading strategies to fully exploit the potential of EVs as brain drug delivery vehicle.

### OD12.04 Exosome Topical Therapy Delivered In Bioinspired Synthetic Protein Hydrogel Enhances Cutaneous Healing Of Diabetic Wounds

Juan F. Cortes, PhD, NIDCR, NIH

Joseph Kuhn, MD, Hansjorg Wyss Department of Plastic Surgery, NYU Langone Health, New York, NY

Priya Katyal, MDPhD, Chemical and Biomolecular Engineering, NYU Tandon School of Engineering

Michael Meleties, Chemical and Biomolecular Engineering, NYU Tandon School of Engineering

Iraines De La Cruz, Hansjorg Wyss Department of Plastic Surgery, NYU Langone Health

Bibi Subhan, Hansjorg Wyss Department of Plastic Surgery, NYU Langone Health

Jin Montclare, Chemical and Biomolecular Engineering, NYU Tandon School of Engineering

Piul Rabbani, New York University School of Medicine

**Introduction**: We have used human bone marrow multipotent stromal cells (hBMSCs) and their secreted exosomes (Exo) to successfully promote wound closure in diabetic animal models of delayed healing. However, safe and easy delivery platforms that maintain exosome efficacy are necessary to for clinical translation. Here, we describe the development of Exo‐Q, a thermoresponsive soft protein matrix loaded with hBMSC‐Exo, that accelerates wound closure in a Type II diabetes model.

**Methods**: We isolated exosome preps by differential ultracentrifugation of conditioned media from hBMSCs, prior to detailed characterization. We synthesized Q, an engineered variant of the coiled‐coil domain of cartilage oligomeric matrix protein, and incorporated 3 × 109 exosomes during gelation to yield Exo‐Q hydrogels. We analyzed Exo‐Q using transmission electron microscopy (TEM) and rheology. using excisional stented wounds on 16 weeks old LepRdb/db mice, we either topically pipetted exosome preps or applied Exo‐Q hydrogels. We observed wounds for closure and collected intermediate time point tissues for biomolecular analysis and histology.

**Results**: Q self‐assembles into a fibrous matrix at low temperatures and exhibits an upper critical solution temperature phase behavior. Exo‐Q entangled protein fibers uniformly interspersed with Exo in TEM and increased Q storage modulus, indicating increased elasticity of a pliable hydrogel. Exo‐Q solubilizes at skin wound temperature (∼31°C) for sustained delivery of Exo into wounds, without invasive/painful routes. Pipetted single doses 1 × 109 or 3 × 109 Exo at post‐operative day 1 (POD1) demonstrated non‐significant changes in closure time (30.25±1.5 and 27±2 days, respectively, n = 4) vs PBS ‐treated diabetic wounds. Exo‐Q application at POD1 decreased time to closure of diabetic wounds to 17±1.4 days vs 28±1.5 days for Q vehicle alone (p < 0.01, n = 3), and correspondingly reduced wound burden relative to that with Q alone. Exo‐Q administration generated extensive CD31+ neovascularization in large areas of granulation tissue in the Leprdb/db diabetic wound bed by POD10. Exo‐Q does not interfere with wound healing progression. A single Exo‐Q dose resulted in upregulated gene expression of angiogenic and wound healing associated factors VEGF, SDF1 and PDGF in diabetic wound beds, compared to Q vehicle‐only treated wounds (all p <.05, n‐3).

**Summary/Conclusion**: Exo‐Q is an efficacious, translatable therapy that can reverse pathologic healing of diabetic wounds. Future iterations can include drugs for compound therapeutic hydrogels

### OD12.05 Extracellular vesicles derived from human liver‐stem cells improve fibrosis and inflammation associated with non‐alcoholic steatohepatitis and modified lncRNA expression profile

Stefania Bruno, sbruno, Department of Medical Sciences

Giulia Chiabotto, Department of Medical Sciences, University of Torino

Elena Ceccotti, Department fo Medical Sciences, University of Torino

Chiara Pasquino, Molecular Biothecnology Center

maria Beatriz Herrera Sanchez, Molecular Biothecnology Center

Marta Tapparo, Molecular Biothecnology Center

Cristina Grange, Department of Medical Sciences, University of Turin

Massimo Cedrino, Molecular Biothecnology Center

Giovanni Camussi, University of Turin

**Introduction**: We recently demonstrated that non‐alcoholic steatohepatitis (NASH) is improved by treatment with human liver stem cells (HLSCs). The aim of the present study was to evaluate whether EVs released by HLSCs can influence the progression of NASH and modify the hepatic lncRNA expression profile.

**Methods**: EVs were obtained by ultracentrifugation and characterized in accordance with ISEV guidelines. NASH has been induced through a methionine‐choline‐deficient diet. EV‐treatment started at week 2 (2.5 × 109 EVs twice a week) and ended at week 4, when mice were sacrificed. Liver fibrosis and inflammation have been evaluated by histological and molecular analyses, using specific histological staining, array and real time PCR analyses. LncRNAs, known to be involved in inflammatory response, have been evaluated by PCR array.

**Results**: EVs significantly improved liver function and reduced liver fibrosis and inflammation, at both morphological and molecular levels. In particular, we observed that 28 out of 29 fibrosis‐associated genes up‐regulated in livers of NASH mice were significantly downregulated by EV‐treatment. Moreover, the anti‐inflammatory effect of EV‐treatment was demonstrated by the reduction of inflammatory cells accumulated in the liver as seen by immunofluorescence. Whereas inflammatory infiltrates were present in the liver of NASH mice, almost no leukocytes were observed in mice treated with EVs. The increase of IL‐10 expression level in livers of EV‐treated NASH mice confirmed the anti‐inflammatory effect of EVs. Evaluation of lncRNA expression profile indicated that 15 lncRNA were modulated by EV‐treatment.

**Summary/Conclusion**: HLSC‐derived EVs display anti‐fibrotic and anti‐inflammatory effects in a model of chronic liver disease, leading to an improvement of liver function. In addition, EV‐treatment induces changing in the expression of lncRNAs known to be involved in inflammation, indicating their possible contribution to the anti‐inflammatory EV‐effect.

### OD12.06 Small Extracellular Vesicles Derived from Bone Marrow Stromal Cells Enhance Proliferation of Intestinal Stem Cells in Mice after Radiation

Lalitha S Y Nanduri, Department of Radiation Oncology, Albert Einstein College of Medicine

Shobhit Bhansali, Department of Radiation Oncology, Albert Einstein College of Medicine

Phaneendra K. Duddempudi, Department of Biochemistry, Albert Einstein College of Medicine

Shahin Shajahan, Department of Radiation Oncology, Albert Einstein College of Medicine

Brett Bell, Department of Radiation Oncology, Albert Einstein College of Medicine

Tatyana L. Tchaikovskaya, Department of Radiation Oncology, Albert Einstein College of Medicine

Weng‐Lang Yang, Department of Radiation Oncology, Albert Einstein College of Medicine

Shilpa Kulkarni, Department of Radiation Oncology, Albert Einstein College of Medicine

Chandan Guha, Department of Radiation Oncology, Albert Einstein College of Medicine

**Introduction**: Radiation damage to the intestine leads to acute and delayed toxicities after abdomino‐pelvic radiation therapy. We have previously demonstrated that bone marrow stromal cells (BMSCs) rescue intestine injury in mice after exposure to lethal‐dose radiation. This beneficial effect of BMSCs may be through secreting paracrine factors. Here, we investigated whether small extracellular vesicles derived from BMSC (BMSC‐sEVs) could promote proliferation and differentiation of intestinal stem cells in mice after radiation.

**Methods**: BMSC's culture supernatant was subjected to sequential ultracentrifugation at 300g for 10 min, 16,500g for 20 min, and 120,00g for 70 min to obtain sEVs. Enteroids were generated by isolating small intestinal crypts from C57BL/6 mice and seeding them into matrigel for culturing 5–7 days with the supplement of EGF, R‐spondin 1, and noggin. For the in vivo study, C57BL/6 mice were irradiated at 11 Gy with 2.5% bone marrow shielding. On days 1 and 3 after irradiation, vehicle and BMSC‐sEVs (100 μg/mouse) were injected intraperitoneally to the mouse (n = 3‐4/group). On day 4, the intestine was harvested for histologic analyses.

**Results**: An average of 1.6 × 10^10 sEV particles were obtained from 1 million BMSCs, which were < 200 nm in size and positive for CD9 and CD63. When enteroids were irradiated at 5 Gy, there was 34,9% survival after 4 days. However, addition of BMSC‐sEVs at 1, 10, and 100 μg/ml after irradiation increased the survival to 46.3%, 80.4%, and 76.8%, respectively (p < 0.001, unpaired t‐test). In the histologic analysis of intestines in the irradiated mice, BMSC‐sEV treatment significantly increased the numbers of crypts (7.3 vs. 4.7 crypts/mm, p < 0.007), EdU‐positive proliferation cells (2.56 vs. 0.93 crypts/mm, p < 0.001), Olfm4‐positive stem cells (11.8 vs. 6.4 crypts/mm, p < 0.001), and Lysozyme 1‐positive Paneth cells (10.1 vs. 3.5 crypts/mm, p < 0.001) compared to vehicle‐treated mice.

**Summary/Conclusion**: BMSC‐sEVs can rescue the survival of cultured enteroids and improve the intestinal stem cell proliferation and differentiation in the mice after exposure to irradiation. BMSC‐sEVs can be a potential mitigator for treating the radiation‐induced intestine injury.

## OD13 EV Biomarkers

Chair: An Hendrix, Laboratory of Experimental Cancer Research, Department of Human Structure and Repair, Ghent University, Ghent, Belgium

Chair: Jan Lötvall, Krefting Research Centre, Institute of Medicine at Sahlgrenska Academy at the University of Gothenburg, Gothenburg, Sweden.

### OD13.01 PSMA, STIP1 and PPIA Detection in Urine‐Derived Extracellular Vesicles in Multiple Cancers

Lyssa Dimapanat, Columbia University Irving Medical Center

Alex J. Rai, PhD, DABCC, FAACC, Columbia University Irving Medical Center

**Introduction**: Biofluids are a favorable sample type for the identification of novel cancer biomarkers. In particular, urine is convenient, non‐invasively obtained, and available in large sample volumes. It can recapitulate biological information typically obtained from tissue biopsies. We are interested in exploring protein biomarkers in urine extracellular vesicles (EV). Recent studies have revealed the critical role of EV‐mediated intercellular communication and in regulating cancer processes including tumorigenesis and metastasis.

**Methods**: We isolated urine EVs using an optimized workflow based on serial centrifugation. The presence of EVs was confirmed by detection of canonical EV markers (ALIX, flotillin, and ACTN4) using western blotting. Nanoparticle tracking analysis and transmission electron microscopy revealed particle distribution and EV size. We surveyed 117 cancer samples and 23 control samples for the presence of three candidate protein markers identified in our earlier studies: PSMA, STIP and PPIA. Cancer samples were categorized into five different groups: genitourinary (GU), gastrointestinal (GI), brain, lymphomas and leukemias.

**Results**: EVs were isolated from urine samples. PSMA was detected in 62% (73/117) of samples across the various cancer types, and only 2 of 23 controls. Highest PSMA signals were obtained from GU cancers (prostate and bladder), with higher levels in metastatic disease. STIP1 was detectable in 29% (35/117), and PPIA in 5% (6/117) of the cancer samples. STIP1 was detected at highest levels in GI cancers (colorectal and liver), while PPIA was restricted to few samples from patients with higher tumor burden.

**Summary/Conclusion**: Our candidate biomarkers are detectable in urine EVs and can fulfill different unmet clinical needs for patient management. We propose that PSMA has potential application for monitoring therapeutic response in GU cancers, STIP1 may be useful for GI cancer subtyping, and PPIA's restricted detection may define a subset of aggressive cancers.

### OD13.02 Clinical significance of circulating exosomal PD‐L1 in solid and hematological cancers

Valentin Vautrot, CGFL/ INSERM1231

marine Cordonnier, INSERM1231

Charlée Nardin, CHU Besançon

François Aubin, CHU Besançon

Cedric ROSSI, CHU Dijon/ INSERM Equipe Epi2THM

RENE‐OLIVIER casasnovas, CHU Dijon/ INSERM1231

Carmen Garrido, INSERM1231

jessica gobbo, CGFL/ INserm 1231

**Introduction**: Nowadays, immunotherapy is a real therapeutic revolution, especially thanks to inhibitors of the PD‐1/PD‐L1 pathway. Nevertheless, this field still lacks predictive biomarkers. Various circulating markers have been evaluated. Exosomal PD‐L1 is reported to be associated with immunosuppression in various cancers. However, its clinical significance has not been defined yet. We conducted a prospective study to demonstrate the clinical significance of ExoPD‐L1 in cancer patients.

**Methods**: A total of 146 patients with skin cancer (melanoma, squamous cell carcinoma (SCC), and Merkel cell carcinoma (MCC)) and lymphoma were included. Plasma exosomes were isolated by ultracentrifugation and evaluated by nanoparticle tracking analysis (NTA technology) and TEM. Isolated exosomes were tested for the expression of exosomal markers (TSG101, CD9, CD63, Alix). PD‐L1 expression in plasma‐derived exosomes (ExoPD‐L1) was measured using an ELISA.

**Results**: First, ExoPD‐L1 was detected in all patients independently of the type of cancer. In melanoma patients, ExoPD‐L1 was significantly higher than in other skin cancers (MCC, SCC) and lymphoma (respectively 64.26, 37.6, 42.5 and 6.64 pg/mL). Furthermore, we demonstrated that ExoPD‐L1 variations between baseline and after treatment varied over the course of the disease treatment. These variations (decreased level) were correlated with tumor response and survival. Moreover, we showed the capacity of ExoPD‐L1 to differentiate pseudoprogression in patients treated with immunotherapy from a true progression for which, to date in clinical practice no referential exists to guide clinicians.

**Summary/Conclusion**: PD‐L1 in circulating exosome was detected in all patients. Monitoring exosomal PD‐L1 is a potential biomarker of tumor response to immunotherapies and could represent a powerful tool to help clinicians to manage treatments in patients. The ability to capture Exo‐PD‐L1 directly from blood using a microchip requiring only a few microliters of blood demands urgent investigation.

### OD13.03 EV‐associated proteins but not DNA are sensitive and specific biomarkers in metastatic breast cancer

Mercedes Tkach, Institut Curie / INSERM U932

Caroline Hego, Institut Curie, Circulating Tumor Biomarkers laboratory, INSERM CIC BT‐1428

Marc Michel, Institut Curie, INSERM U934/CNRS UMR3215, PSL Research University

Jean‐Yves Pierga, Institut Curie, Department of Medical Oncology, Circulating Tumor Biomarkers laboratory, INSERM CIC BT‐1428

François‐Clément Bidard, Institut Curie, Department of Medical Oncology, Circulating Tumor Biomarkers laboratory, INSERM CIC BT‐1428

Clotilde Thery, MD PhD, Institut Curie / INSERM U932

Charlotte Proudhon, Institut Curie

**Introduction**: Detection of cell‐free circulating tumor DNA (ctDNA) and cancer‐specific extracellular vesicles (EVs) in patient blood have been widely explored as non‐invasive biomarkers for cancer detection and disease follow up. However, most of the protocols used to isolate EVs co‐isolate other components and the actual value of EV‐associated markers remain unclear.

**Methods**: To determine the optimal source of clinically‐relevant biomarkers in breast cancer, we assessed the specificity and sensitivity of nucleic acids and surface proteins from various fractions of blood plasma. We applied a size exclusion chromatography procedure to isolate molecules associated to EV‐enriched or EV‐depleted fractions, in comparison to total plasma.

**Results**: Both nuclear and mitochondrial DNA (gDNA and mtDNA) were detected in EV fractions. Tumor‐specific nuclear alleles, targeting known mutations identified from tumor tissues, were detected in EV‐associated gDNA. However, we observed that ctDNA from total plasma shows equal specificity and better sensitivity. In contrast, mtDNA was preferentially enriched in EV fractions. Yet, EV‐associated mtDNA displayed similar levels in healthy and cancer subjects.

Next, using an EV capture test which allows to analyze the presence of 37 protein markers by flow cytometry (MacsPlex), we have identified 3 EV surface proteins which are enriched in metastatic breast cancer. These preliminary results suggest that a small set of these surface molecules could provide a disease signature and could allow enrichment of cancer‐specific vesicles.

**Summary/Conclusion**: Our findings provide evidence that the detection of DNA within total circulating EVs does not provide added value as compared to the whole plasma. However, analysis of a subtype of EV‐associated proteins may reliably identify cancer patients. These non‐invasive biomarkers represent a promising tool for cancer diagnosis and real‐time monitoring of treatment efficacy and our results will impact the development of therapeutic approaches using EVs as targets or biomarkers of cancer.

### OD13.04 Klotho inside uEVs and its potential role as biomarker for kidney physiopathology in single ventricle pediatric patients

Cristina Grange, Department of Medical Sciences, University of Turin

Dario Tomanin, Department of Molecular Biotechnology and Health Sciences, University of Turin

Luca Deorsola, Regina Margherita Pediatric Hospital

Carlo Pace Napoleone, Regina Margherita Pediatric Hospital

Benedetta Bussolati, University of Turin, Department of Molecular Biotechnology and Health Sciences, Italy

**Introduction**: Extracellular vesicles (EVs) from urine are considered a promising biomarker of the overall state of the kidneys. We recently described that uEV also carry Klotho, an anti‐aging hormone released by the kidney with protective activity on the cardiovascular and neurological systems. We here aimed to characterize the uEVs of a population of pediatric patients affected by single ventricle congenital heart disease. In particular, we aimed to evaluate Klotho levels as a potential biomarker of renal damage.

**Methods**: In this study, we isolated uEVs from first morning urine of pediatric patients affect by single ventricle defects undergone to Fontan procedure (n = 15), and from aged match young healthy subjects. We subsequently characterized uEVs, combining the cytofluorimetric analysis of uEV surface markers, obtained with MACSplex Exosome Kit, with the quantification of uEVs‐Klotho measured by ELISA. Moreover, Klotho and classical exosomal and renal markers were confirmed by single‐molecule super resolution microscopy. Renal function was evaluated by creatinine and NGAL levels.

**Results**: We isolated a comparable number of uEVs from pediatric patients and healthy subjects. We observed the presence of renal progenitor markers (SSEA4, CD133, CD24) and of the epithelial marker CD326 in both experimental conditions. However, the expression of renal uEVs markers was decreased in single ventricle patients. Of interest, the quantification of Klotho levels within uEVs revealed the presence of Klotho only in uEVs from healthy subjects, and not in uEVs from single ventricle patients. No differences in renal functional parameters were observed.

**Summary/Conclusion**: The results suggest that in patients affected by single ventricle congenital heart disease, uEV characterization and Klotho expression might predict pre‐clinical damage to the renal tissue. Klotho loss could be involved in the systemic alterations observed in these patients.

### OD13.05 Expression of PD‐L1 on extracellular vesicles in renal cell carcinoma patients

Philip Zeuschner, Department of Urology and Pediatric Urology, Saarland University

Dirk Himbert, Saarland University

Jadzia Sonnleithner, Department of Urology and Pediatric Urology, Saarland University

Greta Jaschkowitz, Department of Urology and Pediatric Urology, Saarland University

Angela Zaccagnino, Department of Urology and Pediatric Urology, Saarland University

Michael Stöckle, Department of Urology and Pediatric Urology, Saarland University

Kerstin Junker, Department of Urology and Pediatric Urology, Saarland University

**Introduction**: The PD‐1/PD‐L1 axis has revolutionized advanced renal cell carcinoma (RCC) therapy. Although the PD‐L1 expression in EVs can have diagnostic, prognostic or predictive potential in various cancers, not much is known about its role in RCC. For the first time, this study examined the PD L1 expression in primary tumor and healthy kidney tissue and their EVs, including the corresponding blood probes.

**Methods**: Nine patient samples with clear cell RCC were analyzed, in two cases, adjacent normal kidney tissue was available. EVs from fresh frozen tissue were isolated by tissue digestion and sequentially centrifugation with a saccharose gradient. In 8/9 patients, EVs from blood plasma were isolated with an EV isolation kit. The PD‐L1 expression was measured by Western Blot and the results were compared with immunohistochemical (IHC) stainings. Quality and quantity of EV isolation was proven by transmission electron microscopy, Nano Tracking Analysis and Western Blot.

**Results**: 8/9 tumors samples were PD L1 positive, but 7/8 had a very weak staining intensity as defined by IHC on tissue sections. All tumors were infiltrated by PD‐L1 positive immune cells and two tumors had very prominent PD‐L1 positive immune cell infiltrates.

From all primary tumor tissues, EVs were successfully isolated. PD‐L1 was detectable in 8/9 tumors and their EVs, but differed between the patients. In 5/9 cases, PD‐L1 was enriched in tumor EVs compared to the tumor tissue. In 3/9 cases, PD‐L1 was almost not detectable in primary tumor tissues, but highly enriched in the corresponding EVs. The PD‐L1 expression was lower in adjacent normal kidney tissues and their EVs. One patient had PD‐L1 positive tumor EVs in Western Blot, but was negative on IHC.

All patients had PD‐L1 positive EVs in plasma samples. PD‐L1 was enriched in the EVs compared to whole plasma samples in 7/8 patients. There was no clear association with the IHC staining.

**Summary/Conclusion**: For the first time, the PD‐L1 expression in EVs from primary RCC tissues was analyzed. PD‐L1 was enriched in the EVs compared to tumor cells, and was higher in the plasma EVs compared to soluble PD‐L1. Hence, the PD L1 status on EVs could have a higher a diagnostic, prognostic or predictive power in RCC than previously thought, which will further be elucidated in this ongoing study.

### OD13.06 Extracellular Vesicle Subtypes Enable Precision Cancer Diagnosis

MEI HE, University of Florida

**Introduction**: Although extracellular vesicles (EVs) are emerging biomarker sources for developing liquid biopsy‐based cancer diagnosis, the large heterogeneity and significant size overlap between vesicle populations pose extraordinary challenges in developing the clinical utility. Defining EV subtypes could particularly improve the precision for cancer diagnosis. However, currently there is no standardized purification methods yet for obtaining pure EV subtypes that are specific to their cellular origin and molecular information.

**Methods**: Herein, we introduced a novel nanographene‐based immunomagnetic bead with unique 3D nano‐wing structures, which allows the specific marker‐defined capture and release of intact EV subtypes from nearly all types of biological fluids, including human blood, urine, cow milk, and cell culture medium, etc. The nanographene sheet layer with surface conjugated immune photo‐click chemistry enables on demand release of intact, captured EVs for ensuring subtype specificity and improving the sensitivity in downstream analysis.

**Results**: We demonstrated such isolated EV subtypes utilized in diagnosing bladder cancer via detecting DNA mutations and miRNA profiles from urinary EVs. Via the next generation sequencing (NGS) analysis, the specific mutations such as the KRAS, PIK3CA, and ERBB2 are abundantly identified from bladder cancer patient in comparison to healthy control group. The droplet digital PCR analysis also identified heterozygous mutation point in EGFR from bladder cancer patient derived urinary EV subtypes and validated by sanger sequence analysis. Compared to ultracentrifugation approach which isolates a mixture of vesicle populations, the purified EV subtypes defined by their surface markers exhibit much better specificity and sensitivity to detect bladder cancer tumor associated biomarkers.

**Summary/Conclusion**: The developed Nano‐Wing EV preparation approach can offer much purer EV subtypes with good specificity to pathogenesis for developing non‐invasive, liquid biopsy diagnosis of bladder cancer.

### OD13.07 Integrated analysis of sequenced miRNAs from circulating extracellular vesicles and spatial profiling of epithelial ovarian cancer

Priyakshi Kalita‐de Croft, University of Queensland Centre for Clinical Research

Dominic Guanzon, PhD, Exosome Biology Laboratory, Centre for Clinical Diagnostics, UQ Centre for Clinical Research, The University of Queensland, Australia

Shayna Sharma, Exosome Biology Laboratory, UQ Centre for Clinical Research, Royal Brisbane and Women's Hospital, The University of Queensland, St Lucia, QLD, Australia

Margaret Cummings, Pathology Queensland, Royal Brisbane and Women's Hospital, Herston, QLD, Australia

Terry K. Morgan, MD, PHD, Oregon Health and Science University

Lewis Perrin, Mater Research Institute, University of Queensland, Translational Research Institute, Woolloongabba, QLD, Australia

John Hooper, PhD, Mater Research Institute

Kenneth O'Byrne, Queensland University of Technology, Centre for Genomics and Personalized Health, School of Biomedical Sciences, Faculty of Health, Woolloongabba, QLD, Australia

Sunil Lakhani, UQ Centre for Clinical Research, Royal Brisbane and Women's Hospital, The University of Queensland, St Lucia, QLD, Australia

Arutha Kulasinghe,Queensland University of Technology

Carlos Salomon, MSc, DMedSc, PhD, The University of Queensland

**Introduction**: A significant proportion of patients with epithelial ovarian cancer (EOC) often present with advanced stage disease, where treatment options are limited. Recent evidence suggests that miRNAs associated with small extracellular vesicles (sEVs) participate in the progression of EOC. Moreover, these sEV cargos may reprogram the tumour microenvironment (TME). Here, we studied the miRNA content of circulating sEVs in patients with EOC, and we integrated this data with spatial information of the tumour microenvironment.

**Methods**: Informed consent was obtained and approved by the human research ethics committee of the University of Queensland. sEVs were isolated and characterized from plasma of 48 patients with different clinical outcomes (recurrence, deceased and disease‐free). sEV‐associated miRNAs were then sequenced and specific miRNAs were validated using qPCR. Nanostring GeoMX Digital Spatial Profiler (DSP) platform was used to ascertain the TME contexture on formalin‐fixed EOCs for protein markers of various immune and tumour‐related modules. This was integrated with the sequencing results using Ingenuity Pathway Analysis.

**Results**: A total of 1218 sEV‐associated miRNAs were identified, out of which 49 were significantly different in disease‐free vs recurrence. Recurrent patients had a >20‐fold increase in miRNAs: miR‐3202‐1, miR‐3202‐2, miR‐4516, miR‐139‐5p, and miR‐6865‐5p. In deceased patients a 17‐fold increase in miR‐4740‐3p, and a 5‐fold increase in miR‐106b‐3p and miR‐144‐3p was observed compared to disease‐free and recurrence, respectively. Spatial profiling revealed 54 differentially expressed proteins between the tumour and its microenvironment. Within EOC, 32 proteins were dysregulated in deceased compared to disease‐free. In addition, the recurrent group displayed 26 proteins as highly expressed compared to the deceased group. Namely B7‐H3, Beta‐2‐microglobulin, CD14, CD34, CD44, and CD45RO were found to be most dysregulated in the TME. We also obtained a regulatory network of 10 miRNAs that could be modulating the expression of the proteins associated with ovarian cancer progression

**Summary/Conclusion**: We propose that sEVs present in the circulation of EOC patients transfer oncogenic miRNAs to cells within the TME to promote cancer progression

### OD13.08 Integrating Machine Learning with exoRNA for Early Cancer Diagnosis

Tzu‐Yi Chen, Icahn School of Medicine at Mount Sinai, Department of Department of Genetics and Genomic Sciences

Edgar Gonzalez‐Kozlova, Department of Genetics and Genomics Sciences, Icahn School of Medicine at Mount Sinai

Taliah Soleymani, Department of Genetics and Genomics Sciences, Icahn School of Medicine at Mount Sinai

Susmita Sahoo, SS, Department of Cardiology, Icahn School of Medicine at Mount Sinai

Ash Tewari, Department of Urology, Icahn School of Medicine at Mount Sinai

Gustavo Stolovitzky, IBM T. J. Watson Research Center

Carlos Cordon‐Cardo, Department of Oncology Sciences and Pathology, Icahn School of Medicine at Mount Sinai

Navneet Dogra, PhD, Department of Genetics and Genomics Sciences, Icahn School of Medicine at Mount Sinai

**Introduction**: One of the greatest challenges in urology is not how to treat prostate cancer (PCa), but rather, whether to treat it at all. The current PCa clinical diagnosis tool, serum prostate‐specific antigen, can only suggest abnormality in prostate glands but cannot ascertain cancer. Furthermore, novel RNA‐based technologies are being developed for real‐time monitoring of various diseases, including cancer. However, a machine‐learning model could facilitate efficient disease prediction and diagnosis from large‐scale data. Tumor cell‐derived EVs, including microvesicles and exosomes, have sparked great interest in their potential as a liquid biopsy‐based, non‐invasive diagnosis, and detection method for cancer. Despite the tremendous clinical outlook of tumor‐derived EVs, we have yet to fully uncover their transcriptomic lineages before and after tumor resection.

**Methods**: EVs were isolated from aggressive PCa serum samples at the time of proctectomy and from post‐prostatectomy subjects with undetectable cancer. This was followed by quality assessment via electron microscopy, immunoblotting, nanoview, TRPS, nanoparticle tracking, and qPCR. Genome‐wide small exoRNA sequencing was performed to identify unique small RNA markers specific to tumor EVs each before and after tumor‐resection. Finally, 15 exoRNA signatures for PCa were successfully identified and implemented for machine learning model training and validation in independent cohorts.

**Results**: We have established ∼60 total small RNA‐sequencing profiles, and hundreds of qPCR from 17 aggressive prostate cancer (PCa) patient's tumor and adjacent normal tissue, and EVs isolated from serum, and cancer cell culture media. The 15 candidate exoRNA signatures are significantly upregulated in tumor EVs of PCa patients in contrast to controls with no detectable cancer. Novel small RNA biomarkers were orthogonally validated for their differential expression in the ‘biomarker discovery’ cohort using RT‐qPCR. Therefore, our ongoing work is to identify cancer specificity and sensitivity for PCa detection from cohorts and scaling it up to big data solutions.

**Summary/Conclusion**: We have established 15 exoRNA biomarkers to distinguish cancer from non‐cancer patients. Our ongoing work is to train and validate the selected cancer‐specific exoRNA in a large cohort of patients, leading to a minimally‐invasive, blood‐only, operator‐independent surveillance biomarker.

## OD14 EVs in Cancer Pathogenesis II

Chair: Emanuele Cocucci, College of Pharmacy, The Ohio State University, United States

### OD14.01 VEGF binding to HSPG on the surface of extracellular vesicles secreted by carcinoma‐associated fibroblasts promotes angiogenesis in a bevacizumab‐resistant manner

Jiao Li, Department of Orthodontics, Shanghai Stomatological Hospital, Fudan University, Shanghai, China

Xue Liu, Department of Basic Science of Stomatology, Shanghai Stomatological Hospital, Fudan University, Shanghai, China

Tingjiao Liu, Department of Basic Science of Stomatology, Shanghai Stomatological Hospital, Fudan University, Shanghai, China

**Introduction**: The blood vessel growth inhibitor bevacizumab targets vascular endothelial growth factor (VEGF), a crucial regulator of angiogenesis. Recently, small extracellular vesicles (sEVs) have been demonstrated to be important vehicles in the transport of growth factors to target cells. It attracts researchers’ attention whether VEGF can bind to EVs. In this study, we investigated the role of EV‐bound VEGF secreted by carcinoma‐associated fibroblast (CAF) on angiogenesis and its sensitivity to bevacizumab.

**Methods**: Four primary CAFs were isolated from human oral squamous cell carcinoma (OSCC) tissues. sEVs were separated from the conditioned medium of four CAFs, respectively. Tube formation of human umbilical vein endothelial cells (HUVEC) and Matrigel plug assay were performed to evaluate angiogenesis in vitro and in vivo.

**Results**: Compared with other non‐extracellular vesicle components, CAF‐derived sEVs were found to be the main regulators of angiogenesis. The ability of CAF sEVs to activate VEGF receptor 2 (VEGFR2) signaling in HUVEC was dependent on the association between sEVs and VEGF. In addition, sEV‐bound VEGF secreted by CAFs further activated VEGFR2 signaling in HUVEC in a bevacizumab‐resistant manner. We also found that CAF sEVs may stimulate angiogenesis via interaction with the receptors on endothelial cells independently of uptake. Besides VEGF was found to interact with heparan sulfate proteoglycans (HSPG) on the CAF sEV surface and could be released by heparinase I/III. The bioactivity of the dissociated VEGF was retained in vitro and in vivo and could be neutralized by bevacizumab.

**Summary/Conclusion**: Our data indicated that CAF‐secreted VEGF binds to the heparin chain of HSPG on the sEV surface, which might be responsible for the resistance to Bevacizumab. Our findings that heparinase releases VEGF from sEVs and regains sensitivity to Bevacizumab raise the possibility that combination of bevacizumab and heparinase might improve the anti‐angiogenic therapy effects in patients with high sEV‐VEGF levels.

### OD14.02 Extracellular vesicles shed by follicular lymphoma B cells promote the polarization of bone marrow stromal cell niche

Erwan Dumontet, CHU de Rennes

Céline pangault, CHU de Rennes

David Roulois, INSERM U1236

Matthis Desoteux, INSERM U1242

Simon Leonard, INSERM U1236

Tony Marchand, CHU de Rennes

Maelle Latour, CHU de Rennes

Patricia Legoix, Institut Curie

Damarys Loew, Université de Paris, INSERM U970, Paris Cardiovascular Research Centre, Paris, France

Florent Dingli,Curie Institute, PSL Research University, Laboratoire de Spectrométrie de masse Protéomique, Paris, France

Joelle Dulong, CHU de Rennes

Erwan FlecherCHU de Rennes

Cédric Coulouarn, INSERM U1242

Guillaume cartron,CHU de Montpellier

Thierry Fest,CHU de Rennes

Karin Tarte,CHU de Rennes

**Introduction**: Follicular Lymphoma (FL) originates in the lymph nodes (LN) and infiltrates bone marrow (BM) early in the course of the disease. BM FL B cells are characterized by a lower cytological grade, a decreased proliferation, and a specific phenotypic and subclonal profile. Mesenchymal stromal cells (MSC) obtained from FL BM display a specific gene expression profile (GEP), including enrichment for a lymphoid‐stromal cell signature, and an increased capacity to sustain FL B‐cell growth. However, the mechanisms triggering the formation of the medullar FL permissive stromal niche have not been yet identified.

**Methods**: Extracellular vesicles (EVs) from malignant B cells or human bone marrow plasma were gather by differential ultracentrifugation and were characterized by TRPS, TEM and proteomic analysis. Their ability to modify the GEP of BM‐MSC were analyzed by RNAseq and compared to the TNF/LT stimulation. mRNA upregulation were confirmed by qPCR, verified at protein level by luminex assays and test‐ for cell differentiation on prolonged cultures. EV‐induced signalization patways in BM‐MSC were studied by a sensor cell line, flow cytometry and immunofluorescence by confocal microcopy. Finally, after comparing the GEP of LN and BM FL B cells by Affymetrix analysis, we used NicheNet algorithm to describe the putative interactome between BM FL B cells and EV‐primed stromal cells.

**Results**: In the current work, we demonstrated that FL B cells produced EVs that could be internalized by BM‐MSC, making them more efficient to support FL B‐cell survival and quiescence. Accordingly, EVs purified from FL BM plasma activated TGF‐b dependent and independent pathways in BM‐MSC, modified their GEP, triggering an upregulation of factors classically associated with hematopoietic stem cell niche, including CXCL12 or angiopoietin‐1. Moreover, we provided the first characterization of BM FL B‐cell GEP, allowing the definition of the landscape of molecular interactions they could engage with EV‐primed BM‐MSC.

**Summary/Conclusion**: This work identified FL‐derived EVs as putative mediators of BM stroma polarization and supported further investigation of their clinical interest for targeting the crosstalk between BM‐MSC and malignant B cells.

OD14 EVs in Cancer Pathogenesis II

### OD14.03 RAB5a‐GTPase regulates prostate tumor growth and progression via targeting exosome biogenesis and cargo loading in hypoxic microenvironment

Ashish Kumar, Wake Forest Baptist Health

Susy Kim, Wake Forest Baptist MEdical Center

Mitu Sharma, Wake Forest Baptist Medical Center, Department of Cancer Biology

Pawan Kumar, Wake Forest Baptist Medical Center, Department of Cancer Biolo

Yixin Su, Wake Forest School of Medicine

Sangeeta Singh, Wake Forest Baptist Medical Center, Department of Cancer Biology

Gagan Deep, PhD, Wake Forest School of Medicine

**Introduction**: RAB5a‐GTPase is a known regulator of early endosome formation, and here we assessed its role in exosome biogenesis, prostate cancer growth and progression under normoxic and hypoxic conditions.

**Methods**: RAB5a knock‐down (KD) was achieved by siRNA or lentivirus. Exosomes concentration and cargos were assessed by nanoparticle tracking analysis, miRNA‐sequencing and mass spectrometry analysis. To mimic hypoxic environment cells were cultured under 1% O2. Effect of RAB5a KD was assessed on prostate cancer (PCa) cells and in vivo in nude and syngeneic mice models.

**Results**: Hypoxia promoted exosomes secretion by PCa cells while RAB5a knock‐down significantly reduced exosomes release, cell growth, migration and invasion. Orthotropic administration of RBA5a‐KD PC3 cells in the prostate of male nude mice showed both reduced primary tumor growth and micro‐metastasis to distant organs compared to vector control cells. Supplementing exosomes derived from PC3 cells under normoxia and hypoxia conditions restored the growth of RBA5a‐KD PC3 tumors; more strongly with hypoxic exosomes, confirming exosomes‐mediated regulation of tumor growth and metastasis. Moreover, in a syngeneic mouse model, injection of lentiviral particles with RAB5a‐shRNA in mice with established RM1 tumors reduced the tumor growth. Mass spectrometry and miRNA‐seq analyses revealed differential exosomal cargo loading under normoxia and hypoxia following RAB5a KD, with changes mainly associated with tumor growth and cancer progression. Notably, stable RAB5a KD was associated with higher carbonic anhydrase (CA9) expression; and simultaneous CA9 inhibition by specific siRNAs or chemical inhibitor, U‐104, almost completely inhibited PCa cells growth.

**Summary/Conclusion**: Present study demonstrate the critical role of RAB5a‐GTPase in the regulation of exosome biogenesis and cargo loading under both normoxia and hypoxia, and suggest RAB5a as a potential therapeutic target for the treatment of advanced PCa.

### OD14.04 Stromal Heat Shock Factor 1 promotes gastric cancer via exosome mediated secretion of Thrombospondin 2 and Inhibin Subunit Beta A

Meirav Pevsner‐Fischer, Department of Bimolecular Sciences, The Weizmann Institute of Science, Rehovot, Israel

Nil Grunberg, Department of Biomolecular Sciences, The Weizmann Institute of Science, Rehovot, Israel

Tal Goshen‐Lago, Division of Oncology, Rambam Health Care Campus, Haifa, Israel

Judith Diment, Department of Pathology, Kaplan medical center. Rehovot, Israel

Yaniv Stein, Department of Biomolecular Sciences, The Weizmann Institute of Science, Rehovot, Israel

Hagar Lavon, Department of Biomolecular Sciences, The Weizmann Institute of Science, Rehovot, Israel

Yifat Ofir‐Birin, Wiezmann institute for science

Li‐Jyun Syu, Department of Dermatology, Rogel Cancer Center, University of Michigan, Ann Arbor, MI, USA

Salomon M Stemmer, Institute of Oncology, Davidoff Cancer Center, Rabin Medical Center, Beilinson Hospital, Petah Tikva, Israel

Baruch Brenner,Institute of Oncology, Davidoff Cancer Center, Rabin Medical Center, Beilinson Hospital, Petah Tikva, Israel

Andrzej A. Dlugosz, Department of Dermatology, Rogel Cancer Center, University of Michigan, Ann Arbor, MI, USA

David LydenWeill Cornell Medicine

Neta Regev‐Rudzki, Department of Biomolecular Sciences, The Weizmann Institute of Science, Rehovot, Israel

Irit Ben‐Aharon,Division of Oncology, Rambam Health Care Campus, Haifa, Israel

Ruth Scherz‐Shouval,Department of Biomolecular Sciences, The Weizmann Institute of Science, Rehovot, Israel

**Introduction**: Gastric cancer is the 3rd most lethal cancer worldwide, accounting for 3% to 10% of all cancer‐related deaths. The mutational landscape of gastric cancer cells has not translated into effective prognostic or therapeutic strategies, and the standard of care is surgical intervention combined with chemotherapy. We hypothesized that outcomes may depend on the tumor microenvironment (TME), and in particular cancer‐associated fibroblasts (CAFs). However, very little is known about the role of CAFs in gastric cancer.

**Methods**: We mapped the transcriptional landscape of human gastric cancer stroma by laser capture microdissection and RNA sequencing of CAFs from gastric cancer patients to identify signature genes associated with aggressive gastric cancer phenotype. EVs were isolated from Hsf1 null fibroblasts and used in mouse models and cell co‐cultures, to explore their functions.

**Results**: We discovered a stromal gene signature associated with aggressive disease and regulated by the transcription factor Heat Shock Factor 1 (HSF1). Among signature genes, we identified Inhibin Subunit Beta A (INHBA) and Thrombospondin 2 (THBS2) to be regulated by the HSF1and to be delivered to cancer cells via EVs to support cancer growth.

Using mouse models and cell co‐cultures, we found that EVs from Hsf1 null fibroblasts showed similar size, quantity, biogenesis and uptake into cancer cells as their WT counterparts, however their protein content was different. Specifically, INHBA and THBS2 were more abundant in WT versus Hsf1 null MEFs EVs. Furthermore, co‐injection of cancer cells with EVs derived from WT fibroblasts caused a significant increase in the growth of MC38‐injected tumors, and this effect was completely abolished when EVs from Hsf1 null MEFs were co‐injected with MC38 cells.

**Summary/Conclusion**: These findings suggest that HSF1 regulates the expression of INHBA and THBS1/2 in stromal cells. INHBA and THBS2 are then packaged into EVs in a HSF1‐dependent manner and secreted to the TME, where they are taken up by cancer cells and promote a more aggressive disease phenotype.

*Grunberg et al. Cancer‐associated fibroblasts promote aggressive gastric cancer phenotypes via heat shock factor 1‐mediated secretion of extracellular vesicles. Cancer Reseach 2021 Feb 5;canres.2756.2020. https://doi.org/10.1158/0008‐5472.CAN‐20‐2756. Online ahead of print.

### OD14.05 IMP1 Enhances Extracellular Vesicle Secretion in a Transformation‐Dependent Manner

Sarah Andres, PhD, OHSU

Madeline Kuhn, OHSU

Ranjan Preet, University of Kansas Medical Center

Aurora Blucher, OHSU

John Favate, Rutgers University

Sukanya Das, Rutgers University

Shun Liang, Rutgers University

Louis R. Parham, University of Pennsylvania Perelman School of Medicine

Priya Chatterji, University of Pennsylvania Perelman School of Medicine

Kathy N. Williams,University of Pennsylvania Perelman School of Medicine

Sudheer Molugu, University of Pennsylvania Perelman School of Medicine

Wei ZhangUniversity of Pennsylvania

Jiegang Yang, University of Pennsylvania

Kathryn E. Hamilton,Children's Hospital of Philadelphia

Premal Shah,Rutgers University

Gordon Mills,OHSU

Dan A. Dixon,University of Kansas

Anil K. Rustgi,Herbert Irving Comprehensive Cancer Center, Columbia University

**Introduction**: The endosomal, extracellular vesicle (EV), and autophagy pathways are interrelated and influenced by their environment and cellular context. The RNA binding protein insulin‐like growth factor 2 mRNA binding protein 1 (IGF2BP1/IMP1) displays tissue‐specific roles in colorectal cancer (CRC) and can regulate autophagy following damage; however, a role for IMP1 in coordinating the endosomal, EV, and autophagy pathways in the gastrointestinal (GI) tract is not known.

Hypothesis: We hypothesized that IMP1 is a mechanistic link between the endosome and autophagy pathways in CRC.

**Methods**: Ribosomal profiling and RNA sequencing compared transcript and translational efficiency profiles between IMP1 null and IMP1‐overexpressing CRC cell lines. EVs were assessed by nanoparticle tracking analysis, western blot, and electron microscopy in CRC cells and non‐transformed, mouse intestinal enteroids. Effects of IMP1 on endosome, EV, and autophagy pathways were assessed by electron microscopy, immunofluorescence, and western blot.

**Results**: EV, endocytic, and exosome‐related pathways were the most significant differentially regulated pathways by gene ontogeny analysis of RNA sequencing data in IMP1 null vs IMP1‐overpressing CRC cells (p = 1.78 × 10‐10, 2.43 × 10‐10). We found that IMP1 increased EV secretion in HT‐29 (4483±403 vs 1934±414, p = 0.006) and SW480 (3604±399 vs 2293±464, p = 0.049) CRC cells vs null controls. IMP1 modulated translational efficiency or protein levels of early endosome proteins, including EEA1, but did not alter LC3II or p62 levels. When autophagic flux was inhibited (BafilomycinA1), the ability of IMP1 to enhance EV release was magnified. By contrast, Imp1 overexpression in non‐transformed intestinal enteroids did not enhance EV secretion.

**Summary/Conclusion**: Our novel findings suggest that IMP1 has transformation‐dependent effects on the endosomal pathway and EV secretion. Our findings have implications for the development of novel early detection and therapeutic approaches in CRC where IMP1 is overexpressed.

### OD14.06 Molecular Imaging of the Distinct Characteristics of Exosomes and Microvesicles in a Mouse Breast Cancer Model

Masamitsu Kanada, Ph. D., Michigan State University

Ahmed Zarea, MSU

Gloria Perez, MSU

Benedikt Dolgikh, MSU

Matthew Bernard, MSU

Amelia McGill, MSU

Victoria Toomajian, MSU

Alicia Withrow, MSU

Lukose Thampy, MSU

Michael Bachmann,MSU

**Introduction**: Exosomes are produced as components of multivesicular bodies (MVBs) and are released from cells via the fusion of MVBs with the plasma membrane. Microvesicles (MVs) are formed by the outward budding of the plasma membrane. We have previously characterized the physical properties and the functional transfer of cargo molecules packaged in exosomes and MVs. To further understand the roles of exosomes and MVs in cancer, we developed a new EV reporter system that enables comparative analysis of distinct EV classes both in vitro and in vivo.

**Methods**: A new EV imaging reporter was created using a highly sensitive red‐shifted bioluminescence resonance energy transfer (BRET) protein called red enhanced Nano‐lantern (ReNL; a fusion of tdTomato with NanoLuc) with a palmitoylation (Palm) signal peptide. Since palmitoylated reporter proteins have been presumed to label all EV classes, we assessed their labeling efficiency in the fractions of exosomes and MVs derived from 4T1 murine mammary carcinoma cells stably ex‐pressing PalmReNL. Cellular uptake of the reporter exosomes and MVs by several recipient cell types were analyzed. Furthermore, we used in vivo bioluminescence imaging (BLI) to determine the biodistributions of the reporter exosomes and MVs in healthy and mammary tumor‐bearing mice.

**Results**: (1) Exosomes and MVs carrying PalmReNL exhibited red‐shifted emission spectra upon the addition of a luminescent substrate. (2) Both exosomes and MVs contained PalmReNL. (3) Culturing the reporter exosomes or MVs with various cell types for 2 h showed the highest cellular uptake efficiency in the recipient cells. (4) Both reporter exosomes and MVs administered intravenously into BALB/c mice showed similar biodistributions. Both EV classes preferentially accumulated in the lung, followed by liver, spleen, heart, bone, lymph nodes, and brain. (5) Unexpectedly, 4T1 cell‐derived MVs with PalmReNL significantly suppressed primary tumor growth in the syngeneic mouse breast cancer model, while MVs without the reporter did not affect the tumor growth. (6) 4T1 cell‐derived MVs induced severe pulmonary hemorrhage in the tumor‐bearing mice.

**Summary/Conclusion**: Using PalmReNL, we demonstrated the successful tracking of distinct classes of EVs both in vitro and in vivo. Both reporter exosomes and MVs derived from 4T1 cells showed similar biodistributions and organotropisms in healthy mice. The reporter MVs showed a negative effect on the primary tumor growth, possibly through host immune activation by antigenic PalmReNL. Our data suggest that tumor cell‐derived MVs do not support primary tumor growth but cause pathological changes in healthy lung tissues.

### OD14.07 Aptamers for selective targeting of breast cancer exosomes in early diagnosis and therapy

Carla Lucia Esposito, 1 Istituto di Endocrinologia ed Oncologia Sperimentale, Consiglio Nazionale delle Ricerche (CNR), 80100, Naples, Italy

Cristina Quintavalle, Istituto di Endocrinologia e Oncologia Sperimentale IEOS‐CNR, Via Pansini 5, IT80131, Napoli.

Francesco Ingenito, Department of Molecular Medicine and Medical Biotechnology, University of Naples Federico II, Via Pansini 5, IT80131, Napoli.

Deborah Rotoli, Istituto di Endocrinologia e Oncologia Sperimentale IEOS‐CNR, Via Pansini 5, IT80131, Napoli.

Giuseppina Roscigno, Department of Molecular Medicine and Medical Biotechnology, University of Naples Federico II, Via Pansini 5, IT80131, Napoli.

Silvia Nuzzo, IRCCS SDN SpA, Via Gianturco 113, IT80143, Naples Italy.

Renato Thomas, Mediterranea Cardiocentro, 80100, Naples, Italy

Silvia Catuogno,Istituto di Endocrinologia e Oncologia Sperimentale IEOS‐CNR, Via Pansini 5, IT80131, Napoli.

Vittorio De Franciscis, Istituto di Endocrinologia e Oncologia Sperimentale IEOS‐CNR, Via Pansini 5, IT80131, Napoli.

Gerolama Condorelli, Department of Molecular Medicine and Medical Biotechnology, University of Naples Federico II, Via Pansini 5, IT80131, Napoli.

**Introduction**: Extracellular vesicles named exosomes have attracted growing interest as early diagnostic and prognostic biomarkers and therapeutic targets in several cancers including breast cancer (BC). However, tools to easily and specifically distinguish cancer cell‐derived exosomes are mostly unknown and are required to realize their clinical utility. In this context, nucleic‐acid aptamers are a promising class of three‐dimensional single stranded oligonucleotides that serve as high affinity ligands of disease‐associated proteins.

**Methods**: In order to isolate aptamers specific for BC‐derived exosomes, we developed a novel SELEX strategy by using exosomes purified from primary BC cells as positive selection target and exosomes derived from primary normal epithelial breast cell lines in the negative selection. The individual sequences were isolated, optimized and characterized as tools to specifically recognize BC exosomes and to alter exosome uptake.

**Results**: We isolated nuclease resistant RNA aptamers able to specifically discriminate BC‐derived exosomes from those produced by normal cells. The best sequences were optimized identifying short molecules (about 30–35 mer) that resulted effective in specifically detect BC exosomes. Further, they inhibited exosome cellular uptake antagonizing cancer exosome‐induced cell migration

**Summary/Conclusion**: We developed a SELEX strategy with wide applicability to exosome targeting in different tumor types and/or clinical problems. The isolated aptamers show great potential as tools for BC exosome targeting in early diagnosis and therapies. The identification of the aptamer targets is on‐going and can allow to find out new specific markers of BC exosomes.

## OD15 EVs of Non‐mammalian Organisms

Chair 1: Cherie Blenkiron, University of Auckland, New Zealand

Chair 2: Yong Song Gho, Department of Life Sciences, POSTECH, Republic of Korea

### OD15.01 Environmental Plasticity Of Staphylococcus aureus Extracellular Vesicles RNA Content

Brenda Silva Rosa da Luz, INRAE, Institut Agro, STLO, F‐35000, Rennes, France

Aurélie Nicolas, INRAE, Institut Agro, STLO, F‐35000, Rennes, France

Svetlana Chabelskaya, University of Rennes, Inserm, BRM [Bacterial Regulatory RNAs and Medicine] UMR_S 1230, Rennes, France

Vinícius Rodovalho, Laboratory of Cellular and Molecular Genetics, Institute of Biological Sciences, Federal University of Minas Gerais, Belo Horizonte, Brazil

Yves Le Loir, INRAE, Institut Agro, STLO, F‐35000, Rennes, France

Vasco Azevedo, Laboratory of Cellular and Molecular Genetics, Institute of Biological Sciences, Federal University of Minas Gerais, Belo Horizonte, Brazil

Brice Felden, University of Rennes, Inserm, BRM [Bacterial Regulatory RNAs and Medicine] UMR_S 1230, Rennes, France

Eric Guédon, INRAe, Institut Agro, STLO, Rennes, France

**Introduction**: Bacterial extracellular vesicles (EVs) carry various macromolecules able to affect host‐pathogen interactions, such as RNAs. Staphylococcus aureus, an important human and animal pathogen, releases EVs whose RNA content is still unkown. Here, we adress what classes of RNAs compose S. aureus EVs.

**Methods**: S. aureus strain HG003 was cultured in Brain Heart Infusion medium under different in vitro conditions: early‐ and late‐stationary phases, in the presence or absence of a sublethal concentration of vancomycin (0.5 μg/mL). EVs were purified from cell‐free culture supernatants using density gradient ultracentrifugation. Bacterial and EV samples were submitted to phenol‐chloroform RNA extraction, DNAse treatment, and library preparation (Ovation Prokaryotic RNA‐Seq, Nugen, rRNA depletion). Sequencing was performed using Illumina, NextSeq500, 75 cycles, single reading, High Output.

**Results**: Particle yields were similar between conditions, however, EVs from late‐stationary phases were ∼55% larger. On average, 78.0% of HG003 annotated genes were identified in EVs, while only ∼5% presented ‐ 90% read coverage. Highly covered EV RNAs included mRNAs coding for virulence‐factors (hld, agrBCD, psmB1, sbi, spa, isaB), ribosomal proteins, transcriptional regulators, and metabolic enzymes. sRNAs were also detected, including the bona fide rsaC. Interestingly, several of these RNAs were shown to belong to the same transcriptional units in S. aureus. Both nature and abundance of the RNAs in EVs were dramatically affected by growth conditions, whereas much less in the parent cells. Finally, the RNA abundance pattern differed between EVs and parent cells.

**Summary/Conclusion**: To our knowledge, this is the first work characterizing the RNA cargo of S. aureus EVs. Our findings show that EV RNAs are shaped by the environment, and suggest the selective packaging of RNAs into EVs. Finally, this study also shedds light to the possible roles of potentially functional RNAs in S. aureus EVs, notably in host‐pathogen interactions.

### OD15.02 From root to fruit: tomato EVs

Ramesh Bokka, EVs&MS Laboratory, Institute of Biosciences and BioResources, National Research Council of Italy

Giorgia Adamo, Institute for Biomedical Research and Innovation, National Research Council of Italy

Alfredo Ambrosone, Department of Pharmacy, University of Salerno

Antonella Bongiovanni, PhD, Institute for Biomedical Research and Innovation, National Research Council of Italy

Darja Božič, Faculty of Health Sciences, Laboratory of Clinical Biophysics, University of Ljubljana

Monica De Palma, Institute of Biosciences and BioResources, Research Division Portici, National Research Council

Immacolata Fiume, EVs&MS Laboratory, Institute of Biosciences and BioResources, National Research Council of Italy

Michele Guescini, University of Urbino Carlo Bo

Matej Hočevar, Department of Physics and Chemistry of Materials, Institute of Metals and Technology

Matic Kisovec,Department of Molecular Biology and Nanobiotechnology, National Institute of Chemistry

Veronika Kralj‐Iglič, Laboratory of Clinical Biophysics, Faculty of Health Sciences, University of Ljubljana

Mauro MannoInstitute of Biophysics, National Research Council of Italy

Ramila Mammadova, EVs&MS Laboratory, Institute of Biosciences and BioResources, National Research Council of Italy

Samuele Raccosta,Institute of Biophysics, National Research Council of Italy

Michelina Ruocco,Institute for Sustainable Plant Protection, National Research Council of Italy

Simon Sugar,MS Proteomics Research Group, Hungarian Academy of Sciences, Research Centre for Natural Sciences

Marina Tucci,Institute of Biosciences and BioResources, Research Division Portici, National Research Council

Lilla Turiák,MS Proteomics Research Group, Hungarian Academy of Sciences, Research Centre for Natural Sciences

Marjetka PodobnikNational Institute of Chemistry, Department of Molecular Biology and Nanobiotechnology

Gabriella Pocsfalvi, EVs&MS Laboratory, Institute of Biosciences and BioResources, National Research Council of Italy

**Introduction**: Similar to a mammalian cell, intra and extracellular vesicles of a plant cell are involved in many physiological processes, including transport, unconventional protein secretion, defense and symbiosis. There are several studies published on the successful isolation and characterization of apoplastic vesicles. Moreover, EV‐like structures from edible plants are under intense investigation because of their utility as delivery vectors in nutra‐cosmeceutical applications. The presentation will focus on the comparison of different vesicle isolates from three different parts of tomato (Solanum lycopersicum L.) plant: root, leaf and fruit.

**Methods**: Vesicles were isolated by differential ultracentrifugation method from hydroponic washing fluid, leaf apoplastic washing fluid and tomato fruit juice. Isolates from the fruit were further separated by gradient ultracentrifugation or purified by size exclusion chromatography. Physical, molecular and biological characterization was performed with established methods to determine size distribution, concentration, density and morphological characteristics. Proteomics, metabolomics, lipidomics and in vitro bioassays were performed for biocargo analysis.

**Results**: Vesicles isolates from root, leaf and fruit showed different physiochemical characteristics and protein profiles. Root‐derived EVs express a set of proteins associated to biotic stress signaling and plant defense mechanisms. Moreover, they were active against fungal pathogens in vitro. The most abundant proteins identified in tomato apoplastic vesicles were key enzymes of the nitrogen and carbohydrate metabolism, photosynthesis and ripening. Tomato fruit‐derived nanovesicles are complex mixture of intra‐ and extra cellular vesicles. Top‐ranking proteins in the nanovesicles isolated from the fruit include lypoxigenase involved in plant growth and development, several proteins related to ripening and defense mechanisms and to chromoplast differentiation.

**Summary/Conclusion**: Tomato is the most highly consumed vegetable in the word, particularly important for the Mediterranean diet because it is the major dietary source of the antioxidant lycopene. The high yield of tomato fruit‐derived nanovesicles and their anti‐inflammatory effects makes them promising for future innovative applications. The comparative biocargo analysis helps to deepen our understanding on the roles of the vesicles in different organs of a plant.

### OD15.03 Extracellular vesicles released by Mycobacterium tuberculosis: Lipid composition and their interactions with macrophages

Pierre Boyer, Institut de Pharmacologie et de Biologie Structurale, Université de Toulouse, CNRS, Université Paul Sabatier

Jérôme Nigou, CNRS

Emilie Layre, Institut de Pharmacologie et de Biologie Structurale, Université de Toulouse, CNRS, Université Paul Sabatier

**Introduction**: Tuberculosis is one of the top ten causes of death worldwide. M. tuberculosis (Mtb) is an intracellular pathogen of alveolar macrophages. In most cases, Mtb infection of macrophages is followed by a fine equilibrium between the bacillus mechanisms of evasion and host immune responses, leading to the formation of a granuloma that controls the bacillus dissemination but not its eradication. Understanding the molecular crosstalk that conditions this equilibrium is key to develop new anti‐TB tools. In this context, vesicles released by the bacillus, which shuttle bacterial factors between cells, are of key interest because they have the potential to significantly modulate the microenvironment at the site of infection. However, still in its early stages, the characterization of mycobacteria membrane vesicles composition and properties on macrophages, key actors of innate immunity, remain incomplete.

**Methods**: An optimized multi‐step purification of mycobacterial vesicles has been required prior to their molecular and functional characterization. The content of the purified vesicles in immunomodulatory lipidic components was comprehensively characterized using global mass spectrometry approach and by western blot. Their uptake by and properties on macrophages were characterized by combining the use of reporter cell lines, high‐resolution microscopy and in vitro bioassays.

**Results**: Our work has allowed defining the content of purified vesicles in (glyco)lipids and lipoglycans, including virulence‐associated molecules. Our functional studies highlighted that vesicles interact with several PRR, beyond the previously described activation of TLR2, such as with lectins. We also describe their rapid uptake by macrophages, their intracellular trafficking and the subsequent impact on macrophages functionalities involved in the control of Mtb infection, like autophagy and inflammatory response.

**Summary/Conclusion**: The current characterization of mycobacterial vesicles composition and immunomodulatory properties is still incomplete. Our work shows that their contribution in virulence effects and in host immune responses regulation is likely more complex than described so far.

### OD15.04 Evaluation of parasite burden and cytokine production in mice immunized with extracellular vesicles released by Leishmania (Leishmania) amazonensis

Talita V. Vieira Dupin, Universidade Federal de São Paulo campus Diadema

Natasha FC. Ferraz de Campos Reis, Universidade Federal de São Paulo campus Diadema

Elizabeth Cristina Perez, Pós Graduação em Patologia Ambiental e Experimental, Universidade Paulista, São Paulo, Brazil

Rodrigo Pedro Soares, Laboratory of Cellular and Molecular Parasitology, René Rachou Institute, Oswaldo Cruz Foundation, Belo Horizonte, Minas Gerais, Brazil

Ana Claudia Claudia. Torrecilhas, Universidade Federal de São Paulo campus Diadema

Patricia X. Xander, Universidade Federal de São Paulo campus Diadema

**Introduction**: Leishmaniasis is a group of neglected diseases caused by Leishmania. The disease is endemic in 97 countries, with Brazil presenting 90% of the cases reported in the Americas. Studies have already shown that some species of Leishmania are capable of releasing extracellular vesicles (EVs) containing antigens, virulence factors, RNA, DNA and lipids from the parasite. It is known that changes in the cargo of EVs can have an impact on the immune response and disease progression, therefore EVs derived from parasites with different virulence profiles (attenuated and virulent parasites) can present relevant differences in the activation of the immune response. This study aimed to evaluate the modulation of the immune system in mice immunized with EVs of virulent and attenuated L. (L.) amazonensis by analysing the parasite burden and the production of cytokines.

**Methods**: Virulent promastigotes of L. (L.) amazonensis were obtained by consecutive and successive recovery of lesions in infected animals and the attenuated parasites were derived after a long period in culture (100 passages in culture). All EVs were obtained by several centrifugations and used in immunization protocols with 15 days of interval. After 2 or 3 immunizations, animals were challenged with the parasite.

**Results**: We observed a significant decrease in the parasite burden in animals immunized with L. (L.) amazonensis EVs emulsified in Alum adjuvant, compared to the non‐immunized animals and the control group (immunized with Alum adjuvant). Interestingly, more reduction in the parasite burden were observed in animals immunized twice as compared with animals that received 3 doses of EVs. The specific IgG1 and IgG2a levels were analyzed and animals that received 2 or 3 immunizations with EVs showed an increase in both Ig subtypes. The cytokine production analyzed in the supernatant of spleen cells re‐stimulated in vitro with total antigens or EVs showed a Th2 profile with high levels of IL‐10 and IL‐4 in animals immunized with 3 doses of EVs, compared with non‐immunized mice.

**Summary/Conclusion**: Thus, our data suggest a potential protective role for EVs in an experimental immunization model for the prevention of leishmaniasis. Furthermore, it seems that the protective role seems to be related to the better development and polarization of the Th1 response. Further studies involving the dose amount and different types of adjuvants may help to improve the potential of using Leishmania EVs in immunization protocols.

### OD15.05 Extracellular vesicles in Cryptoccocus neoformans: from structural insights to vaccine strategy

Juliana Rizzo, Unité Biologie des ARN des Pathogènes Fongiques, Département de Mycologie, Institut Pasteur

Sarah Sze Wah WONG, Unité Mycologie Moléculaire, CNRS UMR2000, Département de Mycologie, Institut Pasteur

Anastasia D. GAZI, Ultrastructural Bio‐Imaging, UTechS UBI, Département de Biologie cellulaire et infection, UMR 3528 CNRS, Institut Pasteur

Frédérique MOYRAND, Unité Biologie des ARN des Pathogènes Fongiques, Département de Mycologie, Institut Pasteur

Thibault CHAZE, Plateforme Protéomique, Unité de Spectrométrie de Masse pour la Biologie (MSBio), Centre de Ressources et Recherches Technologiques (C2RT), UMR 2000 CNRS, Institut Pasteur

Pierre‐Henri COMMERE, Cytometry and Biomarkers, Centre de Ressources et Recherches Technologiques (C2RT), Institut Pasteur

Sophie NOVAULT, Cytometry and Biomarkers, Centre de Ressources et Recherches Technologiques (C2RT), Institut Pasteur

Mariette MATONDO, Plateforme Protéomique, Unité de Spectrométrie de Masse pour la Biologie (MSBio), Centre de Ressources et Recherches Technologiques (C2RT), UMR 2000 CNRS, Institut Pasteur

Gerard PEHAU‐ARNAUDET, Ultrastructural Bio‐Imaging, UTechS UBI, Département de Biologie cellulaire et infection, UMR 3528 CNRS, Institut Pasteur

Flavia C. G. REIS,Instituto Carlos Chagas, Fundação Oswaldo Cruz (FIOCRUZ)

Matthijn VOS, NanoImaging Core Facility, Centre de Ressources et Recherches Technologiques (C2RT), Institut Pasteur

Lysangela R. ALVESInstituto Carlos Chagas, Fundação Oswaldo Cruz (FIOCRUZ)

Robin C. MAY, Institute of Microbiology and Infection and School of Biosciences, University of Birmingham

Leonardo NIMRICHTER,Instituto de Microbiologia Paulo de Góes (IMPG), Universidade Federal do Rio de Janeiro

Marcio L. RODRIGUES,Instituto Carlos Chagas, Fundação Oswaldo Cruz (FIOCRUZ)

Vishukumar AIMANIANDA,Unité Mycologie Moléculaire, CNRS UMR2000, Département de Mycologie, Institut Pasteur

Guilhem JANBON,Unité Biologie des ARN des Pathogènes Fongiques, Département de Mycologie, Institut Pasteur

**Introduction**: The encapsulated yeast Cryptococcus neoformans is the primary agent of cryptococcal meningitis, resulting in nearly 180,000 deaths worldwide each year. This pathogen has been reported to release EV that might regulate host response during infection; even so, the field still lacks fundamental information regarding EV biogenesis, structure, composition, and function.

**Methods**: Using cutting‐edge methodological approaches including cryogenic electron microscopy (cryo‐EM) and cryogenic electron tomography imaging techniques, proteomics, flow cytometry, and in vivo vaccination model, we provided a robust set of data regarding the structural and compositional aspects of EVs from the C. neoformans and explored their potential use as vaccine platforms.

**Results**: Our analysis suggested a new EV structural model, in which the vesicular lipid bilayer is covered by a mannoprotein‐based fibrillar decoration, bearing the capsule polysaccharide as its outer layer. About 10% of the EV population is devoid of fibrillar decoration, adding another aspect to EV diversity. The analysis of EV structural diversity showed a polymorphic population, composed mostly of regular (round‐bilayered) vesicles (81.4%), but also irregular vesicles (not rounded " bilayer or multilayered). Notably, most of the EV analyzed by cryo‐EM was smaller than 100nm, contrasting with the diameter size values obtained by NTA. By analyzing EV protein cargo from three different cryptococcal species, we provided a list of 17 conserved proteins, most of them predicted to be membrane‐associated proteins, including tetraspanin (Tsh proteins) bearing a SUR7/PalI motif. Additionally, most of the Cryptococcus EVs conserved proteins are known to be protective antigens, suggesting their potential use as a vaccine. Indeed, our data showed that mice immunized with EVs obtained from an acapsular C. neoformans mutant rendered a strong antibody response and significantly prolonged survival of mice upon fungal infection.

**Summary/Conclusion**: This study described a new structure model of cryptococcal EV and identified the first EV protein markers. Additionally, the mice model of infection suggested that cryptococcal EVs can be used as an efficient vaccination strategy. Altogether, the data represent tools for future studies on EV biogenesis, cargo loading, and vaccination development approaches for cryptococcosis and potentially other fungal diseases.

### OD15.06 Set‐up of multi‐parametric analytical assays for quantification, quality control and surrogate potency testing of helminth EVs

Francesca Loria, HansaBioMed Life Sciences OÜ, Mäealuse 2/1, 12618 Tallinn, Estonia

Anne Borup, Department of Clinical Medicine, Faculty of Health, Aarhus University, 8200 Aarhus, Denmark

Anders Toftegaard Boysen, Department of Clinical Medicine, Faculty of Health, Aarhus University, 8200 Aarhus, Denmark

Andres Lõhmus, HansaBioMed Life Sciences OÜ, Mäealuse 2/1, 12618 Tallinn, Estonia

Kadi‐Liis Veiman, HansaBioMed Life Sciences OÜ, Mäealuse 2/1, 12618 Tallinn, Estonia

Paolo Bergese, Dipartimento di Medicina Molecolare e Traslazionale, Università degli Studi di Brescia, 25123 Brescia, Italy

Peter Lindberg Nejsum, Department of Clinical Medicine, Faculty of Health, Aarhus University, 8200 Aarhus, Denmark

Nataša Zarovni, Exosomics / HansaBioMed Life Sciences OÜ

**Introduction**: Extracellular vesicles (EVs) have shown to be key players in parasite‐to‐parasite and parasite‐to‐host cross‐talks and have displayed important immunomodulatory activity. Ascaris suum is a prevalent porcine parasite commonly used to model helminth infection in general and, specifically, A. lumbricoides infection, which to date infects more than one billion people. Our objective was to develop an essential analytical package for A. suum EV quantification and characterization to be employed as a quality control (QC) and/or surrogate potency testing tool throughout EV purification and assessment.

**Methods**: EVs from A. suum incubation media were purified by size exclusion chromatography ‐ only or in combination with ultracentrifugation. EV quantification and characterization were performed by (fluorescence) nanoparticle tracking analysis, protein quantification assay, phospholipid quantification assay, enzymatic activity and fluorometric (lectin‐based) microplate assays.

**Results**: Different A. suum EV batches were analyzed in independent experimental rounds providing multi‐modal quantification, generic or source‐specific content determination, purity and bioactivity evaluation. Our results revealed promising robustness of the used methods, as well as structural (i.e. particle number, content and integrity of EV constituents) and functional parameter (i.e. enzymatic activity and ultimate immunomodulatory effects) intercorrelation. Gathered quantitative and qualitative outputs showed to reflect batch‐to‐batch variability and the variance introduced by the adoption of different methods for EV preparation.

**Summary/Conclusion**: We have implemented several user‐friendly and time‐saving analytical options, combining well‐established and ad‐hoc developed source‐tailored benchtop assays, in line with EV purification that result highly informative in correlating bulk and single‐particle parameters in digital and/or analog mode for comprehensive QC assessment and surrogate functional testing.

### OD15.07 Extracellular vesicle formation in Lactococcus lactis is stimulated by prophage‐encoded holin‐lysin system

Yue Liu, Food Microbiology, Wageningen University

Svetlana Alexeeva, Food Microbiology, Wageningen University

Eline van Ophem, Food Microbiology, Wageningen University

Eddy J. Smid, Food Microbiology, Wageningen University

Tjakko Abee, Food Microbiology, Wageningen University

**Introduction**: Mechanistic insights are lacking on how extracellular vesicles (EVs) are released through the thick cell walls in Gram‐positive bacteria, as the peptidoglycan layer had been historically presumed to be a strong physical barrier preventing such event. In this study, we characterized underlying mechanisms of EV production and provide evidence for a role of prophage activation in EV release using Gram‐positive Lactococcus lactis as a model.

**Methods**: We applied a standard EV isolation procedure to the supernatant of (i) a lysogenic Lactococcus lactis strain FM‐YL11, (ii) its prophage‐cured mutant (“prophi) and (iii) its prophage‐encoded holin‐lysin knock‐out mutant (”holin‐lysin). All strains were cultivated under a non‐stressed condition or a prophage‐inducing condition (addition of mitomycin C). Isolated EVs were quantified by membrane‐specific fluorescent dyes, visualized by electron microscopy and characterized by flow cytometry and proteomics analysis.

**Results**: In FM‐YL11, the prophage‐inducing condition led to an over 10‐fold increase in EV production compared to the non‐stressed condition, whereas EV quantities from the two mutants “prophi and ”holin‐lysin remained at very low level under both conditions. Under prophage‐inducing condition, FM‐YL11 did not show massive cell lysis. Defective tailless phage particles were found to be released in and associated with EVs from FM‐YL11. The proteome profile of prophage holin‐lysin induced EVs has been obtained.

**Summary/Conclusion**: We demonstrated that 1) Gram‐positive L. lactis produces EVs; 2) Stress‐induced prophage activation stimulates EV production; 3) Prophage‐encoded holin‐lysin system mediates massive release of EVs. Findings from this study may provide leads to the discovery of EV producing events and underlying mechanisms of EV release from other Gram‐positive probiotic and pathogenic bacteria with roles in health and disease.

## OD16 EV Uptake

Chair: Deborah Goberdhan, Department of Physiology, Anatomy & Genetics, University of Oxford, United Kingdom

Chair: Vincent Hyenne, INSERM / CNRS, France

### OD16.01 Quantitative characterization of Extracellular Vesicle Uptake and Content Delivery within Mammalian Cells

Émeline Bonsergent, Université de Paris, Inserm, UMR7057/CNRS

Eleonora Grisard, Institut Curie U932

Julian Buchrieser, Virus & Immunity Unit, Department of Virology, Institut Pasteur

Olivier Schwartz, Virus & Immunity Unit, Department of Virology, Institut Pasteur

Clotilde Thery, MD PhD, Institut Curie / INSERM U932

Grégory Lavieu, PhD, Université de Paris, inserm, umr7057/cnrs

**Introduction**: Extracellular Vesicles (EVs) are an emerging vector of communication between tissues and cells and seem to be a promising vector for targeted therapeutic delivery. However much remains to be done at the basic research level to hit translational success.

The delivery process within the acceptor cells need a final EV‐content release that is likely to require fusion between the EV membranes and target membranes of the acceptor cell. Such a fusion reaction is used by viruses and intracellular vesicles to release their content within the target compartments. For EV‐content delivery, we still don't know 1) where the final content mixing occurs within the acceptor cells, 2) how efficient is this process, and 3) what molecules are involved in this final delivery process. We developed a cell‐free assay, which suggested that EV‐content delivery occurs within endosomal compartment though a process that resembles viruses’ delivery.

We aimed here to validate our results within live cells and to acutely quantify this delivery process.

**Methods**: We developed a cell‐based assay, to first identify the entry and delivery points within the acceptor cells and then quantify the content delivery efficiency. Briefly, we engineered donor cells releasing EV‐loaded with luciferase‐ or GFP‐tagged Hsp70, a generic marker of EVs. After EV purification by sequential ultracentrifugation, EVs were incubated with acceptor cells. The fate of the EV content was followed by microscopy (GFP‐tag) or by biochemistry (Luciferase‐tag). Cell fractionation of acceptor cells enabled separation of membrane and cytosolic fractions, which were tested for luciferase activity, to quantify the fraction of EV‐cargo that has been released into the cytosol of the acceptor cells.

**Results**: Confocal imaging colocalization showed that at a fraction of EVs were internalized within endocytic compartment and reached lysosomes. Luciferase‐based assay revealed that 1% of the EVs is associated with acceptor cells after 1h of incubation, and that 30% of this fraction is indeed delivered within the cytosol of acceptor cells. Cytosolic delivery is inhibited when acceptor cells are treated with Bafilomycin, an inhibitor of endosome acidification and maturation. In addition, IFITM proteins, known as inhibitor of viral entry and fusion, decreased EV‐content delivery.

**Summary/Conclusion**: Our results suggest that the EV‐content release occurs at the level of acidic‐endosomes/lysosomes through a process that may require membrane fusion.

### OD16.02 Reporter gene assay for membrane fusion and cytoplasmic cargo delivery of extracellular vesicles

Shun'ichi Kuroda, Osaka University

Masaharu Somiya, Osaka University

**Introduction**: Extracellular vesicles (EVs) are expected to mediate intercellular communication by delivering their cargo to recipient cells. However, the mechanisms of cytoplasmic cargo release remain largely unknown. In this study, we have established a novel reporter gene assay that can quantify the efficiency of EV membrane fusion and subsequent cargo release in recipient cells.

**Methods**: The transcription activator (TA) gene was fused with the EV marker protein tetraspanin gene. Forty‐eight to 96 hours after transfection of 293T cells (donor cells), modified EVs were recovered from the supernatant by PEG precipitation. When modified EVs fuse with the cellular membrane of recipient cells, TA is released into the cells and induces reporter gene expression. The expression of the reporter gene was measured after 24 hours.

**Results**: We confirmed that EVs containing TA‐tetraspanin fusion protein were secreted from donor 293T cells into the supernatant. However, the TA‐containing EVs derived from 293T cells did not induce reporter gene expression in recipient reporter cells. In contrast, EVs containing the viral membrane fusion protein VSV‐G strongly induced reporter gene expression. Mutation of VSV‐G resulted in complete loss of EV‐mediated membrane fusion, suggesting that the assay readout reflects the membrane fusion and cargo release of EVs. Besides, the treatment of drugs that inhibit endocytosis and membrane fusion blocked VSV‐G‐mediated EV entry and fusion. These data indicated that the reporter assay is useful in validating the cargo delivery mechanism of EVs.

**Summary/Conclusion**: Membrane fusion is a key event in EV‐mediated cargo delivery. This novel reporter gene assay is useful to decipher the membrane fusion and intercellular signaling of EVs. In the absence of VSV‐G, we couldn't observe membrane fusion in 293T‐EVs and 293T‐recipient cells indicated that unmodified EVs have no or very weak fusion activity. It is worth verifying whether other combinations of donor/recipient cells are more susceptible to EV‐mediated cargo delivery.

### OD16.03 Quantum dot‐based single particle tracking reveals the spatiotemporal dynamics of tumor extracellular vesicle internalization and miRNA‐delivery into endothelial cells

Houfu Xia, School and Hospital of Stomatology,Wuhan University

Zili Yu, School and Hospital of Stomatology, Wuhan University

Lijuan Zhang, College of Chemistry and Molecular Sciences, Wuhan University

Gang Chen, School and Hospital of Stomatology, Wuhan University

**Introduction**: Developing visualization and tracking methods to elucidate the spatiotemporal dynamics of extracellular vesicles’ (EVs) behaviors will facilitate understanding their physiopathological functions and developing therapeutic strategies.

**Methods**: Quantum dots (QDs) were used to label oral cancer EVs (OCEVs) and the carried miRNA respectively based on biotinylation strategy and electroporation method. A universal single particle tracking (SPT) platform was developed based on a spinning disk confocal microscope. The multi‐color and multi‐component labeled OCEVs were co‐cultured with endothelial cells (ECs), and the dynamic interaction between them was visualized and recorded in situ and in real‐time through SPT. The characteristics of the internalization and transportation of EVs by ECs, and the specific steps of subsequent miRNA‐release were analyzed by trajectory reconstruction and motion analysis.

**Results**: By optimizing the culture concentration and period with DSPE‐PEG‐Biotin, we successfully achieved efficient biotinylation of different subtypes of EVs regardless of their size distribution or cell origin. The site‐specific labeling of EVs with QDs was realized through further adjustment of the incubation concentration. Fluorescently labeled miRNA were efficiently loaded into the EVs, achieving simultaneous multi‐color labeling of different components of EVs. ECs efficiently internalized OCEVs through clathrin‐mediated endocytosis in a time‐dependent manner, and subsequently transported them to the perinuclear region through a typical “slow‐fast‐slow” three‐stage pattern. Finally, the internalized EVs rapidly released their miRNA possibly through their interaction with acidic endosomes. In addition, targeting acidification effectively blocked the miRNA‐delivery by EVs, thereby exerting an inhibitory effect on tumor angiogenesis.

**Summary/Conclusion**: This study, for the first time, reported the entire process and detailed dynamics of OCEV transportation and cargo‐release in ECs, leading to better understanding of their pro‐angiogenic functions. Additionally, the QD‐based single particle tracking technique would help uncover more secrets of EV‐mediated cell‐cell communication.

### OD16.04 PDZ networking regulates exosome production, composition, and uptake

Monica Castro‐Cruz, KU Leuven

Raphael Leblanc, Centre de Recherche en Cancérologie de Marseille

Sofie Meeussen, KU Leuven

Lore Van Herck, KU Leuven

Frederique Lembo, Centre de Recherche en Cancérologie de Marseille

Guido David, KU Leuven

Pascale Zimmermann, KU Leuven

**Introduction**: Our poor understanding of the molecular mechanisms governing exosome biogenesis, uptake, and heterogeneity limits the rational use of these nano‐sized extracellular organelles in health and disease. Because tetraspanins and syndecans, major components of exosomes, contain a binding site for PDZ proteins and because PDZ proteins co‐evolved with multicellularity, we hypothesized that PDZ proteins might represent important players in exosome biology.

**Methods**: To test for tetraspanin and syndecan PDZ interactions, we performed a comprehensive yeast two‐hybrid screen (ca. 150 PDZ proteins in the human genome). To collect exosome‐enriched fraction, we used differential ultracentrifugation of conditioned medium from MCF‐7 cells. Exosome number and composition were analyzed by nanoparticle tracking analysis and immunoblotting, respectively. Subcellular protein localizations, syndecan endosomal budding, HS localization at the plasma membrane, and uptake of CD63 positive particles were analyzed by microscopy.

**Results**: We revealed an unsuspected broad number of direct interactions between the PDZ proteome and syndecans, while the number of direct PDZ‐CD9, CD63, and CD81 interactions appears more discrete. We show that PDZ proteins fine‐tune exosome composition and regulate positively or negatively the number of exosomes. We observe that on the contrary to tetraspanins, syndecan c‐terminal fragments are reliable indicators of exosome number. We document that PDZ proteins impact on the steady‐state distribution of syndecans and tetraspanins, and regulate syndecan endosomal budding and processing. Moreover, PDZ proteins impact on the uptake of exosomes and control heparan sulfate levels at the cell surface.

**Summary/Conclusion**: In conclusion, our study establishes that tetraspanin‐ and SDC‐PDZ networking contributes to exosome heterogeneity and turnover, and highlights an important piece of the molecular framework governing intracellular trafficking and intercellular communication.

### OD16.05 Intratumoral cellular heterogeneity as a critical factor for extracellular vesicle uptake in colorectal cancer

Andrea Kelemen, Semmelweis University

Idan Carmi, Semmelweis University, Department of Genetics, Cell‐ and Immunobiology, Molecular Cancer Biology Research Group

Zoltán Wiener, Semmelweis University, Department of Genetics, Cell and Immunobiology, Budapest, Hungary

**Introduction**: Colorectal cancer (CRC) is one of the most common cancers. The functionality of the intratumoral genetic and phenotypic heterogeneity of cancer cells are not yet fully understood. Patient‐derived 3D organoids are one of the most modern methods to study in vitro this heterogeneity. IFITM1 plays a critical role in virus uptake. Since our bioinformatical analysis showed a correlation between IFITM1 expression and patient survival in CRC, we aimed at i) determining the cellular heterogeneity with respect to IFITM1 expression and ii) characterizing the EV uptake of different CRC cell populations.

**Methods**: The Ethics Committee of the Medical Research Council of Hungary approved our experiments and informed consent was obtained from patients. We sorted patient‐derived organoid cells, we measured the ratio of proliferating cells, organoid diameter and analyzed gene expression profiles. We detected EVs by antibody‐coated beads and flow cytometry, NTA, Western‐blotting and TEM. The uptake of labelled large EVs was visualized by confocal microscopy and the functional importance of the uptake was assessed by immunocytochemistry.

**Results**: We observed a higher expression of IFITM1 in adenomas and CRCs than in the normal colon. IFITM1high CRC cell‐derived organoids were larger and they contained more Ki67+ proliferating cells compared to IFITM1low organoids. IFITM1low CRC cells took up more EVs, however, we could not detect this difference for syntetic liposomes. Furthermore, exposure of IFITM1low organoids to fibroblast‐derived EVs resulted in a more pronounced increase in Ki67+ cell number compared to IFITM1high cells, leading to the disappearance of the difference in organoid size.

**Summary/Conclusion**: Our data indicate that intratumoral heterogeneity leads to tumor cell subpopulations with different ability to take up EVs, leading to different proliferation potential. Thus, we propose that intratumoral heterogeneity should be considered as a critical factor when designing targeted EV‐based therapy in CRC.

## OD17 EV‐mediated Pathogenesis and Therapeutics in Cardiovascular Diseases

Chair: J. Bryan Byrd, University of Michigan, United States

### OD17.01 YAP/TAZ facilitates extracellular vesicle release upon mechanical stimulation

Felix Nägele, Department of Cardiac Surgery, Medical University of Innsbruck

René Weiss, Danube University Krems

Tanja Eichhorn, Center for Biomedical Technology, Danube University Krems

Viktoria Weber, Center for Biomedical Technology, Danube University Krems

Johannes Holfeld, Department of Cardiac Surgery, Medical University of Innsbruck

**Introduction**: The physical stimulus of shockwave therapy (SWT) has a pro‐angiogenic impact on ischemic tissue, representing a promising regenerative approach. The Hippo signaling pathway YAP/TAZ plays a key role in angiogenesis and can be regulated by mechanical signals. Both SWT stimulation and YAP/TAZ activation cause a secretory release of extracellular vesicles (EVs). We aim to substantiate the mechanotransduction of SWT via YAP/TAZ facilitated EV release and subsequent angiogenic response.

**Methods**: In order to investigate the detailed underlying mechanisms, human umbilical vein endothelial cells were stimulated with 300 impulses at a frequency of 3 Hz and an energy flux density of 0,1mJ/mm2. Four hours thereafter, mRNA expression of YAP/TAZ target genes (ANKRD1, CYR61) was measured and the nuclear localization of YAP/TAZ was examined by immunofluorescence. The cell culture supernatant was collected. The release of EVs was characterized by flow cytometry to detect bigger EVs using annexin V (Anx5) as a marker of phosphatidylserine (PS) expressing EVs. Furthermore, EVs were analyzed by a bead‐based flow cytometry assay to detect smaller EVs by using CD63‐coupled magnetic beads. The particle concentration was measured by nanoparticle tracking analysis.

**Results**: SWT of HUVECs resulted in a higher concentration of Anx5+ EVs (12,675±2,863 vs.8,650±1614 EVs/μl) in the culture supernatant as compared to the untreated control. This observation was confirmed by a higher percentage of EV‐decorated beads after SWT. This was accompanied by higher mRNA expression of YAP/TAZ target genes ANKRD1 (p = 0.0005, respectively) and CYR61 (p = 0.0006, respectively). Immunofluorescence staining showed a nuclear translocation of YAP/TAZ upon SWT compared to untreated controls. These effects were abolished upon pharmacological inhibition of YAP/TAZ nuclear translocation.

**Summary/Conclusion**: EV release via SWT could represent a regenerative approach for ischemic heart disease.

### OD17.02 Role of extracellular vesicles in the propagation of inflammation and oxidative stress during vascular calcification

Linda Yaker, MP3CV‐UR7517, CURS‐Université de Picardie Jules Verne, Avenue de la Croix Jourdain F‐80054 Amiens, France

Saïd Kamel, MP3CV‐UR7517, CURS‐Université de Picardie Jules Verne, Avenue de la Croix Jourdain F‐80054 Amiens, France

Jérôme Ausseil, INSERM UMR1043, CNRS UMR5282, University of Toulouse III, F‐31024 Toulouse, France

Agnès Boullier, MP3CV‐UR7517, CURS‐Université de Picardie Jules Verne, Avenue de la Croix Jourdain F‐80054 Amiens, France

**Introduction**: Vascular calcification (VC) is a cardiovascular complication in patients with diseases such as atherosclerosis or chronic kidney disease. During VC, vascular smooth muscle cells (VSMCs) undergo an osteogenic switch and secrete a heterogeneous population of extracellular vesicles (EVs). Recent studies have shown the involvement of EVs in inflammation and oxidative stress observed in the VC. The objective of our study is to determine the role and the mechanism of action of EVs in the propagation of inflammation and oxidative stress during the VC process.

**Methods**: Inflammation (IL6, IL1β, TNF‐α, NLRP3), oxidative stress (iNOS, SOD‐1, Nrf2, Keap1), and expression of EVs biogenesis markers (SMPD3, TNAP, phospho‐1) were analyzed by RT‐PCR in a macrophage murine cell line (RAW 264.7) treated with lipopolysaccharide (LPS EK). EVs secreted by these macrophages were then collected by ultracentrifugation and characterized by analyzing the acetylcholinesterase (AChE) activity as well as CD9 and CD81 protein expression by Western blotting. A murine VSMCs cell line (MOVAS‐1) was incubated with these EVs in calcifying conditions (Pi 4 mM " 14 days) and calcification was assessed by the o‐cresolphthalein calcium assay.

**Results**: An increase of oxidative stress and inflammation via the inflammasome activation was observed in LPS‐EK“treated macrophages. The expression of the EVs biogenesis’ markers in these macrophages was significantly decreased as well as the AChE activity in EV‐derived from LPS‐EK” treated macrophages. In calcifying conditions, these EVs significantly increase the calcification of VSMCs. A 24h‐treatment of VSMCs with these EVs induces an inflammatory as well as an oxidative response.

**Summary/Conclusion**: EVs derived from LPS‐EK"treated‐macrophages are themselves able to induce a pro‐inflammatory and pro‐oxidative response in surrounding cells, such as VSMCs, thus aggravating the VC process. These EVs could therefore be a therapeutic target to limit VC in patients.

### OD17.03 miRNAs transferred within extracellular vesicles as key mediators of antifibrotic MSC effects in vitro and in vivo

Nataliya Basalova, Institute for regenerative medicine, Medical Research and Education Center, Lomonosov Moscow State University, Moscow, Russia

Ivan Zaitcev, Faculty of Medicine, Lomonosov Moscow State University, Moscow, Russia

Mikhail Arbatskiy, Faculty of Medicine, Lomonosov Moscow State University, Moscow, Russia

Olga Grigorieva, Institute for regenerative medicine, Medical Research and Education Center, Lomonosov Moscow State University, Moscow, Russia

Maksim Vigovskii, Institute for regenerative medicine, Medical Research and Education Center, Lomonosov Moscow State University, Moscow, Russia

Uliana Dyachkova, Faculty of Medicine, Lomonosov Moscow State University, Moscow, Russia

Anastasiya Tolstoluzhinskaya, Faculty of Biology, Lomonosov Moscow State University, Moscow, Russia

Vladimir Popov, Faculty of Medicine, Lomonosov Moscow State University, Moscow, Russia

Natalia Kalinina, Faculty of Medicine, Lomonosov Moscow State University, Moscow, Russia

Zhanna Akopyan,Medical Research and Education Center, Lomonosov Moscow State University, Moscow, Russia

Anastasia Efimenko, Institute for regenerative medicine, Medical Research and Education Center, Lomonosov Moscow State University, Moscow, Russia

**Introduction**: Applying extracellular vesicles of mesenchymal stromal/stem cells (EV‐MSC) is an effective approach for the treatment of fibrotic diseases. However, the exact mechanism of this effect remains unclear. It is known that the suppression of fibrosis is based on inhibiting the activity of myofibroblasts and stimulating their dedifferentiation in normal tissue‐specific stromal cells. The key molecules regulating these processes are miRNAs, which are transported mainly as a cargo of EVs. Therefore, we investigated the effects of selected miRNAs within EV‐MSC on myofibroblasts dedifferentiation in vitro and in vivo.

**Methods**: EVs were isolated from the conditioned medium of human MSC and characterized by standard methods. Inhibition and overexpression of the studied miRNAs were obtained by EVs transfection with synthetic oligonucleotides. The effects were assessed using in vitro model of dedifferentiation of human myofibroblasts (ICC, western blot, RT‐PCR, collagen contraction test) and in vivo model of bleomycin‐induced pulmonary fibrosis in C57Bl/6 mice.

**Results**: We showed that EV‐MSC stimulated the dedifferentiation of myofibroblasts at the morphological and functional levels. RNA sequencing of EVs revealed more than 50 miRNAs associated with the regulation of myofibroblasts dedifferentiation and fibrosis. We demonstrated that not a single miRNA, but a complex consisting of miRNA‐129 and ‐29c had a critical impact into the observed effects in vitro. Suppression of miRNA‐129 and ‐29c significantly decreased the in vivo antifibrotic effect of EV‐MSC leading to the increased number of activated myofibroblasts and fibrotic foci in the lungs.

**Summary/Conclusion**: A complex of specific miRNAs transferred within EV‐MSC are able to stimulate myofibroblast dedifferentiation in vitro and in vivo, thereby influencing the development of fibrosis. Our findings may provide a basis for a novel antifibrotic EV‐based therapy development.

### OD17.04 Endothelial‐Derived Extracellular Vesicles: A New Paradigm in Cancer Therapy‐Related Cardiac Dysfunction

Crizza Ching, Institute of Medical Science, University of Toronto

Dakota D. Gustafson, Toronto General Hospital Research Institute

Paaladinesh Thavendiranathan, Peter Munk Cardiac Centre, University Health Network

Jason E. Fish, PhD, Toronto General Hospital Research Institute

**Introduction**: Cancer‐therapy related cardiac dysfunction (CTRCD) is a devastating cardiovascular sequalae often associated with anthracycline therapy. The lack of mechanistic understanding remains a barrier to improving cardiovascular prognosis for cancer patients. While most studies have focused primarily on toxicity in cardiomyocytes, the role of the endothelium in CTRCD has remained largely unexplored. Recently, extracellular vesicles (EVs) have been shown to mediate intercellular communication and induce phenotypic changes in recipient cells. As EVs are a component of the endothelial secretome, we aimed to elucidate the effect of cancer treatment on endothelial‐derived EVs.

**Methods**: The extent of endothelial activation and permeability following epirubicin treatment was evaluated through qRT‐PCR and VE‐Cadherin staining, respectively. Endothelial permeability was further assessed in real‐time using the xCELLigence platform. Human umbilical vein endothelial cell (HUVEC)‐derived EVs were isolated by ultracentrifugation following exposure to epirubicin (n = 4). Initially, EVs were characterized by immunoblotting and cryo‐electron microscopy. CytoFLEX was also utilized to further characterize specific EV subsets. Total particle count and mean particle size were determined using nanoparticle tracking analysis. To gauge their effect on endothelial homeostasis, HUVECs were exposed to EVs and permeability was assessed.

**Results**: Treatment of HUVECs with epirubicin led to increased expression of endothelial activation genes and increased permeability, with stronger effects observed at higher doses. Morphological assessment of EVs showed an increase in size and abundance post‐anthracycline treatment (p < 0.05). Changes in EV surface markers were also observed, with increased CD31+ EVs following treatment. Exposure of naïve HUVECs to EVs derived from epirubicin‐treated HUVECs showed increased endothelial permeability immediately after treatment compared to untreated HUVEC‐derived EVs.

**Summary/Conclusion**: Our results demonstrate that anthracycline treatment perturbs endothelial homeostasis, which could lead to the release of effector EVs capable of mediating further endothelial damage. As endothelial‐derived effectors can affect cardiac function, deciphering how these EVs affect cardiomyocyte function could have implications in understanding CTRCD pathogenesis.

### OD17.05 Functional differences of small extracellular vesicle subpopulations

Simonides Immanuel van de van de Wakker, Department of Experimental Cardiology, University Medical Center Utrecht, Utrecht University, The Netherlands

Julia Bauzá‐Martinez, Biomolecular Mass Spectrometry and Proteomics, Bijvoet Center for Biomolecular Research and Utrecht Institute for Pharmaceutical Sciences, Utrecht University, The Netherlands

Carla Rios Arceo, Department of Experimental Cardiology, University Medical Center Utrecht, Utrecht University, The Netherlands

Eduard Willms, Department of Biochemistry and Genetics, La Trobe Institute for Molecular Science, La Trobe University, Melbourne, Australia

Olivier Gerrit G. de Jong, PhD, CDL Research, University Medical Center Utrecht and Department of Pharmaceutics, Utrecht Institute for Pharmaceutical Sciences, (UIPS), Faculty of Science, Utrecht University, The Netherlands

Wei Wu, Biomolecular Mass Spectrometry and Proteomics, Bijvoet Center for Biomolecular Research and Utrecht Institute for Pharmaceutical Sciences, Utrecht University, The Netherlands

Joost P.G. Sluijter, J.P.G., Department of Experimental Cardiology, University Medical Center Utrecht, Utrecht University, The Netherlands

Pieter Vader, CDL Research, University Medical Center Utrecht, The Netherlands

**Introduction**: The use of cardiac progenitor cell (CPC)‐derived small extracellular vesicles (sEVs) has shown potential to stimulate cardiac repair. sEVs are released by cells and play a role in intercellular communication through transfer of their bioactive content. Increasing evidence indicates that sEVs present a heterogeneous population of vesicles. In the context of cardiac regeneration, studying sEV heterogeneity could provide new insights into mechanisms underlying sEV‐mediated cardiac repair properties and help to improve the therapeutic application of CPC‐derived sEVs.

**Methods**: A two‐step size‐exclusion chromatography (SEC) approach was employed to isolate and fractionate different EV‐subfractions derived from CPCs. Western blot analyses were performed on individual fractions to determine protein expression patterns and define sEV subpopulations. Characterization of particle size and number, as well as protein‐, lipid‐ and RNA content of the different sEV subpopulations was performed. Full proteomic composition was studied using mass spectrometry (MS). Functional effects on recipient endothelial cells (ECs) were studied using an ERK/AKT phosphorylation assay, a wound healing assay, and a sprouting assay and on recipient cardiomyocytes using a survival assay.

**Results**: Size‐based fractionation and subsequent characterization of CPC‐derived sEVs revealed that sEVs comprise heterogeneous subpopulations that differ in size and proteomic composition. Based on the differential expression of common EV markers between individual SEC fractions, three distinct sEV subpopulations were identified. MS analysis confirmed the differences in the expression levels of classical EV marker proteins, as well as annexins, rab proteins, integrins, histones and proteasomal proteins. Exposure of recipient cells to sEV subpopulations demonstrated their differential functional effects, including on recipient cell ERK/AKT phosphorylation status and migration properties.

**Summary/Conclusion**: Two‐step SEC allows for the identification of sEV subpopulations and in‐depth study of the functional heterogeneity of sEVs. Increased knowledge of sEV heterogeneity will contribute to a better understanding of the mechanisms of action of sEVs in cardiac regeneration. Usage of specific functional CPC‐derived sEV subpopulations to stimulate cardiac repair will allow an off‐the‐shelf approach to stimulate cardiac regeneration.

### OD17.06 Systemic infusion of regeneration‐associated cells‐derived extracellular vesicles improved ischemia‐injured heart function

Hospital

Ainur Salybekova, MD, Advanced Medical Science, Tokai University School of Medicine

Sheng Ying, MD, PhD, Advanced Medical Science, Tokai University School of Medicine

Yoshiko Shinozaki, Teaching and Research Support Core Center, Tokai University School of Medicine

Keiko Yokoyama, Teaching and Research Support Core Center, Tokai University School of Medicine

Shuzo Kobayashi, MD, PhD, Kidney Disease and Transplant Center, Shonan Kamakura General Hospital

Takayuki Asahara, MD, PhD, Advanced Medical Science, Tokai University School of Medicine

Amankeldi A. Salybekov, MD., Ph.D, Clinical Research Center, Regeneration and Translational Science Department, Shonan Kamakura General Hospital

**Introduction**: In an earlier study, we showed that under vasculogenic conditioning, pro‐inflammatory cell subsets of peripheral blood mononuclear cells (PBMCs) such as macrophages type 1 (M1ɸ), T cells, and primitive EPC cells beneficially shifted their phenotype to pro‐regenerative cells such as vasculogenic EPCs, M2 macrophages, and regulatory T cells, collectively designated as regeneration‐associated cells (RACs). Furthermore, systemic transplantation of the low number of RACs (1 × 105) beneficially improved cardiac functions. Here, we evaluated therapeutic efficacy of RAC‐derived extracellular vesicles (RACev) in comparison with MSC‐derived EVs (MSCev) in the context of rat myocardial ischemia‐reperfusion injury (IRI).

**Methods**: Human PBMCs were cultured with defined growth factors for 7‐days to harvest RACs. RACev and MSCev were isolated via ultracentrifugation. EVs quantity and quality were characterized by NTA, surface protein (CD9, CD63, Alix) expression, and TEM. EVs miR was sequenced and regenerative responsible miRs were validated using TargetScan and miRBase. Allogeneic immune responses to the RACev and MSCev were evaluated. The function of RACev and MSCev were evaluated using HUVECs proliferation and cell‐cycle assays in vitro and repetitive (30min, d1, and d3 after IRI) systemic infusion with either RACev or MSCev in a myocardial IRI model.

**Results**: We observed significant differences in EV‐specific surface markers (CD9 and CD63) expression as well as in quantity of EVs, secreted from RAC than MSC (P>0.03). In vitro assay, RAVev markedly enhanced cell viability, proliferation, and migration of HUVECs in a dose‐dependent manner compared to MSCev. Notably, systemic injection of RAC (5 × 105) derived EVs beneficially ameliorated cardiac functions at four weeks such as fractional shortening (P>0.005), and preserved from mitral regurgitation (P>0.03) than MSCev treated group. In histology, RACev transplanted group showed less interstitial fibrosis and enhanced capillary densities compared to MSCev. These beneficial effects are coupled with significant expression of angiomiRs (miR‐195‐5p, miR‐200b‐5p, miR‐126‐3p/5p) and anti‐fibrosis miR‐133 family in RACev but not in MSCev. In vivo bioluminescence analysis depicted preferential accumulation of RACev into the IR‐injured myocardium (P>0.01) while MSCev is in a modest manner. Immune phenotyping analysis confirmed immunomodulatory effect of MSCev and RACev by inhibiting antigen‐presenting cells to escape from T cell recognition.

**Summary/Conclusion**: Taking together, repetitive systemic transplantation of RACev is superior to MSCev in terms of cardiac function enhancements via crucial angiomiRs, anti‐fibrosis miRs, and anti‐apoptosis miRs delivery to the ischemic tissue.

### OD17.07 Characterisation of Intracardiac Extracellular Vesicles in the Context of Myocardial Infarction and Glucose Intolerance

Stephane M.I. MAZLAN, Paris Cardiovascular Research Centre

Vincent Duval, Université de Paris, INSERM U970, Paris Cardiovascular Research Centre

Cecile Devue, Inserm U970‐ Paris Cardiovascular Research Center

Michael Robillard, Inserm U970‐ Paris Cardiovascular Research Center

Fariza Mezine, Université de Paris, INSERM U970, Paris Cardiovascular Research Centre, Paris, France

Pierre‐Michaël Coly, Université de Paris, INSERM U970, Paris Cardiovascular Research Centre, Paris, France

Shruti Chatterjee, Université de Paris, INSERM U970, Paris Cardiovascular Research Centre, Paris, France

Stephane Camus, Université de Paris, INSERM U970, Paris Cardiovascular Research Centre

Ke Xiao,Fraunhofer Institute for Toxicology and Experimental Medicine (ITEM)

Jan Fiedler, Fraunhofer Institute for Toxicology and Experimental Medicine (ITEM)

Thomas ThumInstitute of Molecular and Translational Therapeutic Strategies (IMTTS), Hannover Medical School & Fraunhofer Institute for Toxicology and Experimental Medicine (ITEM)

Philippe Menasché, Université de Paris, INSERM U970, Paris Cardiovascular Research Centre

Chantal Boulanger,Inserm U970‐ Paris Cardiovascular Research Center

Jean‐Sébastien Silvestre,Université de Paris, INSERM U970, Paris Cardiovascular Research Centre

Xavier LOYER, INSERM U970‐PARCC

**Introduction**: In response to myocardial infarction (MI), extracellular vesicles (EVs), including large (lEVs) and small (sEVs), are released within and from the heart to facilitate intercellular communication and maintain cardiac homeostasis by transferring miRNA content to recipient cells. As diabetes increases the risk of CVD, we investigated how glucose intolerance influences the release of intracardiac EVs post‐MI and their miRNA content.

**Methods**: B6J mice were fed chow diet or high‐fat diet (HFD) for 3 months. MI was induced by coronary artery permanent ligation. Left ventricles were harvested at different timepoints post‐surgery and processed for EV isolation. EVs were quantified by Tunable Resistive Pulse Sensing technology. Parental cell origin and EV characterisation were determined by flow cytometry and Western blot. EV miRNA content was determined by RNAseq and validated by qPCR. Using cardiomyocyte specific GFP+ mice, circulating EVs were analysed by flow cytometry to validate whether GFP+ cardiomyocyte EVs (CMEVs) are released into the circulation. As MI and diabetes involve persistent inflammatory responses, APC+ labelled hypoxic cardiomyocyte cell line (HL‐1) lEVs were injected in HFD/control mice post‐MI to determine preferred target cells in tissues by flow cytometry.

**Results**: In control mice, release of lEVs and sEVs was significantly increased at 24h post‐MI when compared to shams. lEV levels in HFD mice were significantly higher compared to sham and control mice post‐MI. There was no difference in sEV release between sham and MI HFD mice. Most intracardiac lEVs expressed cardiomyocyte marker caveolin‐3 and harbour EV markers. Global qPCR analysis revealed multiple EV miRNAs that were dysregulated post‐MI. Among these, there is a downregulation of miRs 146a‐5p and 503–5p expression in HFD lEVs compared to control and upregulation of miR‐378a‐5p expression post‐MI in both EV subsets. Levels of intracardiac GFP+Cav‐3+ lEVs were lower in HFD than in control mice, whereas levels of circulating GFP+Cav‐3+ lEVs were higher in HFD than in control mice. In vivo biodistribution studies revealed a preferential uptake of hypoxic HL‐1 lEVs by splenic myeloid cells, with an increase in uptake in HFD spleens than in control spleens post‐MI. Further investigations are needed to define specific targeted splenic myeloid subpopulations.

**Summary/Conclusion**: Our results show that glucose intolerance modulates the release of intracardiac EVs post‐MI as well as their miRNA cargo. Furthermore, the release of CMEVs into the circulation is increased as well as their uptake by splenic myeloid cells. Further work is warranted to fully investigate the functional impact of the miRNA of interest in intracardiac EVs in the diabetic heart post‐MI.

## OD18 Bone Repair

Chair: Annalisa Radeghieri, Department of Molecular and Tranlational Medicine‐Università degli Studi di Brescia, Italy

Chair: Dimitris Tsiapalis, School of Pharmacy and Pharmaceutical Sciences & Trinity Biomedical Sciences Institute, Trinity College Dublin, Dublin, Ireland

### OD18.01 The influence of 3D printed scaffold architecture on osteoblast‐derived extracellular vesicles therapeutic efficacy for bone repair

Kenny Man, University Of Birmingham

Sophie Louth, School of Chemical Engineering, University of Birmingham, Birmingham, United Kingdom.

Mathieu Brunet, School of Chemical Engineering, University of Birmingham, Birmingham, United Kingdom.

David Hoey, Trinity Centre for Biomedical Engineering, Trinity Biomedical Sciences Institute, Trinity College Dublin, Ireland.

Sophie C. Cox, School of Chemical Engineering, University of Birmingham, Birmingham, United Kingdom.

**Introduction**: Extracellular vesicles (EVs) are considered promising nanoscale therapeutics for bone tissue engineering. To date, EVs are harvested from cells cultured on tissue culture plastic, limiting the surface area for cell growth as well as not replicating conditions in situ. Numerous studies have demonstrated the impact of 3D culture on promoting osteogenic differentiation. Additive manufacturing techniques such as 3D printing has allowed for the fabrication of biomimetic in vivo environments. Therefore, this study investigated the influence of different 3D printed scaffold architectures on the yield and therapeutic potency of osteoblast‐derived EVs for bone repair.

**Methods**: 3D printed titanium (Ti6Al4V) scaffolds with different pore sizes (500 and 1000 μm) and shapes (square and triangle) were fabricated by selective laser melting. EVs were harvested from osteoblasts cultured on different 3D printed scaffolds in osteogenic conditions for 2 weeks. Relative EV size and concentration were defined using transmission electron microscopy and nanoparticle tracking analysis. The osteogenic differentiation of human bone marrow stromal cells (hBMSCs) cultured with scaffold‐derived osteoblast EVs was evaluated by qPCR, biochemistry and histological analysis.

**Results**: Titanium scaffolds promoted the mineralisation of osteoblasts when compared to 2D culture. Moreover, osteoblasts cultured on 3D printed scaffolds secreted significantly enhanced EV quantity when compared to 2D, with scaffolds exhibiting larger pore sizes (1000 μm) and permeabilities (triangle) promoting EV yield. Osteoblast‐derived EVs isolated from scaffolds exhibited a triangle pore conformation significantly promoted hBMSCs osteogenic differentiation and extracellular matrix mineralisation when compared to EVs derived from scaffolds exhibiting square pore confirmation and 2D culture.

**Summary/Conclusion**: Taken together, these findings demonstrate the influence of 3D printed scaffold architecture on osteoblast‐derived EV yield and efficacy, indicating the potential use of these culture platforms to enhance the production of therapeutically potency EVs for bone repair.

### OD18.02 Controlled release of osteoblast‐derived extracellular vesicles from an injectable chitosan‐collagen composite hydrogel to promote bone regeneration

Kenny Man, University Of Birmingham

Mathieu Brunet, School of Chemical Engineering, University of Birmingham, Birmingham, United Kingdom.

Sophie C. Cox, School of Chemical Engineering, University of Birmingham, Birmingham, United Kingdom.

**Introduction**: For bone tissue engineering, the use of extracellular vesicles (EVs) is emerging as a promising acellular approach compared to cell‐based therapies. Despite their promise, the short half‐life of these cell‐derived nanoparticles following systemic administration hinders their therapeutic potential. Therefore, this study aimed to develop an osteoinductive chitosan‐collagen composite hydrogel capable of controlling the release of osteoblast‐derived EVs to promote bone regeneration.

**Methods**: Chitosan‐collagen composites were fabricated at ratios of 100/0, 65/35, 25/75 and 0/100 wt%. The hydrogels gelation time, compressive modulus and pore size were characterised. Osteoblast‐derived EVs were incorporated within the composites and their release kinetics were determined using the CD63 ELISA. The size, morphology and concentration of released EVs were assessed via nanoparticle tracking analysis and transmission electron microscopy. The osteogenic differentiation of human bone marrow stromal cells (hBMSCs) within the EV‐functionalised hydrogel was evaluated by qPCR, biochemistry and histological analysis.

**Results**: The presence of collagen within the composite hydrogel significantly enhanced compressive modulus and reduced gelation times and pore size. Increasing collagen content within the hydrogel, lead to a dose‐dependent reduction in EV release kinetics. In monolayer, the functional activity of hydrogel released EVs was confirmed with enhanced hBMSCs proliferation and migration. Importantly, EV‐functionalised composite hydrogels significantly promoted encapsulated hBMSCs osteogenic differentiation and extracellular matrix mineralisation when compared to the EV‐free gel during osteogenic culture.

**Summary/Conclusion**: Together, these findings demonstrate the development of an osteoinductive chitosan‐collagen composite hydrogel capable of enhancing the therapeutic delivery of osteoblast‐derived EVs as an acellular tool to promote bone regeneration.

### OD18.03 Senescence did not alter the chondroprotective effect of extracellular vesicles from adipose mesenchymal stem cells in osteoarthritis

Jérémy Boulestreau, Inserm

Marie Maumus, Inserm

Pauline Rozier, Inserm

Christian Jorgensen, University Montpellier

Daniele Noël, Inserm

**Introduction**: Age is the most important risk factor in degenerative osteoarthritis (OA) and is associated with the accumulation of senescent cells that contribute to functional decline of joint. We previously demonstrated that extracellular vesicles (EVs) from mesenchymal stromal cells (MSCs) largely mediate the therapeutic effect of parental cells in OA. Here, we assessed the impact of senescence on the characteristics of EVs from adipose tissue‐derived MSCs (ASC‐EVs) and their properties in an in vitro model of OA.

**Methods**: ASCs were induced to senescence using 25μM etoposide for 24 hours. Senescence was assessed by quantifying proliferation rate, SA‐βGal activity, nuclear γH2AX foci number, phalloidin staining and expression of cyclin dependent kinase inhibitors (CDKI) (RT‐qPCR). ASC‐EVs were isolated by differential ultracentrifugation and characterized by size, concentration, total protein content, structure (cryo‐TEM) and immunophenotype. In vitro OA model used chondrocytes isolated from OA patients, which were stimulated with IL1b for 48h before culture with ASCs or ASC‐EVs for 7 days. Expression of chondrocytic and inflammatory markers was quantified by RT‐qPCR and SASP factors were quantified by ELISA in supernatants.

**Results**: Senescence‐induced ASCs experienced growth arrest and increase of SA‐βGal staining, of p21 CDKI expression, of nuclear γH2AX foci, of stress fibers and of several SASP factors (IL6, IL8, MMP3) confirming the expression of main senescence features. Senescent ASCs produced 4‐fold more EVs than healthy ASCs and senescent ASC‐EVs were larger. In vitro, both healthy and senescent ASCs decreased fibrotic markers (type III COLLAGEN), catabolic and hypertrophic markers (MMP3, MMP13, AP) and increased COX2 expression in OA chondrocytes. By contrast, healthy ASCs decreased the expression of IL6 while senescent ASCs highly increased IL6. Looking at the role of ASC‐EVs on OA chondrocytes, we found out that both healthy and senescent ASC‐EVs were able to increase the expression of AGG and type II COLLAGEN while they decreased the expression of MMP13, AP, type X COLLAGEN, HMOX1 and IL6. Finally, healthy and senescent ASC‐EVs decreased the number of SA‐bGal positive chondrocytes but did not impact the expression of p21 in IL1b‐induced chondrocytes.

**Summary/Conclusion**: Our results indicated a chondroprotective effect of ASC‐EVs, independently of the senescent state of parental cells and suggested that EVs might act through different mechanisms than ASCs, which warrants further investigation

### OD18.04 Exosomes derived from osteogenic tumor activate osteoclast differentiation and concurrently inhibit osteogenesis to promote bone metastasis

Lijuan Yu, Department of Laboratory Medicine

Xiaoke Hao, Clinical Laboratory Department

**Introduction**: In patients with prostate cancer (PCa), bone lesions appear osteoblastic in radiographs; however, pathological fractures frequently occur in PCa patients, and bone resorption is observed in all metastatic lesions under histopathologic assessment. The mechanisms that balance the activities of osteoblasts and osteoclasts in PCa patients remain unclear. Intercellular and interorgan communication, mediated by exosomes, is a novel and powerful means of communication. However, until now, the role of PCa exosomes in PCa bone metastasis is still unknown.

**Methods**: Exosomes were isolated using ultracentrifuge and characterized. The biodistribution was accessed by in vivo imaging, ex vivo imaging and immunolabeling. Trap staining, Trap activity and the mRNA expression were performed to assess the osteoclast differentiation. ARS S staining, ALP staining, ALP activity and the mRNA expression were performed to assess the osteoblast differentiation. The tibia for microCT, HE staining, Trap staining and OCN immunolabeling were harvested to examine the bone homeostasis in vivo. After 4 weeks education, MDA PCa 2b‐luc cells were injected into the right tibia of BALB/C nude mice, and then tumor growth in bone was detected.

**Results**: The bone targeting of PCa exosomes was verified. Exosomes derived from osteoblastic (MDA PCa 2b), osteoclastic (PC3), and mixed (C4‐2) PCa cells were found here to promote osteoclast differentiation and concurrently inhibit osteoblastogenesis in vitro and in vivo, are responsible for osteolytic lesions, bone ECM remodeling, and the aggressive growth of PCa cells in bone.

**Summary/Conclusion**: Our findings not only offer a novel perspective on tumor bone metastasis, where“contrary to our initial hypothesis” exosomes derived from an osteoblastic tumor induce osteoclast differentiation, but also suggest potential therapeutic targets for PCa bone metastasis.

### OD18.05 The LC3‐conjugated extracellular vesicles originated from secretory autophagy initiate pathological calcification in osteoarthritis and regulated by sympathetic tone

Jianfei Yan, The Fourth Military Medical University

Minjuan Shen, The Fourth Military Medical University

Weicheng Lu, The Fourth Military Medical University

Xiaoxiao Han, The Fourth Military Medical University

Wenpin Qin, The Fourth Military Medical University

Kai Jiao, The Fourth Military Medical University

**Introduction**: Pathological cartilage calcification plays a very important role in osteoarthritis (OA) progression, but its initiating factor is still unclear. Secretory autophagy has been reported to facilitate calcified precursors secretion within the extracellular vesicles (EVs). Nevertheless, the relationship between secretory autophagy and EVs and their effects in pathological cartilage calcification of OA remain unknown.

**Methods**: The LC3‐mCherry/EGFP mice and α2 adrenergic receptor (Adra2) knockout mice and their wide types were used to establish the temporomandibular joint (TMJ) OA model, and the LC3‐conjugated EVs and the cartilage calcification were examined dynamically. LC3‐positive EVs from OA cartilage were collected and added to the mimic cartilage extracellular matrix to evaluate their effect on calcification. The histone deacetylase 6 (HDAC6) activity, acetyl‐α‐tubulin level and microtubule stabilization were also tested.

**Results**: LC3‐positive EVs were increased in OA cartilage comparing to the controls, and were tightly related to the OA cartilage calcification by a location‐ and time‐dependent manner. The calcified EVs within OA cartilage were produced by LC3‐positive secretory autophagy, resulting from the decreased fusion of the autophagosomes with lysosomes. In addition, the LC3‐conjugated EVs aggregated to produce calcifying nodules with high concentrations of calcium and phosphate, resembling those observed in OA calcified cartilage. Mechanism wisely, increased levels of HDAC6, but decreased level of acetylated α‐tubulin and aggravated microtubule destabilization were observed in OA cartilage comparing to the controls. The deletion of Adra2 in TMJ‐OA mice efficiently decreased the HDAC6 level, reversed α‐tubulin deacetylation and microtubule destabilization, decreased the secretion of the LC3‐conjugatied EVs, eventually ameliorating cartilage calcification and degradation.

**Summary/Conclusion**: Secretory autophagy is the origin of LC3‐conjugated EVs and plays an important role in initiating pathological cartilage calcification in OA. The activation of Adra2 by sympathetic tone plays an important role in LC3‐conjugated EVs‐induced cartilage calcification in OA, through regulating HDAC6‐mediated α‐tubulin deacetylation and microtubule destabilization.

## OD19 Metabolism and Diabetes

Chair: Ramaroson Andriantsitohaina, INSERM U1063 SOPAM, France

Chair: Sophie Rome, INRAE, France

### OD19.01 Extracellular vesicles from skeletal muscle cells isolated from extremely obese patients with and without type 2‐diabetes show different protein‐ and miRNA‐pattern

Kari Bente Foss Haug, The Blood Cell Research Group, Section for Research, Development and Innovation, Department of Medical Biochemistry, Oslo University Hospital, Ullevål

Vigdis Aas, Department of Life Sciences and Health, Oslo Metropolitan University (OsloMet), Norway

Abdille Hussein, Department of Life Sciences and Health, Oslo Metropolitan University (OsloMet), Norway

Misbah Hussain, Department of Life Sciences and Health, Oslo Metropolitan University (OsloMet), Norway

Hans Christian D. Aass, The Blood Cell Research Group, Section for Research, Development and Innovation, Department of Medical Biochemistry, Oslo University Hospital, Ullevål, Norway

Berit S. Brusletto, The Blood Cell Research Group, Section for Research, Development and Innovation, Department of Medical Biochemistry, Oslo University Hospital, Ullevål, Norway

Ole Kristoffer Olstad, The Blood Cell Research Group, Section for Research, Development and Innovation, Department of Medical Biochemistry, Oslo University Hospital, Ullevål, Norway

Anne‐Marie Siebke Trøseid, The Blood Cell Research Group, Section for Research, Development and Innovation, Department of Medical Biochemistry, Oslo University Hospital, Ullevål, Norway

Trude Aspelin, Oslo University Hospital, Ullevaal

Tuula Anneli Nyman,Proteomics Core Facility, Oslo University Hospital, Rikshospitalet, Norway

Reidun Øvstebø, The Blood Cell Research Group, Section for Research, Development and Innovation, Department of Medical Biochemistry, Oslo University Hospital, Ullevål, Norway

**Introduction**: Skeletal muscle (SkM), the largest and highly adaptable organ in human, is responsible for locomotion and energy metabolism. SkM plays an important role as secretory organ, producing modulating factors like myokines and metabolites. Recently, SkM‐derived extracellular vesicles (EV), containing bioactive components, have been described. The aim of this project was to characterize myotube‐secreted EV from extremely obese patients with type 2 diabetes mellitus (T2DM) or normal glucose tolerance (NGT) and to search for component specific profiles.

**Methods**: Muscle biopsies from bariatric surgery (T2DM: n = 6, NGT: n = 6) were used to isolate, in vitro cultivate and differentiate satellite cells into mature myotubes. EV were isolated, separated and concentrated into microvesicles (MV) and exosomes using centrifugation and filtration. Size and concentration were revealed by NTA, presence of tetraspanins analysed by flowcytometry and Hsc70/Hsp70 and calnexin with Western blot. Proteomics and RNA‐content in exosomes were analysed by LC‐MS/MS and Affymetrix microarray, and bioinformatics examined by Ingenuity Pathway Analysis.

**Results**: Concentration, size and presence of CD63‐ and CD81‐positive MV and exosomes were similar between T2DM and NGT. A total of 494 exosome proteins from both groups were detected, where 204 proteins showed significant higher levels and 144 had significant lower levels when comparing the two groups, suggesting an association to energy homeostasis and metabolism, ubiquitination, and the cardiovascular system. Results from pilot microarray analysis showed series of miRNA with significant different amounts between the groups.

**Summary/Conclusion**: In vitro cultured myotubes from extremely obese patients with T2DM or NGT synthesize similar amounts of CD63‐ and CD81‐positive MV and exosomes. In contrast, large differences in protein and microRNA exosome pattern were observed between the groups, indicating that the exosome load reflect the state of the SkM.

### OD19.03 Placental small extracellular vesicles regulate insulin sensitivity during pregnancy and induce metabolic changes in gestational diabetes mellitus

Soumyalekshmi Nair, University of Queensland

Katherin Scholz Romero, BSc, Exosome Biology Laboratory, Centre for Clinical Diagnostics, UQ Centre for Clinical Research, The University of Queensland, Australia

Dominic Guanzon, PhD, Exosome Biology Laboratory, Centre for Clinical Diagnostics, UQ Centre for Clinical Research, The University of Queensland, Australia

Andrew Lai, PhD, Exosome Biology Laboratory, Centre for Clinical Diagnostics, UQ Centre for Clinical Research, The University of Queensland, Australia

Harold David McIntyre, Mater Research Institute‐University of Queensland, Translational Research Institute, Woolloongabba, Australia

Martha Lappas, Obstetrics, Nutrition and Endocrinology Group, Department of Obstetrics and Gynaecology, University of Melbourne, Victoria, Australia and Mercy Perinatal Research Centre, Mercy Hospital for Women, Heidelberg, Victoria, Australia.

Carlos Salomon, MSc, DMedSc, PhD, The University of Queensland

**Introduction**: Growing evidence shows that extracellular vesicles play important roles in the regulation of metabolic functions. The aim of this study was to elucidate the role of placenta‐derived small extracellular vesicles (sEVs) in the regulation of maternal insulin sensitivity in Gestational Diabetes Mellitus (GDM). GDM is the fastest‐growing type of diabetes and is the most common medical complication of pregnancy.

**Methods**: Primary Human Trophoblast (PHT) cultures from Normal Glucose Tolerant (NGT) and GDM pregnancies were developed and sEVs were isolated from cell‐conditioned media using density gradient centrifugation. The protein content of PHT cells and sEVs analyzed by data‐independent acquisition (DIA) mass spectrometry. Using Illumina TrueSeq Small RNA kit, a small RNA library was constructed and miRNA profile of PHT cells and sEVs were determined.

**Results**: We identified that PHT cells express a differential profile of miRNAs and proteins in GDM compared to NGT. Interestingly, the miRNA profile of sEVs showed a significant correlation (p‐value < 0.0001) to PHT cells, indicating that sEVs are “fingerprints” of the releasing cells and their metabolic status. However, we identified a set of miRNAs and proteins differentially expressed in sEVs compared to PHT cells in GDM and NGT. This indicates the presence of mechanisms by which molecular cargo are specifically sorted to sEVs.The expression of certain miRNAs (miR‐1260b, miR‐181‐5p, miR‐589‐5p, and miR‐660‐5p) varied in a consistent pattern in PTH and their secreted sEVs in GDM compared with NGT. Using IPA analysis, miRNA and proteomic data were integrated and miRNA‐gene interactions were identified. IPA core analysis showed that the top canonical pathway associated with these miRNAs were PI3/AKT signaling and glucose metabolism/insulin resistance, respectively.

**Summary/Conclusion**: sEVs released from placenta can modify the phenotype of target cells and regulate metabolic changes in GDM contributing to changes in insulin sensitivity during pregnancy.

### OD19.04 Impact of lipid accumulation in hepatocytes in the release of COMT associated to extracellular vesicles

Maria Azparren‐Angulo, CICbioGUNE

Edward Milbank, Department of Physiology, CIMUS, University of Santiago de Compostela‐Instituto de Investigación Sanitaria de Santiago de Compostela, CIBERobn, Santiago de Compostela, Spain

Félix Royo, Exosomes laboratory, CIC biogune‐BRTA, CIBERehd, Derio, Bizkaia, Spain

Miguel López, Department of Physiology, CiMUS, University of Santiago de Compostela‐Instituto de Investigación Sanitaria

Juan Manuel Falcón‐Pérez, CIC bioGUNE

**Introduction**: Hepatocytes secrete extracellular vesicles (EVs), and we have published previously that liver damage induce important changes in the composition and amount of secreted EVs. Those circulating EVs are loaded with specific liver proteins, including active enzymes, such as, arginase 1, CYP450 and Catechol‐O‐methyl transferase (COMT). Indeed, COMT (EC 2.1.1.6) is an enzyme that metabolizes catecholamines catalysing the transfer of a methyl group from S‐adenosylmethionine to catecholamines as dopamine, epinephrine and norepinephrine. The activity of COMT has been implicated in different liver processes, such as glucose homeostasis and establishment and progression of obesity and diabetes.

**Methods**: We have studied the changes in COMT in models that mimic fatty liver and lipid accumulation in hepatocytes. As models, we have employed hepatic and non‐hepatic cell lines and compared their COMT expression. Regarding to animal models, we have employed obesity models of rats and mice. To mimic the liver damage caused by obesity, we have treated cells with a mix of oleic and palmitic acid (2:1 ratio, 1mM final) generating a lipid accumulation like the observed in vivo. Apart from in vitro treatment, we also have look at in vivo obesity models using animals with a spontaneous mutations of the Leptin gene (ob/ob) mouse and Zucker rat. For EVs extraction, the cells are cultured after the hepatic perfusion and, 48 hours later, the EVs are obtained by ultracentrifugation. The analyses of the vesicles have been done through western blot and immunofluorescence. Finally, to see the effect that EVs‐associated COMT can have in different organs and knowing that COMT has been linked to eating and neuropsychiatric behaviour, we have also studied the effect of COMT loaded EVs in rat brain.

**Results**: Our work shows differences in the abundance of COMT in extracellular vesicles depending on the cellular model and the metabolic conditions assayed in this study. In addition, the administration of EVs from metabolically‐altered hepatocytes into rat brain shows limited effect on food intake and body weight.

**Summary/Conclusion**: The lipid accumulation produce changes in the release of COMT loaded EVs that could influence food intake of animals.

### OD19.05 Feces‐derived extracellular vesicles disorganize gut‐liver axis homeostasis and contribute in the development of liver diseases

Jérôme Boursier,Centre Hospitalo‐Universitaire Angers

Alexandre Villard, INSERM U1063 SOPAM

Nadia Benabbou, INSERM U1063 SOPAM

Raffaella Soleti, INSERM U1063

Lionel Fizanne, HIFIH

Erwan Delage, LS2N

Mireille Wertheimer, INSERM U1063 SOPAM

Thibauld Oullier, INSERM U1235 TENS

Samuel Chaffron, LS2N

Michel Neunlist, INSERM U1235 TENS

Ramaroson Andriantsitohaina, INSERM U1063 SOPAM

**Introduction**: Non‐alcoholic fatty liver disease (NAFLD) is currently considered as the main chronic liver disease, with a worldwide prevalence of 25%. NAFLD may lead to the apparition of the inflammatory form of the disease, the non‐alcoholic steatohepatitis (NASH) often associated with fibrogenesis. The gut microbiota participates in the progression of NAFLD towards NASH and fibrogenesis, especially by triggering hepatic inflammatory pathways via the toll‐like receptor4 (TLR4)/lipopolysaccharide (LPS)‐mediated response. The gut microbiota is also a provider of bacterial extracellular vesicles (EVs), which are found in feces samples. In this regard, EVs derived from mouse feces have been demonstrated to induce systemic inflammation via TLR4 in a mouse model. However, no real translational study has been performed on the direct involvement of feces‐derived EVs (fEVs) in NAFLD/NASH pathophysiology.

**Methods**: fEVs were isolated from feces samples and small circulating EVs (cEVs) were isolated from blood samples from NAFLD/NASH biopsy‐confirmed patients and from non‐NAFLD/non‐NASH donors.

**Results**: fEVs and small cEVs are derived from prokaryotic and eukaryotic origins including intestinal epithelial cells. Only fEVs from NASH patients exerted deleterious effects. NASH fEVs increased intestinal permeability associated with reduced expression of tight junction proteins by a mechanism sensitive to non‐muscular myosin light chain kinase inhibition in vitro or deletion in vivo. NASH fEVs increased endothelial cell permeability and production of inflammatory cytokines and chemokines by a TLR4/LPS‐mediated pathway. NASH‐fEVs, as well as NASH‐cEVs, increased profibrotic and proinflammatory proteins expression in hepatic stellate cells. Bacterial origins of fEVs were different between stages of the liver disease and 16 amplicon sequence variants were identified as differentially abundant.

**Summary/Conclusion**: Together, our translational results support the potential role of fEVs as key players in NAFLD progression towards NASH by acting on different stages of the gut liver‐axis and warrant further preclinical and clinical studies to confirm the key targets nmMLCK and TLR4 for NASH resolution and fibrosis.

### OD19.06 Exosomes Secreted By Umbilical Cord Blood‐Derived Mesenchymal Stem Cell Promote Pancreatic Regeneration And Insulin Secretion In Mouse Model Of Type 1 Diabetes

manju kumari, Sanjay Gandhi Postgraduate Institute of Medical Sciences

SUMAN MISHRA, Sanjay Gandhi Postgraduate Institute of Medical Sciences

Dharmendra K. Chaudhary, Sanjay Gandhi Postgraduate Institute of Medical Sciences

Alok Kumar, Sanjay Gandhi Postgraduate Institute of Medical Sciences

Avni Batia, Batia Avni MD, Head of outpatient service Department of Bone Marrow Transplantation and Cancer Immunotherapy Hadassah Medical Organization, POB 12000 Jerusalem 91120 Israel

Swasti Tiwari, Sanjay Gandhi Postgraduate Institute of Medical Sciences

**Introduction**: Mesenchymal stem cells (MSCs) therapy is a recent innovative approach in diabetes due to its capacity to modulate tissue microenvironment and regeneration of glucose‐responsive insulin‐producing cells. In this study, we investigated how MSC‐derived exosomes affect the severity of diabetes and their mechanism of action.

**Methods**: Diabetes was induced in male C57Bl/6 mice by five consecutive doses of streptozotocin (STZ; 40 mg/kg body weight, i.p). The diabetic mice were administered (i.v) with MSC (1‐105 umbilical cord blood MSCs cells/day), their derived exosomes (MSC‐Exo group; that received exosomes derived from 1‐105 MSCs cells/day), or the same volume of PBS. Before administration, the potency of MSCs and their exosomes in immune cell modulation were evaluated in vitro T‐cell activation experiments. After day 7 of the treatments, blood samples, and pancreatic tissues were analyzed.

**Results**: The results revealed reduced pancreatic tissue damage with an improved histological structure in mice treated with MSCs or MSC‐Exo compared to PBS‐treated mice. Hyperglycemia was also attenuated in these mice with a concomitant increase in insulin production compared to mice in the PBS‐treated group. We found increased expression of genes associated with tissue regeneration pathways in the pancreatic tissue of mice treated with MSC or MSC‐Exo relative to PBS‐treated mice. miRNA profiling of MSCs derived exosomes showed the presence of miRs that may facilitate pancreatic regeneration.

**Summary/Conclusion**: These results demonstrate a potential therapeutic role of umbilical cord blood MSC ‐derived exosomes in diabetes by activating pancreatic islets' intrinsic regenerative abilities.

## OD20 Cancer Detection & Treatment

Chair: Rossella Crescitelli, Sahlgrenska Center for Cancer Research, Department of Surgery, Institute of Clinical Sciences, Sahlgrenska Academy, University of Gothenburg, Sweden

### OD20.01 LncRNA‐encoded Peptides: a New Form of Cargo in Cell‐derived and Circulating Extracellular Vesicles

Tanxi Cai, Institute of Biophysics, Chinese Academy of Science

Qing Zhang, Institute of Biophysics, Chinese Academy of Science

Bowen Wu, Institute of Biophysics, Chinese Academy of Science

Jifeng Wang, Institute of Biophysics, Chinese Academy of Science

Na Li, Institute of Biophysics, Chinese Academy of Science

Tingting Zhang, Institute of Biophysics, Chinese Academy of Science

Zhipeng Wang, Institute of Biophysics, Chinese Academy of Science

Jianjun Luo, Institute of Biophysics, Chinese Academy of Science

Xiaojing Guo, Institute of Biophysics, Chinese Academy of Science

Xiang Ding,Institute of Biophysics, Chinese Academy of Science

Zhensheng Xie, Institute of Biophysics, Chinese Academy of Science

Lili NiuInstitute of Biophysics, Chinese Academy of Science

Weihai Ning, Department of Neurosurgery, Sanbo Brain Hospital, Capital Medical University, Beijing, China

Xiangqian Guo,Henan Provincial Engineering Centre for Tumor Molecular Medicine, School of Basic Medical Sciences, Henan University

Runsheng Chen,Institute of Biophysics, Chinese Academy of Science

Hongwei Zhang,Department of Neurosurgery, Sanbo Brain Hospital, Capital Medical University, Beijing, China

Fuquan Yang,Key Laboratory of Protein and Peptide Pharmaceuticals & Laboratory of Proteomics, Institute of Biophysics, Chinese Academy of Sciences, Beijing 100101, China

**Introduction**: Advancements in technology over the past few years have led to the discovery of numerous biologically relevant peptides encoded by small open reading frames (smORFs) embedded in long noncoding RNA transcripts (referred to as lncRNA‐SEPs here) in a variety of species. However, the mechanisms and modes of action that underlie the roles of lncRNA‐SEPs have yet to be fully characterized. Of particular interest is whether lncRNA‐SEPs are taking part in intercellular communication between tumor cells and stromal cells in either the local or distant microenvironment.

**Methods**: In order to discover the novel lncRNA‐SEPs from cell‐derived and circulating EVs, we developed an MS‐based lncRNA‐SEPs analytical workflow, including the development of a lncRNA‐SEPs database that could entirely cover the putative lncRNA‐SEPs in humans but avoid generating a dataset as excessively large as the six‐frame translation of the entire genome. The human lncRNA transcripts deposited in the NONCODE (http://www.noncode.org/) database were scanned by ORFfinder and six‐frame translation mode to obtain all possible smORFs, and then theoretically translated into SEPs. Purified EVs of Glioma cancer cells or circulating EVs from health donors and Glioma cancer patients were then subjected to MS‐based lncRNA‐SEPs analysis.

**Results**: We provide the first experimental evidence of 29 lncRNA‐SEPs in EVs derived from Glioma cancer cells, indicating that the EV‐mediated transfer of lncRNA‐SEP may represent a novel mechanism for intercellular communication. Intriguingly, when examining human plasma, 48, 11 and 3 lncRNA‐SEPs were identified from purified EVs, whole plasma and EV‐free plasma, respectively, suggesting that circulating lncRNA‐SEPs are primarily enriched in EVs. Most importantly, our preliminary data demonstrate that lncRNA‐SEPs in EVs can be used to distinguish Glioma cancer patients from healthy controls, indicating circulating lncRNA‐SEPs in EVs has potential diagnostic application in identifying patients with glioma.

**Summary/Conclusion**: Our results suggest that EV‐mediated transfer of lncRNA‐SEPs represent a novel mechanism of intercellular communication between cells in close proximity as well as those at a distance. This communication method could provide an opportunity to establish entirely new paradigms of intercellular and inter‐species information exchange based on the EV‐mediated release, transport, uptake, and regulatory roles of lncRNA‐SEPs.

### OD20.02 Characterization of size‐based isolated EV populations from a metastatic melanoma cell line by lipid analysis and proteomics

Felice Accattatis, Università Degli Studi di Milano

Sara Mazza, Dipartimento Di scienze Farmacologiche e Biomolecolari ‐ Università Degli Studi di Milano

Agnese Granata, Dipartimento Di scienze Farmacologiche e Biomolecolari ‐ Università Degli Studi di Milano

Elisabetta Vergani, Laboratorio di Immunoterapia dei Tumori Umani IRCCS Istituto Nazionale dei Tumori Milano

Monica Rodolfo, Laboratorio di Immunoterapia dei Tumori Umani IRCCS Istituto Nazionale dei Tumori Milano

Sara Baroni, Dipartimento Di scienze Farmacologiche e Biomolecolari ‐ Università Degli Studi di Milano

Alberto Corsini, Dipartimento Di scienze Farmacologiche e Biomolecolari ‐ Università Degli Studi di Milano

Lidia Merlo, Dipartimento Di scienze Farmacologiche e Biomolecolari ‐ Università Degli Studi di Milano

Lorenzo Arnaboldi, Dipartimento Di scienze Farmacologiche e Biomolecolari ‐ Università Degli Studi di Milano

**Introduction**: New insights into size, protein and lipid composition may help in characterizing functionality od Extracellular Vesicles (EVs) and infer with their fast development as delivery tools. Unfortunately, despite latest size‐based classifications which divide EVs in small (50‐80nm) or large (80‐120nm) exosomes, microvesicles (< 1000nm) and the new smallest (< 50nm) population (exomeres), overlapping of different EV populations and unproper separation methods impair the comprehension of their biological role.

**Methods**: To overcome this problem, we set up a reproducible ultracentrifugation (UC) method for a size‐based separation of different EV populations by 5 UC steps, in which physical and dynamic parameters are determined by an algorithm developed by Livshts et al. In the 5 fractions isolated from the culture medium of a metastatic melanoma cell line, EVs size was analyzed by transmission electron microscopy (TEM) and dynamic light scattering (Zetasizer), lipid content by gas chromatography, and protein profile by mass spectrometry and Ingenuity Pathway Analysis (IPA).

**Results**: Zetasizer and TEM analysis documented the existence of 5 different EV populations, whose relative % in saturated fatty acids gradually and continuously increased from larger to smaller EV (from 37.21±0.21 to 64.79±9.47). Proteomics identified a total of 2003 proteins differentially distributed (or even unique) among the 5 EV populations (n = 697, 819, 1079, 1621 and 1654, respectively). IPA analysis of these distributions revealed different characteristics signaling pathways.

**Summary/Conclusion**: Melanoma‐released EVs include vesicles of different size, fatty acid and protein composition. These differences may translate into distinct behaviors and functions in biological fluids and help to define the role of specific EV populations in physiological and pathological processes. Finally, these results pave the road to new pharmacological treatments to modulate EVs functions or to use EVs as pharmaceutical tools.

### OD20.03 Biofunctional peptide‐modified extracellular vesicles with encapsulation of boron compounds for boron neutron capture therapy (BNCT)

Shiori Hirase, Graduate School of Science, Osaka Prefecture University

Ayako Aoki, Graduate School of Science, Osaka Prefecture University

Kenta Morimoto, Graduate School of Science, Osaka Prefecture University

Kosuke Noguchi, Graduate School of Science, Osaka Prefecture University

Yoshihide Hattori, Research Center for Boron Neutron Capture Therapy, Osaka Prefecture University

Mitsunori Kirihata, Research Center for Boron Neutron Capture Therapy, Osaka Prefecture University

Ikuhiko Nakase, Graduate School of Science, Osaka Prefecture University

**Introduction**: Boron neutron capture therapy (BNCT) is a radiation therapeutic method for cancer therapy. In the BNCT, internalization of boron‐10 (10B) atoms by cancer cells induces the cell death by the generation of alpha particles and recoiling lithium‐7 (7Li) nuclei when irradiated with low‐energy thermal neutrons [1]. In this research, we aimed to develop extracellular vesicles (exosomes, EVs)‐based drug delivery technology for BNCT. EVs have been highly expected to be a next‐generation drug delivery carrier, because of their pharmaceutical advantages, including controlled immune responses, effective usage of cell‐to‐cell communication, and brain‐targeting. In our research, we successfully achieved for modification of hexadeca oligoarginine (R16) [2, 3] on the EV membrane to effectively induce the macropinocytotic cellular uptake (accompanied by actin reorganization, ruffling of plasma membrane, and engulfment of large volumes of extracellular fluid), leading to enhanced biological activity in BNCT.

**Methods**: Fluorescently‐labeled dodecaborate (FITC‐BSH) was encapsulated in the EVs (derived from HeLa cells) by electroporation (FITC‐BSH‐EVs). We synthesized the R16 peptides with a succinimide linker (R16‐EMCS)). The R16‐EMCS can covalently bind to amino‐group of FITC‐BSH‐EVs membrane proteins by their simple mixing. Internalization of FITC‐BSH‐EVs by C6‐glioma cells was analyzed using a confocal laser scanning microscope and a flow cytometer. Actin reorganization (lamellipodia) was assessed by phalloidin‐staining.

**Results**: Modification of the R16‐EMCS (20 μM) on the EV membrane significantly increased cellular uptake of FITC‐BSH (230 nM) into C6‐glioma cells (approximately 25‐fold increase by the peptide modification (24 hr treatment)). Macropinocytosis induction by the EVs modified with R16‐EMCS was confirmed by enhanced macropinocytosis marker and lamellipodia formations. In the thermal neutron irradiation experiments, the C6‐glioma cell‐killing effect of BSH was enhanced through macropinocytosis induction by encapsulation in the EVs and modification with R16‐EMCS.

**Summary/Conclusion**: These results provide fundamental knowledge for the further development of EV‐based intracellular delivery system in BNCT.

[1] Nakase, I. et al. ACS Omega, 5, 22731 (2020)

[2] Nakase, I. et al. Sci. Rep., 7, 1991 (2017)

[3] Nakase, I. Processes, 9, 224 (2021)

### OD20.04 Electrical and Sensitive Quantification of Extracellular Vesicles with a Reduced Graphene Oxide Field Effect Transistor Biosensor

Yi Yu, Hubei university of chinese medicine

Guo‐Jun Zhang, Hubei university of chinese medicine

**Introduction**: Microvesicles (MVs) and exosomes have received extensive attention in recent years because they are closely related to the development of tumors. Therefore, it is very important to establish a high‐sensitivity and high‐specificity detection method to detect low‐concentration exosomes and MVs.Field effect transistor (FET) biosensor is one of the most promising biosensors in recent years. Through field effect transistors, microelectrical signals caused by the interactions between biomolecules on the sensing interface are transformed into readable electrical signals and amplified, with high sensitivity and good specificity.However, the detection of exosomes and MVs using FET biosensors has not yet been reported.

**Methods**: We first fixed reduced graphene oxide (RGO) on the surface of the sensor by drip coating, and realized the label‐free detection of exosomes by modifying the CD63 antibody. Secondly, we deposited gold particles on the surface of RGO by the chloroauric acid deposition method, and then modified the double aptamer (TLS 11a, EpCAM) on it to identify the microvesicles derived from liver cancer cells(HepG2‐MVs), through the Dirac point in the electrical signal The deviation of exosomes and microvesicles were detected separately.

**Results**: The exosomes and MVs were purified by ultracentrifugation.The functionalization process was proved by the Id‐Vg electrical signal characteristic curve. The detection sensitivity is 33 particles/ul and 84 parbicles/ul, respectively. The detection method is higher than that of most biosensors, and it can detect the analyte under a complex system with high specificity. Finally, the constructed functional sensor was used to detect the levels of exosomes and MVs in the plasma of prostate cancer patients and liver cancer patients. The results show that the constructed sensor can effectively distinguish cancer patients from healthy volunteers, and can be used repeatedly and still has high detection sensitivity, indicating that the method has the potential for clinical application and is expected to be used for early diagnosis of tumors.

**Summary/Conclusion**: 1. A functional RGO FET biosensor was established for the first time to detect exosomes and MVs. The detection sensitivity is 33 particles/ul and 84 particles/ul, respectively, which is higher than most biosensors. 2. This method can effectively distinguish tumor patients from healthy volunteers and has high specificity. 3. This method can complete the label‐free detection within 30 minutes, and the sensor can be used repeatedly, and the cost is low. 4. We have established a universal method to detect exosomes or MVs derived from specific tumor cells by modifying different antibodies or aptamers, providing a new detection idea and platform for clinical detection.

### OD20.05 Development of a novel serum exosomal miRNA nomogram for the preoperative prediction of lymph node metastasis in esophageal squamous cell carcinoma

Tong Liu, Second Hospital, Shandong University

Chuanxin Wang, Second Hospital, Shandong University

**Introduction**: Preoperative prediction of lymph node (LN) metastasis is accepted as a crucial independent risk factor for treatment decision‐making for esophageal squamous cell carcinoma (ESCC) patients. Our study aimed to establish a non‐invasive nomogram to identify LN metastasis preoperatively in ESCC patients.

**Methods**: Construction of the nomogram involved 3 sequential phases with independent patient cohorts. In discovery phase (N = 20), LN metastasis‐associated miRNAs were selected from next‐generation sequencing (NGS) assay of human ESCC serum exosome samples. In training phase (N = 178), a nomogram which incorporated exosomal miRNA model and clinicopathologic was developed by multivariate logistic regression analysis to preoperatively predict LN status. In the validation phase (n = 188), we validated the predicted nomogram's calibration, discrimination and clinical usefulness.

**Results**: Four differently expressed miRNAs (chr 8‐23234‐3p, chr 1‐17695‐5p, chr 8‐2743‐5p and miR‐432‐5p) were tested and selected in the serum exosome samples from ESCC patients who have or do not have LN metastasis. Subsequently, an optimized 4‐exosomal miRNA model was constructed and validated in the clinical samples, which could effectively identify ESCC patients with LN metastasis, and was significantly superior to preoperative computed tomography (CT) report. In addition, a clinical nomogram consisting of the 4‐exosomal miRNA model and CT‐report was established in training cohort, which showed high predictive value in both training and validation cohorts (AUC: 0.880 and 0.869, respectively). Hosmer‐Lemeshow test and decision curve analysis implied the nomogram's clinical applicability.

**Summary/Conclusion**: Our novel non‐invasive nomogram is a robust prediction tool with promising clinical potential for preoperative LN metastasis prediction of ESCC patients, especially in T1 stage.

### OD20.06 Exosomes loaded with Palladium nanosheets: targeted bioothogonal catalysts against cancer

María Sancho‐Albero, Institute of Nanocience and Materials of Aragon (INMA)

María Sancho‐Albero, Institute of Nanocience and Materials of Aragon (INMA)

Belén Rubio‐Ruiz, GENYO

Ana M Pérez‐López, Technische Universität Berlin

Victor Sebastián, Institute of Nanoscience and Materials of Aragon (INMA)

Pilar Martín‐Duque, ARAID

Manuel Arruebo, Institute of Nanoscience and Materials of Aragon (INMA)

Asier Unciti‐Broceta, Cancer Research UK Edinburgh Centre

Jesús Santamaría, Institute of Nanoscience and Materials of Aragón (INMA)

**Introduction**: The transformational impact of bioorthogonal chemistry has inspired the development of new and exciting strategies for the in vivo synthesis of bioactive agents through non‐natural means. Among these, Palladium (Pd) catalysts have played a key role in the growing subfield of bioorthogonal catalysis by producing uncaging biomolecules in living systems and providing them with new functional properties. Exosomes are proposed to be ideal vehicles for targeting novel therapies. However very little is known about the selectiveness and specificity of the transference processes involving exosomes released from different cells.

**Methods**: Exosomes derived from A549 and U87‐MG cells were isolated and loaded with Pd‐ nanosheets using a CO mediated reduction procedure. Pd‐loaded exosomes (Pd‐Exos) were thoroughly characterized by CryoTEM, UV‐VIS, XPS, Western Blot, Zeta potencial, etc. Their catalytic activity was evaluated by fluorogenic studies and time‐lapse microscopy. Furthermore, we assessed the biocompatibility and the internalization of the Pd‐Exos by metabolic assays and confocal microscopy. Finally, the intracellular catalytic activation of a non‐active prodrug into Panobinostat was evaluated in A549 and U87 cells.

**Results**: A bio‐artificial device consisting of cancer‐derived exosomes loaded with Pd catalysts (Pd‐Exos) has been created by a novel method based on a CO mediated reduction of Pd within exosomes to obtain catalytically active nanostructures inside the extracellular vesicles. Pd‐Exos do not exhibit cytotoxicity at the studied doses and they are co‐localized in the cell cytoplasm and particularly, in the endosomal‐exosomal pathway. This new hybrid system mediates Pd‐triggered dealkylation reactions in vitro (inside glioblastoma cells (U81‐MG) and lung cancer cells (A549)) and serves as Trojan Horse, having a preferential tropism and fingerprint for its progenitor cells. This bioorthogonal reaction leads to cancer cell death in an effective and selective way.

**Summary/Conclusion**: This study illustrates the therapeutic potential of combining the exosome‐mediated catalysts and bioorthogonal uncaging chemistries to activate bioactive substances (such as the recently approved anticancer drug Panobinostat) in a spatio‐temporal selective way.

## OD21 Getting a HEAD Start on EVs

Chair: Vincent Hyenne, INSERM / CNRS, France

### OD21.01 Reduced, Reuse, Recycle: Replenishing extracellular vesicles lost through degeneration‐induced depletion as a novel therapy for the treatment of Age‐related macular degeneration

Yvette S M Wooff, The Australian National University

Yvette S M Wooff, The Australian National University

Adrian Cioanca, The Australian National University

Joshua A. Chu‐Tan, The Australian National University

Riccardo Natoli, The Australian National University

**Introduction**: We have previously demonstrated that retinal degeneration is associated with the depletion of extracellular vesicles (EV) and impaired miRNA shuttling via EV within the retina. We therefore hypothesized that supplementation of healthy retinal EV or their miRNA cargo could ameliorate retinal degeneration.

**Methods**: To characterize the miRNA cargo of retinal EV, RNA‐seq was performed on EV from healthy and degenerating mouse retinas. EV from healthy retinas were supplemented into the degenerating retina via intravitreal injection (2.0 × 1010 EV/eye) at day 2 of a 5‐day photo‐oxidative damage paradigm. To investigate the role of EV‐miRNA in regulating tissue homeostasis, EV abundant miRNA miR‐124‐3p was administered into the degenerating retina by intravitreal injection. Electroretinography and optical coherence tomography were used to assess retinal function and morphology while TUNEL and IBA‐1+ staining was conducted to measure cell death and inflammation.

**Results**: The top 10 most abundant EV‐miRNA made up ∼67% of the EV miRNA content, with miR‐124‐3p alone accounting for 18%. Bioinformatic pathway analysis revealed that these miRNA were associated with the regulation of inflammatory and cell survival pathways known to be heavily involved in retinal degenerations. Compared to controls, mice injected intraocularly with either retinal EV or encapsulated miR‐124‐3p had significantly higher retinal function, reduced inflammation and decreased photoreceptor cell death.

**Summary/Conclusion**: Taken together, this data supports a central hypothesis in which a loss of EV‐miRNA bioavailability is correlated to progressive retinal degeneration. Further, that replenishing levels of retinal EV and their highly abundant EV‐miRNA such as miR‐124‐3p can reduce the pathological features of degeneration. Results from this work therefore support the use of EV‐based therapies to restore homeostatic communication pathways and slow the progression of retinal degenerations.

### OD21.02 Extracellular vesicles as biomarkers in precision medicine: profiling of stroke patients by Surface Plasmon Resonance imaging

Alice Gualerzi, PhD, IRCCS Fondazione Don Carlo Gnocchi ONLUS

Silvia Picciolini, IRCCS Fondazione Don Carlo Gnocchi

Arianna Iannone, IRCCS Fondazione Don Carlo Gnocchi

Cristiano Carlomagno, IRCCS Fondazione Don Carlo Gnocchi

Francesca Rodà, IRCCS Fondazione Don Carlo Gnocchi

Angelo Montesano, IRCCS Fondazione Don Carlo Gnocchi

Marzia Bedoni, IRCCS Fondazione Don Carlo Gnocchi

**Introduction**: Stroke is the second leading cause of death worldwide. The local hypoxia induced by stroke damages the brain tissue and patients that survive after the event may present disabilities that can persist for a long time or permanently after it. The clinical approach to regenerative rehabilitation of stroke currently lacks of easily accessible and sensitive biomarkers to evaluate the optimal rehabilitation and therapy. In this study we exploited the potentiality of Extracellular Vesicles (EVs) as carriers of stroke markers and the innovative technique of Surface Plasmon Resonance imaging (SPRi) that can guarantee a multiplexed and sensitive analysis of EVs isolated from liquid biopsies for the discovery of disease‐related markers.

**Methods**: The isolation of circulating EVs from serum of ischemic stroke patients and healthy controls (age and sex matched) was obtained by size exclusion chromatography and ultracentrifugation. A SPRi biosensor was developed for the detection of EVs with different cellular origin (brain and non‐brain cells), the relative quantification of specific surface molecules related to pathological or regeneration processes was accomplished. SPRi results obtained were then correlated with clinical parameters. In parallel, EV physico‐chemical characterization following MISEV2018 requirements and quantification of serum inflammatory cytokines by ELISA assay were performed.

**Results**: Effective isolation of EVs was obtained. Stroke patients presented higher concentrations of EVs in serum, in particular EVs released by astrocytes and endothelium. Our results demonstrated that specific antigens, like Klotho and Translocator Protein (TSPO) expressed on EVs, can be potential markers for the prediction of a better functional recovery of stroke patients, as evaluated by clinical scales. Besides, EV and non‐EV serum markers related to inflammation were demonstrated to correlate with a stronger response to damage and consequent better physical conditions of patients during the acute phase.

**Summary/Conclusion**: EV associated proteins were proved as potential markers of different stroke outcomes and laid the foundation for further investigations on a wider cohort. Our results demonstrated the ability of the SPRi biosensor to reveal differences in the relative amount of specific cell‐derived EV subpopulations and in their cargo during disease progression and rehabilitation induced recovery, providing support for using the proposed SPRi‐based biosensor to foresee patients’ outcome after rehabilitation protocols and regenerative therapies.

### OD21.03 Embryonic Stem Cells Derived‐Small Extracellular Vesicles Regulate Tregs to Protect against Ischemic Stroke

Yuguo Xia, Department of Neurosurgery, Shanghai Jiao Tong University Affiliated Sixth People's Hospital

Guowen Hu, Department of Neurosurgery, Shanghai Jiao Tong University Affiliated Sixth People's Hospital

Qing Li, Institute of Microsurgery on Extremities, Shanghai Jiao Tong University Affiliated Sixth People's Hospital

Yang Wang, Institute of Microsurgery on Extremities, Shanghai Jiao Tong University Affiliated Sixth People's Hospital

Zhifeng Deng, Department of Neurosurgery, Shanghai Jiao Tong University Affiliated Sixth People's Hospital

**Introduction**: Stem cells derived‐small extracellular vesicles (sEVs) are proven to promote neurological recovery after stroke. Recent studies demonstrate a phenomenal tissue repair ability in embryonic stem cells‐derived sEVs (ESC‐sEVs). However, whether ESC‐sEVs could protect against ischemic stroke remains unknown. Immune responses play an essential role in the pathogenesis of ischemic stroke and modulating post‐stroke immune responses ameliorate ischemia‐induced brain damage. In this study, we aim to determine the therapeutic function of ESC‐sEVs, specifically, focusing on their role in immunomodulation after ischemic stroke.

**Methods**: ESC‐sEVs are intravenously administered after transient middle cerebral artery occlusion (MCAO) in C57/BL6 mice. Infarct volume, immune cells infiltration, neural death, and Tregs population were assessed 3 days after MCAO, neurological recovery was evaluated up to 28 days after stroke. Proteomics analysis was utilized to determine the key factors in ESC‐sEVs‐afforded increase of Treg and the following molecular pathway.

**Results**: ESC‐sEVs significantly decrease leukocytes infiltration, inflammatory cytokines expression, neuronal death, and infarct volume as well as alleviating long‐term neurological deficits and tissue loss after ischemic stroke. Interestingly, ESC‐sEVs induce a marked increase in Tregs after stroke. Further, ESC‐sEVs‐afforded immunomodulatory function and neuroprotection against stroke are dependent on Tregs as the depletion of Tregs almost completely abrogates the protective effects. Mechanistically, proteomic analysis reveals the enrichment of TGF‐β, Smad2, and Smad4 proteins in ESC‐sEVs which could be delivered to activate TGF‐β/Smad pathway in CD4+ T cells and therefore induce Tregs expansion. ESC‐sEVs modulate neuroinflammation and protect against ischemic stroke through the expansion of Tregs, a process which is partially dependent on the activation of TGF‐β/Smad signaling pathway by the transfer of TGF‐β, Smad2, and Smad4.

**Summary/Conclusion**: This study highlights ESC‐sEVs as a novel and promising cell‐free therapeutic for ischemic stroke, potentially other CNS injuries, and autoimmune diseases.

### OD21.04 A novel biosensor based on plastic antibodies for sensing an extracellular vesicle from neuronal cells

Ana P.M. Tavares, Biomark@UC

M.Goreti.F. G. Sales, Biomark@UC

**Introduction**: Extracellular vesicles are nanostructures containing a lipid bilayer with transmembrane proteins. They play a critical role in intercellular communication and depending on the cell origin their transmembrane proteins and their cargos may vary. So, the composition of each EV allows distinguishing its origin through its proteins or their cargos. EVs circulating in plasm may also come from the brain and provide valuable information about the impact of brain on the overall health status of the body. Thus, monitoring brain‐derived EVs may provide additional insights into neuroscience.

**Methods**: This work proposes a biomimetic polymer for EV capture from neuronal cells, acting as a plastic antibody. This material was obtained through molecular imprinting by electropolymerized Pyrrole around a glutamate Ionotropic Receptor AMPA Type Subunit 3 (GRIA3) that is a surface protein expected to be present in EVs from neuronal origin. Then GRIA3 is removed from the polymeric network, creating complementary cavities to this protein. The resulting is a molecular imprinting polymer (MIP). In parallel, a non‐imprinted polymer (NIP) was also produced, but without the protein.

**Results**: The analytical features of the MIP assembly process and its behaviour as sensing material were evaluated by electrochemical impedance spectroscopy (EIS). The MIP material was tested by using different concentrations of GRIA3 from 10ng/mL to 10mg/mL during the recognition process. The results showed a good linear response between 100 ng/mL and 10 mg/mL with ‐0.19 W/decade of slope and square correlation coefficient >0.99. The detection limit was 38.4ng/mL and the quantification limit was 100ng/mL.

**Summary/Conclusion**: Overall, the MIP obtained was sensitive and selective to the external protein GRIA3 from EVs, which can be promising to intact EVs monitoring.

### OD21.05 Highly efficient intercellular spreading of protein misfolding by viral ligand‐decorated extracellular vesicles

Ina M. Vorberg, University Bonn

Shu Liu, DZNE

Andre Hossinger, DZNE

Stefanie Heumüller, DZNE

Annika Hornberger, DZNE

Oleksandra Buravlova, DZNE

Katerina Konstantoulea, KU Leuven, Switch Lab

Stephan Müller, DZNE Munich

Lydia Paulsen, DZNE

Stefan Lichtenthaler,DZNE Munich

Frederic Rousseau, KU Leuven, Switch Lab

Joost SchymkowitzKU Leuven, Switch Lab

Manuela Neumann, DZNE Tübingen

Philip Denner,DZNE

**Introduction**: Aberrant folding and aggregation of host‐encoded proteins into ordered protein assemblies is a pathological hallmark of neurodegenerative diseases such as prion diseases and Alzheimer's disease. Pathologic protein aggregates have the ability to transmit to unaffected cells, thereby templating and propagating their own aberrant conformation onto soluble homotypic proteins. Proteopathic seeds can be released into the extracellular space, secreted in association with extracellular vesicles (EV) or exchanged by direct cell‐to‐cell contact. The extent to which each of these pathways contributes to the prion‐like spreading of protein misfolding is unclear.

**Methods**: Exchange of cellular cargo by both direct cell contact or via EV depends on receptor‐ligand interactions. We hypothesized that enabling these interactions through viral ligands enhances intercellular proteopathic seed transmission. We used different cellular models to study the effect of viral glycoproteins on intercellular exchange and propagation of protein aggregates. Induction of protein aggregates in recipient cells, either cocultured with donor cells or exposed to EV from donor cells, was analyzed using automated confocal microscopy and image analysis. Protein aggregates included self‐replicating model prion aggregates, transmissible spongiform encephalopathy agents and pathologic Tau.

**Results**: We here expressed vesicular stomatitis virus VSV‐G in three different cellular models that propagate different protein aggregates. Coculture of VSV‐G‐expressing donor cells with recipient cells strongly increased protein aggregate induction in the latter. Further, expression of VSV‐G also promoted the secretion of VSV‐G‐coated EV with drastically enhanced aggregate‐inducing capacity in recipient cells. Intriguingly, interactions between SARS‐CoV‐2 spike S protein and its receptor ACE2 similarly contributed to the spreading of prions and Tau aggregates.

**Summary/Conclusion**: We conclude that efficient intercellular proteopathic seed transfer is strongly controlled by receptor‐ligand interactions. Our data raise the intriguing possibility that viral glycoproteins, expressed during acute or chronic infection, could facilitate the spreading of protein misfolding in vivo.

### OD21.06 Extracellular vesicles cargo as biomarker of mitochondrial dysfunction in Huntington's Disease

Margarida Beatriz, CNC‐Center for Neuroscience and Cell Biology, University of Coimbra, Coimbra, Portugal; Institute for Interdisciplinary Research of the University of Coimbra (IIIUC), Coimbra, Portugal

Rita Vilaça, CNC‐Center for Neuroscience and Cell Biology, University of Coimbra, Coimbra, Portugal; Institute for Interdisciplinary Research of the University of Coimbra (IIIUC), Coimbra, Portugal

George Daley, Division of Hematology/Oncology, Boston Children's Hospital and Dana Farber Cancer Institute, Boston, MA 02115, USA; Stem Cell Program, Boston Children's Hospital, Boston, MA 02115, USA; Harvard Medical School, Harvard Stem Cell Institute, Boston, MA, USA

Cristina Januário, Neurology Service, Coimbra Hospital and Universitary Centre, Coimbra, Portugal; FMUC‐Faculty of Medicine, University of Coimbra, Coimbra, Portugal

Thorsten Schlaeger, Division of Hematology/Oncology, Boston Children's Hospital and Dana Farber Cancer Institute, Boston, MA 02115, USA; Stem Cell Program, Boston Children's Hospital, Boston, MA 02115, USA

A. Cristina Rego, CNC‐Center for Neuroscience and Cell Biology, University of Coimbra, Coimbra, Portugal; FMUC‐Faculty of Medicine, University of Coimbra, Coimbra, Portugal

Carla Lopes, CNC‐Center for Neuroscience and Cell Biology, University of Coimbra, Coimbra, Portugal; Institute for Interdisciplinary Research of the University of Coimbra (IIIUC), Coimbra, Portugal

**Introduction**: Huntington's disease (HD) is a neurodegenerative disorder caused by an expansion of a CAG repeat in the HTT gene that encodes for a mutant form of the huntingtin protein. The role of extracellular vesicles (EV) in HD progression is currently being investigated and our results showed the presence of mitochondrial components (DNA, proteins) in EV released from HD patient's cells.

**Methods**: EV were isolated from media of human fibroblasts (Fb) (3 CTR, 3 presymptomatic (pHD) and 2 late‐stage HD (HD)) through differential ultracentrifugation and analyzed by nanosight tracking analysis, transmission‐electron microscopy (TEM) and immunoblotting for characterization of exossomal markers. Live‐imaging was used to evaluate intercellular trafficking of mitochondrial proteins via EV and RT‐qPCR to quantify EV mitochondrial DNA (mtDNA) copy number.

**Results**: We detected the presence of TFAM (mtDNA transcription factor) by TEM and the incorporation of mtDNA in EV released from HD cells. Live‐cell imaging of GFP tagged EV and DsRed labelled mitochondria showed a colocalization in Fb suggesting that EV can transfer mitochondrial proteins intercellularly. An increased secretion of EV was observed in Fb from patients with higher CAGs number. The number of mtDNA copies were higher in pHD vs HD Fb and released EV, displaying a positive correlation between them.

**Summary/Conclusion**: Human Fb exchange EV loaded with mitochondrial components. Additionally, the increased number of mtDNA copies in cells and EV from presymptomatic HD suggests a pathogenic role for mitochondrial cargo within EV in HD progression.

## On Demand Poster Sessions (PS)

## PS01 EVs and Inflammation

Chair: Leonid Margolis, Eunice‐Kennedy National Institute of Child Health and Human Development, United States

Chair: Saara Laitinen, Finnish Red Cross Blood Service, Finland

### PS01.01 KIM‐1‐mediated exosomes uptake is crucial for renal tubulointerstitial inflammation induced by hypoxia

Jun Chen, Institute of Nephrology, Zhongda Hospital, Southeast University School of Medicine, Nanjing, China

Zuo‐Lin Li, Institute of Nephrology, Zhongda Hospital, Southeast University School of Medicine, Nanjing, China

Tao‐Tao Tang, Institute of Nephrology, Zhongda Hospital, Southeast University School of Medicine, Nanjing, China

Jing‐Yuan Cao, Institute of Nephrology, Zhongda Hospital, Southeast University School of Medicine, Nanjing, China

Cui Wang, Institute of Nephrology, Zhongda Hospital, Southeast University School of Medicine, Nanjing, China

An‐Ran Shen, Institute of Nephrology, Zhongda Hospital, Southeast University School of Medicine, Nanjing, China

Xin Zhong, Institute of Nephrology, Zhongda Hospital, Southeast University School of Medicine, Nanjing, China

Bi‐Cheng Liu, Institute of Nephrology, Zhongda Hospital, Southeast University School of Medicine, Nanjing, China

Lin‐Li Lv, Institute of Nephrology, Zhongda Hospital, Southeast University School of Medicine, Nanjing, China

**Introduction**: Renal tubular epithelial cells (RTECs) exposed to hypoxia during kidney injury communicate with interstitial inflammatory cells and contribute to tubulointerstitial inflammation. However, the role and underlying mechanism of the communication among RTECs in renal inflammation induced by hypoxia are not fully understood. The purpose of our study is to explore the role of exosomes secreted by hypoxic RTECs in the development of renal tubulointerstitial inflammation and the related mechanism.

**Methods**: Ischemia reperfusion(I/R) induced acute kidney injury (AKI) and unilateral ureter obstruction (UUO) model were established. HIF‐1α (a marker of hypoxia), inflammatory factors, KIM‐1, CD63 and ALIX (markers of exosomes) were detected. Immunofluorescence staining was used to observe the localization between KIM‐1 and exosomes. In Vitro, HIF‐1α, inflammatory factors, KIM‐1, Rab27a and markers of exosomes were detected in hypoxic RTECs. Furthermore, we detected inflammatory factors after silence of KIM‐1 and Rab27a of hypoxic RTECs. Besides hypoxic RTECs derived exosomes with or without fluorescent probe labelled were cocultured with RTECs with or without inhibition of KIM‐1. Then Q‐PCR and flow cytometry were separately performed to test inflammatory factors and uptake efficiency of exosomes of recipient RTECs. In vivo, hypoxic RTECs derived exosomes with or without fluorescent probe labelled were injected into kidney with or without I/R injury. Finally, phosphatidylserine on the surface of hypoxic exosomes was detected by nanoflow cytometry (NanoFCM).

**Results**: Tubulointerstitial inflammation, increased exosome production and KIM‐1 expression in tubules were observed in AKI and UUO injured kidneys. Interestingly, more exosomes were enriched in KIM‐1 positive renal tubules. In vitro study showed that hypoxia could increase exosomes production and KIM‐1 expression in RTECs. Interestingly, hypoxia induced inflammation could be attenuated when KIM‐1 and Rab27a were inhibited. Phosphatidylserine (PS) was found in parts of hypoxic exosomes as detected by NanoFCM. What is more, hypoxic exosomes could induce inflammatory reaction in normal RTECs and aggravate inflammatory reaction in hypoxic RTECs, which could be relieved when KIM‐1 was knock down via siRNA transfection. Correspondingly, in vivo, exogenous hypoxic exosomes could induce inflammatory reaction in normal kidneys and aggravate inflammatory reaction in I/R injured kidneys. Most importantly, there was a co localization relationship between KIM‐1 expressing tubules and injected exosomes.

**Summary/Conclusion**: Our studies demonstrate that KIM‐1 expressed by injured tubules mediates exosomes uptake via recognizing PS, which participates in mutual dialogue among RTECs and augments the inflammation response in tubulointerstitial inflammation induced by hypoxia.

### PS01.02 Topical application of mesenchymal stem cell exosomes alleviates the imiquimod induced psoriasis‐like inflammation

Poster Presenter

Bin Zhang, Institute of Molecular and Cell Biology

Ruenn Chai Lai, Institute of Molecular and Cell Biology

Wei Kian Sim, Institute of Molecular and Cell Biology

Andre Boon Hwa Choo, Bioprocessing Technology Institute

E Birgitte Lane, Skin Research Institute of Singapore

Sai Kiang Lim, MDPhD, Institute of Medical Biology, Agency for Science, Technology and Research, Singapore. Department of Surgery, Yong Loo Lin School of Medicine, National University of Singapore, Singapore

**Introduction**: Severe psoriasis, a chronic inflammatory skin disease is increasingly being effectively managed by targeted immunotherapy but long‐term immunotherapy poses health risk and loss of response. Therefore, there is a need for alternative therapy strategies.

**Methods**: Mesenchymal stem/stromal cell (MSC) exosomes are widely known for their potent immunomodulatory properties. Here we investigated if topically applied MSC exosomes could alleviate psoriasis‐associated inflammation.

**Results**: Topically applied fluorescent exosomes on human skin explants were confined primarily to the stratum corneum with < 1% input fluorescence exiting the explant over a 24‐hour period. Nevertheless, topically applied MSC exosomes in a mouse model of imiquimod (IMQ) psoriasis significantly reduced IL‐17 and terminal complement activation complex C5b‐9 in the mouse skin. MSC exosomes were previously shown to inhibit complement activation, specifically C5b‐9 complex formation through CD59. Infiltration of neutrophils into the stratum corneum is characteristic of psoriasis and neutrophils are a major cellular source of IL‐17 in psoriasis through the release of neutrophil extracellular traps (NETs).

**Summary/Conclusion**: We propose that topically applied MSC exosomes inhibit complement activation in the stratum corneum and this alleviates IL‐17 release by NETS from neutrophils that accumulate in and beneath the stratum corneum.

### PS01.03 Exosomes isolated from mycobacteria‐infected T lymphocytes activated immune responses in macrophages

Qian Qiu, Chongqing Public Health Medical Center

Yanlin Zhao, National Center for Tuberculosis Control and Prevention, Chinese Center for Disease Control and Prevention

Ping He, National Center for Tuberculosis Control and Prevention, Chinese Center for Disease Control and Prevention

Yaokai Chen, Division of Infectious Diseases, Chongqing Public Health Medical Center, Southwest University

**Introduction**: Approximately 2 billion people are infected with Mycobacterium tuberculosis worldwide, the etiological agent of tuberculosis, and 1.5 million of whom die annually. Macrophages are the primary host cells for Mycobacterium tuberculosis in humans, which has a remarkable capacity to survive within the hostile environment of macrophages. Macrophages infected with mycobacterium release exosomes that promote recruitment and activation of immune cells during granuloma formation was well known, however, the effects of exosomes released by infected lymphocytes on the capacity of macrophages were not well understood.

**Methods**: We identified exosomes from cytotoxic T lymphocytes infected with M. tuberculosis early secretory antigenic target‐6 (ESAT‐6) and culture filtrate proteins (CFP) or left uninfected as control. Relative immune protein expression changes in macrophages (i.e. RAW 264.7 cells) treated by infected and uninfected exosomes were investigated via in‐depth proteomics approach. Pathway enrichment analysis was carried out through the Gene Ontology function, Kyoto Encyclopedia of Genes and Genomes, and Gene Set Enrichment Analysis to identify pathways and functional annotation of the differential expression of immune proteins. Furthermore, assay of colony‐forming units was also used to measure the effects against mycobacteria (i.e. M. tuberculosis H37Rv) in macrophages.

**Results**: Exosomes purified from uninfected T lymphocytes were found to induce antigen specific IL‐2 and IL‐2Rα expression in macrophages, while IL‐2 and IL‐33 were induced by exosomes derived from M. tuberculosis ESAT‐6 and CFP infected lymphocytes IFN‐γ was not found in either group. The production of IL‐2 however was greatly increased in induction by infected lymphocyte exosomes. The mostly enriched proteins in macrophages induced by infected exosomes were found to be related to positive regulation of T cell activation, positive regulation of immunoglobulin production, negative regulation of cell migration in biological processes. They were also especially involved in cytokine‐cytokine receptor interaction, Th1 and Th2 immune responses. In comparison, uninfected exosome induced a more limited Th1 response. Infected exosomes were found to be superior to control exosomes in inhibition of mycobacteria within macrophages.

**Summary/Conclusion**: Our study revealed that exosomes derived from mycobacteria‐infected T lymphocytes may play an important role in regulating Th1 and Th2 responses in macrophages during M. tuberculosis infection, which suggested exosomes might serve as a novel cell‐free vaccine against an M. tuberculosis infection.

### PS01.05 LL‐37 ameliorates mouse sepsis by inducing the section of antimicrobial microvesicles from neutrophils

Yumi Kumagai, Juntendo University

Soichiro Kakuta, Juntendo University

Kyoko Kuwahara, Juntendo University

Etsuo Susaki, Juntendo University

Isao Nagaoka, Juntendo University

**Introduction**: Extracellular vesicles (EV), such as exosomes and microvesicles (MV), secreted upon microbial infection modulate immune and infectious responses. Sepsis is a life‐threatening multiple organ dysfunction caused by a systemic dysregulated inflammatory response to infection. Nevertheless, numerous therapeutic trials concerning immune dysfunction have still been disappointing. We previously revealed that LL‐37, a human cathelicidin host‐defense peptide, improves the survival of cecal ligation and puncture (CLP) septic mice. We herein investigated the potential of LL‐37 to secrete EV and the functions of EV in CLP.

**Methods**: EV isolated from the peritoneal exudates of CLP mice and the supernatant of LL‐37‐stimulated mouse bone marrow neutrophils by differential centrifugation or size exclusion chromatography were analyzed by transmission electron microscopy (TEM), flow cytometry, western blotting, and resistive pulse sensing. The antibacterial activity of EV was evaluated by incubating with Escherichia coli.

**Results**: TEM revealed particles with sizes of 50–1000 nm of EV fractions isolated from both PBS‐ and LL‐37‐injected CLP mice. The level of EV, especially neutrophil‐derived MV, was enhanced by LL‐37 administration. Interestingly, EV isolated from LL‐37‐injected CLP mice contained higher amounts of neutrophil‐derived antibacterial molecules and exhibited higher antibacterial activity compared to EV from PBS‐injected CLP mice. When exosomes and MV were partially separated by differential centrifugation, the MV fractions mainly possessed the antibacterial molecules and the antibacterial activity. Furthermore, LL‐37 stimulated neutrophils to secret EV with antibacterial potential, and the administration of EV isolated from LL‐37‐stimulated neutrophils reduced the bacterial load and improved the survival of CLP mice.

**Summary/Conclusion**: LL‐37 induces the secretion of antimicrobial EV, predominantly MV from neutrophils, in septic mice, thereby reducing the bacterial load and protecting mice from lethal septic conditions.

### PS01.06 Extracellular vesicles are associated with C‐reactive protein during sepsis

René Weiss, Danube University Krems

Birgit Fendl, Center for Biomedical Technology, Danube University Krems

Tanja Eichhorn, Center for Biomedical Technology, Danube University Krems

Silke Huber, Division of Hygiene and Medical Microbiology, Medical University of Innsbruck

Viktoria Weber, Center for Biomedical Technology, Danube University Krems

**Introduction**: We characterized the association of C‐reactive protein (CRP) with extracellular vesicles (EVs) in plasma from sepsis patients and assessed the ability of a commercial CRP adsorbent (Pentrasorb, Pentracor, Hennigsdorf, Germany) to deplete free and EV‐associated CRP. In addition, we characterized the potential pro‐inflammatory effects of EV‐bound CRP on monocytes.

**Methods**: The association of EVs with CRP was characterized by flow cytometry and Western Blotting. Plasma CRP levels were quantified using ELISA. To deplete CRP, plasma from sepsis patients was incubated with Pentrasorb (10 vol%) for 60 min in vitro. Primary human monocytes were stimulated with isolated EVs (20,000 g, 30 min) and monocyte IL‐8 secretion was quantified by ELISA to assess the biological effect of CRP depletion.

**Results**: Septic plasma (n = 30) contained 227.0±88.6 mg/L CRP vs. 0.7±0.4 mg/L for healthy controls (n = 5). Both, total EVs and CRP+ EVs were significantly elevated in septic plasma as compared to healthy controls (14,732±14,657 EVs/μL with 45.9±17.2% CRP+ EVs vs. 3,741±2,328 EVs/μL with 0.2±0.2% CRP+ EVs). Incubation of septic plasma with Pentrasorb resulted in depletion of free CRP (247.2±72.6 mg/L before vs. 1.8±0.7 mg/L after adsorption) as well as in a significant reduction in CRP+ EVs (15,053±3,992 EVs/μL with 61.0±5.0% CRP+ EVs before vs. 6,097±1,973 EVs/μL with 1.8±1.3% CRP+ EVs after adsorption; n = 3). Septic EVs induced a significant release of IL‐8 in monocytes as compared to EVs from healthy donors (3,409.0±3,545 pg/mL, n = 7 vs. 1,333.0±202.9 pg/mL, n = 4). EVs from CRP‐ depleted septic plasma induced significantly lower IL‐8 levels.

**Summary/Conclusion**: Treatment of septic plasma with Pentrasorb efficiently removes free CRP and detaches CRP from the EV surface, resulting in reduced proinflammatory effects.

### PS01.07 Extracellular vesicles from activated platelets induce a shift towards proinflammatory monocyte subsets

Tanja Eichhorn, Center for Biomedical Technology, Danube University Krems

Birgit Fendl, Center for Biomedical Technology, Danube University Krems

René Weiss, Danube University Krems

Andreas Spittler, Core Facility Flow Cytometry & Surgical Research Laboratories, Medical University of Vienna

Viktoria Weber, Center for Biomedical Technology, Danube University Krems

**Introduction**: Circulating monocytes comprise classical (CM, CD14++CD16‐), intermediate (IM, CD14++CD16+), and non‐classical (NCM, CD14+CD16++) subsets. Changes in subset distribution, have been described in various pathologies including sepsis. We analyzed the distribution of monocyte subsets following monocyte isolation from whole blood and the potential influence of platelets and platelet‐derived extracellular vesicles (EVs) on monocyte subset distribution. Additionally, we assessed the immunomodulatory properties of mesenchymal stem cells (MSCs) on monocyte subsets.

**Methods**: Peripheral blood mononuclear cells (PBMCs) were isolated from freshly drawn human whole blood using Ficoll gradient centrifugation. Primary human monocytes were isolated from PBMCs by negative depletion of non‐monocytes using magnetic beads labeled with anti‐CD3 and anti‐CD7 to label T cells, anti‐CD16 and anti‐CD123 (granulocytes), anti‐CD19 (B cells), anti‐CD56 (NK cells, T cells), as well as anti‐CD235a to label red blood cells. The association of monocytes with platelets (CD41+) and platelet‐derived EVs (CD41+lactadherin+) was assessed by flow cytometry.

**Results**: Monocyte subset distribution post isolation (83.7±3.3% CM, 5.4±2.8% IM, 10.9±5.0% NCM; n = 4) did not differ from the distribution in whole blood. Isolated monocytes contained residual platelets (monocyte‐to‐platelet ratio of 1:2) and platelet‐derived EVs. Overnight storage of isolated monocytes, but not of whole blood, led to a significant increase in IM (86.4±6.2% vs. 50.5±11.8% CM, 5.4±2.6% vs. 47.1±13.4% IM, and 8.2±4.2% vs. 2.4±2.0% NCM at 0h vs. 15h).

**Summary/Conclusion**: Storage of isolated monocytes induces a shift towards CD16 expressing proinflammatory monocytes, which seems to be mediated by residual platelets and platelet‐derived EVs. The mechanisms by which platelet EVs can trigger this shift remain to be clarified, and we are currently also assessing whether MSCs and MSC‐derived EVs can revert this shift in an inflammatory setting.

### PS01.08 Human and murine macrophages show differential activation responses to extracellular vesicles released by Leishmania (Leishmania) amazonensis promastigotes with distinct virulence profile

Isabelle Carlos de Souza Perez, Universidade Federal de São Paulo campus Diadema

Natasha FC. Ferraz de Campos Reis, Universidade Federal de São Paulo campus Diadema

Talita V. Vieira Dupin, Universidade Federal de São Paulo campus Diadema

Rogeria Cristina Zauli, Universidade Federal de São Paulo campus Diadema

Ana Claudia Claudia. Torrecilhas, Universidade Federal de São Paulo campus Diadema

Patricia X. Xander, Universidade Federal de São Paulo campus Diadema

**Introduction**: Leishmaniasis is a heterogeneous group of diseases caused by Leishmania protozoan that affects about 700,000 to 1.2 million people annually. The infection initiates when during blood meal infected female sandfly injects promastigote form into vertebrate host. The parasites are then phagocytized by specialized cells, such as macrophages, and within these cells the parasite differentiate into amastigote form. Leishmania subverts the activation of the immune system by several mechanisms including the release of virulence factors in extracellular vesicles (EVs). In this work we evaluated the EVs released by Leishmania (Leishmania) amazonensis with different virulence profile on human (THP‐1 lineage) and murine (RAW 264.7 lineage) macrophages activation.

**Methods**: Virulent L. amazonensis promastigotes were recovered by consecutive and successive infection in animals and attenuated parasites were obtained after long live period in vitro culture (100 passages in culture). After 24 hours of stimulation with EVs released from virulent and attenuated parasites, human and murine macrophages were infected with the parasites for 24 hours. The percentage of infection, mean of internalized parasites, phagocytic index, and the cytokine expression were the parameters evaluated.

**Results**: Our results showed that both human and murine macrophages showed a significant increase in the percentage of infection, mean of internalized parasites and phagocytic index, as compared to no‐stimulated macrophages infected with the parasites. The cytokine expression showed that RAW 264.7 cells treated with virulent and attenuated EVs had a significant increase in IL‐10 cytokine expression and a significant decrease in TNF‐α expression. However, murine cells stimulated with EVs from virulent parasites showed higher expression of IL‐10. On the other hand, THP‐1 cells stimulated with EVs released by virulent or attenuated L. amazonensis showed a significant increase in TNF‐α expression but a significant decrease in IL‐10 expression.

**Summary/Conclusion**: These results suggest that human and murine macrophages showed different cytokine expression profile after stimulation with L. amazonensis EVs. In addition, the EVs released by L. amazonensis modulated macrophages favoring parasite infection. A better understanding of the role of these EVs in immune system modulation and phagocytic cell activation may contribute to uncover the mechanisms of EVs involved in the parasite‐host relationship in leishmaniasis.

### PS01.09 Extracellular vesicles released by tumor cells exposed to cigarette smoke promotes the shift from Th9 differentiation to Foxp3 regulatory T cells

Paula Barbim Donate, University of São Paulo

Carlos Wanderley, University of São Paulo

Fernanda Turaça, University of São Paulo

Fausto Almeida, University of São Paulo

José Alves‐Filho, University of São Paulo

Thiago Cunha, University of São Paulo

Fernando Cunha, University of São Paulo

**Introduction**: Smoking is a major risk factor contributing to diseases development, and poor response to therapy. In cancer, most of smoking effects are related to its mutagenic potential. Extracellular vesicles (EVs) as emerged as important carriers of bioactive molecules related to cancer progression. However, the direct effects of exposure to cigarette smoke on EVs content, release and function are limited. Considering that cigarette smoke is a potent immune response modifier, we hypothesized that exposure of tumor cells to cigarette components would impact the immunomodulatory EVs functions and contributes to tumor immunological escape.

**Methods**: Tumor cell line B16 were cultured in the presence or absence of a Cigarette Smoke Enriched Medium (CSEM). After 48 h the cells were evaluated for the expression of Ki67 and PDL‐1 by flow cytometry. Extracellular vesicles (EVs) from supernatant were isolated by ultracentrifugation and submitted to western blot and nanosight analysis. MicroRNA expression was also evaluated by real‐time PCR. Naïve T cells was polarized in vitro for Th9 in the presence of B16‐derived EVs. C57/BL6 mice were submitted to melanoma experimental model and exposed or not to cigarette smoke.

**Results**: The exposure of mice to cigarrete smoke in melanoma experimental model increases tumor size. In vitro, the presence of CSEM in cultured B16 tumor cells increased their expression of Ki67 and PDL‐1, but EVs size and quantity were not affected. Besides the presence of the CD63 marker, EVs also expressed Ago2 protein. EVs derived from B16 cells exposed to CSEM modulates some microRNAs and increase the numbers of T CD4+ Foxp3+ cells during the differenciation for Th9.

**Summary/Conclusion**: The increased tumor size in melanoma experimental model by cigarette smoke exposure indicate a role in disease progression. The tumor microenvironment is a dinamic and complex place and its modulation can promote tumor immunological scape. In this context, EVs are important molecules involved in cell‐cell communication and the induction of regulatory T cell in place of the Th9 cells, recognized for their anti‐tumor effects, can contribute to tumor development. Our data suggest that smoking can exert its function through EVs immunomodulatory functions mediated, in parts, for their microRNAs content.

### PS01.10 Effect of menthol and audiovisual cue on nicotine metabolism, smoking‐associated oxidative stress, and inflammation

Asit Kumar, Ph.D., University of Tennessee Health Science Center

Namita Sinha, UTHSC

Sanjana Haque, University of North Carolina at Chapel Hill

sunitha kodidela, UTHSC

Tengfei Wang, UTHSC

Angel Garcia G. Martinez, UTHSC

Hao Chen, UTHSC

Santosh Kumar, UTHSC

**Introduction**: Tobacco products such as e‐cigarettes pose potential adverse health effects caused by direct exposure to aerosolized nicotine and flavorant such as menthol. The interaction between nicotine and an audiovisual (AV) cue was also studied. In this study, we aimed to investigate whether nicotine and menthol as flavor cue modulate nicotine‐metabolizing enzyme CYP2A6, α7 nAChR, and antioxidant enzymes such as SOD1 and catalase in plasma extracellular vesicles (EVs). Modulation of these enzymes would eventually lead to nicotine‐induced toxicity and HIV‐1 pathogenesis via EVs.

**Methods**: In this study, rats were assigned into 3 different groups: (a) rats with self‐administered nicotine in response to flavor cue; (b) rats with self‐administered nicotine in response to AV cue; (c) rats with self‐administered nicotine in response to AV and flavor cues. Also, blood samples were collected before self‐administered nicotine from each group. We isolated and characterized EVs as per ISEV guidelines from rat plasma before and after self‐administered nicotine with either flavor cue, AV, or both. Protein associated with CYP2A6, SOD1, and catalase were quantified by western blot. Cytokine and chemokine profiling in plasma and EV before and after self‐administered nicotine was performed using multiplex ELISA.

**Results**: We measured the size, total protein, and AChE activity of EVs and found no significant difference in these characteristics before and after self‐administered nicotine. We evaluated the expression of EV markers CD9 and CD63. The results showed that self‐administered nicotine increased the levels of CD9 (p ≤ 0.05), the marker of small vesicles in response to AV and flavor cues. Expression of CYP2A6 was significantly increased (P ≤ 0.001) after self‐administered nicotine in response to AV and flavor cues. The expression of nicotine receptor α7 nAChR did not change under any conditions used. Despite the noticeable effect on SOD1 and catalase, statistical significance was not observed following self‐administered nicotine. Among cytokine and chemokine profiling, we found a significant increase in the levels of MCP‐1 in EV after self‐administered nicotine in response to response to AV and flavor cues. Further investigation underlying the effect of self‐administered nicotine in response to AV and flavor cues on HIV pathogenesis is underway.

**Summary/Conclusion**: Nicotine self‐administration increased, though not statistically significant, the levels of circulatory EVs. Moreover, the study provided evidence that nicotine in response to AV and flavor cues increased nicotine metabolizing CYP2A6 in all the groups and AOEs and cytokines in specific groups.

### PS01.11 Development of in vitro functional assays to assess the immunomodulatory effects of mesenchymal stromal cells derived‐extracellular vesicles

Giada De Lazzari, Department of Women's and Children's Health, University of Padova, Padua, Italy

Ricardo Malvicini, Instituto de Medicina Traslacional, Trasplante y Bioingenieria (IMeTTyB‐CONICET)

Anna Maria Tolomeo, L.i.f.e.L.a.b. Program, Consorzio per la Ricerca Sanitaria (CORIS), Veneto Region, Padua, Italy

Marcin Jurga, Exo Biologics, Niel, Belgium

Michela Pozzobon, Department of Women's and Children's Health, University of Padova, Padua, Italy

Maurizio Muraca, Department of Women's and Children's Health, University of Padova, Padua, Italy

Gustavo Yannarelli, Instituto de Medicina Traslacional, Trasplante y Bioingenieria (IMeTTyB‐CONICET), Buenos Aires, Argentina.

**Introduction**: There is increasing interest in using extracellular vesicles derived from mesenchymal stromal cells (MSC‐EVs) as therapeutic tools, mainly due to their immunomodulatory properties. However, it is well established that the functional capabilities of these EVs are affected by a large variability, similarly to their cells of origin. Therefore, a potency assay is required to verify that the cellular product exerts the intended effect in a dose‐dependent fashion. Such assays are difficult to standardize due to the inherent inconsistency of biological systems, especially with primary cells in vitro. In the present work, we evaluated the feasibility of a macrophage and a lymphocyte cell line as reproducible tools to measure some modulatory effects of MSC‐EVs, respectively, on the innate and acquired immune system.

**Methods**: Clinical‐grade, Wharton Jelly‐derived MSC‐EVs were provided by Exo Biologics (Niel, Belgium).

For the macrophage assay, the Raw 264.7 cell line was challenged with increasing LPS doses at different time points in a 96‐well plate, measuring NO2‐ production by Griess assay as marker of M1 polarization. M1 polarization was confirmed by FACS analysis of CD80 and CD86.

To set the optimal condition for the T cell assay, Jurkat cells (clone E6‐1) were seeded in a 96‐well plate and stimulated with different anti‐CD3/CD28 beads with different beads/cell ratio on different time points. As read‐outs, IL‐2 production was measured in culture supernatant by ELISA, and cells were characterized by FACS to evaluate the activation marker CD69 and the ratio Treg (CD25+CD127‐ or FOXP3+)/Teff (CD25‐CD127+).

Both assays were tested with increasing doses of dexamethasone (Dex) (0.5, 1, 2 ug/mL) or MSC‐ EVs (5E7, 5E8, and 5E9/mL, determined by RPS.

**Results**: Macrophage stimulation with 10ng/mL LPS for 16h resulted in strong induction of NO production that was inhibited up to 60% by Dex in a dose‐dependent fashion. A similar dose‐dependent inhibition was observed with increasing amounts of MSC‐EVs. Inhibition of NO production was associated with a reduced expression of M1 markers.

Jurkat cell stimulation resulted in increased IL‐2 secretion and CD69 expression. Dex inhibited IL‐2 secretion by 70% and CD69 expression by 40%, in association with an increase of Treg/Teff ratio. Again, the addition of MSC‐EVs resulted in similar, dose‐dependent effects.

**Summary/Conclusion**: We set up a combination of two simple in vitro functional assays, representative of both innate and acquired immunity, to assess the immunomodulatory effects of MSC‐EVs. Although these tests need to be further evaluated on a large scale, we propose that the use of cell lines with a positive internal control (Dex) should ensure both adequate precision and robustness.

### PS01.12 Immunomodulation by extracellular vesicles from Trichinella spiralis muscle larvae: increasing tolerogenic properties of human dendritic cells

Sofija Glamočlija, Institute for Application of Nuclear Energy, INEP, University of Belgrade

Natasa Ilić, Institute for the Application of Nuclear Energy, INEP, University of Belgrade

Alisa Gruden‐Movsesijan, Institute for the Application of Nuclear Energy, INEP, University of Belgrade

Ljiljana Sabljić, Institute for the Application of Nuclear Energy, INEP, University of Belgrade

Sergej Tomić, Institute for Application of Nuclear Energy, INEP, University of Belgrade

Ljiljana Sofronić‐Milosavljević, Institute for the Application of Nuclear Energy, INEP, University of Belgrade

Maja Kosanović, Institute for the Application of Nuclear Energy, INEP, University of Belgrade

**Introduction**: Excretory‐secretory products (ES) of parasitic worms (helminths) shift hosts’ immunological balance toward Th2 and regulatory responses thus acting beneficial in chronic inflammations, i.e. autoimmune diseases. As shown in several helminths, extracellular vesicles (EVs) are active immunomodulatory component of ES. We found that Trichinella spiralis produces EVs (TsEVs) which influence cytokine production by PBMC. Now we aim to show how TsEVs influence human dendritic cells (DC), as key players in initiation, progression and regulation of immune response.

**Methods**: EVs were enriched from conditioned medium of T. spiralis muscle larvae (ES L1) by differential centrifugation. Human monocyte derived dendritic cells (DCs) were treated with TsEVs and subsequently co‐cultivated with allogenic T cells. Phenotypes and cytokine production of DC and T cells were determined by flow cytometry.

**Results**: TsEVs induce stable tolerogenic phenotype of DCs, reflected in the expression of surface markers (HLA‐DR, CD‐40, CD‐86) almost at the level of the control, except for slight elevation in the surface CD‐83, and significantly increased ILT‐3 and CCR‐7. Stimulated DCs produce significant amounts of IL‐10 and TGF‐β, and polarize immune response of T cells towards Th2 and regulatory type. T cells co‐cultured with TsEVs stimulated DCs show significant increase in the production of IL‐4 and IL‐10 with the production of IFN‐g at the level of control. Moreover, TsEVs stimulated DCs induce expansion of CD4+CD25+Foxp3+ regulatory T cells.

**Summary/Conclusion**: TsEVs influence viability, differentiation, maturation potential of DCs and their capacity to regulate T cell‐mediated immune response, similar as ES L1 of T. spiralis do. They induce tolerogenic phenotype of DCs and regulatory response of T cells. Starting from this capacity of TsEVs to convey immunomodulatory properties of ES L1, new therapeutics, based on TsEVs could be designed as novel therapy for autoimmune diseases.

### PS01.13 Air pollution Particulate Matter and EVs: involvement of PM‐fraction and PM‐activated toxic signaling pathways in EVs released by pulmonary epithelial cells

Stéphanie Alkoussa, Unité de Chimie Environnementale et Interactions sur le Vivant (UCEIV, EA 4492)

Sylvain billet, Unité de Chimie Environnementale et Interactions sur le Vivant (UCEIV, EA 4492)

nour jaber, 1Unité de Chimie Environnementale et Interactions sur le Vivant (UCEIV, EA 4492)

Perrine J. J. Martin, 1Unité de Chimie Environnementale et Interactions sur le Vivant (UCEIV, EA 4492)

**Introduction**: Poor air quality associated with high levels of particulate matter (PM) is one of the five greatest environmental risks for health, causing millions of premature deaths each year (cardiovascular diseases, cancer, COPD,…). PM is composed of hundreds of different chemicals, such as organic (Polycyclic Aromatic Hydrocarbons, PAHs) and inorganic or hydrosoluble compounds (ions and metals), as well as biological species (bacteria). Due to their diameter < 2.5 μm, PM2.5, or fine particles, can penetrate deep into the lung alveoli. Exposure of lung epithelial cells to PM2.5 triggers the activation of toxic pathways such as: (1) the AhR signaling pathway involved in the metabolic activation of PAHs, (2) the TLR4 signaling pathway involved in the inflammatory response, and (3) the production of Reactive Oxygen Species (ROS), which cause severe damage to cellular macromolecules. Numerous studies also show that PM2.5 induces the secretion of EV by exposed cells, but neither the fraction of PM nor the signaling pathways involved are known. Answering these questions is the objective of this study.

**Methods**: First, BEAS‐2B lung epithelial cells were exposed to PM2.5 and their organic and hydrosoluble extracts for 24 and 48 hours. Second, in order to determine the signaling pathways involved, BEAS‐2B cells were pre‐treated prior to exposure to PM2.5 with optimized concentrations of the following three specific inhibitors: CH223191 (AhR antagonist), TAK‐242 (TLR4 antagonist) and NAC (antioxidant). At the end, the EVs were isolated by SEC method, quantified by Nanosight and validated by western‐blot.

**Results**: The organic and biological fractions of the PM2.5 induce the release of EVs from exposed cells and the pre‐incubation with the three inhibitors prior to exposure has an impact on this release.

**Summary/Conclusion**: Our study highlights, for the first time, the involvement of organic and biological fractions of PM2.5 and of the induced toxic signaling pathways in EV release.

### PS01.14 T cell‐derived extracellular vesicles in the allergic airway

Kaitlyn E. Bunn, Department of Pathology, Microbiology, and Immunology, Vanderbilt University Medical Center

Heather H. Pua, Department of Pathology, Microbiology, and Immunology, Vanderbilt University Medical Center

**Introduction**: T cells are immune cells known to secrete extracellular vesicles (EVs) following stimulation that carry cell‐derived cargoes. In vitro, it has been shown that T cell‐derived EVs can induce pro‐inflammatory behaviors in target cells. However, the presence and relevance of T cell‐derived EVs in allergic airway pathology, in vivo, is not known. We previously found that immune cell‐derived EVs are increased in the airways of mice with induced allergic airway inflammation compared to control mice. Given that T cells are major drivers of inflammation in the allergic airway, the goal of this study was to determine if T cells contribute to immune cell‐derived EVs present in the allergic airway.

**Methods**: Allergic airway inflammation was induced in mice by ovalbumin sensitization and challenge. Bronchoalveolar lavage fluid (BALF) was collected from control mice and mice challenged with allergen in the airways. BALF was serially centrifuged to remove cells and debris. T cell‐specific labeling of membranes in vivo coupled with high sensitivity vesicle flow cytometry was used to detect the presence of T cell‐derived EVs in the BALF.

**Results**: T cell‐derived EVs were present in the BALF of mice challenged with allergen in the airways but absent from BALF of control mice. Using T cell‐specific membrane labeling and high sensitivity vesicle flow cytometry, we identified that 3% of fluorescence positive vesicles in the BALF of mice with induced allergic airway inflammation were of T cell origin and recovered over 6 million T cell‐derived vesicles per airway.

**Summary/Conclusion**: These results provide evidence that T cells recruited to the airways during allergic airway inflammation contribute to the population of immune cell‐derived EVs in the allergic airway. Now that the presence of T cell‐derived EVs in the allergic airway has been established, we can start to understand how they may mediate intercellular communication in the immune system through the cargoes they carry and contribute to allergic and asthma pathology.

### PS01.15 CD24 and IgM stimulation of B cells triggers transfer of functional CD24 and B cell receptor to B cell recipients via extracellular vesicles

Hong‐Dien Phan, Department of Biochemistry, Memorial University of Newfoundland

Delania J.B. Gormley, Memorial University

Reilly H. Smith, Memorial University of Newfoundland

Modeline N. Longjohn, Department of Biochemistry, Memorial University of Newfoundland

May Dang‐Lawson, University of British Columbia

Linda Matsuuchi, The University of British Columbia Vancouver

Michael R. Gold, PhD, University of British Columbia

Sherri L. Christian, Memorial University of Newfoundland

**Introduction**: Extracellular vesicles (EVs) are membrane‐encapsulated nanoparticles that carry bioactive cargo, including proteins, lipids and nucleic acids. Once taken up by target cells, EVs can modify the physiology of the recipient cells. In past studies, we reported that engagement of the glycophosphatidylinositol‐anchored receptor CD24 on B lymphocytes (B cells) causes the release of EVs. However, a potential function for these EVs was not clear. Thus, we investigated whether EVs derived from CD24 or IgM‐stimulated donor WEHI‐231 murine B cells can transfer functional cargo to recipient cells.

**Methods**: We employed a model system where donor cells expressing palmitoylated GFP (WEHI‐231‐GFP) were co‐cultured, after stimulation, with recipient cells lacking either IgM (WEHI‐303 murine B cells) or CD24 (CD24 knock‐out (CD24KO) mouse bone marrow B cells). Uptake of lipid‐associated GFP, IgM, or CD24 by labeled recipient cells was analyzed by flow cytometry.

**Results**: We found that EVs released in response to stimulation of either CD24 or IgM on the donor cells could mediate the transfer of lipids, as well as both CD24 and IgM, to recipient cells. Importantly, we found that the transferred receptors are functional in recipient cells, thus endowing recipient cells with a second BCR or sensitivity to anti‐CD24‐induced apoptosis.

**Summary/Conclusion**: Overall, these data show that extracellular signals received by one cell can change the sensitivity of neighboring cells to the same or different stimuli, which may impact B cell development or activation.

## PS02 EVs as Delivery Vehicles

Chair: Jaesun Park, Pohang University Science and Technology (POSTECH), Republic of Korea

Chair: Pieter Vader, CDL Research, University Medical Center Utrecht, The Netherlands

### PS02.01 Exosomes from 2D and 3D‐Organized cardiac explant cells show differences in miRNAs content and cytoprotective effects

Federico Buccino, Cardiocentro Ticino Institute

Edoardo Lazzarini, Cardiocentro Ticino Institute

Vanessa Biemmi, Cardiocentro Ticino Institute

Sara Bolis, Cardiocentro Ticino Institute

Giuseppe Vassalli, Cardiocentro Ticino Institute

Lucio Barile, Cardiocentro Ticino Institute

Carolina Balbi, Cardiocentro Ticino

**Introduction**: Exosome (Exo) from Cardiac progenitor cells (CPCs) are well known cardioprotective agents. The role and mechanism of action of such bioactive vesicles was associated to their miRNA content. Different culturing condition, such us hypoxia, could modulate the miRNA content, and consequentially the effect, of exosome. Here we report, for the first time, a head‐to‐head comparison of exosomes between CPC cultured in standard condition (2D‐Exo) and produced from 3D culture (3D‐Exo).

**Methods**: CPC were derived from atrial appendage explants from patients who underwent heart valve surgery. The same number of cells was spitted and growth on classical monolayer (2D culture) or in cardiosphere conformation (3D culture). Exo were obtained from serum free conditioned media and isolated by density gradient ultracentrifugation. Exo were characterized by western blot, NTA and facs analysis. Functional experiments of cardioprotection was performed on HL‐1 cardiomyocyte cell line. The miRNA content of Exo was evaluated by RT‐realtimePCR.

**Results**: Resultes: Western blot analysis, NTA and FACS confirmed the enrichment of Exo in fractions 4 and 5 among the 8 obtained by density gradient ultracentrifugation. Exo were positive for TSG101, ALIX and SYNTENIN‐1, while negative for GRP‐94 used as contaminant control, by western blot. Positivity for CD9, CD63 and CD81 was confirmed by FACS. 2D‐Exo, but not 3D‐Exo, increased cell viability and reduced ROS formation of HL‐1 cells. ERK phosporilation after 30 minutes of treatment as also observed only with 2D‐Exo. RT‐realtimePCR showed an increase of miRNA132‐3p; 146a‐5p and 181 in 2D‐Exo compared to the 3D.

**Summary/Conclusion**: These preliminary results showed how a different culture condition can change the content and role of produced exosome in the same cells.

### PS02.02 EVs bearing tissue factor improve outcome after collagenase‐induced intracranial hemorrhage

Fanny Potzeha, PhD Student in INSERM U1237 ‐ PhIND ‐ Cyceron ‐ Caen

Merve YETIM, INSERM U 1237 ‐ PhIND ‐ Cyceron ‐ Caen

Thomas Gaberel, INSERM U 1237 ‐ PhIND ‐ Cyceron ‐ Caen

Denis Vivien, INSERM U 1237 ‐ PhIND ‐ Cyceron ‐ Caen

Maxime Gauberti, INSERM U 1237 ‐ PhIND ‐ Cyceron ‐ Caen

Sara Martinez de Lizarrondo, INSERM U 1237 ‐ PhIND ‐ Cyceron ‐ Caen

**Introduction**: Hemorrhagic stroke, defined as a bleeding within the brain parenchyma remains a major cause of mortality and permanent disability. There are unmet needs for effective therapies to improve outcomes after hemorrhagic stroke, in particular, intracerebral hemorrhage (ICH), the most severe form of stroke. Early hematoma growth in ICH occurs in one third of patients within 3 hours of stroke onset and is predictor of poor outcome. Clinical trials have shown that untargeted hemostatic therapy using recombinant activated coagulation Factor VII (rFVIIa) reduced hematoma growth but conveyed an unacceptable rate of side effects. Targeted treatments able to selectively promote hemostasis at the site of bleeding are therefore necessary. Previous studies reported that monocyte‐derived extracellular vesicles (mEVs) bearing tissue factor (TF) accumulate during thrombus formation and promote thrombin generation.

The aim was to generate large amount of pro‐coagulant mEVs to be used as hemostatic patches in preclinical models of ICH.

**Methods**: Human monocytic cell‐line THP‐1 were grown in bioreactors and treated with Tumor Necrosis Factor (TNF) to generate TF+ mEVs. Isolated mEVs were finely characterized by number, size and surface antigens (TF, CD14 and P‐Selecting Ligand1) by laser‐scanning confocal microscopy and flow cytometry. Functional studies were performed using human plasma clotting, ROTEM and thrombin generation assays and TF‐activity.

In vivo, mice received an intrastriatal injection of collagenase VII to promote intraparenchymal hemorrhage. 30 minutes thereafter, exogenous mEVs were injected intravenously. Hematoma volume was quantified by Magnetic Resonance Imaging at 24h and neurological deficits were measured at 4h and 24h post‐stroke.

**Results**: Large amounts of mEVs were generated in supernatants from TNF‐stimulated monocytes in bioreactors (Celline). Those mEVs presented monocyte‐specific characteristics: a mean size of 430 nm and a high pro‐coagulant activity reducing the clotting time in a dose‐ and TF‐dependent manner. In preclinical studies of ICH, intravenous injection of mEVs improved stroke outcome in a dose‐dependent manner. mEVs at 1 mg/kg prevented hematoma growth by 43% and improved neurological score at 4h and 24h compared to control mice (p < 0.01, n = 15/group). This beneficial effect was also present in a more severe model of ICH in enoxaparin‐treated mice and was showed in a TF‐inhibition in vivo experiment. Immunohistological studies and 2‐photon imaging revealed accumulation of fluorescently‐labeled mEVs specifically at the bleeding site.

**Summary/Conclusion**: EVs bearing TF improve outcome after collagenase‐induced ICH by acting as intravascular patches.

### PS02.03 The Efficacy of EV Encapsulated Drugs is Associated with Loading Methods, EV sources and EV Uptake Efficiency in Pancreatic Cancer Cells

Wei‐Qun Ding, University of Oklahoma Health Sciences Center

Haoyao Sun, The Affiliated Suzhou Hospital of Nanjing Medical University

Kritisha Bhandari, University of Oklahoma Health Sciences Center

Stephanie Burrola, University of Oklahoma Health Sciences Center

Jingchang Wu, The Second Affiliated Hospital of Xuzhou Medical University, Xuzhou

**Introduction**: Pancreatic cancer is the third leading cause of cancer related death in the United States and the overall 5‐year survival rate of patients with pancreatic cancer is around 9%. Therapeutic options against pancreatic cancer are rather limited compared with other solid tumors. New strategies in therapeutic development is desperately needed. Recent advancement in extracellular vesicle (EV) biology has indicated that certain type of EVs, such as small EVs, possess tumor homing propensity with lower immunogenicity, and are potential drug carriers to effectively deliver cancer therapeutics.The aim of this study is to determine the efficiency of EV drug loading and the efficacy of EV encapsulated drugs in pancreatic cancer cells.

**Methods**: Small EVs were isolated from culture medium of various human cell lines using an established protocol. Paclitaxel and Gemcitabine, two commonly used chemotherapeutics, were incorporated into small EVs via Incubation, Sonication, and Electroporation. Loading efficiency was evaluated by spectrometric measurements. EV drug efficacy was analyzed by MTS assay. EV uptake was assayed by florescent microscopy with PKH‐67 stained small EVs.

**Results**: Small EVs were successfully isolated and verified. Compared with Electroporation and Incubation, Sonication is the most efficient method to incorporate chemotherapeutics into small EVs. However, Incubation led to more cellular uptake of the EV drugs, especially when the drugs encapsulated by the small EVs derived from HPNE cells. Furthermore, compared with Sonication and Electroporation, the EV drugs derived from Incubation is more efficacious in killing pancreatic cancer cells when applied at equivalent drug concentrations.

**Summary/Conclusion**: The efficiency of small EV encapsulation of chemotherapeutics varies among different loading methods, and the efficacy of EV encapsulated chemotherapeutics is associated with the loading methods, EV sources, and EV uptake efficiency.

### PS02.04 Development of Engineered extracellular vesicles with targeting peptide for specific delivery to pancreas tissue

Hiroaki Komuro, Department of Biomedical Engineering, Michigan State University

Yuki Harada, Institute for Quantitative Health Science and Engineering (IQ), Michigan State University

Shakhlo Aminova, Institute for Quantitative Health Science and Engineering (IQ), Michigan State University

Nathaniel Pascual, Institute for Quantitative Health Science and Engineering (IQ), Michigan State University

Christopher Contag, Department of Biomedical Engineering, Michigan State University

Masako Harada, Department of Biomedical Engineering, Michigan State University

**Introduction**: One of the main hurdles of gene therapy is to develop nonviral carriers for efficient and safe delivery. Extracellular vesicles (EVs) play an essential role in several physiological and pathological functions through intercellular communication. One advantage of using EVs as a carrier is the ability to engineer their surface to display targeting moieties. Here we introduce enriched delivery of engineered EVs displaying an organ targeting peptide specific to the pancreas.

**Methods**: Gene fusion of β‐cell‐specific recombinant peptide p88 to the EV‐binding domain of lactadherin (C1C2) allows the generation of EVs harboring the peptide on their surface upon transfection and isolation from HEK‐293T cells. EVs were isolated by differential ultracentrifugation and characterized using nanoparticle tracking analysis, immuno‐transmission electron microscopy (TEM), and western blot. To evaluate the targeting capacity of the EVs, Gaussia luciferase (gLuc) activity was measured in in vitro and in vivo using an in vivo imaging system (IVIS). The pDNA copy numbers recovered from each organ were calculated by qPCR.

**Results**: We successfully displayed the peptide on the EV surface and characterized the properties of the EVs. The presence of peptides did not affect the EV size and morphology. The immune‐TEM and western blot assay showed these EVs contained exosome marker (CD63) and C1C2 fusion protein (HA). We demonstrated higher binding of EVs to the pancreatic β‐cell line NIT1 in the presence of the peptide compared to the non‐β 4T1 cells using an in vitro bioluminescent assay. Furthermore, in vivo biodistribution assay following intravenous injection of EVs to mice showed enriched localization of the engineered EV in the pancreas. The pancreas of mice that received pancreas targeting p88‐EVs featured an accumulation of p88‐pDNA. These results indicate the importance of targeting peptide for the binding of EVs in both in vitro and in vivo models.

**Summary/Conclusion**: Our EV engineering technique is simple, robust, and efficient. This study demonstrates that small peptide‐based ligands can impart affinity to specific organs when displayed on the surface of EVs. We believe that the EV‐mediated targeted delivery will improve the development of therapeutics for human pancreatic diseases.

### PS02.05 Extracellular Vesicle‐Mediated siRNA Delivery, Protein Delivery, and CFTR Complementation in Well‐Differentiated Human Airway Epithelial Cells

Ashley Cooney, Department of Pediatrics, The University of Iowa

Sateesh Krishnamurthy, Department of Pediatrics, The University of Iowa

Patrick Sinn, Department of Pediatrics, The University of Iowa

Brajesh K. Singh, Department of Pediatrics, University of Iowa

**Introduction**: Primary cultures of well‐differentiated human airway epithelial cells (HAE) are a robust model for studying epithelial cell biology. However, well‐differentiated HAE cells are refractory to transfection techniques for delivering expression plasmids, small interfering RNA molecules (siRNA), and single‐stranded oligonucleotides. Viral‐based vectors (such as adenovirus, lentivirus, or adeno‐associated virus) are typically employed to deliver genetic material to HAE which can be expensive and time‐consuming to generate.

**Methods**: Exosomes (Exos) and microvesicles (MVs) were isolated from culture media of HEK‐293T or A549 cells using differential centrifugation. The exosomes were electroporated with siRNAs and their ability to deliver encapsulated siRNAs into well‐differentiated HAE was examined either by confocal microscopy or by QRT‐PCR. To deliver proteins, EVs were isolated from the conditioned media of A549 cells transfected with an expression plasmid or transduced with an adenovirus expressing mCherry (Ad5‐mCherry) or cystic fibrosis transmembrane conductance regulator (Ad5‐CFTR). Delivery of functional CFTR to HAEs from CF human donors was examined by measuring the change in anion channel activity in Ussing chambers.

**Results**: Our results suggest that exosomes are readily taken up by the cells in well‐differentiated HAE and they can efficiently deliver siRNA. We used both MVs and exosomes to deliver proteins in HAE. Although only MVs were capable of delivering large proteins like CFTR. We showed MV‐mediated delivery of functional CFTR protein to correct the transepithelial Cl' current in well‐differentiated HAE from CF donors.

**Summary/Conclusion**: Our study showed the potential use of EVs in manipulating the gene expression in an important in vitro airway model system. Exosomes effectively deliver siRNAs to modulate endogenous gene expression, whereas MVs can be used to deliver large protein cargos. Our data show that EVs can provide a rapid, inexpensive, and robust tool to deliver small RNAs and proteins into an important model system.

### PS02.06 Platelet derived‐extracellular vesicles as drug delivery system of anti‐cancer agents targeting glioblastoma

Deng‐Yao Lee, Graduate Institute of Biomedical materials & Tissue Engineering, Taipei medical University

Yu‐Wen Wu, Graduate Institute of Biomedical materials & Tissue Engineering, Taipei medical University

Ariane Sharif, University of Lille, Lille, France · Lille Neuroscience and Cognition Research Center

Thierry Burnouf, Professor, PhD, Graduate Institute of Biomedical materials & Tissue Engineering, Taipei medical University

**Introduction**: Extracellular vesicles (EVs) are physiologically instrumental for intercellular communications. They may also be of great interest for clinical applications as targeted drug delivery system (TDDS). EVs derived from platelets (PEVs), which express various membrane glycoprotein markers interacting with cancer cells, may be of particular value as a DDS able to target and be retained within the tumor microenvironment. However, methods to generate PEVs from platelets and to prepare drug‐loaded PEVs still need to be developed and optimized. In this study we evaluated various methodologies for generating PEVs. We also studied the capacity of the PEVs to be internalized by glioblastoma cells.

**Methods**: Therapeutic‐grade human platelet concentrates were centrifuged to pellet the platelets (PLTs). Platelets were resuspended in platelet additive solution (PAS) with 6% Dimethyl sulfoxide (DMSO) for frozen storage. PEVs were generated using 5 different methods: thrombin activation (0.1 U/ml; 37°C; 1 hr); sonication (40 kHz; 30 min); glass bead activation (37°C; 1 hr); freeze/thaw treatment activation (3 cycles of ‐80°C/1hr, 37°C/3 min); and room temperature incubation (20‐22°C; 1 hr). Particle sizes of PEVs were characterized by Dynamic light scattering (DLS), nanoparticle tracking analysis (NTA) and Tunable resistive pulse sensing (TRPS; Q‐nano), and concentration by NTA and TRPS. PEVs membrane markers expression was determined by western blot. 5‐(and‐6)‐Carboxyfluorescein Diacetate, Succinimidyl Ester (5(6)‐CFDA, SE) was used to stain PEVs and observe their internalization by U87MG glioblastoma cells using fluorescent microscopy.

**Results**: The particle sizes of PEVs generated by all five methods were in the range 180–200 nm. There were approximately 510 PEVs produced from each PLT when using the freeze‐thaw procedure, compared to less than 200 for the four other methods. All types of PEVs expressed CD41, CD62p, CXCR4, CD9 and CD63 glycoprotein markers. All 5 types of PEVs stained by 5(6)‐CFDA fluorescent dye could be internalized by U87MG cells within 24hrs of incubation and were found to accumulate near the nucleus.

**Summary/Conclusion**: All procedures evaluated (thrombin activation; sonication; glass beads activation, freeze and thaw; and room temperature incubation) can generate PEVs with similar size and membrane marker expression. The highest number of PEVs generated per naïve PLT was achieved by the freeze‐thaw procedure. All types of PEVs were able to be internalized by U87MG cells. Further studies will be carried out to select the optimal procedure for anti‐cancer agent loading within PEVs, and to determine the cytotoxicity against various glioblastoma cell lines.

### PS02.07 Bovine milk‐derived extracellular vesicles as potential nanocarriers of bioactive miRNAs

Lorena del Pozo‐Acebo, IMDEA Food

María del Carmen López de las Hazas, IMDEA Food

Almudena García‐Ruiz, IMDEA Food

Alberto Dávalos, IMDEA Food

**Introduction**: MicroRNAs (miRNAs) are small non‐coding RNAs with a known role as mediators in crucial biological processes, which converts them into high potential powerful candidates for therapeutic intervention. However, circulating miRNAs are unstable and rapidly degraded by endogenous enzymes, diminishing the possibility of successfully exerting a biological function in distant target cells. To achieve the therapeutic potential of miRNAs, efficient, tissue‐specific and nonimmunogenic delivery technologies must be developed. Since the discovery that miRNAs are naturally transported within exosomes, a type of extracellular vesicles that confer protection against RNase digestion and increase miRNA stability, exosomes have been proposed as delivery vehicle for miRNA‐based therapy.

**Methods**: In this study, we aimed to evaluate the use of milk‐derived extracellular vesicles as potential vehicle for extracellular RNA drug delivery. With this purpose, exosomes were isolated from raw bovine milk, combining ultracentrifugation and size exclusion chromatography (SEC) methodology. Isolated exosomes were then loaded with exogenous hsa‐miR148a‐3p, a highly expressed miRNA in milk exosomes. The suitability of exosomes as delivery vehicles for extracellular RNAs was tested by evaluating the absorption of miR‐148a‐3p in hepatic (HepG2) and intestinal (Caco‐2) cell lines. The potential exertion of a biological effect by miR‐148a‐3p was assessed by gene expression analysis, using microarrays.

**Results**: In‐vitro uptake analysis of exogenous hsa‐miR‐148a‐3p (loaded into bovine milk exosomes) revealed statistically significant concentration increases in both HepG2 and Caco‐2 cell lines. The increment found between 2h and 24h of exosome exposure suggests that the absorption of bovine milk‐derived exosomes is time dependent in these cells. Furthermore, the miRNA transported within exosomes can exert a biological effect through the modulation of gene expression.

**Summary/Conclusion**: Results support that bovine milk is a cost‐effective source of exosomes which can be used as nanocarriers of bioactive miRNAs with a potential use in RNA‐based therapy.

### PS02.08 Characterization, Biodistribution and Functional Studies of Immune‐Therapeutic Exosomes: Implications for Acute Respiratory Distress syndrome and COVID19

Mahmoud Elashiry, DDS, MDS, MD, PhD, Augusta University

Ranya Elsayed, DDS, MBA, PhD, Augusta University

Christopher cutler, DDS, PhD, Augusta university

**Introduction**: Dendritic cell (DC)‐derived exosomes (DC EXO), natural nanoparticles of endosomal origin, are under intense scrutiny in clinical trials for various inflammatory diseases. DC EXO are eobiotic, meaning they are well‐tolerated by the host; moreover, they can be custom‐tailored for immune‐regulatory or ‐stimulatory functions, thus presenting attractive opportunities for immune therapy. Previously we documented the efficacy of immunoregulatory DCs EXO (regDCs EXO) as immunotherapy for inflammatory bone disease, in an in‐vivo mice model. We showed a key role for encapsulated TGFB1 in promoting a bone sparing immune response. However, the on‐ and off‐target effects of these therapeutic regDC EXO and how target signaling in acceptor cells is activated is unclear.

**Methods**: In the present report, therapeutic murine regDC EXO were analyzed by high throughput proteomics, with non‐therapeutic EXO from immature DCs and mature DCs as controls, to identify shared and distinct proteins and potential off‐target proteins, as corroborated by immunoblot. Live animal imaging using SPECT/CT was done to track the biodistribution of regDCs EXO injected via tail vein of Black 6 mice. Mechanistic and functional studies were done to explore the mode of action of regDCs EXO on acceptor cells Invitro.

**Results**: The predominant expression in regDC EXO of immunoregulatory proteins as well as proteins involved in trafficking from the circulation to peripheral tissues, cell surface binding, and transmigration, prompted us to investigate how these DC EXO are biodistributed to major organs after intravenous injection. Invivo imaging showed preferential accumulation of regDCs EXO in the lungs, followed by spleen and liver tissue. In addition, TGFB1 in regDCs EXO sustained downstream signaling in acceptor DCs. Blocking experiments suggested that sustaining TGFB1 signaling require initial interaction of regDCs EXO with TGFB1R followed by internalization of regDCs EXO with TGFB1‐TGFB1R complex. Finally, these regDCs EXO that contain immunoregulatory cargo and showed biodistribution to lungs could downregulate the main severe acute respiratory syndrome coronavirus 2 (SARS‐CoV‐2) target receptor, ACE2 on recipient lung parenchymal cells via TGFB1 in‐vitro.

**Summary/Conclusion**: These results in mice may have important immunotherapeutic implications for lung inflammatory disorders and COVID19 induced acute respiratory distress syndrome.

### PS02.09 GE11‐expressing bovine milk‐derived extracellular vesicles for EGFR targeted delivery of oxaliplatin to colorectal cancer

Gyeongyun Go, Department of Biochemistry, Soonchunhyang University College of Medicine, Cheonan 31151, Korea

Sang Hun Lee, Department of Biochemistry, Soonchunhyang University College of Medicine, Cheonan, 31151, Republic of Korea

**Introduction**: Anticancer drugs, such as fluorouracil, oxaliplatin are commonly used to treat colorectal cancer. However, owing to their low response rate and adverse effects, the development of efficient drug delivery systems is required. Bovine milk derived extracellular vesicles (milk EV) can be a potential drug delivery systems since they can be obtained with a large amount and are known to be safe after system administration. However, studies for surface modification of milk EV are limited.

**Methods**: Milk EV were isolated by using differential centrifugation and ultracentrifugation. To display GE11 peptide onto the surface of milk EV, the cholesterol‐PEG‐DBCO was incorporated into the membrane of milk EV. Then, azide‐modified GE11 peptides were treated to the DBCO‐modified milk EV. The GE11‐expressing milk EV (GE11‐milk EV) were loaded with oxaliplatin using simple incubation and the amount of loaded oxaliplatin was quantified using ICP‐MS.

**Results**: GE11‐Milk EV were spherical nanoparticles with an average diameter of 200 nm. GE11‐milk EV showed superior drug delivery efficiency to EGFR overexpressing colorectal cancer cells and breast cancer cells compared to bare milk EV. In the colorectal cancer xenograft mouse model, intravenous administration of oxaliplatin‐loaded GE11‐milk EV resulted in greater inhibition of tumor growth as compared to the treatment of equivalent amount of free oxaliplatin.

**Summary/Conclusion**: In this study, we demonstrated the surface modification of milk EV with GE11 peptide using DBCO‐azide click chemistry reaction. The oxaliplatin‐loaded GE11‐milk EVs showed excellent antitumor effects and have the potential to replace existing anticancer drugs, such as 5‐FU, oxaliplatin.

### PS02.10 BioDrone, a novel drug delivery platform: From the basic science to potential therapeutic promises

Dong Woo Han, MDimune Inc

Jinhee Park, MDimune Inc

Jinju Lee, MDimune Inc

Jun‐Sik Yoon, MDimune Inc

Dayeon Kim, MDimune Inc

Hui‐Chong Lau, MDimune.Inc

Jeong Seon Yoon, MDimune Inc

Seung Wook Oh, PhD, MDimune Inc

**Introduction**: Previously, we have successfully demonstrated the establishment of a manufacturing‐scale extrusion process to allow the production of a large number of nanovesicles, cell‐derived vesicles (CDVs). Here, we present the compatibility of extrusion technology to produce CDVs from multi‐cell sources, including surface engineered cells. Productivity, one of the unique advantages of BioDrone technology, was evaluated at a single particle level. The therapeutic potential of CDVs was assessed by examining key characteristics as a drug carrier in vitro and in vivo.

**Methods**: Different cell sources such as immune cells, mesenchymal stem cells, red blood cells, and others were used to produce CDVs by a serial extrusion. CDVs produced from these cells were subject to a single particle analysis using flow cytometry. Additionally, intracellular uptake and trafficking of CDVs and their RNA cargos were visualized using the confocal microscope. In vivo biodistribution and tissue penetration of CDVs were assessed in mice as well. All the experiments were conducted in comparison with exosome or other drug carriers

**Results**: The extrusion efficiently produced CDVs from various cell sources with consistent quality. Comprehensive single particle analysis revealed genuine productivity of CDVs and conservation of key surface markers of cells, including engineered components. Robust encapsulation of small RNAs into CDVs produced from different cell sources showed promising therapeutic potential of BioDrone as a drug carrier. Additionally, efficient cellular uptake and tissue penetration of CDVs are presented here.

**Summary/Conclusion**: We have demonstrated that the extrusion technology enables the mass production of CDVs from various cell sources. This study highlights the expandability and versatility of BioDrone platform technology and its therapeutic potential as an innovative drug carrier in broad areas.

### PS02.11 Cellular response to a Listeriolysin O mutant Y406A

Apolonija Bedina Zavec, National institute of Chemistry

Rebeka Podgrajšek, National Institute of Chemistry, Department of Molecular Biology and Nanobiotechnology

Ana Špilak, National Institute of Chemistry, Department of Molecular Biology and Nanobiotechnology

Matic Kisovec, Department of Molecular Biology and Nanobiotechnology, National Institute of Chemistry

Maja Jamnik, National Institute of Chemistry, Department of Molecular Biology and Nanobiotechnology

Veronika Kralj‐Iglič, Laboratory of Clinical Biophysics, Faculty of Health Sciences, University of Ljubljana

Gregor Anderluh, National Institute of Chemistry, Department of Molecular Biology and Nanobiotechnology

Marjetka Podobnik, National Institute of Chemistry, Department of Molecular Biology and Nanobiotechnology

**Introduction**: Listeriolysin O (LLO) is a toxin from the intracellular pathogen Listeria monocytogenes, which forms pores in cholesterol‐rich lipid membranes of host cells. Large β‐barrel pores formed by LLO indicate significant plasticity, from arc‐ or slit‐shaped pores to supramolecular assemblies generating large defects in membranes. LLO has pH optimum at pH 5.5, a condition found in late endosomes, while also at neutral pH it can bind to the membrane and form pores, and damage cells. LLO mutant protein Y406A with specific activity at acidic pH that could be interesting for the applications in medicine and biotechnology was generated by our group. Mutant Y406A with substitution (Try to Ala) at the site 406 is able to bound to membranes and oligomerized similarly to the wtLLO, but the final membrane insertion step requires acidic pH. Mutant Y406A has pH optimum at pH 5–6. The cytotoxicity and the release of extracellular vesicles (EVs) was used to examine the response of the cells to LLO and its mutant Y406A.

**Methods**: The cell line K562 was used. Cells were incubated with LLO or its mutant Y406A for 30 min at 37°C. The cytotoxicity was measured by quantifying cellular membrane integrity (propidium iodide and trypan blue staining), cell viability test (Presto Blue), and cell apoptosis. ROS (reactive oxygen species)‐generation activity was also measured. EVs were isolated by differential centrifugation. The concentration of larger EVs in the samples was measured by flow cytometry using Annexin‐FITC. The concentration of smaller EVs in the samples were measured by DLS.

**Results**: The effects of wtLLO and its mutant Y406A were tested on myelogenous leukemia cell line K562, which is highly sensitive in vitro target for the natural killer cells. The cytolethal concentration of wtLLO was between 1 nM and 10 nM. Vesiculation level was increased at cytolethal concentration and at 10‐fold lower concentration than cytolethal. At 100‐fold lower concentrations than cytolethal, the effect was reversed and cells shedding less EVs than control cells. On the other hand, the viability of cells was not affected after treating with Y406A at neutral pH, even at high concentrations of protein, while at pH 6.0 Y406A showed almost the same citotoxicity as wtLLO at pH 7.4. However, a low pore‐forming activity was detected at higher concentrations of Y406A (100 nM) at neutral pH. Besides, the level of EV secretion was slightly increased at higher concentrations of protein (100 nM) at neutral pH.

**Summary/Conclusion**: Mutant Y406A is significantly less toxic than wtLLO under physiological conditions and becomes toxic under acidic conditions; this makes it a potential candidate for stimuli responsive applications and cancer treatment.

### PS02.12 Good things come in small packages: a new hope for preterm babies brains

Mhoyra Fraser, PhD, The University of Auckland

Teena K. Gamage, The University of Auckland

Sam Mathai, PhD, The University of Auckland

**Introduction**: Oxygen deprivation occurring in the womb or during delivery leads to permanent functional impairment of the brain and is one of the most common challenges faced by infants born preterm. Because of major advances in neonatal intensive care, survival of these preterm infants with brain injury has increased significantly. Extracellular vesicles (EVs), are naturally capable of penetrating the blood brain barrier, and can communicate with the microenvironment through transfer of proteins, miRNA and other nucleic acids. Importantly, they have an intrinsic neuroprotective therapeutic activity and are ideal candidates to deliver targeted therapeutic molecules of choice through modifications to enhance delivery. Using our well‐established fetal sheep model of preterm brain injury, we sought to examine the intrinsic therapeutic potential of unmodified human fetal neural stem cell‐derived extracellular vesicles (hFNSC‐EVs) to ameliorate injury.

**Methods**: Fetal sheep at 0.7 gestation (day 103–104; term ∼145 days) received intranasal infusions of sterile hFNSC‐EVs (hFNSC‐EVs‐occlusion) or vehicle (vehicle‐occlusion and vehicle sham‐occlusion) commencing 60 minutes following a 25 minute umbilical cord occlusion (UCO). After 3 days recovery in utero, ewes were killed and fetal brains collected for histopathology.

**Results**: Umbilical cord occlusion was associated with significant brain injury to areas commonly affected by asphyxia in preterm infants. We are currently comparing treatment outcomes to determine whether intranasal delivery of hFNSC‐EVs can improve survival of immature and mature oligodendrocytes (Oligo2, CNPAse) and if so does this occur in association with reduced apoptosis and astrogliosis within white matter regions of the injured preterm brain.

**Summary/Conclusion**: To the best of our knowledge, this study is the first to determine the usefulness of intranasal delivery of unmodified hFNSC‐EVs in a fetal sheep model of preterm brain injury. Our study has the potential to revolutionise treatment strategies and offer the possibility for amelioration and recovery in the injured preterm brain.

## PS03 Metabolism, diabetes and cardiovascular diseases

Chair: Aleksandra Gasecka, Medical University of Warsaw, Poland

Chair: Mohsin Khan, Temple University, United States

### PS03.01 Human umbilical cord mesenchymal stem cells derived exosomes alleviate diabetic retinopathy by delivering miR‐5068 and miR‐10228 to regulate HIF‐1α/PGC‐1α pathway

Fengtian Sun, Jiangsu University

Hui Qian, jiangsu university

Wenrong Xu, Jiangsu University

**Introduction**: Diabetic retinopathy (DR) that is a leading cause of vision decline and blindness in adults still lacks of satisfactory treatments. Human umbilical cord mesenchymal stem cells derived exosomes (hucMSC‐Ex) are considered as novel therapeutic approaches and promising nanomaterials in regenerative medicine. Engineered exosomes have become an important approach to improve the curative effect.

**Methods**: In this study, we established a streptozocin‐induced DR rat model to evaluate the effect of hucMSC‐Ex on the repair of retinal damage. Meanwhile, human retinal microvascular endothelial cells (hRMECs) and adult retinal pigment epithelial cell line‐19 (ARPE‐19) were stimulated by 30 mM glucose medium to induce the high glucose environment in vitro followed by the treatment of hucMSC‐Ex to study the therapeutic mechanism. Therapeutic miRNAs were loaded into exosomes by electroporation.

**Results**: HucMSC‐Ex ameliorated hyperglycemia‐induce retinal injury by relieving apoptosis, inflammation, oxidative stress and pathological angiogenesis. Mechanistically, high glucose induced the increased expression and nuclear localization of hypoxia inducible factor‐1α (HIF‐1α) that inhibited peroxisome proliferator‐activated receptorγcoactivator‐1α (PGC‐1α) activation by binding with enhancer of zeste homolog 2 (EZH2) to accelerate DR progression. HucMSC‐Ex delivered miR‐5068 and miR‐10228 to reverse the upregulation of HIF‐1α and promote the expression of PGC‐1α, thereby exerting therapeutic functions in DR. Importantly, engineered hucMSC‐Ex loaded with mimics of miR‐5068 and miR‐10228 by electroporation further enhanced retinal repairing effects.

**Summary/Conclusion**: HucMSC‐Ex alleviate DR by transporting miR‐5068 and miR‐10228 to regulate HIF‐1α/PGC‐1α pathway, and engineered hucMSC‐Ex with miR‐5068 and miR‐10228 further strengthen the therapeutic outcome.

### PS03.02 Effect of adverse pregnancy events on the protein cargo and surface marker distribution of human amniotic epithelial cell derived Extracellular Vesicles

Mehri Barabadi, Ritchie Centre

Naveen Kumar, Ritchie Centre

Gina D. Kusuma, PhD, Ritchie Centre

Dandan Zhu, Ritchie Centre

David W Greening, Baker Institute

Rebecca Lim, Ritchie Centre

**Introduction**: Human amniotic epithelial cell (hAEC) derived extracellular vesicles (EVs) have shown to exhibit therapeutic potentials such as immunomodulatory and repair properties. Adverse pregnancy events reflecting placental dysfunctions can induce changes in placental EVs. This study aimed to investigate the effect of pregnancy complications such as hypertension, diabetes, intrauterine growth retardation (IUGR), and preeclampsia on the protein cargo and surface markers of hAEC‐derived EVs.

**Methods**: hAECs were isolated from healthy term placenta (n = 6) as well as pregnancy complications such as Gestational Diabetes Mellitus on diet (GDM+D, n = 4), GDM on insulin (GDM+I, n = 5), hypertension (n = 5), preeclampsia (n = 4), and IUGR (n = 2). The cells were cultured and EVs were isolated using tangential flow filtration (TFF) coupled with size exclusion chromatography (SEC) techniques. The protein composition of EVs was characterised using mass spectrometry analysis. Furthermore, single interferometric reflectance imaging sensing (IRIS) technology was used to characterise EVs positive for tetraspanins CD9, CD81, and CD63 to determine size distribution and particle counts.

**Results**: IRIS technology indicated that size distribution and quantity of EVs present with CD63, CD81, CD9 tetraspanins were not significantly affected by the pregnancy complication events. Differentially expressed proteins (DEPs) were identified using mass spectrometry analysis by conducting Welch's t‐test. The DEPs from each group were subjected to functional enrichment analysis and gene sets corresponding to identified proteins were annotated using Reactome, KEGG, and Gene Ontology (GO) databases. We observed an over‐representation of DEPs involved in dysregulated neutrophil‐mediated immunity and leukocyte activation in the Preeclampsia group. We also observed dysregulating ECM organisation in EVs from GDM+I pregnancies and those affected by pregnancy‐associated hypertension. Furthermore, we observed an over‐representation of DEP associated with the dysregulation of cellular metabolic process in EVs from pregnancies in the GDM+D group.

**Summary/Conclusion**: In summary, these preliminary data suggest that pregnancy complications do not have a significant effect on the distribution and particle numbers of hAEC‐EVs, however, proteomic profiling in pregnancy complication groups changes compared to the healthy control.

### PS03.03 Isolation and proteome profiling of plasma‐derived extracellular vesicles from the non‐obese diabetic (NOD) mouse

Isabel M. Diaz lozano, Karolinska Institutet

Helena Sork, PhD, Institute of Technology, University of Tartue

Virginia Stone, PhD, Karolinska Institutet

Maria Eldh, Karolinksa Institutet

Susanne Gabrielsson, Karolinska Institutet

Malin Flodström ‐Tullberg, Professor, Karolinska Institutet

**Introduction**: The mechanism(s) through which pancreatic beta‐cells are destroyed in type 1 diabetes (T1D) remain to be fully understood but may comprise of more than one distinct pathophysiological mechanism (known as T1D endotypes). Discovery of one or several endotype‐specific biomarkers during the pre‐diabetic period may open up the potential of personalized disease interventions. Extracellular vesicles (EVs) may harbor biological markers reflecting the pre‐diabetic stages of T1D. Here, we describe the enrichment and characterization of EVs from plasma collected from non‐obese diabetic (NOD) mice, a model for T1D.

**Methods**: EVs were enriched from prediabetic NOD mouse plasma by size exclusion‐chromatography (SEC) and membrane affinity purification (MA) based methods. EVs were characterized by nanoparticle trafficking analysis (NTA), protein concentration measurements, transmission electron microscopy (TEM) and LC‐MS/MS analysis, before or after affinity‐based depletion of highly abundant plasma proteins.

**Results**: SEC enriched a larger number of particles exhibiting a “characteristic” cup‐shaped EV morphology than MA. MA‐purified EV samples had a higher protein content and relatively higher levels of proteins that are abundant in plasma as compared to SEC‐purified EVs. The column‐based depletion successfully removed abundant plasma proteins, but the protein yield obtained by MS increased only in whole plasma samples and not in EV samples. An optimized LC‐MS/MS based analysis of SEC‐enriched EVs yielded a total of 680 proteins including canonical EV markers.

**Summary/Conclusion**: SEC can be employed to enrich EVs from NOD mouse plasma. The established method may be used to identify new biomarkers specific for the prediabetic stage, thereby serving as a tool to differentiate disease endotypes and patient stratification in intervention studies.

### PS03.04 Single‐cell sequencing analysis the role of hucMSC exosomes in inhibiting renal interstitial fibrosis and preventing diabetic kidney diseases

cheng ji, jiangsu university

jiahui zhang, jiangsu university

Hui Qian, jiangsu university

**Introduction**: Diabetic kidney diseases (DKD) are characterized by progressive fibrosis and lead to the end‐stage renal disease (ESRD). mesenchymal stem cells (MSC) exosomes have obvious repair effects in acute and chronic kidney injury. Based on 10x genomics single‐cell sequencing technology, this article aims to explore the molecular mechanism of human umbilical cord mesenchymal stem cells derived exosomes (hucMSC‐Ex) to inhibit renal fibrosis and delay DKD progression.

**Methods**: A T2DM rat model was constructed with 45% high‐fat diet combined with streptozotocin (STZ, 35mg/kg) tail vein injection. Then injected with PBS as control, the experimental group was injected with hucMSC‐Ex (10mg/kg, every 3 days/time) through the tail vein to observe the therapeutic effect in DKD. Collect and separate kidney tissue single‐cell suspensions for 10x genomics single‐cell sequencing to analyze the changes in renal cell communities, numbers and gene levels in the state of diabetic nephropathy. It was found that hucMSC‐Ex could promote the increase of ubiquitin molecules bound by YAP, and the CK1δ/β‐TRCP kinase ubiquitin system with significantly high expression in hucMSC‐Ex was screened by protein profiling analysis. and promote its ubiquitination degradation.

**Results**: Through 10x genomics single‐cell sequencing analysis, it was found that the number of macrophage colony cells in the kidney tissue of DKD rats increased significantly. By activating the TGF‐β1/Smad2/3/YAP signal axis, it promotes the change of mesangial cells to myofibroblast‐like cells, and tissue immunity Fluorescence and histochemical experiments found that YAP protein in kidney tissue was significantly activated, and the expression of α‐SMA increased and the progression of fibrosis was aggravated. The hucMSC‐Ex treatment inhibited the decrease of YAP level and α‐SMA expression in mesangial cells. HucMSC‐Ex treatment resulted in significant up‐regulation of Ser381‐YAP and Ser127‐YAP in the cytoplasm. YAP decreases. HucMSC‐Ex is rich in CK1δ/β‐TRCP kinase ubiquitin system, which promotes YAP ubiquitination degradation and inhibits YAP signaling pathway, improves renal fibrosis and delays the process of diabetic nephropathy.

**Summary/Conclusion**: The DKD associated macrophages promoted the transformation of mesangial cells into myofibroblast‐like cells by activating the TGF‐β1/Smad2/3/YAP signal axis to accelerate the progression of renal interstitial fibrosis. And hucMSC‐Ex promotes the YAP ubiquitination and degradation by delivering the CK1δ/β‐TRCP kinase ubiquitin system to inhibit renal interstitial fibrosis, improve renal function and delay the progression of DKD. This experiment provides a new treatment strategy and experimental basis for the treatment of DKD with mesenchymal stem cell exosomes.

### PS03.05 Lipopolysaccharide‐enriched small extracellular vesicles from metabolic syndrome patients trigger endothelial dysfunction by activation of Toll Like Receptor 4

Sakina Ali – INSERM U1063

Marine Malloci – INSERM U1063

Zainab Safiedeen – INSERM U1063

Raffaella Soleti – Engineer, INSERM U1063

Luisa Vergori – Engineer, INSERM U1063

Xavier Vidal Gómez – INSERM U1063

Charlène Besnard – INSERM U1063

Séverine Dubois – INSERM U1063, Centre Hospitalo‐Universitaire d'Angers

Frederic Gagnadoux – INSER U1063, Centre Hospitalo‐Universitaire d'Angers

Soazig Le Lay – INSERM U1063

Jérôme Boursier – Centre Hospitalo‐Universitaire d'Angers

Arnaud Chevrollier – CNRS 6015, INSERM U1083, Centre Hospitalo‐Universitaire d'Angers

Gilles Simard – INSER U1063, Centre Hospitalo‐Universitaire d'Angers

Ramaroson Andriantsitohaina – Director of Research INSERM, INSERM U1063 SOPAM

Paul Calès – Metabol study Group, Centre Hospitalo‐Universitaire d'Angers

Frédéric Oberti – Metabol study Group, Centre Hospitalo‐Universitaire d'Angers

Isabelle Fouchard‐Hubert – Metabol study Group, Centre Hospitalo‐Universitaire d'Angers

Adrien Lannes – Metabol study Group, Centre Hospitalo‐Universitaire d'Angers

Ingrid Allix – Metabol study Group, Centre Hospitalo‐Universitaire d'Angers

Pierre‐Henri Ducluzeau – Metabol study Group, Centre Hospitalo‐Universitaire d'Angers

Wojciech Trzepizur – Metabol study Group, Centre Hospitalo‐Universitaire d'Angers

Nicole meslier – Metabol study Group, Centre Hospitalo‐Universitaire d'Angers

Pascaline Priou – Metabol study Group, Centre Hospitalo‐Universitaire d'Angers

Samir Henni – Metabol study Group, Centre Hospitalo‐Universitaire d'Angers

Georges Leftheriotis – Metabol study Group, Centre Hospitalo‐Universitaire d'Angers

Pierre Abraham – Metabol study Group, Centre Hospitalo‐Universitaire d'Angers

Christophe Aubé – Metabol study Group, Centre Hospitalo‐Universitaire d'Angers

Gilles Hunault – Metabol study Group, Centre Hospitalo‐Universitaire d'Angers

Odile Blanchet – Metabol study Group, Centre Hospitalo‐Universitaire d'Angers

Belaid sekour – Metabol study Group, Centre Hospitalo‐Universitaire d'Angers

Jean‐Marie Chrétien – Metabol study Group, Centre Hospitalo‐Universitaire d'Angers

Carmen Martínez – research scientist, INSER U1063, Centre Hospitalo‐Universitaire d'Angers

**Introduction**: Metabolic syndrome (MetS) is characterized by a cluster of interconnected risk factors leading to an increased risk of cardiovascular events. Small extracellular vesicles (sEVs) can be considered as new biomarkers of different pathologies, and they are involved in intercellular communication. Here, we hypothesize that sEVs is implicated in MetS‐associated endothelial dysfunction.

**Methods**: Circulating sEVs of non‐MetS subjects and MetS patients were isolated from plasma and characterized. Thereafter, sEVs effects on endothelial function were analyzed by measuring nitric oxide (NO) and reactive oxygen species (ROS) production and mitochondrial dynamic proteins, on human endothelial aortic cells (HAoECs).

**Results**: Circulating levels of sEVs positively correlated with anthropometric and biochemical parameters including visceral obesity, glycaemia, insulinemia, and dyslipidemia. Treatment of HAoECs with sEVs from MetS patients decreased NO production through the inhibition of the endothelial NO‐synthase activity. Injection of MetS‐sEVs into mice impaired endothelium‐dependent relaxation induced by acetylcholine. Furthermore, MetS‐sEVs increased DHE and MitoSox‐associated fluorescence in HAoECs, reflecting enhanced cytosolic and mitochondrial ROS production which was not associated with mitochondrial biogenesis or dynamic changes. MetS patients displayed elevated circulating levels of LPS in plasma, and, at least in part, it was associated to circulating sEVs. Pharmacological inhibition and down‐regulation of TLR4, as well as sEV‐carried LPS neutralization, results in a substantial decrease of ROS production induced by MetS‐sEVs.

**Summary/Conclusion**: These results provide evidence that sEVs from MetS patients as potential new biomarkers for this syndrome and that activation of the TLR4 pathway by sEVs provides a link between the endothelial dysfunction and metabolic disturbances in MetS.

### PS03.06 Adipocyte‐Derived Exosome Induced Obesity‐Mediated Insulin Resistance and Lipotoxicity

Yujeong Kim, Division of Food and Nutrition, Chonnam National University

Ok‐Kyung Kim, Division of Food and Nutrition, Chonnam National University

**Introduction**: Obesity is directly or indirectly associated with the development of insulin resistance that causes type 2 diabetes. Recently, extracellular vesicle from adipose tissue has been shown to be involved in the development of insulin resistance. Here, we performed the isolation of exosomes from adipose tissue in obese conditions and investigated the effect of adipocytes‐derived exosome in the mechanism of insulin resistance development during obese conditions.

**Methods**: We isolated exosome from adipose tissue in normal diet‐treated mice or high fat diet‐induced obese mice, and confirmed the vesicle size, exosome markers, and uptake into differentiated C2C12 cells. We investigated whether exosome from adipose tissue in high fat diet‐induced obese mice induces insulin resistance and lipotoxicity in differentiated C2C12 cells.

**Results**: We found that the culture medium of differentiated 3T3‐L1 cells induced insulin resistance in differentiated C2C12 cells, whereas the culture medium from exosome inhibitor treated‐differentiated 3T3‐L1 cells improved insulin resistance. Exosome secretion was increased in adipose tissue from high fat diet‐induced obese mice, compared to normal diet. In addition, exosome from adipose tissue in high fat diet‐induced obese mice caused directly insulin resistance and lipotoxicity in differentiated C2C12 cells, compared to normal diet.

**Summary/Conclusion**: In conclusion, we suggest that adipocytes‐derived exosome in obese condition lead to insulin resistance and lipotoxicity in muscle tissue. This finding assessed the role played by exosome derived from adipocytes in the development of insulin resistance and potential strategies for the development of therapeutics aimed at obesity and metabolic disorders such as type 2 diabetes.

### PS03.07 Identification and quantification of key metabolites of the steroid hormone biosynthesis pathway in extracellular vesicles

Guillermo Bordanaba, CIC bioGUNE

Sebastiaan van Liempd, CIC bioGUNE

Diana Cabrera, CIC bioGUNE

Félix Royo, CIC bioGUNE

Juan Manuel Falcón‐Pérez, CIC bioGUNE

**Introduction**: Prostate cancer (PCa) is among the most frequently diagnosed type of cancer worldwide. The lack of sensitive diagnostic tools and knowledge in its mechanisms of emergence and progression are a major challenge. In this regard, the metabolomic analysis of PCa provides unprecedent pathophysiological information about metabolic changes and responses to the microenvironment in healthy and tumorigenic cells. Such data often remains hidden in genomics, transcriptomics and proteomics approaches. Moreover, extracellular vesicles (EVs) are heterogeneous lipid containers with a complex cargo of molecules that are a valuable asset for cell‐to‐cell communication and signalling. EVs play a key role in pathophysiological processes by actively triggering various genetic or metabolic responses. Remarkably, PCa cells secrete EVs that participate in driving tumour growth and metastasis towards healthy recipient cells by building up a local environment, which increases tumour chemotaxis. There is literature reporting the transport of metabolic components using EVs as carriers; however, the manner their cargo (metabolites and enzymes) interacts with PCa metabolism is yet to be properly described. In previous research from our group, an increased level of dehydroepiandrosterone sulphate (DHEAS) in urinary EVs derived from PCa patients samples was detected. Moreover, steroid‐related metabolites and enzymes have been reported as important modulators of PCa progression.

**Methods**: In this work, we have developed an assay for the extraction and simultaneous identification of eleven metabolites of the steroid hormone biosynthesis pathway from cellular matrices and EVs. The metabolites included as chemical standards are pregnenolone, pregnenolone sulphate, DHEA, DHEAS, androsterone, androsterone sulphate, aldosterone, cortisol, estrone, testosterone and dihydrotestosterone. The metabolites were extracted using a liquid biphasic method and this process was optimized by testing the effect of different solvent combinations.

**Results**: The identification method was performed using hydrophobic integration chromatography coupled with time‐of‐flight mass spectrometry (UPLC‐MS) and has a run time of 5 min. In addition, this chromatographic method was optimized by testing various phases and gradients over the run. So far, this UPLC‐MS method has successfully identified steroid‐related metabolites in a panel prostate cell lines (PC‐3, DU145, 22Rv1, LnCaP and BPH‐1). Furthermore, EVs derived from this panel of cells and EVs isolated from urine fluids have been assayed using the method.

**Summary/Conclusion**: In summary, we present an optimized and rapid assay for the extraction and identification of steroid‐related metabolites from cells and extracellular vesicles.

### PS03.08 Exercise modulation of Extracellular Vesicles’ content: focus on Nrf2 and antioxidant enzymes

Veronica Lisi, University of Rome Foro Italico

Chantalle Moulton, University of Rome Foro Italico

Ambra Antonioni, University of Rome Foro Italico

Elisa Grazioli, University of Rome Foro Italico

Cristina Fantini, University of Rome Foro Italico

Flavia Guidotti, University of Rome Foro Italico

Laura Capranica, University of Rome Foro Italico

Luigi Di Luigi, University of Rome Foro Italico

Paolo Sgro, University of Rome Foro Italico

Ivan Dimauro,University of Rome Foro Italico

Daniela Caporossi, University of Rome Foro Italico

**Introduction**: Extracellular vesicles (EVs) are lipid‐bound vesicles secreted by cells that mediate intercellular communication by shuttling functional molecules, such as different RNA species, lipids, DNA, and proteins. In literature it's well described that physical activity (PA) stimulates the release of molecules into circulation as EV cargo. The transcription NF‐E2‐related factor 2 (Nrf2) plays an important role in maintaining Redox homeostasis (RH) by regulating downstream antioxidants. The function of PA to trigger Nrf2, in response to the increase in ROS, is already known. Considering the role of RH in exercise‐induced signaling and adaptation, we focused on the exercise‐related intercellular communication of redox components mediated by EVs, including upstream and downstream factors.

**Methods**: Plasma EVs were isolated from trained and untrained healthy males (n = 14, 20–35 yrs) before and after (3 and 24 hours) an acute bout of endurance exercise (70% HRmax for 30’), or a short‐term endurance training (70% HRmax for 30’/day for 5 consecutive days), and analyzed for Nrf2, Catalase (CAT), Glutathione Peroxidase 1 (GPX1), Thioredoxin reductase 1 (TrxR1), Thioredoxin reductase 2 (TrxR2), Thioredoxin 2 (Trx2), SOD1, MnSOD (SOD2) and oxidative stress markers, as protein carbonilation and lipid peroxidation (4‐HNE), content.

**Results**: Our results showed that plasma EVs contain Nrf2 and antioxidant enzymes differently modulated by the fitness level and the exercise intervention. While no specific modulation was detected for the Nrf2 content in EVs, our data highlighted that SOD2 (p = 0.05) and CATALASE (p = 0.005) content in EVs’ cargo is decreased in trained with respect to the untrained subjects, with no significant effects exerted by a single acute exercise. When untrained subjects were submitted to 5 days of endurance training, CATALASE (p = 0.05) and SOD2 (p = 0.05) content were decreased, reaching levels similar to those found in trained subjects.

**Summary/Conclusion**: This study shows the presence of Nrf2 and antioxidant enzymes in plasma EVs, indicating a cross‐tissue molecular system to maintain and restore RH, and possibly to counteract, at systemic level, the oxidative stress derived by poor fitness levels or during physical exercise.

### PS03.09 Extracellular Vesicle‐derived Epigenetic Biomarkers for Early Type 2 Diabetes Diagnosis from Saliva

Poster Presenter

Ulrike Kegler, AIT Austrian Institute of Technology

Manuela Hofner, AIT Austrian Institute of Technology

Nathalie Ropek, AIT Austrian Institute of Technology

Anja Buhmann, AIT Austrian Institute of Technology

Michael Leutner, Medical University of Vienna

Alexandra Kautzky‐Willer, Medical University of Vienna

Julie Krainer, AIT Austrian Institute of Technology

Klemens Vierlinger,AIT Austrian Institute of Technology

Christa Noehammer, AIT Austrian Institute of Technology

**Introduction**: Saliva is a readily and even within short time intervals repeatedly available body fluid, which can be obtained via non‐invasive, painless collection. The aim of this study was to define salivary extracellular vesicle (EV)‐derived epigenetic biomarkers suitable for early Type 2 diabetes (T2D) diagnosis.

**Methods**: A diabetic patient cohort (T2D, pre‐diabetes, gestational diabetes, healthy) was recruited. Plasma and cell‐free saliva was collected and EVs thereof prepared. Genome‐wide DNA methylation profiling was performed using EV‐derived DNA on Illumina EPIC arrays, whereas EV‐RNA was used for small RNA sequencing after several library preparation as well as EV isolation kits had been evaluated.

**Results**: After having identified the best suited EV isolation kit as well as the in the body fluids cell‐free saliva and plasma best performing small RNA library preparation kit, a variety of smallRNA /miRNA candidate biomarkers could be identified. In addition, we were able to successfully run a genome‐wide DNA methylation study from only 100 ng of salivary EV DNA and identified corresponding DNA‐methylation based candidate biomarkers. Verification of the identified potential epigenetic biomarkers for T2D is currently ongoing and performed in independent patient samples.

**Summary/Conclusion**: This study once more demonstrated saliva to be a most promising sample matrix for disease diagnostics which by far is not limited to oral diseases. Further genome‐wide high‐end profiling technologies such as methylation bead arrays and small RNA Seq could be successfully applied to cell‐free body fluids despite low amount of circulating DNA and RNA present there.

### PS03.10 Extracellular Vesicles in Hypermobile Ehlers‐Danlos syndrome: deconstructing the fibroblast secretome to define bioactive molecules and disease mechanisms

Miriam Romano, Department of Molecular and translational Medicine‐University of Brescia, Italy

Nicoletta Zoppi, University of Brescia, Department of Molecular and Translational Medicine

Marco Giuseppe Ritelli, University of Brescia, Department of Molecular and Translational Medicine

Nicola Chiarelli, University of Brescia, Department of Molecular and Translational Medicine

Paolo Bergese, Dipartimento di Medicina Molecolare e Traslazionale, Università degli Studi di Brescia, 25123 Brescia, Italy

Annalisa Radeghieri, Department of Molecular and Tranlational Medicine‐Università degli Studi di Brescia

Marina Colombi, University of Brescia, Department of Molecular and Translational Medicine

**Introduction**: Hypermobile Ehlers‐Danlos syndrome (hEDS) is a multisystemic connective tissue disorder without known molecular bases. hEDS dermal fibroblasts show widespread extracellular matrix (ECM) disarray, including that of fibronectin (FN), increased levels of matrix metalloproteinase 9 (MMP9), and a myofibroblast‐like phenotype with α‐smooth muscle actin (α‐SMA) cytoskeleton organization. Control fibroblasts treated with hEDS conditioned media (CM) acquire this phenotype, indicating that patient cells’ CM contains key factors controlling this phenotypic switch. We hence dissected hEDS cells’ secretome into its nanoscale components, i.e., extracellular vesicles (EVs) and nanosized macromolecular complexes (nMC), to explore their possible role in the hEDS pathomechanisms.

**Methods**: Culture media of control and hEDS fibroblasts were treated by differential ultracentrifugation (0.8k g x 30’,16k g x 45’ and 100k g x 4h). Pellets and relative supernatants (SNs) were characterized according to the MISEV 2018 guidelines by AFM imaging, colorimetric nanoplasmonic assay (CONAN), and Western blotting (WB). Functional assays were performed by treating control fibroblasts with the different CM fractions from control and patient cells.

**Results**: We observed that both 16k and 100k control and hEDS pellets were composed of round‐shaped objects with average diameters < 150 nm. AFM imaging and CONAN assay confirmed the absence of exogenous protein contaminants. WB proved the presence of EVs and showed the presence of MMP9 in the 100k hEDS pellet. Functional assays revealed that the treatment of control cells with either 16k and 100k pellets or nMCs derived from hEDS cells partly induces the phenotypic switch (i.e., partial FN‐ECM degradation with few α‐SMA‐positive cells), whereas the different control CM fractions did not display any effect.

**Summary/Conclusion**: Overall, these data suggest that both EVs and nMCs fractions synergistically contribute to the hEDS fibroblast‐to‐myofibroblast transition.

### PS03.11 Purine metabolism: new regulator of senescence associated with the secretory phenotype mediated by exosome‐like particles

Juan Fafián‐Labora, Universidade da Coruña

Rocio Mato Basalo, Universidade da Coruña

Miriam López‐Morente, Universidade da Coruña

Onno Arntz, Radboudumc

Fons van de Loo, Radboud University Medical Centre

María C. Arufe, University of La Coruña

**Introduction**: Cellular senescence is a hallmark of ageing and characterized by cell cycle arrest and production of cytokines, interleukins, lipid mediators and extracellular vesicles. Exosome‐like particles are a group involved in the transmission of paracrine senescence and rejuvenation and they are an attractive target to therapeutic application. In the last years, the metabolic changes had revealed crucial in the senescence signature in ageing and premature ageing and our group has focused to determined what metabolism pathways were altered in Hutchinson‐Gilford progeria syndrome (HGPS). This disease is a very rare fatal disease characterized for accelerated aging. Because of that, we focused our study on how the alteration of purine‐metabolism could affect the SASP mediated by exosome‐like particles.

**Methods**: In this study, we work with several models of cellular senescence: oncogenic‐induced senescence and DNA‐damage induced senescence (DDis) in mesenchymal stem cells, chondrocytes and fibroblasts from human origin. During the senescence induction the medium was supplemented with S‐adenosyl‐methionine (SAMe), a metabolite that is an alternative source of purine and besides the exosome‐like particles from that senescent cell models were used to treat proliferative cells.

**Results**: It was observed that the production of exosome‐like particles characterized by NTA from senescence signature models were decreased in a significantly way. It was observed that the production of exosome‐like particles characterized by NTA from senescence signature models were decreased in a significantly way. Besides, the transmission of paracrine senescence, which occurs through exosome‐like particles, it is no happens when cells are treated with SAMe as indicated in our results on proliferation using crystal violet and b‐galactosidase activity.

**Summary/Conclusion**: Altogether, our data suggests that purine metabolism is altered in premature aging through SASP mediated by exosome‐like particles. This opens a new window for the therapeutic treatment of the age‐related disease and HGPS

### PS03.12 Endothelial shear stress affects extracellular vesicles release and biodistribution

Cecile Devue, Inserm U970‐ Paris Cardiovascular Research Center

Michael Robillard, Inserm U970‐ Paris Cardiovascular Research Center

Pierre‐Michaël Coly, Université de Paris, INSERM U970, Paris Cardiovascular Research Centre, Paris, France

Chantal Boulanger, Inserm U970‐ Paris Cardiovascular Research Center

Xavier LOYER, INSERM U970‐PARCC

**Introduction**: Atherosclerosis initiates at endothelial level at specific areas where blood flow is low and disturbed contributing to the recruitment of inflammatory cells. Extracellular vesicles (EVs) emerged as new mediators of intercellular crosstalk. Additionally endothelial atheroprone conditions (shear stress‐ SS) have been showed to influence large EVs release. During their formation, EVs package and carry some material, especially miRNAs, from originating cells to recipient cells, in which they contribute to specific phenotypical changes through regulation of gene expression. To date, mechanisms linking EVs‐mediated transfer of information onto the development of atherosclerosis have not been characterized so far. We hypothesize that endothelial‐derived EVs can selectively be released as function of endothelial shear stress and transferred to the spleen given its recently recognized importance in regulating the immune response in atherosclerosis.

**Methods**: Confluent mouse endothelial cells (SVEC) were exposed to either Low SS or High SS for 24 hours in a serum free medium. Large (>200 nm) and small EVs were isolated from conditioned medium by sequential centrifugation; they were then characterized by Western blot and tunable resistive pulse sensing. In vitro EV biodistribution to splenocytes was assessed after co‐culture for 12hours by flow cytometry using labelled EVs with Vybrant DID (VD) or Membright. miRNAs content have been analyzed by microarray and qPCR.

**Results**: Levels of large and small EVs were not modulated by low SS conditions. In response to High SS conditions EVs " both large and small " levels were further increased to LSS conditions. No changes in EVs size were observed as a function of SS levels. Western blot analysis revealed that EV markers were not affected by SS conditions. Labelling EVs with VD or Membright do not alter EVs size, concentration and EVs markers expression. Analysis of EVs biodistribution co‐cultured with splenocytes revealed that large EVs derived from low SS conditions are preferentially transferred to myeloid cells including macrophages, monocytes and neutrophils as compared to EVs derived from high SS. Small EVs do not exhibit any specific tropism and are poorly transferred to cells. EVs miRNAs content analysis revealed that EVs selectively contain miRNAs into large and small EVs. Among such miRNAs, we showed that miR‐24 is selectively packaged into large EVs upon LSS. In vitro studies showed that specific miR‐24 transfer could modulate defined targets into splenocytes.

**Summary/Conclusion**: Altogether, these findings revealed that shear stress affect selectively EVs release, miRNAs content and biodistribution to splenocytes. Future perspectives will aim to decipher the in vivo role of such EVs in the context of atherosclerosis.

## PS04 EVs and Pathogens: From Bacteria to Viruses

Chair: Ana Claudia Torrecilhas, Universidade Federal de São Paulo campus Diadema, Brazil

Chair: Chioma Okeoma, Stony Brook University, United States

### PS04.01 Exosomes derived from covid‐19 infected mesenchymal stem cells can activate antigen‐specific immune response against covid‐19 virus

zahra naseri, Department of molecular biology, Faculty of Curative Medicine, Khatam‐al‐nabieen University, Kabul, Afghanistan.

hossein Rahimi, Department of Biology and Microbiology, Medical laboratory Science Institute, Khatam‐al‐nabieen University, Kabul, Afghanistan.

**Introduction**: Due to the rapid spread of covid‐19 infection around the world, there is a fundamental need to design an effective vaccine. Mesenchymal stem cells (MSCs) are unique multipotent progenitor cells that are presently being exploited as gene therapy vectors for a variety of conditions. MSCs have also demonstrated some success in anti‐microbial prophylactic vaccines. MSCs exert their therapeutic effects via secretion of soluble paracrine factors and exosomes. Exosomes are known as naive nanovesicles that play an important role in intercellular communications. Some studies have shown the presence of some viral components in exosomes secreted by virus‐infected cells. So, we hypothesized that covid‐19 infected mesenchymal stem cells produce exosomes carrying viral components that can be used as an effective vaccine against covid‐19 infection.

**Methods**: The mouse bone marrow‐extracted MSCs became infected with the covid‐19 virus obtained from positive patients. Then, the exosomes were extracted from the infected cells by ultracentrifugation and characterized. The secreted exosomes were then injected to balb/c mice intranasally to evaluate the antisera.

**Results**: The extracted MSCs had a defined‐shaped fibroblastic morphology with moltipotent nature that were positive for for CD29, CD44, CD90, CD105 and negative for CD34 and CD45. TEM analysis verified the disc‐shaped of MSCs‐Exo and showed that the exosomes had average size between 30–150 nm. Expression of some important exosomal markers such as CD81 and CD63, and not expression of Calnexin, endoplasmic reticulum marker, were characterized using western blot. In addition, the expression of a specific SARS‐CoV‐2 spike antigen, S protein, was confirmed using specific antibody in western blotting. Interestingly, intranasal administration of mice with the exosomes isolated from covid‐19 virus infected MSCs induced neutralizing antibody titers that confirmed by ELISA and plaque reduction neutralization (PRNT) assays.

**Summary/Conclusion**: In conclusion, our results suggest that the exosomes may be a promising vaccine candidate against covid‐19 infection.

### PS04.02 Extracellular Vesicles derived from Antigen Presenting Cells pulsed with inactivated Foot and Mouth Disease VIrus express a high level of viral proteins and induce a specific antiviral response

Florencia Menay, National Research Council of Argentina (CONICET)

Federico Cocozza, Institut Curie / INSERM U932

Maria Jose Gravisaco, INTA

Analia Elisei, INTA

Javier Re, INTA

Claudia Waldner, National Research Council of Argentina (CONICET)

Pura Sampedro, Universidad de Moron

Alejandra Ferella, National Research Council of Argentina (CONICET)

Claudia Mongini, National Research Council of Argentina (CONICET)

**Introduction**: Foot and mouth disease (FMD) is a worldwide economically important infection caused by FMD‐virus (FMDV).The main strategy for the control is vaccination with FMDV chemically inactivated with binary ethylenimide (FMDVi). In FMDV infection and in vaccination, the B cell response plays a major role by providing neutralizing/protective antibody in both animal models and natural hosts. EVs secreted by antigen presenting cells (APC) participate in the activation of B and T cells through the presentation of native antigen membrane associated (to B cells) or by transferring MHC‐peptide complexes (to T cells) and even complete antigen from DCs. We aimed to evaluate the immune properties of EVs derived from APC pulsed with FMDVi in a murine model.

**Methods**: Bone marrow‐derived DCs differentiation. In Vitro APC pulse load with BEI‐FMDVI. EVs isolation by differential centrifugation ultrafiltration, and ultracentrifugation. EVs immunofluorescence staining and Flow cytometry. Size distribution by Nanoparticle Tracking Analysis. Expression of viral protein on EVs at different secretion and pulse time. Carboxyfluorescein Succinimidyl Ester (CFSE) Proliferation Assay and Flow cytometry.

**Results**: APC differentiated from bone marrow cells internalized FMDVi labelled with FITC after incubation for 60 min. EVs were isolated after 24h from APC pulse with FMDVi. By flow cytometry EVs expressed the EVs (CD9, CD81, CD63) and APC markers (MHC‐II and CD86). Remarkably, FMDV antigens were expressed on >89% EVs. We demonstrated that EVs‐FMDVi induced specific proliferation in vitro in splenocytes sensitized with FMDVi, EVs‐FMDVi induced specific B cell (16.05% ± 0.61 p < 0.001) and T cell proliferation (8.5% ± 0.81 p < 0.01) when compared to the control (9.66% ± 0.17 and 5.70% ± 0.15, respectively) detected by CFSE dilution.

**Summary/Conclusion**: We demonstrated that APC cells can internalize FMDVi and release EVs expressing APC markers and high level of viral proteins. Our results revealed that EVs‐FMDVi could present FMDV proteins in native conformation or partially processed. These peptides can be recognized by the BCR and stimulate specific B cell response against viral infection. In addition, EVs‐FMDVi activate direct or indirectly a T cell response that could collaborate in B cell activation. The knowledge derived from this work will serve to deepen the knowledge of the interrelation between the FMDV and the immune system for the rational design of vaccines.

### PS04.03 Let‐7b in extracellular vesicles secreted by human airway epithelial cells increases the ability of beta‐lactam antibiotics to reduce P. aeruginosa biofilm formation

Katja Koeppen, Department of Microbiology and Immunology, Geisel School of Medicine at Dartmouth

Amanda Nymon, Department of Microbiology and Immunology, Geisel School of Medicine at Dartmouth

Roxanna Barnaby, Department of Microbiology and Immunology, Geisel School of Medicine at Dartmouth

Laura Bashor, Department of Microbiology and Immunology, Geisel School of Medicine at Dartmouth

Zhongyou Li, Department of Microbiology and Immunology, Geisel School of Medicine at Dartmouth

Thomas H. Hampton, Department of Microbiology and Immunology, Geisel School of Medicine at Dartmouth

Amanda E. Liefeld, Department of Microbiology and Immunology, Geisel School of Medicine at Dartmouth

Fred W. Kolling, Norris Cotton Cancer Center, Geisel School of Medicine at Dartmouth

Ian S. LaCroix, Norris Cotton Cancer Center, Geisel School of Medicine at Dartmouth

Scott A. Gerber,Norris Cotton Cancer Center, Geisel School of Medicine at Dartmouth

Deborah A. Hogan, Department of Microbiology and Immunology, Geisel School of Medicine at Dartmouth

Swetha KasettyDepartment of Biological Sciences, Dartmouth College

Carey D. Nadell, Department of Biological Sciences, Dartmouth College

Bruce A. Stanton,Department of Microbiology and Immunology, Geisel School of Medicine at Dartmouth

**Introduction**: P. aeruginosa is an opportunistic pathogen that forms antibiotic‐resistant biofilms which facilitate chronic infections in immunocompromised hosts. We have previously shown that P. aeruginosa secretes outer membrane vesicles that deliver a small RNA to human airway epithelial cells where it suppresses the innate immune response. Here, we demonstrate that inter‐domain communication through small RNA‐containing membrane vesicles is bi‐directional and that miRNAs in extracellular vesicles (EV) secreted by human airway epithelial cells regulate protein expression, antibiotic sensitivity and biofilm formation by P. aeruginosa.

**Methods**: We used RNA‐seq to characterize the RNA content of EVs secreted by human airway epithelial cells and to show delivery of human miRNAs from EVs to P. aeruginosa. We evaluated the response of P. aeruginosa to human EVs and to let‐7b‐5p using biotic and abiotic biofilm formation assays. Sensitivity to the beta‐lactam antibiotic aztreonam in the presence and absence of EVs was determined using growth curve assays. Targets of let‐7b‐5p in P. aeruginosa were predicted using IntaRNA and validated in proteomics experiments.

**Results**: We found that human EVs deliver let‐7b‐5p, a 22‐nt miRNA, to P. aeruginosa, which systematically decreases the abundance of proteins essential for biofilm formation, including PpkA and ClpV1, and increases the ability of beta‐lactam antibiotics to reduce biofilm formation by targeting the beta‐lactamase AmpC. Let‐7b is bioinformatically predicted to target PpkA, ClpV1 and AmpC not only in P. aeruginosa, but also the corresponding orthologs in Burkholderia cenocepacia, another notorious opportunistic lung pathogen, suggesting that the ability of let‐7b‐5p to reduce biofilm formation and increase beta‐lactam sensitivity is not limited to P. aeruginosa.

**Summary/Conclusion**: To our knowledge, this is the first direct evidence for transfer of miRNAs in EVs secreted by eukaryotic cells to a prokaryotic organism, resulting in subsequent phenotypic alterations in the prokaryote as a result of this transfer. Since let‐7 family miRNAs are in clinical trials to reduce inflammation and, given that chronic P. aeruginosa lung infections are associated with a hyper‐inflammatory state, treatment with a combination of let‐7b‐5p and a beta‐lactam antibiotic in nanoparticles or EVs are predicted to benefit patients with antibiotic‐resistant P. aeruginosa infections.

### PS04.04 Urinary SARS‐CoV‐2 RNA is an indicator for the progression and prognosis of COVID‐19 disease

Huiming Wang, Renmin Hospital of Wuhan University

Lu Zhang, Renmin Hospital of Wuhan University

Maoqing Tian, Renmin Hospital of Wuhan University

**Introduction**: SARS‐CoV‐2 RNA can be detected in the urine of Coronavirus disease 2019 (COVID‐19) patients. However, it is unclear whether urinary SARS‐CoV‐2 RNA (URNA+) is associated the severity and clinical manifestations of hospitalized COVID‐19 patients.

**Methods**: In this study, we analyzed the demographic and clinical data of COVID‐19 patients and detected SARS‐CoV‐2 RNA in urine sediments collected from 53 COVID‐19 patients enrolled in Renmin Hospital of Wuhan University from January 31, 2020 to February 18, 2020 with qRT‐PCR analysis, and then classified those patients based on clinical conditions (severe or non‐severe syndrome) and urinary SARS‐CoV‐2 RNA (URNA‐ or URNA+).

**Results**: We found that COVID‐19 patients with severe syndrome (severe patients) showed significantly higher positive rate (11 of 23, 47.8%) of urinary SARS‐CoV‐2 RNA than non‐severe patients (4 of 30, 13.3%, p = 0.006). URNA+ patients or severe URNA+ subgroup exhibited higher prevalence of inflammation and immune discord, cardiovascular diseases, liver damage and renal disfunction, and higher risk of death than URNA‐ patients. To understand the potential mechanisms underlying the viral urine shedding, we performed renal histopathological analysis on postmortems of patients with COVID‐19 and found that severe renal vascular endothelium lesion characterized by increase of the expression of thrombomodulin and von Willebrand factor, markers to assess the endothelium dysfunction. We proposed a theoretical and mathematic model to depict the potential factors determining the urine shedding of SARS‐CoV‐2.

**Summary/Conclusion**: This study indicated that urinary SARS‐CoV‐2 RNA detected in urine specimens may be used to predict the progression and prognosis of COVID‐19 severity.

### PS04.05 Bacterial lipid content of extracellular vesicles released by macrophages infected by Mycobacterium tuberculosis

Pierre Boyer, Institut de Pharmacologie et de Biologie Structurale, Université de Toulouse, CNRS, Université Paul Sabatier

Jérôme Nigou, CNRS

Emilie Layre, Institut de Pharmacologie et de Biologie Structurale, Université de Toulouse, CNRS, Université Paul Sabatier

**Introduction**: Tuberculosis is one of the top ten causes of death worldwide. Mycobacterium tuberculosis (Mtb) is an intracellular pathogen of alveolar macrophages that has evolved strategies to adapt to and subvert host defenses. In most cases of infection, the host immune responses only controls the bacillus dissemination, leading to latent tuberculosis. Understanding the molecular bases of the complex cross‐talk between the host and the bacillus is key to design new anti‐TB strategies. The lipids, glycolipids, lipoproteins and lipoglycans that compose Mtb envelope play a major role in host‐pathogen interactions, acting as Pathogen Associated Molecular Patterns, antigens or virulence factors. If the functions of these molecules is conceived at the bacillus surface, a yet incompletely characterized repertoire of bacterial lipid traffic within infected cells and out of the infected cells within extracellular vesicles (EV) that have the potential to modulate the response of bystanders cells. A better characterization of the lipid content of EV released by infected cells will provide better insight into the role of mycobacterial lipids and EV themselves in host‐pathogen interactions.

**Methods**: The populations of EV released by infected macrophages were purified by combining centrifugal centrifugation and density gradient. Purified vesicles were characterized by nanosight, microscopy, FACS and western blot. Their content in mycobacterial lipids and lipoglycans was characterized by western blot and using a high sensitivity and last generation mass spectrometry‐based approach.

**Results**: EV populations were purified from cultures of infected macrophages. Using western blot analyses and a targeted mass spectrometry approach, we performed a comprehensive analysis of EV content in mycobacterial lipidic molecules. We highlighted the presence of additional mycobacterial lipid families. Using different bacterial species, we have also assessed whether the lipid content of EV released by infected cells differs with the bacillus virulence degree, which would subsequently result in different immunomodulatory properties.

**Summary/Conclusion**: Several families of immunomodulatory mycobacterial lipids, lipoproteins and lipoglycans, traffic within vesicles released by macrophages, by which they likely modulate immune responses beyond the infected cells. Further investigations are required to assess the diversity of EV populations, their fine composition and immunomodulatory properties.

### PS04.06 Plasma‐derived EV analysis indicates the persistency of fibrogenic signals in HCV DAA‐treated patients that reached sustained virological response

Cecilia Battistelli, Sapienza University of Rome

Claudia Montaldo, Spallanzani National Institute for Infectious Diseases

Michela Terri, Sapienza University of Rome

Veronica Riccioni, Sapienza University of Rome

Veronica Bordoni, Spallanzani National Institute for Infectious Diseases

Gianpiero D'Offizi, Spallanzani National Institute for Infectious Diseases

Maria Giulia Prado, Sapienza University of Rome

Tiziana Vescovo, Spallanzani National Institute for Infectious Diseases

Eleonora Tartaglia, Spallanzani National Institute for Infectious Diseases

Raffaele Strippoli,Sapienza University of Rome

Chiara Agrati, Spallanzani National Institute for Infectious Diseases

Marco TripodiSapienza University of Rome

**Introduction**: HCV SVR achievable now by means of DAA therapy identifies a new class of patients requiring medical surveillance to be designed in relation to the liver disease stage advancement. To this end, identification of both disease biomarkers and therapeutic target appears necessary.

**Methods**: Extracellular Vesicles (EVs) purified from plasma of 5 healthy donors (HD), and 5 HCV infected patients before (T0) and after (T6) DAA treatment have been utilized for functional and miRNA cargo analysis. EVs purified from plasma of 13 HD and 13 T0 and T6 patients have been employed for proteomic and western blot analysis. Functional analysis in LX2 cells measured fibrotic markers (mRNAs and proteins) in response to EVs. Structural analysis was performed by qPCR, mass spectrometry (orbitrap) and Western blot.

**Results**: On the basis of observations indicating functional differences of plasma‐derived EVs from HD, T0 and T6, we endeavour EV structural analysis. We found consistent differences in terms of both miRNAs and proteins cargos; (i) antifibrogenic miR204‐5p, miR93‐5p and miR181a‐5p were found statistically underrepresented in T0 EVs with respect to HD and miR204‐5p and miR143‐3p were found statistically different between HD and T6 (p‐value < 0.05) (ii) proteomic analysis highlighted, in both T0 and T6, the modulation of several proteins with respect to HD; among them the fibrogenic DIAPH1 was found upregulated by western blot (4.4 Log2 fold change).

**Summary/Conclusion**: Taken together, these results highlight structural EVs modifications, conceivably causal for long‐term liver disease progression in HCV patients that persist despite the DAA‐mediated HCV SVR.

### PS04.07 Syntenin facilitates dissemination of ZIKV through regulating exosomes release and uptake

Rui Zhang, Nan Jing university, China

Min Cheng, NanJing University

Zhiwei Wu, NanJing University

**Introduction**: Exosomes was considered as small membrane‐encapsulated vesicles that was loaded with proteins, lipids and RNAs that compose ‘a signature’ of the cell of origin and potentially can reprogram‐alter recipient cells. One of the main exosomal functions is to mediate intercellular communication during viral pathogenesis and immune responses. Zika virus (ZIKV), a re‐emerging mosquito‐borne flavivirus, is an enveloped, positive single‐stranded RNA virus, had reported continuing vector‐borne transmission of ZIKV. ZIKV infection is the association of some viral strains with neurological diseases. During viral infection, some exosomes regulatory proteins regulate the release and uptake of viral exosomes, In particular, syntenin plays a role in exosome biogenesis. Given the pivotal role played by syntenin in exosome biogenesis, here we studied the role of syntenin in the spread of exosome‐zika.

**Methods**: A549 cells were infected with Zika, and the secretion of exosomes was significantly increased. The role of Syntenin in the process of viral exosome generation was explored by CRISPA‐CAS9 technique.

**Results**: We found that Zika virus (ZIKV) infection increased the release of exosomes in A549 cells, and exosomes derived from zika virus infected cells have the ability to re‐establish infection in vivo and in vitro (in a manner independent of traditional viral receptor‐dependent pathways). In addition, we also found that ZIKV infection up‐regulates syntenin (which was supported the formation of intraluminal vesicles that compose the source of a major class of exosomes, supported the recycling of these same components to the cell surface.), and syntenin controlled re‐infection of exosome‐zika by affecting exosome release and uptake.

**Summary/Conclusion**: Our study significantly extended RNA virus (Zika) infection and regulated exosome secretion, and the related molecular protein (syntenin) regulated the biological significance of exosome‐zika.

### PS04.08 Outer membrane vesicles from Hypervirulent Klebsiella pneumoniae mediate virulence factor transfer

Yuneng Hua, Southern Medical University

**Introduction**: Outer membrane vesicles (OMVs) are potent virulence factors, naturally secreted by gram‐negative bacteria, acting as mediators of inter‐ and intra‐species communication. Since Hypervirulent Klebsiella pneumoniae(HvKp) has emerged as an important nosocomial community‐acquired pathogen, because of more virulent than classical K. pneumoniae (CKp), it is crucial to investigate its pathogenetic mechanism as it relates to OMVs.

**Methods**: NTA;WB;TEM;BCA;PCR;flow cytometry;proteomics;CLSM

**Results**: In this work, we indicate that HvKp OMVs may contain several virulence factors that contribute to virulence and disease pathology. HvKp OMVs are mediating virulence factors transfer and allowing increase in the virulence level of CKP or CRKP.

**Summary/Conclusion**: In this work, we indicate that HvKp OMVs may contain several virulence factors that contribute to virulence and disease pathology. HvKp OMVs are mediating virulence factors transfer and allowing increase in the virulence level of CKP or CRKP.

### PS04.09 Characterisation of membrane vesicles in bacterial sepsis pathogens

Christian Grätz, Technische Universität München

A. Ronja D. Binder, Technische Universität München

Anja Lindemann, University Hospital, Ludwig‐Maximilians‐Universität München

Genia Lücking, Technische Universität München

Michael W. Pfaffl, PhD, Chair of Animal Physiology & Immunology

Gustav Schelling, University Hospital, Ludwig‐Maximilians‐Universität München

Marlene Reithmair, University Hospital, Ludwig‐Maximilians‐Universität München

**Introduction**: Sepsis is defined as a life‐threatening organ dysfunction due to a dysregulated host response to infection with bacteria as the most common infectious agent. Gram‐positive (G+) and Gram‐negative (G‐) bacteria shed membrane vesicles (MVs) which carry various types of RNA. By merging with eukaryotic cells, these may induce a systemic inflammatory response. After antibiotic treatment, it is often impossible to identify life bacteria by cultivation. MV‐associated RNA shed by these bacteria may be used as a more reliable indicator of bacterial infection.

**Methods**: Here, we characterised MVs and their RNA cargo from six bacteria, five of them commonly involved in sepsis pathogenesis: S. saprophyticus (G+), S. aureus (G+), C. jeikeium (G+), H. influenzae (G‐) and H. parainfluenzae (G‐). The pathogenic E. coli strain O1:K1:H7 served as a control although it is not necessarily associated with sepsis. MVs were isolated from the sterile‐filtered bacterial culture supernatant using ultracentrifugation and for further purification a density gradient step was added. Nanoparticle tracking analysis (NTA) was used to measure particle number and size distribution of the isolated MVs, which were then visualised using transmission electron microscopy (TEM). Western blotting identified various MV markers. RNA from selected samples was analysed by next‐generation sequencing (NGS).

**Results**: All six bacteria shed membrane‐enclosed vesicles, with a tendency to a higher MV number in G‐ bacteria. In the supernatant of E. coli and C. jeikeium, a high number of flagella was found. MV density varied between the six bacteria, ranging from 1.12 to 1.21 g/ml. The MV marker groEL was detected in the purified MVs of all G‐ bacteria. Preliminary NGS results for E. coli and H. influenzae showed bacteria‐specific rRNA transcripts at the top 15 genes with the most generated reads, followed by protein coding genes.

**Summary/Conclusion**: All pathogens produced high numbers of RNA‐containing MVs. The uptake of those MVs and their RNA cargo into human blood cells will be assessed in a follow‐up study. It remains to be shown that bacterial MVs or their RNA can be detected in blood samples from sepsis patients, but MV‐associated RNA remains a promising sepsis biomarker.

### PS04.10 Differential shedding of EVs by distinct Trypanosoma cruzi strains during metacyclogenesis

Ana Claudia Claudia. Torrecilhas, Universidade Federal de São Paulo campus Diadema

Paula Meneghetti Meneghetti, UNIFESP

Rafael Pedro Madeira, UNIFESP

João Paulo Ferreira, UNIFESP

Nobuko Yoshida, UNIFESP

**Introduction**: The protozoan parasite Trypanosoma cruzi spontaneously releases extracellular vesicles (EVs). In a previous research, we have shown that T. cruzi EVs are shed from epimastigote forms during interaction with triatomine bugs Rhodnius prolixus and Triatoma infestans. The EVs delayed parasite migration to rectum only in the gut of R. prolixus. The aim of our research is the characterization of EVs released during metacyclogenesis of different T. cruzi strains (Y, CL and G)

**Methods**: Methods The epimastigotes forms from T. cruzi strains (Y, CL and G) were cultivated during 30 days to follow the parasite growth, metacyclogenesis, and release of EVs. Nanoparticle tracking analysis (NTA) was performed to check the size and concentration of the EVs isolated from different strains during metacyclogenesis.

**Results**: Results The size of EVs isolated from CL strains was large than Y and G strains. Y strain parasites released more EVs (1 × 109 particles/ mL) than strains G (6 × 108 particles / mL) and CL (5 × 108 particles / mL).

**Summary/Conclusion**: Conclusion During metacyclogenesis, T. cruzi strains Y, CL and G released EVs of different sizes and their numbers varied between strains. This may be associated with the parasite development and differentiation.

### PS04.11 Circulatory EVs as potential biomarkers of HIV‐drug abuse interactions and neurological dysfunction in HIV‐Infected subjects and alcohol/tobacco Users

Sunitha Kodidela, UTHSC

Namita Sinha, UTHSC

Asit Kumar, Ph.D., University of Tennessee Health Science Center

Prashant Kumar, UTHSC

Santosh Kumar, UTHSC

**Introduction**: Abuse of alcohol and tobacco can exacerbate HIV pathogenesis by transferring materials through extracellular vesicles (EVs). EVs present a stable and accessible source of biological information from one cell to various types of cells, including brain cells. Therefore, we aimed to study the plasma EVs proteins, which are altered in both HIV and drug abusers to identify a physiological marker to indicate the immune status and neuronal damage of HIV‐positive drug abusers

**Methods**: EVs were isolated from plasma samples of the following subjects by double isolation method to improve their purity: a) Healthy b) HIV c) Alcohol drinkers d) Smokers e) HIV+drinkers f) HIV+smokers. Quantitative proteomic profiling of EVs was performed by mass spectrometry and potential EV proteins associated (G‐FAP and L1‐CAM) with glial and neuronal dysfunction were quantified by western blot.

**Results**: The EVs were characterized according to the MISEV guidelines. Comparison of proteins among all the study groups revealed that hemopexin was significantly altered in HIV+drinkers compared to drinkers and HIV subjects. Further, our study is the first to show properdin expression in plasma EVs, which was decreased in HIV+smokers and HIV+drinkers compared to HIV patients. The levels of astroglial marker (GFAP) were elevated in plasma EVs from HIV subjects compared to healthy subjects. Further, the levels of GFAP and L1‐CAM were increased in drinkers and smokers without HIV infection compared to healthy subjects

**Summary/Conclusion**: The present findings suggest that plasma EVs serve as potential markers for complications associated with substance abuse in HIV‐subjects. In particular, hemopexin, and properdin show potential as markers for HIV‐drug abuse interactions. The present study has also established that the astrocytic and neuronal‐specific markers (GFAP and L1CAM), which have the potential to play a role in neurological dysfunctions, can be packaged in EVs and circulated in plasma.

### PS04.12 Extracellular vesicles from ethanol‐treated and HIV‐infected macrophages induce inflammasome activation in hepatocytes

Edward Makarov, Department of Pharmacology and Experimental Neuroscience, University of Nebraska Medical Center, Omaha, NE, USA 68105

Moses New‐Aaron, Research Department, Veteran Affairs Medical Center, Department of Environmental, Agriculture and Occupational Health, University of Nebraska Medical Center, Omaha, NE, USA 68105

Murali Ganesan, Research Department, Veteran Affairs Medical Center, Internal Medicine Department, University of Nebraska Medical Center, Omaha, NE, USA 68105

Larisa Y. Poluektova, Department of Pharmacology and Experimental Neuroscience, University of Nebraska Medical Center, Omaha, NE, USA 68105

Natalia A. Osna, Research Department, Veteran Affairs Medical Center, Internal Medicine Department, University of Nebraska Medical Center, Omaha, NE, USA 68105

Raghubendra S. Dagur, PhD, University of Nebraska Medical Center

**Introduction**: Hepatic inflammation is a common trigger of hepatic steatosis and progresses rapidly to cirrhosis, and finally, hepatocellular carcinoma in HIV‐patients who have a history of alcohol abuse. During the disease conditions, macrophages are quickly recruited to the liver and produce many extracellular vesicles (EVs). How these EVs from alcohol and HIV‐infected macrophages regulate hepatocyte survival is not clear and is our study's focus.

**Methods**: Monocyte‐derived macrophages (MDM) were exposed to 50 mM ethanol (EtOH) before infecting the cells with the HIV‐1ADA strain at a multiplicity of infection of 0.1. HIV‐infected cells were further exposed to 25mM EtOH, and the conditioned medium was collected from 4 groups of cells: untreated, HIV‐, EtOH‐ and EtOH+HIV. Quantification of EVs number and size was evaluated with Nanosight and characterized for EVs markers following the Minimal Information for Studies of Extracellular Vesicles guidelines, 2018. To assess EV‐mediated liver inflammation, we exposed human hepatocyte Huh7.5CYP2E1 [hepatoma cells stably transfected with CYP2E1 designated as RLW cells] to MDM‐EVs and then measured inflammasome activation based on NLRP3, caspase 1, and IL‐1β mRNAs with qPCR.

**Results**: Alcohol treatment stimulated the EVs secretion from HIV‐infected macrophages. Size distribution assessed by Nanosight revealed more than 90% of particles distributed in the range of 50 to 200 nm. Western blotting of MDM‐EVs demonstrated positivity for small EVs enriched proteins Alix, TSG 101, and CD9 and negative for endoplasmic reticulum protein calnexin and HIV protein. The uptake of MDM‐EVs by hepatocytes was apparent, as demonstrated by immunofluorescence. Transfer of MDM‐EVs from the EtOH+HIV group, but not from other groups, significantly increases the activation of NLRP3 inflammasome in hepatocytes. The activation of NLRP3 was accompanied by an increase in the expression of inflammatory caspase 1 and IL‐1β.

**Summary/Conclusion**: We conclude that alcohol treatment stimulates EVs secretion from HIV‐infected macrophages. The uptake of EtOH‐and HIV‐ modified MDM‐EVs leads to activation of canonical NLRP3 inflammasome signaling in hepatocytes to promote liver inflammation and may trigger fibrosis development.

### PS04.13 Pseudomonas aeruginosa extracellular vesicles from cystic fibrosis sputum induce inflammation in human bronchial epithelial cells

Andrea L. Hahn, MD, Children's National Hospital

Aszia Burrell, BS, Center of Genetic Medicine, Children's National Research Institute

Kylie I. Krohmaly, BS, Institute of Biomedical Sciences, George Washington University

Kayla Authelet, BS, Center of Genetic Medicine, Children's National Research Institute

Claire Hoptay, PhD, Center of Genetic Medicine, Children's National Research Institute

Robert J. Freishtat, MD, MPH, Center of Genetic Medicine, Children's National Research Institute

**Introduction**: Pseudomonas aeruginosa (Pa) is an important chronic pathogen associated with intermittent pulmonary exacerbations (PEx) and lung inflammation in persons with cystic fibrosis (CF), likely mediated in part by extracellular vesicles (EVs). We hypothesized that Pa EVs obtained from the sputum of persons with CF would induce inflammation in CF human bronchial epithelial (HBE) cells.

**Methods**: Following IRB approval and informed consent, sputum samples were collected from three persons with CF, homogenized, and centrifuged at 12000g x 10 minutes. EVs were isolated from the supernatants using precipitation and size exclusion chromatography. Pa antibodies were attached to magnetic beads to isolate Pa‐specific EVs. Life extended CF HBE cells (F508del/F508del) were grown to 80% confluency, and equivalent volumes of Pa EVs or PBS (250 μL) were added and incubated for 22 hours before cell harvesting. Cells were immediately lysed with TRIzol and RNA extracted with a DirectZol MiniPrep kit. RNAseq was performed using a Mid‐Output, 75 cycle kit on a NextSeq 500. A Galaxy workflow incorporating HISAT2, Stringtie, Gffcompare, featureCounts, and DESeq2 was used to determine differential gene expression. Ingenuity pathway analysis (IPA) was used to identify differences in canonical pathways.

**Results**: RNA quality assessment showed RIN values near 10, with an average of 19 million reads per sample (range 14–25 million). Differential expression of 394 transcripts were identified using an unadjusted p value of < 0.05. When filtering for lung tissue, 8 canonical pathways with a Z‐score of ‐2 were upregulated in HBE cells exposed to Pa EVs compared to PBS exposure. This included several signaling pathways associated with inflammation and immunity: G Beta Gamma (Z‐score +2, genes GNB1L, GNG11, PRKAG2, PRKCD, RRAS), P2Y Purigenic Receptor (Z‐score +2, genes GNB1L, GNG11, PRKAG2, PRKCD, RRAS), IL‐8 (Z‐score +2, genes GNB1L, GNG11, NCF2, PRKCD, RRAS), and HMGB1 (Z‐score +2, genes IL1A, PLAT, RRAS, TGFB2).

**Summary/Conclusion**: CF HBE cells exposed to Pa EVs isolated from the sputum of persons with CF demonstrated upregulation of inflammatory and immunity pathways compared to PBS exposure. Future studies comparing the effect of Pa EVs isolated during times of wellness and PEx may provide insight into how Pa EVs impact variations in inflammation with chronic infection.

### PS04.14 miRNAs in extracellular vesicles, novel therapeutic agents for COVID‐19

Jisook Moon, College of Life Science, Department of Biotechnology, CHA University

Jae Hyun Park, CHA University

Yuri Choi, CHA University

**Introduction**: Severe acute respiratory syndrome coronavirus 2 (SARS‐CoV‐2) causes causes severe respiratory failure and there is no treatment yet. Micro‐RNAs (miRNAs) are potential novel anti‐viral agents because of their ability to degrade viral RNAs. Extracellular vesicles (EVs) is a small vesicle that secreted from cell and can transfer miRNAs to recipient cells and regulate conditions within them.

**Methods**: Mesenchymal stem cell derived EVs (MSC‐EVs) contain anti‐viral miRNAs that play important roles in the virus‐infected host cells. Here, we examined their potential impact on viral and immune responses. Here, we identified candidate therapeutic miRNAs with important roles in the biological functions of virusinfected host cells, and characterized the antiviral effects of miRNAs derived from placenta‐derived MSC‐EVs

(pMSC‐EVs) or placenta EVs, which exhibit potent regenerative and anti‐inflammatory effects.

**Results**: MSC‐EVs contained 18 miRNAs predicted to interact directly with the 3’ UTR of SARS‐CoV‐2. In addition, five major miRNAs in MSC‐EVs suppressed viral 3’ UTR sequence in a luciferase reporter assay and these EVs suppressed SARS‐CoV‐2 infection in Vero E6 cells.

Moreover, MSC‐EVs showed anti‐inflammatory effect which may prevent lethal cytokine storms caused by viral infection and we confirmed that MSC‐EVs suppressed inflammatory responses by several cell types.

**Summary/Conclusion**: miRNAs in MSC‐EVs have several advantages as therapeutic agents against SARS‐CoV‐2: 1) they can inhibit viral 3’ UTR and suppress viral replication; 2) because the 3’ UTR is highly conserved and rarely mutates, MSC‐EV miRNAs could be used against new variants of SARS‐CoV‐2; and 3) unique cargoes carried by MSC‐EVs can have immunomodulatory effect.

### PS04.15 Rapid Isolation Protocol for Circulating Extracellular Vesicles in Chronic Chagas Disease Patients

Ana Claudia Claudia. Torrecilhas, Universidade Federal de São Paulo campus Diadema

Rafael Pedro Madeira, UNIFESP

Lucas Alexandre Barros, UNIFESP

**Introduction**: Extracellular vesicles are lipid bilayer envelopes that encase several types of molecules in their interior in addition to those in their membranes. Their contents mostly reflect their cell of origin and possible targets at other places in the organism and can also be modified in pathological conditions to interfere with intercellular communication, promoting disease establishment and development. These characteristics, in addition to their presence in virtually all body fluids, make them ideal for biomarker research

**Methods**: In Chagas disease where no biomarker exists to date to infer prognosis in indeterminate stage chronic patients, our work proposed establishing a protocol for circulating extracellular vesicle isolation in this population. For this, we isolated extracellular vesicles from blood collected with different anticoagulants by centrifugation, quantified their protein content and, through nanoparticle tracking analysis, characterized their size and concentration

**Results**: We observed that while anticoagulants did not interfere with the parameters analyzed, the occurrence of Chagas disease had an impact on the mean size and size dispersion. Altogether, our protocol is adequate for the isolation of circulating extracellular vesicles and an important basis for further studies on biomarker research.

**Summary/Conclusion**: Considering that monitoring of chronic Chagas disease cardiac burden and prognosis can only be done after the onset of symptoms and that just one study has been performed to date to assess circulating EVs in patients with chronic Chagas disease, but in this case concerning one patient with reactivation after a heart transplant, our study proposed to develop a rapid and reliable isolation and characterization method for chronic Chagas patients’ circulating EVs

## PS05 Advances in EV characterization

Chair: Michael Paulaitis, Johns Hopkin University School of Medicine, United States

Chair: Sabrina La Salvia, Icahn School of Medicine at Mount Sinai, United States

### PS05.01 Structural and mechanical characterization of the extracellular vesicles from two protozoan microorganisms using atomic force microscopy and force spectroscopy

Lissette Retana Moreira, Departamento de Parasitología, Facultad de Microbiología, Universidad de Costa Rica

Fátima Linares, Centro de Instrumentación Científica, Universidad de Granada, Spain

Antonio Osuna, 1. Instituto de Biotecnología. Universidad de Granada, Spain

Elizabeth Abrahams Sandí, Departamento de Parasitología, Facultad de Microbiología, Universidad de Costa Rica

**Introduction**: The use of atomic force microscopy (AFM) in the analysis of biological samples has been growing. AFM is a versatile technology employed to study samples at nanoscale. In AFM, a cantilever with a very sharp tip is employed to scan over the sample. Both attractive and repulsive forces between the surface and the tip make the cantilever deflect and these deflections are detected using a laser beam. In this way, changes in the direction of the reflected beam are tracked with a position‐sensitive photodiode. In biology, most of the studies using AFM have focused in imaging and characterizing the structure of DNA and proteins, the ultrastructure of organelles and the dynamics of the cell membranes, among others (Schafer et al., 2002; Heinisch et al., 2012). Regarding the extracellular vesicles (EVs), AFM has been employed during the last decade and most of the research has focused in performing structural and mechanical characterizations of exosomes isolated from different sources such as saliva (Sharma et al., 2010) and malignant (metastatic and non‐metastatic) cells (Xiao et al., 2013; Whitehead et al., 2015), and to compare how different isolation methods affect the surface structure and size of exosomes (Woo et al., 2016). In the specific area of parasitology, only few groups have employed this technology.

**Methods**: In this work, we employed AFM for imaging the EVs (mostly exosomes) secreted by epimastigotes and trypomastigotes of the protozoan parasite Trypanosoma cruzi, as well as from trophozoites of the free living amoeba Acanthamoeba sp. incubated at different temperatures. Besides topographic images, we employed force spectroscopy to determine the mechanical properties (stiffness, adhesion, Young modulus) of the EVs secreted from each organism and to compare the results between the stages or the incubation conditions. The analyses were performed using a Park Systems NX‐20 instrument (CIC, Universidad de Granada).

**Results**: The results obtained reflect significant differences in the nanomechanical properties of the EVs of trypomastigotes and epimastigotes of T. cruzi, which could be related to the protein cargo assessed by proteomics. In the case of Acanthamoeba sp., drastic differences in adhesion were observed in EVs isolated at 28°C and 37°C. These differences could have implications in the survival and damaging potential of these protozoan microorganisms in different biological environments.

**Summary/Conclusion**: Atomic force microscopy is a technology that could be employed to assess possible differences between extracellular vesicles secreted by different stages or different inubation conditions of protozoan microorganisms.

## PS05.02 Quantitative Analysis of the Size and Concentration of Exosomes Using Nanoparticle Tracking Analysis: Internal and External Calibrations

Eunjin Choi, Student of Hanyang Univ.

Sehee Park, student of Hanyang Univ.

Jaewoo Song, professor of Yonsei Univ.

Tae Hyun Yoon, professor of Hanyang Univ.

**Introduction**: Cell‐derived extracellular vesicles(EVs) and exosomes have been spotlighted recently both in fundamental research and clinical applications, since they are recognized as important mediators of intercellular signaling pathways and may provide breakthroughs in diagnosis and treatments of various diseases. However, for further applications in medical and pharmaceutical industry, it is essential to have precise, accurate, and robust quantification methods for their size, concentration, and other characteristics.

**Methods**: Nanoparticle tracking analysis(NTA) is one of the widely used characterization methods of EVs and exosomes. However, it is also known to have several drawbacks such as size‐dependant sensitivity and poorer detection limit for the biological materials with lower refractive index. For example, when measuring polydisperse particles, larger particles with high scattering signals are easily detected and tracked, while smaller particles with weak scattering signals are often ignored, resulting in biased measurements of the size distributions and number concentrations of EVs and exosomes. In this study, we have adapted NTA technique to quantitatively measure size distributions and number concentrations of platelet‐derived exosome, both in scattering and fluorescence modes. To overcome current limitations of NTA technique and minimize potential bias caused by the size‐dependant sensitivity issue, we have conducted internal and external calibrations using reference materials, such as polystyrene beads with sizes of 50, 100, and 200nm as well as the verity shells with sizes of 189nm and 374nm.

**Results**: The calibration results showed that there are size‐ and measurement mode‐ dependent deviations in the number concentrations. Depending on the size and type of the reference materials as well as the measurement mode, the number concentrations from the NTA measurements displayed a significant differences from the actual number concentrations of reference materials.

**Summary/Conclusion**: Although further validation study should be performed, the results from our study, the internal and external calibrations of the NTA measurements using various reference materials with different sizes and material types, will contribute for the establishment of more accurate and reliable characterization protocols of EVs and exosomes.

### PS05.03 Charge optimization in electrokinetic sensor for highly sensitive surface protein profiling of cancer cell derived extracellular vesicles

Siddharth S. Sahu, Department of Electrical Engineering, The Angstrom Laboratory, Uppsala University, Uppsala, Sweden

Petra Hååg, Department of Oncology/Pathology, Karolinska Institutet, Stockholm, Sweden

Amelie E. Karlström, Department of Protein Science, KTH Royal Institute of Technology, Stockholm, Sweden

Kristina Viktorsson, Department of Protein Science, KTH Royal Institute of Technology, Stockholm, Sweden

Rolf Lewensohn, Department of Oncology/Pathology, Karolinska Institutet, Stockholm, Sweden

Jan Linnros, Department of Applied Physics, KTH Royal Institute of Technology, Stockholm, Sweden

Apurba Dev, Department of Electrical Engineering, Uppsala University, Uppsala, Sweden

**Introduction**: Extracellular vesicles, including small extra cellular vesicles (sEVs)/exosomes play a vital role in inter and intracellular communication of tumors and have attracted a lot of interest as sources of biomarkers for cancer diagnostics and treatment monitoring. We have already shown that surface protein profiling of tumor cell derived sEVs is possible using streaming current method. However, a better understanding of the role of surface charge in electrokinetic biosensing through both theoretical and experimental means has opened up the scope of enhancing the signal from sEVs detection. Alternative surface functionalization strategies can be explored for this purpose.

**Methods**: EVs were isolated from conditioned cell culture media of the non‐small cell lung cancer (NSCLC) cells H1975 by size exclusion chromatography. They were profiled for size/amount by nanoparticle tracking analysis. Evaluation of EV markers and purity was performed by western blotting. sEVs from both untreated and Epidermal Growth Factor Receptor (EGFR) tyrosine kinase inhibitors (EGFR‐TKI) treated cells were used. The charge contrast between the negatively charged sEVs and the sensor surface was increased by coating the sensor surface with positively charged biotinylated copolymers of poly‐L‐lysine and polyethylene glycol. Further charge tuning was achieved by the choice of linkers (avidin/streptavidin), followed by immobilization of biotinylated antibodies/affinity capture probes against CD9, CD63, and EGFR. This method was then used for assessing change in the expression levels of the sEV surface markers prior and post EGFR‐TKI treatments.

**Results**: When streptavidin and avidin were used as the linkers, the surface zeta potential was ‐10.6 and ‐2.5 mV respectively. These are significantly less negative than the previously reported value of ‐32.5 mV, obtained by a silanisation based functionalization approach with glutaraldehyde linker. With the new method, the net signals against CD9 detection were 7.7 and 23.1 mV for streptavidin and avidin linkers respectively, marking 3‐fold and 10‐fold improvement against the previously reported value for the sEV concentration 3.5e9 particles/mL. This is clearly a result of the surface charge getting less negative before capturing negatively charged sEVs, and is consistent with theoretical predictions. The sEVs surface expression studies of CD9, CD63 and EGFR in samples from NSCLC cells prior and post EGFR‐TKI treatments ongoing.

**Summary/Conclusion**: Our results demonstrate the possibility to enhance the signal by tuning the surface charge, thus expanding the scope of surface protein profiling of cancer cell derived sEVs by electrokinetic sensing.

### PS05.04 Phenol‐free extraction workflow for analysis of urinary exosomal RNA and detection of TMPRSS2:ERG in prostate cancer

Anne Bickel, Exosome Diagnostics GmbH

Anja Reichert, Exosome Diagnostics GmbH

Nike Bahlmann, QIAGEN GmbH

Georg Stoll, Exosome Diagnostics GmbH

Lisa Meyer, MSc, Exosome Diagnostics GmbH

Kurt Franzen, Exosome Diagnostics, Inc.

JAMES HURLEY, EXOSOME DIAGNOSTICS

Markus Sprenger‐Haussels, QIAGEN GmbH

Martin Schlumpberger, QIAGEN GmbH

Johan Skog, Ph.D.,Exosome Diagnostics, Inc.

Mikkel Noerholm, Exosome Diagnostics GmbH

Daniel EnderleExosome Diagnostics GmbH

**Introduction**: Exosomes and other extracellular vesicles (EVs) are a valuable source of RNAs from biofluids. EV messenger RNAs (mRNA) include known biomarkers that are used in tissue biopsies for oncological diseases, e.g. TMPRSS2:ERG fusion transcripts found in prostate cancer. To facilitate clinical research, reliable isolation methods for EV‐derived RNA (exoRNA) are needed that do not contain harmful chemicals like phenol or chloroform. This is especially challenging for urine samples due to the significant variability in the major constituents of this biofluid, many of which are potent inhibitors of RT‐qPCR.

**Methods**: We developed an improved membrane affinity‐based workflow for simple bind‐wash‐elute isolation that does not require phenol/chloroform to extract exoRNA from up to 20 mL Urine. We used this workflow to extract exoRNA from urine samples from healthy donors and confirmed prostate cancer subjects scheduled for radical prostatectomy. RT‐qPCR was used to quantify housekeeping mRNAs and prostate specific biomarkers TMPRSS2:ERG fusion (T2:E) and KLK3 (Prostate Specific Antigen mRNA, PSA).

**Results**: Urinary mRNAs are detected from 2–20 mL of input volume with RT‐qPCR signals scaling with input volume and depletion of mRNA signals in the remaining flow‐through. Repeated extractions on the same urine demonstrated robust performance of isolation and inhibitor removal from the sample. In addition to mRNAs, exosomal miRNAs can be analyzed in the isolates. TMPRSS2:ERG and KLK3 biomarkers were detected in urine from a cohort of subjects with high‐grade prostate cancer, consistent with observations in tissue previously reported in literature.

**Summary/Conclusion**: We present a new workflow for extraction of urinary RNAs that is suitable for application in routine laboratory use.

### PS05.05 NanoBioAnalytical platform for the characterization of Canine Mesenchymal Stromal Cells extracellular vesicles

geetika Raizada, FEMTO‐ST Institute

Rodolphe Rakic, Vetbiobank

Céline Elie‐Caille, Institute FEMTO‐ST

Nathalie Saulnier, Vetbiobank

Wilfrid Boireau, Institute FEMTO‐ST

**Introduction**: Mesenchymal stromal cells (MSCs) are of clinical interest because of their validated safety profile and their tremendous biological properties. These cells also secrete a large set of paracrine factors that support the healing process mainly through their interaction with the host immune cells. Canine MSCs have been used successfully for veterinary application and represent a valuable preclinical model for naturally‐occuring diseases. The aim of this study is to characterize, through an innovative analytical platform, the EVs populations produced by canine neonatal MSCs, possibly their subset and evaluate their properties.

Technically challenging task of measuring the concentration, size and characterization of a diverse population of EVs has led researchers to explore several technologies as standalone or in combination. In this study, we have utilized the Tunable Resistive Pulse Sensing (TRPS) to obtain EVs concentration and size distribution and the NanoBioAnalytical (NBA) platform especially developed in our group (Obeid et al. 2017 and 2019) to characterize EVs from different samples of canine‐MSC conditioned media (CM).

**Methods**: NBA platform utilizes Surface Plasmon Resonance imaging (SPRi) for real‐time detection and characterization of EVs according to their phenotype; which were then further deeply characterized by Atomic Force Microscopy (AFM) to precise size distribution and distinguish captured EVs from other biological materials (like protein aggregates).

**Results**: Captured EVs from different MSC‐CM were found to be CD44+, CD81+, CD9+ and CD90+ while presenting different phenotype profiles. Moreover, based on AFM investigation of immunocaptured EVs, we could refine SPRi signals obtained, as example the anti‐CD81 immunospot was mainly due to a small‐EVs population whereas the presence of EVs, cell debris and filament like structures are mainly characterized on anti‐CD44 spots. Current progress aims to compare results from the multiplexed SPR/AFM study and in vitro functionality of various EV subsets to distinguish the most promising culture conditions for EVs bioproduction and their future use for therapeutic treatments.

**Summary/Conclusion**: For the first time, our NBA platform serves to establish, optimize and qualify the production line of EVs for clinical purposes. We would address in the near future genomic and proteomic analysis directly on the immunocaptured EVs that will help us in establishing a comprehensive correlation between the functional activity and the components of the conditioned media.

### PS05.06 Comparison of MSC‐EV populations from different MSC sources using MSCSPlex, ExoView and Nanoimager instruments

Renata Skovronova, University of Turin

Cristina Grange, Department of Medical Sciences, University of Turin

Benedetta Bussolati, University of Turin, Department of Molecular Biotechnology and Health Sciences, Italy

**Introduction**: Mesenchymal stromal cells and their extracellular vesicles (MSCs‐EVs) have been in the centre of regenerative research. The current field of EV‐stem cell therapy focuses on the small‐EVs fraction of EVs, as they have been implicated to ameliorate tissue injury. This project aims to characterize trough different techniques and compare the different fraction of MSC‐EVs (small, large EVs and apoptotic bodies) from different MSC sources (bone marrow, adipose tissue, and umbilical cord).

**Methods**: MSCs are cultured until 80% confluency at 37°C in Alpha MEM medium with 10% of serum. To collect apoptotic EVs, apoptosis is induced using 500ng/mL Anti‐Fas antibody. Medium from overnight starved cells is collected then centrifuged at 1500g for 15mins to pellet apoptotic bodies. The supernatant is centrifuged further at 10,000g for 1h, to pellet large‐size EVs, another ultracentrifugation step of 100,000g for 1h is applied to collect small‐size EVs. EVs are pooled and kept at ‐80°C in medium with 0.1%DMSO. Nanosight is performed to analyse the concentration and size of EVs. dSTORM analysis using super‐resolution microscopy (NanoImager) is used to detect single vesicles and their markers. ExoView multiplex analysis is used to detect the size and marker expression. MACSPlex, a semi‐quantitative kit aimed to assess EV surface markers is used to test each fraction of the MSC‐EVs.

**Results**: The techniques used showed the EVs to be in their expected size ranges. The presence of tetraspanin (CD63, CD81, CD9) and mesenchymal (CD105, CD49e, CD44, CD29, CD146) markers were confirmed. In comparison with all fractions, umbilical cord‐derived small‐EVs showed a higher expression of CD63 and CD105. All large size EVs showed a higher expression of CD40 whereas the small size EVs of Annexin A1. Super resolution microscopy and multiplex analysis further allowed us to determine the EV particle count and size. Interestingly, overlapping results were obtained using different instruments.

**Summary/Conclusion**: Full and detailed characterization of EV sources and types are required for safe and effective application of stem cells and/or their bioproducts in clinical application. Specific, but often superimposable results, can be obtained using different EV‐dedicated instruments.

### PS05.07 Power‐Law Characterization of EV Size Distributions

Michael Paulaitis, Johns Hopkin University School of Medicine

Olesia Gololobova, Johns Hopkins University School of Medicine

Kenneth W. Witwer, Johns Hopkins University School of Medicine

**Introduction**: A power‐law model is described for characterizing EV size distributions. The scaling exponent in this model captures the asymmetry of these size distributions, which are notably right‐skewed to larger vesicles, independent of the minimum detectable vesicle diameter.

**Methods**: This model is applied to analyze the power‐law behavior of EV size distributions measured by nanoparticle tracking analysis (NTA), microfluidic resistive pulse sensing (MRPS), nanoflow cytometry (nanoFCM), and single‐particle interferometric reflectance imaging sensing (SP‐IRIS). EVs from the human T lymphocyte line H9, the promonocytic line U937, and released apically and basally from polarized retinal pigmented epithelial cells were separated from culture media by differential ultracentrifugation and size exclusion chromatography.

**Results**: We show that the scaling exponent derived from model fits of the measured EV size distribution is sensitive to the sizing platform, the cell source, and treatment conditions, and insensitive to the minimum detectable vesicle size intrinsic to the different detection methods.

**Summary/Conclusion**: Our results establish the scaling exponent as a quantitative biophysical parameter for characterizing EV populations. In that the power‐law behavior of EV size distributions reflects changes in the membrane composition of the EVs, we can consider the measured scaling exponent to be a biophysical EV marker comparable and complementary to biochemical EV markers, such as the tetraspanins.

### PS05.08 Novel approach for quantitative real‐time nanoparticle analysis of extracellular vesicles

Marie Berger, Myrade lab

Quentin Sabbagh, INSERM, CNRS, Université de Nantes

Mathilde Richard, CRCINA INSERM U1232

Gwennan ANDRE‐GREGOIRE, CRCINA UMR1232 and Integrated Center for Cancerology (ICO)

Laetitia Guevel, INSERM, CNRS, Université de Nantes

Julie Gavard, INSERM, CNRS, Université de Nantes, Institut de Cancérologie de l'Ouest

**Introduction**: EVs are abundant and stable in body fluids (plasma, urine, breast milk…), they emerged as promising biomarkers for the assessment of health status, but also responses to treatments and outcomes in pathological conditions. As nanosized objects, research on EV requires advanced technologies and specialized expertise to assess both their specificity and sensitivity as biomarkers, as well as their efficacy and safety as therapeutic tools. This context calls for new technological improvement for measuring easily and quickly EVs concentration and size. A new method for quick and reliable EV quantification, compatible with the use of multiple larger patient cohorts and with limited volume of liquid biopsies was developped by Myriade. Based on Interferometric Light Microscopy (ILM), this method lies on its simplicity.

**Methods**: We compare the quantification of EVs (recently called Vesiclemia), by ILM and the TRPS, a well‐established method for the characterization of EVs separated from serum and biological fluids. ILM was also used on a stability study at different temperature of EVs.

**Results**: EVs were separated from 10 plasma and urine samples using size exclusion chromatography (SEC) and analyzed upon their separation, without any step of storage. Vesiclemia was estimated either by ILM and TRPS. ILM and TRPS display a linear correlation regarding EV concentration (correlation coefficient R2 = 0.95). The stability study shows EVs should be preferentially stored and quantified by Videodrop after freezing at ‐80°C.

**Summary/Conclusion**: The correlation between Videodrop analysis and the reference method TRPS appeared to be robust, with high R² values.

Videodrop is particularly adapted fulfill the pre‐requisite for characterization of EVs as non‐invasive biomarkers. This fast, real‐time titration method consuming low volumes of product turns out to be suitable for EV quantification.

### PS05.09 A semi‐quantitative assay for extracellular vesicles in vivo correlates EV overproduction with developmental phenotypes

Lauren Pitts, University of Denver

Katharina Beer, University of Würzburg

Gholamreza Fazeli, University of Würzburg

Julia Frondoni, University of Denver

Ann M. Wehman, University of Denver

**Introduction**: To clarify the roles of extracellular vesicles (EV) in vivo, it is important to visualize EVs. However, most EV reporters are also present in the releasing cell, making it challenging to visualize and count EVs in vivo.

**Methods**: To tackle these problems, we developed a technique to remove background fluorescence from the source cell. We use degradation motifs called degrons to target proteins for ubiquitination and degradation in the cytosol, while leaving EVs labelled. We use different degrons in the nematode model organism Caenorhabditis elegans and specifically label MVs by degron‐tagging the PI4,5P2‐binding PH domain of the cytosolic phospholipase PLC1∂1, which is primarily found at the plasma membrane.

**Results**: Using degron‐tagged reporters, we can visualize EVs released after fertilization as they are often trapped between different layers of the eggshell. By counting these puncta, we have developed a semi‐quantitative assay for EV release and have begun to characterize different perturbations to see their effect on EV number. For example, we have been studying the role of a lipid flippase TAT‐5 and its activating protein PAD‐1 in inhibiting EV release. Different alleles of tat‐5 and pad‐1, including FP‐knock‐ins, result in different numbers of EVs released. Intriguingly, the EV numbers correlate with cellular and developmental phenotypes such as phagocytic capacity and viability. The higher the EV numbers, the fewer endogenous cargos are successfully phagocytosed and the more likely the embryos are lethal.

**Summary/Conclusion**: Our results indicate the importance of tightly regulating the EV biogenesis machinery to avoid the deleterious effects of EV overproduction. The semi‐quantitative assay also reveals intermediate alleles, which will be useful genetic tools for enhancer/suppressor screens to identify both positive and negative regulators of EV biogenesis.

### PS05.10 Simple density‐based method for enrichment of plasma‐EVs and its use in biomarker discovery for thrombotic antiphospholipid syndrome

Marija Holcar, Institute of Biochemistry and Molecular Genetics, Faculty of Medicine, University of Ljubljana, Ljubljana, Slovenia, EU

Ula Štok, Department of Rheumatology, University Medical Centre Ljubljana, Ljubljana, Slovenia, EU

Jana Ferdin, Institute of Biochemistry and Molecular Genetics, Faculty of Medicine, University of Ljubljana, Ljubljana, Slovenia, EU

Simona Sitar, Department of Polymer Chemistry and Technology, National Institute of Chemistry, Ljubljana, Slovenia, EU

Magda Tušek‐Žnidarič, Department of Biotechnology and System Biology, National Institute of Biology, Ljubljana, Slovenia, EU

Ana Plemenitaš, Institute of Biochemistry and Molecular Genetics, Faculty of Medicine, University of Ljubljana, Ljubljana, Slovenia, EU

Ema Žagar, Department of Polymer Chemistry and Technology, National Institute of Chemistry, Ljubljana, Slovenia, EU

Vita Dolžan, Institute of Biochemistry and Molecular Genetics, Faculty of Medicine, University of Ljubljana, Ljubljana, Slovenia, EU

Snežna Sodin‐Šemrl, Faculty of Mathematics, Natural Sciences and Information Technologies, University of Primorska, Koper, Slovenia, EU

Saša Čučnik,Faculty of Pharmacy, University of Ljubljana, Ljubljana, Slovenia, EU

Polona Žigon, Department of Rheumatology, University Medical Centre Ljubljana, Ljubljana, Slovenia, EU

Metka LenassiInstitute of Biochemistry and Molecular Genetics, Faculty of Medicine, University of Ljubljana, Ljubljana, Slovenia, EU

**Introduction**: Extracellular vesicles (EVs) are promising minimally invasive biomarkers of various pathologies, but their isolation from blood is hindered by other nanoparticles (lipoproteins, proteins/protein aggregates, viruses) present in the blood. Here, we established a simple but reliable method for effective enrichment of EVs from human plasma and applied it prior to characterizing size and concentration of plasma‐EVs as biomarkers of thrombotic antiphospholipid syndrome (APS).

**Methods**: EVs were first enriched from plasma of 10 healthy subjects, using density‐based (ultracentrifugation on 20% sucrose cushion; sUC) method. Size, concentration and purity of sUC‐enriched EVs were evaluated with NTA, AF4‐MALS, TEM, ApoA1 and ApoB100 ELISA, qPCR of EV‐miRNA, and later compared to EVs enriched with size exclusion chromatography (SEC). For APS biomarker discovery, size and concentration of sUC‐enriched plasma‐EVs from 14 thrombotic APS patients, 5 aPL negative patients with idiopathic thrombosis (aPL‐ IT) and new 7 healthy subjects (HS) were determined using NTA. All subjects provided informed consent, NMEC approved the study.

**Results**: NTA detected 3.11*109 particles/mL of plasma (mode size 109 nm) and AF4‐MALS 0.66*109 particles/mL of plasma (2*Rgeom 195 nm) in samples after EV‐enrichment with sUC. The method was highly repeatable (NTARSD = 5.4%, AF4 MALSRSD = 2.1%) and resulted in more pure EVs with significantly fewer lipoprotein contaminants and more miRNA cargo compared to SEC‐enriched EVs. In the APS biomarker study, NTA showed similar EV sizes (110"170 nm) between the groups, but higher concentrations indicated enhanced shedding of EVs in patients with APS and aPL‐ IT compared to HS (p = 0.021 and p = 0.007, respectively).

**Summary/Conclusion**: The sUC method results in satisfactory purity of enriched EVs for use in biomarker studies. Quantification of plasma‐EVs enriched by sUC showed potential for future investigation of biomarkers in thrombotic APS.

### PS05.11 Rapid Separation and Detection of Exosomes based on Metal Organic Framework‐Aptamers

Bo Li, Department of Laboratory Medicine, Nanfang Hospital, Southern Medical University

Feng Jun Jie, Department of Laboratory Medicine, Nanfang Hospital, Southern Medical University

Zheng Lei, Department of Laboratory Medicine, Nanfang Hospital, Southern Medical University

**Introduction**: To construct an integrated platform for the separation and detection of specific exosomes subpopulations through a new method of metal organic framework (MOF)‐aptamers and fluorescence signal amplification and detection.

**Methods**: ZrOCl2·8H2O and organic ligand 2‐aminoterephthalic acid (NH2‐BDC) were used as raw materials, and kept at 120°C for 12 hours to synthesize MOF material UiO‐66‐NH2. A functionalized specific nucleic acid aptamer (PO34–Spaser‐PD‐L1‐Aptamer) PSPA was modified on the surface of UiO‐66‐NH2, and the modified MOF@PSPA platform was used to isolate and enrich the breast cancer cell line MDA‐MB‐ 231 expresses a specific exosomal subset of PD‐L1 membrane protein. Then, by lipid probe identification and rapid nucleic acid isothermal detection (RNAID), the captured specific exosomes subpopulations are amplified and detected by fluorescence signals.

**Results**: The established MOF@PSPA@RNAID integrated platform for exosomes separation and detection successfully separated and detected exosomes secreted by breast cancer cell lines. The detection was nonlinear, Y = 75.61*X^0.163, R2 = 0.984, The detection range reaches 4.5‐900 (105particles/μL)

**Summary/Conclusion**: MOF@PSPA based on synthetic modification can realize rapid separation and enrichment of specific exosomes subgroups. Combined with the rapid nucleic acid isothermal detection method, the difference in the number of separated exosomes subpopulations can be converted into the difference in fluorescence signal intensity. The new integrated method for separation and detection of exosomes based on the MOF@PSPA platform can be used for rapid separation and detection of specific exosomes subgroups of tumor cells.

### PS05.12 Quantification and size distribution of extracellular vesicles: a comparative study of available tools

Romain Sausset, INRAe, UMR1319, Micalis, domaine de Vilvert, Jouy en Josas, France

Eric Guédon, INRAe, Institut Agro, STLO, Rennes, France

Zuzana Krupova, Excilone, Departement R&D, 6 rue Blaise Pascal, Parc Euclide, Bat. A, 78990 Elancourt, France

Marie‐Agnès Petit, INRA (National Institute for Agronomy), France

Marianne De Paepe, INRAe, UMR1319, Micalis, domaine de Vilvert, Jouy en Josas, France

**Introduction**: It is now largely accepted that the intestinal microbiota has a key role in Intestinal Bowel Diseases (IBD). An imbalance in the composition and diversity of the intestinal microbiota (i.e. dysbiosis) of patients has been repeatedly pointed out by several teams. Production of extracellular vesicles (EVs), either by the microbiota or by intestinal epithelial cells, depends on the environment. Because of the stress due to chronic inflammation during IBD, the quantity of EVs could be more numerous in stools from patients. In order to address this question in large cohorts, we needed a quick and reliable tool to quantify extracellular vesicles.

**Methods**: Interferometric Light Microscopy (ILM), a new way to quantify nanoparticles that relies on the creation of single beam interferences between two signals from the same light path by nanoparticles such as small vesicles, was compared to Nanoparticle Tracking Analysis (NTA). For this, extracellular vesicles were purified from the stools of conventional and axenic mice and rats; and humans.

**Results**: We show that the use of ILM leads to quantification and size profiling similar to those given by NTA with all samples. In addition, ILM results were obtained with a significant time gain relative to NTA, facilitating large cohorts analyses.

**Summary/Conclusion**: ILM is particularly adapted for EV characterization, since it is a quick and handy method, consuming low volumes of product.

### PS05.13 Detection and treatment monitoring of small extracellular vesicle surface proteins in liquid biopsies of lung cancer patients by a multiplexed electrokinetic sensor

Sara Cavallaro , KTH Royal Institute of Technology

Petra Hååg, Department of Oncology/Pathology, Karolinska Institutet, Stockholm, Sweden

Kristina Viktorsson, Department of Protein Science, KTH Royal Institute of Technology, Stockholm, Sweden

Rolf Lewensohn, Department of Oncology/Pathology, Karolinska Institutet, Stockholm, Sweden

Jan Linnros, Department of Applied Physics, KTH Royal Institute of Technology, Stockholm, Sweden

Apurba Dev, Department of Electrical Engineering, Uppsala University, Uppsala, Sweden

**Introduction**: Small extracellular vesicles (sEVs) have attracted interest as a source of biomarker for cancer diagnostics and monitoring based on liquid biopsies, as they are secreted in different body fluids and their contents (proteins, RNAs, etc.) in part reflect their parent cells. While for diagnostics it is fundamental to study the genomic alterations driving a tumor, e.g. mutated EGFR for Non‐small cell lung cancer (NSCLC), multiple pathways may co‐exist in a tumor and hence contribute to treatment response. Therefore, it is also highly relevant to monitor the oncogenic markers that are responsible for these pathways. Recently, PD‐L1 on tumors has gained increasing attention, as it is related to the capacity of the immune system to attack the tumor and offers a way to therapy. It has also been suggested that PD‐L1 is expressed in sEVs and that Tyrosine Kinase Inhibitors (TKIs) against mutated EGFR‐ or EML4‐ALK, two clinically used treatments for NSCLC, may influence PD‐L1 expression in NSCLC.

**Methods**: Herein, we use our multiplexed electrokinetic platform for label‐free detection and treatment monitoring of sEV surface proteins in liquid biopsies of NSCLC patients. The technique relies on the electrokinetic phenomena of streaming current and zeta potential (z*) and measures the z* change upon sEV binding on functionalized microcapillary surfaces. The current platform can measure 3–4 channels simultaneously, but can be further extended. For the analysis, we used sEVs derived from the pleural effusions (PEs) of NSCLC patients with different genetic aberrations (EGFR, EML4‐ALK or KRAS). The vesicles were isolated by size exclusion chromatography, verified to be of sEV size by NTA and confirmed by western blot to express CD9, TSG101 but not calnexin.

**Results**: We already demonstrated that our electrokinetic sensor successfully detects sEVs, profiling sEV surface proteins up to a change of 3% in their expression levels. Moreover, the platform has been improved in order to measure multiple capillaries simultaneously. Here, we apply the multiplexed platform to detect and compare relevant tumor markers, e.g. EGFR, PD‐L1 in sEVs from PEs of NSCLC patients. Interpatient differences were evident for all markers. In ongoing studies, we are analyzing if we can monitor changes in the expression levels of these markers at different treatment stages, as well as studying the influence of TKI‐treatment on sEV PD‐L1 expression. This data will be presented.

**Summary/Conclusion**: The sensor results show successful monitoring of sEVs in liquid biopsies of NSCLC patients. With further development, the platform may be used for monitoring sEV alterations during treatments.

### PS05.14 Introducing the New ExoView Flex technology: Enabling easy, fast and robust characterization of extracellular vesicles using any antibody on the ExoView platform

Aditya Dhande, NanoView Biosciences

Dennis Zimmermann, NanoView Biosciences

George Daaboul, MDPhD, NanoView Biosciences

**Introduction**: The ExoView chip allows the characterization of single extracellular vesicles (EVs) using a combination of interferometric sizing and fluorescence imaging. Following incubation of the sample on the chip, EVs are captured on an array of target‐specific tetraspanin antibodies that are covalently linked to the chip. Once captured, these EVs can then be immunofluorescence (IF)‐stained with up to three additional probes. For users seeking to capture EVs with probes other than tetraspanins, NanoView Biosciences can provide custom chips, however this approach comes with longer lead times and higher costs.

**Methods**: Here we present NanoView Biosciences’ new ExoView Flex technology enabling one to design a single EV custom assay at the bench, allowing specific capture and detection of EVs using antibodies of choice. The assay proves to be extremely time‐efficient and the ExoView Flex Chip can be ready for sample incubation in about two hours.

**Results**: This new technology adds flexibility and has also been proven to yield highly robust and reproducible results (CV 4%). Experiments performed demonstrate a range of different EV concentrations and show a linear range of 2.5 logs. Furthermore, the ExoView Flex technology enables users to characterize EVs without purification even when challenged with matrices like biofluids and can also be used to characterize internal cargo.

**Summary/Conclusion**: This new multiplexed ExoView Flex technology allows sensitive detection of rare events in biomarker discovery while at the same time enables screening of antibody and biomarker candidates. The ExoView Flex technology is aligned with any of the core capabilities of the ExoView technology, such as purification‐free single‐particle detection, counting, phenotyping, sizing and biomarker colocalization of individual EV

and their biomarkers.

## PS06 Single‐particle Analysis

Chair: Edwin van der Pol, Biomedical Engineering and Physics, Amsterdam UMC, University of Amsterdam, Amsterdam, the Netherlands

Chair: Estefanía Lozano‐Andrés, Utrecht University, Netherlands

### PS06.01 Flow Cytometric Strategies for Reproducible Extracellular Vesicles Quantitation and Phenotyping

Gabriele De Rubis, Laboratory of Cancer Cell Biology and Therapeutics, Discipline of Pharmacy, Graduate School of Health, The University of Technology Sydney, Australia

Michael Wallach, Laboratory of Cancer Cell Biology and Therapeutics, Discipline of Pharmacy, Graduate School of Health, The University of Technology Sydney, Australia

Mary Bebawy, MD PhD, Laboratory of Cancer Cell Biology and Therapeutics, Discipline of Pharmacy, Graduate School of Health, The University of Technology Sydney, Australia

**Introduction**: Extracellular vesicles (EVs) are mediators of cell‐to‐cell communication in many pathological conditions including cancer. Their ubiquitous presence in biofluids makes them a promising source of systemic biomarkers in the field of liquid biopsies. Among the technologies employed in EVs analysis, flow cytometry (FCM) allows rapid, multiparametric characterization of EVs at single particle resolution. However, its clinical application is still hampered by limited cross‐platform reproducibility, mainly caused by the small size of EVs and by lack of standardization. Here, we describe a reproducible and sensitive EV detection and phenotyping protocol across two commercial flow cytometers designed for EVs analysis.

**Methods**: Scatter resolution, enumeration accuracy and precision of the instruments were determined by analysing submicron silica reference beads (ApogeeMix). Large EVs were enriched from the conditioned supernatant of two cancer cell lines by high‐speed centrifugation (18,900g) and characterized using electron microscopy and dynamic light scattering. EVs were stained with AnnexinV for phosphatidylserine (PS) exposure and detected by FCM, using a scatter‐based triggering strategy, using two complementary size gates: a “Latex” gate (300 to 1100 nm polystyrene beads) and a “Silica” gate (180 to 1300 nm silica beads). FCMPass software was used to estimate the size of the EVs detected by these size gates. Validation of vesicular constituents was performed, and serial dilutions analysed to assess swarm detection.

**Results**: We observed compatible scatter resolution, enumeration accuracy (error ≤15%) and precision (CV ≤10%) across both flow cytometers. Similar estimated EV sizes are detected by the two instruments in both size gates of interest according to FCMPass software modelling. We obtained linear and cross‐platform reproducible detection of PS+ EVs, with inter‐instrument CV≤20% for the “Latex” gate and ≤10% for the “Silica” gate, across an EV range of 0.125 ‐ 2 μg/mL. This corresponds to 10–800 PS+ EVs/μL in the “Latex” gate and 500–10,000 PS+ EVs/μL in the “Silica” gate. Larger EVs amounts resulted in loss of linearity in the “Silica” gate in one of the two instruments, indicating interference of swarm detection for EVs amounts >2 μg/mL.

**Summary/Conclusion**: Our results show the cross‐platform reproducible FCM analysis of large EVs using a scatter‐based triggering approach. This work provides the basis for the development of robust protocols for clinically viable EV‐based liquid biopsy tests.

### PS06.02 Nanoscale imaging and analysis of cerebrospinal fluid derived single EVs

Shivani Sharma, PhD, University of California Los Angeles

**Introduction**: EVs circulating in cerebrospinal fluid (CSF) offers unique ‘nanoscale windows’ into brain tumors, but the low abundance and nanometer‐scale dimensions of EVs pose challenges. Uncertainties exist with due to different isolation techniques employed, as well as the rigor and reliability of EV characterization. Also, there is a need to pool individual CSF for downstream bio‐molecular analysis. Besides evaluating the downstream proteomic and genomic cargoes of CSF‐derived EVs, information on quantitative, high resolution, structural‐mechanical properties of CSF EV isolates, and the impact of isolation techniques is scarce.

**Methods**: Using minimal (less than 100 micro‐liter) volumes of CSF, we successfully compared EVs isolated via different isolation methods within the same patient samples, in replicates using atomic force microscopy.

**Results**: Our results provide new biophysical insights into the effects of isolation techniques on single EVs reveal a combination of size exclusion and precipitation as the most optimal method for label‐free isolation of unperturbed EV particles from limited CSF samples, compared to either method alone.

**Summary/Conclusion**: Our study on nanoscale EV structural‐mechanical analysis in glioblastoma and other CSF samples highlights the novel potential implications for AFM technology in improved isolation, quantification, and single vesicle characterization of CSF EVs for glioblastoma (and other brain associated) biomarkers.

### PS06.03 Quantitative multi‐parameter analysis of individual fecal extracellular vesicle via a laboratory‐built nano‐flow cytometer

Haisheng Liu, Department of Chemical Biology, College of Chemistry and Chemical Engineering, Xiamen University, Xiamen, Fujian 361005, China

Yongyu Chen, Department of Chemical Biology, College of Chemistry and Chemical Engineering, Xiamen University, Xiamen, Fujian 361005, China

Yuhang Qin, Department of Chemical Biology, College of Chemistry and Chemical Engineering, Xiamen University, Xiamen, Fujian 361005, China

Xiaomei Yan, PhD, Department of Chemical Biology, College of Chemistry and Chemical Engineering, Xiamen University

**Introduction**: Fecal extracellular vesicles (fEVs) has been implicated in physiological processes in various diseases and host immune response. To further explore their prominent biological potential, an in‐depth study of fEVs at the single‐particle level is important. Employing a laboratory‐built nano‐flow cytometer (nFCM) that facilitates multiparameter analysis of single EVs as small as 40 nm, here we report quantitative measurement of size distribution, purity, nucleic acids and surface markers of fEVs.

**Methods**: fEVs were isolated from stool samples collected from healthy donors via iodixanol gradient ultracentrifugation. Six fractions of 2 mL each were collected from the top of the tube (F1 " F6). TEM was used to characterize the morphology of fEVs. Monodisperse silica nanoparticles were used as the size reference standards for the size distribution measurement of fEVs via light scattering detection. The purity of fEVs was examined by measuring the particle concentration before and after Triton X‐100 treatment. Subpopulation of fEVs expressing specific surface markers, such as CD9, CD63, CD81, CD24, lipopolysaccharide (LPS) and lipoteichoic acid (LTA) were analyzed via immunofluorescent staining. SYTO 16, a cell‐permeant stain, was used to stain the DNA of fEVs before and after DNase I treatment.

**Results**: The purity of isolated F1 ‐ F6 fEVs via density gradient UC was ranging from 55.1% ‐ 93.2%. We found that there was almost no expression of CD9, CD63, CD81 and CD24 for fEVs. We also found that ∼20% of fEVs expressing LPS (the marker of EVs from gram‐negative bacteria) or LTA (the marker of EVs from gram‐positive bacteria). The ratio of fEVs that can be fluorescently stained by SYTO 16 had no obvious change after DNase I treatment, suggesting that all the EV‐DNA of fEVs resides in the lumen of EVs.

**Summary/Conclusion**: The laboratory‐built nFCM is applicable to the multiparameter biochemical analysis of individual fEV via protein and nucleic acid staining. We expect nFCM will facilitate more in‐depth studies of fEVs.

### PS06.04 Single extracellular vesicle analysis performed by imaging flow cytometry in contrast to NTA rigorously assesses the accuracy of urinary extracellular vesicle preparation techniques

Marvin Droste , Department of Pediatrics II (Pediatric Nephrology), University Hospital Essen, University of Duisburg‐Essen, Essen, Germany

Tobias Tertel, Institute for Transfusion Medicine, University Hospital Essen, Germany

Stefanie Jeruschke, Department of Pediatrics II (Pediatric Nephrology), University Hospital Essen, University of Duisburg‐Essen, Essen, Germany

Robin Dittrich, Institute for Transfusion Medicine, University Hospital Essen, Germany

Evangelia Kontopoulou, Department of Pediatrics III, University Hospital Essen, University of Duisburg‐Essen, Essen, Germany

Bernd Walkenfort, Electron Microscopy Unit, Imaging Center, University Hospital Essen, University of Duisburg‐Essen, Essen, Germany

Verena Börger, Institute for Transfusion Medicine, University Hospital Essen, University of Duisburg Essen, Essen, Germany

Peter F. Hoyer, Department of Pediatrics II (Pediatric Nephrology), University Hospital Essen, University of Duisburg‐Essen, Essen, Germany

Anja K. Büscher, Department of Pediatrics II (Pediatric Nephrology), University Hospital Essen, University of Duisburg‐Essen, Essen, Germany

Basant K. Thakur,Department of Pediatrics III, University Hospital Essen, University of Duisburg‐Essen, Essen, Germany

Bernd Giebel, Prof, Institute for Transfusion Medicine, University Hospital Essen, Germany

**Introduction**: Urinary small extracellular vesicles (sEVs) are studied as potential biomarkers. They can be enriched with different protocols resulting in significant variance regarding purity and yield. Assuming that the evaluation of EV preparation methods largely depends on the applied analysis tools, obtained samples were analyzed by different techniques, i.e., by imaging flow cytometry (IFCM), conventional nanoparticle tracking analysis (NTA), Western Blot (WB) and transmission electron microscopy (TEM).

**Methods**: Cell‐free urine was first screened for the presence of CD9+, CD63+ and CD81+ objects by IFCM. Urinary sEVs were then prepared from healthy donor void urine applying five different methods based on combinations of frequently used EV preparation techniques: polyethylene glycol (PEG)‐precipitation + ultracentrifugation (UC), PEG + size exclusion chromatography (SEC), UC + SEC, ultrafiltration (UF) + SEC, or the commercial ExoEasy Maxi kit. We determined the obtained amount of CD9‐labeled sEVs as well as the average particle numbers by conventional NTA. Furthermore, we assessed the intensity of the bands of TSG101 and the contaminant protein uromodulin (UMOD) in WBs. The morphology of the particles was documented by TEM.

**Results**: Urine contains a prominent population of CD9+ sEVs, but hardly any CD63+ and CD81+ sEVs. The number of recorded CD9‐positive objects detected by IFCM correlated with the TSG101 WB band intensities. In contrast, average particle numbers as determined by conventional NTA correlated with the intensity of UMOD WB bands.

**Summary/Conclusion**: Overall, our data question the reliability of conventional NTA analyses for identifying the optimal EV preparation method. In our method comparison, the combination of SEC and UF showed the highest CD9+ object and TSG101 protein recovery, and in relation to the number of CD9‐positive objects, the lowest amount of UMOD contamination.

### PS06.05 Considerations towards flow cytometric calibration of fluorescent signals from nanoparticles and extracellular vesicles by using MESF‐bead based calibrators

Estefanía Lozano‐Andrés, Utrecht University

Tina Van den Broeck, BD Biosciences, Erembodegem, Belgium

Lili Wang, Biosystems and Biomaterials Division, National Institutes of Standards and Technology (NIST), Gaithersburg, MD 20899

Majid Mehrpouyan, BD Biosciences, 2350 Qume Drive, San Jose, CA 95131

Marca H.M. H.M. Wauben, Department of Biomolecular Health Sciences, Utrecht University, The Netherlands

Ger. J.A. Arkesteijn, Department of Biomolecular Health Sciences, Faculty of Veterinary Medicine, Utrecht University, Utrecht, The Netherlands

**Introduction**: low cytometry is a powerful technique to characterize nanoparticles (NP) and Extracellular Vesicles (EV). However, in the majority of reported experiments, arbitrary units are used to indicate fluorescence intensity. This hampers comparison of results from different laboratories and different platforms. We investigated the use of calibrated Molecules of Equivalent Soluble Fluorophores (MESF)‐beads designed for cell‐based flow cytometric analysis for assignment of absolute fluorescence to NP and EV.

**Methods**: FITC‐MESF and PE‐MESF bead sets of 2 μm and 6 μm were evaluated and used as calibrators on different platforms (BD Influx, CytoFLEX, SORP BD FACSCelesta). 550 nm silica NP with six different FITC fluorescent intensities were used as a synthetic NP sample. EV were isolated from conditioned media of the 4T1 mammary carcinoma cell line (dUC), stained with CFSE and CD9PE followed by density gradient floatation. Various EV‐densities were collected and further analysed. Synthetic NP and stained EV were measured on the BD Influx and their respective fluorescent signals were calibrated in standardized units of FITC‐MESF, CFSE‐ERF (Equivalent Reference Fluorophores) and PE‐MESF.

**Results**: Fluorescence calibration, using two different sizes, brightly fluorescent calibrators designed for cell‐based flow cytometry, makes inter‐platform comparison possible. However, MESF numbers based on extrapolation into the dim fluorescence range of NP and EV vary depending on the MESF‐bead based set used. These variations ranged from 27.3 to 76.5% when calibrating different FITC fluorescent signals from synthetic NP in FITC‐MESF units and were 78.6% and 156.9% respectively, when calibrating CFSE‐ERF and PE‐MESF signals of CFSE and CD9PE stained biological EV.

**Summary/Conclusion**: The differences in the slopes of the regression lines between different calibrator bead sets, caused by uncertainties in the assignment of MESF to the calibrators, are exaggerated during extrapolation through linear regression into the dimmer fluorescent area. For proper assignment of low fluorescent sub‐micron particles such as EV, calibrator beads with high accuracy of MESF assignment are required; preferably in the fluorescence range of EV. Furthermore, for robust interpretation and for benchmarking studies the use of the same calibration materials is advised when possible.

### PS06.06 Comparison of extracellular vesicle isolation and storage methods using high‐sensitivity flow cytometry

Sarah Deville, Flemish Institute for Technological Research (VITO), Health Unit, Boeretang 200, 2400 Mol, Belgium

Pascale Berckmans, VITO nv

Rebekka Van Hoof, KU Leuven, UHasselt, VITO

Ivo Lambrichts, Hasselt University

Anna Salvati, University of Groningen

Inge Nelissen, VITO nv

**Introduction**: Extracellular vesicles (EVs) are membrane‐bound carriers with complex cargoes which are released by most biological cells and are of interest for a wide variety of applications, including the early monitoring of diseases, as primary therapeutics and as drug delivery vehicles. Flow cytometry is emerging as a very promising technology for EV characterization due to its high throughput and multiplex fluorescence possibilities. As the use of high‐sensitivity flow cytometry and the availability of fluorescent dyes for labeling of EV subsets are expanding, there is a growing need for standardization efforts to enhance the reproducibility of measurements. A critical aspect which is poorly investigated, is the influence of EV storage conditions on the EV concentration and the stability of EV‐associated fluorescent labels.

**Methods**: We used EVs from lipopolysaccharide‐stimulated monocytic THP‐1 cells which were obtained by two different EV isolation methods, differential centrifugation and exoEasy membrane affinity spin column purification. The EV fractions were stored at 4°C and ‐80°C for up to one month, and EV concentrations were evaluated at regular time intervals using scatter‐based nanoparticle tracking analysis (NS500) and flow cytometry (BD Influx) after fluorescent EV labeling with CFDA‐SE and PKH67.

**Results**: Unlabeled THP‐1 cell‐derived EVs remained relatively stable after one month of storage at both 4°C and ‐80°C. When storing CFDA‐SE‐ and PKH67‐labelled EVs, those kept at 4°C lost their fluorescence intensity within one day, while the EVs stored at ‐80°C remained stable over time.

**Summary/Conclusion**: Good practice in EV sample storage is a major determinant for standardization of fluorescence‐based analysis methods, such as flow cytometry.

### PS06.07 Plasmon‐enhanced detection and molecular profiling of single extracellular vesicles

Taehwang Son, Massachussets General Hospital

Jouha Min, Massachusetts General Hospital

Jae‐Sang Hong, Massachusetts General Hospital

Ralph Weissleder, Massachusetts General Hospital

Hakho Lee, Massachusetts General Hospital

Hyungsoon Im, Massachusetts General Hospital

**Introduction**: Extracellular vesicle (EV) analyses have shown the potential for molecular cancer diagnosis from non‐invasive liquid biopsies. Considering EVs’ physical and molecular heterogeneity, a sensitive and robust platform with a single EV analysis capability is still needed to further explore EVs’ potential as biomarkers and accelerate their clinical adaption. However, multiplexed single EV analysis is technically challenging due to EVs’ small sizes and weak optical signals. Here, we report a nanoplasmonic platform based on plasmon‐enhanced fluorescence (PEF) detection for single EV analyses.

**Methods**: Periodic Au nanohole arrays were employed for PEF detection where excitation/emission wavelengths of fluorophore overlap with plasmon resonance wavelength. EVs are captured on the nanohole surface and labeled by fluorescently labeled antibodies. We used a conventional fluorescence microscope for single EV imaging and detected both surface and intravesicular markers. We identified EVs by signals of a tetraspanin combination (CD9, CD63, and CD81), and those from glioblastoma cancer cells by signals for EGFR. Then we detected EGFRvIII mutation proteins from the cancer‐derived EV population. We reported the fraction for target markers among colocalized spots positive to EGFR and the tetraspanin combination.

**Results**: The PEF signals of nanohole arrays were characterized by a streptavidin monolayer conjugated with 4 different fluorophores (AF488, Cy3, Cy5, and Cy5.5). The strongest enhancement was achieved at the Cy5 channel with a 23‐fold signal enhancement compared to a plain Au. When we captured biotinylated EVs on glass and nanohole substrates and labeled them with streptavidin‐Cy5, we showed a one‐order enhancement in the numbers and intensities of EVs on Au nanoholes compared to glass. This indicates the plasmon enhancement unveils EVs with weak fluorescence signals otherwise undetected without signal enhancement (glass substrates). The feasibility of its clinical application was tested using EVs from Gli36‐WT and Gli36‐EGFRvIII cell lines spiked in human plasma. We successfully identified cancer cell‐derived EVs by EGFR signals and detected EGFRvIII signals only from Gli36‐EGFRvIII EVs.

**Summary/Conclusion**: We developed PEF detection for multiplexed EV molecular profiling. Fluorescence signals from multiple channels were amplified simply using Au nanohole substrates. Especially, a marker expected to be low abundant can be strategically assigned to the strongest signal enhanced channel. This approach will provide a better understanding of the molecular heterogeneity of EVs and could improve the robustness and accuracy of EV‐based cancer detection.

### PS06.08 Multiscale characterisation approach to uncover the correlation between isolation methods, physicochemical composition and biological function of extracellular vesicles

Huyen T. Phan, The University of Sydney, Sydney Nano Institute, School of Pharmacy, Faculty of Medicine and Health

Shiva Kamini Divakarla, The University of Sydney, Sydney Nano Institute, Faculty of Medicine and Health, Sydney School of Pharmacy

Jia Hao Yeo, The University of Sydney, School of Chemistry, Camperdown, NSW 2006, Australia.

Qingyu Lei, The University of Sydney, Sydney Nano Institute, Faculty of Medicine and Health, Sydney School of Pharmacy

Priyanka Tharkar, The University of Sydney, Sydney Nano Institute, Faculty of Medicine and Health, Sydney School of Pharmacy

Taisa Pansani, UNESP – Univ. Estadual Paulista, Araraquara School of Dentistry, Department of Dental Materials and Prosthodontics, Araraquara, Centro 14801–903, Brazil

Karthryn G. Leslie, The University of Sydney, School of Chemistry, Camperdown, NSW 2006, Australia.

Maggie Tong, The University of Sydney, School of Chemistry, Camperdown, NSW 2006, Australia.

Victoria Coleman, National Measurement Institute Australia, Nanometrology Section, Lindfield, NSW 2070, Australia

Åsa Jamting,National Measurement Institute Australia, Nanometrology Section, Lindfield, NSW 2070, Australia.

Mar‐Dean Du Plessis, National Measurement Institute Australia, Nanometrology Section, Lindfield, NSW 2070, Australia.

Elizabeth NewThe University of Sydney, Sydney Nano Institute, Faculty of Science, School of Chemistry, Camperdown, NSW 2006, Australia

Bill Kalionis, Department of Maternal‐Fetal Medicine Pregnancy Research Centre and University of Melbourne Department of Obstetrics and Gynaecology, Royal Women's Hospital, Parkville, VIC 3052, Australia

Philip Demokritou,Harvard T.H Chan School of Public Health, Center for Nanotechnology and Nanotoxicology, Department of Environmental Health, MA 02115, USA.

Hyun‐Kyung Woo,Center for Soft and Living Matter, Institute for Basic Science (IBS), Ulsan 44919, Republic of Korea.Department of Biomedical Engineering, Ulsan National Institute of Science and Technology (UNIST), Ulsan 44919, Republic of Korea.

Yoonkyoung Cho,Center for Soft and Living Matter, Institute for Basic Science (IBS), Ulsan 44919, Republic of Korea.Department of Biomedical Engineering, Ulsan National Institute of Science and Technology (UNIST), Ulsan 44919, Republic of Korea.

Wojciech Chrzanowski,The University of Sydney, Sydney Nano Institute, Faculty of Medicine and Health, Sydney School of Pharmacy

**Introduction**: Extracellular vesicles (EVs) have been lauded as next generation medicines, but very few EV‐based therapeutics have progressed to clinical use. Limited clinical translation is largely due to technical barriers that hamper our ability to mass‐produce EVs, i.e. to isolate, purify and characterise them effectively. Technical limitations in comprehensive characterisation of EVs leads to unpredicted biological effects of EVs.

**Methods**: To measure EVs size and concentration we used Particle Tracking Analysis (PTA), Dynamic Light Scattering (DLS), Nano‐flow Cytometry (nFCM), Tunable Resistive Pulse Sensing (TRPS), and Asymmetric Flow‐Field fractionation (AF4). Nanoscale infrared spectroscopy (AFM‐IR) and nFCM were used to determine EV composition at the single EV and EV sub‐population levels. For the first time, we used Resonant Mass Measurement (RMM) for the characterisation of dry mass and buoyant mass of large EVs (>100 nm) and distorted grid (DG) for the sedimentation prediction of EVs. The actual functional effects of EV isolates on cells was determined using newly developed nitric oxide fluorescent probe to measure intracellular stress in an in vitro model of acute lung injury.

**Results**: Here, using a range of optical and non‐optical techniques, we showed that the differences in molecular composition of EVs isolated using two isolation methods correlated with the differences in their biological function. Our results demonstrated that the isolation method determines the composition of isolated EVs at single and sub‐population levels. Besides the composition, we measured for the first time the dry mass and predicted sedimentation of EVs. These parameters were shown to correlate well with the biological and functional effects of EVs on single cell and cell cultures.

**Summary/Conclusion**: We anticipate that our multiscale characterisation approach will support fundamental understanding of EVs as well as elucidate the functional effects of EVs in in vitro and in vivo studies. Our findings and methodology will be pivotal for developing optimal isolation methods and establishing EVs as mainstream therapeutics and diagnostics. This innovative approach is applicable to a wide range of sectors including biopharma and biotechnology as well as to regulatory agencies.

### PS06.09 Extracellular vesicles: not only size matters

Pietro Parisse, Istituto Officina dei Materiali‐CNR

**Introduction**: The elucidation of biophysical and biochemical characteristics of EVs is crucial for the understanding of their interaction with recipient cells and their functional activity. In particular, for therapeutic applications understanding how the process of manufacturing affects the biological function of EVs is mandatory before going to the clinical testing. The absence of standardized methodologies and technologies to establish reliable criteria has been the main hurdle for real therapeutic applications of EVs.

**Methods**: We focused on the analysis of biophysical properties of standardized Umbilical Cord‐MSC‐EVs preparations, to help elucidating the role of phenotypic parameters (size,morphology, structure, protein/lipid ratio) in view of possible therapeutic applications. In particular we combined different techniques to capture morphological, structural and chemical information on EVs isolated with different protocols (Tangential Flow Filtration, Ultracentrifugation, Size Exclusion Chromatography). We exploited the nanometer resolution of Atomic Force Microscopy (AFM) to visualize the morphology of single EVs combined to structural information from Small Angle Scattering experiments and to the chemical information from vibrational spectroscopies, namely Fourier Transform Infrared and Ultraviolet Resonant Raman spectroscopies, to go beyond the mere size analysis and capture novel insights on the molecular contamination/stability of the different EVs preparation.

**Results**: We evidenced that size distribution analysis is not sufficient to distinguish different preparations, but that the structural, biophysical and chemical fingerprints of EVs and their co‐isolation products need to be taken into account to address stability and purity issues. The structural insights obtained by scattering techniques allow for a detailed description of the EVs bilayer structure; AFM reveals the presence of particles smaller than 50 nm, usual limit for standard optical techniques, allowing to distinguish vesicles from other particles based on their morphology and nanomechanical behavior; vibrational spectroscopies allow the extraction of protein/lipid ratio giving an easy and fast screening of sample purity.

**Summary/Conclusion**: Our results point towards the necessity of: a multi parametric analysis, capable of giving a wide overview of the biophysical properties of EVs; monitoring the morphology of EVs also in native environment; evaluating not only size, but also purity and stability of the EV preparations.

### PS06.10 The Impact of Limits of Detection (LOD) on Studies of Extracellular Vesicles (EVs) Using Flow Cytometry

Sabrina La Salvia, SL, Icahn School of Medicine at Mount Sinai

Luca Musante, University of Virginia

Emily M. Heiston, EM, 1University of Virginia, Charlottesville, VA.

Nathan R. Stewart, NRS, Rutgers University, New Brunswick, NJ.

Steven K. Malin, SKM, University of Virginia

Uta Erdbrügger, UE, University of Virginia

Joanne Lannigan, JL, Cytek Biosciences

**Introduction**: Use of flow cytometry (FC) is one of the most commonly used technologies to study EVs isolated from various sources and a tool for understanding the role of EVs in clinical pathologies. There is a great deal of discordance in the literature regarding the size and frequencies of EVs from clinical samples. In this study, we sought to understand the impact of two flow cytometers with different LOD on size, phenotype, and concentration of EVs isolated from patient's samples.

**Methods**: Plasma samples were collected from 20 adults with metabolic syndrome after an overnight fast before and after testing for insulin sensitivity (n = 10) and a treadmill exercise test (n = 10). Plasma was centrifuged at 5000 g for 15 min and supernatant (SN) at 17,000g for 10 min. Samples were labeled with CD9,63,81, FITC, CD105 PE, CD31 AF647, CD41 PacBlue, and CD45 PE‐Dazzle594, split in two aliquots and acquired on a standard Cytek 5 laser Aurora and a 5 laser Aurora modified with a small particle enhancement option. Both instruments were calibrated for size (nm) and fluorescence (MESF) using FCMPass (nanopass.ccr.cancer.gov). Size was calibrated using NIST certified polystyrene beads (Series 3000 " ThermoFisher) and [Quantum MESFTM beads; Bangs Laboratories].

**Results**: Size (315‐330nm vs. 122–126nm) and concentration of total events < 1 u (2.2‐6.5E9/mL vs. 6.8E10‐1.5E11/mL) were statistically different across instruments (p < 0.05). Additionally, differences in the sizes of the major subsets (CD31, CD45, CD105) were also significant (p < 0.05), with the exception of CD41+ EVs. The Tetraspanin negative subsets were significantly smaller for the subtype CD45 and CD105. However, no significant difference was observed in the concentrations when comparing same samples across instruments or between patient groups or time points. Interestingly, the trends in the patient groups as well as the trends in changes across time points appeared to be similar, suggesting some level of reproducibility.

**Summary/Conclusion**: EV size and concentration detection using FC can be greatly impacted by an instrument's LOD. In order to compare results across instruments, it is imperative the instruments be calibrated to determine the LOD for size and fluorescence and results reported in the context of this information. Deeper subset analysis regarding size concentrations and MESF values is needed

### PS06.11 New kid on the block: Nano‐flow cytometry measures up against first‐generation technologies for EV physical characterisation

Ben Peacock, NanoFCM

Adriele Prina, Trinity College Dublin

Alice Law, NanoFCM

Dimitri Aubert, NanoFCM

Robert Vogel, PhD, Izon

**Introduction**: Physical characterisation of extracellular vesicles, and other polydisperse complex biological samples, is critical to assess variation in quality deriding from their production and isolation. We recently published data analysing polystyrene nanoparticles as well as EVs using six applied techniques include multi‐angle dynamic light scattering (MADLS), asymmetric flow field flow fractionation coupled with multi‐angle light scattering (AF4‐MALS), centrifugal liquid sedimentation (CLS), nanoparticle tracking analysis (NTA), tunable resistive pulse sensing (TRPS), and high‐sensitivity nano flow cytometry (nFCM).

**Methods**: Ability to detect and distinguish particles of different size and concentration was investigated using monomodal samples and complex polystyrene mixtures allowing for development of reliable post‐processing data protocol. Liposomes with known physicochemical properties closer to EVs, as well as EV containing plasma samples were analysed with all the tested techniques providing insight into the measurement of biological nanoparticles.

**Results**: Only nFCM and TRPS were capable of detecting the smallest populations of Polystyrene populations also distinguishing them in mxture. Liposome, concentrations and size distributions, as measured with NTA, TRPS, and AF4‐MALS were in good agreement (with a coefficient of variance of 27%), whereas nFCM measured a significantly lower. EV concentrations and size distributions of plasma EVs were generally in agreement for NTA, TRPS and nFCM measurements highlighting that single particle analysis techniques are well suited to measure particle concentration of biological samples such as EVs. For CLS the measured concentration was significantly higher compared with the single particle analysis techniques, and for AF4‐MALS the concentration was below the acceptable threshold for size and concentration measurements,

**Summary/Conclusion**: The data should help researchers decide which methodologies to implement in their own research, with additional information on ease of use and technical parameters included within the manuscript.

Declaration of Interest Statement

RV is a contractor at IZON Science, JM and MM are employed by IZON Science and their contributions to this paper were made as part of their contract/employment.

AL, BP, DA are employees of NanoFCM and their contributions to this paper were made as part of their employment.

### PS06.12 Direct Measurement of Small Extracellular Vesicles in Unprocessed Human Plasma by Imaging Flow Cytometry

Wouter W. Woud, Nephrology and Transplantation, Department of Internal Medicine, Erasmus MC, University Medical Center Rotterdam, The Netherlands

Erik Mul, Department of Blood Cell Research, Sanquin Research and Landsteiner Laboratory, Academic Medical Center, University of Amsterdam, Amsterdam, the Netherlands

Martin Hoogduijn, Nephrology and Transplantation, Department of Internal Medicine, Erasmus MC, University Medical Center Rotterdam, The Netherlands

Carla Baan, Nephrology and Transplantation, Department of Internal Medicine, Erasmus MC, University Medical Center Rotterdam, The Netherlands

Karin Boer, Nephrology and Transplantation, Department of Internal Medicine, Erasmus MC, University Medical Center Rotterdam, The Netherlands

Ana Merino, Nephrology and Transplantation, Department of Internal Medicine, Erasmus MC, University Medical Center Rotterdam, The Netherlands

**Introduction**: Characterization of EVs is hampered by their small size, low epitope copy number and the use of different isolation methods which may modify the EVs of interest. Analysis of EVs in human plasma is even more complicated due to the molecular complexity of plasma (e.g. lipoproteins and soluble factors). In recent years, Imaging Flow Cytometry (IFCM) has emerged as a potential technique that is sensitive enough to discriminate and analyse single EVs. Here we present an easy to use sample protocol without prior purification / isolation of EVs from plasma samples. Additionally, IFCM instrument settings as well as a set of controls are provided to accurately quantify, phenotype and visualize single human plasma derived small EVs (< 300 nm, sEVs) while excluding potential artefacts.

**Methods**: Platelet‐Poor Plasma (PPP) from 5 healthy individuals was stained directly without prior purification / isolation of EVs with CFSE and antibodies directed against proteins of interest including tetraspanins and CD31. Strict controls were used for each sample to allow for the accurate detection of sEV signatures. Acquisition was performed by running each sample for 3 minutes on an ImageStreamX (ISX) MkII IFCM.

**Results**: Fluorescent background levels of the IFCM were established and a gating strategy to accurately analyse sEVs was developed. sEV signatures in healthy individuals were identified as double‐positive events expressing a tetraspanin marker (CD9/CD63/CD81) in conjunction with enzyme activity (CFSE+) or presence of a cellular origin marker (CD31+); 2.7E6 ± 1.64E6 objects/mL and 3.57E6 ± 6.99E5 objects/mL respectively. Detergent lysis was performed to enable discrimination of biological signals from artefacts. Serial dilution experiments demonstrated accurate quantification of single sEVs.

**Summary/Conclusion**: We successfully developed a method to discriminate, identify and quantify sEVs in complex mixtures such as human plasma without prior purification or isolation of EVs. In this work, we propose a set of criteria for events to be classified as true sEVs by IFCM.

### PS06.13 Imaging flow cytometry‐based detection of small extracellular vesicles in the synovial fluid of Rheumatoid Arthritis and Osteoarthritis patients

Edveena Hanser, University of Basel

Diego Kyburz, University of Basel

**Introduction**: Rheumatoid arthritis (RA) is an autoimmune disease characterized by chronic joint inflammation and progressive destruction of cartilage and bone leading to severe pain and disability. A number of publications indicate either a pro or anti‐inflammatory role of extracellular vesicles (EVs) in RA and OA. EVs are produced by almost all cells. EVs have gained significant interest as biomarkers in health and disease. These nanobioparticles are believed to transfer cargo consisting of protein, lipids, nucleic acids, thus facilitating communications among cells. Here, we have optimized a method to detect small EVs called exosomes in the synovial fluid (SF) of RA and OA patients using imaging flow cytometry (iFCM)

**Methods**: Synovial fluid was collected from RA (n = 6) and OA (n = 4) patients after obtaining their written informed consent. EVs were isolated from hyaluronidase‐treated cell free SF by size exclusion chromatography (SEC) using iZON qEVoriginal size exclusion columns. The fractions were pooled in and concentrated by using Amicon ultra4 10KDa cellulose ultrafiltration filter units. The concentration and size determination of enriched EVs was determined by ZetaView(R) nanoparticle tracking analyzer (NTA). The concentration of EV samples were adjusted for staining with fluorescently labelled antibodies CD63 and CD9. The samples were acquired by imaging flow cytometry (iFCM) using Amnis(R) Imagestream(R) MK II imaging flow cytometry and analyzed by IDEAS(R) and FCS Express software. The EVs were also subjected to negative staining for transmission electron microscopy

**Results**: The transmission electron micrograph (TEM) showed the presence of EVs isolated from synovial fluid of RA and OA patients.

Imagestream(R) depicted small EVs labelled with fluorescently conjugated antibodies CD63 and CD9, which are the surface markers of exosomes. The various controls and other calibration parameters as stated in MIFlowCytEV‐ reporting framework was taken into account while conducting experiments to ensure the standardization and reliability of data

**Summary/Conclusion**: We have shown here an optimized method to detect small EVs present in the synovial fluid of RA and OA patients using imaging flow cytometry‐based technique. The protocol uses iFCM to identify small EVs, it combines high fluorescence sensitivity, image confirmation ability and powerful data analysis tools. As EVs carry markers of their parent cells and reflect their parent cells from where they originate because their membrane orientation is the same as that of the donor cell. Thus, they can be considered to be miniature versions of a cell. This property of EVs can be exploited in the search of biomarkers for diagnosis, prognosis, therapeutic potential and imaging flow cytometry can be used to identify EV origin via targeted and high‐throughput phenotyping

## PS07 EVs in neurodegenerative diseases

Chair: Efrat Levy, Center for Dementia Research, Nathan S. Kline Institute, Orangeburg, New York 10962, USA

Chair: Berta Puig, UKE, Germany

### PS07.01 The role of Extracellular Vesicles (EVs) in Amyotrophic Lateral Sclerosis (ALS) and Frontotemporal Lobar Degeneration (FTLD)

Elena Casarotto, Dipartimento di Scienze Farmacologiche e Biomolecolari (DiSFeB), Department of excellence 2018–2022, University of Milan, Milan (Italy)

Daisy Sproviero, Mondino Foundation – IRCCS, Pavia (Italy)

Stella Gagliardi, Mondino Foundation – IRCCS, Pavia (Italy)

Eleonora Corridori, Mondino Foundation – IRCCS, Pavia (Italy)

Fabrizio Fabbiano, Centre of Integrative Biology (CIBIO), University of Trento, Trento (Italy)

Maria Cristina Gagliani, University of Genoa, Genoa (Italy)

Marta Cozzi, Dipartimento di Scienze Farmacologiche e Biomolecolari (DiSFeB), Department of excellence 2018–2022, University of Milan, Milan (Italy)

Barbara Tedesco, Dipartimento di Scienze Farmacologiche e Biomolecolari (DiSFeB), Department of excellence 2018–2022, University of Milan, Milan (Italy)

Riccardo Cristofani, Dipartimento di Scienze Farmacologiche e Biomolecolari (DiSFeB), Department of excellence 2018–2022, University of Milan, Milan (Italy)

Veronica Ferrari,Dipartimento di Scienze Farmacologiche e Biomolecolari (DiSFeB), Department of excellence 2018–2022, University of Milan, Milan (Italy)

Marta Chierichetti, Dipartimento di Scienze Farmacologiche e Biomolecolari (DiSFeB), Department of excellence 2018–2022, University of Milan, Milan (Italy)

Paola RusminiDipartimento di Scienze Farmacologiche e Biomolecolari (DiSFeB), Department of excellence 2018–2022, University of Milan, Milan (Italy)

Mariarita Galbiati, Dipartimento di Scienze Farmacologiche e Biomolecolari (DiSFeB), Department of excellence 2018–2022, University of Milan, Milan (Italy)

Vito D'agostino, Centre of Integrative Biology (CIBIO), University of Trento, Trento (Italy)

Katia Cortese,University of Genoa, Genoa (Italy)

Cristina Cereda, Genomic and post‐Genomic Unit, IRCCS Mondino Foundation, Pavia, Italy

Angelo Poletti, Dipartimento di Scienze Farmacologiche e Biomolecolari (DiSFeB), Department of excellence 2018–2022, University of Milan, Milan (Italy)

Valeria Crippa, Dipartimento di Scienze Farmacologiche e Biomolecolari (DiSFeB), Department of excellence 2018–2022, University of Milan, Milan (Italy)

**Introduction**: ALS and FTLD are neurodegenerative diseases characterized by pathological ubiquitinated and phosphorilated inclusions in the cytosol of affected cells. In 98% of ALS and in the majority of Tau‐negative FTLD cases the main component is the TAR DNA‐binding protein of 43 KDa (TDP‐43) together with its C‐terminal fragments of 35 (TDP‐35) and 25 KDa (TDP‐25).

TDP‐inclusions are mainly removed from cells via the protein quality control (PQC) system, but they could also be secreted within extracellular vesicles (EVs).

In our work we first analysed the TDP‐content of the EVs, by comparing large (LVs) with small vesicles (SVs); then, we evaluated the presence of some PQC‐members. Finally, we investigated the effect of PQC blockage on EVs secretion and content.

**Methods**: We isolated EVs produced by NSC34 cells untreated or treated with MG132 or NH4Cl (proteasome and autophagy inhibitors). To isolate EVs we used the differential ultracentrifugation method. We analysed EVs size, count and morphology through the Nanoparticle Tracking Analysis and the transmission electron microscopy, and their protein content through western blot analysis.

**Results**: We showed that both TDP‐43 and its C‐terminal fragments (especially TDP‐35) are secreted in EVs, mainly in LVs. Interestingly, in cells TDPs are present as soluble forms, instead the secreted TDPs are mainly insoluble. We found that many PQC‐components are secreted in EVs and PQC modulation resulted in a significant increase in EVs numbers, that is paralleled by a slight increase in TDP‐content.

**Summary/Conclusion**: EVs may positively contribute to the clearance of insoluble TDPs species by cooperating with PQC, having a protective role for affected cells. However, they may also contribute to the prion‐like distribution of TDP‐neurotoxic forms in neighboring and more distant cells.

### PS07.02 Biochemical signatures in extracellular vesicles from sporadic Amyotrophic Lateral Sclerosis patients revealed by Raman Spectroscopy and Mass Spectrometry lipidomics

Maria Chiara Mimmi, IRCCS Mondino Foundation Neurological Institute

Daisy Sproviero, PhD, IRCCS Mondino Foundation

Carlo Morasso, Maugeri Scientific Clinical Institutes IRCCS

Fabio Corsi, Maugeri Scientific Clinical Institutes IRCCS

Orietta Pansarasa, Genomic and post‐Genomic Unit, IRCCS Mondino Foundation, Pavia, Italy

Cristina Cereda, Genomic and post‐Genomic Unit, IRCCS Mondino Foundation, Pavia, Italy

**Introduction**: There is no validated blood‐based biomarker for sporadic Amyotrophic Lateral Sclerosis (ALS). Extracellular vesicles (EVs) have the potential to solve this unmet clinical need, as they can be involved in the pathogenesis/progression of neurodegenerative diseases (Selmaj et al., 2017). Lipids are essential molecular components of EVs, but at the moment the knowledge about their distribution and function is limited. The aim of this work was to find biomarkers of ALS by investigating biochemical composition of plasma‐derived EVs with Raman Spectroscopy (RS) and HPLC‐MS (High Performance Liquid Chromatography‐Mass Spectrometry).

**Methods**: We isolated small and large extracellular vesicles (sEVs and lEVs), from blood plasma of 20 sporadic ALS patients and a matched group of healthy controls, by differential centrifugation/ultracentrifugation. We characterized sEVs, lEVs and blood plasma firstly by RS and subsequently by HPLC‐MS, targeting a panel of around 200 lipids. Statistical analysis included univariate and multivariate analysis techniques such as PCA (Principal Component Analysis) and PLS‐DA (Partial Least Squares‐ Determinant Analysis).

**Results**: Raman spectroscopy highlighted lEVs as a particularly promising biomarker for ALS. Raman spectra showed in fact that sporadic ALS patients have a different lipid content and less intense bands relative to the aromatic amino acid phenylalanine. HPLC‐MS revealed some lipid species discriminating between ALS and healthy subjects. They were mainly phospholipids, belonging to the subclasses of phosphatidylcholines (PC), phosphatidylethanolamines (PE) and phosphatidylinositols (PI), and sphingolipids, belonging to the subclasses of ceramides (Cer), mono/di‐hexosyl‐ceramides (M/DHC). In particular the increase of PC(34:1), MHC(24:1) and Cer(24:1) was observed in either plasma, lEVs and sEVs from ALS patients. The species PI(36:3) was up‐regulated in both large and small vesicles of ALS patients.

**Summary/Conclusion**: Interestingly, some species significantly altered in our analysis of plasma lipidome, overlap with the ones highlighted by Blasco et al. in their work on cerebrospinal fluid (CSF) (Blasco et al. 2017), namely PC(38:2), MHC(24:1) and the plasmalogen PCO(34:1). This supports the idea of plasma and plasma‐derived EVs as easily available source of robust biomarkers. Among the other results, the perturbed sphingolipids are particularly relevant as they are involved in key pathways for ALS patogenesis, such as autophagy, energy metabolism and neuroinflammation.

### PS07.04 Plasma extracellular vesicles size and concentration are altered in Alzheimer's disease, dementia with Lewy bodies and frontotemporal dementia

Antonio Longobardi, Molecular Markers Laboratory, IRCCS Istituto Centro San Giovanni di Dio Fatebenefratelli, Brescia, Italy

Luisa Benussi, Molecular Markers Laboratory, IRCCS Istituto Centro San Giovanni di Dio Fatebenefratelli, Brescia, Italy

Roland Nicsanu, Molecular Markers Laboratory, IRCCS Istituto Centro San Giovanni di Dio Fatebenefratelli, Brescia, Italy

Sonia Bellini, Molecular Markers Laboratory, IRCCS Istituto Centro San Giovanni di Dio Fatebenefratelli, Brescia, Italy

Clarissa Ferrari, Service of Statistics, IRCCS Istituto Centro San Giovanni di Dio Fatebenefratelli, Brescia, Italy

Claudia Saraceno, Molecular Markers Laboratory, IRCCS Istituto Centro San Giovanni di Dio Fatebenefratelli, Brescia, Italy

Roberta Zanardini, Molecular Markers Laboratory, IRCCS Istituto Centro San Giovanni di Dio Fatebenefratelli, Brescia, Italy

Marcella Catania, Neurology 5 / Neuropathology Unit, Fondazione IRCCS Istituto Neurologico Carlo Besta, Milan, Italy

Giuseppe Di Fede, Neurology 5 / Neuropathology Unit, Fondazione IRCCS Istituto Neurologico Carlo Besta, Milan, Italy

Rosanna Squitti, Molecular Markers Laboratory, IRCCS Istituto Centro San Giovanni di Dio Fatebenefratelli, Brescia, Italy

Giuliano Binetti, MAC‐Memory Clinic, IRCCS Istituto Centro San Giovanni di Dio Fatebenefratelli, Brescia, Italy

Roberta Ghidoni, Molecular Markers Laboratory, IRCCS Istituto Centro San Giovanni di Dio Fatebenefratelli, Brescia, Italy

**Introduction**: Alzheimer's disease (AD), Lewy body dementia (LBD), frontotemporal dementia (FTD), are the major neurodegenerative dementias. Abnormal protein accumulation characterizes all these diseases. An alteration in extracellular vesicles (EVs) release and or composition might be a common pathological mechanism across dementia influencing the fate of disease‐related proteins. Loss of neurotrophic factors might be one of the determinants affecting EVs release.

**Methods**: EVs were isolated with commercial kit from plasma of n = 30 AD, n = 30 LBD, n = 30 FTD and n = 30 controls (CTRL). Nanoparticle Tracking Analysis (NTA) was performed to evaluate EVs concentration and size distribution. PGRN, BDNF, GDNF and Cystatin C plasma concentrations were measured by Bioplex and ELISA. A classification tree (CT) was applied to detect the best predictors for discriminating CTRL vs patients’ (PTS) group. Patients provided written informed consent. Local ethics committee approval Prot. N. 111/2017.

**Results**: A decrease of plasma EVs concentration and an increase of EVs size were measured in AD, LBD and FTD vs CTRL. Levels of PGRN were reduced in FTD and Cystatin C levels were increased in LBD vs CTRL samples. CT revealed that EVs concentration and size are the best predictors to classify dementia patients from CTRL (96.8%). ROC analysis revealed a good diagnostic performance (AUC = 0.86) of EVs concentration/size ratio. Cystatin C was the only neurotrophic factor associated with EVs concentration.

**Summary/Conclusion**: Alterations in the intercellular communication mediated by EVs might be a common molecular pathway in neurodegenerative dementias. Cystatin C might be one of the determinants affecting EVs release. The identification of shared disease mechanisms is of pivotal importance to develop treatments to delay disease progression.

### PS07.05 EVs size and concentration are altered in frontotemporal dementia caused by progressive loss of progranulin and C9orf72

Sonia Bellini, Molecular Markers Laboratory, IRCCS Istituto Centro San Giovanni di Dio Fatebenefratelli, Brescia, Italy

Luisa Benussi, Molecular Markers Laboratory, IRCCS Istituto Centro San Giovanni di Dio Fatebenefratelli, Brescia, Italy

Claudia Saraceno, Molecular Markers Laboratory, IRCCS Istituto Centro San Giovanni di Dio Fatebenefratelli, Brescia, Italy

Antonio Longobardi, Molecular Markers Laboratory, IRCCS Istituto Centro San Giovanni di Dio Fatebenefratelli, Brescia, Italy

Roland Nicsanu, Molecular Markers Laboratory, IRCCS Istituto Centro San Giovanni di Dio Fatebenefratelli, Brescia, Italy

Roberta Zanardini, Molecular Markers Laboratory, IRCCS Istituto Centro San Giovanni di Dio Fatebenefratelli, Brescia, Italy

Sara Cimini, Neurology 5 / Neuropathology Unit, Fondazione IRCCS Istituto Neurologico Carlo Besta, Milan, Italy

Giacomina Rossi, Neurology 5 / Neuropathology Unit, Fondazione IRCCS Istituto Neurologico Carlo Besta, Milan, Italy

Giuliano Binetti, MAC‐Memory Clinic, IRCCS Istituto Centro San Giovanni di Dio Fatebenefratelli, Brescia, Italy

Roberta Ghidoni, Molecular Markers Laboratory, IRCCS Istituto Centro San Giovanni di Dio Fatebenefratelli, Brescia, Italy

**Introduction**: Frontotemporal dementia (FTD) is a neurodegenerative disease among the most common forms of presenile dementia, mainly affecting individuals under 65 years of age. Mutations in GRN, C9orf72 and MAPT are currently the most common causes of inherited FTD. Cutting‐edge molecular research suggests that lysosomal/exosomal dysfunctions are pathological events driven by the loss of functional proteins in GRN/C9orf72‐associated FTD. We therefore investigated whether extracellular vesicles (EVs) in cells and plasma are altered in association with progressive loss of progranulin and C9orf72.

**Methods**: EVs isolation was performed with a commercial kit from i) human plasma samples: 43 controls, 31 C9orf72 pathological expansion, 9 C9orf72 intermediate expansion, 72 heterozygous GRN null mutation (45 affected and 27 pre‐symptomatics), 3 homozygous GRN null mutation, 4 GRN missense mutation carriers and 10 sporadic FTD; ii) human primary fibroblast conditioned media: 3 controls, 3 C9orf72 pathological expansion, 1 C9orf72 intermediate expansion, 7 GRN null mutation carriers. Size and concentration were measured by Nanoparticle Tracking Analysis (NTA). Patients provided written informed consent. Local ethics committee approval Prot. N.44/2018.

**Results**: In human plasma samples EVs concentration was significantly reduced both in C9orf72 pathologically expanded and GRN+ patients compared to controls, while EVs size significantly increased in the same mutated groups. In human primary fibroblasts we observed a trend toward a progressive decrease of EVs release associated with a progressive loss of progranulin and C9orf72.

**Summary/Conclusion**: EVs dosage and size characterization might be a promising biomarker in GRN/C9orf72‐associated FTD. Taken together these results suggest a correlation between progressive loss of GRN/C9orf72 and lysosomal/exosomal dysfunctions in FTD.

### PS07.06 Levels of neuronal factors in circulating extracellular vesicles predict the progression of preclinical subjects to Alzheimer's disease in APOE ε4 carriers

Mohamed Raâfet Ben Khedher, INRS‐CAFSB

Mohamed Haddad, INRS‐CAFSB

Danielle Laurin, University Laval

Charles Ramassamy, INRS‐Centre Armand‐Frappier Santé‐ Biotechnologie

**Introduction**: Background: In brain, extracellular vesicles (EVs) play an essential role in neuron‐glia interface and ensure the crosstalk between the brain and the periphery. Some studies now link EVs pathway dysfunction to apolipoprotein E4 variant (APOE ε4) and the risk of progression to Alzheimer's disease (AD). To better understand the role of APOE ε4 in pre‐clinical AD, we determined levels of pathogenic, neurotrophic and inflammatory proteins in peripheral EVs (pEVs) and in plasma from cognitively impaired‐no dementia (CIND) participants stratified upon the absence (APOE ε4‐) or the presence (APOE ε4+) of the ε4 allele of APOE.

**Methods**: Method: Levels of 15 neurodegenerative, neurotrophic and neuroinflammatory proteins were quantified in pEVs and compared to their plasma levels from cognitively normal and CIND participants

**Results**: For the first time, several neurotrophic and inflammatory markers including LCN‐2, S100B, ANGPTL‐4, NPTX‐2 and α‐synuclein were evidenced in pEVs. Some proteins such as α‐Syn, NPTX‐2 and S100B were enriched in pEVs as compared to plasma. APOE ε4 presence was associated with differential regulation of 7 markers and compromised the release of pEVs formed by an endosomal route. The pentraxin‐2/α‐synuclein ratio measured in pEVs was able to predict AD. 5 years before the onset, among APOE ε4+ CIND individuals.

**Summary/Conclusion**: Discussion: Our findings suggest an alteration of the endosomal pathway in APOE ε4+ carriers and that pEVs pentraxin‐2/α‐synuclein ratio could serve as a useful early biomarker for AD susceptibility.

### PS07.07 Microglial depletion reduces adeno‐associated virus mediated tau propagation from the entorhinal cortex to the dentate granular cells in human APP NL‐G‐F knock‐in mice while increasing amyloid burden

Kevin A. Clayton, Boston University School of Medicine

Jean‐Christophe Delpech, INRAE

Shawn Herron, Boston University School of Medicine

Seiko Ikezu, Department of Pharmacology & Experimental Therapeutics, Boston University School of Medicine

Tsuneya Ikezu, MD, PhD, Department of Pharmacology & Experimental Therapeutics, Center for Systems Neuroscience,Boston University School of Medicine; Department of Neuroscience, Mayo Clinic Florida

**Introduction**: Microglia are the innate immune cells in the brain, and known to phagocytose apoptotic neurons and dystrophic neurites containing phosphorylated tau (p‐tau), possibly enhancing the spread of pathological tau via extracellular vesicles (EVs). Evidence suggests that proteinopathic stress from amyloid plaques transforms microglia into a neurodegenerative phenotype (MGnD), possessing enhanced phagocytic and exocytotic functions, which may exacerbate propagation of p‐tau in the diseased brain.

**Methods**: C57BL/6 (WT) and APPNL‐G‐F mice were fed with a CSF1R inhibitor (PLX5622) or control chow for one month before and after stereotaxic injections of AAV2/6‐SYN1‐P301Ltau expressing P301L tau mutant into the medial entorhinal cortex (MEC) at 5 months of age. Propagation of tau to the dentate granular cells of the hippocampus, amyloid plaque formation, and association of microglia and p‐tau with plaques were assessed by immunohistochemistry after one month. MGnD and homeostatic microglia were separately isolated from APPNL‐G‐F mouse brains by FACS and evaluated for the expression of EV marker molecules. To investigate the propensity of DAM/MGnD to hyper‐secrete EVs in vivo, we developed a novel lentivirus (pLV‐ mEmerald ‐CD9) to specifically express mEmerald fused to CD9, an exosomal marker, in microglia and co‐injected with AAV‐P301L tau into the brains of diseased and WT mice.

**Results**: APPNL‐G‐F mice exhibited approximately a 10‐fold increase in tau propagation compared to WT mice. Strikingly, PLX5622 treatment, which depleted ∼ 99% of microglia, showed 74 and 87% reduction of tau propagation in WT and APPNL‐G‐F groups respectively. Contrarily, PLX5622 increased intensity of plaque associated p‐tau along with increased size and number of amyloid plaques in the APPNL‐G‐F mice, suggesting their regulation by MGnD microglia. Gene expression of EV markers, CD9 and CD63 was upregulated in Clec7a+ microglia compared to Clec7‐ microglia isolated from APPNL‐G‐F mice, which was further supported by co‐localization of Tsg101, an exosome marker, with Clec7a+ MGnD microglia. The mEmerald‐CD9 lentivirus allowed for visualization of microglia‐specific EVs, which are released by DAM/MGnD at a 3‐fold higher rate than homeostatic microglia and contain pathologic pTau.

**Summary/Conclusion**: Tau propagation may be influenced by engulfment of pathological tau seeds by microglia and secretion through EVs, which is exacerbated in microglia activated by plaques, and mitigated by PLX5622 treatment.

### PS07.08 Extracellular Vesicles: Mediator and Biomarker for Oxidative Stress in Parkinson's Disease

Adityas Purnianto, The Florey Institute of Neuroscience and Mental Health, The University of Melbourne

Leah Beauchamp, The Florey Institute of Neuroscience and Mental Health

Eleanor Saunders, 2.The Department of Biochemistry and Molecular Biology, The University of Melbourne, Parkville, VIC, Australia

Ashley Bush, The Florey Institute of Neuroscience and Mental Health, Parkville, VIC, Australia

David Finkelstein, The Florey Institute of Neuroscience and Mental Health

Kevin Barnham, The Florey Institute of Neuroscience and Mental Health

Laura Vella, The Florey Institute of Neuroscience and Mental Health

**Introduction**: Parkinson's disease (PD) develops insidiously from underlying pathogenesis such as mitochondrial dysfunction and oxidative stress. Increasing evidences in diseases such as cancer and inflammation have demonstrated that oxidative stress induces release of functional extracellular vesicles that contain a source biomarkers of disease characterized by oxidative stress. As a disease characterized by oxidative stress, this phenomenon has been understudied in PD.

**Methods**: We are using cell models of mitochondrial dysfunction and oxidative stress in PD as well as clinical samples (nasal secretion and plasma) from newly diagnosed PD patients to explore the potential role of EVs in oxidative stress in PD. Utilizing different techniques such as western blot and mass spectrometry, we are examining the impact of oxidative stress in the metabolite, metal profiles, and mitochondrial components of EVs content. We are investigating the pathological effects of the intercellular transfer of oxidative‐stress‐modified EVs content, for example by using Seahorse assay, to determine if they contribute to the progression of disease

**Results**: Our initial findings showed that the EV contents of some mitochondrial electron transport chain complexes and metals were altered in EVs from SH‐SY5Y cells treated with rotenone, a mitochondrial complex I inhibitor.

**Summary/Conclusion**: The results of this study will generate knowledge on the biosignature of EVs in early stage PD and the role of EVs in intercellular transfer of oxidative stress to provide insight into the development of EV based biomarkers of prodromal PD.

### PS07.09 Do microbiota‐derived outer membrane vesicles promote inflammation and neurodegeneration in Parkinson's disease?

Tiana F. Koukoulis, The Florey Institute of Neuroscience and Mental Health

Leah Beauchamp, The Florey Institute of Neuroscience and Mental Health

David Finkelstein, The Florey Institute of Neuroscience and Mental Health

Victoria Lawson, Department of Microbiology and Immunology, The University of Melbourne

Neil O'Brien‐Simpson, Centre for Oral Health Research, Melbourne Dental School, University of Melbourne

Maria Kaparakis‐Liaskos, Department of Physiology, Anatomy and Microbiology, La Trobe University

Kevin Barnham, The Florey Institute of Neuroscience and Mental Health

Laura Vella, The Florey Institute of Neuroscience and Mental Health

**Introduction**: The microbiome‐gut‐brain axis plays an important role in Parkinson's disease pathogenesis with dysbiosis of the gut microbiota proposed to initiate a proinflammatory cascade that drives neurodegeneration. The mechanisms by which gut microbes communicate with host cells to trigger inflammation in Parkinson's disease is currently unclear. We hypothesise that microbiota‐derived outer membrane vesicles (OMVs), rich in the potent immune stimulator, lipopolysaccharide (LPS), promote gastrointestinal inflammation and leaky gut, but also the systemic and neural inflammation that characterises Parkinson's disease.

**Methods**: OMVs were isolated from Gram‐negative bacteria in culture or faeces from Parkinson's disease animal models and characterized by density, size and morphology and LPS content. The ability of OMVs to induce a proinflammatory response in vitro was determined and compared to OMVs from WT animals and equivalent concentrations of purified LPS. In vivo studies to determine whether orally administered OMVs predispose the enteric and central nervous system to Parkinson's disease pathology are currently underway.

**Results**: OMVs isolated from Escherichia coli cultures are shown to be more potent at promoting immune activation than an equivalent dosage of LPS in vitro. OMV induced immune activation results in the promotion of neurodegeneration in vitro and anticipated to exacerbate gastrointestinal dysfunction in a neurotoxin mouse model of Parkinson's disease.

**Summary/Conclusion**: We are investigating the functional role of OMVs in Parkinson's disease with OMVs isolated from in vitro culture and the faeces from animal models of the disease. Our preliminary data suggest that OMVs from gram negative bacteria are potent immune stimulators that have the potential trigger and/or exacerbate Parkinson's disease pathogenesis.

### PS07.10 Mesenchymal stem cell‐derived extracellular vesicles ameliorate Alzheimer's disease‐like phenotypes in a 5XFAD mouse model

Allaura S. Cone, Florida State University College of Medicine

Xuegang Yuan, Florida State University College of Engineering

li sun, FSU College of Medicine

Leanne Duke, Florida State University College of Medicine

Michael P. Vreones, Florida State University College of Medicine

Allison N. Carrier, Florida State University College of Medicine

Stephanie M. Kenyon, Florida State University College of Medicine

Spencer R. Carver, Florida State University College of Medicine

Sarah D. Benthem, Florida State University College of Psychology

Alina C. Stimmell,Florida State University College of Psychology

Shawn C. Moseley, Florida State University College of Psychology

Aaron A. WilberFlorida State University College of Psychology

James M. Olcese, Florida State University College of Medicine

David G. Meckes, PhD,FSU college of medicine

**Introduction**: Alzheimer's disease (AD) is an irreversible neurodegenerative disorder that affects more than 44 million people worldwide. Despite the high disease burden, there is no effective treatment for people suffering from AD. Mesenchymal stem cells (MSCs) are multipotent stromal cells that have been widely studied due to their therapeutic potential. However, administration of cells has been found to have a multitude of limitations. Recently, extracellular vesicles (EVs) derived from MSCs have been studied as a therapeutic candidate, as they exhibit similar immunoprotective and immunomodulatory abilities as the host hMSCs.

**Methods**: To test the potential therapeutic effects of MSC EVs, human bone‐marrow derived MSCs were grown in 3D cell culture, and small EVs were harvested using differential centrifugation and PEG precipitation. These small EVs were given to non‐transgenic (NT) or 5XFAD (5 familial Alzheimer's disease mutations) mice intranasally (IN) every 4 days for 4 months. The mice were then required to perform a variety of behavioral assays to measure changes in learning and memory. Afterwards, immunohistochemistry was performed on brain slices to measure amyloid beta (Aβ) and glial fibrillary acidic protein (GFAP) levels.

**Results**: The data revealed that 5XFAD mice that received EV treatment behaved significantly better in cognitive tests than saline treated 5XFAD mice, with no significant change between EV‐treated 5XFAD mice and NT mice. Additionally, we found lower Aβ plaque load in the hippocampus of the EV‐treated mice. Finally, less colocalization between GFAP and Aβ plaques was found in the brain of EV‐treated mice compared to saline.

**Summary/Conclusion**: Taken together, these data suggest that IN administration of MSC‐derived EVs can slow down AD pathogenesis in a preclinical mouse model.

### PS07.11 A Parkinson's disease‐causing LRRK2 mutation leads to an early disruption of brain exosome biogenesis

Lital Rachmany, Department of Psychiatry, New York University School of Medicine, New York, NY 10016, USA

Brainson Liemisa, Center for Dementia Research, Nathan S. Kline Institute, Orangeburg, New York 10962, USA

Samantha F. Newbury, Center for Dementia Research, Nathan S. Kline Institute, Orangeburg, New York 10962, USA

Adaora Aroh, Center for Dementia Research, Nathan S. Kline Institute, Orangeburg, New York 10962, USA

Efrat Levy, Center for Dementia Research, Nathan S. Kline Institute, Orangeburg, New York 10962, USA

Paul M. M. Mathews, Center for Dementia Research, Nathan S. Kline Institute, Orangeburg, New York 10962, USA

**Introduction**: Parkinson's disease is a neurodegenerative disorder clinically characterized by motor and cognitive deficiencies with the loss of dopaminergic neurons in the substantia nigra pars compacta and the presence of alpha synuclein aggregates. Variants of the Leucine‐Rich Repeat Kinase 2 (LRRK2) are associated with an increased risk of Parkinson's disease, with the Gly2019Ser mutation a relatively common cause of familial Parkinson's disease. Knockin mice carrying this mutation develop motor symptoms with aging and cell death in cortical neurons, mediated in part by altered macroautophagy. At a pre‐symptomatic age in these mice, we investigated the effect of this mutation on brain exosome biology, a key component of the endosomal‐lysosomal system.

**Methods**: Six‐month‐old mutant LRRK2 mice and wildtype controls were compared. Motor skills were studied using RotaRod and wire hanging tests. Extracellular vesicles (EVs) were isolated from brain tissue using a density‐base column and analyzed by nanoparticle tracking and electron microscopy. Specific markers for exosomes as well as alpha‐synuclein levels were determined using Western blot analysis.

**Results**: The six‐month‐old mice were pre‐symptomatic for Parkinsonian motor deficiencies. Lower levels of exosomes were seen in the brains of LRRK2 mice as compared to controls. While no difference in the level of alpha‐synuclein was found in brain homogenates, alpha‐synuclein levels were significantly lower in EVs in the LRRK2 mice compared to controls.

**Summary/Conclusion**: Our findings argue that brain exosome biogenesis is compromised by the LRRK2 mutation prior to the development of clinical symptoms in a mouse model. Additionally, our data show that alpha‐synuclein in EVs is reduced. While additional studies are needed, an alteration in exosomal alpha‐synuclein packaging may prove to be a useful biomarker of early Parkinson changes in the endosomal‐lysosomal system and reflect pathological alterations in alpha‐synuclein within neurons.

## PS08 EVs in Regenerative Medicine

Chair: Qing‐Ling Fu, The First Affiliated Hospital, Sun Yat‐sen University, China (People's Republic)

Chair: Sai Kiang Lim, Institute of Medical Biology, Agency for Science, Technology and Research, Singapore. Department of Surgery, Yong Loo Lin School of Medicine, National University of Singapore, Singapore

### PS08.01 Optimization of culture conditions for the production of mesenchymal stromal cell‐derived extracellular vesicles towards its translation into large‐scale manufacturing

Raquel MS Cunha, Instituto Superior Técnico, University of Lisbon

Cecília Calado, ISEL ‐ Instituto Superior de Engenharia de Lisboa

Joaquim M.S. Cabral, Department of Bioengineering and Institute for Bioengineering and Biosciences, Instituto Superior Técnico, Universidade de Lisboa

Cláudia Lobato da Silva, Department of Bioengineering and Institute for Bioengineering and Biosciences, Instituto Superior Técnico, Universidade de Lisboa

Ana Fernandes‐Platzgummer, Department of Bioengineering and Institute for Bioengineering and Biosciences, Instituto Superior Técnico, Universidade de Lisboa

**Introduction**: Therapies based on mesenchymal stromal cells‐derived extracellular vesicles (MSC‐EV) have emerged as a potential alternative to whole cell therapies, as MSC‐EV offer specific advantages for patient safety such as the minimal predisposition to activate innate and immune responses. However, MSC‐EV productivity and efficacy are still limited, and clinical translation entails scalable and GMP‐compliant processes for their production, isolation and characterization.

**Methods**: To optimize MSC‐EV production, we compared different MSC tissue sources (bone marrow, adipose tissue, umbilical cord matrix) and culture conditions (feeding regime, oxygen tension, temperature, chemical cues). MSC isolated from multiple donors were expanded using Serum‐free and Xeno‐Free culture medium under static conditions. The conditioned medium (CM) was collected at different time points and MSC‐EV were isolated by ultracentrifugation or with a commercially available isolation kit and characterized according to ISEV guidelines.

**Results**: MSC derived from the different sources and donors were able to grow under the different culture conditions tested, while maintaining their immunophenotype and differentiation potential, according to the minimal criteria defined by the ISCT. The feeding regime was optimized and the best time point for pre‐conditioning and collection of CM for MSC‐EV isolation was determined. To further optimize MSC‐EV production, different physical (oxygen tension, temperature) and chemical cues were tested to determine the conditions that resulted in higher EV production using techniques as NTA, protein and lipid quantification and purity assessment. Additional MSC‐EV characterization techniques included western blot, flow cytometry, imaging, FTIR and omic tools (using the cells, CM and culture medium as controls, according to ISEV guidelines).

**Summary/Conclusion**: In summary, this study contributes to the establishment of optimal culture conditions for MSC‐EV production using different MSC tissue sources. The optimized culture conditions are being translated into scalable processes for MSC‐EV production using bioreactor systems.

### PS08.02 Immortalization strategies for human mesenchymal stromal cells for large scale production of extracellular vesicles

Yanis Mouloud, Universitätsklinikum Essen

**Introduction**: Mesenchymal stromal cells (MSCs) are considered as therapeutic agent for many diseases due to their immunomodulatory properties. Apparently, secreted extracellular vesicles (EVs) that MSCs also release in vitro mediate these activities. Indeed, we have successfully confirmed the therapeutic potential of EVs prepared from conditioned media of cultured MSCs in several animal models and a treatment resistant GvHD patient. Thus, MSC‐EVs provide a promising therapeutic agent for the future.

Currently, we aim to scale the MSC‐EV production process for the clinical setting. However, the scaling process is limited by the life span of EV releasing cells.

**Methods**: To address that issue, we have compared different strategies to immortalize primary MSCs for the production of immunomodulatory EVs.

**Results**: Indeed, we were able to establish immortalized clonal MSC lines which maintained their bona fide MSC features and secrete immunomodulatory active EVs.

**Summary/Conclusion**: To learn whether the immortalization affects the quality of released EVs, the immune modulatory capabilities of secreted EVs were analysed in a mixed lymphocyte reaction assay. EVs isolated from immortalized MSC supernatants retained their ability to modulate immune responses in the MLR assay just like EVs harvested from supernatants of the original primary MSCs. EVs produced by these clonal cell lines will now broadly be tested in various disease models. Importantly, batch‐to‐batch variations will be addressed.

### PS08.03 Regenerative capacity of blood‐derived EVs on primary osteoarthritic chondrocytes coincides with the EV‐associated miRNA functional repertoire

Alexander Otahal, Danube University Krems

Karina Kramer, Danube University Krems

Olga Kuten‐Pella, Orthosera GmbH

Christoph Stotter, Danube University Krems

Markus Neubauer, Danube University Krems

Zsombor Lacza, Orthosera GmbH

Stefan Nehrer, Danube University Krems

Andrea De Luna, Danube University Krems

**Introduction**: Regenerative medicine increasingly focuses on blood‐derived products for osteoarthritis therapy. Frequently, citrate‐anti‐coagulated platelet‐rich plasma (CPRP) is intra‐articularly injected into diseased joints, however, cell‐free alternatives such as hyperacute serum (hypACT) are under development. Mechanisms of action of blood products are still poorly understood. The discovery of EVs in blood and EV‐associated cargo molecules such as miRNAs opened up new levels of complexity in understanding the therapeutic potential of blood products.

**Methods**: To investigate the role of EVs isolated from CPRP and hypACT during osteoarthritis (OA),primary human OA chondrocytes were treated with EVs enriched via ultracentrifugation (UC) from these two blood products. Chondroprotective and anti‐inflammatory effects were evaluated based on gene expression analysis via reverse transcription quantitative PCR (RT‐qPCR) and Western Blot, as well as cytokine release via ELISA, respectively. EV‐associated miRNA profiles were analysed by screening a 372 miRNA panel via RT‐qPCR in EVs purified via UC and size exclusion chromatography (SEC) as well as in the respective blood products.

**Results**: EVs from either blood product increased the expression of anabolic markers type II collagen (COL2A1), SRY‐box transcription factor 9 (SOX9) and aggrecan (ACAN) compared to blood products, but also the catabolic marker and tissue remodeling factor matrix metalloproteinase 3 (MMP3). CPRP blood product increased SOX9 protein expression, in contrast, CPRP EVs decreased NFκB and COX2 expression in IL1β‐stimulated chondrocytes compared to unstimulated cells. However, hypACT EVs induced SOX9 protein expression while preventing IL6 secretion compared to hypACT blood product. Analysis of the functional repertoire encoded in EV‐associated miRNAs revealed that CPRP EV‐associated miRNAs strongly target NFκB signaling and hypACT EV‐associated miRNAs were predicted to strongly affect IL6‐ and TGFβ/SMAD signaling.

**Summary/Conclusion**: The results indicate that blood EVs are sufficient to induce chondrogenic gene expression changes in OA chondrocytes, while preventing pro‐inflammatory cytokine release compared to full blood products. In addition, the effects observed in the biological assays can be explained by EV‐associated miRNAs. This highlights the potential of blood‐derived EVs to be regulators of cartilage extracellular matrix metabolism and inflammation as well as candidates for new cell‐free therapeutic approaches for OA.

### PS08.04 Cardiac progenitor cell‐derived EVs affect human macrophage polarization

Margarida Viola, Laboratory of Experimental Cardiology, Circulatory Health Laboratory, UMC Utrecht Regenerative Medicine Center, University Medical Center Utrecht, Utrecht University, The Netherlands

Pieter Vader, CDL Research, University Medical Center Utrecht, The Netherlands

Saskia C.A. de Jager, Laboratory of Experimental Cardiology, Circulatory Health Laboratory, Center for Translational Immunology, UMC Utrecht Regenerative Medicine Center, University Medical Center Utrecht, Utrecht University, The Netherlands

Joost P.G. Sluijter, J.P.G., Department of Experimental Cardiology, University Medical Center Utrecht, Utrecht University, The Netherlands

**Introduction**: Regeneration of damaged heart tissue upon myocardial infarction remains a major challenge. Transplantation of cardiac progenitor cells (CPCs) has been studied as a potential regenerative therapy. Interestingly, recent studies have shown that the cardioprotective effect of CPCs is mediated by the release of extracellular vesicles (EVs). The benefits of CPC‐EVs have mostly been associated with stimulation of angiogenesis and inhibition of cell death. Although macrophages have been suggested to be key for cardiac repair, the effect of CPC‐EVs on macrophages is poorly explored.

**Methods**: EVs were isolated from serum‐starved CPCs by ultrafiltration followed by size exclusion chromatography. Human monocytes were isolated from the blood of healthy donors and differentiated into macrophages with M‐CSF. In addition, macrophages were stimulated with LPS + IFNy or with IL4 in order to induce an inflammatory M1 and reparative M2 phenotype, respectively. The macrophages were subsequently stimulated with CPC‐EVs and analyzed by flow cytometry, bulk RNA sequencing and confocal microscopy to assess macrophage phenotype changes.

**Results**: Stimulation of macrophages with CPC‐EVs enhances the expression of pro‐inflammatory markers, including CD80, while decreasing anti‐inflammatory markers, such as CD200R and CD206. In line with these findings, CPC‐EV‐stimulated macrophages adopt a morphology that reflects the inflammatory M1 macrophage. Ongoing bulk sequencing on these conditions will provide in‐depth insight on phenotype changes in these cells.

**Summary/Conclusion**: Our data suggest that CPC‐EVs are able to induce macrophage polarization towards an inflammatory phenotype, which might have implications for CPC‐EV treatment after myocardial infarction. This underlines an urgent need to understand the molecular mechanisms underlying the immunomodulatory effect of CPC‐EVs before moving into a clinical setting.

### PS08.05 Human cardiac mesenchymal stromal cell‐derived extracellular vesicles are protective of ischemia‐reperfusion injury

Max M. Chen, MS, Graduate Institute of Biomedical Materials & Tissue Engineering, Taipei Medical University, Taiwan

Andreas Czosseck, MSc, Graduate Institute of Biomedical Materials & Tissue Engineering, Taipei Medical University, Taiwan

Chuan‐Chih Hsu, MD, Department of Surgery, Taipei Medical University Hospital, Taiwan

Annette Meeson, PhD, Biosciences Institute, Newcastle University, UK

Thomashire A. George, MbChB, International PhD Program in Biomedical Engineering, Taipei Medical University, Taiwan

Chen‐Lin Chen, School of Biomedical Engineering, Taipei Medical University

David J. Lundy, PhD, Graduate Institute of Biomedical Materials & Tissue Engineering, Taipei Medical University, Taiwan

**Introduction**: During myocardial infarction, cardiomyocytes (CMs) die by apoptosis in response to ischemia, leading to permanent cardiac remodeling and loss of heart function. Nanometer scale particles such as EVs are amenable to delivery by intracoronary infusion and show greater uptake and retention than live cells and a longer duration of activity than isolated growth factors and cytokines. Furthermore, their complex cargo is able to activate multiple survival pathways, preventing apoptosis and promoting endogenous regeneration. Here, we explore whether EVs from a novel population of human cardiac stromal cells (CMSCs) may protect CMs from ischemia‐reperfusion injury (IRI).

**Methods**: CMSCs were derived from human heart tissues and maintained under MSC conditions with 10% exosome‐depleted FBS (Gibco A2720801). Ethical approval was obtained from TMU‐JIRB (N201910027). Surface markers were characterised by flow cytometry. Bone marrow‐derived MSCs (BM‐MSCs, Lonza) were used as a control. Extracellular particles were collected by ultracentrifugation (100,000g, 16h, 4˚C) of conditioned culture media and characterised by NTA, TEM and Western Blot (WB). H9C2 rat cardiomyoblasts, as a model for CMs, were maintained in DMEM‐HG, 10% FBS, 5% CO2. To induce ischemic injury, cells were subjected to 48h hypoxia (BD AnaeroPack), 0% FBS. To mimic reperfusion injury, cells were restored to normoxia with fresh culture media ± CMSC‐EVs. CCK‐8 and flow cytometry (Annexin‐V/PI) were used to measure H9C2 viability, apoptosis and necrosis.

**Results**: CMSCs were found to express typical MSC markers, such as CD44, CD90, CD166 and CD105, and were negative for CD19, CD45 and CD11b. RT‐qPCR analysis revealed that these cells express pro‐regenerative, anti‐apoptotic growth factors (VEGFA, IGF‐1, FGF2, ANGPT1 etc.) at levels equal or greater than BM‐MSCs. NTA revealed particles 160.1 ± 2.0 nm diameter at significantly higher yield than BM‐MSCs. TEM showed characteristic particles with diameters 100–200 nm. WB was positive for Hsp70, CD9, CD63 and CD81.

In IRI rescue experiments, CMSC‐EVs protected H9C2 cells from 48h ischemic injury, showing CCK‐8 absorbance at 144% compared to 10% FBS (100%) and 0% FBS (‐71%). Following reperfusion injury, CMSC‐EV treated cells had viability of 76%, compared to 27.7% for 0% FBS and 53.7% for 10% FBS. Flow cytometry showed that the percentage of apoptotic cells significantly decreased from 26.2% (0% FBS) or 14.8% (10% FBS) to 10.5% (CMSC‐EVs).

**Summary/Conclusion**: These results clearly demonstrate the therapeutic potential of CMSC‐EVs. Further work is underway to characterise their precise cargo of CMSC‐EVs, quantify their uptake, and to examine rescue pathway induction in the target cells.

### PS08.06 Impact of mesenchymal stromal/stem cell isolation strategies on the immunomodulatory activity of their extracellular vesicles

Oumaima Stambouli, Institute for Transfusion Medicine

Robin Dittrich, Institute for Transfusion Medicine, University Hospital Essen, Germany

Fabiola Nardi Bauer, Institute for Transfusion Medicine, University Hospital Essen, Germany

Tobias Tertel, Institute for Transfusion Medicine, University Hospital Essen, Germany

Peter A. Horn, Institute for Transfusion Medicine, University Hospital Essen, Germany

Bernd Giebel, Prof, Institute for Transfusion Medicine, University Hospital Essen, Germany

**Introduction**: Mesenchymal stromal cell (MSC) derived extracellular vesicles (EVs) are increasingly considered as therapeutic agents. In our hands, only a proportion of obtained MSC‐EV preparations revealed immunomodulatory activities, both, in murine acute Graft‐versus‐Host Disease and in a multi‐donor mixed lymphocyte reaction assay (mdMLR). According to our understanding, variations in the MSC‐EV preparations’ activities are due to the heterogeneity of their parental MSCs. Here, we aimed to study impacts of the initial growth conditions on human bone marrow (BM)‐derived MSCs and their resulting EV products.

**Methods**: MSCs were either raised from BM aspirates or from mononuclear cells (MNCs) harvested thereof. BM aspirates and MNCs were seeded into DMEM low media supplemented with human platelet lysate (hPL) as well as in EBM media supplemented with cytokines and hPL. Non‐adherent cells were removed after 24h and cultures continued in hPL supplemented DMEM low. Growth rates, cell surface phenotypes and the osteogenic and adipogenic differentiation capabilities of obtained MSCs were analyzed. Starting from passage 1, conditioned media (CMs) were harvested every 48 hours and stored at ‐20°C until processing. After thawing, EVs were prepared from CMs applying the PEG‐ultracentrifugation method. Obtained samples were characterized according to the MISEV criteria and by imaging flow cytometry. Their immunomodulatory activities were investigated in the mdMLR assay.

**Results**: The initial culture conditions affect the growth rates and phenotypes of MSCs. First results imply, aspirate‐derived MSCs grow slower, reach senescence quicker and expressed less CD59 on their EVs than those derived from MNC‐derived MSCs. MSCs initially raised in EGM revealed higher expansion capacities than MSCs directly raised in DMEM low. Although, all MSC‐EV preparations harvested from early passage CMs revealed immunomodulatory activities, they showed different impacts on macrophages, which we are currently dissecting.

**Summary/Conclusion**: Our preliminary data imply initial seeding strategies impact the characteristics of obtained MSCs and the immunomodulatory activity of their EV products. However, more experiments need to be performed to obtain robust data.

### PS08.07 Electrospun scaffolds as novel platforms for sustained release of extracellular vesicles for cell‐free tissue engineering

Hatim Alqurashi, 1‐ University of Sheffield 2‐ King Faisal University

Stuart Hunt, School of Clinical Dentistry, University of Sheffield, Sheffield, UK

Ilida Ortega Asencio, School of Clinical Dentistry, University of Sheffield, Sheffield, UK

Daniel Lambert, School of Clinical Dentistry, University of Sheffield, Sheffield, UK

**Introduction**: Extracellular vesicles (EVs) are membrane‐enclosed vesicles that are secreted by cells and mediate cell"cell communication via their protein, lipid, carbohydrate, and nucleic acid (RNA, DNA) cargo. EVs are involved in a multitude of physiological processes including development, cell differentiation and angiogenesis having also been associated with tissue repair. Thus, they have been suggested to offer opportunities for the development of novel cell‐free tissue engineering (TE) approaches.

**Methods**: Method: EVs were isolated from the conditioned media of cells in culture using ultracentrifugation (UC) and size exclusion chromatography (SEC). After characterising the size and protein abundance of EVs, they were incorporated into polycaprolactone (PCL) electrospun scaffolds. EV‐modified scaffolds were characterized using SEM, TEM and fluorescence microscopy. The release kinetics of EVs from scaffolds was examined using nanoparticle‐tracking analysis (NTA). The influence of myofibroblast‐derived EVs on cell growth and migration was studied using scratch assays.

**Results**: Result: EVs were successfully isolated by UC and SEC as assessed by NTA and determination of presence of EV marker proteins (CD9, CD63 and CD81). Following different methods of functionalization of PCL scaffold, EVs were well‐incorporated and distributed within scaffolds. NTA showed slow release of 40% of EVs from the scaffolds over 21 days. Myofibroblast‐derived EVs (myo‐EV) significantly increased fibroblast cell growth and migration compared to TGFbeta 1‐treated cells and untreated controls.

**Summary/Conclusion**: Conclusion: Here we provide evidence that electrospun scaffolds can be functionalised with EVs and provide sustained slow release, offering an opportunity to develop novel, cell‐free and tuneable approaches to tissue engineering. We also showed that myo‐EV may have a role in wound healing, and that incorporation of myo‐EVs in electrospun scaffolds may have potential as a regenerative medicine approach.

### PS08.08 Mesenchymal Stromal Cell‐derived Extracellular Vesicles restore alteration of endoplasmic reticulum stress genes in Human Corneal Endothelial Cells

Lola Buono, University of Turin, Department of Molecular Biotechnology and Health Sciences, Italy

Raffaele Nuzzi, S.C.U. Ophthalmology Unit, “City of Health and Science”

Marco De Iuliis, S.C.U. Ophthalmology Unit, “City of Health and Science”

Simona Scalabrin, S.C.U. Ophthalmology Unit, “City of Health and Science”

Benedetta Bussolati, University of Turin, Department of Molecular Biotechnology and Health Sciences, Italy

**Introduction**: Human Corneal Endothelial Cells (HCECs) are essential to visual function, however, since they have limited proliferative capacity in vivo, they are prone to corneal endothelial dysfunction. The aim of this work was to evaluate whether EVs derived from Mesenchymal Stromal Cells (MSC‐EVs) were able to promote regeneration of HCECs after exposing them to endoplasmic reticulum stress (ER‐stress) and the mechanisms involved.

**Methods**: We isolated HCECs from discarded corneas in patients undergoing corneal transplantation (n = 23 patients). Human bone marrow MSCs were obtained from Lonza, cultured and characterized. MSC‐EVs were obtained from supernatants of MSCs. We evaluated the proliferation rate, apoptosis and migration of HCECs after exposure to nutrient deprivation or Tunicamycin. We then evaluated the regulation of ER‐stress‐related genes, such as CHOP, ATF4, EIF2a, BIM, XBP1, BCLXL in presence or in absence of MSC‐EVs and compared their effect with a different source of EVs, derived from patient blood serum (SER‐EV).

**Results**: In the selected damage conditions, the treatment with different doses of MSC‐EVs resulted in a significantly higher proliferation rate of HCECs at all the tested concentrations of EVs and in the decrease of total apoptotic cells. Moreover, MSC‐EVs induced a faster repair of the wound after 24 hours of serum‐deprivation. At molecular level, we observed an upregulation of the ER stress‐related genes ATF4, CHOP, BIM, XBP1 during HCECs damage, and a significant down‐regulation of their expression after the treatment with MSC‐EVs in all the genes tested. SER‐EVs were able to down‐regulate significantly the expression of ATF4, but not the other genes studied.

**Summary/Conclusion**: Our results highlight the well‐known pro‐regenerative potential of MSC‐EVs and bring to light their ability to restore HCECs from ER‐stress induced by nutrient deprivation.

### PS08.09 Epigenetic reprogramming enhances the therapeutic efficacy of osteoblast‐derived extracellular vesicles for bone regeneration

Kenny Man, University Of Birmingham

Mathieu Brunet, School of Chemical Engineering, University of Birmingham, Birmingham, United Kingdom.

Maria Fernandez‐Rhodes, School of Sport, Exercise and Health Sciences, Loughborough University, Loughborough, United Kingdom.

Soraya Williams, School of Sport, Exercise and Health Sciences, Loughborough University, Loughborough, United Kingdom.

Owen Davies, School of Sport, Exercise and Health Sciences, Loughborough University, Loughborough, United Kingdom.

Angelica Federici, Trinity Centre for Biomedical Engineering, Trinity Biomedical Sciences Institute, Trinity College Dublin, Ireland.

David Hoey, Trinity Centre for Biomedical Engineering, Trinity Biomedical Sciences Institute, Trinity College Dublin, Ireland.

Sophie C. Cox, School of Chemical Engineering, University of Birmingham, Birmingham, United Kingdom.

**Introduction**: For bone regeneration, there is great precedence to develop acellular technologies that circumvent limitations associated with cell‐based therapies. Extracellular vesicles (EVs) have the potential to stimulate stem cell mineralisation due to their diverse cargo offerings compared to single growth factor treatments. Regulating the cell's epigenetic function through histone deacetylase (HDAC) inhibition increases their differentiation potential. Therefore, we investigated altering osteoblasts epigenome via the HDAC inhibitor Trichostatin A (TSA) on promoting osteoblast‐derived EVs potency.

**Methods**: TSA effect on osteoblast epigenetic functionality and mineralisation was determined by quantifying HDAC activity and calcium deposition. EVs were isolated from untreated/TSA treated osteoblasts for 2 weeks. EV size and concentration were defined using nanoparticle tracking analysis and transmission electron microscopy. EVs microRNA expression was evaluated using microarray analysis. Osteogenic differentiation of human bone marrow stromal cells (hBMSCs) cultured with untreated (MO‐EVs)/TSA treated osteoblast‐derived EVs (TSA‐EVs) was evaluated by qPCR, biochemistry and histological analysis.

**Results**: TSA significantly reduced osteoblast HDAC activity and enhanced calcium deposition when compared to untreated cells. The quantity of EVs generated, in addition to their protein content and size correlated with the degree of osteoblast differentiation. TSA‐EVs accelerated hBMSCs migration and proliferation compared to MO‐EVs. Importantly, TSA‐EV treatment significantly upregulated hBMSCs osteoblast‐related gene/protein expression (ALP, Col1a, OCN) and promoted extracellular matrix mineralisation when compared to MO‐EVs. Microarray analysis revealed TSA‐EV enriched microRNAs were involved in regulating mechanisms such as “endocytosis” and “Wnt signalling pathway”.

**Summary/Conclusion**: Taken together, these findings demonstrate the considerable utility epigenetic reprogramming provides a novel engineering approach to enhance EVs therapeutic efficacy for bone regeneration.

### PS08.10 Extracellular vesicles derived from induced pluripotent stem cells in hypoxic conditions enhance angiogenesis

André Cronemberger Andrade, Paracelsus Medical University

Martin Wolf, Cell Therapy Institute, Spinal Cord Injury and Tissue Regeneration Center Salzburg (SCI‐TReCS), Paracelsus Medical University (PMU), Salzburg, Austria

Fausto Gueths Gomes, Cell Therapy Institute, Spinal Cord Injury and Tissue Regeneration Center Salzburg

Patricia Ebner, Cell Therapy Institute, Spinal Cord Injury and Tissue Regeneration Center Salzburg (SCI‐TReCS), Paracelsus Medical University (PMU), Salzburg, Austria

Heidi Marie Binder, Cell Therapy Institute, Spinal Cord Injury and Tissue Regeneration Center Salzburg (SCI‐TReCS), Paracelsus Medical University (PMU), Salzburg, Austria

Katarina Schallmoser, Department of Transfusion Medicine and SCI‐TReCS, PMU, Salzburg, Austria

Strunk Dirk, Cell Therapy Institute, Spinal Cord Injury and Tissue Regeneration Center Salzburg (SCI‐TReCS), Paracelsus Medical University (PMU), Salzburg, Austria

**Introduction**: Stem cells secrete paracrine factors including EVs that are important in cellular communication and can support the regeneration of injured tissues. Reduced oxygen conditions (hypoxia) are crucial for proliferation and self‐renewal of stem cells. As hypoxia is a key regulator in development and regeneration it may also be an important factor influencing cellular communication via EVs. Therefore we investigated whether hypoxic pre‐conditioning can impact iPSC‐EV quantity and/or quality (phenotype and cargo) & thus have an impact for EV‐based therapy.

**Methods**: We produced iPSC EVs using tangencial fow filtration (TFF) from iPSC conditioned media from diferent oxygen levels conditions. The EVs were quantified by Tunable Resistive Pulse Sensing (TRPS). The protein content was quantified and analyzed by immunoblotting for EV markers. In addition to check if the cells were effetely under hypoxia conditions using a hypoxia marker (pimonidazole) we also checked the HIF‐1α expression by immunoblotting. The viability of the cells cultivated under different oxygen levels conditions were checked staining with 7AAD and Annexin V. The functionality of the EVs derived from different oxygen conditions were checked by Angioassay.

**Results**: The method using TFF to concentrate the condition media is a good strategy to produce a large amount of EVs with good recovery. A higher pimonidazole positive signal was found in the cells under 1% oxygen compared to the cells on 5% and 18% oxygen conditions. HIF‐1α was incresed in 1% oxygen condition compared with 5%, 18% and the hypoxia mimetic chemical CoCl2 (positive control). In all conditions we didn't find any loss on viability.The EV derived from 1% oxygen increased the tube like formation compared with the other vesicles and also the soluble factors.

**Summary/Conclusion**: The EVs derived from iPSC preconditioned in hypoxia condition showed a enhance angiogenesis pontecial. This strategy could be an important tool in the production of particularly potent and targeted EV‐based therapeutics.

### PS08.11 The immune‐regulatory properties of extracellular vesicles derived from human mesenchymal stem/stromal cells

Dimitrios Tsiapalis, School of Pharmacy and Pharmaceutical Sciences & Trinity Biomedical Sciences Institute, Trinity College Dublin, Dublin, Ireland

Tobias Tertel, Institute for Transfusion Medicine, University Hospital Essen, Germany

Achilleas Floudas, Molecular Rheumatology, Trinity College Dublin, Ireland

Ursula Fearon, Molecular Rheumatology, Trinity College Dublin, Ireland

Bernd Giebel, Prof, Institute for Transfusion Medicine, University Hospital Essen, Germany

Verena Börger, Institute for Transfusion Medicine, University Hospital Essen, University of Duisburg Essen, Essen, Germany

Lorraine O'Driscoll, School of Pharmacy and Pharmaceutical Sciences, Trinity Biomedical Sciences Institute, Trinity St. James's Cancer Institute & TRAIN‐EV Marie Skłodowska‐Curie Action‐Innovative Training Network, train‐ev.eu

**Introduction**: Extracellular vesicles (EVs) from human bone‐marrow mesenchymal stem/stromal cells (BM‐MSCs) are of much interest because they may exert therapeutic effects, such as modulating immune response at a site of injury. However, a reliable potency assay for evaluating immune‐modulatory effects of EVs has yet to be established. Towards achieving this, here we isolated and characterised EVs from MSCs cultured under different conditions and assessed their immuno‐regulatory properties, in term of influence on inflammatory cytokines release from CD4+ T‐cells derived from peripheral blood mononuclear cells (PMBCs) of rheumatoid arthritis (RA) patients.

**Methods**: BM‐MSCs were cultured in T175‐flasks in media supplemented with either human platelet lysate (hPL) or EV‐depleted FBS. After 48h, conditioned media was collected. EVs were isolated using PEG precipitation and characterised using nanoparticle tracking analysis (NTA), transmission electron microscopy (TEM), immunoblotting, and flow‐imaging (Amnis ImageStream). Flow cytometry (LSRFortessa) analysed a panel of cytokines released from CD4+ T‐cells exposed to the MSC‐EVs.

**Results**: NTA indicated significantly more EVs/particles released when MSCs were cultured with hPL‐supplemented medium versus medium with EV‐depleted FBS. TEM revealed spherical‐shaped EVs, with both cultures. Immunoblots showed the presence of EV‐associated proteins CD9, CD63, CD81, CD105 and Syntenin‐1 and the absence of Calnexin. Supporting these findings, flow‐imaging showed increased numbers of CD9+, CD63+ and CD81+ positive sEVs when MSCs were cultured in hPL‐medium. Preliminary data demonstrated that the MSC‐EVs modulate (typically suppressing) the release of four cytokines that are associated with RA pathogenesis, i.e. TNFα, IL‐22, GM‐CSF and IL‐2.

**Summary/Conclusion**: These findings highlight the immuno‐regulatory potential of MSC‐EVs. Further studies validating immune‐modulatory effects of MSC‐EVs on CD4+ T‐cells are underway.

### PS08.12 Comparative analysis of extracellular vesicles (EVs) derived from adipose tissue‐ and bone marrow‐ derived mesenchymal stromal cells

Cansu Gorgun, University of Genova

Maria Elisabetta Federica Palamà, Department of Experimental Medicine (DIMES), University of Genoa, Italy

Daniele Reverberi, U.O. Molecular Pathology, IRCCS Policlinico San Martino, Genoa, Italy

Roberta Tasso, University of Genova

Chiara Gentili, Department of Experimental Medicine (DIMES), University of Genoa, Italy

**Introduction**: Mesenchymal stromal cells (MSCs) isolated from different tissue origins already present variations in their secretory profile. Since EVs from different origin contain diverse contents and exert different functions, some studies have come up with the comparison of exosomes from adipose tissue‐ and bone marrow derived MSCs.Yet, these studies were limited for considering only one type of EV subpopulation. Considering whether an EV‐dependent function is specific to a given EV subtype, in this study, we carried out a detailed and comparative characterization of middle‐sized and small‐sized EVs released by both adipose‐tissue (AD)‐ and bone marrow(BM)‐MSCs to investigate their involvement as modulators of MSC paracrine effects.

**Methods**: EVs have been separated from the conditioned media of AD‐ and BM‐MSCs by serial differential centrifugations in order to collect both medium‐sized and small‐sized EVs. EVs were characterized by transmission electron microscopy, tunable resistive pulse sensing and western blot. The expression of the tetraspanin family members was evaluated using a non‐conventional flow cytometry approach. The role of both medium and small‐sized EVs derived from AD and BM in the different phases of the endochondral ossification process has been evaluated by ex vivo mouse metatarsal culture model that allows for the study of both vessel sprouting and linear bone growth.

**Results**: Although EVs derived from adipose tissue‐ and bone marrow derived MSCs present similar characteristics in terms of size, concentration and marker expression, they possess significant differences in their protein content that are reflected in their functional effects. The comparison of proteins with a wide range protein array showed that EVs derived AD‐MSCs contained pro‐ angiogenic factors in comparison to the BM counterpart. As a consequence, they were able to induce a significant increase in vessel sprouting. On the other hand, EVs derived from the bone marrow contained a higher amount of pro‐differentiation and chemotactic proteins in comparison to the adipose tissue MSC‐EVs, and they were able to prompt growth plate morphology. Interestingly, during these comparisons of functional effects, even though that same concentration of EVs applied, small sized EVs had a significant effect in comparison with their counterpart middle‐sized EVs.

**Summary/Conclusion**: In conclusion, this study highlights the importance of selecting the appropriate MSC‐source for EV‐ based therapies for targeted therapeutic applications. Indeed, in the context of endochondral ossification, EVs from both ADSCs and BMSCs have diverse functional effects. Considered these EV properties of the EVs mirroring the parental cells, further study of the synergistic effect of both MSC‐ derived EVs would be great interest.

### PS08.13 Chitin‐based polysaccharides enhance the immunomodulating potential of human mesenchymal stem cell‐derived extracellular vesicles

Eun Seo Kim, Seoul National University

Katsuhiko Kida, Nissan Chemical Corporation

Jongsoo Mok, Seoul National University

Yeonwoo Seong, Seoul National University

Seo Yeon Jo, Seoul National University

Tatsuro Kanaki, Nissan Chemical Corporation

Masato Horikawa, Planning and Development Division

Kyung‐Hee Kim, National Cancer Center

Tae Min Kim, Seoul National University

Tae Sub Park,Seoul National University

Joonghoon Park, Seoul National University

**Introduction**: Mesenchymal stem cell (MSC) transplantation is a promising treatment in regenerative medicine. However, MSCs grown in two‐dimensional (2D) culture conditions vary significantly in cell shape from in vivo, with downregulated stemness genes and secretion of paracrine factors.

**Methods**: Here, we evaluated the effect of three‐dimensional (3D) culture with chitin‐based, water‐insoluble polysaccharides on the characteristics of human Wharton's jelly‐derived MSCs (hMSCs).

**Results**: After 3D culture of hMSCs with the chitin‐based polysaccharides, the retrieved cells showed significantly improved cell proliferation rate compared with the conventional 2D counterpart. Transcriptome analyses indicated that 3D culture enhanced gene expression involved in cell stemness, migration ability, and extracellular vesicle (EV) production. Subsequent biochemical studies showed that expression levels of OCT4, NANOG, and SSEA4 were upregulated and migration ability was increased in hMSCs grown under 3D culture conditions. In addition, EV production was significantly increased in 3D cells compared to 2D cells, and EVs from 3D cells showed to have a differentiated protein profile compared to 2D EVs. Analyses of gene and drug connectivity revealed that 2D and 3D EVs had similar functions as immunomodulators. However, 3D EVs had entirely different therapeutic profiles based on activation of the disease‐associated signaling pathways for different infectious and metabolic diseases. Transcriptome responses after cell treatment revealed that fenbendazole, an immunomodulator, formed the same cluster as 3D EVs, and 2D EVs belonged to the same cluster as vehicle control. Therefore, through the similarity analysis of the global transcriptome response, it was confirmed that 3D EVs induces a mostly similar pharmacological response to fenbendazole.

**Summary/Conclusion**: The pharmacological mechanisms and predicted applications based on Connectivity map analyses supported the high therapeutic potential of 3D EVs for certain conditions. Therefore, EVs from 3D cultured hMSCs using chitin‐based polysaccharides can be applied as novel therapeutic agents for treatment of immune and metabolic diseases.

### PS08.14 Investigating the functionality of miRNAs in Wharton's Jelly‐derived small extracellular vesicles (sEV) and their potential role in neuro‐regeneration

Vera Tscherrig, Department for BioMedical Research (DBMR), Graduate School for Cellular and Biomedical Sciences (GCB), and Department of Obstetrics and Feto‐Maternal Medicine, Inselspital

Sophie Cottagnoud, Department of Obstetrics and Feto‐maternal Medicine, University Women's Hospital, Inselspital, Bern University Hospital, Bern, Switzerland

Valérie Haesler, Department of Obstetrics and Feto‐maternal Medicine, University Women's Hospital, Inselspital, Bern University Hospital, Bern, Switzerland

Patricia Renz, Department of Obstetrics and Feto‐maternal Medicine, University Women's Hospital, Inselspital, Bern University Hospital, Bern, SwitzerlandGraduate School for Cellular and Biomedical Sciences (GCB), University of Bern, Bern, Switzerland, and

Daniel Surbek, Department of Obstetrics and Feto‐maternal Medicine, University Women's Hospital, Inselspital, Bern University Hospital, Bern, Switzerland

Andreina Schoeberlein, Department of Obstetrics and Feto‐maternal Medicine, University Women's Hospital, Inselspital, Bern University Hospital, Bern, Switzerland

Marianne Jörger‐Messerli, Department of Obstetrics and Feto‐maternal Medicine, University Women's Hospital, Inselspital, Bern University Hospital, Bern, Switzerland

**Introduction**: Perinatal white matter injury (WMI) is one of the most common neurological complications of preterm birth and it is a global health problem resulting in long‐term neurodevelopmental and neurobehavioral disabilities. Until now there is no cure for perinatal WMI. Recently, our lab and others have shown promising results towards the use of mesenchymal stromal cell derived small extracellular vesicles (MSC‐sEV) as a therapeutic approach for neuronal injuries. It is known that sEV secreted by MSC carry small non‐coding RNA such as microRNAs (miRNAs). MicroRNAs might interfere with signaling pathways involved in premature WMI. Thus, we hypothesize that miRNAs, released by sEV upon uptake in their target cells, have a key function in the observed beneficial effects of MSC‐sEV.

**Methods**: MSC were isolated from the connective tissue of human umbilical cords, the so‐called Wharton's jelly. sEV were purified from the cells using ultracentrifugation, followed by size‐exclusion chromatography (SEC). The fractions were characterized according to the expression of sEV markers using western blot analysis and miRNAs by quantitative PCR.

**Results**: The SEC fractions with the highest protein content also showed positive signals for the sEV markers CD81 and CD63. No cellular contamination has been observed (no signal for GM130 or Grp94). These sEV fractions contained high amounts of miRNAs, such as miRNA 22–5p, miRNA 27b‐3p or let7b‐5p.

**Summary/Conclusion**: The highly abundant miRNAs in the sEV fractions target specific apoptotic or inflammatory pathways and drive oligodendrocyte differentiation. Therefore, these miRNAs might influence WMI outcomes. For better understanding of this hypothesis, the sEV fractions, containing the most abundant miRNAs, are currently tested for their functionality using dual luciferase assays.

### PS08.15 Matrix‐bound vesicles within extracellular matrix in the regulation of mesenchymal stem cells differentiation in vitro

Ekaterina Novoseletskaya, Institute for regenerative medicine, Medical research and education center, Lomonosov Moscow State University

Daria Selina, Faculty of Biology, Lomonosov Moscow State University

Nataliya Basalova, Institute for regenerative medicine, Medical Research and Education Center, Lomonosov Moscow State University, Moscow, Russia

Yana Danilova, Faculty of Biology, Lomonosov Moscow State University

Olga Sokolova, Faculty of Biology, Lomonosov Moscow State University

Anastasia Efimenko, Institute for regenerative medicine, Medical Research and Education Center, Lomonosov Moscow State University, Moscow, Russia

**Introduction**: Extracellular matrix (ECM) regulates various cell functions, including stem cell differentiation, but little is known about the impact of matrix‐bound vesicles (MBVs) into these effects. Mesen‐chymal stem/stromal cells (MSCs) are critical contributors of tissue renewal and repair pro‐cesses due to the secretion of paracrine substances that regulate the homeostasis of tissue‐specific stem cell niches. We evaluated how MBVs within the native ECM produced by MSC could affect the behavior of stem cells.

**Methods**: ECM was obtained from cell sheets of immortalized MSCs by decellularization (dECM) using CHAPS and DNase type I treatment, and vesicle‐like structures in dECM were observed using scanning electron microscopy (SEM) and confirmed by transmission electron microscopy (TEM). We compared 3 approaches to isolate MBVs by treating dECM with 1) collagenase I type, 2) collagenase I type and hyaluronidase, 3) trypsin. Isolated MBVs were visualized by TEM and characterized for the presence of key exosome markers by immunoblotting. Then we explored the effects of MBVs on human adipose‐derived MSC trilineage differentiation (adipo‐genic, osteogenic or chondrogenic) by the addition of isolated MBVs or their removal from dECM by phospholipase, isopropanol, or RNase treatment.

**Results**: We confirmed that MBVs were comprised of MSC‐produced ECM and could be effectively iso‐lated or removed from dECM by all tested protocols. No significant effect on MSC differentia‐tion was detected after the treatment of dECM with RNase, while isopropanol treatment led to the slight decrease of MSC osteogenic and chondrogenic differentiation and isolated MBVs stimulated MSC adipogenic differentiation.

**Summary/Conclusion**: We assume that MSC‐produced ECM includes MBVs which could regulate the functional activity of stem cells interacted with ECM. Further research is needed to explore if MBVs in MSC‐produced ECM represent a specific class of extracellular vesicles.

## PS09 Engineering and Loading EVs

Chair: Charles Lai, Institute of Atomic and Molecular Sciences, Academia Sinica, Taiwan (Republic of China)

Chair: Xabier Osteikoetxea, Head of H‐CEMM Extracellular Vesicle Lab, Department of Genetics, Cell‐ and Immunobiology, Semmelweis University, Hungary

### PS09.01 3D‐Printed Gelatin Methacrylate Hydrogel for Sustained Release of MSC EVs

Louis J. Born, University of Maryland

Shannon McLoughlin, University of Maryland

Bhushan Mahadik, University of Maryland

John Fisher, University of Maryland, College Park

Steven M. Jay, University of Maryland, College Park

**Introduction**: Complex wounds, resulting from disease, trauma, or surgical intervention, remain a major source of morbidity; thus, continued effort to develop new wound therapies is necessary. Mesenchymal stem/stromal cell (MSC) extracellular vesicles (EVs) are one avenue of investigation that have shown a therapeutic benefit in wound healing, particularly by inducing angiogenesis. However, rapid clearance of EVs from the body after a single, naked administration is one limitation hindering clinical translation. We hypothesize that a customizable gelatin methacrylate hydrogel using 3D‐printing will allow for the sustained release of MSC EVs within a therapeutic window.

**Methods**: Gelatin methacrylate (GelMa) hydrogels will be 3D‐printed as both homogenous and 3D‐concentric discs using MSC EV‐loaded GelMa as a bioink. Crosslinking density will be varied based on amount of LAP photoinitiator added to the GelMa solution, and ultraviolet radiation will be used to crosslink the gelatin methacylate. Hydrogels will be placed in PBS, and PBS will be collected and replenished periodically over time. EVs will be concentrated using 300kDa nanoseps and resuspended in equal volumes of PBS. EVs released will be measured using BCA and an ELISA for exosomal marker CD63. An endothelial scratch assay will be used to test for retained bioactivity of EVs.

**Results**: MSC EVs were successfully loaded into GelMa, 3D printed into discs, and crosslinked by UV irradiation. Initial release studies of a homogenous disc resembled a burst release profile using BCA and CD63 ELISA. EVs released from the discs within a 24h period showed retained bioactivity in a human umbilical cord endothelial cell (HUVEC) scratch assay. HUVECs treated with GelMa‐released EVs in basal media had increased gap closure compared to basal media alone (p < 0.05).

**Summary/Conclusion**: Release of bioactive MSC EVs from 3D‐printed GelMa is achievable and utilizing such a printing method may allow for the tailoring of release profiles for optimized wound healing.

### PS09.02 Extracellular vesicles from Red blood cells Modified with gp350 protein deliver therapeutic cargo for Targeting Therapy of Burkitt lymphoma

Huiqing Xiu, Zhejiang University School of Medicine

**Introduction**: The feasibility of extracellular vesicles (EVs) as a drug carrier has been verified countlessly. For clinical treatment, the source of EVs is an important issue to be considered. At present, most of the sources of EVs in literature are cell lines, but cells secrete a small amount of EVs, and the EVs from which they originate may carry carcinogenic DNA and other harmful substances. A potential ideal source of EVs for therapeutic use may be human red blood cells.: 1) Red blood cells lack of nuclear DNA and mitochondrial DNA; 2) Red blood cells are the most abundant cell type in the body; 3) Red blood cells can be easily obtained from the human body and have been used safely and routinely for decades For blood transfusion. In addition, another problem with drug‐loaded treatment of EVs is its targeting. Most of the EVs that have not been modified will accumulate in the liver tissue. At present, most of the modification methods are by overexpression of the plasmid into the cell line, collecting the supernatant after the overexpression of the plasmid, and extracting EVs. However, this method is time‐consuming and labor‐intensive, and is not suitable for primary cells or cells that are difficult to overexpress. Therefore, we need to explore new ways to modify EVs.

**Methods**: 1. Purify gp350 protein with transmembrane segment using prokaryotic system.

2. Extract human blood, separate red blood cells, incubate overnight with calcium ionophore, and extract EVs derived from red blood cells after gradient high ionization and ultraionization.

3. Red blood cells‐derived EVs are mixed with doxorubicin and electroporated to load the drug.

4. The EVs derived from red blood cells are incubated with the purified gp350 protein with transmembrane segments and electroporated at low voltage.

**Results**: 1. Gp350‐modified 293T‐derived EVs can target CD21 positive tumor cells in vitro

2. The gp350 protein was successfully loaded into the red blood cell‐derived EVs membrane by low voltage electroporation

3. Gp350‐modified red blood cell‐derived EVs can target CD21 positive tumor cells, loaded with chemotherapeutic drugs have increased cytotoxicity in CD21+ tumor

4. Gp350‐modified red blood cell‐derived EVs loaded with chemotherapeutic drugs have high biosafety

**Summary/Conclusion**: First, we found that gp350 modified EVs can effectively target CD21 positive tumor cells. We purified the gp350 protein and successfully loaded it into the red blood cell‐derived EVs membrane by co‐incubation and low‐voltage electroporation (termed RBC‐EVs/gp350), which effectively targeted CD21 positive tumors. The RBC‐EVs/gp350 loaded with chemotherapeutic drug showed high toxicity to CD21 positive tumors and have high biosafety. In conclusion, we developed a new method for modifying extracellular vesicles.

### PS09.03 Hybrid nanoconstructs for cancer therapy based on zinc oxide nanocrystals shielded by extracellular vesicles

Bianca Dumontel, Department of Applied Science & Technology, Politecnico di Torino

Francesca Susa, Department of Applied Science & Technology, Politecnico di Torino

Tania Limongi, Department of Applied Science & Technology, Politecnico di Torino

Luisa Racca, Department of Applied Science & Technology, Politecnico di Torino

Nadia Garino, Department of Applied Science and Technology, Politecnico di Torino

Doriana Debellis, Electron Microscopy Facility, Fondazione Istituto Italiano di Tecnologia Genova

Roberto Marotta, Electron Microscopy Facility, Fondazione Istituto Italiano di Tecnologia Genova

Valentina Cauda, Department of Applied Science & Technology, Politecnico di Torino

**Introduction**: Thanks to peculiar properties connected with their biological origin and function, extracellular vesicles (EVs) are emerging as promising tools in drug delivery. This study focuses on the encapsulation of zinc oxide nanocrystals (ZnO NCs) in cell‐derived EVs for the creation of a hybrid nanoconstruct for the treatment of cancer cells, which will combine the cytotoxic potential of ZnO nanostructures with the stability and biomimicry imparted by EVs‐shielding.

**Methods**: ZnO NCs synthesized by a microwave‐assisted synthesis were combined with EVs extracted form cell culture supernatants of B‐lymphocytes through a differential ultracentrifugation protocol. The encapsulation was obtained through an active loading method, based on the application of freeze‐thaw cycles followed by co‐incubation steps. The nanoconstructs were characterized by fluorescence microscopy, transmission electron microscopy, nanoparticle tracking analysis and flow‐cytometry.

**Results**: The encapsulation of ZnO NCs in EVs was successfully achieved, obtaining hybrid nanoconstructs characterized by promising loading efficiency. The optimization of the number of applied freeze‐thaw cycles and of the duration of the subsequent incubation steps allowed the production of loaded‐EVs characterized by a well‐preserved morphology and surface protein expression. The greater colloidal stability in biological media of the hybrid nanoconstructs with respect to pristine ZnO was also assessed.

**Summary/Conclusion**: In this study, we show the successful encapsulation of ZnO NCs in cell‐derived EVs for the creation of a novel therapeutic nanoconstruct. The EVs‐shielding efficiently stabilizes the ZnO NCs in biological environment and would also improve their biocompatibility and capability to interact with cells, proposing a strategy for the development of more reliable and effective tools for the treatment of cancer cells.

### PS09.04 In vitro homing and targeting capabilities characterization of native and engineered lymphocytes‐derived extracellular vesicles

Francesca Susa, Department of Applied Science & Technology, Politecnico di Torino

Tania Limongi, Department of Applied Science & Technology, Politecnico di Torino

Bianca Dumontel, Department of Applied Science & Technology, Politecnico di Torino

Luisa Racca, Department of Applied Science & Technology, Politecnico di Torino

Valentina Cauda, Department of Applied Science & Technology, Politecnico di Torino

**Introduction**: In this study the homing and targeting capabilities of native (nEVs) and engineered (eEVs) extracellular vesicles (EVs) were investigated to develop new therapeutic/theranostic strategies. The targeting efficiency towards lymphocytes, myeloid (HL60) and lymphoid (Daudi) cell lines was evaluated by anti‐CD20 engineering the EVs.

**Methods**: Lymphocytes‐derived EVs were isolated through a differential ultracentrifugation protocol and characterized by nanoparticle tracking analysis, bradford assay. and flow cytometry (FC) analysis. The cytotoxic effect and the internalization capabilities of both nEVs and eEVs were tested after 24 and 48 hours treatments using WST‐1 assay, FC and fluorescence microscopy (FM).

**Results**: Results evidenced that EVs isolated from healthy cells are fully tolerated and characterized by having an exceptional tropism towards the parental cell line and the two tested cancer cell lines. FC and FM showed a significantly higher internalization of nEVs in lymphocytes and Daudi cells than in HL60, while, by anti‐CD20 engineering EVs, it was possible to successfully tune EVs tropism towards the target Daudi cells.

**Summary/Conclusion**: In this study the innate tropism of nEVs and the active heterologous targeting of eEVs were compared and eEVs showed a significant ability to selectively target the Daudi cancer cell line.

These EVs showed a high potential for the design of a new artificial or hybrid vesicular systems for therapeutic or theranostic applications for a broad category of diseases.

### PS09.05 Isolation of Extracellular Vesicles Based on Cyclic Tangential Flow Filtration system

Kimin Kim, University of Brain Education

Jungjae Park, Korea Advanced Institute of Science and Technology

Jik‐Han jung, Korea Advanced Institute of Science and Technology

Ruri Lee, University of Brain Education

Ji‐Ho Park, Korea Advanced Institute of Science and Technology

Jong Min Yuk, Korea Advanced Institute of Science and Technology

Hyundoo Hwang, BBB Inc.

Ju Hun Yeon, University of Brain Education

**Introduction**: Conventional isolation for extracellular vesicles (EVs) including size‐based filtration techniques have several limitations in that large particles generally not only reduce the number of open pores of the membrane, but also rupture cells by hydrodynamic resistance. However, Tangential flow filtration (TFF) systems have emerged as a commercially viable approach to flow along the feed stream to the side of the membrane and allow the flow to continue to circulate for preventing clogging problems.

**Methods**: We propose a double cyclic tangential flow filtration (dcTFF) system consisting two modules with different membrane pore sizes, in this case 30–200 nm, for size‐exclusion‐based exosomes isolation. Exosomes are continuously moved along the tubing by peristaltic pump for size‐exclusion‐based exosome isolation. We compared the isolation efficiency of dcTFF chip with direct filtration and single"cyclic TFF (scTFF) chip.

**Results**: We confirmed that the cyclic TFF system allows simultaneous separation of EVs with specific 30–200 nm size range corresponding to upper and lower membranes and shows higher isolation efficiency compared with direct filtration (DF) and single cyclic TFF (scTFF). We found that dTFF system sorted heterogenous EV population from cell culture media into different‐sized EV subpopulations at a particular filter size in the appropriate outlet. Furthermore, this system used for buffer‐exchange and concentration together by eliminating soluble protein, without interference from large vesicles, in one step. Finally, we compared the EVs isolated by dcTFF and ExoQuick technique. The EVs separated by dcTFF chip had more abundant EV marker proteins.

**Summary/Conclusion**: The cyclic TFF system not only has great potential to improve isolation efficiency in terms of recovery rate and EV yield but can be used in clinical applications, including therapeutics, diagnostic biomarkers. Accordingly, EV‐based diagnostic technique can help provide information on treatment efficacy in real‐time for optimal therapeutic responses.

### PS09.06 Photoporation for loading of extracellular vesicles with exogenous molecules: a platform technology to study their cell interaction specificity

Jana Ramon, Laboratory of General Biochemistry and Physical Pharmacy, Faculty of Pharmaceutical Sciences, Ghent University, Ghent, Belgium

Stephan Stremersch, Laboratory of General Biochemistry and Physical Pharmacy, Faculty of Pharmaceutical Sciences, Ghent University, Ghent, Belgium

Koen Raemdonck, Laboratory of General Biochemistry and Physical Pharmacy, Faculty of Pharmaceutical Sciences, Ghent University, Ghent, Belgium

Kevin Braeckmans, Laboratory of General Biochemistry and Physical Pharmacy, Faculty of Pharmaceutical Sciences, Ghent University, Ghent, Belgium

**Introduction**: Evidence is growing that EVs are important players in cell‐to‐cell communication. Moreover, it is believed that EVs have a unique surface fingerprint that steers their cell tropism. However, it remains a challenge to study this type of intercellular communication in an in vivo mimicking context. Ideally, transfer of cargo‐loaded EVs from one cell to another is monitored while those cells are embedded in a physiologically relevant environment. We therefore propose photoporation as a pre‐formation EV loading method that has the ability to target cells in complex co‐cultures in a spatial‐selective manner. This technique uses photothermal nanoparticles and pulsed laser light to create transient pores in the cell membrane through which cargo molecules are able to passively diffuse directly into the cell's cytosol. As it is extensively reported that EVs obtain their cargo from the parent cell's cytoplasm, we hypothesize that the loaded compounds are subsequently encapsulated in EVs during their formation.

**Methods**: Spontaneous encapsulation of fluorescent dextrans into EVs was compared with fluorescently labeled nanobodies that target GFP‐labeled EVs (active targeting). Those molecules were delivered into the cytosol of in vitro‐cultured HEK293t cells using photoporation. Subsequently, after 48h EVs were purified from conditioned medium and EV loading efficiency was assessed with a spinning disk microscope. Furthermore, uptake of cargo‐loaded EVs by recipient cells was evaluated by means of co‐culture experiments.

**Results**: We demonstrated that a fraction of the delivered cargo was spontaneously passed from the parent cell's cytoplasm into the formed EVs confirming that EVs indeed obtain their cargo from the cytosol. Furthermore, we were able to track the loaded EVs after uptake by recipient cells in a co‐culture experiment. More specifically, the fraction of recipient cells that had taken up nanobody‐loaded EVs was twice as high compared to dextran‐loaded EVs indicating an improved EV labeling efficiency with the active targeting approach.

**Summary/Conclusion**: Altogether these results hint toward the potential of photoporation for EV loading, which could facilitate the study of EV behavior under physiologically relevant conditions and paves the way for future clinical applications of EV‐based drug carriers.

### PS09.07 Scalable bioreactor production and angiogenic potential of extracellular vesicles derived from human mesenchymal stromal cells

Miguel Almeida Fuzeta, Department of Bioengineering and iBB – Institute for Bioengineering and Biosciences, Instituto Superior Técnico, Universidade de Lisboa, Lisboa, Portugal

Nuno Bernardes, Department of Bioengineering and Institute for Bioengineering and Biosciences, Instituto Superior Técnico, Universidade de Lisboa

Simonides Immanuel van de van de Wakker, Department of Experimental Cardiology, University Medical Center Utrecht, Utrecht University, The Netherlands

Marieke T. T. Roefs, Department of Experimental Cardiology, University Medical Center Utrecht, Utrecht University, The Netherlands

Wilte Olijve, Department of Experimental Cardiology, University Medical Center Utrecht, Utrecht University, The Netherlands

Ana Fernandes‐Platzgummer, Department of Bioengineering and Institute for Bioengineering and Biosciences, Instituto Superior Técnico, Universidade de Lisboa

Pieter Vader, CDL Research, University Medical Center Utrecht, The Netherlands

Joost P.G. Sluijter, J.P.G., Department of Experimental Cardiology, University Medical Center Utrecht, Utrecht University, The Netherlands

Joaquim M.S. Cabral, Department of Bioengineering and Institute for Bioengineering and Biosciences, Instituto Superior Técnico, Universidade de Lisboa

Cláudia Lobato da Silva,Department of Bioengineering and Institute for Bioengineering and Biosciences, Instituto Superior Técnico, Universidade de Lisboa

**Introduction**: Mesenchymal stromal cells (MSC) hold great promise for cell‐based therapies due to their inherent immunomodulatory and trophic activities. Increasing evidence suggests that several MSC‐associated therapeutic features are mediated by extracellular vesicles (EVs). Despite the promising potential of EVs for therapeutic applications, robust manufacturing processes that would increase the consistency and scalability of EV production are still lacking.

**Methods**: In this work, EVs were produced by MSC isolated from different human tissues (bone marrow (BM), adipose tissue and umbilical cord matrix (UCM)). A serum‐/xeno‐free microcarrier‐based culture system was implemented in a Vertical‐Wheel™ bioreactor (VWBR) towards the scalable production of MSC‐derived EVs (MSC‐EVs). EVs were isolated using a commercial precipitation kit and compared with EVs obtained from static culture systems (i.e. T‐flasks).

Furthermore, a 3D in vitro assay using endothelial spheroids was applied to study the angiogenic potential of MSC‐EVs obtained from different tissues (BM and UCM). For this purpose, MSC‐EVs were isolated from static cultures through size exclusion chromatography.

**Results**: The VWBR system allowed the production of EVs at higher EV number per volume of conditioned medium (5.7‐fold increase) and higher number of EVs generated per cell (3‐fold increase), compared to T‐flasks.

Both BM and UCM MSC‐EVs (from static cultures) significantly enhanced sprouting in an endothelial spheroid assay, increasing the total sprouting length per spheroid by 92 ± 13 % and 89 ± 9 % compared to controls, respectively, after 24 h of treatment.

**Summary/Conclusion**: A scalable bioreactor culture system was implemented, allowing a substantial improvement in the production of MSC‐EVs. MSC‐EVs obtained from different human tissues showed the ability to stimulate angiogenesis in a 3D in vitro assay. Further studies using such assays will allow to elucidate the angiogenic potential of MSC‐EVs obtained from scalable bioreactor cultures.

### PS09.08 SPION decorated neutrophil‐derived exosomes for targeted cancer therapy

jiahui zhang, jiangsu university

xu zhang, jiangsu university

**Introduction**: Neutrophils are the most abundant white cells in human circulation, which acted as an important player in tumor development and progression.

**Methods**: Neutrophils were isolated from peripheral blood by using polymorphprep (Axis‐Shield Po CAS, Norway), and the study was approved by the ethics committee of Jiangsu University (2014280).

**Results**: 1. Isolation, characterization and uptake of exosomes derived from Neutrophils

2.N‐Ex inhibited cell proliferation and promoted cell apoptosis by activating caspase signaling pathway in tumor

3.N‐Ex displayed tumor suppressive activity in vivo

4.The magnetic separation, characterization and cellular uptake of SPION‐Ex/MF

5.In vitro cytotoxicity and inhibition of the SPION‐Ex/MF to human gastric cancer cells

**Summary/Conclusion**: Our results suggest that SPION‐Ex under the external magnetic field displayed great potential in membrane targeting for cancer therapy.

### PS09.09 The dynamic of lipidic vesicles membrane: processing and testing of engineered cell‐derived Exosomes

Eugenia Romano, Department of Chemical, Materials Engineering & Industrial Production, University of Naples Federico II; Interdisciplinary Research Center on Biomaterials, CRIB; Center for Advanced Biomaterials for Health Care, CABHC; Istituto Italiano di Tecnologia

Enza Torino, Department of Chemical, Materials Engineering & Industrial Production, University of Naples Federico II; Interdisciplinary Research Center on Biomaterials, CRIB, Center for Advanced Biomaterials for Health Care, CABHC; Istituto Italiano di Tecnologia

**Introduction**: In recent decades, endogenous nanocarrier‐exosomes (Exos) have received considerable scientific interest as drug delivery system. Exos are cell‐derived particles that present various advantages over traditional delivery vehicles. The unique proteo‐lipid architecture allows the crossing of various natural barriers and protects Exos cargo from degradation in the bloodstream. However, the presence of this membrane as well as their endogenous content make loading of exogenous molecules challenging. Indeed, Exos therapeutic applications have been limited due to a lack of efficient drug loading methods. At date several approaches are involved, nonetheless there is no consensus on which technique results in more advantages for this purpose: aggregation, fusion of Exos themselves, overestimation and low encapsulation efficiency are some of the main drawbacks related to traditional methods.

With the present work we will investigate how to promote the manipulation of vesicles curvature by microfluidics system as ground‐breaking method for Exos encapsulation. The rationale behind this whole new encapsulation approach is to exploit physical gradients acting on vesicles to induce the formation of transient pores or even the deformation of Exos without disrupting them, thus allowing inward diffusion of cargos from the surrounding media.

**Methods**: Exos isolation from U87‐MG brain tumoral cell culture media was performed through Ultracentrifugation. Once validated all the exosomal properties by particle size, morphology and total protein content, we applied a novel method for the encapsulation in the lipidic bilayer of a chemotherapeutic agent. For high drug‐loading efficiency, a series of ratios were assessed. Then, encapsulation efficacy was calculated through UV‐VIS analysis. Finally, we performed in vitro preliminary test to validate the nanobiointeraction of Exos, uptake mechanisms and cytotoxic effect in a 3D cell culture model.

**Results**: U87‐MG derived Exos showed structural and biological stability before and after the encapsulation with the chemotherapeutic agent. Furthermore, in vitro preliminary test demonstrated an increased cytotoxic effect of engineered cell‐derived Exos compared to free drug.

**Summary/Conclusion**: In our work we validated a novel active encapsulation method for extracellular vesicles. Considering the “homing feature”, we tested brain tumoral cell derived Exos in their biological environment. We analyzed Exos uptake mechanisms and demonstrated an increased toxic effect of such a natural nanoplatform for drug delivery in a cancer cell model.

### PS09.10 Xenogenization of tumor cells by fusogenic exosomes in tumor microenvironment ignites and propagates antitumor immunity

Kim Gibeom, Korea Institute of Science and Technology

Gihoon Nam, Dana Farber Cancer Institute

Insan Kim, Korea Institute of Science and Technology

**Introduction**: Immune checkpoint blockades have revolutionized the treatment of patients with several types of malignancies, but only a subset of patients responds to these therapies. To increase the response rates to current immunotherapies, researchers have sought to modify tumor cells in order to render them more immunogenic. The term “xenogenization” refers to the use of pathogenic antigen increase the likelihood that a cancer cell will be recognized as non‐self, foreign by the host immune system. This xenogenization of tumor cells increases their antigenicity, and the generated danger signals activate dendritic cells to induce cross‐presentation of tumor antigens to CD8+ T cells. Here, we demonstrate a new exosome based approach for increasing the immunogenicity of tumors via xenogenization of tumor cells, by eliciting a robust anti‐tumor immune response. Exosomes, which are membrane vesicles that shuttle genetic information and proteins between both neighboring and distant cells, are positioned to become a widespread tool for drug delivery.

**Methods**: Here, we engineered a fusogenic exosome to introduce viral antigens onto cancer cell membranes. We used a mutant form of VSV‐G; that efficiently promotes membrane fusion at the tumor extracellular pH. The fusogenic exosome enables viral PAMPs to be efficiently transferred into the cancer cell membrane.

**Results**: The xenogenized tumor cells by the fusogenic exosome, readily recognized and engulfed by DCs, thereby eliciting the anti‐cancer immune responses. Combination therapy with anti‐PD‐L1 antibodies yielded potent tumor‐specific immune responses. Together, our findings provide a rationale for the therapeutic application of exosome‐based tumor xenogenization to enhance tumor immunogenicity and induce potent anti‐cancer immunity.

**Summary/Conclusion**: There are also still challenges pertaining to the efficiency of protein delivery by fusogenic exosomes. The heterogeneity of exosomes makes it difficult to quantify and control the dose of PAMP proteins needed to enhance phagocytosis. Nevertheless, our present results indicate that exosome‐induced xenogenization appears to offer a new approach for improving the therapeutic efficacy of immune checkpoint blockade in cancer patients. The proposed approach is expected to represent a powerful tool for understanding the direct communication between tumor immunogenicity and the cancer susceptibility to the host's immune responses.

### PS09.11 Encapsulating Cas9 into extracellular vesicles by protein myristoylation

Chenming Ye, University of Georgia

Joseph Whitley, University of Georgia

Houjian Cai, University of Georgia

**Introduction**: The CRISPR/Cas9 system is poised as a promising technology for treatment of human diseases. Delivery of Cas9/sgRNA ribonucleoprotein complexes (RNPs) is considered as the most reliable approach since it has a shorter life in targeted cells, which minimizes the potential off‐target effects. However, one of the major obstacles is how to deliver the RNPs in vivo for clinical applications.

Protein N‐myristoylation is a co/post‐translational modification that leads to the covalent attachment of the myristoyl group (14‐carbon saturated fatty acyl) to the N‐terminus of a target protein. The myristoylation of Src kinase is essential for its cellular localization, intracellular trafficking, subsequently its kinase activity. In this study, we intend to explore if protein myristoylation can be utilized for encapsulation of Cas9 into extracellular vesicles (EVs) for delivery of RNPs into recipient cells.

**Methods**: 1) Myristoylated proteins in EVs were extracted and analyzed in several EVs database. 2) The optimal length of peptides was identified using docking analysis. 3) Myristoylation of octapeptides was measured by an NMT1 activity assay. 4) Src levels and multiple protein markers in the EVs and cell lysate were analyzed by immunoblotting. 5) EVs were characterized by ZataView and transmission electron microscope. 6) Encapsulation of Cas9 in EVs was evaluated by proteinase K, and genomic editing function was examined by T7 endonuclease assay.

**Results**: We demonstrate that myristoylated proteins were preferentially encapsulated into EVs. Myristoylation promoted Src kinase to be encapsulated into EVs, and an optimal octapeptide which is a favorable substrate for N‐myristoyltransferase 1 was identified. Importantly, the octapeptide served as an EVs‐encapsulation tag to pack Cas9 protein into EVs when fused to its N‐terminus genetically. The modified Cas9 underwent myristoylation and maintained its genome editing function when targeting GFP and luciferase genes. The gain of myristoylation facilitated the encapsulation of Cas9 along with sgRNA into EVs.

**Summary/Conclusion**: Genetic engineering Cas9 by fusing a leading sequence of Src kinase promotes encapsulation of Cas9/sgRNA into EVs. This strategy may open an effective avenue to utilize EVs as a vehicle to deliver RNPs for genome editing in recipient cells.

### PS09.12 Post production modified murine mesenchymal stem cells (mMSC) derived EV: interesting bioinspired nanocarriers for biomolecules intracellular delivery?

Sarah LE SAUX, ICGM, Univ. Montpellier, CNRS, ENSCM, Montpellier, France

Anne AUBERT, ICGM, Univ. Montpellier, CNRS, ENSCM, Montpellier, France

Hanna Aarrass, ICGM, Univ. Montpellier, CNRS, ENSCM, Montpellier, France

Khaled Elhady MOHAMED, CBS, Univ Montpellier, INSERM, CNRS, Montpellier, France

Joséphine LAI‐KEE‐HIM, CBS, Univ Montpellier, INSERM, CNRS, Montpellier, France

Patrick Bron, CBS, Univ Montpellier, INSERM, CNRS, Montpellier, France

Jean ARMENGAUD, Laboratory « Innovative technologies for Detection and Diagnostics », CEA‐Marcoule, DRF/JOLIOT/DMTS/SPI/Li2D, Bagnols sur‐ Cèze, France

Guylaine Miotelo, Laboratory « Innovative technologies for Detection and Diagnostics », CEA‐Marcoule, DRF/JOLIOT/DMTS/SPI/Li2D, Bagnols sur‐ Cèze, France

Simon GEORGE, MGX‐Montpellier GenomiX, IGF, CNRS, INSERM, Univ Montpellier, Montpellier

Emeric DUBOIS,MGX‐Montpellier GenomiX, IGF, CNRS, INSERM, Univ Montpellier, Montpellier

Laurence Guglielmi, IRCM, Univ Montpellier, Inserm, Montpellier, France

Pierre MartineauIRCM, Univ Montpellier, Inserm, Montpellier, France

Justine Bertrand‐Michel, MetaToul‐LIPIDOMIQUE, Institut des Maladies Métaboliques et Cardiovasculaires (I2MC) Inserm/Université Paul Sabatier UMR1048, Toulouse, France

Philippe LEGRAND,ICGM, Univ. Montpellier, CNRS, ENSCM, Montpellier, France

Joël Chopineau,ICGM, Univ. Montpellier, CNRS, ENSCM, Montpellier, France

Jean‐Marie DEVOISSELLE,ICGM, Univ. Montpellier, CNRS, ENSCM, Montpellier, France

Marie Morille , ICGM, Univ. Montpellier, CNRS, ENSCM, Montpellier, France

**Introduction**: In regards to their key role in intercellular communication, extracellular vesicles (EVs) have a strong potential as bio‐inspired drug‐delivery systems. Yet, EVs still failed to convince users as a pertinent drug delivery system (DDS). This could be explained by several drawbacks such as (i) scalability of production and isolation, (ii) lack of standard storage conditions, (iii) heterogeneity of vesicle population hindering EV clear identification and subsequent behavior, (iv) drug loading efficiency and reproducibility, or (v) plasma instability if considered for an intravenous (i.v) non autologuous administration.

**Methods**: With the aim of circumventing some of these well‐known issues, we specifically focused on switching the biological vision of these entities to a more physico‐chemical one, and to consider and fine‐tune EVs as synthetic vectors. To allow a rational use, we first performed a full physico‐chemical (size, concentration, surface charge, cryoTEM), biochemical (western blot, proteomics, lipidomics, transcriptomics) and biological (cell internalisation) characterisation of murine mesenchymal stem cell (mMSC)‐derived EVs. A stability study based on evaluating the colloidal behaviour of obtained vesicles was performed in order to identify optimal storage conditions (freezing, freeze‐drying).

**Results**: We evidenced the interest of using EVs instead of liposomes, in regards to target cell internalisation efficiency. EVs were shown to be internalised through a caveolae and cholesterol‐dependent pathway, following a different endocytic route than liposomes. We then characterised the effect of physical methods scarcely investigated with EVs (extrusion through 50 nm membranes, freeze‐drying, sonication) on EV size, concentration, structure and cell internalisation properties. Our extensive characterisation of the effect of these physical processes highlights their promise as loading methods to make EVs efficient delivery vehicles (Le Saux et al., 2020).

**Summary/Conclusion**: Based on these results, we are currently evaluating sEV‐mMSC for antibody fragments (single‐chain variable fragment, scFv) transfer in cell cytoplasm, as no reference synthetic vectors were reported till now, especially for in vivo use. Our preliminary results confirm the interest of associating scFv with sEV‐mMSC for intracellular delivery of a functional, biologically active protein. We propose here to present our recently published data as well as our last results on protein encapsulation.

### PS09.13 Intravenous injections of small extracellular vesicles correct obesity through modulation of hypothalamic AMPKα1

Edward Milbank, CiMUS‐ NeurObesity ‐ POL2 (Universidad de Santiago de Compostela)

Nathalia Dragano, Department of Physiology, CiMUS, University of Santiago de Compostela‐Instituto de Investigación Sanitaria

Ismael González‐García, Institute for Diabetes and Obesity, Helmholtz Diabetes Center, Helmholtz Zentrum München, German Research Center for Environmental Health (GmbH), German Center for Diabetes Research (DZD)

Grégory Hilairet, University of Angers

Marcos Rios Garcia, Department of Physiology, CiMUS, University of Santiago de Compostela‐Instituto de Investigación Sanitaria

Francisco Ruiz‐Pino, Department of Cell Biology, Physiology and Immunology, University of Córdoba; Instituto Maimónides de Investigación Biomédica (IMIBIC)/Hospital Universitario Reina Sofía

Verónica Rivas‐Limeres, Department of Physiology, CiMUS, University of Santiago de Compostela‐Instituto de Investigación Sanitaria

Patricia Mallegol, SOPAM, U1063, INSERM, University of Angers

Donald A Morgan, Department of Pharmacology, University of Iowa Carver College of Medicine

Ramón Iglesias‐Rey,Clinical Neurosciences Research Laboratory, Instituto de Investigación Sanitaria

Cristina Contreras, Department of Physiology, CiMUS, University of Santiago de Compostela‐Instituto de Investigación Sanitaria

Luisa VergoriINSERM U1063

Juan Cuñarro, Department of Physiology, CiMUS, University of Santiago de Compostela‐Instituto de Investigación Sanitaria

Begoña Porteiro,Department of Physiology, CiMUS, University of Santiago de Compostela‐Instituto de Investigación Sanitaria

Anxo Vidal,Department of Physiology, CiMUS, University of Santiago de Compostela‐Instituto de Investigación Sanitaria

Juan Roa,Department of Cell Biology, Physiology and Immunology, University of Córdoba; Instituto Maimónides de Investigación Biomédica (IMIBIC)/Hospital Universitario Reina Sofía

Tomás Sobrino,Clinical Neurosciences Research Laboratory, Instituto de Investigación Sanitaria

Carlos Diéguez,Department of Physiology, CiMUS, University of Santiago de Compostela‐Instituto de Investigación Sanitaria

Rubén NogueirasDepartment of Physiology, CiMUS, University of Santiago de Compostela‐Instituto de Investigación Sanitaria

Cristina García‐Cáceres,Institute for Diabetes and Obesity, Helmholtz Diabetes Center, Helmholtz Zentrum München, German Research Center for Environmental Health (GmbH), German Center for Diabetes Research (DZD)

Manuel Tena‐Sempere, Department of Cell Biology, Physiology and Immunology, University of Córdoba; Instituto Maimónides de Investigación Biomédica (IMIBIC)/Hospital Universitario Reina Sofía

Carmen Martínez,INSER U1063, Centre Hospitalo‐Universitaire d'Angers

Kamal Rahmouni, Department of Neuroscience & Pharmacology, University of Iowa Carver College of Medicine

Miguel López,Department of Physiology, CiMUS, University of Santiago de Compostela‐Instituto de Investigación Sanitaria

Ramaroson Andriantsitohaina,INSERM U1063 SOPAM

**Introduction**: Current pharmacological therapies for treating obesity are scarce and of limited efficacy. Recent data have demonstrated that genetic ablation or loss of function of AMP‐activated protein kinase alpha 1 (AMPKα1) in steroidogenic factor 1 (SF1) neurons of the ventromedial nucleus of the hypothalamus (VMH) induces feeding‐independent resistance to obesity. This action involves sympathetic activation of brown adipose tissue (BAT) thermogenesis.

**Methods**: Herein, we have developed a nanobiomedicine approach using neuronal‐targeted exosomes to deliver a plasmid encoding an AMPKα1 dominant negative mutant (AMPKα1‐DN) for targeting VMH SF1 neurons following intravenous injections.

**Results**: SF1‐AMPKα1‐DN loaded exosomes significantly decreased body weight in obese mice. Notably, this effect was feeding‐independent but involved sympathetic nerve activation and an increase in BAT thermogenesis.

**Summary/Conclusion**: These results underscore the potential of this exosome‐driven approach allowing hypothalamic AMPK specific neuronal‐targeting, introducing a new strategy to manipulate body weight and eventually limit obesity expansion.

## PS10 Separation and Concentration

Chair: Raghubendra Dagur, University of Nebraska Medical Center, United States

Chair: Willem Stoorvogel, Department of Biochemistry & Cell Biology, Faculty of Veterinary Medicine, Utrecht University, Utrecht, The Netherlands

### PS10.01 Evaluation of Capillary‐Channeled Polymer Fibers of Different Geometry for the Simultaneous Isolation and Quantification of EVs using High Performance Liquid Chromatography

Lacey S. Billotto, Clemson University

R. Kenneth Marcus, FRSC, FAAAS, FSAS, FNAI, Clemson University

**Introduction**: The capillary‐channeled polymer (C‐CP) fiber stationary phases employed in HPLC workflows have previously demonstrated high efficiency in the isolation and quantification of proteins, extracellular vesicles (EVs), and lentiviruses from complex biofluid matrices. The hydrophobicity‐based isolation method has produced high concentrations of vesicle targets with preserved morphology and bioactivity. Further, the HPLC method allows for quantitative information to be gained by on‐line absorbance and/or fluorescence detection. Despite significant chromatographic benefits previously exhibited by the 8‐channeled fiber morphology, a new, trilobal‐shaped C‐CP fiber phase is evaluated here to improve chromatographic performance during EV and other biomolecule isolations using the hydrophobic interaction chromatography (HIC) method.

**Methods**: The trilobal polyester (PETY) C‐CP fiber phase was employed in a HIC protocol using a Dionex Ultimate 3000 HPLC system. EVs were isolated from cell culture media, urine, blood (plasma and serum), and saliva using the PETY C‐CP fiber stationary phase. UV‐Vis absorbance (scattering) detection was used to monitor the eluting species at 216 nm. Successful EV isolations were confirmed using scanning electron microscopy (SEM), nanoparticle tracking analysis (NTA), and bioassays for total protein quantification.

**Results**: The trilobal fiber phase provides greater packing homogeneity than the eight‐channeled form, increasing the chromatographic performance during EV isolations. The HIC method allows the isolation and quantification of vesicle targets in less than 15 min., on volumes from 10–100s of microliters. Chromatographic figures of merit including separation efficiency, peak area, fraction purity, and dynamic binding conditions were compared for the EV separations from the biological matrices for the two fiber shapes.

**Summary/Conclusion**: The C‐CP fiber platform having a trilobal geometry further enhances the isolation efficiency for EVs (and viruses) on appropriate time (< 15 min) and financial scales (< $20 per column, having 30 repetitions) while yielding high purity, concentrated vesicle recoveries for clinical analysis, fundamental studies, and biopharmaceutical applications.

### PS10.02 Assessment of Scalability of a Polyethylenimine‐based Method for Isolation of Functional Extracellular Vesicles

Marie Ange Djeungoue Petga, Atlantic Cancer Research Institute (ACRI)

Catherine R. Taylor, Msc, Atlantic Cancer Research Institute

Surendar‐Reddi Dhadi, Ph.D., Atlantic Cancer Research Institute

Biji Anish, Atlantic Cancer Research Institute

Awanit Kumar, Ph.D., Atlantic Cancer Research Institute

Jeremy Roy, Atlantic Cancer Research Institute

Stephen M Lewis, Atlantic Cancer Research Institute, Department of Chemistry and Biochemistry, Université de Moncton

Rodney J. Ouellette, Atlantic Cancer Research Institute, Department of Chemistry and Biochemistry, Université de Moncton

**Introduction**: Extracellular Vesicles (EVs) are a heterogeneous group of biological nanoparticles, which are secreted by every cell type and can be isolated from a variety of biological fluids. Due to their established role in intercellular communication and signaling, EVs have highly diverse physiological functions (immune response regulation, reproduction, growth, tissue repair, etc.). EVs have also gained significant interest as potential therapeutic delivery vehicles since they can transfer endogenous and exogenous cargo into recipient cells. In order to enable their therapeutic potential, there is an industrial need for inexpensive and scalable EV isolation methods. A variety of EV isolation methods have been developed, such as ultracentrifugation, ultrafiltration, antibody‐capture, and polymer‐based sedimentation, but there are some limitations due to EV isolation efficiency, purity, and complexity for each method. Our laboratory has determined that polyethylenimine (PEI) polymers can be used for rapid, high‐efficiency isolation of EVs from a variety of biofluids with standard lab equipment. PEI is an organic, water soluble, linear or branched polymer well‐known as a transfection reagent and which is relatively non‐toxic and biocompatible. The aim of this study is to determine if EV isolation with PEI is efficient and scalable and to test the functionality of the recovered EVs.

**Methods**: The utility of branched and linear PEI to isolate GFP‐labeled EVs from conditioned cell culture media was tested on volumes ranging from 1 mL to 50 mL. The PEI‐captured GFP+ EVs (PEI‐EVs) were dissociated from PEI using either salt or heparin solutions and characterized using nanoparticle tracking analysis, Western blotting, flow cytometry and fluorescence spectrophotometry. PEI‐captured EVs from DSP1‐CD63/DSP2 EV‐producing cells (Toribio V. et al. Sci Rep 9, 10522, 2019) were also tested for their ability to deliver cargo to SUM159 cells and were compared to EVs isolated by ultracentrifugation and Exoquick isolation methods.

**Results**: Our data show that the capture of EVs by PEI is scalable up to 50 mL with a better concentration factor resulting from use of larger volumes. Our preliminary data suggest that PEI‐EVs captured in this model cell culture system are functional when delivered to recipient cells.

**Summary/Conclusion**: The ability of PEI polymers to deliver oligonucleotides by adhering and condensing DNA to form spherical complexes that are easily endocytosed by cells could potentially lead to better efficiency of PEI‐EVs uptake by recipient cells. The constantly evolving interest in EVs as potential therapeutics needs inexpensive, reproducible, efficient, non‐toxic, scalable and biocompatible isolation methods to improve safety and meet future clinical therapeutic needs.

### PS10.03 MagPEG: a fast and inexpensive EV isolation/analysis solution for high throughput biomarker detection

li sun, FSU College of Medicine

David G. Meckes, PhD, FSU college of medicine

**Introduction**: Current extracellular vesicle (EV) isolation methods depend on large expensive equipment like ultracentrifuges and are laborious and time consuming. There is also currently no method available for high throughput isolation to meet clinical demands.

**Methods**: We present a method that combines our previous published ExtraPEG (Rider et al. 2016) method and magnetic beads to isolate EVs from concentrated cell culture medium and human serum samples.

**Results**: With this newly developed workflow we have successfully isolated up to 16 EV samples from pre‐cleared serum in less than 30 minutes. Western blot and nanoparticle tracking analysis (NTA) of the purified EVs revealed higher or equivalent recovery and purity with this method compared to ExtaPEG or size exclusion chromatography methods. Moreover, DNA/protein purification and profiling steps could be seamlessly integrated into the isolation workflow. To profile EV protein markers, EVs were lysed from the binding step and covalently bound to the surface of the beads. TotalSeq antibodies, which are commercially available DNA barcoded conjugated antibodies, were then used to detect the EV proteins on the beads as measured by real‐time qPCR. With this combined TotalSeq method, researchers can easily complete EV isolation and protein profiling within 8 hours. Because this method utilizes magnetic beads, it could be easily adapted to an automated liquid handing platform to expand its capacity and applications.

**Summary/Conclusion**: Taken together, we provide a high throughput method for EV isolation and molecular analyses that may be used for sensitive biomarker detection from biological fluids.

### PS10.04 Comparison of Amicon and Vivaspin ultrafiltration columns for the pre‐concentration of culture medium derived extracellular vesicles (EV) prior to size‐exclusion chromatography (SEC)

Maria Fernandez‐Rhodes, School of Sport, Exercise and Health Sciences, Loughborough University, Loughborough, United Kingdom.

Soraya Williams, School of Sport, Exercise and Health Sciences, Loughborough University, Loughborough, United Kingdom.

Bahman Adlou, Loughborough University

Mark P. Lewis, School of Sports, Health and Exercise Sciences, Loughborough University, Leicestershire, UK.

Owen Davies, School of Sport, Exercise and Health Sciences, Loughborough University, Loughborough, United Kingdom.

**Introduction**: SEC represents a ubiquitously applied method for the isolation of EV‐enriched fractions. When applying SEC, manufacturers suggest an optimal window of isolation. However, this window has not been optimised in skeletal muscle cultures. Furthermore, this method is frequently combined with ultrafiltration (UF) to increase the throughput of the technique and allow for scalable EV isolation. However, the impact of different UF columns and sample pre‐concentration on the critical parameters, such as the recovery window had not been comprehensively assessed. In this study we compared Amicon and Vivaspin pre‐SEC UF concentration protocols to distinguish their effects on critical EV outputs.

**Methods**: C2C12 (murine myoblasts) cultures were collected after 48 hours differentiation, pre‐concentrated by UF, using Amicon or Vivaspin centrifugal filters following manufacturer's instructions, and processed using SEC (IZON qEV 35nm). 30 fractions collected were analysed individually by Nanoparticle Tracking Analysis (NTA), Western Blot (WB) for Alix, Annexin A2, TSG101, CD9, CD63, ApoA1 and Calnexin, and CD63 specific ExoELISA.

**Results**: Using Amicon devices most of the EV content was found from fractions 1–5. Using Vivaspin devices, between fractions 2–10, recovering ∼35% more particles. Average size distributions of particles among targeted fractions are in range and similar between both Amicon and Vivaspin (163.41±30.12 nm vs. 158.15±19.33 nm). Higher particle‐to‐protein (PTP) ratios were detected in EVs fractions obtained following Vivaspin pre‐concentration, except for the first three. The presence of EV in optimal targeted fractions was confirmed showing positive signal of CD9, CD63 and TSG101. Finally, lack of ApoA1 expression was also detected up to fraction 6 and 9 respectively, confirming the absence of lipoproteins.

To account for observed differences and recover a maximum and comparable yield of material, samples were combined and re‐concentrated using fractions 2–10 for each method. When concentrating with Vivaspin, SEC recovered ∼15% more particles relative to Amicon. EV samples re‐concentrated with Amicon have a larger average particle size (153.96 ± 12.31 nm) compared to Vivaspin (119.76 ± 15.30 nm). PTP ratios were similar between both. Regarding EV markers, CD9 is appearing more prominent following Amicon pre‐concentration, whilst TSG101 and CD63 enrichment was evidence following Vivaspin pre‐concentration.

**Summary/Conclusion**: SEC columns manufacturers suggest that the majority of the EV are obtained from fractions 7 to 14, but this was not accurate for the isolation of myoblast EVs. Furthermore, outcomes such as particle concentration and marker profile varied depending on the type of UF device applied for sample pre‐concentration. To conclude, we have identified that when applying UF and SEC for the isolation of myoblast EV from cell culture medium, different EV populations might be recovered depending on the UF filter applied.

### PS10.05 Comparison of Extracellular Vesicle Isolation Processes for C2C12 Mouse Myoblast Cells

Soraya Williams, School of Sport, Exercise and Health Sciences, Loughborough University, Loughborough, United Kingdom.

Alice Law, NanoFCM

Ben Peacock, NanoFCM

Mark P. Lewis, School of Sports, Health and Exercise Sciences, Loughborough University, Leicestershire, UK.

Owen Davies, School of Sport, Exercise and Health Sciences, Loughborough University, Loughborough, United Kingdom.

**Introduction**: Evidence suggests skeletal muscle (SkM) myotube derived extracellular vesicles (EVs) drive SkM processes, indicating their therapeutic potential. However, studies are limited by the ability to efficiently obtain high yield purified populations from minimal sample volumes in a cost‐effective manner. This study compared isolation methods to understand EV output variation using Nanoparticle tracking analysis (NTA), BCA protein assay, western blots (CD9, CD63, Alix, Annexin A2), ExoELISA's (CD63 and CD81) and nano flow cytometry (NanoFCM) (CD9, CD63, CD81). To further understand advantages and limitations (e.g. efficiency, cost, scalability, purity) a survey was internationally distributed via Qualtrics within the EV community.

**Methods**: EVs were isolated from C2C12 mouse myoblast cells by ultracentrifugation (UC), polyethylene glycol (PEG) precipitation, Total Exosome Isolation Reagent (TEIR), an aqueous two‐phase system (ATPS) utilising PEG and dextran, and size exclusion chromatography (SEC). ATPS was repeated with multiple washes of the top PEG phase for additional purification.

**Results**: TEIR displayed the highest particle concentration (1.15E+09) followed by UC (8.46E+08) and PEG precipitation (7.11E+08), with protein quantities 1606μg/ml, 693μg/ml and 1031μg/ml respectively. SEC and ATPS showed higher protein quantities of 4066μg/ml and 3173μg/ml and particle counts of 4.57E+08 and 6.31E+08 respectively. ATPS with repeat washes displayed the lowest particle counts (2.71E+08) and significantly lower protein quantity (135μg/ml). EV markers were present in all isolations but with distinctly variable profiles.

**Summary/Conclusion**: This high‐throughput comparison study for myogenic EVs indicates that methods pull out EV‐enriched fractions with variable purity and marker profiles. This could have significant implications when analysing downstream biological function and therapeutic utility.

### PS10.06 Magnetic bead‐based capture‐and‐release of surface marker‐defined EV subpopulations allows study of their functionality

Rowan Frunt, University Medical Center Utrecht, The Netherlands

Ioanna Paspali, University Medical Center Utrecht, the Netherlands

Olivier Gerrit G. de Jong, PhD, CDL Research, University Medical Center Utrecht and Department of Pharmaceutics, Utrecht Institute for Pharmaceutical Sciences, (UIPS), Faculty of Science, Utrecht University, The Netherlands

Mark Tielemans, University Medical Center Utrecht, the Netherlands

Raymond M. Schiffelers, University Medical Center Utrecht, the Netherlands

Pieter Vader, CDL Research, University Medical Center Utrecht, The Netherlands

Sander A.A. A. Kooijmans, University Medical Center Utrecht

**Introduction**: The surface composition of extracellular vesicles (EVs) is highly heterogeneous, potentially reflecting functional differences between EV subpopulations. However, this hypothesis awaits confirmation as it remains challenging to purify intact EV subpopulations based on their surface molecule expression. We developed a universal magnetic bead‐based capture‐and‐release method to enrich intact EV subpopulations by their surface profile and compare their characteristics.

**Methods**: EVs from SKOV‐3 and MDA‐MB‐231 cells were isolated using size exclusion chromatography. EV subpopulations were then isolated based on presence of CD9, CD63, CD81 or phosphatidylserine (PS) using a magnetic bead‐based capture‐and‐release platform. Proteinase K protection assays were performed to study EV integrity. EV subpopulations were further characterized by transmission electron microscopy (TEM), Nanoparticle Tracking Analysis (NTA) and western blotting. EV labelling efficiency with fluorescent dyes PKH67 and Cell Tracker Deep Red (CTDR) was measured using fluorescence spectroscopy and cellular uptake was examined using flow cytometry.

**Results**: EV subpopulations released from antibody‐coated magnetic beads appeared intact as observed by NTA and TEM analysis, and lacked typical contaminants observed in the initial EV isolate. EV membrane integrity was preserved during the release procedure, as their internal protein content remained protected from proteinase K digestion. Western blot analysis revealed that EV subpopulations differed in their protein composition. Strikingly, EVs enriched based on PS exposure lacked most canonical EV markers, but were more effectively labelled with the esterase‐dependent fluorescent dye CTDR than the other assessed subpopulations. Cellular uptake experiments revealed that EV subpopulations isolated based on their CD9 and CD81 expression were taken up more efficiently by HMEC‐1 and MDA‐MB‐231 recipient cells than CD63 or PS enriched subpopulations.

**Summary/Conclusion**: We here employed a novel magnetic bead‐based capture‐and‐release platform to show that composition, enzymatic activity and cellular uptake efficiency differs between EV subpopulations with distinct surface profiles. The platform can in principle be used in combination with any capture antibody, allowing its use to map EV surface‐functionality relationships and enrich EVs with desirable characteristics for therapeutic purposes.

### PS10.07 Characterizing extracellular vesicles from cerebrospinal fluid by a novel size exclusion chromatography method

Yael Hirschberg, VITO

Karin Schildermans, VITO

Annemieke van Dam, Amsterdam UMC

Karen Sterck, University of Antwerp

Kurt Boonen, VITO

Inge Nelissen, VITO nv

Yannick Vermeiren, University of Antwerp

Inge Mertens, University of Antwerp

**Introduction**: Extracellular vesicles (EVs) have recently been considered as a potential biomarker source for a variety of diseases, including neurodegenerative disorders. EVs are important mediators of intercellular communication due to their capacity to transfer genetic material, lipids and proteins. By means of their communication role, interesting biomarkers are often enriched in EVs compared to total biofluid. In order to isolate EVs from a biofluid, like cerebrospinal fluid (CSF), a variety of isolation methods are available of which each methods shows different results, and, to date, there is no golden standard. Our study evaluates the use of size exclusion chromatography (SEC) by the SmartSEC HT kit for EV isolation from CSF.

**Methods**: EVs were obtained from 500μL CSF samples, isolated by SmartSEC HT. SmartSEC traps contaminants, like bulk proteins, into the resin beads and subsequently elutes EVs. To evaluate this isolation method, various characterisation analyses were run. To detect cargo or surface proteïns, analyses on the ExoView R100, Surface Plasmon Resonance imaging (SPRi) and LC‐MS based proteomics analysis were performed. Concentration, and size of the vesicles were evaluated by use of transmission electron microscopy (TEM), and fluorescence nanotracking analysis (Zetaview).

**Results**: By means of the ExoView, tetraspanins such as CD81 and CD9 were measured on the surface of the EVs. Other EV‐specific proteins, e.g. lactadherin, flotillin‐1, and HSP70, were detected by SPRI. Untargeted protein analysis revealed the cargo proteins. TEM images visualized the presence of EVs. Particle size and concentration was calculated by nanotracking analysis, and more specifically the particles with a lipid membrane, like EVs, with fluorescence nanotracking analysis.

**Summary/Conclusion**: As EVs hold a lot of promise in the biomarker field, a standard high‐performance isolation method ‐ regarding purity and yield " is essential. From our data, SmartSEC HT is suggested to be an efficient size exclusion chromatography EV isolation method for low volume CSF samples, especially for high throughput, since samples are loaded on a 96‐well plate.

### PS10.08 High purity extracellular vesicles isolated from high‐density cultures using novel Capto Core bind‐elute chromatography

Scott Edward Bonner, University of Oxford

Simonides Immanuel van de van de Wakker, Department of Experimental Cardiology, University Medical Center Utrecht, Utrecht University, The Netherlands

Eduard Willms, Department of Biochemistry and Genetics, La Trobe Institute for Molecular Science, La Trobe University, Melbourne, Australia

Sofie Heij, Faculty of Science, School of Pharmacy, Utreht University, Utrecht, The Netherlands

Imre Mäger, Department of Paediatrics, University of Oxford, Oxford, United Kingdom.

Matthew Wood, Department of Paediatrics, University of Oxford, Oxford, United Kingdom.

Pieter Vader, CDL Research, University Medical Center Utrecht, The Netherlands

**Introduction**: As application of therapeutic extracellular vesicles (EVs) has expanded, so has the need to obtain clinically relevant EV concentrations, thus high‐density cell culture techniques such as bioreactors are being adopted. Since 2015 ISEV has seen a significant increase in use of size exclusion chromatography (SEC) for EV purification. SEC yields high purity EVs, though it is time consuming and can result in low yields; problems further exacerbated by high‐density cultures. Here we attempt to validate the use of Capto Core bind‐elute chromatography as a novel alternative to SEC to purify EVs from high‐ and low‐density cultures.

**Methods**: HEK293T cells were cultured in a Fibre Cell Systems C2011 bioreactor. Cardiac progenitor cells (CPCs) were cultured in 2D. Supernatant was periodically harvested and concentrated. EVs were purified by SEC, or Capto Core chromatography with benzonase treatment. EVs were characterised according to MISEV2018 guidelines and quantified by nanoparticle tracking analysis. Protein and NA concentrations were determined. EV function was assessed using CPC derived EVs in endothelial ERK/AKT phosphorylation assays and wound healing assays.

**Results**: Three successive passes of high‐density conditioned supernatant through a SEC column were needed to isolate EVs, resulting in significant decreases in EV yield versus Capto Core purification which yielded six‐fold higher EV concentrations. Protein‐particle ratios presented a two‐fold increase in EV purity from Capto Core versus SEC. What's more, NA‐particle ratios presented a greater purity of EVs from NAs in Capto Core. From low‐density cultures, EV purification by Capto Core gave higher EV yields and preserved function compared to SEC.

**Summary/Conclusion**: Overall, Capto Core chromatography is a viable, accessible and time saving alternative to SEC for EV purification from high and low‐density cultures. Capto Core purification provides high EV yields, high purity of EVs from free proteins and NAs and helps retain EV function.

### PS10.09 Comparison of scalable isolation methodologies using different EVs sources with potential as functional ingredients

Joaquin Espi, AINIA

Laura Soriano, PhD, AINIA

Begoña Ruiz, AINIA

**Introduction**: Extracellular vesicles (EVs) are increasing its potential in different applications. Despite of the advances in the characterization and evaluation of EVs in recent years, the scaling up of the production process at the industrial level remains a bottleneck in this field.

The objective was to explore and compare existing methodologies to isolate EVs, including techniques with available industrial scale equipment, to compare yields, purity and functionality and to validate them using EVs from different sources. For that, plant‐, probiotic‐ and human cells‐derived EVs were isolated by 3 different isolation techniques and characterized in terms of yield, morphology, and functionality.

**Methods**: To meet our goal, ultracentrifugation, size exclusion chromatography and ion exchange chromatography were performed to isolate EVs from the different origins indicated: from a human hepatic cell line, a Lactobacillus strain and a plant. Then, EVs were characterized by NTA and electronic microscopy, and evaluated in vitro for its molecular effect in immune cells, analyzing changes in the expression of the transcription factor NF‐κβ that controls cytokine production and cell survival, after its activation using lipopolysaccharide (LPS).

**Results**: The EV yields obtained after downstream processing were calculated for each source selected and isolation technique used by means of NTA and total protein content. Moreover, EVs were visualized by electron microscopy for its morphology and size characterization. The in vitro evaluation of isolated EVs was based on the effect on the transcription factor NF‐κβ, which is involved in cellular responses to stressful stimuli such as cytokines, UV radiation, free radicals, and bacterial or viral antigens, among others.

**Summary/Conclusion**: In order to develop an isolation method which allows to obtain functional EVs and transfer the downstream process at industrial scale, two chromatographic techniques were studied as an alternative to ultracentrifugation. Additional experiments will be needed to increase and optimize the yields obtained in the EV manufacturing process.

### PS10.10 A Label‐free Extracellular‐vesicle Automated Purification System (LEAP System)

Yuchao Chen, WellSIM Biomedical Technologies Inc

Gianluca Roma, WellSIM Biomedical Technologies Inc

Fei Liu, WellSIM Biomedical Technologies Inc

**Introduction**: Extracellular vesicles (EVs) are small lipid bilayer particles secreted by most cell types, playing a vital role in intercellular communication. Profiling of EV‐derived biomarkers (e.g., RNA, protein, and lipid) provides a promising approach for early diagnosis of various diseases, including cancers, neurodegenerative diseases, and autoimmune diseases. However, one of the primary challenges to the research and application of EVs is lack of a robust and reliable method for effective and efficient EV isolation from complex biofluids. Scientists are suffering from tedious work process, long processing time, high cost, or poor EV quality (e.g., purity, yield, and integrity). It has been emphasized in the recent publications that developing efficient and reliable EV isolation methods is urgent to further advance in this field. Therefore, development of a more efficient, flexible, and robust EV‐isolation technology is highly desirable.

**Methods**: We present a Label‐free Extracellular‐vesicle Automated Purification System (LEAP System) by implementing a novel strategy to achieve clog‐free ultrafiltration of EVs. The system is based on alternating negative pressure combined with dual‐membrane harmonic oscillations to suppress the formation of fouling layers by resuspending particles into the liquid while preserving the particle integrity.

**Results**: This technology allows for processing flexible sample volumes as well as simultaneous EV isolation, enrichment, and medium exchange in a non‐invasive and cost‐effective manner, which provides another powerful ultrafiltration strategy and shows significant advantages over the conventional filtration approaches and current EV isolation technologies. Our LEAP system can improve processing speed by 15 folds (4 min vs. 60 min for 10 mL urine) and enrich EVs by 1000 folds (from 50 mL urine to 50 μL).

**Summary/Conclusion**: Our studies demonstrated that alternating negative pressure and membrane oscillation can both substantially improve the processing speed and throughput volume by limiting the fouling buildup. Furthermore, EVs isolated by the LEAP System have superior yield, purity, and integrity over the other main‐stream EV isolation techniques.

### PS10.11 Evolving reversible iMmunocapture by membrAne sensing peptides: towaRds scalable extracellular VEsicLes isolation ‐ (MARVEL)

Marina Cretich, CNR‐SCITEC

**Introduction**: The use of EV as either a therapeutic agent or as a source of diagnostic biomarkers strictly relies on the high‐yield isolation and recovery of pure, homogeneous EV sub‐populations from complex biological matrices. In this scenario, a particularly arduous task is to design robust, versatile, scalable and economically viable processes for achieving such goal beyond the research‐grade scale. Revolutionary, versatile, and cost‐effective methodologies to enable scalable EV isolation in high purity (>95%) from bio‐samples, from laboratory analysis (μL to mL) to the manufacturing (>1L) scale, are still necessarily demanded to open new perspectives in EV‐based therapeutics and diagnostics.

**Methods**: MARVEL out‐of‐the‐box solution to these limitations is a paradigm shift from antibodies to peptides as an alternative class of affinity ligands characterized by high efficiency of EV capturing. In particular, MARVEL will introduce the use of membrane‐sensing peptides (MSP) as novel ligands for the size‐selective capturing of small EV, unbiased by differential surface protein expression (membrane as universal EV marker); in parallel, specific peptide probes (SPP) with high affinity for clinically relevant EV protein markers will be developed to enrich selective EV subpopulations. Key figures of merit for peptide ligands are the ease of chemical manipulation, offering virtually unlimited possibilities for system integration, and the limited costs of preparation with well‐known possibilities for scale‐up of production.

**Results**: Thanks to its versatile and modular nature, system integration of MARVEL technology result in a portfolio of products to be applied for EV isolation on different scales, opening new scenarios for both diagnostics and therapeutics. These will include: 1) lab‐scale and point of need tools for urinary EV enrichment and analysis with unprecedented performances, enabling the liquid biopsy of bladder cancer and 2) the first integrated TFF‐affinity isolation system for therapeutic EV on a manufacturing scale.

**Summary/Conclusion**: MARVEL expects to produce direct impacts on the field of EV by empowering the sustainability of their use in both regenerative medicine and in many diagnostics workflows. Such empowerment is expected to increment readiness level of EV technologies and endow them with clinical grade maturity. This will ultimately impact society by providing better clinical outcomes at lower costs through the precision medicine paradigm with implications on life quality and sustainability of public healthcare

### PS10.12 Optimization of Microfluidic Geometry for Extracellular Vesicle Liquid Biopsy

Colin L. Hisey, University of Auckland

AJ Tyler, University of Auckland

Arvin Lim, University of Auckland

Cherie Blenkiron, BSc, PhD, University of Auckland

Lawrence W. Chamley, University of Auckland

Richard Clarke, University of Auckland

**Introduction**: Microfluidic affinity‐based isolation of extracellular vesicle (EV) subpopulations holds immense potential for liquid biopsy applications in cancer diagnosis and monitoring. However, several important parameters including flow rates, linker chemistry, and channel geometry must first be optimized to improve capture efficiency and advance these tools towards clinical applications. In this study, we describe initial successes in microfluidic geometry optimization using supercomputer simulations.

**Methods**: Both pillar and herringbone microfluidic geometries were first parameterized and several assumptions were established, such as a periodic unit cell approximation, massless 100nm particles that perfectly follow streamlines, flow rate and channel height. COMSOL Multiphysics microfluidic and particle tracing modules were combined with an in‐house Python‐based parallel pattern search algorithm to optimize the channel geometries by maximizing the objective function of particle transmission probability. Simulations were performed on the Mahuika High Performance Computer Cluster (Cray CS400) at the New Zealand eScience Infrastructure (NESI).

**Results**: A chip design based on triangular pillar arrays was successfully optimized over the initial parameters, with a significant improvement being observed. The best solution was found to have a pillar radius of r = 20 μm and a pillar separation factor of a = 3.55. The staggered herringbone design was also explored, and found to be extremely sensitive to initial conditions. This supported the initial intent behind the herringbone design, which was to induce chaotic flow patterns.

**Summary/Conclusion**: A parallel pattern search optimization method using supercomputers demonstrates the potential for EV‐specific microfluidic geometry optimization. Experimental validation of the best and worst pillar geometries are currently being tested and other approaches for optimizing the herringbone geometries are being considered. In the future, simulations of microfluidic EV liquid biopsy will be valuable in advancing these devices towards clinical applications.

### PS10.13 Molecular evaluation of five different isolation methods for extracellular vesicles reveals different clinical applicability and subcellular origin

Rosanne E. Veerman, Karolinksa Institutet

Loes Teeuwen, Karolinksa Institutet

Paulo Czarnewski, Science for Life Laboratory

Gözde Güclüler Akpinar, Güclüler Akpinar

Ann‐Sofi Sandberg, Karolinska Institutet

Xiaofang Cao, Karolinska Institutet

Maria Pernemalm, Karolinska Institutet

Lukas Orre, Karolinska Institutet

Susanne Gabrielsson, Karolinska Institutet

Maria Eldh,Karolinksa Institutet

**Introduction**: Extracellular vesicles (EVs) are increasingly tested as therapeutic vehicles and biomarkers for disease, but still EV subtypes are not fully characterised and understood. Moreover, new methods for isolating EVs are emerging leading to discrepancy in isolation methods between published studies and thereby making the comparison of results difficult. Therefore, we conducted a study to compare commercially available methods based on five different principles by isolating EVs from both cell conditioned medium and 250 μl or 3 ml plasma samples.

**Methods**: The used methods included precipitation, membrane affinity, Size‐Exclusion Chromatography (SEC), iodixanol gradient and phosphatidylserine affinity. EVs were characterized by electron microscopy, Nanoparticle Tracking Analysis, electrophoresis for RNA quality, flow cytometry and LC‐MS/MS.

**Results**: The different methods yield samples of different morphology, particle size, purity, and proteomic profile. For the conditioned medium, SEC with a pore size of 35 isolated most number of EV proteins while membrane affinity isolated the purest samples with enrichment for larger EVs. Also for the plasma samples, membrane affinity isolated the purest samples, while SEC with a pore size of 70 isolated most EV proteins. Furthermore, bioinformatic analyses and comparisons with EV databases and plasma proteins show that the isolation methods reveal different levels of contamination of (lipo‐)proteins. Moreover, Gene Set Enrichment Analysis shows that the different isolation methods enrich for distinct subtypes of EVs with signatures derived from diverse cellular compartments.

**Summary/Conclusion**: This study provides information for optimising and designing future EV‐studies since our data shows that different methods might be favored depending on interest in EV subtype, sample number and origin, volume, reproducibility, and budget.

### PS10.14 Axillary lymphatic exudate from breast cancer patients: evaluation of different methods to isolate extracellular vesicles

Rossella Crescitelli, Sahlgrenska Cancer Centre, Department of Surgery, Institute of Clinical Sciences, Sahlgrenska Academy, University of Gothenburg

Karin Ekström, 1. Department of Surgery, Institute of Clinical Sciences, Sahlgrenska Academy at the University of Gothenburg, Gothenburg, Sweden. 2. Department of Surgery, Sahlgrenska University Hospital, Region Västra Götaland, Gothenburg, Sweden. 3. Wallenberg Centr

Hafsteinn Ingi Pétursson, 1. Department of Surgery, Institute of Clinical Sciences, Sahlgrenska Academy at the University of Gothenburg, Gothenburg, Sweden. 2. Department of Surgery, Sahlgrenska University Hospital, Region Västra Götaland, Gothenburg, Sweden.

Roger Bagge, 1. Department of Surgery, Institute of Clinical Sciences, Sahlgrenska Academy at the University of Gothenburg, Gothenburg, Sweden. 2. Department of Surgery, Sahlgrenska University Hospital, Region Västra Götaland, Gothenburg, Sweden. 3. Wallenberg Centr

**Introduction**: Extracellular vesicles (EVs) play a role in cell‐cell communication and are also involved in breast cancer development. EVs can be used as biomarkers for early detection, and to monitor disease during and after treatments. A source of EVs can be serous fluid obtained after axillary lymph node dissection (ALND). The aim of this study was to find a suitable method to isolate EVs from lymphatic exudate.

**Methods**: Approximately 50 ml of axillary drain fluid were collected from six breast cancer patients the day after ALND. Three EV isolation methods were tested: ultracentrifugation (UC) (n = 4) to collect large and small EVs, iodixanol cushion (UC‐DC) (n = 2) and size exclusion chromatography (SEC) (qEV10/35 nm) (n = 3). EVs were analyzed by TEM and NTA. Seven fractions (F1‐7) collected by SEC were further analyzed by western blot.

**Results**: TEM pictures from large and small EVs isolated by UC showed presence of round elements with different size (250 nm vs 100 nm) surrounded by strong background due to contaminants. Similar pictures were observed when the combined large and small EVs were further purified by UC‐DC. TEM pictures from single fractions obtained by SEC, showed presence of round elements in the fractions 2, 3 and 4, and an abundance of non‐EV elements in fractions 5, 6 and 7. The presence of EVs were confirmed by NTA analysis (UC: large EVs: mean 8.3 × 109 particles/ml, small EVs: mean 3.4 × 1010 particles/ml; UC‐DC: mean 4.5 × 109 particles/ml). NTA showed increased number of particles in F2‐4 compared to F5‐7 (mean 4.5 × 1010 particles/ml vs mean 3.9 × 109 particles/ml). Moreover, western blot analysis indicated that F2‐4 were composed by particles positive for EV markers (CD63 and Flotillin‐1) compared to F5‐7 which mainly contained protein contaminants (Albumin and ApoA1).

**Summary/Conclusion**: All methods were able to isolate EV subpopulations from lymphatic exudate, but SEC was the only one able to separate EVs from protein contaminants.

### PS10.15 The importance of including multiple cell lines when comparing Extracellular Vesicles separation methods: A Differential Ultracentrifugation and Polyethylene glycol‐based precipitation comparison

Sarai Martinez pacheco, School of Pharmacy and Pharmaceutical Sciences, Trinity Biomedical Sciences Institute, Trinity St. James's Cancer Institute & TRAIN‐EV Marie Skłodowska‐Curie Action‐Innovative Training Network, train‐ev.eu

Lorraine O'Driscoll, School of Pharmacy and Pharmaceutical Sciences, Trinity Biomedical Sciences Institute, Trinity St. James's Cancer Institute & TRAIN‐EV Marie Skłodowska‐Curie Action‐Innovative Training Network, train‐ev.eu

**Introduction**: Several techniques have been developed to harvest extracellular vesicles (EVs) from biofluids, including cell conditioned media (CM). While differential centrifugation (dUC) remains an excellent commonly used method, a limitation is its scalability. This study aimed to investigate the reproducibility of Polyethylene glycol (PEG)‐based precipitation to enrich EVs from CM and compare its efficacy with dUC.

**Methods**: With both dUC and PEG, efforts were made to separate EVs from CM of three independent cell lines, HCC1954, SKBR3 and EFM192A. In brief, CM was divided equally between dUC and PEG. dUC was performed according to Thery et al1. PEG method was as described by Ludwig et al2. EVs were fundamentally characterised by immunoblots for positive and negative EV markers, NTA and TEM.

**Results**: EVs separated using both dUC and PEG carried syntenin, CD9 and CD63 (positive markers) and lacked GR94 and calnexin (negative markers). With CM from SKBR3 and HCC1954, no significant differences occurred between the particle numbers/per cell, regardless of whether dUC or PEG was used. However, with EFM192 CM significantly (p = 0.0036) difference EV/particle numbers resulted from the two methods. Regarding size estimates, significantly (p = 0.0023) differences in SKBR3‐EV sizes were observed following dUC versus PEG (125.7nm and 110.2 nm, respectively). However, this was not so with HCC1954‐EVs or EFM192A‐EVs.

**Summary/Conclusion**: The PEG‐based method was equally reproducible and less laborious than dUC. Both methods were reproducible within the method itself. However, whether or not similar quantities and/or sizes of EVs were obtained with PEG and dUC was cell line dependent. This indicates that careful consideration is necessary when comparing EV separation methods and CM from more than one cell line/biological source should be analysed.

## PS11 Sources of EVs: Sample Collection and Processing

Chair: Cecilia Lässer, University of Gothenburg, Sweden

Chair: Pia Siljander, University of Helsinki, Finland

### PS11.01 Lipid domains as new key players in the red blood cell vesiculation process upon storage?

Anne‐Sophie Cloos, De Duve Institute

Marine Ghodsi, De Duve Institute

Amaury Stommen, De Duve Institute

Juliette Vanderroost, De Duve Institute

Patrick Van der Smissen, De Duve Institute

Nicolas Cellier, Belgian Red Cross

Tome Najdovski, Belgian Red Cross

Donatienne Tyteca, De Duve Institute

**Introduction**: The shedding of extracellular vesicles (EVs) from the red blood cell (RBC) surface affects its functionality. Using blood stored in K+/EDTA tubes at 4°C, we recently revealed that the intracellular calcium increase, the oxidative stress and alterations of the RBC plasma membrane lipid composition, organization in lipid domains and biophysical properties contribute to EV biogenesis (Cloos et al., 2020).

**Methods**: To explore the relevance of our observations to RBC concentrates employed in transfusion medicine, we here used 12 RBC concentrates stored for up to 55 days. We determined EV abundance, morphology, size and biogenesis mechanisms.

**Results**: EVs exhibited a constant size of ∼180nm. Within the legal conservation period of 42 days, the average vesiculation rate was of 7–8 EVs released per RBC. Extension up to 55 days did not exceed 20 EVs released per RBC, with one exception. As expected, storage was associated with decreased ATP content and a loss of biconcavity at the benefit of echinocytes and spherocytes. Surprisingly, the vesiculation process was not accompanied by modifications of the RBC intracellular calcium, the reactive oxygen species content or the plasma membrane transversal asymmetry revealed by phosphatidylserine surface exposure. In contrast, an alteration of the spectrin cytoskeleton, an increase of sphingomyelin‐enriched domains and a decrease of cholesterol‐enriched domains were observed. All those changes were RBC concentrate‐ and storage time‐dependent.

**Summary/Conclusion**: The specific loss of cholesterol‐enriched domains could suggest their contribution to vesiculation in concentrates during the 55 days‐storage period. The next steps will be to evaluate EV abundance and lipid composition after specific modulation of lipid domains and to determine the potential role of donor specificity to explain differences between RBC concentrates. Our ultimate goal is to limit RBC vesiculation to increase the time of RBC concentrate conservation while minimizing the post‐transfusion effects.

### PS11.02 Characterization of milk Extracellular Vesicles (milk EVs) separated from bovine colostrum, first milk and mature milk over the lactation curve

Jessie Santoro, School of Pharmacy and Pharmaceutical Sciences & Trinity Biomedical Sciences Institute, Trinity College Dublin, Dublin 2, Ireland

Anindya Mukhopadhya, School of Pharmacy and Pharmaceutical Sciences & Trinity Biomedical Sciences Institute, Trinity College Dublin and Trinity St. James's Cancer Institute, Dublin 2, Ireland

Charlotte Oliver, Teagasc Food Research Centre

Andrea Brodkorb, Teagasc

Linda Giblin, Teagasc Food Research Centre

Lorraine O'Driscoll, School of Pharmacy and Pharmaceutical Sciences, Trinity Biomedical Sciences Institute, Trinity St. James's Cancer Institute & TRAIN‐EV Marie Skłodowska‐Curie Action‐Innovative Training Network, train‐ev.eu

**Introduction**: While cow milk has a specific composition, during the lactation period, this composition differs. Extracellular vesicles (EVs) in milk contribute in regulating the biological processes and cellular communication. Recently, we established an optimal methodology to remove non‐EV proteins from complex ‘milk matrix’ by combining isoelectric precipitation followed by gradient ultracentrifugation. In this study, we aim to separate EVs over the lactation curve i.e. from colostrum, first milk and mature milk and characterise them.

**Methods**: Three Holstein‐Friesian cows were selected for the study and colostrum (Col) was collected within 24 hours post‐calving, first milk (FM) was collected 7‐days post calving and mature milk was collected every month up‐to month 9 (months 1, 5 and 9 used in this study). All samples were treated with HCl to precipitate casein micelles and protein aggregates were removed by filtration. EVs were separated using Optiprep density gradient using the bottom‐up approach. EVs characterisation was performed by BCA to estimate total protein concentration, SDS page to characterise milk proteins; immunoblotting and imaging flow cytometry to investigate EVs specific markers, NTA to evaluate particle size and concentration and TEM to evaluate EVs morphology.

**Results**: No difference in EV size and concentration was observed between Col (1.19E+14 particles/mL) and FM (9.36E+13 particles/mL). Compared to Col and FM, lower concentration of EV particles were observed in mature milk (2.32E+12 particles/mL) (P < 0.01), whereas no difference in size were observed. Particles resembling EV morphology were detected by TEM analysis. Immunoblotting analysis indicated that Actinin4 was absent in all samples, TSG101, CD63 and CD9 were detected in all analysed milk samples. Imaging flow cytometric analysis indicated that there were no differences in samples for HLADR, Col had significantly higher CD9 and CD63 positive particles compared to FM and mature milk (P < 0.05).

**Summary/Conclusion**: Colostrum and first milk samples are enriched in EVs compared to mature milk and colostrum has the highest concentration of EVs which then decrease and is maintained throughout the milking period.

### PS11.03 A chemically defined, xeno‐ and blood‐free culture medium sustains increased production of small extracellular vesicles from mesenchymal stromal cells

Aliosha Figueroa, Cells for Cells, Santiago, Chile

Catalina de la Fuente, Cells for Cells, Santiago, Chile

Yessia Hidalgo, Cells for Cells, Santiago, Chile

Ana María Vega‐Letter, Cells for Cells, Santiafo, Chile

Rafael Tapia‐Limonchi, Cells for Cells, Santiago, Chile

Maroun Khoury, Cells for Cells, Santiago, Chile

Francisca Alcayaga‐Miranda, Cells for Cells, Santiago, Chile

**Introduction**: small Extracellular Vesicles (sEV) are acquiring a major interest in medicine as cell‐free therapy. However, their successful clinical translation will rely ultimately on their large‐scale production necessary to achieve the doses‐required quantities apted to guarantee an efective treatment in patients. The aim of our research was to develop a chemically defined medium (Oxium™EXO) capable of increasing sEV secretion without modifying the typical physical and biochemical sEV's characteristics.

**Methods**: We aimed to compare the sEV production from mesenchymal stromal cells (MSCs) using Oxium™EXO (developed by Cells for Cells S.A.), standard DMEM and a commercially available sEV‐production medium. The harvest kinetics considered several time points at 2, 4 and 6 days. Following the sEV production cycle, the phenotype and cell viability analyses were performed on the parental MSCs. The sEV secretion rate to the supernatant was evaluated by nanoparticle tracking analysis (NTA). Further isolation of larger batches by ultracentrifugation allowed the purification of sEV and their characterization through NTA & flow cytometry. Functional tests included in vitro cell uptake assays and in vivo biodistribution studies in mice.

**Results**: No changes of the MSCs phenotype and differentiation capacity was observed, in all three culture conditions. Interestingly, Oxium™EXO allowed a higher cell viability (16% increase) evaluated at 6 days post‐ production and without medium supplementation. In addition, we observed a 3‐fold increase of total particle secretion to the supernatant and a 4‐fold increase in the size fraction between 50 to 200nm, in comparison with the DMEM or the commercial medium. The isolated‐sEV characterization showed the presence of CD63, CD9 and CD81 on sEV isolated from all the different conditions. All three sEV production conditions displayed similar cell uptake in vitro and biodistribution and organ accumulation patterns in mice.

**Summary/Conclusion**: Oxium™EXO allowed a significant increase of particle secretion rates while conserving the classic sEV functional properties of internalization into acceptor target cells and biodistribution in vivo. These results support the amount and quality requirement of sEV for the development of efficient cell‐free therapies.

### PS11.04 Acidification of blood plasma facilitates the isolation and analysis of extracellular vesicles

Danilo Mladenović, Tallinn University / Hansabiomed Life Sciences

Delaram Khamari, Semmelweis University

Agnes Kittel, Hungarian Academy of Sciences, Institute of Experimental Medicine

Kadi‐Liis Veiman, HansaBioMed Life Sciences OÜ, Mäealuse 2/1, 12618 Tallinn, Estonia

Francesca Loria, HansaBioMed Life Sciences OÜ, Mäealuse 2/1, 12618 Tallinn, Estonia

Mattia Criscuoli, Exosomics

Antonio Chiesi, Exosomics / HansaBioMed Life Sciences

Kairi Koort, Tallinn University, School of Natural Sciences and Health

Edit Buzás, Semmelweis University, Department of Genetics, Cell‐ and Immunobiology

Nataša Zarovni,Exosomics / HansaBioMed Life Sciences OÜ

**Introduction**: Human blood plasma takes the spotlight as one of the main body fluids in clinical biochemistry and research due to its availability, historically extensive characterization as a biomarker source and recently, because it streams plethora of extracellular vesicles (EVs) originating from different cell sources, that harbour immense diagnostic potential. However, presence of many contaminants that outnumber EVs by orders of magnitude, aggravates the isolation and detection. Here, we investigated the effect of acidification on enrichment, quality and downstream analysis of vesicles from blood plasma.

**Methods**: Plasma preclearing and EV isolation: acidification, differential centrifugation, size exclusion chromatography (SEC).

Quantification and characterization of EVs: ELISA, Nanoparticle Tracking Analysis (NTA), Cytoflex, BCA, Western Blot, WES system, TEM

**Results**: Our results show significant enrichment of EV‐associated markers upon acidification, by promoting aggregation and precipitation from whole plasma samples. Furthermore, differential centrifugation was able to separate vesicles from other components and particles with higher efficiency in acidic environment, from both whole and SEC‐purified plasma.

**Summary/Conclusion**: Acidification prompts a simple straightforward approach for improved yield and detection of relevant plasma EV markers, with high potential impact on biomarker discovery and diagnostic development.

### PS11.05 Comparison of different tomato plant explants for the production of extracellular vesicles in suspension culture

Maneea Moubarak, EVs&MS Laboratory, Institute of Biosciences and BioResources, National Research Council of Italy

Pasquale Chiaiese, Department of Agricultural Sciences, University of Federico ll

Veronika Kralj‐Iglič, Laboratory of Clinical Biophysics, Faculty of Health Sciences, University of Ljubljana

Darja Bozic, MSc, Laboratory of Clinical Biophysics, Faculty of Health Sciences, University of Ljubljana

Gabriella Pocsfalvi, EVs&MS Laboratory, Institute of Biosciences and BioResources, National Research Council of Italy

**Introduction**: Comparing to mammalian systems, the biogenesis and biological function of plant extracellular vesicles (EVs) have been less investigated. Only recently has been shown that tomato roots release EVs into the environment. Plant cell suspension culture is grown in a relatively simple mineral containing liquid medium under controlled conditions and thus may be suitable for the production of EVs. Tomato suspension cultures have been used for the advancement of genetic character, studying responses to abiotic stress factors and for biopharming of secondary metabolites, like carotenoids but not for EV production. Here, in the framework of the European greenEV project, we have established tomato (Solanum lycopersicum L) cell suspension cultures to evaluate the feasibility of the production of EVs using different tomato explants.

**Methods**: Seeds of two tomato cv M82 and Microtom were sowed in plant tissue culture basal medium. Leaves, stem and roots explants were collected and cultivated in a medium containing high amount of a synthetic auxin under controlled conditions in dark. Friable callus from each type of explants were inoculated in a liquid medium to establish fine cell suspension cultures. Cell subcultures' supernatants were collected for the evaluation of rate of cells growth. EVs were isolated by gradient ultracentrifugation (gUC). Physical, morphological and molecular characteristics of the different fractions were analyzed by DLS, SEM, BCA assay and SDS‐PAGE. Densities of the EV containing fractions were determined by iodixanol gUC. EV yields were calculated based on the protein concentrations and vesicle numbers in each isolates.

**Results**: We have successfully established six tomato suspension cultures. All the suspension cultures studied yielded high amount of vesicles, i.e. from 94 to 233 pg of proteins per cell. Our analysis showed that root suspension cultures from Microtom variant has the highest yields of EVs (233 pg protein/cell) followed by the leaves suspension culture of M82 (146 pg protein/cells). gUC resulted in two visible bands (at low and high densities) between 1.098 and 1.117 g/mL densities. Protein profiles of the root, stem and leaves derived EVs were similar on the SDS‐PAGE. Vesicle morphology were visualized by SEM. The two cultivar showed similar SDS‐PAGE profiles.

**Summary/Conclusion**: We have successfully set up six batch suspension cultures for two tomato varieties to produce between 30–136 mL culture supernatant and demonstrated that they contain EVs. Our results show that cell suspension culture could be a promising source of EVs generated by plant cells.

### PS11.06 Enrichment of plasma in platelets and extracellular vesicles by centrifugation

Darja Bozic, MSc, Laboratory of Clinical Biophysics, Faculty of Health Sciences, University of Ljubljana

Domen Vozel, University Medical Centre Ljubljana, Department of Otorhinolaryngology and Cervicofacial Surgery

Matej Hocevar, Department of Physics and Chemistry of Materials, Institute of Metals and Technology

Matic Kisovec, Department of Molecular Biology and Nanobiotechnology, National Institute of Chemistry

Manca Pajnic, University of Ljubljana, Faculty of Health Sciences, Laboratory of Clinical Biophysics

Ljubisa Paden, University of Ljubljana, Faculty of Health Sciences, Laboratory of Clinical Biophysics

Marko Jeran, University of Ljubljana, Faculty of Health Sciences, Laboratory of Clinical Biophysics

Apolonija Bedina Zavec, National institute of Chemistry

Marjetka Podobnik, National Institute of Chemistry, Department of Molecular Biology and Nanobiotechnology

Saba Battelino,University Medical Centre Ljubljana, Department of Otorhinolaryngology and Cervicofacial Surgery

Ales Iglic, University of Ljubljana, Faculty of Electrical Engineering, Laboratory of Physics

Veronika Kralj‐IgličLaboratory of Clinical Biophysics, Faculty of Health Sciences, University of Ljubljana

**Introduction**: Platelet and extracellular vesicle‐rich plasma (PVRP) is a product, prepared from peripheral venous blood, which was proved to have beneficial immune, hemostatic, and regenerative effects. Extracellular vesicles (EVs) are thought to be important mediators of the regenerative effects of PVRP. Motivated by a goal to optimize preparation of plasma for treatment of chronic ear wounds, we studied the effects of centrifugation on blood samples from 53 blood donors.

**Methods**: The study was aproved by the National Medical Ethics Committee, Republic of Slovenia (No. 0120–146 / 2019/5). PVRP was prepared by two consecutive centrifuge spins (spin 1: to separate plasma from erythrocytes and spin 2: to concentrate platelets and EVs in plasma). In both spins the centrifugal pull was under 1000g. The effect of centrifugation on blood cell vesiculation was studied also by a graded centrifugation pull (up to 100.000g) experiment. Concentration of blood cells and EVs was measured by flow cytometry. Samples were observed by scanning electron microscopy and cryo transmission electron microscopy. Standard blood parameters were assessed in a clinical laboratory. A mathematical model was constructed to describe the distribution of blood cells and EVs in samples during the process.

**Results**: We observed up to three‐fold enrichment of platelets and of EVs in plasma collected after spin 1. This was explained by a flux of plasma in the direction opposite to the direction of the centrifugal force that was caused by movement of erythrocytes (i.e. their sedimentation). Enrichment of platelets and of EVs in plasma collected after spin 2 depended on the distance of the sample from the rotor axis (R = ‐0.49, p = 0.01). Prolonged handling time decreased the concentration of platelets and increased the concentration of EVs in PVRP, indicating blood cell fragmentation during processing of the samples. This was supported by the graded centrifugation experiment which showed increasing portion of EVs with centrifugation pull and degradation of material in centrifugation at 100.000g.

**Summary/Conclusion**: Movement of EVs during centrifugation importantly depends on the movement of blood cells. EVs are created during processing of the samples. Agreement of model predictions with observed concentrations of platelets and of EVs in PVRP indicates a possibility of individualized adjustment of centrifugation time and speed to optimize platelet and EV yield in PVRP preparation.

### PS11.07 Isolation, characterization, and functionality of plant‐derived extracellular vesicles

Mart Toots, HansaBioMed Life Sciences OU

Kadi‐Liis Veiman, HansaBioMed Life Sciences OÜ, Mäealuse 2/1, 12618 Tallinn, Estonia

Kairi Koort, Tallinn University, School of Natural Sciences and Health

Paolo Guazzi, HansaBioMed Life Sciences OU

**Introduction**: While mammalian extracellular vesicles (EVs) have been heavily studied over the last decades, much less is known about plant‐derived EVs (P‐EVs). This work aims to describe the improved methods for isolation, characterization and initial analyses of P‐EVs.

**Methods**: TFF‐based ultiafiltration, size‐exclusion chromatography (SEC), NTA, DLS, ELISA and ELISA‐like lectin based characterization assays.

**Results**: Our latest research efforts have led to the development of robust, scalable and cost‐effective isolation methods adapted for P‐EV extraction from Seaberry, Potato and Garlic. Extensive physical and biochemical characterization of P‐EVs indicates that P‐EVs are distinct from their mammalian counterparts in terms of size and surface molecule composition. Our initial functionality analyses of P‐EVs using mammalian cell models shows that these vesicles are readily up‐taken by the cells, and they modify intracellular pathways related to oxidative stress. Moreover, P‐EVs seem to differentially modify the growth of different bacterial strains isolated from Estonian environment.

**Summary/Conclusion**: The initial intriguing data from this research project suggests that P‐EVs might have yet unexplored translational applications in many relevant areas such as cosmetics or epithelial regeneration. Moreover, P‐EVs might play an important role in modulating plant's response to changing environment conditions or pathogen invasion.

### PS11.08 Efficient depletion of Hepatitis B virus from plasma derived extracellular vesicles

Stephanie Jung, University of Bonn

Karolin Fiona Kirsten Jacobs, TU München

Mikhail Shein, TU München

Anne Schütz, TU München

Fabian Mohr, IBA Lifesciences

Herbert Stadler, IBA Lifesciences

Daniela Stadler, TU München

Aaron Lucko, TU München

Sebastian Altstetter, TU München

Li Deng,TU München

Ulrike Protzer, TU München

**Introduction**: Extracellular vesicles (EVs) play a fundamental role in viral infections by shuttling viral components, mediating immune responses, and likely the spread of the virus. To fully assess the clinical and diagnostic relevance for the development of new treatment options targeting EV, further research examining specific EV‐mediated effects on viral infections is required. Because of the similarities in size, density, membrane composition, and mode of biogenesis of EVs and virions there are no standardized protocols for virus‐removal from EV preparations. Additionally, EV samples must be devoid of antibody contaminations for functional studies. Consequently, the study of EVs in virology needs reliable and effective protocols to purify EVs and remove contaminating viral particles.

**Methods**: Here, we established a protocol for EV purification from HBV‐containing plasma by a combination of size‐exclusion chromatography and affinity‐based purification.

**Results**: After purification, EV samples were free of virus‐sized particles, HBV surface antigen, HBV core antigen, antibodies or infectious material. Viral genomic contamination was also decreased following purification.

**Summary/Conclusion**: In summary, we established a fast, reproducible, and robust approach for the removal of HBV from EV preparations. Looking forward to the point of purifying EVs from clinical samples, this method should enable studies shedding light on the underlying mechanisms of EVs in viral infections and their diagnostic and prognostic potential.

### PS11.09 Pre‐processing of bovine milk to clear casein micelles can influence the colloidal and functional properties of milk extracellular vesicles

Martijn van Herwijnen, Department of Biomolecular Health Sciences, Faculty of Veterinary Medicine, Utrecht University, The Netherlands.

Marije Kleinjan, Department of Biomolecular Health Sciences, Utrecht University, The Netherlands

Anna Carnerup, Division of Physical Chemistry, Lund University, Sweden

Tommy Nylander, Division of Physical Chemistry, Lund University, Sweden

Andrea Ridolfi, Dipartimento di Chimica “Ugo Schiff”, Università degli Studi di Firenze, 50019 Firenze, Italy

Marco Brucale, Consiglio Nazionale delle Ricerche, Istituto per lo Studio dei Materiali Nanostrutturati, 40129 Bologna, Italy

Francesco Valle, Consiglio Nazionale delle Ricerche, Istituto per lo Studio dei Materiali Nanostrutturati, 40129 Bologna, Italy

Lucrezia Caselli, Dipartimento di Chimica “Ugo Schiff”, Università degli Studi di Firenze, 50019 Firenze, Italy

Lucia Paolini, Dipartimento di Medicina Molecolare e Traslazionale, Università degli Studi di Brescia, 25123 Brescia, Italy

Paolo Bergese,Dipartimento di Medicina Molecolare e Traslazionale, Università degli Studi di Brescia, 25123 Brescia, Italy

Marca H.M. H.M. Wauben, Department of Biomolecular Health Sciences, Utrecht University, The Netherlands

**Introduction**: Although bovine milk is rich in extracellular vesicles (EVs), other abundant macromolecular complexes, like casein micelles, hamper the isolation of pure EVs. Pre‐clearing milk by precipitation or disruption of caseins prior to EV isolation overcomes this issue. However, the impact of pre‐clearing on EV integrity and function is poorly investigated. We compared three different compounds used to reduce casein micelles and comprehensively analyzed their effect on subsequently isolated EVs.

**Methods**: Raw bovine milk was subjected to differential centrifugation after which caseins were either cleared by precipitation caused by acidification with hydrochloric acid (HCl), or after disruption by adding EDTA or sodium citrate. EVs were further purified using density gradient floatation and Size Exclusion Chromatography (SEC). EV purity was assessed by Augmented COlorimetric NANoplasmonic (CONAN); presence of EV markers by Western blotting (WB); morphology and size by Cryo‐Electron Microscopy, Atomic Force Microscopy (AFM) and Dynamic Light Scattering (DLS). Additionally, colloidal properties including Zeta potential, membrane stiffness and adhesion to lipid surfaces were determined. Functionality of EVs was analyzed in an in vitro T cell modulation assay.

**Results**: Clearing casein micelles from bovine milk prior to density gradient separation and SEC greatly enhanced the purity of the milk EV samples. However, precipitation of caseins by acidification influenced lipid surface interactions and in vitro functionality, while EDTA treatment influenced size and membrane stiffness. Only sodium citrate treatment did not influence the evaluated EV properties.

**Summary/Conclusion**: Using a comprehensive analysis, we were able to show that pre‐processing of bovine milk can greatly enhance purity of milk EV samples, but can also influence the functionality and colloidal properties of milk EVs. Based on our results we recommend the use of sodium citrate for the clearance of caseins in bovine milk.

### PS11.10 A Bioinformatics approach to identify the cellular origin and targets of human milk extracellular vesicles

Martijn van Herwijnen, Department of Biomolecular Health Sciences, Faculty of Veterinary Medicine, Utrecht University, The Netherlands.

Marca H.M. H.M. Wauben, Department of Biomolecular Health Sciences, Utrecht University, The Netherlands

**Introduction**: Breast milk is a highly complex and dynamic biological fluid that contains extracellular vesicles (EVs) that transfer tailor‐made messages to support the developing gastro‐intestinal tract and immune system of the new born. Milk EVs are heterogenous in size and compositions and potentially produced by many different cell types that are locally present in the mammary tissue, or in breast milk. However, it has remained elusive which maternal cells are the dominant producers of milk EVs and which cells are their potential targets.

**Methods**: For bioinformatic analysis we combined 35 previously published studies to determine the full human milk EV proteome. We used FunRich for GO‐analysis for site of expression analysis and the CellTalkDB database to identify receptor‐ligand matches for milk EV proteins and their cellular targets.

**Results**: We found that the full human milk EV proteome consists of 2069 unique proteins. Our analysis showed that not mammary epithelial cells are the likely producers of milk EVs, but that mostly immune cells link to the EV proteome and especially antigen‐presenting cells and T cells were highly represented. By identifying receptor‐ligand matches between milk EV membrane proteins and their corresponding receptors on target cells we revealed that the cellular targets of milk EVs are not only epithelial cells lining the infants gastro‐intestinal tract, but also include multiple immune cells, including B cells and neutrophils.

**Summary/Conclusion**: These findings are important for further functional analysis on milk EV subsets and their specific target cells in order to understand how maternal milk EVs are able to influence and shape the infant's early development.

### PS11.11 Bulk nano‐sized vesicles from tomato fruit show discrete populations in gradient ultracentrifugation and dose‐dependent pro‐ and anti‐inflammatory effects on THP‐1 monocyte cell line

Ramila Mammadova, EVs&MS Laboratory, Institute of Biosciences and BioResources, National Research Council of Italy

Ramesh Bokka, EVs&MS Laboratory, Institute of Biosciences and BioResources, National Research Council of Italy

Immacolata Fiume, EVs&MS Laboratory, Institute of Biosciences and BioResources, National Research Council of Italy

Michele Guescini, University of Urbino Carlo Bo

Serena Maggio, University of Urbino

Veronika Kralj‐Iglič, Laboratory of Clinical Biophysics, Faculty of Health Sciences, University of Ljubljana

Darja Božič, Faculty of Health Sciences, Laboratory of Clinical Biophysics, University of Ljubljana

Matej Hočevar, Department of Physics and Chemistry of Materials, Institute of Metals and Technology

Matic Kisovec,Department of Molecular Biology and Nanobiotechnology, National Institute of Chemistry

Marjetka Podobnik, National Institute of Chemistry, Department of Molecular Biology and Nanobiotechnology

Gabriella PocsfalviEVs&MS Laboratory, Institute of Biosciences and BioResources, National Research Council of Italy

**Introduction**: Nano‐sized membrane bound vesicles can be isolated from a variety of plants, including tomato (S. lycopersicum L). Due to their advantages, such as stability, low toxicity, immunomodulatory, and regenerative properties and the fact that they can be produced from an economically sustainable green resource, they are highly‐promising systems in future therapeutic and nutraceutical applications.

**Methods**: Bulk nanovesicles were isolated from tomato fruit by differential ultracentrifugation (dUC). The complex intra and extracellular vesicle populations were separated using discrete sucrose or iodixanol density gradient ultracentrifugation (gUC). Fractions were collected and characterized by their physical and molecular properties, such as density, size‐distribution, vesicle and protein concentrations, SDS‐PAGE profiles, as well as by scanning electron microscopy (SEM) and cryo‐transmission electron microscopy (cryo‐TEM). To evaluate the cytotoxicity of the visible fractions, MTT assay using THP‐1 monocyte cell line at increasing vesicle concentration (from 5 to 100 ug/mL) for 24h and 48h was performed. Inflammatory effect of the vesicles were tested by RT‐qPCR using IL‐6 and CxCl10 pro‐inflammatory and IL‐10 and CD‐163 anti‐inflammatory mRNA markers.

**Results**: We have isolated tomato fruit‐derived vesicles with a high yield using dUC. The bulk vesicle isolates were separated by gUC based on their buoyant density. Two visible bands were obtained at densities 1.08 and 1.13 g/mL. Depending on the fruit quality, vesicle quantity in the two bands was variable and sometimes a third band could also be observed. CyroTEM analysis confirmed the presence of membrane enclosed vesicles with different sizes and forms in both visible bands. SEM and TEM revealed presence of vesicles of heterogenous morphology (50‐1000nm) and other amorphous material. We have found that tomato‐derived NVs were slightly toxic only at high concentrations. Moreover, both fractions stimulates pro‐ and anti‐inflammatory cytokines in a concentration‐dependent manner.

**Summary/Conclusion**: Tomato NVs were isolated with a high yield by dUC and sucrose and iodixanol gUC was applied to separate the EVs into 2–3 visible bands within the EV density range. SEM and cryo‐TEM analysis proved the presence of vesicles in the collected fractions. Due to their anti‐inflammatory activities, tomato‐derived NVs could be explored as potential therapeutic agents or therapeutic vehicles.

### PS11.12 Extracellular Vesicles Analysis in the COVID‐19 Era: Insights on the Effects of Serum Inactivation Protocols Towards Downstream Isolation and Analysis

Alessandro Gori, National Research Council of Italy

Roberto Frigerio, National Research Council of Italy

Angelo Musicò, National Research Council of Italy

Marco Brucale, Consiglio Nazionale delle Ricerche, Istituto per lo Studio dei Materiali Nanostrutturati, 40129 Bologna, Italy

Andrea Ridolfi, Dipartimento di Chimica “Ugo Schiff”, Università degli Studi di Firenze, 50019 Firenze, Italy

Silvia Galbiati, IRCCS San Raffaele Scientific Institute

Riccardo Vago, IRCCS San Raffaele Scientific Institute

Greta Bergamaschi, National Research Council of Italy

Marcella Chiari, National Research Council of Italy ‐ Institute of Chemical Sciences and Technologies (CNR ‐ SCITEC)

Francesco Valle,Consiglio Nazionale delle Ricerche, Istituto per lo Studio dei Materiali Nanostrutturati, 40129 Bologna, Italy

Marina Cretich, CNR‐SCITEC

**Introduction**: Since the outbreak of SARS‐CoV‐2 crisis, the handling of biological samples from known or suspected COVID‐19 positive individuals demanded the use of inactivation protocols aimed at ensuring laboratory operators safety. As such, these routine procedures should also apply to samples intended for Extracelluar Vesicles (EVs) analysis but, currently, there are no standardized practices. Assessing the impact of inactivating pre‐treatments is therefore of pivotal importance, given the well‐known variability introduced by different pre‐analytical steps on downstream EVs isolation and analysis. Common guidelines on inactivation protocols tailored to best address EVs‐specific requirements will be likely needed among the EVs community, yet deep investigations in this direction haven't been reported so far. We here provide preliminary insights on this relevant topic by comparing solvent/detergent treatment vs. heat inactivation.

**Methods**: pre‐COVID serum samples from healthy donors were processed to mimic heat inactivation (56°C, 30 min) and solvent/detergent treatment (S/D, 10mg/mL Tween 80, 3 mg/mL TNBP). The subsequent analysis entailed the evaluation of EVs recovery and purity along with biochemical and biophysical profiling by means of Nanoparticle Tracking Analysis, Western Blotting, Atomic Force Microscopy, Transmission Electron Microscopy, miRNA content (digital droplet PCR) and tetraspanin assessment by antibody microarrays.

**Results**: Our data showed a marked increase of EVs‐related contaminants following serum heat inactivation. This was demonstrated by higher particle counting (NTA), decresed ratios between EV‐markers and lipoparticle contaminants (WB), different nanomechanic behaviour (AFM), decreased levels of tetraspanin‐responsive particles (EV‐arrays) and higher levels of miRNAs due to RNA‐binding protein co‐isolation (ddPCR). On the contrary, S/D treatment led to no remarkable differences from untreated controls when small EVs (< 150nm range) are considered, yet a depletion of larger vesicular entities was detected.

**Summary/Conclusion**: Far from being conclusive, this work represents a first step towards the identification of optimal biosamples inactivation protocols targeted to EVs analysis. Our data suggest that the use of solvent/detergent addition could be seen a as a preferable virus deactivating method, yet the virus inactivation procedure should be tailored considering the downstream analysis to be undertaken, and further work will be needed in this direction to identify the best possible practices.

### PS11.13 Flexible Immunomagnetic Isolation for Extracellular Vesicle Subtypes Targeting Surface Markers such as CD9, CD63, CD81, EpCAM, and CD45

Mandy Chan, MSc, STEMCELL Technologies

Adil Kassam, PhD, PEng, STEMCELL Technologies Inc.

Allen C. Eaves, OBC, MD, PhD, FRCPC, STEMCELL Technologies Inc.

Sharon A. Louis, PhD, STEMCELL Technologies Inc.

Karina L. McQueen, PhD, STEMCELL Technologies Inc.

**Introduction**: Extracellular vesicles (EVs) have shown promise as a disease biomarker, and their surface markers can reflect their cell of origin. Bulk EV isolation and analysis often fails to detect disease‐specific EVs due to their low frequency. Immunomagnetic isolation based on EV‐ associated surface markers enriches for EV subtypes and increases the sensitivity of downstream analyses. We have developed two immunomagnetic (EasySep™) EV isolation methods that isolate EV subtypes from biofluids based on either CD9, CD63, CD81 or user‐defined surface markers.

**Methods**: CD9+, CD63+, and CD81+ EVs were targeted directly with specific antibody complexes and magnetic particles. EpCAM+ or CD45+ EVs were first labeled with biotinylated‐EpCAM or PE‐conjugated CD45 antibodies, respectively, then bound to magnetic particles using biotin‐ or PE‐specific antibody complexes. The desired, magnetically labeled EVs were separated from unwanted EVs using an EasySep™ magnet. Magnetic particles were released from EVs using a specialized release buffer in an optional step. Bulk EVs were isolated by ultracentrifugation. EV markers were analyzed by western blot, and EV integrity was assessed by RNase digestion followed by RT‐qPCR.

**Results**: EpCAM+ EVs were captured from conditioned cell culture medium containing 2 × 10^8 ‐ 2 × 10^10 EVs/mL (n = 1) and from human plasma spiked with 2 × 10^8 ‐ 2 × 10^9 EVs/mL (n = 2). Over 80% of EVs could be removed from the particles after isolation (n = 2). EpCAM+ EVs were intact and protected miRNA cargos from RNase digestion. Immunomagnetic EasySep™ isolation targeting CD9+, CD63+, CD81+, CD45+, or EpCAM+ EVs resulted in higher target marker expression than bulk EV isolation (n = 3).

**Summary/Conclusion**: Immunomagnetic isolation (EasySep™) can be used to directly (e.g. CD9, CD63, or CD81) or indirectly (e.g. EpCAM or CD45) isolate virtually any EV subtype based on surface marker expression. This will enable flexible isolation of disease‐related EVs and facilitate new strategies for biomarker discovery.

### PS11.14 An extracellular vesicle separation and characterisation study to evaluate how representative of skim milk is infant milk formula?

Anindya Mukhopadhya, School of Pharmacy and Pharmaceutical Sciences & Trinity Biomedical Sciences Institute, Trinity College Dublin and Trinity St. James's Cancer Institute, Dublin 2, Ireland

Jessie Santoro, School of Pharmacy and Pharmaceutical Sciences & Trinity Biomedical Sciences Institute, Trinity College Dublin, Dublin 2, Ireland

Lorraine O'Driscoll, School of Pharmacy and Pharmaceutical Sciences, Trinity Biomedical Sciences Institute, Trinity St. James's Cancer Institute & TRAIN‐EV Marie Skłodowska‐Curie Action‐Innovative Training Network, train‐ev.eu

**Introduction**: Many infants are fed infant milk formula (IMF). However, IMF production from skim milk (SM) involves harsh treatment. So, we hypothesised that the quantity and/or quality of extracellular vesicles (EVs) in IMF may be reduced. Thus, firstly, we aimed to optimise separation of EVs from IMF and SM and, secondly, we aimed to compare the EV isolates from these two sources.

**Methods**: Prior to EV isolation, abundant casein micelles of similar sizes to EVs were removed by treating milk samples with either acetic acid or hydrochloric acid. Samples progressed to differential ultracentrifugation (DUC) or gradient ultracentrifugation (GUC). EV characterisation included BCA, SDS‐PAGE, nanoparticle tracking (NTA), electron microscopy (TEM), RNA analysis, immunoblotting, and imaging flow cytometry (IFCM).

**Results**: Reduced EV concentrations were found in IMF. SM‐derived EVs were intact, while IMF contained disrupted EV‐like structures. EV biomarkers were more abundant with isolates from SM, indicating EV proteins in IMF are compromised.

**Summary/Conclusion**: Altogether, a suitable method combining acid pre‐treatment with GUC for EV separation from milk products was developed. EVs appear to be substantially compromised in IMF compared to SM.

### PS11.15 Can Extracellular Vesicles release truly be pharmacologically inhibited?

Mariadelva Catalano, Trinity College Dublin

Niamh McNamee, Trinity College Dublin

Lorraine O'Driscoll, School of Pharmacy and Pharmaceutical Sciences, Trinity Biomedical Sciences Institute, Trinity St. James's Cancer Institute & TRAIN‐EV Marie Skłodowska‐Curie Action‐Innovative Training Network, train‐ev.eu

**Introduction**: We previously reported extracellular vesicles (EVs) to be causally involved in transmitting phenotypic traits of aggressiveness in prostate cancer. We hypothesised that if we could inhibit EV released, we could block many of these serious problems. Thus, this study aimed to evaluate compounds that others have reported to block EV release. Specifically, we selected calpeptin and Y2763 (reported to inhibit EVs budding from cell membrane) and manumycin A and GW4869 (reported to inhibit EVs deriving from MVBs).

**Methods**: To ensure that any effects observed were not simply due to cell death induced by the compounds, suitable concentrations that were non‐toxic to cells were first determined by cytotoxicity assay and flow cytometry (FC). Conditioned medium (CM) was then collected from 2 prostate cancer drug‐resistant variants (PC3RD, DU145RD) cells after incubation with or without the 4 compounds. Any EVs that continuing to be released following exposure to these compounds, and from controls, were separated from the CM by orthogonal tangential flow filtration and Optiprep density gradient and characterised in line with MISEV2018 guidelines.

**Results**: When working with concentrations of calpeptin, Y2763, manumycin A or GW4869, that did not induce significant cell death, none of the 4 inhibitors (alone or in combination) significantly inhibited EV release from any of the 4 prostate cancer cell line variants, based on nanoparticle tracking analysis, immunoblots for positive and negative EV markers, and transmission electron microscopy analysis.

**Summary/Conclusion**: When used at non‐toxic levels, previously reported EV inhibitors proved not to significantly inhibit EV release from a range of prostate cancer cell line variants. This highlights the importance of considering off‐target effects in efforts to block EV release.

## PS12 Cancer Metastasis and Tumor Angiogenesis

Chair: Olivier De Wever, Laboratory of Experimental Cancer Research, Department of Human Structure and Repair, Ghent University, Ghent, Belgium

Chair: Zoltán Wiener, Semmelweis University, Department of Genetics, Cell and Immunobiology, Budapest, Hungary

### PS12.03 Exosomal CD147 has a role in promoting tumorigenesis via paracrine signaling in pediatric rhabdomyosarcoma

Assil Fahs, Department of Biology, Faculty of Science II, EDST, Lebanese University, Fanar, Lebanon. Department of Anatomy, Cell Biology and Physiology, American University of Beirut, Beirut, Lebanon

Farah Ramadan, Department of Biology, Faculty of Science II, EDST, Lebanese University, Fanar, Lebanon.

Farah Ghamloush, Department of Anatomy, Cell Biology and Physiology, American University of Beirut, Beirut, Lebanon

Bassam Badran, Department of Cancer Biology and Molecular Immunology, Faculty of Science I, EDST, Lebanese University, Hadath, Lebanon.

Nader Hussein, Department of Cancer Biology and Molecular Immunology, Faculty of Science I, EDST, Lebanese University, Hadath, Lebanon.

Raya Saab, Department of Anatomy, Cell Biology and Physiology, American University of Beirut, Beirut, Lebanon. Department of Pediatrics and Adolescent Medicine, Children's Cancer Institute, American University of Beirut, Beirut, Lebanon

Sandra E. Ghayad, Department of Biology, Faculty of Science II, EDST, Lebanese University, Fanar, Lebanon.

**Introduction**: Rhabdomyosarcoma (RMS) is an aggressive childhood soft tissue tumor, with two distinct histologic subtypes: alveolar (ARMS) and embryonal (ERMS). Exosomes are small secreted microvesicles shown to mediate paracrine signaling by delivering intact and functional proteins and miRNA to recipient cells altering their cellular environment. In a previous study, we have identified 80 proteins by LC‐MS/MS analysis that are common to both ERMS and ARMS subtypes. These proteins include exosomal markers, but also proteins involved in cell signaling and cancer such as EMMPRIN/CD147/Basigin. The expression of CD147 was found to be increased in various tumor cells and was tightly correlated with poor prognosis in several types of cancer.

**Methods**: CD147 expression was downregulated in ERMS and ARMS cells using a shRNA approach. The effects of CD147 on paracrine signaling were investigated through RMS exosomes, and its contribution to invasion and metastatic ability of RMS cells by treating normal human BJ fibroblasts with corresponding exosomes. Immunohistochemistry was performed to detect the expression of CD147 in a panel of human RMS tumor tissue.

**Results**: By downregulating CD147 expression in ERMS and ARMS cells, we uncovered a potentiating effect of CD147 on RMS invasive properties, as evidenced by the results of scratch and anchorage‐independent growth assays in vitro. In accordance with previous results, treatment with RMS‐ derived exosomes resulted in a significant increase in BJ fibroblasts proliferation, migration, and invasion. Upon CD147 knockdown in the RMS cells, there was a significant decrease in proliferation, migration, and invasion in BJs treated with the corresponding exosomes. Furthermore, we found that CD147 protein expression correlates with TNM stage and metastatic tumor properties.

**Summary/Conclusion**: Altogether, our results demonstrate that CD147 can exert pro‐tumorigenic effects of RMS cells through paracrine signaling via exosomes. CD147 is a promising possible target for therapeutic intervention in RMS that should be further studied.

### PS12.04 Evaluation and characterization of extracellular vesicles released by 4T1 mammary adenocarcinoma cells after interaction with macrophages raw 264.7

Vanessa Xavier, Icon plc

Patricia X. Xander, Universidade Federal de São Paulo campus Diadema

Elizabeth Hurtado, Universidade Paulista

**Introduction**: Cancer is generally defined as the abnormal and uncontrolled proliferation of cells by changes in the DNA that leads to the formation of tumors. Breast cancer is the most common type of cancer among women and the second deadliest form of cancer among the female population worldwide, mainly due to the presence of metastases, which characterize the most aggressive type of the disease. Several studies have shown that interactions between tumor cells and macrophages in the tumor microenvironment are relevant to the acquisition of a more aggressive phenotype. In order to understand the mechanisms involved in the acquisition of metastatic phenotype in the development of cancer, tumor cells are known to release extracellular vesicles that can modulate the immune system cell responses by promoting favorable responses for invasion, progression and establishment of new secondary tumors. Objective: Characterize extracellular vesicles (EVs) released by 4T1 mammary adenocarcinoma cells before and after interaction with RAW 264.7 macrophages.

**Methods**: EVs produced by 4T1 cells before and after interaction with macrophages RAW 264.7 were collected and submitted to both physico‐chemical and biological analysis for their characterization and evaluation of their effects under macrophages

**Results**: Scanning electron microscopy analysis and quantification of proteins showed 4T1 cells after contact with macrophages with reduction of EVs production and lower memory capacity when compared with 4T1 cells that did not come into contact with macrophages. The characterization of EVs by nanoparticle tracing analysis (NTA) and by flow cytometry revealed that the EVs produced are on average between 100 and 200 nm with the same number of exosomes and microvesicles. Regarding the biological effect, macrophages treated with EVs, those produced by 4T1 cells prior to interaction with the macrophages around an increase in the phagocytic index as an elimination ability of the phagocytosed material.

**Summary/Conclusion**: Conclusion: Macrophages reduce the migration and invasion capacity of 4T1 breast adenocarcinoma cells. These data suggest that the decrease in the production of EVs by tumor cells may be one of the efficient mechanisms to reduce the formation of secondary tumors or metastases.Therefore, the reduction of the production of EVs by 4T1 cells induced by contact with macrophages may be a promising mechanism for the control of highly aggressive tumors such as breast adenocarcinoma.

### PS12.05 May a pharmacological lipid modulation of parental cells reduce protumogeric signalling of secreted Extracellular Vesicles? In vitro proteomic studies on a human metastatic melanoma cell line

Felice Accattatis, Università Degli Studi di Milano

Sara Mazza, Dipartimento Di scienze Farmacologiche e Biomolecolari ‐ Università Degli Studi di Milano

Agnese Granata, Dipartimento Di scienze Farmacologiche e Biomolecolari ‐ Università Degli Studi di Milano

Elisabetta Vergani, Laboratorio di Immunoterapia dei Tumori Umani IRCCS Istituto Nazionale dei Tumori Milano

Monica Rodolfo, Laboratorio di Immunoterapia dei Tumori Umani IRCCS Istituto Nazionale dei Tumori Milano

Alberto Corsini, Dipartimento Di scienze Farmacologiche e Biomolecolari ‐ Università Degli Studi di Milano

Sara Baroni, Dipartimento Di scienze Farmacologiche e Biomolecolari ‐ Università Degli Studi di Milano

Lidia Merlo, Dipartimento Di scienze Farmacologiche e Biomolecolari ‐ Università Degli Studi di Milano

Lorenzo Arnaboldi, Dipartimento Di scienze Farmacologiche e Biomolecolari ‐ Università Degli Studi di Milano

**Introduction**: Extracellular Vesicles (EVs) are a very attractive pharmacological target, due to their involvement in cell‐cell communication in physiological and pathological conditions. Among lipids, cholesterol and BisMonoacylGlyceroPhosphate (BMP) play a paramount role in EV biogenesis, since their interplay drive endosomes towards the secretory or recycling pathway. Based on these premises, we modulated cholesterol biosynthesis and BMP metabolism of metastatic melanoma cells and evaluated EVs protein content, potentially leading to altered biological functions.

**Methods**: In vitro treatment LM‐16 cells with simvastatin and/or KT182, respectively inhibitors of cholesterol biosynthesis and of BMP degradation, was carried for 3 days with 10% FCS and for 3 days in serum free conditions. 10K (microvesicles) and 100K (exosomes) fractions were isolated by ultracentrifugation and characterized by Nanosight. Quantitative proteomic analysis was performed by mass spectrometry and results analyzed by Ingenuity Pathway Analysis (IPA).

**Results**: Treatment with simvastatin (0.1μM) and KT182 (50nM) alone or in combination did not affect cell proliferation, and EVs size and counts. Proteomics identified 1294 and 1192 proteins respectively for 100K and 10K, showing different significant expression patterns, compared to control, with several proteins up‐ and down regulated. IPA showed that treatments specifically decreased the expression of proteins involved in cellular movement, migration, proliferation and cytoskeleton arrangement, both in 100K and 10K fractions, with typical and striking differences and patterns.

**Summary/Conclusion**: Cholesterol and BMP modulation of melanoma cells dramatically alters the protein content of released EVs, possibly leading to altered EVs functionality, suggesting the potential of lipid modulation in reshaping tumor cell‐released EVs to develop new therapeutic approaches.

### PS12.06 Effect of radiation on prostate derived, β1 integrin positive small Extracellular Vesicles

Vaughn Garcia, Thomas Jefferson University

Rachel M. DeRita, PhD, Thomas Jefferson University

Aejaz Sayeed, Thomas Jefferson University

Shiv Krishn, PhD, Thomas Jefferson University

Peter McCue, Thomas Jefferson University

Adam Dicker, Thomas Jefferson University

Lucia R. R. Languino, Thomas Jefferson University

**Introduction**: EVs are emerging as critical mediators of cell‐to‐cell communication in response to cancer therapy. Our laboratory has previously reported that prostate irradiation of TRAMP mice significantly blocks tumor growth. Also, our results have demonstrated that β1 integrins in circulating tumor‐cell‐derived sEVs are required for stimulation of anchorage‐independent growth.

**Methods**: Here we characterize the circulating sEVs from TRAMP mice and to add the clinical perspective, we analyzed sEVs from the plasma of PrCa patients. We use plasma sEVs isolated using differential ultra‐centrifugation and iodixanol gradient fractionation. We used NTA to analyze sEVs. Mouse pelvises were irradiated using 10 Gy, for 5 consecutive days.

**Results**: Circulating sEVs from the plasma of prostate cancer patients show robust expression of β1 integrins and Src. We also demonstrate a robust expression of β1 integrins and c‐Src in sEVs isolated from TRAMP mice which promotes anchorage‐independent growth of recipient cells. We observe that upon pelvic irradiation of TRAMP mice, the levels of the β1 integrins and c‐Src are reduced in plasma‐derived sEVs. In addition, upon irradiation the size of sEVs is increased from 50–100nms to 70–250nms, whereas the concentration of sEVs is not affected. Similarly, upon irradiation, PC3 cell derived sEVs show profound reduction in β1 and c‐Src/c‐SrcpY416 and in their ability to enhance anchorage‐independent growth and cell migration.

**Summary/Conclusion**: Taken together, we have identified a novel effect of irradiation which has profound implications in suppressing sEV‐mediated pre‐metastatic lesions in cancer patients. These data pave the way to future investigations of circulating EVs from patients who have undergone radiation therapy to evaluate stratification, accuracy of prognosis and efficacy of therapeutic efforts.

### PS12.07 Cancer cell derived extracellular vesicles alter endothelial cell metabolism and stimulate angiogenesis

Joël E.J. Beaumont, Department of Radiotherapy, GROW‐School for Oncology and Developmental Biology, Maastricht University

Marijke I. Zonneveld, Postdoctoral researcher, GROW‐School for Oncology and Developmental Biology, Maastricht University, The Netherlands

tom G.H. keulers, dept. of Radiotherapy

Kasper M.A. Rouschop, Department of Radiotherapy, GROW‐School for Oncology and Developmental Biology, Maastricht University

**Introduction**: Hypoxia is a common feature of solid tumors, associated with poor prognosis, increased metastasis and therapy resistance. Recently, Extracellular Vesicles (EV) have emerged as transmitters of this hypoxic phenotype throughout the tumor environment. We previously demonstrated that hypoxic cancer cells release angiogenic EV which are characterized by the presence of GABARAPL‐1 on their outer membrane. In this study, we aim to further characterize the angiogenic and metabolic effects of these (hypoxic) EV and to evaluate the therapeutic potential of anti‐GABARAPL‐1 antibodies in inhibiting these EV mediated effects.

**Methods**: HT29 cancer cells were cultured under normoxia, moderate hypoxia (0.2% O2) or severe hypoxia (< 0.02% O2) in medium with 5% EV depleted serum. Conditioned medium (CM) was cleared from cells and debris by centrifugation (300g 10’, 2000g 20’, 16.000g 30’) and concentrated with 100kDa filters. EV were isolated using size exclusion chromatography, aliquoted and stored at ‐80°C. To evaluate the angiogenic potential of EV in vitro, migration (scratch assay), proliferation (BrdU‐incorporation) and tube formation of endothelial RF24 cells were measured upon EV stimulation. To evaluate metabolic effects of EV, endothelial glucose uptake and lactate production were measured.

**Results**: Stimulation with normoxic EV, but not hypoxic EV, increased endothelial cell migration. In contrast, both moderate and severe hypoxic EV increased endothelial cell proliferation. Furthermore, EV from cells exposed to severe hypoxia increased endothelial tube formation. Metabolically, normoxic EV reduced endothelial glucose uptake, but caused an increase in lactate production, whereas hypoxic EV did not alter either glucose uptake or lactate production.

**Summary/Conclusion**: Depending on the oxygenation status of cancer cells, their EV have different effects on recipient endothelial cells. Hypoxic cancer cell derived EV stimulate angiogenesis in vitro as demonstrated by increased endothelial cell proliferation and tube formation. Next, we aim to inhibit these effects with antibodies aimed against GABARAPL‐1. Glucose tracing experiments will be performed to investigate how and why cancer cell derived EV alter endothelial cell metabolism.

### PS12.08 Radiation‐Induced Secretion of Extracellular Vesicles Drives Phenotypic Changes within the Tumor Microenvironment

Greg Berumen, Vanderbilt University

Marjan Rafat, Vanderbilt University

**Introduction**: Triple‐negative breast cancer (TNBC) patients experience high rates of recurrence after radiotherapy. The role of intracellular communication in this process is unknown. Recent studies have shown that ionizing radiation (IR) activates several systemic biological responses, which largely depend on interactions between healthy and damaged tissue cells. Therefore, we hypothesized that extracellular vesicle (EV) secretion from irradiated cells alters the normal cell response to promote tumor cell recruitment. This study represents a crucial step toward elucidating how microenvironmental changes caused by IR influence cancer recurrence.

**Methods**: We explored the impact of IR on EV secretion in stromal cells. Mouse 3T3 fibroblasts and 3T3‐L1 pre‐adipocytes were irradiated to a dose of 10 Gy. We used nanoparticle tracking analysis (NTA) and flow cytometry to characterize EV secretion dynamics in control and irradiated cells. Furthermore, we evaluated the ability of irradiated cell‐derived EVs to alter untreated recipient cells by examining murine 4T1 TNBC cell migration and proliferation and visualizing fibroblast cytoskeletal dynamics through fluorescent labeling of F‐actin.

**Results**: Our NTA results indicate that irradiation enhanced vesicular secretion while flow cytometry analysis revealed distinct subpopulations following IR. Additionally, our findings suggest the ability of irradiated cells to induce bystander effects through EVs. Treating non‐irradiated fibroblasts with small EVs from irradiated cells led to cytoskeletal rearrangement. EVs from irradiated normal cells also enhanced the migration and proliferation of TNBC cells.

**Summary/Conclusion**: Our results establish cell‐specific phenotypic changes arising from interactions between irradiated cells and non‐irradiated cells through EVs. This work will further our understanding of EV‐mediated communication patterns that arise after radiotherapy and will lead to profound insights for improving TNBC treatment regimens.

### PS12.09 Extracellular vesicles derived from prostate cancer‐educated osteoclasts regulate osteoblast activity

Takaaki Tamura, Department of Molecular and Cellular Medicine, Tokyo Medical Univercity

Akiko Kogure, PhD, Tokyo medical university

Yusuke Yoshioka, Department of Molecular and Cellular Medicine, Tokyo Medical University

Shinichi Sakamoto, Graduate School of Medicine and School of Medicine, Chiba University

Tomohiko Ichikawa, Graduate School of Medicine and School of Medicine, Chiba University

Takahiro Ochiya, PhD, Department of Molecular and Cellular Medicine, Tokyo Medical University

**Introduction**: Prostate to bone cancer metastases induce mixed lesions containing areas of bone destruction (osteolysis) and formation (osteogenesis) that are directed by osteoclasts and osteoblasts, respectively. Even in osteogenic bone metastases of prostate cancer, osteoclasts are present in the tumor invasion areas and play an essential role in developing bone metastatic lesions. Emerging evidence suggests that osteoclast‐derived Extracellular Vesicles (EVs) regulate osteoblast activity in normal bone homeostasis. However, there is no study investigating the role of EVs derived from osteoclasts educated by prostate cancer cells in the tumor bone microenvironment.

**Methods**: We compared function and RNA profiles of EVs secreted by two types of osteoclasts; osteoclasts differentiated from RAW264.7 cell lines (OC cells) and OC cells co‐cultured with prostate cancer cells (OCP cells). Osteoclast differentiation was induced by incubation of RAW264.7 cells in the presence of RANKL. EVs were purified from cell culture media using ultracentrifugation. To investigate the effect of these EVs on osteoblasts, we added equal amounts of EVs derived from OC cells and OCP cells to mineralizing osteoblasts (MC3T3‐E1). ALP staining was conducted, and the expression levels of osteoblastic marker genes were compared by qPCR. Furthermore, to identify EV‐delivered molecules regulating osteoblast activity, next‐generation sequencing (NGS) was performed to obtain RNA profiles for OC‐derived EVs and OCP‐derived EVs.

**Results**: OCP cell‐derived EVs significantly inhibited osteoblast activity; OCP cell‐derived EVs significantly down‐regulated the expression levels of COL1A1, ALP, and BGLAP, which are stably expressed osteoblast marker genes in MC3T3‐E1. In addition, NGS identified some candidate EV components regulating osteoblast activity.

**Summary/Conclusion**: We report the role of EVs derived from osteoclasts educated by cancer cells for the first time. Osteoclasts activated in the tumor invasion areas may release EVs, inhibiting osteoblast activity, and leading to further bone destruction. We will evaluate the credibility of these findings in vivo experiments. We hope our findings will contribute to elucidate the whole mechanism of bone metastases.

### PS12.10 Secretion of pro‐angiogenic extracellular vesicles during hypoxia is dependent on the autophagy‐related protein GABARAPL1

Tom G.H. keulers, dept. of Radiotherapy

Sten Libregts, Department of Biomolecular Health Sciences, Faculty of Veterinary Medicine, Utrecht University, Utrecht

Kim Savelkouls, Department of Radiotherapy, GROW‐School for Oncology and Developmental Biology, Maastricht University

Hans Duimel, Microscopy CORE Lab, Maastricht Multimodal Molecular Imaging Institute, FHML Division of Nanoscopy, University of Maastricht

Jan Bussink, 3Department of Radiation Oncology, Radboud University Medical Center, Nijmegen, the Netherlands

Marijke I. Zonneveld, Postdoctoral researcher, GROW‐School for Oncology and Developmental Biology, Maastricht University, The Netherlands

Jeroen Demmers, Proteomics Center, Erasmus University Medical Center, Rotterdam, the Netherlands

Carmen Lopez Iglesias, Microscopy CORE Lab, Maastricht Multimodal Molecular Imaging Institute, FHML Division of Nanoscopy, University of Maastricht, the Netherlands.

Marca H.M. H.M. Wauben, Department of Biomolecular Health Sciences, Utrecht University, The Netherlands

Kasper M.A. Rouschop,Department of Radiotherapy, GROW‐School for Oncology and Developmental Biology, Maastricht University

**Introduction**: Hypoxia is a hallmark of solid tumours and is associated with tumour progression and therapy resistance. In response to hypoxia, tumour cells secrete pro‐angiogenic factors to induce blood vessel formation and restore oxygen supply to the tumour. Exosomes and microvesicles, collectively termed Extracellular vesicles (EVs) are emerging as mediators of intercellular communication in the tumour microenvironment. EVs have the ability to reprogram recipient cells by shuttling biological information such as nucleic acids and proteins, which contributes to tumour progression, angiogenesis and metastasis formation.

**Methods**: Ht29 and U87 doxycycline‐inducible GABARAPL1 knockdown cell lines were exposed to hypoxia (16 hours, < 0.02% O2). EVs were isolated by sucrose density gradient isolation, and analyzed by western blot, qNANO or high‐resolution flow cytometry. Angiogenic potential of cells was assessed by tube formation assays. Xenografts were implanted subcutaneously at the lateral flanks of NMRInu/nu mice and tumour size was measured by caliper.

**Results**: In this study we demonstrate that the LC3/ GABARAP protein family member GABARAPL1, is required for endosomal maturation and cargo loading of EVs during hypoxia. Furthermore, we demonstrate that GABARAPL1 is expressed on the surface of EVs released during hypoxic conditions and that these GABARAPL1+EVs have pro‐angiogenic properties. Silencing GABARAPL1 in inducible knockdown models perturbs GABARAPL1+EV secretion and results in decreased growth of xenografted tumours due to decreased vascularisation and enhanced necrosis. Additionally, targeting GABARAPL1 directly after radiotherapy resulted in enhanced tumour regrowth delay, demonstrating the therapeutic potential of these observations. Since GABARAPL1 is expressed on the EV surface, it is accessible for targeting with antibodies. GABARAPL1+EVs elicit pro‐angiogenic responses in vitro by inducing tube‐formation in HUVEC cells. This effect can be fully blocked by GABARAPL1 blocking antibodies.

**Summary/Conclusion**: During hypoxia, GABARAPL1+EVs are released by tumour cells. These EVs have proangiogenic properties and contribute to tumour progression through induction of angiogenesis. GABARAPL1 is expressed on the outside of EVs, and thus accessible for targeting. Since we detected elevated levels of GABARAPL1+EVs in the blood of cancer patients, these findings could open therapeutic opportunities for concurrent therapies to control tumour growth, and is therefore interesting to pursue as therapeutic target

### PS12.11 In vivo‐mimicking 3D cultures secrete distinct extracellular vesicles upon cancer cell invasion

Jens C. Luoto, MSc, Åbo Akademi University

Leila Coelho Rato, MSc, Åbo Akademi University

Sara Bengs, BSc, Åbo Akademi University

Jannica Roininen, Åbo Akademi University

John Eriksson, PhD, Åbo Akademi University

Lea Sistonen, PhD, Åbo Akademi University

Eva Henriksson, PhD, Åbo Akademi University

**Introduction**: Extracellular vesicles (EVs) are important in intercellular communication and mediate local and long‐range signals in cancer metastasis. However, it is currently unknown how the development of the primary tumor and onset of invasion affects the secretion and characteristics of EVs. In this study, we developed an EV production method utilizing in vivo‐mimicking extracellular matrix‐based 3D cultures, and characterized the EVs over the course of invasive development of tumor organoids.

**Methods**: Human prostate cancer PC3 cells were grown in 3D cultures using ECM‐based hydrogel or in standard 2D culture conditions. EVs were isolated with differential centrifugation or high‐resolution iodixanol gradient centrifugation. The isolated EVs were characterized with nanoparticle tracking analyses, electron microscopy, immunoblotting and mass spectrometry (MS).

**Results**: Using the ECM‐based cell culture method combined with proteomic profiling, we show that PC3 human prostate cancer organoids secrete EVs with previously undefined protein cargo, which substantially differs from EV cargo of 2D cultured cells. Intriguingly, an increase in EV amounts and extensive changes in EV protein composition were detected upon invasive transition of the organoids.

**Summary/Conclusion**: Our results demonstrate that the culture conditions and the developmental status of the organoid cultures have a major impact on EV secretion and cargo loading, highlighting the necessity of in vivo‐mimicking conditions for discovery of novel cancer‐derived EV components, which can be linked to cancer progression and developed as biomarkers for different stages of cancer.

### PS12.12 Extracellular vesicles from ocular melanoma have pro‐fibrotic and pro‐angiogenic properties on liver cells

Kelly CK Coutant, Université Laval

Léo Piquet, Département d'ophtalmologie et d'ORL‐CCF, Faculté de médecine, Université Laval, Quebec City, Quebec, Canada; Axe médecine régénératrice, Centre de recherche du CHU de Québec‐Université Laval, Quebec City, Quebec, Canada; Centre de recherche sur le cancer

Andrew Mitchell, Axe médecine régénératrice, Centre de recherche du CHU de Québec‐Université Laval, Quebec City, Quebec, Canada; Centre de recherche sur le cancer de l'Université Laval, Quebec City, Quebec, Canada; Centre de recherche en organogénèse expérimentale de l'Un

Julie Bérubé, Axe médecine régénératrice, Centre de recherche du CHU de Québec‐Université Laval, Quebec City, Quebec, Canada; Centre de recherche sur le cancer de l'Université Laval, Quebec City, Quebec, Canada; Centre de recherche en organogénèse expérimentale de l'Un

François Bordeleau, Département de biologie moléculaire, biochimie médicale et pathologie, Faculté de médecine, Université Laval, Quebec City, Quebec, Canada; Centre de recherche sur le cancer de l'Université Laval, Quebec City, Quebec, Canada; Centre de recherche en organog

Alain Brisson, UMR‐CBMN, CNRS, Université de Bordeaux, Bordeaux, France.

Solange Landreville, Département d'ophtalmologie et d'ORL‐CCF, Faculté de médecine, Université Laval, Quebec City, Quebec, Canada; Axe médecine régénératrice, Centre de recherche du CHU de Québec‐Université Laval, Quebec City, Quebec, Canada; Centre de recherche sur le cancer

**Introduction**: Uveal melanoma (UM) is the most common primary intraocular tumor in adults and arises from the transformation of melanocytes. Half of patients develop hepatic metastases thus reducing their survival. Extracellular vesicles (EVs) are released by cancer cells, which allow oncoproteins or genetic material to be transferred to distant cells in order to modify their microenvironment and promote the spread of cancer. This study aimed to determine how EVs released by UM cells modify the hepatic microenvironment.

**Methods**: EVs were isolated from UM cells and melanocytes by differential centrifugation. Their surface markers, concentration and diameter range were characterized by Western blotting, high‐sensitivity flow cytometry and cryogenic electron microscopy using exosomal or melanomic markers. The mechanisms of internalization of EVs in hepatic stellate cells and endothelial cells were investigated by confocal microscopy using inhibitors of endocytosis pathways. The contractility of stellate cells on more or less stiff matrices and the tubular organization of endothelial cells on Matrigel post‐exposure to melanomic EVs were determined by collagen gel contraction or endothelial tube formation assays. The selective biodistribution of EVs in organs was studied by in vivo fluorescence imaging in immunodeficient mice.

**Results**: The extravesicular fraction of UM cells and melanocytes contained exosomes and microvesicles of heterogeneous diameter positive for Annexin‐5, CD63, CD81 and melanomic markers. The hepatic stellate cells that have internalized the melanomic EVs were more contractile, while the EV‐treated endothelial cells have developed capillary‐like tubular networks faster. Melanomic EVs were mainly accumulated in the liver and lungs of mice.

**Summary/Conclusion**: We have demonstrated that the extravesicular signaling from UM cells activates hepatic stellate cells, which become more contractile. Melanomic EVs have also a proangiogenic potential. The discovery of melanoma‐specific proteins on the surface of the EVs of metastatic patients could lead to the development of new imaging modalities or drug vectors for targeted delivery to liver metastases.

### PS12.13 Extracellular Vesicles associated with Triple‐Negative Breast Cancer Induce Platelet Aggregation

Niamh McNamee, Trinity College Dublin

Laura Rodriguez de la Fuente, Garvan Institute of Medical Research

Maria J Santos‐Martinez, Trinity College Dublin

Lorraine O'Driscoll, School of Pharmacy and Pharmaceutical Sciences, Trinity Biomedical Sciences Institute, Trinity St. James's Cancer Institute & TRAIN‐EV Marie Skłodowska‐Curie Action‐Innovative Training Network, train‐ev.eu

**Introduction**: Cancer patients have an increased risk of developing venous thromboembolism (“blood clots”), with up to 30% dying with 1 month of their development. Some cancer cells are known to induce platelet aggregation, and this interaction is thought to contribute to thrombosis and metastasis of tumour cells. Many researchers have reported on extracellular vesicles (EVs) released from platelets. However, less is known about how cancer EVs may affect platelet function. Here EVs released by triple‐negative breast cancer (TNBC) cell line variants were investigated for their potential interaction with human platelets.

**Methods**: EVs were separated from conditioned media of TNBC Hs578T and Hs578Ts(i)8 cells using orthogonal filtration and ultracentrifugation and were fundamentally characterised by NTA, immunoblots, and TEM. Light transmission aggregometry and optical microscopy were used to evaluate the potential interaction of TNBC cells or their EVs with human platelets from healthy volunteers. Global proteomic analysis was performed on the EVs, by in‐solution digestion and mass spectrometry.

**Results**: Both TNBC cell variants induced platelet aggregation (p < 0.0001). Increased cell concentrations also significantly (p < 0.0001) reduced the time taken for platelet aggregation to occur (lag phase). Exposure of platelets to EVs also resulted in platelet aggregation and in a reduction of the lag phase in a concentration‐dependent manner (p < 0.0001). Proteomics profiling of the EVs’ cargo identified candidate proteins (including EVA1B, RFF2, ELOC) that may be causally involved in platelet aggregation.

**Summary/Conclusion**: TNBC cells and their released EVs induce platelet aggregation. Furthermore, even without cells present, their released EVs induced this undesirable effect. Further investigation is warranted on the EV surface proteins that may play a role in platelet aggregation during this interaction and thus be relevant as therapeutic targets.

### PS12.14 Can the platelet‐derived extracellular vesicles change invasiveness of colorectal cancer cells by transferring the chemokine receptor 4?

Hassan Kassassir, Institute of Medical Biology, Polish Academy of Sciences, 93–232 Lodz, Poland

Izabela Papiewska‐Pająk, Institute of Medical Biology, Polish Academy of Sciences, 93–232 Lodz, Poland

Jakub Kryczka, Institute of Medical Biology, Polish Academy of Sciences, 93–232 Lodz, Poland

Anna M. Kowalska, Institute of Medical Biology, Polish Academy of Sciences, 93–232 Lodz, Poland

**Introduction**: High mortality of patients suffering from colorectal cancer results not only from developing primary tumor but also from distant metastases formed in crucial organs. Platelet‐derived extracellular vesicles (P‐EVs) can carry and transfer proteins and nucleic acid responsible for cell‐to‐cell communication or formation of metastatic niches. Among different proteins transported by P‐EVs, chemokine receptor 4 (CXCR4) may play role since its overexpression was reported in invasive cancers and the CXCR4/SDF1 signaling was proved to be important feature in cancer development. The aim of this study was to identify whether P‐EVs can transfer CXCR4 into colorectal cancer cells (CRCs) on various epithelial‐to‐mesenchymal transition stage and whether P‐EVs‐derived CXCR4 can modulate the invasive potential of host cells.

**Methods**: We used HT29 (epithelial) and SW620 (strongly mesenchymal) colorectal cancer cell lines. P‐EVs were isolated from outdated concentrates of thrombin‐stimulated human blood platelets by ultracentrifugation. Motility of cells fused or not with P‐EVs was measured using wound healing method and transwell migration assay. Moreover, the effect of P‐EVs‐derived CXCR4 on CRCs migration was also verified. Flow cytometry and confocal imaging were applied to identify whether P‐EVs can transfer CXCR4 and platelet integrins into host CRCs. Lastly we used cell‐mediated immunitiy‐deficient mouse in vivo model to study of the development of metastasis after intravenous injection of CRCs fused or not with P‐EVs.

**Results**: We showed that after internalisation into CRCs, P‐EVs increased the migration of HT29 cells but had no significant effect on SW620 cells. We found that activation of CXCR4 receptor by its ligand SDF‐1 stimulated the motility of HT29 but to lesser extent compared to P‐EVs‐induced migration. Blocking of CXCR4 only partly inhibited the migration rate of HT29 cells fused with P‐EVs. Moreover, the number of several receptors but not CXCR4 was increased on the surface of HT29 and SW620 cells after internalization of P‐EVs.

**Summary/Conclusion**: We conclude that the effect of P‐EVs on epithelial non‐invasive colorectal cancer cells is only partly dependent on CXCR4 and is not related with the surface form of this protein since the expression of CXCR4 on CRCs does not change after incorporation of P‐EVs.

### PS12.15 Cancer cell‐derived EVs promote osteoclasts differentiation

Akiko Kogure, PhD, Tokyo medical university

Takahiro Ochiya, PhD, Department of Molecular and Cellular Medicine, Tokyo Medical University

**Introduction**: Osteolytic bone metastasis occurs at particularly high rates in the breast cancer and causes a reduction in the quality of life of patients with cancer. In the bone metastatic site, cancer cells release factors that switch from preosteoclasts to osteoclasts. However, the cellular mechanisms which induce osteoclasts by cancer cells are still not clear. Recent research has demonstrated that extracellular vesicles (EVs) play a vital role in cancer metastasis. In the present study, we focused on breast cancer cell‐derived EVs and investigated their role in osteolytic metastasis.

**Methods**: To investigate the function of breast cancer cell‐derived EVs in osteoclast differentiation, cancer cell‐derived EVs were isolated from several types of breast cancer cell lines (Luminal type, Triple‐negative type, and Her2 positive type). EVs were purified by ultracentrifugation and characterized by Nanosight Tracking Analysis (NTA). The EVs were added to osteoclast precursors (RAW264.7 cells) with or without RANKLs. The osteoclast differentiation was evaluated by Tartrate‐resistant acid phosphatase (TRAP) stain and by measuring the expression level of osteoclast markers using by qRT‐PCR.

**Results**: We found that EVs derived from one of the Her2 positive breast cancer cell line significantly increased TRAP positive cells compared with PBS treatment and other breast cancer cell line derived EVs treatment in the presence of RNAKL. Moreover, the EVs‐derived from Her2 positive breast cancer cells upregulated the expression levels of osteoclast‐related genes such as TRAP, NFATc1, and CTSK genes.

**Summary/Conclusion**: Our results suggest that the EVs derived from Her2 positive breast cancer cell line promote the osteoclast differentiation. We intend to investigate the factors which is responsible for promoting of osteoclast differentiation in the breast cancer derived EVs.

## PS13 EVs in Cancer

Chair: Shang‐Chun Guo, Shanghai Jiao Tong University Affiliated Sixth People's Hospital, China (People's Republic)

Chair: Vincent Hyenne, INSERM / CNRS, France

### PS13.01 Cancer exosome cargo microRNAs supporting distant metastasis

Luhan Li, Nankai University

Shuo Liu, Nankai University

Shijing Yue, Nankai University

**Introduction**: Epithelial‐mesenchymal transition (EMT) of cancer cell has disclosed one part of “seed‐and‐soil” hypothesis. Cancer cell and bone marrow derived factors predispose distant tissues to change microenvironment into a pre‐metastatic niche. However, cancer derived elements educating the context of soil remains a large gap.

**Methods**: Here, we identified cancer derived exosome cargo miRNAs mediating cancer metastasis via sequencing analysis of the samples from pre‐ and post‐surgery of pancreatic cancer (PaCa) patients. Cancer derived exosomes are efficiently targeted lung, liver and spleen by circulation. The functional role of specific miRNAs was evaluated in vivo and in vitro. The target genes of specific miRNAs were screened by online database and validated by in vivo and in vitro. The molecular mechanism of exosome cargo miRNAs mediating cancer metastasis was confirmed by functional assays in vitro.

**Results**: Through comparative small RNA Hiseq, we found that miR‐92a‐3p at the top of miRNA list involved in cancer metastasis. Exosomal miR‐92a‐3p interfered with the expression of DAB2IP, NF2, PTEN, SMAD7 and ZO‐1 that activated angiogenesis, endothelial cell surface adhesive molecules and leakiness of capillary in lung and liver.

**Summary/Conclusion**: Sustaining cancer exosomal miR‐92a‐3p supported reprogramming of target cells in the distant organs. Cancer derived exosome cargo miRNAs altering the phenotype of endothelial cells in the distant tissues. The changed microenvironment is prepared for cancer cells arrival that fill up the gap of soil part.

### PS13.02 Microvesicles as mediators of metastasis formation in colorectal cancer

Suganja Sivaloganathan, University Hospital Muenster

Raquel Blazquez, University Hospital Regensburg

Pukrop Tobias, University Hospital Regensburg

Darius Wlochowitz, Insitute of Medical Bioinformatics, Göttingen

Tim Beißbarth, Institute of medical bioinformatics, Göttingen

Simone König, IZKF Core Unit Proteomics

Linda Groeneweg, Institute of Immunology, Universtiy Hospital Muenster

Noelia Alonso gonzalez, Institute of Immunology, Universtiy Hospital Muenster

Claudia Binder, Department of Hemaotology/Medical Oncology, University Medical Center Göttingen

Matthias Schulz,Dept. of Hematology/Medical Oncology, University Medical Center Göttingen, Göttingen

Kerstin Menck, Dept. of Medicine A, Hematology, Oncology, and Pneumology, University Hospital Münster

Annalen BleckmannDept. of Medicine A, Hematology, Oncology, and Pneumology, University Hospital Münster

**Introduction**: Colorectal cancer (CRC) accounts for one of the most commonly diagnosed cancer type worldwide. The major obstacle in treating CRC occurs when tumour cells form metastases in distant organs. A critical step in metastasis formation is the modulation of the surrounding environment and priming of pre‐metastatic niches via autologous and heterologous cell‐cell communication. Tumour‐derived extracellular vesicles (T‐EV) have been identified to exert a striking impact on the ability of tumour cells to colonize distant organs. In this study we identified in particular the large plasma membrane‐derived microvesicles as critical determinants driving CRC invasiveness.

**Methods**: The impact of T‐EV from CMT93 CRC cells on tumour invasion was investigated with trans‐well invasion assays. The T‐EV were then subjected to biochemical, quantitative as well as proteomic analysis to elucidate a molecular signature responsible for their effects on invasion and metastasis.

**Results**: Using a syngeneic mouse model for CRC metastasis, we identified two variants of the CMT93 cell line, which differed significantly in their metastatic behaviour in vivo. Using in vitro assays, we demonstrated that while the cells did not show any differences regarding their basic tumorigenic characteristics as measured by proliferation, invasion and migration assays, they were strikingly different in their secretome, in particular in their released T‐EV. On the one hand, the malignant CMT93 variant released higher amounts of plasma membrane‐derived microvesicles (MV) compared to the benign CMT93 variant. On the other hand, analysis of the MV cargo by proteomics and metabolomics revealed an enrichment of factors associated with tumour adhesion, migration and angiogenesis, which might potentially explain the pro‐invasive phenotype of the MV from the malignant CMT93 variant.

**Summary/Conclusion**: Taken together, this study highlights the importance of T‐EV in metastatic colonization and identifies a specific proteomic and metabolic MV signature that determines the invasive phenotype of CMT93 cells.

### PS13.03 The effect of extracellular vesicles and other microenvironmental factors on the chemosensitivity of colorectal cancer patient‐derived organoids

András Áron Á Soós, Semmelweis University, Department of Genetics, Cell‐ and Immunobiology

Ádám Nagy, Semmelweis University, Department of Genetics, Cell and Immunobiology, Molecular Cancer Biology Research Group

Zoltán Wiener, Semmelweis University, Department of Genetics, Cell and Immunobiology, Budapest, Hungary

**Introduction**: Colorectal cancer (CRC) is one of the most frequent causes of cancer‐related death. Patient‐derived organoids maintain the cellular and genetic heterogeneity of the original tissues and have proved to be so far the best in vitro model of human cancers. Thus, they are a promising tool in the personalized medicine to select effective drug combinations. However, organoids contain only cells of epithelial origin and they lack the stromal component that may fundamentally modify the outcome of drug tests. Fibroblasts are an abundant cell type in the tumor stroma and they critically contribute to tumorigenesis in CRC. Here we studied whether extracellular matrix components and EVs derived from fibroblasts, a stromal cell type that critically contributes to tumorigenesis, modify the sensitivity of CRC organoids to selected chemotherapeutic drugs.

**Methods**: We used fibroblasts and CRC patient‐derived organoids, cultured in Matrigel or in collagen I. The Medical Research Council of Hungary approved the experiments and informed consent was obtained from patients. EVs were detected with antibody‐coated beads, NTA and TEM. Drug sensitivity was characterized with the IC50 value.

**Results**: Based on a previous drug screen carried out in an organoid library, we selected molecules that had an effect on the majority of CRC organoids (17‐AAG, gemcitabine). We found a large variation among patients when determining the IC50 with cell viability or measuring the organoid diameter. Although collagen I resulted in a morphological change of the organoids with migrating cells, this had no statistically different effect on the IC50. Similarly, fibroblast‐derived EVs did not modify the sensitivity of organoids to the studied chemotherapeutic drugs.

**Summary/Conclusion**: We provide evidence that changes in the ECM or the presence of EVs have no major effect on the IC50 value in CRC organoids. Thus, our results suggest that patient‐derived organoids may be a robust tool in personalized medicine even without stromal components.

### PS13.04 Using 3D biomimetic models to test the efficacy of targeting cancer‐associated fibroblast‐derived extracellular vesicles to overcome chemoresistance in pancreatic cancer

Chae Y. Eun, Lawrence J. Ellison Institute for Transformative Medicine of USC

Weikun Xiao, Lawrence J. Ellison Institute for Transformative Medicine of USC

Xinyu Zhang, Lawrence J. Ellison Institute for Transformative Medicine of USC

Charlene DeKalb, Lawrence J. Ellison Institute for Transformative Medicine of USC

Reginald Hill, Lawrence J. Ellison Institute for Transformative Medicine of USC

**Introduction**: Extracellular vesicles play a critical role in promoting proliferation and survival of cancer cells. Our previous studies have shown that cancer‐associated fibroblasts (CAFs), which make up the bulk of pancreatic ductal adenocarcinomas (PDAC), hypersecrete exosomes upon chemotherapy treatment. When these CAF‐derived exosomes were taken up by cancer cells, we observed more cell proliferation and chemoresistance. Considering these findings, there is a clear need to develop therapeutic strategies that target exosome biogenesis and secretion. Here, we investigate the effectiveness of several compounds, including farnesyl transferase inhibitors, imidazoles, and tricyclic antidepressants in blocking exosome hypersecretion and suppressing chemoresistance in PDAC CAF and cancer cell lines.

**Methods**: Human PDAC cell line PANC‐1 was used to test different compounds’ effectiveness in reducing exosome secretion. Exosome secretion was measured by comparing tetraspanin biomarker levels from cell culture media using NanoView Biosciences’ ExoView R100. In addition, we established a 3D organoid‐CAF co‐culture system from our genetically engineered PDAC mouse models to better understand the impact of these drugs in a biomimetic system complete with tumor‐promoting fibroblasts.

**Results**: Using human PANC‐1 cells, we observed the characteristic hypersecretion of exosomes upon chemotherapy (gemcitabine) treatment; this hypersecretion was reduced when the cells were treated together with ketoconazole, tipifarnib, neticonazole, climbazole, and imipramine. With our 3D biomimetic model, we observed that some of the tested compounds showed both a reduction in exosome secretion and significantly increased sensitivity to chemotherapy even in the presence of growth and chemoresistance‐promoting fibroblasts.

**Summary/Conclusion**: Together, our 2D and 3D models illuminate the potential for pharmacological targeting of exosome secretion as a treatment option that can improve treatment efficacy in PDAC.

### PS13.05 Glioblastoma Cell Populations With Distinct Oncogenic Programs Release Podoplanin as Procoagulant Extracellular Vesicles

NADIM TAWIL, McGill University ‐ RIMUHC

Rayhaan bassawon, McGill

Brian Meehan, Ri‐MUHC

Ali Nehme, McGill

Laura Montermini, RIMUHC

Cristiana Spinelli, McGill‐RIMUHC

Shilpa Chennakrishnaiah, McGill RIMUHC

Dongsic Choi, RI MUHC

Lata Adnani, McGill RIMUHC

Janusz Rak, MD, PhD, Professor

**Introduction**: Vascular anomalies, including thrombosis, are a hallmark of glioblastoma (GBM) and an aftermath of dysregulated cancer cell genome and epigenome. The upregulation of podoplanin (PDPN) by cancer cells has recently been linked to an increased risk of venous thrombo‐embolism (VTE) in GBM patients. Thus, dysregulation and release of this transmembrane platelet‐activating protein by transforming events are of considerable interest.

**Methods**: We used single cell and bulk transcriptome data mining, as well as cellular and xenograft models in mice to analyze the nature of cells expressing PDPN, their impact on activation of coagulation system, and platelets. We also took advantage of various analytical techniques including immunoblotting, immunofluorescence, electron microscopy, density gradient fractionation, Cytoflex, and ELISA.

**Results**: We report that PDPN is expressed by a distinct (mesenchymal) GBM cell subpopulation and downregulated by oncogenic mutations of EGFR and IDH1 genes, via changes in chromatin modifications (EZH2) and DNA methylation, respectively. GBM cells exteriorize their PDPN and/or tissue factor (TF) as cargo of exosome‐like extracellular vesicles (EVs) shed from cells in vitro and in vivo. Injection of glioma PDPN‐EVs activates platelets, while TF‐EVs weakly activate the clotting cascade. Similarly, increase of platelet activation (PF4) or coagulation markers (D‐dimer) occurs in mice harboring the corresponding glioma xenografts expressing PDPN or TF, respectively. Co‐expression of PDPN and TF by GBM cells cooperatively affects tumor microthrombosis.

**Summary/Conclusion**: In GBM distinct cellular subsets drive multiple facets of cancer‐associated thrombosis. These cells may represent anticoagulant targets and indicators of VTE risk. We suggest that the preponderance of PDPN expression as a risk factor in GBM and involvement of platelets in our GBM model may merit investigating antiplatelets for potential inclusion in VTE management strategies in GBM setting.

### PS13.06 Expression pattern of integrins in ganglioside‐remodeling melanoma EVs

Kei Kaneko, Department of Life Biomedical Sciences, College of Life and Health Sciences, Chubu University

Yuhsuke Ohmi, Department of Clinical Engineering, College of Life and Health Sciences, Chubu University

Mariko Kambe, Department of Life Biomedical Sciences, College of Life and Health Sciences, Chubu University

Qi Li, Department of Life Biomedical Sciences, College of Life and Health Sciences, Chubu University

Hiroaki Kitasawa, Department of Life Biomedical Sciences, College of Life and Health Sciences, Chubu University

Yesmin Farhana, Department of Biochemistry II, Nagoya University Graduate School of Medicine

Satoko Yamamoto, Department of Life Biomedical Sciences, College of Life and Health Sciences, Chubu University

Yoko Kitaura, Department of Clinical Engineering, College of Life and Health Sciences, Chubu University

Takako Ito, Department of Clinical Engineering, College of Life and Health Sciences, Chubu University

Orie Tajima,Department of Life Biomedical Sciences, College of Life and Health Sciences, Chubu University

Koichi Furukawa, Chubu University College of Life and Health Sciences

Keiko Furukawa, Department of Life Biomedical Sciences, College of Life and Health Sciences, Chubu University

**Introduction**: Ganglioside, sialic acid‐containing glycosphingolipids, are konwn as a regulator of cell proliferation, and human malignant melanomas express high levels of ganglioside GD3. To analyze the roles of GD3, we established GD3‐over expressing cells (GD3+) from a GD3‐lacking mutant cell line (GD3‐) by introducing GD3 synthase cDNA. Using these cell lines, we demonstrated that GD3 enhances tumor malignancy such as cell proliferation and invasion activities, and upregulates phosphorylation levels of focal adhesion kinase (FAK), p130Cas and paxillin. We also reported integrin β1 localizes in glycolipid‐enriched microdomain (GEM)/rafts of GD3+ melanomas. During cell adhesion, phosphorylated FAK was found at GEM/rafts in GD3+ melanomas. The aim of this study is to clarify the behaviors and roles of integrins in the extracellular vesicles (EVs) from ganglioside‐remodeling melanomas.

**Methods**: EVs were isolated by ultracentrifugation from the culture medium of GD3+/GD3‐ melanoma cells. Isolated EVs were analyzed by using Western blotting. GD3‐ melanoma cells were cultured with FCS‐free Dulbecco's modified Eagles (DMEM) medium and incubated with GD3+ melanoma‐derived EVs. After incubation, cells were lysed and analyzed by Western blotting.

**Results**: Among integrin family members, some integrins (alfa2, alfa3, alfa6) were highly expressed in GD3+ EVs, while others (e.g. beta3) were not. Addition of GD3+ melanoma‐derived EVs to cultured cells resulted in the induction of activation of ERK1/2 and Akt, suggesting that GD3 on EVs might play important roles in the regulation of tumor environments.

**Summary/Conclusion**: We identified the expression patterns of integrins in EVs were different between GD3+ and GD3‐ melanoma cell lines. Gangliosides may contribute to selective packaging of integrins into EVs.

### PS13.07 Hypoxic exosomes are messengers of signalings driving Neuroblastoma aggressiveness

Noemi Torriero, Dept. of Industrial Engineeing (DII) University of Padua

Pina Fusco, Dept. of Industrial Engineeing (DII) University of Padua

Luca Zanella, Dept. of Industrial Engineeing (DII) University of Padua

Maria Rosaria Esposito, Dept. of Industrial Engineeing (DII) University of Padua

Elisa Cimetta, Dept. of Industrial Engineeing (DII) University of Padua

**Introduction**: Neuroblastoma (NB) is a paediatric solid tumour with heterogeneous biological, genetic and clinical characteristics. About half of cases classify as high‐risk for disease relapse, with survival rates < 40% at 5 years from diagnosis. Low oxygen tension in the cellular microenvironment relates to NB aggressive phenotypes. This study aims at assessing the role of exosomes (EXOs) released at different oxygenation conditions in directing NB progression, in order to uncover novel mechanisms involved in cancer dissemination.

**Methods**: EXOs were observed with a Tecnai G2 (FEI) transmission electron microscope operating at 100 kV. EXOs size distribution and particle quantification were evaluated using qNano system (Izon Science Ltd.). We performed proteome analysis of EXOs’ cargo by liquid chromatography with tandem mass spectrometry (LC‐MS/MS) using micro‐LC Eksigent Technologies. We evaluated miRnome content of EXOs’ cargo using the FirePlex(R) miRNA assay (abcam, Cambridge, MA, USA).

**Results**: We purified EXOs released by NB cell lines (SKNAS and SKNDZ) under controlled oxygen concentrations: normoxia (20% O2), hypoxia (1.5% O2) and reoxygenation (1.5 % O2 followed by 20% O2). The cargo was analyzed focusing on the miRnome and proteome profile. We found that five miRNAs (miRNA‐210‐3p, miRNA‐183‐5p, miRNA‐33a‐5p, miRNA‐378i, miRNA‐195‐5p) regulated in hypoxia and persisting after reoxygenation, are associated with tumour invasion. Moreover, hypoxic EXOs are enriched in proteins associated with remodelling of epithelial adherens junctions, EIF2 signaling and protein ubiquitination pathway. Hypoxic EXOs also carry high numbers of proteins associated with cell growth. Finally, we identified TP53 and MAPT as upstream regulators of hypoxic EXOs proteins driving tumour progression. Combining the miRNA gene targets and the protein dataset, we identified the actin cytoskeleton as a regulated pathway in hypoxia.

**Summary/Conclusion**: Our findings suggest that hypoxic EXOs are enriched in miRNAs and proteins associated with pathways selectively driving tumour dissemination. These results increase our knowledge on the tumour‐microenvironment cross‐talk and may contribute to opening novel lines of research seeking alternative treatment strategies.

### PS13.08 Exosomes from imatinib resistant K562 cells increase survival of imatinib sensitive cells in the presence of imatinib

Tereza Hrdinova, Institute of Hematology and Blood Transfusion

Ondrej Toman, Institute of Hematology and Blood Transfusion

Jiri Dresler, Military Health Institute, Military Medical Agency

Jana Klimentova, Faculty of Military Health Sciences, University of Defense in Brno

Barbora Salovska, Department of Genome Integrity, Institute of Mlecular Genetics of The Czech Academy of Sciences

Vaclava Polivkova, Institute of Hematology and Blood Transfusion

Katerina Machova Polakova, Institute of Hematology and Blood Transfusion

Hana Kabickova, Military Health Institute, Military Medical Agency

Barbora Brodska, Institute of Hematology and Blood Transfusion

Matyas Krijt,Institute of Hematology and Blood Transfusion

Jan Zivny, Institute of Pathological Physiology, First Faculty of Medicine, Charles University

Daniel VyoralInstitute of Hematology and Blood Transfusion

Jiri Petrak, BIOCEV, First Faculty of Medicine, Charles University

**Introduction**: Chronic myeloid leukemia is a malignant hematopoietic disease characterized by reciprocal translocation between chromosomes 9 and 22 t(9;22). This rearrangement is known as the Ph chromosome and generates the Bcr‐Abl oncoprotein with constitutive tyrosine kinase activity. Targeted treatment by tyrosine kinase inhibitors markedly improved survival of patients with CML. However, development of resistance limits benefits from the treatment. Recently, exosomes released by normal and cancer cells participate in drug resistance and cancer progression by exchange of DNA, RNA or proteins between cells. The aim of our study was to characterize exosomes released by imatinib sensitive (K562) and imatinib resistant K562 (K562IR) cells.

**Methods**: K562IR cells were derived from imatinib sensitive K562 cell line by prolonged incubation in increasing imatinib concentrations in cell culture medium. Exosomes were isolated from K562 and K562IR cells by differential centrifugation. Internalization of exosomes was observed under a confocal laser scanning microscope. Label free quantification proteomic analysis was performed using Q‐Exactive mass spectrometer. Flow cytometric analysis was used to determine the potential of identified proteins as cell surface markers.

**Results**: The K562IR derived exosomes were internalized by K562 cells, which thereby increased survival of K562 cells in the presence of 2 μM imatinib. Using a label free quantification proteomic analysis there were identified over 3000 exosomal proteins of which 35 were found to be differentially expressed. From this differentially expressed group, a total of 3 membrane proteins, namely interferon'induced transmembrane protein 3 (IFITM3), CD146 and CD36 were markedly upregulated in K562IR derived exosomes and exhibited surface localization. The upregulation of these 3 proteins was verified in K562IR derived exosomes and also in K562IR cells. Flow cytometric analysis demonstrates the potential of CD146 and IFITM3 to serve as cell surface markers associated with imatinib resistance in K562 cells.

**Summary/Conclusion**: These results suggest that exosomes and their particular candidate surface proteins could be used as potential diagnostic markers of TKI drug resistance in CML therapy.

### PS13.09 The Von Willebrand Factor stamps Plasmatic Extracellular Vesicles from Glioblastoma Patients

Quentin Sabbagh, INSERM, CNRS, Université de Nantes

Gwennan ANDRE‐GREGOIRE, CRCINA UMR1232 and Integrated Center for Cancerology (ICO)

Carolina Alves‐Nicolau, INSERM, CNRS, Université de Nantes

Nicolas BIDERE, CRCINA UMR1232

Laetitia Guevel, INSERM, CNRS, Université de Nantes

Jean‐Sebastien Frenel, Institut de Cancérologie de l'Ouest

Julie Gavard, INSERM, CNRS, Université de Nantes, Institut de Cancérologie de l ' Ouest

**Introduction**: Although rare, glioblastoma is a devastating tumor of the central nervous system characterized by a poor survival and an extremely dark prognosis, making its diagnosis, treatment and monitoring highly challenging. Numerous studies have highlighted extracellular vesicles (EVs) as key players of tumor growth, invasiveness and resistance, as they carry oncogenic material in the local tumor microenvironment and at distance. Indeed, recent reports demonstrated the presence of brain tumor‐derived EVs into body fluids such as plasma and cerebrospinal fluid. However, whether vesiclemia reflect health status and changes in homeostasis is still not fully elucidated.

**Methods**: Here, we established rigorous, reproducible methodologies to isolate EVs from plasma collected at different time points within the clinical management of GBM patients. The vesiclemia is expressed in particles/mL and can be measured by at least two different and complementary methods, according to the International Society of Extracellular Vesicles (ISEV) recommendations: Electron microscopy or atomic force microscopy, with close‐up and wide field provided, Single particle analysis techniques, such as tunable resistive pulse sensing (TRPS), single particle tracking analysis and interferometry, and Biochemical techniques, such as ELISA. We also reiterate few hints on storage, minimal volume and pre‐processing of the samples.

**Results**: Our findings confirm that plasmatic EVs could be isolated and characterized with standard protocols, thereby ensuring the reliability of measuring vesiclemia, i.e. extracellular vesicle concentration in plasma. To tackle the question whether quantity and/or quality of circulating EVs could represent potential biomarker of glioblastoma (GBM) evolution, we performed a longitudinal analysis of the vesiclemia (i.e. concentration of particles/mL) throughout tumor management. This unveils that vesiclemia is a dynamic and fluctuating parameter, which could be reflecting tumor burden and/or response to treatments. Further label‐free liquid chromatography tandem mass spectrometry unmasks the Von Willebrand Factor (VWF) as a selective protein hallmark for GBM‐patient isolated EVs.

**Summary/Conclusion**: Our data thus support the notion EVs from GBM patients are enriched with selective protein cargos that can be further surveyed in circulating EVs, together with vesiclemia.

### PS13.10 Breast cancer‐derived exosomes trigger stromal fibroblasts in tumor microenvironment via microRNA cargo

Iolanda Scognamiglio, Department of Molecular Medicine and Medical Biotechnology, University of Naples Federico II, Via Pansini 5, IT80131, Napoli.

Lorenza Cocca, Department of Molecular Medicine and Medical Biotechnology, University of Naples Federico II, Via Pansini 5, IT80131, Napoli.

Ilaria Puoti, Department of Molecular Medicine and Medical Biotechnology, University of Naples Federico II, Via Pansini 5, IT80131, Napoli.

Francesco Palma, Department of Molecular Medicine and Medical Biotechnology, University of Naples Federico II, Via Pansini 5, IT80131, Napoli.

Francesco Ingenito, Department of Molecular Medicine and Medical Biotechnology, University of Naples Federico II, Via Pansini 5, IT80131, Napoli.

Cristina Quintavalle, Istituto di Endocrinologia e Oncologia Sperimentale IEOS‐CNR, Via Pansini 5, IT80131, Napoli.

Alessandra Affinito, Percuros BV, Albinusdreef 2, 2333 ZA Leiden, The Netherlands.

Giuseppina Roscigno, Department of Molecular Medicine and Medical Biotechnology, University of Naples Federico II, Via Pansini 5, IT80131, Napoli.

Silvia Nuzzo, IRCCS SDN SpA, Via Gianturco 113, IT80143, Naples Italy.

Maria Patrizia Stoppelli, International Institute of Genetics and Biophysics (IGB), Consiglio Nazionale delle Ricerche, Via Marconi, Napoli, Italy

Stefania Belli, International Institute of Genetics and Biophysics (IGB), Consiglio Nazionale delle Ricerche, Via Marconi, Napoli, Italy

Rosario Vincenzo ChianeseDepartment of Molecular Medicine and Medical Biotechnology, University of Naples Federico II, Via Pansini 5, IT80131, Napoli.

Gerolama Condorelli, Department of Molecular Medicine and Medical Biotechnology, University of Naples Federico II, Via Pansini 5, IT80131, Napoli.

**Introduction**: Triple negative breast cancer (TNBC) is the most aggressive breast cancer subtype, because of its high metastasis potential. TNBC clinical prognosis and patient treatment are undermined by the recruitment of a strong and unique tumor microenvironment (TME), that is mainly composed of activated fibroblasts (Cancer‐Associated Fibroblasts‐CAFs) able to endorse tumor hallmarks. Increasing evidence demonstrated that exosomes can mediate the cross‐talk between cancer cells and TME. Accordingly, we examined the contribution of TNBC‐derived exosomes and their microRNAs (miRNAs) cargo in the activation of normal fibroblasts (NFs) towards CAFs.

**Methods**: Exosomes were obtained from TNBC cell line (MDA‐MB‐231) and incubated with primary cultures of normal fibroblasts (NFs) to study their activation. Thus, in vitro transwell migration and contraction assays, together with western blot and RT‐PCR analysis were performed on NFs upon TNBC‐derived exosome treatment to demonstrate the functional and molecular conversion of NFs to CAFs. Moreover, an organotypic co‐culture model was set up with exosome‐activated fibroblasts and normal epithelial breast cells (MCF10A) to study the impact of fibroblast activation on breast cells invasion ability in an in vivo‐like environment. A small‐RNA sequencing was performed on NFs treated with TNBC‐derived exosomes to investigate the involvement of exosomal miRNA cargo in the transformation of NFs into CAFs.

**Results**: We demonstrated that NFs treated with TNBC‐derived exosomes exhibited increased collagen contraction and cell migration abilities, widely reported as the principal hallmarks of activated fibroblasts within the TME. Furthermore, we observed that those fibroblasts activated by exosomes promoted the invasion potential of normal breast epithelial cells as assessed by the organotypic co‐culture model. A small RNA‐sequencing on NFs treated with TNBC‐derived exosomes revealed several miRNAs up‐regulated in NFs after exosome treatment, that included miRNAs‐185‐5p, ‐652‐5p, and ‐1246. In particular, we observed that the synergistic action of these miRNAs was able to strongly activate NFs to CAFs. Indeed, the combined miRNAs boosted fibroblast migration and contraction abilities, increasing the expression of typical molecular markers associated to CAFs, to cell invasion and motility. Definitely, we found that these miRNAs led to a specific CAF subspecialization towards a pro‐migratory functional state.

**Summary/Conclusion**: All together these data highlighted the role of breast cancer cells in the re‐education of TME, thus contributing to tumor evolution

### PS13.11 Role of small extracellular vesicles in the non‐targeted bystander effects of targeted radiotherapy

Jihad Karam, Institut de Recherche en Cancérologie de Montpellier

Julie Constanzo, IRCM, Institut de Recherche en Cancérologie de Montpellier, INSERM U1194, Université de Montpellier, Institut régional du Cancer de Montpellier

Alexandre Pichard, IRCM, Institut de Recherche en Cancérologie de Montpellier, INSERM U1194, Université de Montpellier, Institut régional du Cancer de Montpellier

Laurent Gros, IRCM, Institut de Recherche en Cancérologie de Montpellier, INSERM U1194, Université de Montpellier, Institut régional du Cancer de Montpellier

Joël Chopineau, ICGM, Univ. Montpellier, CNRS, ENSCM, Montpellier, France

Marie Morille, ICGM, Univ. Montpellier, CNRS, ENSCM, Montpellier, France

Jean‐Pierre Pouget, IRCM, Institut de Recherche en Cancérologie de Montpellier, INSERM U1194, Université de Montpellier, Institut régional du Cancer de Montpellier

**Introduction**: Non‐targeted effects have been identified as contributors to the efficacy of targeted radiotherapy (TRT) of cancer. Triggered by components secreted by irradiated cells, these cytotoxic effects can be observed at a short distance from the irradiated cells (bystander response), or at longer distances (systemic response). Unlike conventional X‐ray radiotherapy, the precise contribution of small extracellular vesicles (EV) to these effects in TRT has not been explored yet.

**Methods**: Small EV were isolated from the conditioned medium of murine B16F10 melanoma cells using differential centrifugations and further purified on a sucrose gradient. These cells were previously treated with 4MBq/mL of Auger electron‐based TRT (Auger EV), irradiated by X‐rays with a 3 × 8 Gy dose (X EV), or left untreated (control EV). Next they were characterized by nanoparticle tracking analysis and western blot and incubated in vitro with untreated B16F10 cells to assess their therapeutic efficacy.

**Results**: We obtained distinct responses when cells were treated with the different vesicles. For example, the colony formation ability of B16F10 cells treated with 4 × 10^8 particles/mL of Auger EV decreases by 50%, as compared to X EV with a 33% decrease and control EV which decreased survival from 2 to 15%. To understand the mechanisms leading to these cytotoxic effects, we have shown an increase of 5–10% in apoptosis of recipient cells following 3h of treatment only, with Auger and X EV compared to control EV. However, we observed a delayed increase in DNA damage as measured by using the formation of γ‐H2AX foci and micronuclei assays. Treatment with Auger EV increased 5 times the number of γ‐H2AX foci and 3 times the number of micronuclei after 48h and 72h of treatment respectively, while X EV increased 3 times γ‐H2AX foci and 2 times the number of micronuclei, compared to control EV. Furthermore, the DNA content of these small EV was explored. We found that Auger EV carry between 3 × 10^‐4 and 10^‐2 fg of dsDNA per particle versus 0.2 fg for X EV and undetectable dsDNA in control EV.

**Summary/Conclusion**: Our results establish the role of small extracellular vesicles in Auger TRT as mediators of an intercellular communication network that contributes to the efficacy of the therapy. Ongoing proteomic and miRNA profiling studies of these vesicles will deepen our understanding of the cytotoxic mechanisms generated by them following their uptake by recipient cells.

### PS13.12 New targets and new approaches in multiple myeloma: extracellular vesicles as liquid biomarkers

Antonia Reale, Medical Oncologist, Monash University

Iśka Carmichael, Monash University

Rong Xu, Monash University

Tiffany Khong, Monash University

Sridurga Mithraprabhu, Monash University

Maoshan Chen, NA

David W Greening, Baker Institute

Andrew Spencer, MD, Monash University

**Introduction**: Circulating small extracellular vesicles (sEV) represent promising non‐invasive biomarkers that may aid in the diagnosis and risk‐stratification of multiple myeloma (MM), an incurable blood cancer, given the optimization of their isolation/purification method.

**Methods**: sEV isolated from human MM cell lines (HMCL) were purified using density gradient ultracentrifugation (DG‐UC). sEV were isolated from peripheral blood plasma (PBPL) using commercially available kits. Pre‐analytical conditions (collection tube types, storage conditions) were assessed for sEV yield and EV‐marker enrichment. Proteomic profiling (nLC and high‐resolution mass spectrometry) and functional studies were also performed.

**Results**: Highly purified HMCL‐sEV were comprehensively characterized by specific EV‐marker enrichment and morphology. Importantly, we demonstrated that HMCL‐sEV are readily taken up by stromal cells to functionally modulate proliferation. Higher purity/recovery, evaluated by immunoblotting, nanoparticle tracking analysis and electron microscopy, were observed for PBPL‐sEV isolated using a resin‐based kit. Fresh PBPL samples and Streck‐RNA collection tubes provided sEV with higher purity vs frozen PBPL and EDTA tubes. Functionally, PBPL‐sEV were shown to regulate stromal cell proliferation and migration. In turn, pre‐educated stromal cells favour HMCL adhesion. Interestingly, PBPL‐sEV isolated from patients with both pre‐malignant plasma cell disorders (monoclonal gammopathy of undetermined significance, MGUS; smouldering MM, SMM) and newly diagnosed MM have a similar ability to promote cell migration and adhesion, suggesting a role for both malignant and pre‐malignant PBPL‐sEV in disease progression. Proteomic profiling of MM‐sEV (339 proteins) revealed enrichment of factors implicated in cell migration and adhesion, in comparison to healthy donors sEV.

**Summary/Conclusion**: The characterization and proteomic profiling of disease‐specific circulating sEV as a biomarker discovery strategy may provide translational applications in MM. Pre‐analytical and isolation/purification steps are of critical importance for the characterization of sEV. Importantly, MM‐sEV may play an important role in disease progression by re‐programming the tumour microenvironment.

### PS13.13 GD3/GD2‐expressing glioma‐derived EVs enhance malignant properties of gliomas, and regulate tumor microenvironments

Koichi Furukawa, Chubu University College of Life and Health Sciences

Yuhsuke Ohmi, Department of Clinical Engineering, College of Life and Health Sciences, Chubu University

Qi Li, Department of Life Biomedical Sciences, College of Life and Health Sciences, Chubu University

Hiroaki Kitasawa, Department of Life Biomedical Sciences, College of Life and Health Sciences, Chubu University

Yesmin Farhana, Department of Biochemistry II, Nagoya University Graduate School of Medicine

Satoko Yamamoto, Department of Life Biomedical Sciences, College of Life and Health Sciences, Chubu University

Yoko Kitaura, Department of Clinical Engineering, College of Life and Health Sciences, Chubu University

Takako Ito, Department of Clinical Engineering, College of Life and Health Sciences, Chubu University

Kei Kaneko, Department of Life Biomedical Sciences, College of Life and Health Sciences, Chubu University

Mariko Kambe,Department of Life Biomedical Sciences, College of Life and Health Sciences, Chubu University

Orie Tajima, Department of Life Biomedical Sciences, College of Life and Health Sciences, Chubu University

Keiko FurukawaDepartment of Life Biomedical Sciences, College of Life and Health Sciences, Chubu University

**Introduction**: Gliomas account for approximately 50% of all primary brain neoplasms, and the most common type is glioblastoma multiforme. Glioblastoma multiforme is an aggressive, rapidly progressive, infiltrative parenchymal neoplasm with a poor prognosis. Ganglioside GD3 and GD2 are frequently expressed in human gliomas, and have been reported to enhance tumor malignancy such as progression and invasion. We have also reported that GD3/GD2 enhance tumor properties of gliomas by increasing activation levels of membrane receptors and cell signals. Extracellular vesicles (EVs) secreted from cancer cells have been reported to be involved in the regulation of tumor microenvironment and metastasis. However, roles of gangliosides in EVs secreted from gliomas have not been well understood.

**Methods**: Ganglioside expression on glioma cell lines was analyzed by flow cytometry (Accuri6) with specific monoclonal antibodies for individual gangliosides. Gangliosides on EVs were also analyzed by Accuri6 using Tim4‐beads. EVs were isolated by ultra‐centrifugation methods. Tumor phenotypes were analyzed by MTT assay (cell growth), Boyden‐chamber method (invasion), RT‐CES (cell adhesion), and cell motility assay. Activation of signaling molecules was analyzed by immuno‐blotting.

**Results**: Ganglioside GD3 synthase cDNA was transfected into a glioma cell line, U251 MG, leading to the establishment of GD3/GD2‐expressing transfectant lines and controls. One line each of GD3/GD2+ cells (GT16) and negative cells (CV3) were used for subsequent analysis. GT16‐EVs caused stronger activation of Erk1/2 and Akt than CV3‐CVs when added to cultured murine astrocytes. GT16‐EVs caused activation of those molecules when added to cultured CV3 cells. GT16‐EVs also caused increased invasion activity of CV3 cells.

**Summary/Conclusion**: All these results suggested that GD3/GD2‐expressing glioma‐derived EVs might enhance malignant properties of gliomas, and also regulate tumor microenvironments.

### PS13.14 Small extracellular vesicles (sEV): a new “liquid biopsy” for CD20 monitoring in DLBCL?

Marine AITAMER, EA3842 CAPTuR

Hussein Akil, UMR CNRS 7276 /INSERM U1262

Chantal Vignoles, UMR CNRS 7276 /INSERM U1262

Julie Abraham, Service d'Hématologie Clinique, CHU de Limoges

Nathalie Gachard, UMR CNRS 7276 /INSERM U1262, Laboratoire d'hématologie, CHU de Limoges

Jean Feuillard, UMR CNRS 7276 /INSERM U1262, Laboratoire d'hématologie, CHU de Limoges

Marie‐Odile Jauberteau, EA3842 CAPTuR Facultés de Médecine et de Pharmacie

Hamasseh Shirvani, Institut Roche, 30, cours de l'île Seguin 92650 Boulogne‐Billancourt

Hafidha Bentayeb, EA3842 CAPTuR Facultés de Médecine et de Pharmacie

Danielle Troutaud,EA3842 CAPTuR Facultés de Médecine et de Pharmacie

**Introduction**: Diffuse Large B‐cell Lymphomas (DLBCL) are aggressive non‐Hodgkin lymphomas with two main sub‐groups (ABC and GCB). Among these, GCB are of better prognosis. Monoclonal anti‐CD20 antibody, rituximab, in combination with CHOP chemotherapy (R‐CHOP immunochemotherapy) has been widely used with favorable results; however, 30–40% of patients are not cured and will relapse or be refractory to R‐CHOP. Decreased CD20 expression has been postulated to be one of the most important etiologies contributing to rituximab resistance. Interestingly, small Extracellular Vesicles (sEV) including exosomes, carrying the CD20, could be involved in immunotherapy resistance in DLBCL. Moreover, recent data showed that RNA contents of peripheral EVs from DLBCL patients could enable disease monitoring through liquid biopsy and can be used as predictors of prognosis and chemotherapeutic efficacy.

**Methods**: Here, we performed a comparative study of sEV production by 2 GCB and 3 ABC DLBCL cell lines, and evaluated CD20 content in relation to: (i) CD20 membrane level of parental cell lines (ii) the differential capacity of autologous and heterologous small EVs to rescue lymphoma cells from rituximab‐mediated complement‐dependent cytotoxicity (CDC) (iii) cell treatments (i.e. rituximab, TrkB activation) that could modulate CD20 gene (MS4A1) expression.

**Results**: Our results show that sEV production varied significantly between DLBCL cells, independently of the GCB/ABC subtype. CD20 level of sEV was consistent with that of membrane parental cells, and decreased after rituximab exposure. Moreover, we demonstrate that DLBCL‐derived sEVs have differential capacity to interfere with immunotherapy that was associated with the CD20 membrane expression (MFI) of parental cells. Interestingly, we show that BDNF/TrkB axis, that we previously reported as an autocrine/paracrine survival pathway in DLBCL, also target sEV release enhancing protection to rituximab.

**Summary/Conclusion**: Collectively, beyond their role in exercising a decoy function against rituximab, our data strongly suggest that CD20 level in sEVs reflects regulation of CD20 in parental cells, and argue for a potential role of peripheral sEVs from patients for monitoring changes in CD20 phenotype of tumoral B cells.

## PS14 EV Protein Biomarkers

Chair: Gabriella Pocsfalvi, EVs & MS Laboratory, Institute of Biosciences and BioResources, National Research Council of Italy, Italy

Chair: Michael Graner, University of Colorado Anschutz Medical Campus, United States

### PS14.01 Evaluation of an exosomal protein as early prognostic biomarker in melanoma patients

Andrea Agüera‐Lorente, Universidad del País Vasco / Euskal Herriko Unibertsitatea

Maria Larrinaga, Universidad del País Vasco / Euskal Herriko Unibertsitatea

Maria Dolores Boyano, Universidad del País Vasco / Euskal Herriko Unibertsitatea

Aintzane Asumendi, Universidad del País Vasco / Euskal Herriko Unibertsitatea

Aintzane Apraiz, Universidad del País Vasco / Euskal Herriko Unibertsitatea

**Introduction**: Malignant melanoma is one of the most aggressive and deadly cancers due to its high rate of metastasis, even when identified in early stages. The lack of effective strategies for metastatic melanoma supports the need for molecular markers to accurately classify patients.

In a previous proteomic study, we identified some exosomal proteins as prospective biomarkers in melanoma, based on their differential expression in exoproteomes from melanocytes vs melanoma cells. We chose one of them, hereinafter called protein A, with the objective to evaluate its usefulness to stratify melanoma patients according to their progression. For that purpose we analized melanoma biopsies and serum‐derived exosomes.

**Methods**: Melanoma patients were recruited at the Basurto and Cruces Hospitals. Disease stages were classified according to the AJCC. The study was conducted in accordance with the Declaration of Helsinki principles and approved by the Euskadi Ethics Committee.

EVs derived from human sera were purified based on media concentration and differential ultracentrifugation. Sections of FFPE blocks were analyzed by immunochemistry (IHC). Protein A in sera‐derived EVs was quantified by ELISA.

**Results**: We first identified the protein A in histologic biopsies of melanoma patients and EVs from sera samples. CrioEM, Western Blot (CD63, CD81, CD9) and NanoSight analysis indicated an exosomal enrichment in the isolated EVs.

The IHC analysis of melanoma biopsies revealed a higher expression of the protein A in advanced stages but no significant differences were observed when comparing developing‐metastasis early stages (I and II) with non‐developing ones.

Concerning liquid biopsy, our results showed that the protein A amount in sera‐derived exosomes can discriminate patients in early stages (II AJCC) in terms of their progression.

Studies are ongoing using exosomes from protein A‐knockdown cells to investigate some of its biological functions in melanoma progression.

**Summary/Conclusion**: Protein A could be a useful biomarker to discriminate, in early‐stages, melanoma patients that will develop metastasis.

### PS14.02 Expression of ACE2, TMPRSS2, FURIN and PN1 in EVs secreted in the paranasal sinuses and nasal cavity. A comparative analysis with urinary and plasma EVs to understand the mechanisms of cellular tropism in Sars‐Cov2 infection

Luca Musante, University of Virginia

Christopher Wilcox, Georgetown University

Uta Erdbrügger, UE, University of Virginia

**Introduction**: Extracellular vesicles (EVs) secreted in the sinuses and the nasal cavity mucosa might play an important role as first line of defence from aerial pathogens. The current pandemic caused by SARS‐CoV‐2 (COVID‐19) highlights the fact that the human host defence system is still poorly understood especially in subjects with hypertension, diabetes and other comorbidities.

Angiotensin converting enzyme 2 (ACE2) is the receptor which allows SARS‐CoV‐2 to enter into cells through the proteolytic processing of its spike protein by transmembrane protease receptor serine type 2 (TMPRSS2) and furin. In parallel, Protease nexin 1 (PN1) inhibits furin ‐ among other proteases. These additional proteins can have a critical role in host defence but presently little is known about their regulation. The aim of this study is to evaluate if EVs secreted into the paranasal sinuses and nasal cavity and other common biofluids such as urine or blood harbour these proteins.

**Methods**: Paranasal sinuses and nasal cavity lavage fluid (NLF) was obtained by flushing 250 mL of saline nasal rinse solution made of sodium chloride and sodium bicarbonate using the squeeze bottle kit by Neilmed Sinus Rinse. After the first centrifugation at 4,600g the resulting supernatant was used to enrich EV by hydrostatic filtration dialysis (HFD). EVs from blood and urine were enriched by consolidate protocols: differential centrifugation and HFD. Western blot, NTA and TRPS were used to characterize EVs and associated markers

**Results**: Western blot analysis showed that ACE2, TMPRSS2, Furin and NP1 were detected in NLF while urinary EVs did not carry any furin. Interestingly, ACE2 in uEVs showed a higher molecular weight than NLF EVs. On the other hand, NP1 showed a higher molecular weight in NLF EVs which can reflect its activation trough the formation of carboxylic ester bonds with serine proteases at their active cleavage site. NTA and TRPS using a NP400 nanopore membrane showed to have a very close particle size distribution (PSD): NTA mean 218.3 ± 14.5 nm mode 167.67 ± 11.6 nm; TRPS mean 213.4 ± 11.3 nm mode 173.5 ± 5.8 nm and 1.13 × 109 ± 8.1 × 107 particles/mL and 2.36 × 107 ± 4.0 × 106 particles/mL for NTA and TRPS respectively.

**Summary/Conclusion**: EV isolated from NLF carry ACE2, TMPRSS2 furin and PN1 with a specific isoform profile for ACE2 and PN1. It has to be further investigated if this tissue specificity change following viral binding, entry and onset of the infection. NLF can be easily collected without the use of any invasive methods, thus offering a fair amount of EVs for downstream analysis.

### PS14.03 Extracellular vesicle and biomarkers to follow up patients with metastasic breast cancers

Mathilde Richard, CRCINA INSERM U1232

Gwennan ANDRE‐GREGOIRE, CRCINA UMR1232 and Integrated Center for Cancerology (ICO)

Julie Gavard, INSERM, CNRS, Université de Nantes, Institut de Cancérologie de l'Ouest

Laetitia Guevel, INSERM, CNRS, Université de Nantes

**Introduction**: Extracellular vesicles (EVs) have been suggested as promising biomarkers and can be used as source of tumor‐derived information in biofluids from cancer patients because they contain proteins from their originating cells and are readily available in fluids.

Small EVs are a heterogeneous group of cell‐derived membranous structures, defined as nanosized bioparticles (50‐300nm). They are produced by almost all organisms and cell types ‐ including tumor cells ‐ and are released from the cell into the extracellular space. They circulate via the various body fluids like blood, and urine where they have important roles in intercellular communication by transferring their contents.

Breast cancer is the most common cancer in women, it accounts for more than one‐third of all new cases of cancer in women. While between 20 to 50% of breast cancer patients will develop metastases, early detection is generally associated with a better prognosis and a 5‐year survival rate of 90%. Histological and phenotypical differences between tumors guide cancer diagnosis, prognosis and the selection of treatment. Metastasic breast cancers are graded on the basis of tumor structure, molecular characterization and cellular morphology, and subcategorized in 3 groups (HR+, HER2‐), (HER2+), Triple negative. In this project, we ambition to determine the vesiclemia standard value, number and size distribution, using the recently developed interferometry technology (Videodrop) that enables to quickly and reliably visualize and quantify nanoparticles in real time.

**Methods**: Our study is based on the characterization of EVs from plasma and urine isolated from the healthy population and patients with metastatic breast cancer. Our study is organized in three aims:

- Characterization of EVs from breast cancer patients and comparison with healthy donors
- Longitudinal analysis of vesiclemia throughout breast cancer treatment and follow‐up
- Determination of specific protein cargoes transported in circulating EVs

**Results**: Vesiclemia and size distribution will be monitored in randomized healthy population, to characterize EV variations and establish the normal range in healthy donors. In more than 40 breast cancer patients were followed up over one year to evaluate the clinical potential of vesiclemia in their blood and urine.

**Summary/Conclusion**: Our data aims at establishing vesiclemia as a new biological parameter in medical blood tests. We ambition to tailor new diagnostic and predictive approaches using EV tests as innovative therapeutic companion, a strategy coined under the name vesiclemia.

### PS14.04 Immunological surface markers of small and large extracellular vesicles from plasma of ALS patients

Daisy Sproviero, PhD, IRCCS Mondino Foundation

Eleonora Corridori, Genomic and post‐Genomic Unit, IRCCS Mondino Foundation, Pavia, Italy

Jessica Garau, Genomic and post‐Genomic Unit, IRCCS Mondino Foundation, Pavia, Italy

Stella Gagliardi, Genomic and post‐Genomic Unit, IRCCS Mondino Foundation, Pavia, Italy

Orietta Pansarasa, Genomic and post‐Genomic Unit, IRCCS Mondino Foundation, Pavia, Italy

Luca Diamanti, Neuro‐Oncology Unit, IRCCS Mondino Foundation, Pavia, Italy

Paolo Bergese, Dipartimento di Medicina Molecolare e Traslazionale, Università degli Studi di Brescia, 25123 Brescia, Italy

Annalisa Radeghieri, Department of Molecular and Tranlational Medicine‐Università degli Studi di Brescia

Cristina Cereda, Genomic and post‐Genomic Unit, IRCCS Mondino Foundation, Pavia, Italy

**Introduction**: Extracellular vesicles (EVs) have a central role in inflammatory processes and they could be plausible targets in Amyotrophic Lateral Sclerosis (ALS), which is characterized by an immunological reaction to motor neuron death (Cereda et al., 2008; Henkel et al., 2013). We have previously demonstrated that leukocyte derived EVs are upregulated in ALS patients and they can be considered possible markers of disease progression (Sproviero et al., 2019). The aim of this study was to investigate more specific immunological surface markers on large and small EVs (LEVs and SEVs) from plasma of ALS patients and healthy donors.

**Methods**: LEVs and SEVs were isolated from plasma of 30 sporadic ALS patients and 30 healthy controls (CTRLs) by differential centrifugation as previously described (Sproviero et al., 2018) and characterized by Nanoparticle Tracking Analysis (NTA), Atomic force microscopy (AFM), Colorimetric NANoplasmonic method (CONAN). In this study, we used a multiplex bead‐based flow cytometric assay for the detection of 37 surface protein markers in one sample simultaneously (MACSPlex Exosome Kit).

**Results**: Endosome‐specific tetraspanins (CD9 and CD63), endothelial marker (CD31), T‐cell and Natural Killer markers (CD2, CD45 and CD69), HLA Class 1 group (HLA‐ABC) and homing cell adhesion molecule (CD44) were more expressed in SEVs derived from CTRLs than in sALS patients (*, P < 0,05).

LEVs derived from CTRLs were enriched in cell adhesion molecule of endothelial cells and platelets (CD31, CD42a, CD41b and CD62P), tetraspanins CD63, CD9 and HLA‐ABC (*, P < 0,05) compared to sALS patients.

On the other hand, we demonstrated that the expression of the tetraspanin CD81 (*, P < 0,01), an MHC class II cell surface receptor (HLA‐DR) and the glycosphingolipid SSEA4 were higher in the SEVs derived from sALS patient compared to CTRLs.

**Summary/Conclusion**: Endosome tetraspanins CD9 and CD63 decrease in both LEVs and SEVs in accordance with the autophagy‐endolysosomal system dysregulation previously described in ALS (Webster et al., 2016). On the other hand, we identified higher expression of exosome surface markers such as CD81 and molecules related to viral infection, such as HLA‐DR, molecules presenting the antigens to T cells and less T‐cell antigens on SEVs from ALS patients (Martínez‐González et al. 2020). LEVs from ALS patients have less cell adhesion markers of platelets and endothelial cells and this is in line with the literature (Shrivastava et al., 2011). These data suggest that LEVs and SEVs carry different surface markers which might discriminate their role in ALS pathogenesis.

### PS14.05 Expression of specific tumor markers on extracellular vesicles in renal cell carcinoma cell culture and patient samples

Dirk Himbert, Saarland University

Philip Zeuschner, Department of Urology and Pediatric Urology, Saarland University

Hiresh Ayoubian, Department of Urology and Pediatric Urology, Saarland University

Michael Stöckle, Department of Urology and Pediatric Urology, Saarland University

Kerstin Junker, Department of Urology and Pediatric Urology, Saarland University

**Introduction**: Kidney cancer is the third most common cancer type. Extracellular vesicles (EVs) secreted by tumor tissues represent a new class of biomarkers. However, tumor released EVs represent only a small fraction in body fluids. Therefore, enrichment of tumor EVs based on tumor‐specific biomarkers could improve its diagnostic utility. The aim of this study is to identify and to prove tumor specific EV markers in clear cell renal cell carcinoma (ccRCC).

**Methods**: EVs from RCC cell lines (786‐O, RCC53, Caki1, Caki2) and 10 tumor tissues samples were isolated using ultracentrifugation (UC) or with UC and a three‐layer‐gradient (0.6 M, 1.3 M, 2.5 M sucrose). Protein expression of cell‐specific (GM130), exosome‐specific (CD9, CD63, CD81, Syntenin) and tumor cell‐specific markers (EpCAM, CA9, CD70, CD147) was analyzed via Westernblot and immunohistochemistry (IHC). Nanotracking analysis and transmission electron microscopy were used for EV characterization. By using nano‐chip technology (NanoViewBiosciences) the distribution and co‐expression of selected markers on EVs of cell culture and patient plasma samples was analysed.

**Results**: We successfully isolated EVs from cell culture supernatant and tumor tissues with sufficient concentration and purity. EpCAM was found to be weakly expressed in cells and EVs from cell cultures, and was expressed in 5/8 cellular and EV fractions of tissue samples. Expression of CA9 was observed in all cell lines, their EVs as well as in all primary tumor tissues and their EVs. CD70 exhibited different patient specific expression patterns in tumor cells and their EVs, but was absent in cell culture EVs. High amounts of CD147 were present on EVs compared to the corresponding parental cell lines, as well as to the cell lysates and EVs in all primary tumor tissue samples. Chip‐analysis of EVs from cell culture and patient derived plasma samples proved the coexpression of CD147 with distinct exosomal markers and a lower concentration compared to exosomal markers.

**Summary/Conclusion**: We established an effective technique to isolate EVs directly from human tissue samples with high purity and high concentration. CA9, CD70 and CD147 could represent promising tumor specific biomarkers for EVs in RCC. Further investigations will focus on the development of a bead‐based technique for enrichment of tumor specific EVs and the investigation of the co‐expression of tumor markers and exosome markers by using new high‐resolution techniques.

### PS14.06 Proteomic profiling of tissue‐derived extracellular vesicles from human pancreatic tumors

Nasibeh Karimi, Krefting Research Centre, Institute of Medicine at Sahlgrenska Academy, University of Gothenburg, Gothenburg, Sweden

Caroline Vilhav, Department of Surgery, Institute of Clinical Sciences, Sahlgrenska Academy at the University of Gothenburg, Sahlgrenska University Hospital, Gothenburg, Sweden.

Cecilia Lässer, PhD, University of Gothenburg

Jan Lötvall, Krefting Research Centre, Institute of Medicine at Sahlgrenska Academy at the University of Gothenburg, Gothenburg, Sweden.

**Introduction**: Pancreatic cancer is a highly metastatic cancer and one of the major causes of cancer mortality due to its poor prognosis and lack of effective treatments. Profiling of the protein and RNA cargo of EVs has shown promising results in identifying potential diagnostic and prognostic markers and uncovering mechanisms of cancer. In this study, we hypothesized that tissue‐derived EVs may identify candidate biomarkers leading to the development of a non‐invasive diagnostic tool for the early detection of pancreatic cancer.

**Methods**: Tumor tissue and non‐tumor tissue were excised from 3 pancreatic cancer patients. The tissue was sliced into small pieces and incubated with DNase 1 and Collagenase D in cell culture medium for 30 minutes at 37°C (Crescitelli et al JEV 2020). Tissue‐derived EVs were then isolated from the media by using ultracentrifugation and an iodixanol density cushion. Isolated EVs were then analysed using quantitative mass spectrometry analysis.

**Results**: In total 6114 proteins were quantified in all samples. Of these proteins, 1010 were differentially expressed between non‐tumor tissue‐derived EVs and tumor tissue‐derived EVs. In total, 837 proteins were significantly upregulated, and 173 proteins were downregulated in tumor tissue‐derived EVs compared to non‐tumor tissue‐derived EVs (p < 0.05; fold change > 1.5). The analysis showed that Common EV markers including CD63, CD81, CD9, and TSG101 were present in both non‐tumor tissue‐derived EVs and tumor tissue‐derived EVs. Gene ontology analysis indicated that “Extracellular Exosome” was the top GO term associated with the proteins identified in both non‐tumor tissue‐derived EVs and tumor tissue‐derived EVs. Moreover, subcellular localizations of quantified proteins showed a major distribution to the membrane which verifies our previous finding (Crescitelli et al JEV 2020).

**Summary/Conclusion**: Our results showed that isolation and characterization of EVs directly from pancreatic tissue is practical and might show to be a valued source of potential biomarkers of pancreatic cancer.

### PS14.07 Surface marker characterization of extracellular vesicles isolated from lymphatic exudate from breast cancer patients

Karin Ekström, Department of Surgery, Institute of Clinical Sciences, Sahlgrenska Academy at the University of Gothenburg, Department of Surgery, Sahlgrenska University Hospital, Region Västra Götaland, Wallenberg Centr

Rossella Crescitelli, Sahlgrenska Cancer Centre, Department of Surgery, Institute of Clinical Sciences, Sahlgrenska Academy, University of Gothenburg

Hafsteinn Ingi Pétursson, Department of Surgery, Institute of Clinical Sciences, Sahlgrenska Academy at the University of Gothenburg, Department of Surgery, Sahlgrenska University Hospital, Region Västra Götaland

Junko Johansson, Department of Surgery, Institute of Clinical Sciences, Sahlgrenska Academy at the University of Gothenburg, Department of Surgery, Sahlgrenska University Hospital, Region Västra Götaland, Wallenberg Centr

Cecilia Lässer, PhD, University of Gothenburg

Roger Olofsson Bagge, Department of Surgery, Institute of Clinical Sciences, Sahlgrenska Academy at the University of Gothenburg, Department of Surgery, Sahlgrenska University Hospital, Region Västra Götaland, Wallenberg Centr

**Introduction**: Breast cancer is the most common diagnosed cancer and the leading cause of cancer‐related deaths among females world‐wide. Recent research suggests that extracellular vesicles (EVs) play a major role in breast cancer. Axillary lymph node dissection (ALND) is a procedure for patients with lymph‐node metastasis, where large amounts of serous fluid are produced from the axilla post‐operatively. The overall aim was to isolate and characterize EVs from axillary serous fluid, and more specifically to determine if potential biomarkers could be identified and whether there is a difference in EV protein profile between patients with Her2+ and Her2‐ tumors.

**Methods**: Lymphatic drain fluid was collected from 7 breast cancer patients the day after ALND. The fluid was centrifuged to deplete cells and debris prior to EV isolation using qEV10/35 nm size‐exclusion chromatography (SEC) isolation. EVs were quantified and detected, and EV and contamination markers were evaluated using nanoparticle tracking analysis, electron microscopy and western blot. The expression of 37 EV surface proteins was evaluated by flow cytometry using the MACSPLEX Exosome kit.

**Results**: EVs in the size range of 30 to 100 nm were isolated from the 7 breast cancer patients (7.6E11 +/‐ 1.9E11 particles per ml). The isolated EVs were positive for the typical EV markers CD9, CD63, CD81 and Flotillin‐1 while albumin was absent, indicating low contamination from blood proteins. In total, 24 different EV surface proteins were detected. Eleven of those proteins were detected in all patients, including the common EV markers CD9, CD63 and CD81, cancer‐related markers CD24, CD29, CD44 and CD146, platelet markers CD41b, CD42a and CD62p as well as HLA‐DR/DP/DQ. Furthermore, CD29 and CD146 were enriched in Her2+ patients compared to patients with Her2‐ tumors.

**Summary/Conclusion**: Lymphatic drainage exudate retrieved from breast cancer patients after surgery contains EVs that can be isolated using SEC isolation. The EVs show several cancer‐related markers including CD24, CD29, CD44 and CD146, proteins of potential interest as biomarkers. These findings are of potential interest but needs to be investigated further in a larger study.

### PS14.08 Extracorporeal photopheresis modulates extracellular vesicle concentration and antigen profile in lung transplantation patients

Mario Barilani, Fondazione IRCCS Cà Granda Ospedale Maggiore Policlinico

Valeria Peli, Fondazione IRCCS Cà Granda Ospedale Maggiore Policlinico

Paolo Manzini, Fondazione IRCCS Cà Granda Ospedale Maggiore Policlinico

Giuseppe Buono, IRCCS Multimedica

Ilaria Righi, Fondazione IRCCS Cà Granda Ospedale Maggiore Policlinico

Lorenzo Rosso, Fondazione IRCCS Cà Granda Ospedale Maggiore Policlinico

Mario Nosotti, Fondazione IRCCS Cà Granda Ospedale Maggiore Policlinico

Lorenza Lazzari, Fondazione IRCCS Cà Granda Ospedale Maggiore Policlinico

**Introduction**: Lung transplantation (LuTx) is the last therapeutic option for end‐stage pulmonary failure, when other treatments are no longer effective. Yet, clinical complications may rise: acute rejection is common during the first year after LuTx and can trigger chronic rejection, the leading cause of late morbidity and mortality of LuTx. Extracorporeal photopheresis (ECP) is a promising treatment for chronic rejection, but its mechanism of action is not fully resolved, even though massive apoptosis and consequent immunomodulatory effects have been observed. Therefore, we focused our attention on extracellular vesicles (EV) as cell‐to‐cell communication vectors possibly involved in ECP‐triggered cellular events and as innovative biomarkers to assess lung quality and to monitor organ engraftment.

**Methods**: Two cohorts of cystic fibrosis patients receiving (n = 12) or not (n = 12) ECP will be enrolled in the study. Currently, n = 7 ECP‐treated patients (ECP group) and n = 11 not treated patients (control group) have been included in the study. Nanoparticle tracking analysis (Nanosight NS300, Malvern) and MACSplex bead‐based assay were applied to analyse bronchoalveolar lavage and peripheral plasma extracellular vesicles (EV).

**Results**: The presence of nanoparticles (NP) of different sizes was detected in bronchoalveolar lavage (BAL) and plasma of both donors (n = 17) and recipients (n = 17). The NP showed highly polydispersed size distributions in a 50–1000 nm range, compatible with a mixed population of small and large EV. Recipient BAL NP mean size of the control group was significantly reduced from 278±51 at 6 days post‐transplant (dpt) to 181±21 at 17 dpt.

Different kinetics of NP production were observed in the recipients (10^8‐10^14 particles/mL range). BAL control samples showed a peak of NP release at 6 dpt that was significantly reduced in the ECP group and at 10 dpt. Plasma samples showed a general increasing trend for NP release over time that reached significance at 17 dpt only for the control group.

The expression of EV‐associated markers CD63, CD9 and CD81 was detected, further suggesting that the analysed NP are bona fide EV. Significant modulations of surface antigens involved in the immune response were observed in the ECP group: 1) upregulation of CD29 (integrin subunit beta 1), CD62P (activated platelet and endothelial cell receptor) and CD31 (platelet/endothelial cell adhesion molecule 1) at 6 dpt; 2) downregulation of CD49e (integrin subunit alpha 5) and CD41b (integrin subunit alpha 2b) at 20 dpt.

**Summary/Conclusion**: Correlation with lung transplantation outcome will be evaluated at the conclusion of the study. Further studies need to be performed to validate the EV nature of the herein analysed NP and to better define their role in the context of ECP prophylactic therapy for lung transplantation.

### PS14.09 Comprehensive proteomic profiling of plasma extracellular vesicles via SWATH mass spectrometry determines biomarkers for GBM progression

Susannah M. Hallal, Neurosurgery Department, Chris O ' Brien Lifehouse, Camperdown, NSW

Agota Tuzesi, Department of Neuropathology, Royal Prince Alfred Hospital, Camperdown, NSW

Heng Wei, Department of Neuropathology, Royal Prince Alfred Hospital, Camperdown, NSW

Maggie Lee, Department of Neuropathology, Royal Prince Alfred Hospital, Camperdown, NSW

Hao‐Wen Sim, Department of Medical Oncology and NHMRC Clinical Trials Centre, Chris O'Brien Lifehouse, Camperdown, NSW

Brindha Shivalingam, Neurosurgery Department, Chris O'Brien Lifehouse, Camperdown, NSW

Michael Buckland, Department of Neuropathology, Royal Prince Alfred Hospital, Camperdown, NSW

Kimberley Alexander(Kaufman), PhD, Neurosurgery Department, Chris O'Brien Lifehouse, Camperdown, NSW

**Introduction**: Glioblastoma (GBM) is the most common and severe adult primary brain tumour. GBMs often recur quickly, developing more aggressive and treatment‐resistant characteristics, and current monitoring methods can be insensitive to GBM progression. The availability of a liquid biopsy (i.e. blood test) that could measure GBM molecules in body fluids and assess tumour evolution in real‐time has great potential to enhance patient care. In this regard, circulating extracellular vesicles (EVs) are promising biomarkers that are readily accessible in the blood of GBM patients. Despite their suitability, in‐depth proteomic characterisation of circulating EVs by traditional shot‐gun proteomic methods has been hindered by the complexity of the blood. Recently, glioma plasma‐EV proteomes were comprehensively analysed using a data‐independent acquisition (DIA) mass spectrometry (MS) platform, SWATH‐MS, and the plasma‐EV proteomes were capable of discriminating glioma patients by subtype/grade.

**Methods**: EVs were isolated by size exclusion chromatography from plasma sampled serially from 35 GBM patients at multiple time points over the course of their tumour (pre‐operative, post‐operative and recurrence; n = 81), metastatic brain tumours (n = 21) and age/gender matched healthy controls (n = 22). Nanoparticle tracking and transmission electron microscopy confirmed the isolation of small‐EV subtypes (< 200 nm). The plasma‐EV peptides were sequenced by SWATH‐MS, and protein identities and quantities were extracted using a custom spectral library comprised of 8662‐protein species.

**Results**: A total of 3278 proteins were identified in the plasma‐EVs across the sample groups. Significantly changing proteins across the GBM and controls (p < 0.05) included proteins previously reported to have significance in GBM‐EVs. PCA showed excellent discrimination between the patient groups, samples clustered with their respective cohorts, and plasma‐EVs resampled at recurrence grouped with more aggressive samples.

**Summary/Conclusion**: Using SWATH‐MS we have comprehensively profiled GBM plasma‐EV proteomes and showed that plasma‐EV markers can predict GBM tumour progression. Future studies using larger independent cohorts could validate these findings and determine a set of circulating‐EV markers that can stratify GBM patients and predict recurrence, progression and treatment resistance.

### PS14.10 Expression of PD‐L1 on extracellular vesicles from renal cell carcinoma cell lines

Greta Jaschkowitz, Department of Urology and Pediatric Urology, Saarland University, Homburg/Saar, Germany

Philip Zeuschner, Department of Urology and Pediatric Urology, Saarland University

Dirk Himbert, Saarland University

Angela Zaccagnino, Department of Urology and Pediatric Urology, Saarland University

Michael Stöckle, Department of Urology and Pediatric Urology, Saarland University

Elfriede Noessner, Helmholtz Zentrum München, German Research Center for Environmental Health, IMA Immunoanalytics Research Group

Kerstin Junker, Department of Urology and Pediatric Urology, Saarland University

**Introduction**: Checkpoint inhibitors have changed the current therapeutic landscape for many malignant tumors including renal cell carcinoma (RCC). The interaction between PD‐1/PD‐L1 has been of particular interest and the expression of PD‐L1 on extracellular vesicles (EVs) has been shown to carry prognostic, diagnostic and predictive value in various cancers. However, its role in RCC has not yet been fully explored. This study examined the PD L1 expression in six RCC cell lines and their corresponding EVs in vitro.

**Methods**: PD‐L1 expression was examined in six renal cell carcinoma cell lines (Caki1, Caki2, 786‐O, RCC26, RCC53, KTCTL26). EVs were isolated from cell culture supernatants by serial ultracentrifugation. The cells were stimulated once or two times before EV isolation with 100 or 200ng/ml interferone gamma (IFNγ) over 48h. The PD‐L1 expression of the EVs and cell lysates was compared by Western Blot. Quality and quantity of EV isolation was proven by transmission electron microscopy, Nano Tracking Analysis and Western Blot.

**Results**: All six RCC cell lines expressed PD‐L1. RCC53 had the highest expression in unstimulated cell lines, 786‐O and KTCTL26 the lowest. In the corresponding EVs, the PD‐L1 expression was lower and similar between all cell lines. One‐time stimulation was insufficient for induction of PD‐L1 expression in cells and corresponding EVs. Repetitive stimulation with IFNγ increased PD‐L1 both in the cells and EVs of RCC53, RCC26, 786‐O and KTCTL26, but not in Caki1 and Caki2. 786‐O had the strongest increase of PD‐L1 expression. Between the two varying concentrations of IFNγ, no difference in induction efficiency was observed.

**Summary/Conclusion**: RCC cell lines express PD‐L1 in cells and their secreted EVs. The expression of PD‐L1 is lower in the EVs, but can be increased by repetitive stimulation with IFNγ in a cell‐line dependent manner. Hence, this experimental design can help to investigate the role of PD‐L1 on tumor EVs in communication with immune cells and their prognostic and predictive potential.

### PS14.11 Identification of Potential Aetiopathological Pathways in Early Onset Preeclampsia (EOPE) through Cluster Analysis of Medium/Large Syncytiotrophoblast Membrane Extracellular Vesicle's Protein‐Protein Interaction Network

Toluwalase Awoyemi, MB;BS, NDWRH, University of Oxford

Maryam Rahbar, NDWRH, University of Oxford

Adam Cribbs, NDORMS, University of OXford

Wei Zhang, NDWRH, University of Oxford

Chris Redman, NDWRH, University of OXford

Manu Vatish, NDWRH, University of OXford

**Introduction**: Preeclampsia is a pregnancy related disease of dire maternal and foetal consequences. It is diagnosed between 20 to 34 weeks’ gestation and characterised by elevated blood pressure and end organ damage. The placenta releases soluble factors and syncytiotrophoblast membrane extracellular vesicles (STBEV's) which help propagate the disease. Attempts have been made to characterise the cargoes of STBEV's in EOPE and normal pregnancy but typically, these differentially expressed proteins are explored in isolation as biomarkers when the interaction between these proteins is equally fascinating. We hypothesized that DEPs between EOPE and normal pregnancy (NP) interact with themselves and that micro‐clusters exists within this interactive network that may help identify possible or additional pathogenetic mechanisms in EOPE.

**Methods**: The protein‐protein interaction network of differentially expressed medium/large STBEV proteins between 8 EOPE and 6 normal pregnancy patients in our previous study (unpublished) were constructed using the String plug‐in and visualised with Cytoscape 3.0. The main network component was further analysed following removal of orphan proteins with the MCODE algorithm. The top 3 clusters in this network were identified and then their function ascertained with the CLUEGO plug in.

**Results**: Analysis of the largest micro‐cluster identified proteins revealed perturbed biological processes that cut across the following themes 1) response to hypoxia and oxidative stress 2) protein catabolism and folding 3) glucose and carbohydrate metabolism 4) transcription initiation, among others. Some of the results corroborate existing theories in EOPE and reflect clinical and biochemical observations that exist in EOPE patients.

**Summary/Conclusion**: Cluster analysis of molecular networks offer systems level I biological insight and ascribes potential biological information to differentially expressed proteins (DEPs). This along with the potential of EVs to serve as circulating biomarkers creates a unique way to identify common and patient specific dysfunctional pathways in EOPE in tissues/organs traditionally difficult to access. This can be potentially explored to personalise treatment and management of groups of patients with EOPE.

### PS14.12 Interleukin Detection in Umbilical Cord‐derived Mesenchymal Stem Cell Secretome

Angliana Chouw, Prodia StemCell Indonesia

Tiana Milanda, Universitas Padjajaran

Ahmad Faried, Universitas Padjajaran

Cynthia Retna Sartika, Prodia StemCell Indonesia

**Introduction**: Mesenchymal stem cell (MSC)‐derived secretome has gained many attention in the field of regenerative medicine. This due to the complex bioactive molecule that have the ability to repaired the damage tissue and promote regeneration. Several clinical trials have reporter the positive effect in treating the inflammatory disorder. The bioactive molecules secreted by MSC composed of cytokine, chemokines, growth factor, protein and extracellular factos. The MSC‐derived secretome could be collected during in vitro culture. In this study, we evaluated the pro‐ and anti‐inflammatory interleukin (IL) concentration in Umbilical cord (UC) MSC‐derived secretome.

**Methods**: UCMSC were culture in a normoxic conditioned until it reach 80% confluency using DMEM High Glucose and supplemented with 5% Human Platelet Lysate. Upon it reached the confluency, growth medium were discarded and change with serum free media and incubated for 24 hours. Medium were collected and centrifuge for 500xg for 5 minutes and filtered using 0.2μm filter membrane. The protein concentration were standardized using BCA Protein Assay before detecting the concentration using MACSPlex Cytokine 12 kit.

**Results**: Pro‐inflammatory such as IL‐5, IL‐6, IL‐9, IL‐12, and IL‐17A, with concentration also anti‐inflammatory interleukin such IL‐2, IL‐4, and IL‐10 are detected. The highest and the lowest concentration detected are IL‐6 (50,858±3,610 ng/mL) and IL‐4 (469±45 ng/mL), respectively. The high concentration of IL‐6 is due to the multifunction of IL‐6 which involved in inflammation response and metabolic regulation. IL‐4 acts as anti‐inflammatory factor which activate the T‐helper 2 cells.

**Summary/Conclusion**: Pro‐ and anti‐inflammatory interleukins were found in UCMSC‐derived secretome with different concentration.

### PS14.13 Screening for CD117 (KIT) on ovarian cancer EVs as a potential target of binase –based anticancer therapy

Irina Yu. Petrushanko, Engelhardt Institute of Molecular Biology, RAS, Moscow, Russia

Ekaterina I. Borovkova, Pirogov Russian National Research Medical University, Moscow, Russia

Vladimir A. Mitkevich, Engelhardt Institute of Molecular Biology, RAS, Moscow, Russia

Victoria O. Shender, Federal Research and Clinical Center of Physical‐Chemical Medicine of Federal Medical Biological Agency, Moscow, Russia

Polina V. Shnaider, Federal Research and Clinical Center of Physical‐Chemical Medicine of Federal Medical Biological Agency, Moscow, Russia

Alexander A. Makarov, Engelhardt Institute of Molecular Biology, RAS, Moscow, Russia

Elena Khomyakova, Genopole Shaker

**Introduction**: Ovarian cancer is the most lethal gynecological malignancy. Most of ovarian cancer deaths, particularly for patients with high grade serous (HGS) ovarian cancers, results from the metastatic propagation of drug‐resistant cells. According to stem cell concept of carcinogenesis, ineffectiveness of standard therapies is attributed to subpopulation of self‐renewing, drug‐resistant cell population ‐ cancer stem cells (CSCs). Mast/stem cell growth factor receptor CD117 (KIT), was demonstrated to be one of the main CSCs markers expressed in aggressive ovarian tumors. Earlier we've demonstrated that sensitivity of tumor cells to ribonuclease binase toxic effect depends on the level of CD117. Here we analyze the correlation between CD117 expression and tumor histological type on different cell lines and on EVs isolated from urine of ovarian cancer patients. Moreover, the effect of binase treatment on cell viability and EVs production was studied on cell lines with different CD117 status.

**Methods**: Urine samples from ovarian cancer patients were collected before treatment. The EVs were isolated by ultrafiltration. Pre‐purified EVs were incubated with anti CD9 magnetic beads following by CD81‐PE and CD117‐PE staining. Cells were treated by binase. Analysis of cells and cell cultures derived EVs were performed by flow cytometry.

**Results**: The pilot study demonstrated that CD117 is detectable exclusively on urinary EVs of the patients with HGS ovarian cancers. It was found that binase affects the level of EVs in cells with different expression of CD117 and changes the level of KIT and nucleic acids in them.

**Summary/Conclusion**: Binase action leads to the loss of “communication channels” by the cancer cell with the microenvironment, which will reduce the risk of tumor spread. We are discussing the possible application of binase in the treatment of high aggressivity CD117 positive ovarian cancers and EVs as a marker of cancer diagnostics and effectives of therapy.

### PS14.14 Proteomic and lipidomic profiling of extracellular vesicles isolated from cells grown in high and normal density conditions

Olesia Gololobova, Johns Hopkins University School of Medicine

Marina Sokolsky‐Papkov, Center for Nanotechnology in Drug Delivery, Eshelman School of Pharmacy, University of North Carolina at Chapel Hill

Alexander Kabanov, Center for Nanotechnology in Drug Delivery, Eshelman School of Pharmacy, University of North Carolina at Chapel Hill

**Introduction**: Extracellular vesicles (EVs) have attracted a lot of attention as drug/gene delivery carriers. However, EV production is difficult to scale up. One of the potential directions to increase vesicles yield is to use cell bioreactors (BR) which can provide reproducible and standardized cell products.

**Methods**: IC21 macrophages were cultured in RPMI 1640 medium supplemented with 10% EV depleted FBS in DWK Life Sciences Wheaton bioreactors (high density condition) or T175 flasks (normal density condition). EVs were isolated by differential centrifugation. Vesicles were characterized using nanoparticle tracking analysis, single‐particle zeta potential, transmission electron microscopy, Simple Western blot, lipidomic and proteomic profiling.

**Results**: We show that significantly more EVs can be isolated from IC21 cells cultured in BR, and these vesicles are smaller in size. No differences were observed in particle's charge and phenotype. Simple Western blot revealed that bioreactor EVs had 4 times higher abundant of HSP90 beta and twice lower expression of CD9. Notably, there is a variation in lipid composition and content between two groups of EVs. Some sphingolipids and glycerophospholipids are more expressed in flask vesicles and PG 16:0_16:0 and PI 18:0_16:1 are not detected in BR EVs. 950 unique EV‐associated proteins were identified and compared. Vesicles isolated from bioreactor cultured cells showed higher abundance of proteins like Flottilin‐1, HSP‐90a and b, and CD63. Low abundant contaminant proteins (Calnexin, Histones) were detected in EVs isolated from both flask and BR, however no significant difference in their levels was observed.

**Summary/Conclusion**: Cells grown in bioreactors can be promising source to scale up the EV production, showing higher yield and more uniform and reproducible outcome. However, smaller size and differences in lipidomic and proteomic profiles can suggest the importance of careful and full characterization of EVs.

## PS15 Nucleic Acid Biomarkers

Chair: Navneet Dogra, Icahn School of Medicine at Mount Sinai, United States

Chair: Tom Driedonks, Johns Hopkins Medical School / Utrecht University, United States

### PS15.01 Circulating serum exosomal long non‐coding RNAs FOXD2‐AS1, NRIR, and XLOC_009459 as diagnostic biomarkers for colorectal cancer

Miao Yu, Department of Clinical Laboratory, Shandong Cancer Hospital and Institute, Shandong First Medical University and Shandong Academy of Medical Sciences

Xingguo Song, Department of Clinical Laboratory, Shandong Cancer Hospital and Institute, Shandong First Medical University and Shandong Academy of Medical Sciences

Xianrang Song, Department of Clinical Laboratory, Shandong Cancer Hospital and Institute, Shandong First Medical University and Shandong Academy of Medical Sciences

Li Xie, Department of Clinical Laboratory, Shandong Cancer Hospital and Institute, Shandong First Medical University and Shandong Academy of Medical Sciences

**Introduction**: Exosomes derived from cancer cells encapsulate various kinds of tumor‐specific molecules, thus can interact with adjacent or distant cells to mediate information exchange. Long non‐coding RNAs (lncRNAs) in exosomes have the potential as diagnostic and prognostic biomarkers in different types of cancers. The current study was aimed to identify circulating exosomal lncRNAs for the diagnosis of colorectal cancer (CRC).

**Methods**: Exosomes were isolated from the serum by ultracentrifugation and verified by transmission electron microscope (TEM), qNano and immunoblotting. Exosomal lncRNAs FOXD2‐AS1, NRIR, and XLOC_009459 were selected by lncRNA microarray and validated by qPCR in 203 CRC patients and 201 healthy donors. The receiver operating characteristic curve (ROC) was used to assess the diagnostic efficiency of serum exosomal lncRNAs.

**Results**: Exosomal FOXD2‐AS1, NRIR, and XLOC_009459 (TCONS_00020073) levels were significantly upregulated in 203 CRC patients and 80 early‐stage CRC patients compared to 201 healthy donors, possessing the area under the curve (AUC) of 0.728, 0.660 and 0.682 for CRC, as well as 0.743, 0.660 and 0.689 for early‐stage CRC, respectively. Notably, their combination demonstrated the markedly elevated AUC of 0.736 for CRC and 0.758 for early‐stage CRC, indicating their potential as diagnostic biomarkers for CRC.

**Summary/Conclusion**: Our data suggested that exosomal lncRNAs FOXD2‐AS1, NRIR and XLOC_009459 act as the promising biomarkers for the diagnostics of CRC and early‐stage CRC.

### PS15.02 Comparative analysis of plasma and urinary EV RNA content in prostate cancer patients

Cristina Bajo‐Santos, Latvian Biomedical Research and Study Centre

Pawel Zayakin, Latvian Biomedical Research and Study Centre

Juris Jansons, Riga East Clinical University Hospital

Alberts Belovs, Riga East Clinical University Hospital

Mikus Melderis, Latvian Biomedical Research and Study Centre

Vilnis Lietuvietis, Riga Stradins University

Aija Linē, Latvian Biomedical Research and Study Centre

**Introduction**: Recently, EVs have emerged as a very attractive source of cancer‐derived RNA biomarkers for the early detection, prognosis and monitoring of cancer progression. However, biofluids contain a complex mixture of EVs derived from various tissues and to what extent their RNA cargo reflects the RNA content of cancer cells is unknown. Moreover, a systematic comparison of RNA cargo in blood and urinary EVs has not been reported before.

**Methods**: EVs were isolated from plasma and urine samples collected before and after the surgery from 10 patients with prostate cancer (PC) and RNAseq libraries were prepared in duplicates. A total of 80 small RNA libraries were constructed from the EV‐enclosed RNA. In addition, 40 small RNA and 40 transcriptome libraries were constructed from paired PC and normal prostate tissues. All libraries were sequenced using Illumina's NextSeq instrument. RNAseq data were analysed using an in‐house software tool.

**Results**: The most common RNA types in EVs were miRNAs, piRNAs, tRNAs, lncRNAs and mRNAs. The majority of RNAs that were overexpressed in the tumour tissues were detectable in the pre‐operation urinary and/or plasma EVs. The levels of 63 mRNAs, 3 lncRNAs, 2 miRNAs and 1 piRNA were significantly decreased in the post‐operation urinary EVs thus suggesting that the main tissue source of these RNAs in urine is PC and/or normal prostate tissues. These RNAs represent biomarker candidates for the development of liquid biopsies for the detection or active surveillance of PC. No such RNA biomarkers were identified in plasma EVs. Moreover, both plasma and urinary EVs contained a substantial fraction of exogenous RNAs that are mapped to various microbial genomes thus possibly representing a human microbiome.

**Summary/Conclusion**: This study provided new insight into the composition of RNA cargo in plasma and urinary EVs and its correlation with PC tissues and revealed a number of RNA biomarkers for the detection and active surveillance of PC.

### PS15.03 Mitochondrial DNA diversity of plasma extracellular vesicles from rectal cancer patients

Tonje Bjørnetrø, Akershus University Hospital

Paula A Bousquet, Department of Oncology, Akershus University Hospital, Lørenskog, Norway

Kathrine Røe Redalen, Department of Physics, Norwegian University of Science and Technology, Trondheim, Norway

Sebastian Meltzer, Department of Oncology, Akershus University Hospital, Lørenskog, Norway

Anne Hansen Ree, Department of Oncology, Akershus University Hospital, Lørenskog, Norway

**Introduction**: Mitochondria‐derived cell free DNA has been found in the blood of healthy subjects and patients with various diseases, especially cancer. The entire mitochondrial DNA (mtDNA) genome can be packed inside extracellular vesicles (EVs). We aimed to compare EV‐mtDNA to whole blood (WB)‐mtDNA and examine how EV‐mtDNA may relate to patient outcome in rectal cancer.

**Methods**: In a prospective biomarker study (approved by designated authorities; informed consent obtained), WB and plasma were collected from 54 rectal cancer patients at the time of diagnosis. Plasma EVs were isolated by size exclusion chromatography, measured by nanoparticle tracking analysis, and treated with DNase and proteinase. DNA was isolated from EVs and WB and mtDNA analyzed by next generation sequencing. Patients that received treatment with curative intent were followed for progression‐free survival (PFS). Estimated 5‐year PFS was calculated from the time of study enrolment to the date of recurrent disease. P‐values less than 0.05 were considered statistically significant.

**Results**: The size of the EVs increased with more advanced disease stage, and stage IV patients had higher plasma concentration of EVs than stage I cases. Mutant mtDNA copies are often mixed with wild‐type copies (heteroplasmy), and EVs displayed significantly higher number of total mtDNA variants and more heteroplasmy compared to WB. Specifically, EVs contained significantly fewer synonymous mtDNA variants and more missense variants than WB. A high degree of EV heteroplasmy was associated with poor PFS, whereas WB heteroplasmy was not.

**Summary/Conclusion**: Compared to WB, plasma EVs from rectal cancer patients contained higher number of total mtDNA variants and more heteroplasmy and missense substitutions. Patients with high EV‐mtDNA heteroplasmy had increased risk of PFS events, most being metastatic progression.

### PS15.04 The potential value and mechanism of exosomal hsa_circ_0000670 as a diagnotic marker for gastric cancer

yan wang, Jiangsu Key Laboratory of Medical Science and Laboratory Medicine, School of Medicine, Jiangsu University, Zhenjiang, Jiangsu 212013, China

xiaojuan huang, Jiangsu Key Laboratory of Medical Science and Laboratory Medicine, School of Medicine, Jiangsu University, Zhenjiang, Jiangsu 212013, China

Wenrong Xu, Jiangsu University

huiting wang, Jiangsu Key Laboratory of Medical Science and Laboratory Medicine, School of Medicine, Jiangsu University, Zhenjiang, Jiangsu 212013, China

Hui Qian, jiangsu university

**Introduction**: Exosomal circRNAs play crucial roles in regulating the crosstalk of cancer cells in the tumor microenvironment. However, the underlying mechanisms by which exosomal hsa_circ_0000670 (circ670) contributes to gastric cancer (GC) are unknown.

**Methods**: The expression of GC serum and cells exosomal circ670 were detected by qRT‐PCR. Functional assays and co‐culture of GC cells and exosomes were used to evaluate the impact of exosomal circ670. Bioinformatic analysis and luciferase reporter assay were used to predict and validate miRNA and protein binding to circ670.

All patients gave their written informed consent to participate in this study. And the study was approved by the Medical Ethics Committee of Jiangsu University ((IRB protocol number: 2020161).

**Results**: The expression of circ670 was increased in GC serum, cells and their exosomals. And the area under ROC curve of GC serum‐derived exosomes was better than that of serum. Tissue and cellular FISH experiments demonstrated that circ670 was mainly localized in the cytoplasm. Exosomal circ670 promoted the proliferation, migration and stemness of GC cells. Exosomal circ670 acted as the sponge for miR‐4659a/b in GC, and it had the binding site to Lin28A.

**Summary/Conclusion**: Exosomal circ670 was a tumor promoter by sponging miR‐4659a/b and combining Lin28A to promote the proliferation, migration and stemness in GC, and may provide a novel therapeutic target for GC.

### PS15.05 Discovery and validation of a urinary exosome mRNA signature for the diagnosis of human kidney transplant rejection

JAMES HURLEY, EXOSOME DIAGNOSTICS

Vasisht Tadigotla, Ph.D., EXOSOME DIAGNOSTICS

Rania El Fekih, Brigham and Women's Hospital

Areej Alghamdi, Brigham and Women's Hospital

John Choi, Brigham and Women's Hospital

Jamil Azzi, Brigham and Women's Hospital

Johan Skog, Ph.D., Exosome Diagnostics, Inc.

**Introduction**: Patients with end stage renal disease usually undergo transplantation, but acute rejection carries a risk of 10–15%. Current methods for monitoring clinical rejection, such as in serum creatinine and urinary protein excretion, may not reflect subclinical rejection. Similarly repeat protocol biopsy may result in increased complications and cost. An accurate non‐invasive method would allow for earlier diagnosis and guide clinical decision for treating transplant rejection. Extracellular vesicles such as exosomes, released from cells and carrying the parent cells’ surface proteins and nucleic acids, can be easily isolated and their nucleic acid profile interrogated. In the transplanted kidney, exosomes will originate from glomerular podocytes, renal tubular cells and from immune cells, generated during rejection, and, thus, reflect the state of the transplant.

**Methods**: Urine samples were collected from patients undergoing a transplant kidney biopsy for clinical indications. A total of 190 urine samples ‐ split between training (112) and validation (78) cohorts ‐ were collected. Exosomal RNA was isolated for expression profiling. Using the OpenArray(R) Real‐time PCR system, 112 mRNAs involved in inflammation response were analyzed. A subset of RNAs were identified and validated for the detection of overall transplant rejection and acute cellular rejection. Additionally, a potential signature for antibody mediated rejection was identified.

**Results**:: A multi‐gene signature was identified that differentiated patients with any‐cause rejection from no‐rejection, with AUCs of 0.838 for the training set and 0.689 in the validation set. Negative predictive values (NPV) were 87.17% and 77.5% respectively. A second signature for acute cellular rejection has AUCs of 0.879 (training) and 0.769 (validation), with NPVs of 93.9% and 93.9%. Finally, with a limited sample set, a gene signature for antibody mediated rejection (AMR) was identified with an AUC of 0.809 and NPV of 94.0%.

**Summary/Conclusion**: A non‐invasive, robust method of detecting kidney transplant rejection using urine exosomal RNA has been demonstrated. Further validation with a larger prospective cohort of samples is required, particularly for the AMR signature.

### PS15.06 Identification of RCC‐specific mRNA‐biomarkers using kidney cancer extracellular vesicles

Richard C. Zieren, Brady Urological Institute Johns Hopkins University School of Medicine

Liang Dong, MD, The Brady Urological Institute Johns Hopkins University School of Medicine

Kengo Horie, MD, PhD, The Brady Urological Institute Johns Hopkins University School of Medicine

Morgan Kuczler, The Brady Urological Institute Johns Hopkins University School of Medicine

Sarah Amend, The Brady Urological Institute Johns Hopkins University School of Medicine

Theo de Reijke, Department of Urology AmsterdamUMC University of Amsterdam

Kenneth Pienta, The Brady Urological Institute Johns Hopkins University School of Medicine

**Introduction**: The majority of renal cell carcinoma (RCC) patients are diagnosed before symptoms have occurred. The diagnostic process relies vastly on imaging modalities such as ultrasound, CT and MRI. Limited cancer‐specificity of biomarkers leads to overtreatment such as surgical resection of benign small renal masses. Liquid biopsy‐acquired extracellular vesicles (EVs) are a promising source of diagnostic biomarkers in cancer and other diseases. In kidney cancer research, only few studies describe EV biomarkers. Previously, we have optimized methods to obtain tumor‐specific EVs from RCC tissue. In this study, we aimed to discover EV‐based mRNA biomarkers in liquid biopsies using tissue‐derived RCC EVs.

**Methods**: Matched RCC tumor tissue, normal kidney tissue, plasma, and urine were collected from 12 patients undergoing radical nephrectomy. Informed consent was obtained from all patients. All procedures were performed in accordance with the Institutional Review Board. Tissue EVs were enriched using our previously validated workflow: a combination of differential centrifugation, filtration, and ultracentrifugation. EVs from plasma and urine were enriched by differential centrifugation, ultrafiltration, and size‐exclusion chromatography. Total RNA was extracted from EVs using the Qiagen miRNeasy micro kit. The NanoString nCounter low RNA input kit was used for RNA amplification, followed by mRNA‐analysis by the Nanostring nCounter PanCancer Progression assay. Expression data of 770 mRNAs were analyzed with the nSolver software.

**Results**: Subgroup analyses comparing tumor EVs with normal EVs show significantly different expression patterns. Upregulated and downregulated tumor‐specific mRNAs were compared with plasma EVs and urinary EVs, which enabled the selection of candidate liquid biopsy biomarkers for future validation. Additionally, Gene Set Enrichment Analysis was performed to assess functional pathways in these RCC EVs

**Summary/Conclusion**: Using tissue‐derived EVs, we found tumor‐specific EV mRNA expression profiles. Additionally, we selected genes that are potential biomarkers for RCC in plasma EVs and urinary EVs. Further validation of the most significantly up‐ and downregulated mRNAs in a large patient cohort is required in plasma EVs and urinary EVs.

### PS15.07 Multi‐omics of EV cargo from benign kidney EVs and kidney cancer EVs

Richard C. Zieren, Brady Urological Institute Johns Hopkins University School of Medicine

David Clark, Department of Pathology, The Johns Hopkins University

Liang Dong, MD, The Brady Urological Institute Johns Hopkins University School of Medicine

Leandro Ferreira Moreno, Laboratory for Experimental Oncology and Radiobiology & Center for Experimental and Molecular Medicine Amsterdam UMC University of Amsterdam

Morgan Kuczler, The Brady Urological Institute Johns Hopkins University School of Medicine

Kengo Horie, MD, PhD, The Brady Urological Institute Johns Hopkins University School of Medicine

Sarah Amend, The Brady Urological Institute Johns Hopkins University School of Medicine

Theo de Reijke, Department of Urology AmsterdamUMC University of Amsterdam

Kenneth Pienta, The Brady Urological Institute Johns Hopkins University School of Medicine

**Introduction**: Renal cell carcinoma (RCC) accounts for over 400,000 new cases and 175,000 deaths annually. Non‐invasive biomarkers are needed to distinguish benign from RCC in early stage renal masses and to distinguish clear cell RCC (ccRCC) from papillary RCC (pRCC) subtypes. Extracellular vesicles (EVs) are a promising source of biomarkers for RCC. Compared with EVs enriched from plasma and urinary, EVs enriched from conditioned cell media (CCM) are readily available and their cellular origin is specific. In this study we aimed to select potential biomarkers by analyzing mRNA and protein cargo of EVs from various RCC and immortalized benign kidney epithelial cell lines and assess biological functions of the EV cargo.

**Methods**: The following cell lines were used; 786O, 769P, and Caki1 (all ccRCC); ACHN, and Caki2 (all pRCC); and HK2, and RPTEC (benign kidney epithelial cells). CCM EVs were enriched by a combination of differential centrifugation, ultrafiltration, and size exclusion chromatography. All CCM EVs were counted by NanoFCM, negative stained and imaged by TEM, and assessed for EV‐markers (CD63, CD81, Flot1) by Western blot. The Qiagen miRNeasy kit was used to extract RNA from CCM EVs derived of 786O, 769P, ACHN, HK2, and RPTEC cells, The NanoString nCounter low RNA input kit was used for RNA amplification in combination with the Nanostring nCounter PanCancer Progression assay. EVs from 786O, 769P, ACHN, Caki1, Caki2, and HK2 were processed by Tandem Mass Tag mass spectrometry (TMT MS).

**Results**: Counts by NanoFCM demonstrated ACHN secreted the most EVs per million cells (1.4 ‐ 10e7) and Caki‐1 the least (5.6 ‐ 10e4) EVs per million cells. Western blot expression of EV‐associated markers CD81, CD63, CD9, and Flot1 was highly correlated with marker expression detected by TMT MS. In mRNA‐analyses we found abundance of 356 mRNAs in benign EVs, 335 in ccRCC EVs, and 268 in pRCC EVs. By MS analysis we identified 1,726 proteins of which 186 proteins were enriched (> 1.5‐fold change) in all types of EVs compared to their parental cell lysates. We found 83–124 proteins with increased abundance in pRCC EVs compared with benign EVs and 83–121 proteins with decreased abundance. In ccRCC EVs we found 95–141 increased abundance compared with benign EVs and 116–130 with decreased abundance.

**Summary/Conclusion**: We compared CCM EVs derived from ccRCC, pRCC, and benign renal cell lines with a multi‐omics approach. Although we used different experimental designs for the gene analysis and MS analysis due to technical limitations, nonetheless we were able to analyze cargos of EVs from various renal origins, cancer and benign. We identified several potential mRNA‐ and protein RCC‐biomarkers that require further clinical validation.

### PS15.08 Detection of ALK fusions in Extracellular Vesicles from non‐small cell lung cancer cells

Estela Sánchez‐Herrero, Hospital Universitario Puerta de Hierro‐Majadahonda

Carmen Campos‐Silva, MA, Immunology and Oncology Department, Spanish National Centre for Biotechnology (CNB‐CSC), Madrid, Spain

Yaiza Cáceres Martell, Immunology and Oncology Department, Spanish National Centre for Biotechnology (CNB‐CSC), Madrid, Spain.

Sandra Sanz‐Moreno, Liquid Biopsy Laboratory, Biomedical Sciences Research Institute Puerta de Hierro, Majadahonda, Spain

Roberto Serna‐Blasco, Liquid Biopsy Laboratory, Biomedical Sciences Research Institute Puerta de Hierro, Majadahonda, Spain.

Mariano Provencio, Medical Oncology Department, Hospital Puerta de Hierro, Majadahonda, Spain.

Mar Valés‐Gómez, Immunology and Oncology Department, Spanish National Centre for Biotechnology (CNB‐CSC), Madrid, Spain.

Atocha Romero, Liquid Biopsy Laboratory, Biomedical Sciences Research Institute Puerta de Hierro, Majadahonda, Spain.

**Introduction**: The detection of ALK‐fusions in NSCLC tumors identifies a subset of patients that benefit from targeted therapies. However, the availability of tumors biopsies is sometimes limited in the clinical setting. The analysis of Extracellular Vesicles (EVs) in liquid biopsy can potentially overcome such limitation.

**Methods**: H3122 and H2228 cell lines, derived from NSCLC patients with EML4‐ALK variant 1 (E13;A20) and variant 3 (E6a/b;A20) respectively, were cultured to isolate EVs by sequential centrifugation of cell culture supernatants. Morphology and size of the preparations enriched in EVs was assessed by transmission electron microscopy (TEM). In addition, specific EVs markers, namely CD63, CD81 and CD9, were measured by flow cytometry and Western‐Blot. EML4‐ALK variant proteins were analyzed by Western blot using 5A4 antibody, and their corresponding RNA by digital PCR (dPCR), using a QuantStudio(R) 3D Digital PCR System and specific TaqMan(R) assays for EML4‐ALK variant 1 and 3, and by next‐generation sequencing (NGS), using Oncomine™ Pan‐Cancer Cell‐Free Assay.

**Results**: EV preparations obtained from both cell lines, analyzed by TEM were spherical and around 100 nm. Flow cytometry and Western blot confirmed expression of the EV specific markers CD63, CD81 and CD9 tetraspanins. Western blot also showed EML4‐ALK variant 1 (120 kDa) and variant 3 (87 kDa) in H3122 and H2228 purified EVs, respectively. EV preparations contained an RNA profile composed of small RNA (< 200 nt), although 18S and 28S rRNA peaks were also present. dPCR and NGS confirmed EML4‐ALK rearrangements in EVs derived from both cell lines.

**Summary/Conclusion**: EML4‐ALK fusions can be detected at the RNA levels and at the protein levels analyzing EVs derived from H3122 and H2228 NSCLC cell lines. These results set the stage for the development of EV‐based non‐invasive ALK testing in NSCLC patients.

### PS15.09 Cancer Genotyping in Extracellular Vesicle‐Derived DNA by Droplet Digital PCR: Evaluation of Sample Preparation Workflows

Rebekka Van Hoof, KU Leuven, UHasselt, VITO

Karen Hollanders, Flemish Institute for Technological Research (VITO), Health Unit, Boeretang 200, 2400 Mol, Belgium

Sarah Deville, Flemish Institute for Technological Research (VITO), Health Unit, Boeretang 200, 2400 Mol, Belgium

Patrick Wagner, KU Leuven, Laboratory for Soft Matter and Biophysics, Celestijnenlaan 200D, 3000 Leuven, Belgium

Jef Hooyberghs, Flemish Institute for Technological Research (VITO), Health Unit, Boeretang 200, 2400 Mol, Belgium

Inge Nelissen, VITO nv

**Introduction**: The sensitivity of lung cancer mutation detection in minimally invasive liquid biopsies, where only a blood samples is taken, can potentially be improved by enriching and analyzing extracellular vesicles (EVs). Whereas the effect of different EV separation methods on their protein and nucleic acid content is well described, a comparison of the downstream genotyping performance on the EV‐derived RNA/DNA is currently lacking.

**Methods**: We applied droplet digital PCR in comparison to real‐time PCR to assess genotyping performance for three workflows, consisting of distinct EV separation methods and a common method for extraction of enclosed RNA/DNA. Ultracentrifugation was used in parallel to two column‐based methods, size exclusion chromatography (SEC) and exoEasy membrane affinity chromatography, that are possibly suited for clinical translation. EVs were separated from conditioned media of the human non‐small cell lung carcinoma cell line NCI‐H1975, containing the epidermal growth factor receptor gene mutation c.2369C>T, p.T790M. The EV fractions were analyzed for their purity and identity, RNA/DNA fragment length profile, and type and concentration of nucleic acid template sequences bearing the mutation.

**Results**: The purest fractions of small EVs (< 200 nm) were obtained by SEC. We found that RNase/DNase treatment of isolated EV fractions was necessary to remove ribosomal RNA and cell‐free DNA, and recover pure EV‐RNA/DNA fractions from cell cultures. Despite differences in nucleic acid yields and fragment length profiles following the three workflows, we detected the mutation with similar sensitivity (down to 300 copies/μL) and observed that mainly EV‐derived genomic DNA served as a template

**Summary/Conclusion**: We suggest that fast and easy‐to‐use column‐based EV separation combined with genomic DNA typing may be a suitable approach for translation to clinical applications. This may lead to a broader application of liquid biopsies for screening processes and routine follow‐up of patients.

### PS15.10 Detection of exosomal long non‐coding RNAs in low‐volume blood serum samples from colorectal cancer patients

Marie Madrzyk, CEITEC Masaryk University

Tina Catela Ivković, CEITEC Masaryk University

Sára Vilmanová, CEITEC Masaryk University

Jan Kotouček, Department of Pharmacology and Toxicology, Veterinary Research Institute

Josef Mašek, Department of Pharmacology and Toxicology, Veterinary Research Institute

Ondřej Slabý, CEITEC Masaryk University

**Introduction**: Colorectal carcinoma (CRC) is the third most common cause of cancer related deaths in the world population. As the early detection decreases mortality rates of the disease, the development of a sensitive and non‐invasive detection method for early stages of CRC is of high priority. This gap could be filled by long non‐coding RNA (lncRNA) which have been shown to have high potential as diagnostic biomarkers. In blood, lncRNAs can be found encapsulated within exosomes that protect them from degradation and thus serve as a valuable source of intact RNA. We aimed at developing a protocol that would enable the detection of lncRNA in low‐input samples which is often the main limitation for this approach.

**Methods**: Exosomes were isolated from human blood serum from CRC patients and healthy volunteers. For protocol standardization we compared different exosome isolation techniques and two commercially available RNA isolation kits. The results were evaluated according to the ISEV recommendations. Exosomes were validated by dynamic light scattering, electron microscopy and by exosome‐specific content analysis. Protein markers were detected by western blot and RNA markers by qRT‐PCR. Long ncRNAs were analysed by qRT‐PCR with an additional preamplification step.

**Results**: We optimized and standardized the protocol for exosome purification and subsequent RNA isolation starting from very low amount of human blood sera. We successfully detected lncRNA GAS5, which is known to be associated with CRC progression in samples isolated from only 150 μL of CRC patient blood sera. In accordance with the current literature, lncRNA FRG2DP was not detectable. The obtained expression results were confirmed in CRC tissue samples.

**Summary/Conclusion**: Although lncRNA show to be a class of promising biomarkers, the detection of exosomal lncRNA in low‐input samples using available methods proves challenging. Our results show that exosomal lncRNA could potentially be used for diagnostic purposes even with limited amounts of patient sample; however, careful optimization and standardization of the protocol is a prerequisite for further research.

### PS15.11 Extracellular vesicles as one of the sources of transrenal mycobacterial DNA for urine‐based liquid biopsy of Tuberculous Meningitis

Manisha Dass, PGIMER (Post Graduate Institute of Medical Education and Research)

Simran Aittan, PGIMER (Post Graduate Institute of Medical Education and Research)

Rajagopalan Muthumohan, Translational Health Science and Technology Institute

Divya Anthwal, PGIMER (Post Graduate Institute of Medical Education and Research)

Rakesh Kumar Gupta, Post Graduate Institute of Medical Education and Research

Pooja Kumari, All India Institute of Medical Sciences

Neera Sharma, Dr. Ram Manohar Lohia Hospital

Rajesh S Taneja, Dr. Ram Manohar Lohia Hospital

Lokesh Kumar Sharma, Dr. Ram Manohar Lohia Hospital

Jaya Sivaswami Tyagi,All India Institute of Medical Sciences

Sagarika Haldar, PGIMER (Post Graduate Institute of Medical Education and Research)

**Introduction**: Tuberculous Meningitis (TBM), a devastating form of extrapulmonary tuberculosis (EPTB), has a poor diagnostic and prognostic clinical scenario. We evaluated the utility of extracellular vesicles (EVs) as a source of transrenal DNA (cell‐free DNA beyond the glomerular filtration of our body) for urine‐based liquid biopsy diagnosing TBM.

**Methods**: We isolated EVs from urine and characterized them using immunoblotting (CD63, CD9, Alix and Lamp2) and scanning electron microscopy (vesicle sizes: 50 nm to 900 nm). We further isolated DNA from EVs (EV‐DNA) and extracellular vesicles‐free fraction (EV‐F DNA). Targeting a highly repetitive 36‐bp fragment specific to Mycobacterium tuberculosis complex, we developed transrenal TBM DNA (Tr‐TBM DNA) assay and applied the same in EV‐DNA and EV‐F DNA of urine from suspected ‘TBM’ group (n = 44) [categorized as ‘Definite’ TBM (n = 8), ‘Probable’ TBM (n = 15), ‘Possible’ TBM (n = 21)] and ‘Non‐TBM’ group (n = 10). We also used GAPDH primers to estimate DNA concentration in urinary EVs of TBM patients (n = 10) and healthy individuals (n = 10).

**Results**: ROC‐curves were generated using qPCR results of ‘Definite’ TBM and ‘Non‐TBM’ category in both EV‐DNA and EV‐F DNA samples and cut‐off values were selected to provide 100% specificity. In ‘Definite’ TBM category, Tr‐TBM DNA assay had a sensitivity of 100% for EV‐DNA and 87.5% for EV‐F DNA. When EV DNA+EV‐F DNA results were combined, the sensitivity of Tr‐TBM DNA assay was 81.8% in combined ‘TBM’ group whereas Xpert MTB/RIF assay showed a marginal sensitivity of 18.2%. DNA concentration in urinary EVs of TBM patients was statistically higher than in healthy individuals; indicating either more release of EVs or more DNA packaging in EVs of patients.

**Summary/Conclusion**: Our assay's sensitivity of ∼82% corresponded to the WHO's Target Product Profile prescribed for EPTB. Our study showed that EVs travel from brain to the urine carrying biomarkers, indicating their importance in TBM pathogenesis.

### PS15.12 mRNA expression panel analysis of brain‐derived extracellular vesicles reveals increased immunological defense‐related mRNA in a mouse model of stroke

Santra Brenna, UKE

Annika Bub, UKE

Paul Kügler, UKE

Yuqi Gui, UKE

Tim Magnus, UKE

Berta Puig, UKE

**Introduction**: Extracellular vesicles (EVs) are lipid bilayer enclosed vesicles secreted by all types of cells investigated so far and are important means of cell‐to‐cell communication. Apart from containing and delivering proteins and lipids, they also carry exRNA which can elicit a response on the recipient cells. In previous experiments, we demonstrated an increase of astrocytic brain‐derived EVs (BDEVs) in the acute phase (24h hours) after stroke‐reperfusion in a mice model of transient Middle Cerebral Artery Occlusion (tMCAO). The aim of the present study was to analyze the mRNA present in BDEVs in mice subjected to tMCAO for 72h (when probably some recovery mechanisms are already set in motion) and compare them to sham‐operated mice.

**Methods**: We applied a targeted approach by using an mRNA expression panel containing a multiplexed expression of 770 genes. Moreover, we also took advantage of the capacity of these panels to selectively quantify mRNAs without the need for their isolation, and we compared the results obtained with and without mRNA extraction from EVs.

**Results**: Our results indicate, on the one hand, that 72 hours after tMCAO and reperfusion, EVs from brain contain increased amounts of several mRNAs related to defense response and immune system process (GO terms: 0006952 and 0002376). Importantly, we also selected mRNAs with higher expression, and we found them to be full‐length in BDEVs, and therefore, translatable to proteins.

**Summary/Conclusion**: On the other hand, we obtained similar results when comparing the same panel processed with or without RNA extraction from EVs, indicating that these types of panels can help the study of RNAs in EVs by minimizing the purification steps and thus, the variability among the studies.

## PS16 miRNA Biomarkers

Chair: D. Michiel Pegtel, Exosome Research Group, Dept. Pathology, Cancer Center Amsterdam, VU University Medical Center, de Boelelaan 1118, 1081 HV Amsterdam, The Netherlands

Chair: Leonora Balaj, Massachusetts General Hospital and Harvard Medical School, United States

### PS16.01 A novel electrochemical biosensor for exosomal microRNA‐181 detection based on a catalytic hairpin assembly circuit

Shihua Luo, Department of Laboratory Medicine, Nanfang Hospital, Southern Medical University, Guangzhou 510515, PR China

Ruyi Zhang, Department of Laboratory Medicine, Nanfang Hospital, Southern Medical University, Guangzhou 510515, PR China

**Introduction**: Exosomal microRNAs (miRNAs) derived from different cells are proposed to be important noninvasive biomarkers for the diagnosis of cardiovascular disease. Recently, sensitive and reliable sensing of exosomal miRNAs has been garnered significant attention.

**Methods**: Herein, a novel electrochemical biosensor based on a step polymerization catalytic hairpin assembly (SP‐CHA) circuit is designed for exosomal miR‐181 detection. Exosomal miR‐181 as a trigger, induced SP‐CHA process and generated a large number of T shaped concatemers with different length on the electrode surface.

**Results**: These ultra‐concatemers could provide a much enhanced signal‐to‐noise ratio with the linear range from 10 fM to 100 nM and the detection limit of 7.94 fM. Furthermore, this assay was successfully applied to the detection of exosomal miR‐181 in serum samples of normal healthy controls and patients with coronary heart disease (CHD) and the results were consistent with those analysis collected from qRT‐PCR. The assembly demonstrated great performance in differentiating CHD patients from healthy controls (AUC:0.9867).

**Summary/Conclusion**: Collectively, this sensing system possessed high stability and sensitivity with ease of operation and cost efficiency, leading to great potential for exosomal miRNAs detection in cardiovascular disease.

### PS16.02 Expression Profiling of Exosomal MicroRNA in Asymptomatic HBsAg Carriers and Chronic Hepatitis B Patients

Daming Wang, Clinical Laboratory Medicine Center

Tingting Huang, Clinical Laboratory Medicine Center,Shenzhen Hospital,Southern Medical University

Tingyu Ren, Clinical Laboratory Medicine Center,Shenzhen Hospital,Southern Medical University

Zheng Lei, Department of Laboratory Medicine, Nanfang Hospital, Southern Medical University

**Introduction**: The differential expression levels of miRNAs in the peripheral plasma and the plasma exosome of chronic hepatitis B patients, hepatitis B carriers and healthy subjects were compared and analyzed, to evaluate the potential value of exosome miRNAs in HBV infection.

**Methods**: There were 55 patients in experimental group 1 (chronic hepatitis B patients), 49 patients in experimental group 2 (hepatitis B carriers) and 46 subjects in control group (healthy adults). One part, total RNA was extracted and hybridized by gene chip for image capture and data analysis from the plasma. Candidate genes related to hepatitis B were screened by the fluorogenic quantitative PCR validation according to the differential expression of miRNAs. Another part, total RNA in the plasma exosome was extracted and the expression of candidate genes in plasma was detected by fluorogenic quantitative PCR.

**Results**: The up‐regulated candidate genes are miRNA‐7a‐5p, miRNA‐16‐5p, miRNA‐27a‐3p,miRNA‐29a‐3p, miRNA‐142‐3p and miRNA‐221‐3p, and the down‐regulated candidate genes are miRNA‐5787,and miRNA‐8069 in peripheral plasma.The down‐regulated candidate genes are miRNA‐1246, miRNA‐150‐5p, miRNA‐5787 and miRNA‐8069 in peripheral plasma exosomes.

**Summary/Conclusion**: These 8 miRNAs are initially identified as candidate miRNA biomarkers in peripheral plasma after hepatitis B virus infection. These 4 miRNAs are initially identified as candidate miRNA biomarkers in peripheral plasma exosomes after hepatitis B virus infection.

### PS16.03 Nondestructive profiling of exosomal miRNA by sensitive nanoprobe for breast cancer diagnostics

Ye Zhang, Southern Medical University

Yuan Wu, Southern Medical University

Zheng Lei, Department of Laboratory Medicine, Nanfang Hospital, Southern Medical University

**Introduction**: To date, direct visualization of exo‐miRNA in situ has remained extremely challenging due to i) their short length (≈22 nt), ii) their sequence homology, and particularly iii) the difficulties associated with transporting intact molecular probes into sEVs. Conventional methods for circulating miRNA quantification such as reverse transcription quantitative polymerase chain reaction (RT¬qPCR), northern blotting, and the use of microarrays fail at nondestructive detection of exo‐miRNA. All the above mentioned methodologies are mostly restricted to an inevitable extraction step prior to nucleic acid detection procedures, making the analytical results less reliable in reflecting the original exosome gene. Such method has greatly hindered the tracing of exo‐miRNA and hampered attempts at unveiling the mechanisms of exosome ¬mediated intercellular communication.

**Methods**: Herein, a nanowire‐guided catalytic hairpin assembly (nanowire‐CHA) nanoprobe was reported for direct determination of exo‐miRNA with high sensitivity. Combining the advantages of nondestructive penetration via nanowire, high sensitivity and specificity of CHA, and wash‐free florescence readout, the developed strategy enables sensitive detection of exo‐miRNA without RNA extraction.

**Results**: Under the best combination of exo‐miRNAs, the proposed platform can distinguish breast cancer patients with more than 99% accuracy in 121 plasma samples with the help of machine learning algorithms.This exo‐miRNA platform with a capacity to learn could have potential for clinical early diagnostics and advance the clinical application of exo‐miRNA.

**Summary/Conclusion**: For the first time, we describe a robust and reliable nanowire‐CHA nanoprobe for direct visualization of exo‐miRNA in situ without the need for damaging the exosome membrane or extracting exosome cargoes. By employing a DNA nanowire to facilitate exosome penetration, CHA system can be conveniently delivered into the exosome to enable real‐time detection of various exosome cargoes such as nucleic acids and proteins. Meanwhile, the three florophores carried by the DNA nanowire nanostructure further increase the analytical sensitivity and the potential for multiplex detection. This technology opens an avenue for tracing exosome cargoes in situ, providing a novel strategy to investigate exosome‐derived fundamental biological mechanisms and explore subsequent clinical applications.

### PS16.05 Subpopulations of exosomes purified via different exosomal markers carry different microRNA contents

Ching‐Hua Hsieh, Kaohsiung Chang Gung Memorial Hospital

Yi‐Chan Wu, Kaohsiung Chang Gung Memorial Hospital

**Introduction**: The present study was designed to investigate the miRNA profiles of circulating exosomes from septic mice by affinity techniques and the exosomes subpopulations captured by affinity purification using antibodies against Rab5b, CD9, CD31, and CD44. These findings revealed the existence of exosome subpopulations with unique miRNA contents.

**Methods**: In this study, exosomes from the serum of C57BL/6 mice after cecum ligation and perforation (CLP) or sham operation were isolated by precipitation using ExoQuick‐TC and affinity purified with anti‐Rab5b, anti‐CD9, anti‐CD31, and anti‐CD44 antibodies using the Exo‐Flow Exosome Capture kit to collect exosome subpopulations. RNA extracted from the exosomes isolated by ExoQuick‐TC were profiled by next‐generation sequencing (NGS). Real‐time quantitative reverse transcription polymerase chain reaction (RT‐qPCR) was also employed to determine the expression profiles of four representative exosomal miRNAs (mmu‐miR‐486‐5p, mmu‐miR‐10a‐5p, mmu‐miR‐143‐3p, and mmu‐miR‐25‐3p) selected from the NGS analysis.

**Results**: The results revealed that the expression patterns of these miRNAs in exosomes isolated by ExoQuick‐TC as determined by RT‐qPCR and NGS were similar, showing upregulation of mmu‐miR‐10a‐5p and mmu‐miR‐143‐3p but downregulation of mmu‐miR‐25‐3p and mmu‐miR‐486‐5p following CLP when compared to the levels in exosomes from sham control mice. However, their expression levels in the antibody‐captured exosome subpopulations varied. The miRNAs in the exosomes captured by anti‐Rab5b or anti‐CD9 antibodies were more similar to those isolated by ExoQuick‐TC than to those captured by anti‐CD44 antibodies. However, there were no significant differences in these four miRNAs in CD31‐captured exosomes.

**Summary/Conclusion**: This study demonstrated that purification with different exosomal markers allows the collection of different exosome subpopulations with various miRNA contents. The results of this study demonstrate the heterogeneity of circulating exosomes and suggest the importance of stratifying exosome subpopulations when using circulating exosomes as biomarkers or investigating exosome function. In addition, this study also emphasized the necessity of using a consistent exosome marker across different samples as detecting biomarkers.

### PS16.06 Ovarian cancer exosomal proteins and miRNAs are associated with disease progression

Shayna Sharma, University of Queensland Centre for Clinical Research

Xiaoqi Qian, University of Queensland Centre for Clinical Research

Yohaann Jafrani, University of Queensland Centre for Clinical Research

Dominic Guanzon, PhD, Exosome Biology Laboratory, Centre for Clinical Diagnostics, UQ Centre for Clinical Research, The University of Queensland, Australia

Andrew Lai, PhD, Exosome Biology Laboratory, Centre for Clinical Diagnostics, UQ Centre for Clinical Research, The University of Queensland, Australia

Nihar Godbole, University of Queensland Centre for Clinical Research

Priyakshi Kalita‐de Croft, University of Queensland Centre for Clinical Research

John Hooper, PhD, Mater Research Institute

Terry K. Morgan, MD, PHD, Oregon Health and Science University

Carlos Salomon, MSc, DMedSc, PhD,The University of Queensland

**Introduction**: Ovarian cancer has the highest mortality rate amongst gynaecological tumours, with the 5‐year survival rate at an advanced stage being approximately 20%. Therefore, there is an urgent need to identify the mechanisms underlying disease progression. Small extracellular vesicles, specifically, exosomes, have gained significant attention due to their ability to transport vital information, in the form of proteins and miRNAs

**Methods**: Exosomes were isolated from plasma (benign or epithelial ovarian cancer (Stages I‐IV, n = 127)) using differential centrifugation and size exclusion chromatography. Using a quantitative, information‐independent acquisition (Sequential Windowed Acquisition of All Theoretical Mass Spectra [SWATH]) approach, differentially abundant circulating exosomal proteins were identified across ovarian cancer progression. miRNA sequencing was used to obtain the miRNA profile. To identify the origin of exosomes present in plasma, SKOV‐3 and OVCAR‐3 cell lines were used to determine the expression of specific exosomal proteins and miRNAs, in both the cell lines and their exosomes, using Multiple Reaction Monitoring (MRM) Mass Spectrometry, and qPCR.

**Results**: SWATH analysis to quantify exosomal protein expression identified 356 proteins that were differentially expressed. A cladogram was generated in which proteins with similar trends clustered together, with 25 proteins changing significantly (p < 0.05) across stage. Noteworthy proteins included: TRFE (Transferrin); and A1AG1 (alpha‐1‐acid glycoprotein‐1), both of which have a similar trend of expression. SKOV‐3 cells showed greater expression of miRNAs associated with advanced stage disease compared to the less aggressive OVCAR‐3. Furthermore, the qRT‐PCR also revealed that there was higher expression of these miRNAs in exosomes secreted by SKOV‐3 cells compared to OVCAR‐3 exosomes.

**Summary/Conclusion**: We propose that exosomes carry a specific set of proteins and miRNAs, which are closely associated with ovarian cancer progression.

### PS16.07 Development of a biomarker for peripheral artery disease using extracellular vesicles associated miRNA

Naoya Kuriyama, Division of Molecular and Cellular Medicine, Institute of Medical Science Tokyo Medical University

Yusuke Yoshioka, Department of Molecular and Cellular Medicine, Tokyo Medical University

Shinsuke Kikuchi, Department of Vascular Surgery, Asahikawa Medical University, Asahikawa, Japan

Nobuyoshi Azuma, Department of Vascular Surgery, Asahikawa Medical University, Asahikawa, Japan

Takahiro Ochiya, PhD, Department of Molecular and Cellular Medicine, Tokyo Medical University

**Introduction**: As a result of an increase of metabolic disorders, peripheral artery disease (PAD) has been increasing. Chronic limb‐threatening ischaemia (CLTI) is an advanced stage of PAD and associated with poor mortality and limb loss. To prevent limb loss, we need a non‐invasive biomarker for early detection of CLTI. Extracellular vesicles (EVs) can be a potential biomarker for CLTI and have important roles in atherosclerosis. This study was to focus on EV associated miRNAs as a biomarker for CLTI.

**Methods**: Sampling was approved by the local ethics committee. Ten patients with CLTI and 10 patients without CLTI were analyzed. All patients were diagnosed as diabetes mellitus and 50% were dependent on hemodialysis (HD) due to end‐stage renal failure. EVs were isolated from patients’ serum before surgery using ultracentrifugation and total RNA including miRNA was isolated from EVs. The expression profile of miRNAs associated with CLTI was analyzed by next generation sequencing (NGS). Results in non‐HD group (44 patients with CLTI and 18 patients without CLTI) were validated by qPCR and ddPCR. In addition, we also analyzed EV associated miRNAs isolated from the cultured medium of human atherosclerotic plaques obtained after femoral endarterectomy.

**Results**: Although 43 miRNAs associated with CLTI were identified, the expression pattern was difference between non‐HD and HD group. Twenty and 23 miRNAs were identified as non‐HD and HD patients with CLTI, respectively. There are no overlapping miRNAs between non‐HD and HD. However, these target miRNAs were not detected by qPCR. One of the possible reasons is that the expression levels of these miRNAs were very low. On the other hand, ddPCR revealed that the expression patterns of several miRNAs were same as NGS. A part of them consisted with miRNAs derived from atherosclerotic plaque.

**Summary/Conclusion**: We identified the expression profile of miRNA associated with CLTI by NGS. The efficacy of ddPCR as a validation method was indicated and EV miRNAs could be a potential biomarker for diagnosis of CLTI. EVs are important role in early detection of PAD and may involve atherosclerosis.

### PS16.08 Exosomal or non‐exosomal miRNA expression level – does it really make sense?

Alisa Petkevich, PhD student, RUDN University

Vadim Pospelov, RUDN University

**Introduction**: When it comes to diagnostic approaches and search for molecular markers one of the main points is the sensitivity of the chosen marker to the pathogenesis of the disease and resistance to external factors effects associated with the preanalytical stage of the research. This research is an attempt to answer the question which miRNA form " associated with exosomes or not associated with exosomes " is more stable under different condition of preanalytical step in the search for diagnostic miRNA marker of nonalcoholic fatty liver disease (NAFLD).

**Methods**: Blood samples were collected from 12 healthy volunteers and 14 patients with NAFLD, blood was collected in 2 ml EDTA tubes (5 tubes 8 hour fasting and 5 tubes 2 hours after meal). The storage conditions were as follows: immediate centrifugation after blood collection and freezing ‐80°C (3000 g 10 minutes), storage of the collected blood during 24 hours at room temperature, storage of the plasma during 24 hours at room temperature and storage of the collected blood during 24 hours at +4°C. All the samples were stored at ‐80°C. Exosomes were isolated with sucrose density following precipitation method with miRCURY Exosome Serum/Plasma Kit (Qiagen, Germany). MiRNAs from exosomes and supernatant were isolated with miRNeasy Serum/Plasma Advanced kit (Qiagen, Germany), qPCR was performed with LNA primers for following miRNAs: miRNA‐21, miRNA‐34a, miRNA‐122.

**Results**: The AUC value for exosomal miRNAs was >0,7 in every described case condition whilst the AUC value for free miRNAs was in some cases lower than 0,7. The highest meaning 0,85 (sensitivity " 0,8; specificity " 0,77) was for the exosomal miRNAs isolated from blood obtained 2 hours after meal and immediately centrifugated to collect plasma.

**Summary/Conclusion**: In this research it seems exosomal miRNAs allow to obtain more reproducible results then free miRNAs. Nevertheless, it is worth to note that exosomal miRNA expression level identification require more sophisticated technique comparatively to free miRNA what maybe crucial for implementation into clinical routine practice. However, in strict observance of the certain conditions free miRNAs may allow to get reliable results.

### PS16.09 MicroRNA profiling of plasma‐derived extracellular vesicles distinguishes symptomatic and asymptomatic carotid artery stenosis

SNEHA RAJU, MD, TGHRI

Dakota D. Gustafson, Toronto General Hospital Research Institute

Kamalben Prajapati, Toronto General Hospital Research Institute, University Health Network, Toronto, Canada

Natalie J. Galant, Princess Margaret Cancer Center, Toronto, Canada

Steven R. Botts, Toronto General Hospital Research Institute, University Health Network, Toronto, Canada

Giuseppe Papia, Sunnybrook Health Sciences Center, Toronto, Canada

Jason E. Fish, PhD, Toronto General Hospital Research Institute

Kathryn L. Howe, MD, PhD, Toronto General Hospital Research Institute, University Health Network, Toronto, Canada

**Introduction**: Carotid artery atherosclerosis is a major cause of ischemic stroke. Managing patients with asymptomatic disease remains challenging given the lack of reliable tests to identify those prone to plaque progression and stroke. Given the functional and diagnostic roles of extracellular vesicle (EV) contents, we hypothesized that plasma EV‐derived microRNA (miRNA) differs between symptomatic and asymptomatic patients.

**Methods**: EVs were isolated via serial centrifugation and enriched using size exclusion chromatography (qEVoriginal columns, 70 nm; Izon Science Ltd). EVs were confirmed with MISEV 2018 guidelines: western blot analysis of common EV markers (CD63, CD81, Alix), nanoparticle tracking analysis (NTA), and cryogenic transmission electron microscopy. Differential EV‐miRNA expression was determined with next‐generation sequencing (HTG Molecular Diagnostics Inc.) and analysis using Partek Genomics Suite (v8.0).

**Results**: Twelve patient plasma samples were collected (n = 6 symptomatic, n = 6 asymptomatic). CD63 expression confirmed EV enrichment in fractions 7–10, with minimal lipoprotein contamination. EV isolation was further confirmed by CD81 and Alix expression (n = 3 samples/group). NTA revealed no significant differences in EV concentration or size between groups (n = 3, p>0.05). Principal component and heatmap analysis of miRNA sequence data revealed clustering of symptomatic carotid plasma samples, while asymptomatic samples were either starkly different (n = 5) or approximated symptomatic profiles (n = 1), suggesting a disease gradient. When symptomatic and asymptomatic carotid EV‐miRNA profiles were compared, 190 miRNAs were differentially expressed, with miRNA‐654‐5p and miRNA‐127‐3p showing the greatest up‐ and downregulation, respectively (p < 0.05, fold‐change ‐2< or >2, excluding low expression miRNAs).

**Summary/Conclusion**: Plasma EV‐miRNA profiles may differentiate symptomatic vs. asymptomatic carotid stenosis and together with clinical characteristics, may be used to risk stratify asymptomatic patients.

### PS16.10 Urinary extracellular vesicles as a source of biomarkers in the prevention of kidney subclinical rejection

Andrea Carraro, University Hospital of Padua, U.O.C Pediatric Nephrology, Dialysis and Transplant Unit, Department of Women's and Children's Health, Italy

Diana Marzenta, University Hospital of Padua, U.O.C Pediatric Nephrology, Dialysis and Transplant Unit, Department of Women's and Children's Health, Italy

Emanuele Vianello, University Hospital of Padua, U.O.C Pediatric Nephrology, Dialysis and Transplant Unit, Department of Women's and Children's Health, Italy

Benedetta Bussolati, University of Turin, Department of Molecular Biotechnology and Health Sciences, Italy

Federica Collino, Major General Hospital of Milan, Ca Granda Foundation IRCCS, Laboratory of Translational Research in Pediatric Nephro‐urology, Italy

Loris Bertoldi, BMR‐Genomics, Padua, Italy

Giuseppe Benvenuto, BMR‐Genomics, Padua, Italy

Luisa Murer, University Hospital of Padua, U.O.C Pediatric Nephrology, Dialysis and Transplant Unit, Department of Women's and Children's Health, Italy

Elisa Benetti, University Hospital of Padua, U.O.C Pediatric Nephrology, Dialysis and Transplant Unit, Department of Women's and Children's Health, Italy

Susanna Negrisolo,University Hospital of Padua, U.O.C Pediatric Nephrology, Dialysis and Transplant Unit, Department of Women's and Children's Health, Italy

**Introduction**: Urinary extracellular vesicles (UEVs) are lipid membrane‐bound nanoparticles released from different cells of the nephro‐urological system. They may carry different types of proteins, lipids, and miRNAs, reflecting the physio pathological status of the cells they originated from. Particularly, UEVs and their miRNAs could be useful biomarkers for kidney allograft injury discovery. The aim of this study is to characterize UEVs and evaluate the expression of vesicles miRNAs in kidney transplanted children, to identify possible non‐invasive markers of rejection.

**Methods**: The UEVs were isolated from urine samples of 20 pediatric transplanted patients, with a stable condition or kidney rejection at one‐year post‐transplantation. The UEVs were isolated by the ultracentrifugation method. Their characterization was performed by electron microscopy and scattering analysis (Nanosight 3000). Vesicular miRNAs were extracted with a commercial kit and enriched before sequencing with the Illumina instrument.

**Results**: The UEVs concentration was between 2,79 × 1011 " 9,56 × 1011 with a size diameter of about 197 ± 7 nm. The miRNA concentration was between 197 " 907 pg/μl. The sequencing showed about 522 different miRNAs and 48 of them were differentially expressed between patients with subclinical rejection or stable condition.

**Summary/Conclusion**: These data support the presence of an identifiable vesicular miRNAs profile in UEVs, in association with subclinical rejection in pediatric kidney transplantation.

### PS16.11 Novel serum exosomal miRNAs blend into radiogenomics to predict Early‐stage Lung cancer at foremost biopsy

Nishant Patel, Zhongda Hospital, Medical School of Southeast University, Nanjing

Lili Ren, School of Pharmacy, Nanjing Tech University

Haijun Zhang, Zhongda Hospital, Medical School of Southeast University, Nanjing

**Introduction**: Detection of early‐stage lung cancer (ESLC) is the center of the attraction for improving lung cancer survival, which is drastically declined. Therefore, the initial preventing and treating lung cancer is desperately needed throughout the world. Our study has primarily aimed at serum‐based exosome‐derived miRNAs in the detection of ESLC and discrimination between Benign lung nodules (BLN) and Healthy (H) individuals. We aim to determine sensitive and specific liquid biopsy‐based biomarkers, which would blend in with clinic‐radiological features to develop the radiogenomics model for predicting ESLC from BLN in < 20mm lung nodules.

**Methods**: Exploration of the non‐invasive serum exosomal based miRNAs (sEXO‐miRNAs) biomarkers through NGS in 9 participants (ESLC‐3, BLN‐3 and H‐3) and validated by 143 (ESLC‐46, BLN‐46 and H‐51) recruiters via RT‐PCR in a cohort of Zhongda Hospital affiliated to Southeast University. Furthermore, the sEXO‐miRNAs were combined with clinic‐radiological data to establish the radiogenomics model via logistic regression analysis of 2 groups (ESLC‐46, BLN‐46). The study was approved by the ethics committee of Zhongda Hospital affiliated to Southeast University, Nanjing, China.

**Results**: We have determined >700 differently expressed miRNAs mutual between the 3 groups through NGS. Despite those miRNAs, we have elected the foremost highly up‐or down‐regulated significant sEXO‐miRNAs as biomarkers following certain threshold and statistical criteria, which were sEXO‐miR‐424‐5p, sEXO‐miR‐512‐5p, and sEXO‐miR‐1271‐5p for subsequent validation by RT‐PCR in 3 groups. Moreover, the radiological data (12 signs) were combined with 3 sEXO‐miRNAs in logistic regression analysis, which were representing the AUC of 0.9844 (0.966∼1.000,p < 0.0001), whereas the clinical data (4 signs) combination was showing the AUC of 0.8852 (0.8173∼0.9530, p < 0.0001). The multivariate logistic based radiogenomics model for predicting ESLC significantly achieved AUC of 0.9272 (0.8774∼0.9770), whereas sensitivity, specificity, PPV, NPV and accuracy were 86.36%, 83.33%, 82.61%, 86.96% and 84.78% respectively.

**Summary/Conclusion**: Our study discovered significant 3 DEs sEXO‐miRNAs (hsa‐miR‐424‐5p, hsa‐miR‐512‐5p, and hsa‐miR‐1271‐5p), which blend in with clinicoradiological data, lead to the development of radiogenomics model, which represented great potential in the detection of initial ESLC. The radiogenomics model can predict ESLC precisely and discriminate from BLN in < 20mm lung nodules along with specific significant predictor factors such as Diameter, Lobulation, Calcification, sEXO‐miR‐424‐5p and sEXO‐miR‐1271‐5p.

### PS16.12 E‐liquid mediated alteration of sEV derived microRNAs from human lung cells is distinctly different from nicotine exposure

Sowmya Chinta, University of Salford

Pika Miklavc, University of Salford

Arijit Mukhopadhyay, University of Salford

**Introduction**: E‐Cigarette or vaping product use‐associated lung injury (EVALI) is a pulmonary disease with damaging health effects. In this study, we explored miRNAs from small extracellular vesicles (sEVs) for e‐liquid associated biomarkers. We focused on the large miRNA cluster on C14q32 (C14MC), known to be dysregulated in lung‐associated diseases. We asked if e‐liquid exposure alters the release of these miRNAs via sEVs, compared to nicotine.

**Methods**: We treated lung epithelial cell lines A549 and BEAS‐2B with e‐liquid (18mg/ml nicotine content) and commercial nicotine (both 100μM) for 72hrs. After treatment, sEVs were isolated using SEC method, followed by RNA isolation. Isolated sEVs were characterized using nanoparticle tracking analysis. MiRNA expression profile for 10 miRNAs from C14MC were measured by qPCR using 2^“” Ct method.

**Results**: NTA revealed 10 times higher concentration of sEV from cancer‐derived cell line A549 than from normal lung cell line BEAS‐2B (18 × 108 vs 1.4 × 108 particles/mL, both untreated), while the sEV sizes were similar (mean diameter: 75.5 nm vs 81.5 nm). With e‐liquid treatment, 2 miRNAs, miR‐323a‐5p and mir‐758‐5p, showed opposite trends in the two cell lines. MiR‐323a‐5p was downregulated in A549 (0.14 fold) while upregulated in BEAS‐2B (5.12 fold); for miR‐758‐5p the trend was reversed (12 fold vs 0.1 fold in A549 and BEAS‐2B, respectively). In comparison, with nicotine treatment both miRNAs were downregulated in both cell lines (0.86 to 0.09 fold).

**Summary/Conclusion**: Our initial results indicate that e‐liquid treatment cause distinct alterations in expression of sEV mediated miRNA. The two highlighted candidates are known as tumor‐suppressor miRNAs in multiple human cancers and can potentially serve as non‐invasive biomarkers for EVALI.

### PS16.13 Ischemic stroke risk assessment based on the detection levels of miRNA‐638 derived from exosomes in serum by a new developed lateral flow assay

Ana Rubio‐Monterde, Paperdrop Dx

Lourdes Rivas, Paperdrop Dx

Marc Gallegos, Paperdrop Dx

Josep M Aran, IDIBELL

Arben Merkoçi, Paperdrop Dx

Daniel Quesada‐González, Paperdrop Dx SL

**Introduction**: It has been reported that the levels of miR‐638 biomarker, a miRNA carried by exosomes in human serum, can be associated to atherosclerotic plaque vulnerability and the risk of suffering an ischemic stroke. However, there is currently a lack of rapid, efficient and affordable methods to detect miR‐638.

**Methods**: Herein, we have developed a new lateral flow assay, a paper‐based rapid diagnostic test, for the detection of miR‐638. Our lateral flow assay employs DNA probes for the recognition of the biomarker and gold nanoparticles as colorimetric labels, so that the color intensity of the test line is an indicative of the level of miR‐638. Therefore, our test turns out to be portable, equipment and battery‐free, fast (around 15 min), affordable and highly user‐friendly since, in a single step (just by adding approx. 100 μL of serum on the paper strip), any user would be able to carry on the assay without any type of previous expertise or training.

**Results**: At this moment, our assay is able to detect a concentration of miR‐638 as low as 10 ng/mL without previous amplification of the target. Within a miR‐638 concentration range between 10 and 1000 ng/mL, the different miRNA levels (very low, low, medium and high) can be semi quantitatively related to the color intensity observed on the lateral flow strip just by naked eye. The miR‐638 levels found in a given sample can be correlated with a calibration curve carried out from scanned lateral flow strips at different concentrations.

**Summary/Conclusion**: We present for the very first time a low‐cost and rapid lateral flow assay able to assess ischemic stroke risk through a non‐invasive biomarker. The principle of this assay is the colorimetric detection of different levels of exosomal miR‐638 in serum, with a preliminary limit of detection of 10 ng/mL.

## PS17 EV and EV Cargo Characterization

Chair: Carla Oliveira, BIOINF2BIO, Porto, Portugal; i3S, Universidade do Porto, Portugal

Chair: Esther Nolte ‐ ‘t Hoen, Utrecht University, Netherlands

### PS17.01 A survey of current trends in urinary extracellular vesicle research

Uta Erdbruegger, University of Virginia Health System

Charles J. Blijdorp, MD, Erasmus MC, University Medical Center Rotterdam

Alicia Llorente, Oslo University Hospital

Elena S. Martens‐Uzunova, PhD, Department of Urology, Erasmus MC, University Medical Center Rotterdam

Dylan Burger, PhD, ISHF, Ottawa Hospital Research Institute, University of Ottawa

**Introduction**: The Urine Task Force of the International Society of Extracellular Vesicles (ISEV) was created to advocate for best practices in this emerging area of research. Here we present the results of a survey, conducted using SurveyMonkey by the Urine Task Force, to better understand current research practices in the study of urinary extracellular vesicles (EVs).

**Methods**: Two separate, identical surveys were administered: one directed to Urine Task Force members (28 respondents at the time of reporting) and one directed to the ISEV community (33 respondents).

**Results**: The mean time studying urinary EVs was 7.8 years for task force members and 5.4 years for community respondents. For task force members: 48.3% of respondents primarily focus on kidney, 44.8% prostate and 6.9% bladder. For the community focus was 29.4% kidney, 21.5% prostate, 19.6% bladder and 29.4% “other”. Both communities largely collect spot urines compared with timed collection (Task force: 78.6% spot vs 21.4% timed, Community: 75.8% spot vs 24.2% timed). Urine storage was a significant focus of the survey. For the Task Force: 92.9% of respondents studied samples stored >3 months, 57.1% samples stored < 3 months, and 50% studied fresh samples. For the community respondents this was 65.6% >3 months, 45.8% < 3 months, and 18.8% fresh. Both groups predominantly stored samples as “cell‐free urine”: 85.7% for task force and 65.6% for community. All task force respondents study samples frozen at ‐80°C with 10.7% of respondents also studying samples stored at 4°C. By contrast, 93.8% ‐of community respondents stored samples at 80°C, 9.4% at ‐20°C and 6.3% at 4°C. The task force ranked the following isolation methods in order of priority 1) centrifugation, 2) size exclusion chromatography, and 3) filtration. For the community survey this was 1) centrifugation, 2) size exclusion chromatography, and 3) precipitation. Both surveys prioritized the same downstream applications: 1) protein analysis, 2) RNA analysis, 3) functional assays. When asked to identify the key knowledge gap in the study of urinary EVs the urine task force identified “understanding of approaches to normalization” while the community identified “Impact of renal disease and comorbidities on EV analysis”.

**Summary/Conclusion**: In summary, the present survey identified key similarities and differences between current practices for the Urine Task Force and the urinary EV research community. Such information will be used to help guide future efforts to address key knowledge gaps.

### PS17.02 An interactive database and website to interrogate a novel proteomic profiling analysis of Jurkat cells extracellular vesicles and the dynamics of their composition upon HIV‐1 infection

Lorena Martin‐Jaular, Institut Curie / INSERM U932

Nathalie Névo, Institut Curie / INSERM U932

Julia Schessner, Max Planck Institute of Biochemistry

Mercedes Tkach, Institut Curie / INSERM U932

Mabel Jouve, Institut Curie / CNRS UMR 3215

Florent Dingli, Curie Institute, PSL Research University, Laboratoire de Spectrométrie de masse Protéomique, Paris, France

Damarys Loew, Université de Paris, INSERM U970, Paris Cardiovascular Research Centre, Paris, France

Kenneth W. Witwer,Johns Hopkins University School of Medicine

Matias Ostrowski, Instituto INBIRS, Universidad de Buenos Aires‐CONICET

Georg BornerMax Planck Institute of Biochemistry

Clotilde Thery, MD PhD, Institut Curie / INSERM U932

**Introduction**: Specific markers to distinguish different extracellular vesicles (EVs) of similar sizes or densities, such as exosomes, small ectosomes, and enveloped viruses like HIV, are still lacking. We present here a novel approach to tackle this question: unbiased proteomic profiling and network analysis for characterizing EV subtype composition without the need for exhaustive EV subtype separation. This method was applied to human Jurkat T cells, in either control conditions or upon infection by HIV‐1. It unravels the dynamics of EV subtype protein composition between these two conditions (Martin‐Jaular et al, 2021, EMBO J, e105492).

**Methods**: We partially separated EV subtypes from SILAC‐labeled Jurkat cells conditioned medium by centrifugal separation (10K, 30K, 100K). We performed quantitative proteomic analysis, and calculated a distribution profile between the 3 fractions for each identified protein. An interactive database calculates and displays profile neighbours of each EV protein, and plots respective protein networks. The same analysis comparing EVs under control versus HIV‐1 infection conditions identifies modifications of EV subtype‐specific protein composition. EV Protein partners inferred by the profiling tools were validated by immuno‐isolation, Western blot and MacsPlexExo analysis.

**Results**: We generated an interactive database to define groups of proteins with similar profiles, suggesting release in similar EVs, available at http://evprofiler.institut‐curie.org. Another interactive database comparing the protein profiles in control versus HIV‐1 infection will be also presented with this poster. We identified several preferred protein partners of subtypes of EVs bearing CD81 with or without CD63, or the T cell receptor. When comparing control and HIV‐induced EVs, we 1) observed differential behavior of the Nef viral protein compared to the other viral‐encoded proteins, suggesting its possible release in host‐EVs 2) identified several proteins that move towards the virus protein cluster and thus are released in the viral particle (MOV10, SPN), and 3) identified proteins that conversely move away from the virus protein cluster, and thus are released in non‐viral host‐derived EVs (SERINC3).

**Summary/Conclusion**: Our workflow provides a powerful, unbiased approach for identifying candidate markers and potential regulators of EV subtypes. It can be widely applied to in vitro experimental systems for investigating physiological or pathological modifications of EV release.

### PS17.04 Assessment of urinary extracellular vesicles isolation methods for proteomics application

Yilan Hu, Key Laboratory of Protein and Peptide Pharmaceuticals & Laboratory of Proteomics, Institute of Biophysics, Chinese Academy of Sciences, Beijing 100101, China

Xiulan Chen, Key Laboratory of Protein and Peptide Pharmaceuticals & Laboratory of Proteomics, Institute of Biophysics, Chinese Academy of Sciences, Beijing 100101, China

Fuquan Yang, Key Laboratory of Protein and Peptide Pharmaceuticals & Laboratory of Proteomics, Institute of Biophysics, Chinese Academy of Sciences, Beijing 100101, China

**Introduction**: Urinary extracellular vesicles (uEVs) are single‐layer membrane structures secreted by urothelial cells. Proteins packaged in uEVs are non‐degraded and non‐diluted, making uEVs attractive sources of potential biomarkers of urinary diseases. However, poor reproducibility of uEVs purification and low proteome coverage caused by uromodulin are still two significant difficulties in proteomic studies. There is a need for finding an efficient method which can meet the requirements of both good reproducibility and high proteome coverage.

**Methods**: We compared 4 methods for uEV isolation ‐ traditional ultracentrifugation (UC), ultracentrifugation with DTT treatment (DTT+UC), ultracentrifugation with filtration (F+UC), and ultrafiltration with filtration (F+UF). Then, different techniques, including electron microscopy, immunoblotting, nanoparticle tracking analysis, and LC‐MS/MS, were used for assessment of the 4 methods.

**Results**: 1.The F+UC method achieved the best performance in reproducibility, purity, and proteome coverage among the four methods.

2.The 0.22 μm filtration is an efficient step for removing uromodulin from uEVs, leading to in‐depth proteomic identification.

3.DTT treatment not only could not effectively remove uromodulin but also could cause side effects like morphological changes in uEVs and modification changes in surface proteins.

4.CD63, a classical maker protein of exosomes, was shown more enriched in urinary proteins than in uEVs.

**Summary/Conclusion**: In all, we have conducted a comprehensive assessment of uEVs isolation methods and F+UC method has shown a potential for application in clinical samples for finding biomarkers.

### PS17.05 NGS and Mass Spectrometry Profiles of Different Populations of Cancer‐Derived EVs Overlap in Specific Pathways

Tatyana Vagner, Cedars‐Sinai Medical Center

Elizabeth Hutchins, Translational Genomics Research Institute

Andrew Chin, Cedars Sinai Medical Center

Javier Mariscal, Cedars Sinai Medical Center

Minhyung Kim, Cedars Sinai Medical Center

Sungyong You, Cedars Sinai Medical Center

Agnes Kittel, Hungarian Academy of Sciences, Institute of Experimental Medicine

Krizia Sagini, Department of Surgery, Division of Cancer Biology and Therapeutics, Cedars‐Sinai Medical Center

Marco De Simone, Cedars‐Sinai Medical Center

Edit Buzás, Semmelweis University, Department of Genetics, Cell‐ and Immunobiology

Wei Yang, Cedars Sinai Medical Center

Clotilde Thery, MD PhDInstitut Curie / INSERM U932

Kendall Van Keuren‐Jensen, Translational Genomics Research Institute

Dolores Di Vizio,Cedars Sinai Medical Center

**Introduction**: Extracellular vesicles (EVs) are important mediators of intercellular communication that can be analyzed via liquid biopsy in patient biological fluids. EVs are typically isolated and analyzed in bulk; however, they are highly heterogeneous in size, cargo, biogenesis, intracellular origin, and function. Specific markers for different EV populations are missing, limiting our understanding of EV functional and molecular diversity. This study stems from the need to identify EV‐type‐specific as well as general EV markers in 3 EV fractions purified from 3 different cancer cell lines by differential ultracentrifugation followed by discontinuous iodixanol gradient

**Methods**: Differential centrifugation, discontinuous iodixanol density gradient, TRPS, TEM, LC‐MS/MS, RNA‐Seq

**Results**: We first refined a protocol to separate large oncosomes (EVs >1μm diameter released by highly metastatic cancer cells) from other large EVs of the ectosomal origin. Quantitative proteomic analysis of 3 EV populations (2,8K, 10K, and 100K), obtained from prostate cancer, glioma, and breast cancer cell lines showed that several proteins frequently attributed to exosomes are in fact present in all EVs and can be used as general EV markers. The most distinct protein expression patterns were identified in the fractions containing the largest and the smallest EVs (2,8K and 100K), while the intermediate fraction (10K) represented a transition between the two. Protein and mRNA expression profiles mirrored each other in all EV types, with the 2.8K fraction showing the largest overlap between the corresponding protein and mRNA species. PCA analysis showed well separated clusters containing the EV fractions and their corresponding cells of origin. Additionally, some of the top pathways, such as oxidative phosphorylation and mitochondrial dysfunction, were enriched in the fraction with largest EVs both at the protein and transcript level. We found that ∼80% of the RNA reads were coding RNAs in all EV fractions but the non‐coding RNAs seemed to be slightly enriched in 100K. Finally, we identified proteins that might be used as novel markers of EV populations

**Summary/Conclusion**: Comprehensive proteomic and transcriptomic analyses identify specific mRNA and protein profiles in 2,8K and 100K suggesting that cancer pathways are conserved at the protein and RNA level

### PS17.06 Proteomic Profiling of Plasma Exosomes From Chronic Myeloid Leukemia With Imatinib Resistance

Meiyong Li, The Second Affiliated Hospital of Nanchang University

xiao‐zhong wang, The Second Affiliated Hospital of Nanchang University

Cui Zhao, The Second Affiliated Hospital of Nanchang University

Qinghua Min, The Second Affiliated Hospital of Nanchang University

jin lin, The Second Affiliated Hospital of Nanchang University

Bo Huang, The Second Affiliated Hospital of Nanchang University

**Introduction**: Imatinib (IM), a tyrosine kinase inhibitors (TKIs), has markedly improved the survival and life quality of chronic myeloid leukemia (CML) patients. However, IM resistance remains a critical clinical challenge due to a lack of specific biomarkers for a subset of patients. Recently, growing evidence has suggested that exosomes (Exo) were involved in tumor resistance and can be an excellent reservoir of novel biomarkers and candidate therapeutic targets for cancer. Therefore, in this study, we aimed to investigate the proteomic profile of plasma exosomes derived from imatinib‐resistant CML and imatinib‐sensitive CML to identify a new biomarker for IM resistance.

**Methods**: Exosomes were extracted from pooled plasma samples of 9 imatinib‐resistant CML and 9 imatinib‐sensitive CML by Total Exosome Isolation Reagent. Then, the expression levels of exosomal proteins were identified by liquid chromatograph‐mass spectrometer (LC‐MS/MS), and bioinformatic analyses were used to analyse the proteomic data. Finally, the western immunoblotting (WB) and Parallel reaction monitoring (PRM) analyses were applied to validated the candidate proteins in individual subjects.

**Results**: A total of 2812 proteins were identified in plasma exosomes from imatinib‐resistant CML and imatinib‐sensitive CML, including 279 differentially expressed proteins (DEPs) with restricted criteria (p < 0.05, fold change>1.5). Compared with imatinib‐sensitive CML, 151 proteins were up‐regulated and 128 proteins were down‐regulated. Bioinformatics analysis revealed that the upregulated proteins were enriched in the process of the protein synthesis, whereas the downregulated proteins were largely involved in lipid metabolism. The top 20 hub genes were obtained using STRING and Cytoscape, most of which are the components of ribosomes. These 20 key proteins were further verified by PRM, and 18 proteins were consistent with the mass spectrometry analysis. Among these proteins, EIF3B, EIF3J, and EIF3K exhibited significant differences between imatinib‐resistant CML and imatinib‐sensitive CML, and may plays an important role in the development of Imatinib resistance in CML. Finally, the 3 DEPs (EIF3B, EIF3J, and EIF3K) were verified again by WB in the plasma derived exosomes from imatinib‐resistant CML and imatinib‐sensitive CML.

**Summary/Conclusion**: We identified differences in the plasma exosome proteome between imatinib‐resistant CML and imatinib‐sensitive CML. The exosomal protein (EIF3B, EIF3J, and EIF3K) may serve as biomarkers for imatinib resistance in CML.

### PS17.07 Proteomics of small extracellular vesicles produced by non‐malignant cells in plasma of healthy donors and cancer patients

Aneta Zebrowska, Maria Sklodowska‐Curie National Research Institute of Oncology, Gliwice Poland

Marta Gawin, Maria Sklodowska‐Curie National Research Institute of Oncology, Gliwice Poland

Lucyna Ponge, Maria Sklodowska‐Curie National Research Institute of Oncology, Gliwice Poland

Piotr Widłak, Maria Sklodowska‐Curie National Research Institute of Oncology, Gliwice Poland

Sujan Mondal, UPMC Hillman Cancer Center, University of Pittsburgh Cancer Institute

Soldano Ferrone, Massachusetts General Hospital, Harvard University

Theresa L. Whiteside, UPMC Hillman Cancer Center, University of Pittsburgh Cancer Institute

Monika Pietrowska, PhD, Maria Sklodowska‐Curie National Research Institute of Oncology

**Introduction**: The role of small extracellular vesicles (sEV) derived from non‐malignant cells in cancer plasma may be as important in cancer biology as is the role of sEV produced by tumor cells. Preliminary studies showed that the molecular profile of sEV derived from non‐malignant cells in cancer contributed to suppression of anti‐tumor immune responses and reprogramming of the tumor microenvironment. To confirm this observation, here we use immunoaffinity‐based capture to separate plasma sEV into the sEV subsets produced by malignant and non‐malignant cells.

**Methods**: Plasma samples were obtained from 10 healthy donors (HDs) and 10 patients with metastatic melanoma. sEV isolated by ‐Size‐Exclusion Chromatography (SEC) and recovered in fraction #4 were immunocaptured with anti‐CD63/CD81mAbs in HDs or with anti‐CSPG4 Abs in patients. Proteomic profiles of the following sEV subsets were obtained by high resolution mass spectrometry (HRMS) and compared: (i) total sEV isolated from HD's plasma, (ii) CD63+/CD81+ sEV isolated from HD's plasma, (iii) CSPG4(neg) sEV from patients' plasma.

**Results**: Proteomic profiles of the CSPG4(neg) sEV derived from non‐malignant cells in cancer plasma significantly differed from profiles of CD63+/CD81+ sEV isolated from plasma HDs. We identified about 800 proteins/sEV fraction. Differences in the protein cargo indicated a higher content of immunosuppressive proteins in the CSPG4(neg) cancer sEVs than HD's sEV. This was also confirmed by functional studies using sEV coincubated with primary immune cells.

**Summary/Conclusion**: The proteome of CSPG4 (neg) sEV from melanoma patients’ plasma and their anti‐tumor functions were not comparable to those of CD63/CD81+ sEV from HD's plasma, suggesting reprogramming by cancer of non‐malignant cells. The latter are a source of sEV with immunosuppressive functions similar to those mediated by melanoma‐cell‐derived sEV.

### PS17.08 Single Versus Multi‐Stressor Conditions Alter Extracellular Vesicle Size and Surface Composition Differently in Men and Women

William R. Conkright, MS, RD, CSSD, CSCS, University of Pittsburgh

Meaghan E. Beckner, MS, University of Pittsburgh

Qi Mi, PhD, Neuromuscular Research Lab / Warrior Human Performance Research Center, University of Pittsburgh, Pittsburgh, PA, USA

Amrita Sahu, PhD, McGowan Institute for Regenerative Medicine, University of Pittsburgh, Pittsburgh, PA, USA

Zachary J. Clemens, MS, McGowan Institute for Regenerative Medicine, University of Pittsburgh, Pittsburgh, PA, USA

Mita Lovalekar, PhD, Neuromuscular Research Lab / Warrior Human Performance Research Center, University of Pittsburgh, Pittsburgh, PA, USA

Shawn D. Flanagan, PhD, Neuromuscular Research Lab / Warrior Human Performance Research Center, University of Pittsburgh, Pittsburgh, PA, USA

Fabrisia Ambrosio, PhD, McGowan Institute for Regenerative Medicine, University of Pittsburgh, Pittsburgh, PA, USA

Bradley C. Nindl, PhD, Neuromuscular Research Lab / Warrior Human Performance Research Center, University of Pittsburgh, Pittsburgh, PA, USA

**Introduction**: Extracellular vesicles (EVs) have been implicated as mediators of tissue crosstalk with downstream implications in pathological and non‐pathological processes. Singular stressors, such as exercise, alter circulating concentration and composition of EVs, whereas the effect of multi‐stressor environments, which are common in real‐world settings, is poorly understood. We tested the hypothesis that combined stress, including exercise together with sleep and caloric restriction, affects EV size and surface protein composition differently than exercise alone in men and women.

**Methods**: All procedures were approved by the University of Pittsburgh IRB. Following informed consent, 20 subjects (10 men: 25.6±5.8 y, 178.3±7.2 cm, 81.4±7.8 kg, 18.8±4.2 body fat %; 10 women: 27.1±5.9 y, 169.0±7.7 cm, 71.0±8.1 kg, 28.2±7.0 body fat %) completed a 90 min physical exertion protocol daily for 4 days. On days 2 and 3, caloric intake and sleep were reduced by 50%. Creatine kinase (serum), myoglobin (serum), and isolated EVs (plasma) were analyzed from blood drawn before and immediately after exercise on day 1 (D1) and day 3 (D3). EVs were probed for markers associated with exosomes (CD63) and skeletal muscle (SGCA) using imaging flow cytometry. EV features were stratified based on brightfield (60x) area (small: < 0.031; medium: 0.031"0.785; large: >0.785 μm2) and analyzed for the effect of sex, day, and time, on size and fluorescent intensity of EV markers using three‐way ANOVAs.

**Results**: From D1 to D3, the proportion of small EVs increased 7% (p = 0.029) and large EVs decreased 18% (p = 0.015) in men only, with no change in proportion of medium EVs in either sex. CD63 intensity increased 511% (p = 0.014) from pre‐ to post‐exercise and 322% (p = 0.045) after 48 h of sleep and caloric restriction in both sexes. A day*time interaction (p = 0.021) was present for CD63 intensity in the small EV subpopulation, increasing 77% from pre‐ to post‐exercise on D3, but no change on D1 (p = 0.014 vs. p = 0.492). SGCA intensity increased 29% (p = 0.019) following exercise and 40% from D1 to D3 (p = 0.001) in both sexes. In agreement with these results, markers of muscle damage, including myoglobin (135%; p < 0.001) and creatine kinase (9%; p = 0.061) also increased across time and day, respectively, in men and women.

**Summary/Conclusion**: Acute sleep and caloric restriction may have an additive effect on small EV surface cargo not present under a single stress (exercise) condition. Single and multi‐stress conditions cause an increase in SGCA intensity, which may indicate greater contribution of circulating skeletal muscle‐derived EVs during combined sleep restriction, underfeeding, and exercise or exercise alone. Future studies may be warranted to investigate the implications of differential shifts in EV size across multi‐stress conditions in men and women.

### PS17.09 Surface enhanced Raman scattering of extracellular vesicles for cancer diagnostics despite isolation dependent lipoprotein contamination

Hanna J. Koster, UC Davis

Tatu Rojalin, MD PhD, UC Davis

Alyssa Powell, UC Davis

Dina Pham, UC Davis

Rachel R. Mizenko, UC Davis

Andrew Birkeland, M.D., UC Davis

Randy Carney, UC Davis

**Introduction**: Given the emerging diagnostic utility of extracellular vesicles (EVs), it is important to account for non‐EV contaminants. Lipoprotein present in EV‐enriched isolates may inflate particle counts and decrease sensitivity to biomarkers of interest, skewing chemical analyses and perpetuating downstream issues in labeling or functional analysis.

**Methods**: We used label free surface enhanced Raman scattering (SERS) to examine isolates from clinical biofluids using three common EV isolation methods (differential ultracentrifugation, density gradient ultracentrifugation, and size exclusion chromatography).

**Results**: We found that the type of lipoproteins co‐isolated with a given EV preparation varied depending upon the technique that was used. EVs isolated by SEC grouped with chylomicrons and VLDL/LDL, while EVs isolated by UC and DG shared more overlap with each other and HDL. However, combining SERS analysis with machine learning assisted classification, we show that the disease state is the main driver of distinction between EV samples, with a diagnostic accuracy of >97% for EVs isolated from blood of head and neck cancer patients. Interestingly, accuracy was largely unaffected by choice of isolation method. A protocol of subsequent UC and SEC largely eliminated lipoprotein contamination, yet diagnostic accuracy suffered (dropping to 87%).

**Summary/Conclusion**: This study describes a convenient SERS assay to obtain accurate diagnostic information from clinical samples while overcoming differences in lipoprotein contamination according to isolation method. Our rapid, inexpensive, and label‐free platform was sensitive enough to tease out cancer‐specific signatures using microliter volumes of biofluids isolated using either UC, SEC, or DG.

### PS17.10 NanoBioAnalytical (NBA) Platform for EVs quantification and sizing: The analytical performance

Balasubramaniam NAMASIVAYAM, Institute FEMTO‐ST

Céline Elie‐Caille, Institute FEMTO‐ST

Wilfrid Boireau, Institute FEMTO‐ST

**Introduction**: Challenges in the reliable characterization of EVs are well known owing to the biological sample heterogeneity and complexity for EVs from diverse biological origins. The NBA platform is a combination of biophysical characterization techniques integrating Surface Plasmon Resonance‐imaging (SPRi) system and Atomic Force Microscopy (AFM). Utilized in complementary with an in‐solution particle quantification method such as Tuneable Resistive Pulse Sensing (TRPS), the NBA platform can serve as a powerful tool for the multifaceted characterization of EVs. The current work explores the limit of the sensitivity and the applicable range of concentration of platelet‐derived EVs for a consistent estimation of EVs characteristics such as expression of a specific marker and their size parameters.

**Methods**: EVs from human platelets are isolated by centrifugation,2x of 3000g for 15 minutes and 1 × 20000g for 90 minutes. The EVs concentration is estimated using TRPS. EVs are immunocaptured on a biochip functionalized with a‐CD41 antibodies in an SPRi system. The EVs are then imaged by AFM. The detected signal from SPRi measurement is processed using the negative control subtraction method as the negative control signal is not specific to EVs thus subtracted for differentiating the specific signal and noise. AFM images are treated in JPK data processing software and particle (EVs) analysis is performed using Gwyddion software. Data analysis of the SPRi and AFM quantification are performed using a custom‐written software code.

**Results**: The current study allowed establishing the limit of detection of the NBA platform for characterizing platelet‐derived EVs expressing CD41 and injected at a rate of 20 μL/minute for 9 minutes. The result showed that the system is sensitive at e10^5 EVs/mL level (Estimated average concentration: 1.62e05 EVs/mL) and has a wide dynamic range up to 10^9EVs/mL (estimated average concentration of 1.98e09 EVs/mL). This analytical performance underscored the sensitivity and selectivity of the NBA system for characterizing heterogeneous EVs in a complex media, in a one‐step bio‐detection procedure without amplification. From our knowledge, detection performances got in this study are the highest in the field of plasmonic sensors without amplification procedure (Chin et al., ACS Nano 2020,). Moreover, AFM quantification data even showed a difference in EVs size distribution profile between high and low concentration ranges, highlighting the (bad) influence of EV sample with a high dilution factor in a functional context.

**Summary/Conclusion**: The NBA system offers a reliable estimation of EVs subsets concentrations and metrology, under flow conditions and associated with a specific marker. The multiplexed, label‐free method remains unique characterization tool for biological EVs subsets coexisting in a complex biological sample.

### PS17.11 Determining the Benchmark Proteome of Breath Exosomes

Deanna Ayupova, Victoria Univeristy of Wellington

Lifeng Peng, School of Biological Sciences, Victoria University of Wellington

Paul Teesdale‐Spittle, School of Biological Sciences, Victoria Univeristy of Wellington

**Introduction**: Exosomes from breath have been reported but not fully characterised. As with exosomes from other sources, breath‐derived exosomes have potential in diagnostic applications, particularly those relating to lung health. In this study we characterised the proteome of exosomes obtained from the breath of healthy volunteers. This proteome may be used as a benchmark of breath exosomes for assessment of patients with impaired lung function in the future.

**Methods**: Preliminary characterisation of the proteome of breath‐derived exosomes was conducted through studies with 60 healthy participants that were divided into 3 groups (20 volunteers in each). Breath‐derived exosomes were isolated from exhaled breath condensate by ultracentrifugation and then characterized by conventional methods. Proteins were isolated by solubilisation in 8 M urea, precipitated, and then subjected to tryptic digestion.The resulting tryptic peptides were analysed by liquid chromatography mass spectrometry in an Ultimate3000 and an Orbitrap Fusion Lumos mass spectrometer system (ThermoFisher Scientific). The spectra were searched against the SwissProt human protein database (TAXID = 9606, v2017) using Proteome Discoverer (v2.4 ThermoFisher Scentific) to identify the proteins. Identified proteins were subjected to gene ontology (GO) functional enrichment analysis finding cellular components (CC) and molecular functions (MF) of common proteins across 3 samples.

**Results**: During this study, 720 proteins in total were found across 3 samples from breath‐derived exosomes. As expected, many of these proteins were annotated to “regulated exocytosis”, although 535 proteins were associated with other ontologies. Further analysis revealed 194 proteins were found at least twice and not characterized as “regulated exocytosis” proteins. The GO analysis indicated that these proteins were associated with “extracellular space”, “extracellular exosomes”, and “extracellular vesicles” for CC. The top MF associated with these proteins are “antigen binding”, “Immunoglobulin receptor binding”, and “structural constituent of cytoskeleton”.

**Summary/Conclusion**: Our proposed use of breath exosomes is in diagnosis of lung health. The benchmark proteome that we have determined of exosomes from the breath of healthy volunteers may be used in future studies identifying new biomarkers of lung dysfunction.

### PS17.12 Characterization of extracellular vesicles by FT‐IR spectroscopy: a specific spectral signature of drug resistant osteosarcoma‐derived extracellular vesicles

FRANCESCA PERUT, BST Biomedical Sciences and Technologies Lab, IRCCS Istituto Ortopedico Rizzoli

Gabriela Graziani, Laboratory of Nanobiotechnology (NaBi), IRCCS Istituto Ortopedico Rizzoli

Laura Roncuzzi, BST Biomedical Sciences and Technologies Lab, IRCCS Istituto Ortopedico Rizzoli

Nicoletta Zini, CNR Institute of Molecular Genetics “Luigi Luca Cavalli‐Sforza”, Unit of Bologna

Sofia Avnet, Department of Biomedical and Neuromotor Sciences, University of Bologna

Nicola Baldini, Department of Biomedical and Neuromotor Sciences, University of Bologna

**Introduction**: Osteosarcoma (OS) is the most common primary bone cancer in children and adolescents. Despite aggressive treatment regimens, the outcome is unsatisfactory, and drug resistance is a pivotal process in OS treatment failure. Extracellular vesicles (EVs) promote resistance to chemotherapy and target therapy through different mechanisms. EVs secreted from drug resistant cells are able to transfer drug resistant traits between cancer cells, and to improve the survival of resistant cancer clones which constitute a reservoir of minimal residual disease. In this study, we investigated the possibility to characterize subpopulations of OS‐derived EVs by Fourier Transform Infrared Spectroscopy (FT‐IR) to define a specific spectral signature for the drug resistant OS‐derived EVs.

**Methods**: EVs were isolated from MG‐63 (EVs/s), doxorubicin‐resistant MG‐63DXR30 (EVs/DXR) cell lines, and human mesenchymal stem cells (MSC) by differential centrifugation and ultracentrifugation. EVs morphology and size was assessed by TEM. The expression of P‐gp, CD63, CD9, CD81, and hsp70 was verified by western blot analysis. EVs pellets were analysed by FT‐IR/ATR (Perkin Elmer Spectrum 2), using the following parameters: acquisition range 400"4000 cm'1, resolution 4 cm'1 and 128 scans. As a reference for the analysis, phosphate‐buffered saline solution was acquired.

**Results**: EVs/s, EVs/DXR and MSC‐derived EVs showed the expected morphology and size range (30‐100nm). EVs markers were enriched in EVs preparations compared to cell lysates. P‐gp protein expression was observed in MG‐63DXR30 cells and EVs/DXR. The amount of EVs released by MG‐63DXR30 cells was significantly higher than that released by MG‐63.

FT‐IR spectra were consistently different between the EVs and cells from which they originate (in the 1300‐1000 cm‐1 area). A specific spectral signature, characterized by a shift and new band (1601cm‐1), allow to clearly distinguish EVs isolated by MG‐63 and MG‐63DXR30 cells. Additionally, we identified a different spectra profile and spectroscopic protein‐to‐lipid ratio from OS and MSC‐derived EVs.

**Summary/Conclusion**: Our findings suggest that FT‐IR spectroscopy allows to characterize and define specific spectral signature for human OS derived EVs and particularly for drug resistant OS cells‐derived EVs.

### PS17.13 A combined method to isolate blood plasma extracellular vesicles to support Small RNAs sequencing for biomarkers discovery

Daniela Fignani, Diabetes Unit, Department of Medicine, Surgery and Neurosciences, University of Siena, Siena, Italy.

Giuseppina E. Grieco, Diabetes Unit, Department of Medicine, Surgery and Neurosciences, University of Siena, Siena, Italy.

Noemi Brusco, Diabetes Unit, Department of Medicine, Surgery and Neurosciences, University of Siena, Siena, Italy.

Giada Licata, Diabetes Unit, Department of Medicine, Surgery and Neurosciences, University of Siena, Siena, Italy.

Laura Nigi, Diabetes Unit, Department of Medicine, Surgery and Neurosciences, University of Siena, Siena, Italy.

Caterina Formichi, Diabetes Unit, Department of Medicine, Surgery and Neurosciences, University of Siena, Siena, Italy.

Guido Sebastiani, Diabetes Unit, Department of Medicine, Surgery and Neurosciences, University of Siena, Siena, Italy.

Francesco Dotta, Diabetes Unit, Department of Medicine, Surgery and Neurosciences, University of Siena, Siena, Italy.

**Introduction**: Extracellular Vesicles (EVs) are mediators of tissues crosstalk and contain potential biomarkers. However, it is still a challenge to straightforwardly isolate them with high specificity and purity. The aim of this study is to establish a protocol to isolate a highly pure fraction of human blood plasma EVs to support Small RNAs sequencing for biomarkers discovery.

**Methods**: EVs from 200 μl of plasma from healthy subjects were isolated using: (i) Size Exclusion Chromatography (SEC) (EVs < 200 nm); (ii) lectin‐based affinity isolation procedure (Capturem) (based on EVs surface glycoproteome); (iii) a sequential combination of SEC+Capturem. EVs were analyzed using Nanoparticle Tracking Analysis (NTA) and Transmission Electron Microscopy (TEM). Protein content was evaluated using 2D‐DIGE and Small RNAs using Small RNA‐seq (QIAseq).

**Results**: The NTA analysis revealed that we were able to isolate 10.3 × 1010, 3.9 × 109 and 4.2 × 108 particles/ml from SEC, Capturem and SEC+Capturem, respectively. All the three methods retrieved a median particle size of 67.6‐70.3 nm. TEM confirmed the presence of cup‐shaped vesicles with exosomes‐compatible morphology and size. EVs isolated using SEC+Capturem were fewer in number but free from contaminants. 2D‐DIGE proteome analysis confirmed the higher purity of EVs isolated using the sequential approach.

EVs RNA and whole plasma were subjected to Small RNA‐seq. Small RNA libraries QC steps showed a peak around 170–180bp, in accordance with the presence of Small RNAs. Small RNA‐seq revealed that the percentage of miRNAs reads found in whole plasma was higher than in EVs (25% vs 4%, respectively). However, EVs showed an higher number of miRNAs (SEC:1985, Capturem:2046; SEC+Capturem:1998) vs whole plasma (1482), revealing a high miRNAs diversity in EVs, compatible with their role in cell‐cell communication and miRNAs selective packaging. In EVs we detected unique miRNAs respect to whole plasma (SEC:514, Capturem: 574; SEC+Capturem: 525). Finally, the combined approach was able to deplete 99,9 % of free‐circulating AGO2‐associated miRNAs.

**Summary/Conclusion**: In conclusion, we reported a novel protocol to isolate EVs from 200 μl of plasma with high purity and adequate concentration, using a two sequential isolation steps approach to support small RNA‐seq and expanding our ability to discover novel EVs associated biomarkers.

### PS17.14 The human gut bacteria Bacteroides thetaiotaomicron releases extracellular vesicles enriched in proteins that influence host cell physiology and metabolism

Regis Stentz, Quadram Institute Bioscience

Udo Wegmann, University of East Anglia

Maria Guirro, Eurecat

Will Bryant, Great Ormond Street Hospital for Children NHS Foundation Trust

Avani Ranjit, University of East Anglia

Andrew Goldson, Quadram Institute Bioscience

Arlaine Brion, Quadram Institute Bioscience

Kathryn Gotts, Quadram Institute Bioscience

Catherine Booth, Quadram Institute Bioscience

Ariadna Miquel‐Clopes, Quadram Institute Bioscience

Emily Jones, Quadram Institute Bioscience

Patrick GunningQuadram Institute Bioscience

Padhmanand Sudhakar, TARGID, KU Leuven

Dezső Módos, Quadram Institute Bioscience

Ian Brown,University of Kent

Tamás Korcsmáros,Earlham Institute

Simon Carding, Quadram Institute Bioscience

**Introduction**: It is increasingly apparent that bacterial extracellular vesicles (BEVs) produced by members of the intestinal microbiota contribute to microbe‐host cell interactions. Unresolved questions are, what is the nature of the cargo packaged into BEVs and how do they impact on host cell function? Here we analyzed the proteome of BEVs produced by the major human gut symbiont Bacteroides thetaiotaomicron (Bt) under in vitro cultures using minimal and complex media, and in vivo in fed or fasted animals to determine the impact of nutrient stress on the BEV proteome, and identify proteins enriched in BEVs produced in vivo.

**Methods**: In vitro conditions consisted of cultures of Bt grown in complex or minimal media. In vivo studies consisted of orally administering Bt to germfree mice and collecting cecal contents 3 days later. Vesicles from culture and cecal contents were concentrated by crossflow filtration, separated by size exclusion chromatography and recovered by ultracentrifugation followed by protein extraction. Differential expression of BEV proteins obtained under different conditions were explored using tandem mass tagging (TMT) combined with liquid chromatography mass spectrometry (LC‐MS/MS). Data sets were analyzed using the Proteome Discoverer v2.1 software.

**Results**: BEVs produced in vitro where limiting nutrient provision resulted in an increase in a large fraction of proteins. By contrast, BEV proteins from fasted versus fed mice were less affected with similar numbers of proteins showing increased and decreased abundance. We identified 102 proteins exclusively enriched in BEVs in vivo of which the majority (66/102) were enriched independently of their expression in parental cells. These abundantly expressed proteins included enzymes that were isolated and characterised further and shown to be active and able to degrade host cell‐derived substrates.

**Summary/Conclusion**: These findings provide new insights into the role BEVs play in microbiota‐host interactions with their contents capable of playing key roles in the maintenance of intestinal homeostasis and host metabolism.

### PS17.15 Antigen‐induced eosinophilic respiratory inflammation alters the protein cargo of lung‐derived EVs in mice

Cecilia Lässer, PhD, University of Gothenburg

Yassunari Kishino, Department of Medicine, Division of Respiratory Medicine and Allergology at Showa University, Tokyo, Japan

Jan Lötvall, Krefting Research Centre, Institute of Medicine at Sahlgrenska Academy at the University of Gothenburg, Gothenburg, Sweden.

**Introduction**: Analysis of the proteome of tissue‐derived EVs is of great importance both to identify biomarkers of disease but also to understand cell‐to‐cell communication in diseased tissue. The aim of this study was to establish an isolation method that isolates lung vesicles of high purity for proteomic analysis and to determine the proteome of lung tissue‐derived vesicles during an antigen‐induced eosinophilic respiratory inflammation in mice.

**Methods**: A mouse model for allergic asthma was used by sensitization and challenge of BALB/c mice to ovalbumin (OVA). Animals were sacrificed and lungs were removed and chopped in to smaller pieces that were incubated in media with DNase 1 and Collagenase D for 30 minutes at 37°C (Crescitelli et al JEV 2020). Vesicles were isolated from the medium by ultracentrifugation and bottom loaded iodixanol density cushion. Isolated vesicles were evaluated by electron microscopy (EM) and the proteome was analysed with mass spectrometry (LC‐MS/MS, N∈‐6).

**Results**: Electron microscopy showed that the protocol isolated vesicles that where on average 40–200 nm in size. In total 4510 proteins were quantified in all samples. The identified proteins were analyzed with DAVID to identify enriched cellular components compared to the genome frequency, and the top associated terms were “Extracellular exosome” and “Membrane”. Principle component analysis showed that component 1, representing 40% of the variability, distinguished the OVA‐EVs from the PBS‐EVs. Over 1000 proteins were significantly altered (fold change >2 and p‐value < 0.05), with 614 proteins being up‐regulated and 425 proteins being down‐regulated in OVA‐EVs. The 614 proteins upregulated during allergen‐induced inflammation was mainly associated with the GO biological processes terms; “ribosomal units”, “translation”, “mRNA processing”, “immune system processes”, “innate immune response”, “response to virus” and “B cell receptor signaling pathway”. The majority of the top‐15 most upregulated proteins were associated the immune related terms.

**Summary/Conclusion**: Extracellular vesicles can be isolated from mouse lung tissue and these vesicles are highly associated with previously identified proteins in extracellular vesicles. In EVs present in OVA/OVA mice immune associated proteins were upregulated reflecting the ongoing antigen‐induced eosinophilic respiratory inflammation, suggesting that airway‐derived EVs can be altered in diseases with inflammation of the lung, such as asthma.

## PS18 EVs & The Liver

Chair: Hernando Del Portillo, ISGlobal, Hospital Clínic ‐ Universitat de Barcelona. Institute for Health Sciences Trias I Pujol (IGTP), Badalona, Spain. Catalan Institution for Research and Advanced Studies (ICREA), Barcelona, Spain

### PS18.01 Anoectochilus roxburghii derived exosomes‐like nanovesicles (AELVs) protect against alcohol‐induced liver damage

Lupeng Zeng, Fujian Medical University

Huaying Wang, Fujian Medical University

Xiu Zou, Fujian Medical University

Wanhua Shi, Fujian Medical University

Tingting Chen, Fujian Medical University

Guanyu Chen, Fujian Medical University

Wenqian Chen, Fujian Medical University

Lilan Xu, Fujian Medical University

Jinghua Chen, Fujian Medical university

**Introduction**: Alcoholic liver disease is one of the most prevalent chronic liver diseases worldwide, representing one of the main etiologies of cirrhosis and hepatocellular carcinoma. The traditional Chinese medicine, anoectochilus roxburghii, has been reported to alleviate alcoholic liver injury. However, whether its derived exosomes‐like nanovesicles (AELVs) with similar functions has not been reported.

**Methods**: The control group only treated with PBS, other groups were administered by oral gavage with PBS, silybin, AELVs for 9 days and then the mice were treated with 50% alcohol to induce acute alcohol liver damage model. After a 12h fast, the mice were euthanized, the serums and livers were harvested for examination. The liver injury relevant indexes of alanine aminotransferase (ALT), aspartate aminotransferase (AST), triglyceride (TG) and cholesterol (CHOL) in serum were detected, and analyzed by Graphpad prism 8. The livers were sectioned and stained with H&E for pathological examination.

**Results**: In the AELVs group, the serum ALT, AST, TG and CHOL were significantly lowered, when compared to the alcoholic liver injured model group, which was beneficial to reduce the risk of alcoholic fatty liver disease after acute alcohol consumption. Meanwhile, pathological examination also indicated that alcohol‐induced hepatocellular injury was improved by the AELVs treatment. Additionally, all the results of AELVs group presented better than the commercial liver protection agent, silybin, which manifested AELVs had great potency of being the liver protector.

**Summary/Conclusion**: The AELVs have great liver protective effect which expected to develop a novel liver protective agent. Meanwhile, AELV as the exosomes‐like vesicle is a potential drug carrier, which provides a new option for the drug delivery of the liver diseases therapy.

### PS18.02 Comparative analysis of miRNA biomarkers in total circulating RNA and vesicular RNA from global and liver‐derived extracellular vesicles in non‐alcoholic fatty liver disease

Lauren A. Newman, Flinders University

Zivile Useckaite, Flinders University

Andrew Rowland, Flinders University

**Introduction**: Non‐alcoholic fatty liver disease (NAFLD) is the most common chronic liver disease globally and manifests on a spectrum of severity from simple steatosis to non‐alcoholic steatohepatitis (NASH). Definitive NASH diagnoses require liver biopsy, but the invasiveness of this technique poses risks to patients and does not lend itself to ongoing evaluation of disease. Altered circulating miRNA profile shows potential for minimally invasive tracking of NAFLD. miRNA circulates bound to proteins or within extracellular vesicles (EVs). Compared to total circulating miRNA, that isolated from EVs, particularly tissue‐specific EVs, may provide a more disease‐specific source of biomarkers due to selective miRNA packaging that reflects parent cell physiology.

**Methods**: Biobanked plasma samples from patients with NAFL, biopsy‐confirmed NASH and matched healthy controls were obtained. Global EVs were isolated by size exclusion chromatography and liver‐derived EVs were immunoprecipitated with anti‐asialogylcoprotein receptor 1 (ASGR1) antibody‐conjugated magnetic beads. Morphology, size and concentration of global EVs were assessed by TEM and NTA. Total circulating RNA and vesicular RNA were isolated by phenol‐chloroform extraction and expression of miR ‐122, ‐192 and ‐128‐3p quantified by RT‐qPCR.

**Results**: Patterns of biomarker expression varied in each source of RNA (total/global EV/liver EV). While significant dysregulation of miRNA biomarkers was observed in total miRNA in NAFLD, analysis of ASGR1+ EVs produced a clear directional trend with increasing miR ‐122 and ‐192 expression in disease. Capacity for miR‐128‐3p to distinguish groups was not improved by sample fractionation.

**Summary/Conclusion**: miRNA may be useful for tracking disease and treatment response in NAFLD patients, but the specific source of circulating miRNA is an important consideration for biomarker development. Liver‐specific EVs present a particularly informative source, but this should be assessed with respect to individual markers.

### PS18.04 Extracellular vesicles: Natural liver‐accumulating drug delivery vehicles for the treatment of liver diseases

Gensheng Zhang, Department of Critical Care Medicine of the Second Affiliated Hospital, Zhejiang University School of Medicine

Xiaofang Huang, Department of Critical Care Medicine of the Second Affiliated Hospital, Zhejiang University School of Medicine

Huiqing Xiu, Zhejiang University School of Medicine

Yan Sun, Department of Comprehensive Medical Oncology, Zhejiang Cancer Hospital

Jiming Chen, Institute of Immunology, and Department of Orthopedics of the Second Affiliated Hospital, Zhejiang University School of Medicine

Guoping Cheng, Department of Pathology, Zhejiang Cancer Hospital

Zhengbo song, Department of Medical Oncology, Zhejiang Cancer Hospital

Yanmei Peng, Institute of Immunology, and Department of Orthopedics of the Second Affiliated Hospital, Zhejiang University School of Medicine

Yingying Shen, Institute of Immunology, and Department of Orthopedics of the Second Affiliated Hospital, Zhejiang University School of Medicine

Jianli Wang,Institute of Hematology, Zhejiang University & Zhejiang Engineering Laboratory for Stem Cell and Immunotherapy

Zhijian cai, Zhejiang University

**Introduction**: Extracellular vesicles are excellent vectors for the delivery of therapeutic drugs. However, biological safety and disease targeting issues greatly limit their clinical application. Extracellular vesicles from red blood cells (RBC‐EVs) have been reported to be ideal deliverers of RNA drugs because of their unique biological safety profile. We aimed to explore the potential use of drug‐loaded RBC‐EVs for clinical applications for liver disease therapy.

**Methods**: RBC‐EVs were labeled with fluorescent dyes, and their distribution in vivo was detected by an in vivo imaging system and fluorescence microscopy after intravenous injection. Antisense oligonucleotides of miR‐155, Doxorubicin or Sorafenib were loaded into RBC‐EVs by electroporation. D‐galactosamine‐ and LPS‐induced acute liver failure model mice were used to assess the protective effects of RBC‐EVs

loaded with antisense oligonucleotides of miR‐155 on acute liver failure. A murine orthotopic liver cancer model was used to evaluate the therapeutic effects of RBC‐EVs loaded with Doxorubicin or Sorafenib on liver cancer. The toxicity of drug‐loaded RBC‐EVs was estimated by blood biochemical testing, histopathological examination and immunofluorescent staining.

**Results**: RBC‐EVs showed natural liver tropism. Mechanistically, the liver environment induces macrophages to phagocytize RBC‐EVs in a C1q‐dependent manner. RBC‐EVs loaded with antisense oligonucleotides of miR‐155 showed pronounced protective effects in D‐galactosamine‐ and LPS‐induced acute liver failure model mice that were depending on macrophages. Furthermore, compared to routine doses of Doxorubicin and Sorafenib, RBC‐EVs loaded with Doxorubicin or Sorafenib showed enhanced therapeutic effects on murine orthotopic liver cancer depending on macrophages. Importantly, drug‐loaded RBC‐EVs showed no systemic toxicity, whereas routine doses of Doxorubicin and Sorafenib showed obvious toxicity.

**Summary/Conclusion**: Drug‐loaded RBC‐EVs are natural liver‐targeting reagents. RBC‐EVs loaded with antisense oligonucleotides of miR‐155 or antitumor drugs can be used to treat acute liver failure or liver cancer, respectively. Thus, drug‐loaded RBC‐EVs hold high potential for clinical applications for liver disease therapy.

### PS18.05 Liver derived extracellular vesicles characterize risk of metabolic drug interactions

Andrew Rowland, Flinders University

Michael Sorich, Flinders University

**Introduction**: Cytochrome P450 (CYP) 3A4 is the most important drug metabolizing enzyme as it is responsible for the clearance of more than 30% of all drugs used in clinical practice. Metabolic drug interactions involving induction of CYP3A4 expression reduce efficacy and represent a major source of variability in treatment outcomes. The ability to predict and the magnitude of CYP3A4 induction can inform treatment decisions and lead to more effective drug dosing. The primary aim of this study was to evaluate the capacity of small extracellular vesicle (sEV) derived biomarkers to predict the magnitude of CYP3A4 induction.

**Methods**: Liver‐derived sEV were isolated from the serum of healthy males (n = 9) by size exclusion chromatography followed by immunoprecipitation, before and after administration of the strong CYP3A4 inducer rifampicin. The abundance of sEV derived CYP3A4 protein was quantified by liquid chromatography / mass spectrometry and correlated with exposure to the CYP3A4 probe substrate midazolam.

**Results**: Proteomic analysis revealed induction (mean fold‐increase, 90% confidence interval) of liver sEV CYP3A4 protein expression following rifampicin dosing for 7 days at a dose of 300 mg/day [3.5 (2.5 to 4.5), p = 0.029] and 14 days at a dose of 600 mg/day [3.7 (2.1 to 6.0), p = 0.018] was consistent with the mean oral midazolam area under the plasma concentration time curve ratio in the same subjects of 3.6 (2.9 to 4.5) and 5.9 (4.5 to 8.3), respectively.

**Summary/Conclusion**: Consistent strong concordance was observed between the change in sEV‐derived CYP3A4 protein expression and the change in midazolam exposure. The significance of these data is that CYP3A4 is the drug‐metabolizing enzyme of greatest clinical importance and variability in CYP3A4

activity is poorly described by existing strategies.

### PS18.06 pH‐responsive functionalized extracellular vesicles as targeted doxorubicin delivery vehicles for the treatment of hepatocarcinoma

Ying Luo, PhD, Tianjin Key Laboratory of Extracorporeal Life Support for Critical Diseases, Tianjin Institute of Hepatobiliary Diseases, Tianjin Third Central Hospital

Yingtang Gao, PhD, Tianjin Key Laboratory of Extracorporeal Life Support for Critical Diseases, Tianjin Institute of Hepatobiliary Diseases, Tianjin Third Central Hospital

Yijun Wang, Department of Hepatobiliary Surgery, Tianjin Third Central Hospital

Yuyu Luo, The Third Central Clinical College of Tianjin Medical University

**Introduction**: Hepatocellular carcinoma (HCC) is a progressive development of fatal neoplastic disease. Accumulated evidence has demonstrated that extracellular vesicles (EVs) can be considered as a promising drug carrier for the delivery of antitumor drugs due to their ideal size range and low immunogenicity. Herein, considering that the internal pH of tumor tissue is lower than normal tissue, we combined the pH‐responsiveness of carboxymethyl chitosan (CMC) and the potential advantages of EVs, to further investigate the treatment efficiency of chemotherapy drug‐loaded EVs coated with CMC in HCC.

**Methods**: EVs were isolated from HepG2 cells and further loaded them with the chemotherapeutic drug doxorubicin (Dox). Furthermore, we coated the Dox‐loaded EVs (Dox@EVs) with CMC to form a complex (Dox@EVs‐CMC). Briefly, Dox@EVs‐CMC were characterized by transmission electron microscopy (TEM), dynamic light scattering (DLS) and western blotting (WB). The cytotoxicity of Dox@EVs‐CMC on cancer cells was detected by MTS and flow cytometry in vitro. Hepa1‐6 tumor bearing mice were used to evaluate targeting capability and therapeutic effect of Dox@EVs‐CMC in vivo.

**Results**: Dox@EVs‐CMC had good binding affinity and antiproliferative effects in tumor cells, as indicated by increased uptake of the complex. An in vivo study showed that Dox@EVs‐CMC had excellent tumor targeting ability and were able to significantly slow tumor growth without damaging vital organs.

**Summary/Conclusion**: pH‐responsive CMC provides a safe and effective way to enhance tumor targeting ability of Dox@EVs. This regimen can be viewed as a promising delivery system for the targeted treatment of HCC.

### PS18.07 Screening and identification of differential phospholipids in urinary exosomes of patients with hepatocellular carcinoma

Qiong‐hui zhong, The Second Affiliated Hospital of Nanchang University

Bo Huang, The Second Affiliated Hospital of Nanchang University

mei‐yong li, The Second Affiliated Hospital of Nanchang University

jing liu, The Second Affiliated Hospital of Nanchang University

yan‐mei xu, The Second Affiliated Hospital of Nanchang University

wei‐ming yang, The Second Affiliated Hospital of Nanchang University

hai‐bin zhang, The Second Affiliated Hospital of Nanchang University

jin lin, The Second Affiliated Hospital of Nanchang University

xiao‐zhong wang, The Second Affiliated Hospital of Nanchang University

**Introduction**: Hepatocellular carcinoma(HCC) is the most common clinical pathological subtype of hepatocellular carcinoma, and the current diagnostic tools for HCC are still invasive; therefore, a non‐invasive method for the clinical diagnosis of HCC is urgently needed.

**Methods**: Exosomes were extracted from urine using ultra‐high‐speed centrifugation, laboratory self‐research methods, and exosome extraction kits, and the most clinically appropriate method was selected for exosome extraction. Four groups of specimens (10 cases in each experimental cohort and 20 cases in each validation cohort), including HCC, cirrhosis, chronic hepatitis B and healthy controls, were examined by EESI‐MS to compare the differences in fingerprint profiles; the differential phospholipids were screened by principal component analysis (PCA). The target ions were identified and confirmed using collision‐induced dissociation (CID) test. The area under curve (AUC) and Jorden index were used to determine the diagnostic efficacy of each differential phospholipid component and combination for HCC. The metabolic pathway enrichment of the differential phospholipids was also performed using Metaboanalyst online software.

**Results**: Our experiments revealed that the use of a self‐researched laboratory method could meet the need for exosome sorting concentration and purity and save separation time. The analysis revealed that the hepatocellular carcinoma group was clearly distinguished from the other 3 groups, and the significantly different mass spectral peaks were m/z 726, m/z 754, m/z 757, m/z 787, m/z 803, m/z 832 and m/z 877, which were identified as 2 neurosphingolipids, 4 phosphatidylcholines and 1 phosphatidylglycerol. 7 differential phospholipids were combined to diagnose hepatocellular carcinoma with The area under the ROC curve for the combined diagnosis of hepatocellular carcinoma was more than 98%. The differential phospholipids were involved in five metabolic pathways, namely sphingolipid metabolism, glycerophospholipid metabolism, arachidonic acid metabolism, linoleic acid metabolism and α‐linolenic acid metabolism.

**Summary/Conclusion**: EESI‐MS detection of urinary exosomes phospholipids in patients with hepatocellular carcinoma is reproducible, rapid and efficient; it may provide a new idea for non‐invasive and non‐radiative detection of clinical diseases.

### PS18.08 Selecting the optimal size exclusion chromatography approach for extracellular vesicle isolation from human serum to facilitate quantitative liver‐derived biomarker analysis

Zivile Useckaite, Flinders University

Michael Sorich, Flinders University

Andrew Rowland, Flinders University

**Introduction**: Size exclusion chromatography (SEC) is an efficient, scalable approach to extracellular vesicle (EV) isolation. A number of SEC options are available offering different size isolation ranges, each with trade offs in terms of vesicle enrichment, contamination and vesicle yield. The aim was to define the yield, purity and EV characteristics isolated by two different SEC approaches and to evaluate the impact to downstream applications relevant to liver‐specific biomarker quantification.

**Methods**: Human serum EVs were isolated by SEC (qEV35 and qEV70 columns), quantified and characterized by nanoparticle tracking analysis (NTA), vesicle integrity and morphology was assessed by transmission electron microscopy (TEM), EV markers CD9, CD63, CD81, and liver‐specific markers ASGR1 and CYP3A4 were measured by flow cytometry (FC). CD9, CD63, CD81, TSG101, Calnexin V, albumin, ASGR1 and CYP3A4 abundance was measured by LC‐MS/MS. Additionally, SEC‐isolated EVs were separated by density using ultracentrifugation on density gradient (UC‐DG), and all of the above methodologies were applied to further evaluate high (HD) and low density (LD) EV contribution to biomarker analysis, with particular focus on liver‐specific ASGR1 and CYP3A4. CYP3A4 functionality was further assessed by Hydroxy‐midazolam LC‐MS/MS Assay.

**Results**: There was no difference in particle concentration isolated by qEV35 vs qEV70, with mean particle size smaller in qEV35 isolated sample. TEM confirmed good vesicle integrity for both isolations and smaller EV presence in qEV35 isolates. FC and LS‐MS/MS revealed differences in the profiles of EV cargo between EVs isolated by the two SEC methods, with a higher proportion of known EV markers and tissue‐specific ASGR1, CYP3A4 in EVs isolated by qEV70. Following EV separation by density, significantly higher levels of ASGR1 and CYP3A4 were detected in LD EVs by FC, with higher proportion of LD EVs carrying functional CYP3A4.

**Summary/Conclusion**: Serum derived EVs have potential to be utilized as a biomarker source. However, the robust application of EV derived biomarkers requires a fundamental understanding optimal EV isolation approach. In the case of the liver‐specific EV‐derived biomarkers considered here, qEV70 provided higher biomarker yield and reduced contamination, making it the superior isolation approach for this application.

### PS18.09 Could protein content of Urinary Extracellular Vesicles be useful for detecting Alcoholic Liver Disease?

Esperanza González, CIC bioGUNE

Mikel Azkargorta, CIC bioGUNE

Clara García‐Vallicrosa, CIC bioGUNE

Félix Elortza, CIC bioGUNE

Sonia Blanco‐Sampascual, Hospital Universitario de Basurto.

Juan Manuel Falcón‐Pérez, CIC bioGUNE

**Introduction**: Alcohol abuse has a high impact on the mortality and morbidity related to a great number of diseases and is responsible for the development of alcoholic liver disease (ALD). It remains challenging to detect and evaluate its severity, which is crucial for prognosis. In this work, we studied if urinary EVs (uEVs) could serve in diagnose and evaluate cirrhosis in ALD.

**Methods**: uEVs characterization by cryo‐electron microscopy (Cryo‐EM), Nanoparticle Tracking Analysis (NTA) and Western blotting (WB) was performed in a cohort of 21 controls and 21 cirrhotic patients. Then, to identify new putative biomarkers for cirrhosis in ALD, proteomics of urinary EVs (uEVs) was achieved in a second cohort of 6 controls and 8 patients.

**Results**: uEVs concentration, size and composition was altered in cirrhotic patients. A total of 1304 proteins were identified in uEVs, and 90 of them were found to be altered in cirrhotic patients.

**Summary/Conclusion**: uEVs could be considered as a tool and a supplier of new biomarkers for ALD, whose application would be especially relevant in chronic patients. Yet, further research is necessary to obtain more relevant result in clinical terms.

## PS19 EVs and Cancers Throughout the Body

Chair: Gagan Deep, Wake Forest School of Medicine, United States

Chair: Janusz Rak, Professor, Canada

### PS19.01 Colorectal cancer and peritoneal mesothelial cells: a dangerous communication driven by Extracellular Vesicles

simona Serratì, Laboratory of Nanotechnology, IRCCS Istituto Tumori Giovanni Paolo II, Bari Italy

Roberta Di Fonte, Laboratory of Experimental Pharmacology, IRCCS Istituto Tumori Giovanni Paolo II, Bari Italy

Michele Simone, Department of Surgery Oncology, IRCCS Istituto Tumori Giovanni Paolo II, Bari Italy

Francesco Fragrassi, Department of Surgery Oncology, IRCCS Istituto Tumori Giovanni Paolo II, Bari Italy

Marianna Garofoli, Laboratory of Experimental Pharmacology, IRCCS Istituto Tumori Giovanni Paolo II, Bari Italy

Rosa Maria Iacobazzi, Laboratory of Experimental Pharmacology, IRCCS Istituto Tumori Giovanni Paolo II, Bari Italy

Livia Fucci, Pathology Department, IRCCS Istituto Tumori Giovanni Paolo II, Bari Italy

Amalia Azzariti, Laboratory of Experimental Pharmacology, IRCCS Istituto Tumori Giovanni Paolo II, Bari Italy

Letizia Porcelli, Laboratory of Experimental Pharmacology, IRCCS Istituto Tumori Giovanni Paolo II, Bari Italy

**Introduction**: Emerging evidences suggest a crucial role of Extracellular Vescicles (EV) released by cells in the microenvironment in mediating directional tumor metastasis to peritoneal during the colorectal cancer (CRC) progression. Herein, we investigated the EV‐mediated crosstalk between tumor and peritoneal mesothelial cells (PMC) which may drive the remodelling of premetastatic niche allowing tumor invasion of the peritoneal surface.

**Methods**: During surgery, peritoneal lavage liquids (PLFs) were collected from twelve colon cancer patients. PMC and EV were isolated. EV were isolated by ultracentrifugation and characterized by NTA and flow cytometry. Apoptotic potential of EV was evaluated by AnnexinV/PI staining and tumor/mesothelial cells invasion by Boyden chamber Matrigel assay. The mesothelial‐to‐mesenchymal transition (MMT) was assessed by immunofluorescence and gelatin zymography assay. Invasion biomarkers modulation was evaluated by RT‐PCR and western blotting.

**Results**: Firstly, colon and mesothelial‐derived EV, expressing CK‐20 or calretinin, were assessed in PLF. Next, we demonstrated the uptake of exosomes by both tumor and PMC. Upon uptake, cancer‐derived EV triggered apoptosis and the reduction of invasion abilities in mesothelial cells. Additionally, such EV promoted the MMT, by increasing the α‐SMA expression in PMC. Nevertheless, mesothelial cells were not simple bystander, instead they actively supported tumor invasion by releasing EV expressing CD44 and high amount of MMP‐9. Indeed, these vesicles induced the upregulation of the major pro‐invasive system u‐PAR/u‐PA in tumor cells, supporting tumor invasion.

**Summary/Conclusion**: For the first time, our findings elucidate a previously unknown mechanism of CRC dissemination. We provide evidence of EV‐driven mechanisms of CRC progression in patients‐derived models, highlighting the crucial role of EV‐driven reprogramming of PMC and tumor cells in the establishment of peritoneal carcinosis.

### PS19.02 Exosome‐mediated secretion of CST1

promotes colorectal cancer cell

invasion and metastasis

weili duan, shandong university

Chuanxin Wang, Second Hospital, Shandong University

lutao du, shandong university

Chuanxin Wang, Second Hospital, Shandong University

**Introduction**: Cystatin SN (CST1), encoded by CST1, is a secretory peptide and belongs to the type 2 cystatin superfamily. Emerging evidence has demonstrated that CST1 has diverse critical biological functions, especially its roles in tumor development and metastasis.

**Methods**: Conditioned medium (CM) preparation and exosome isolation,Nanoparticle tracking analysis (NTA), Transmission electron microscopy (TEM),were used to validate the exosomes. Exosome labeling and analysis was used for exosome tracing,Tube formation assay, Transwell migration and invasion assays were used to investage the effect of exosome on human umbilical vein endothelial cells (HUVECs) and CRC cells.

**Results**: In the present study, CST1 was commonly upregulated in CRC tissues and predicted a poor prognosis. Upregulation of CST1 promoted, whereas downregulation of CST1 inhibited cell migration, invasion and EMT of CRC in vitro. Most interestingly, we found that CRC‐derived exosomes transferred CST1 between HCC cells promoted cell migration by activating the MAPK pathway. In addition, CRC‐derived exosomes transferred CST1 to human umbilical vein endothelial cells (HUVECs) to promote angiogenesis.

**Summary/Conclusion**: Taken together, our results demonstrate a novel function of CST1 in tumor metastasis mediated by exosomes through regulation of the MAPK pathway and angiogenesis in CRC.

### PS19.03 Exosomes engineered by miRNA retard the malignant progression of gastric cancer

Peipei Wu, 212000, Jiangsu university

Linli Li, Jiangsu University

Hui Qian, jiangsu university

Wenrong Xu, Jiangsu University

**Introduction**: Gastric cancer (GC) is the fourth most common cancer and the second leading cause of cancer‐related death worldwide. Despite rapid advances in treatment in recent decades, clinical outcomes are still poor. Therefore, it is an urgent to find a new alternative strategy to effectively intervene the malignant progression of GC.

**Methods**: Through miRNA sequencing analysis, we found that miRNA molecules highly enriched in human umbilical cord mesenchymal stem cells derived exosomes (hucMSC‐Ex) can delay the malignant progression of GC cells through a variety of different molecular mechanisms. Western blotting, CCK8, clone formation and migration experiments were used to confirm the effects of miRNA and miRNA mimics or inhibitor overexpressed‐hucMSC‐Ex on the proliferation and migration of GC cells.

**Results**: hucMSC‐Ex are efficiently taken up by GC cells, and miRNA carried in hucMSC‐Ex can restrain the growth and metastasis of GC cells by inhibiting autophagy levels of GC cells. In addition, the preparation of miRNA‐loaded engineered hucMSC‐Ex by electroporation exerts significant anti‐tumor effects in vitro.

**Summary/Conclusion**: Exosomes of approximately 30–200 nm in diameters are the promising nano‐carrier for therapeutic cargos. Our study shows that miRNAs engineered hucMSC‐Ex are promising as an emerging approach for targeted therapy of GC.

### PS19.04 Exosomes from cells deprived of Insulin‐like Growth Factor 2 mRNA Binding Protein 3 dampen Ewing Sarcoma invasive phenotype

Giulia Caldoni, Laboratory of Experimental Oncology, IRCCS Istituto Ortopedico Rizzoli, Bologna, Italy

Veronica Giusti, Laboratory of Experimental Oncology, IRCCS Istituto Ortopedico Rizzoli, Bologna, Italy

Maria Antonella Laginestra, Laboratory of Experimental Oncology, IRCCS Istituto Ortopedico Rizzoli, Bologna, Italy

Lisa Toracchio, Laboratory of Experimental Oncology, IRCCS Istituto Ortopedico Rizzoli, Bologna, Italy

Alessandro Parra, Laboratory of Experimental Oncology, IRCCS Istituto Ortopedico Rizzoli, Bologna, Italy

Alessandra De Feo, Laboratory of Experimental Oncology, IRCCS Istituto Ortopedico Rizzoli, Bologna, Italy

Katia Scotlandi, Laboratory of Experimental Oncology, IRCCS Istituto Ortopedico Rizzoli, Bologna, Italy

**Introduction**: Insulin‐like Growth Factor 2 mRNA Binding Protein 3 (IMP3) has a key oncogenic role in Ewing Sarcoma (EWS), an aggressive pediatric bone tumor. Our previous evidence showed how low IMP3 levels reduce EWS cell migration and impact patient survival. As horizontal transfer of key molecules via exosomes (EXOs) mediates cell‐cell communication, we aim to demonstrate that EXOs from IMP3neg less‐invasive EWS cells exert an oncosoppressive role reducing the migratory abilities of IMP3pos receiving cells.

**Methods**: EXOs were isolated from stable IMP3 knockdown EWS cellular models (A673 and TC71) and their cargo was evaluated by RT‐qPCR and Western Blotting (WB). We performed miRNA profiling using QIAseq miRNA kit and sequenced libraries on NextSeq500 platform. MirWALK was used for miRNA‐target prediction followed by GO enrichment analysis. We evaluated differential migration properties and modulation of IMP3 targets upon IMP3pos cells exposure to IMP3neg EXOs by transwell assay and by WB.

**Results**: EXOs mirror IMP3 levels from donor IMP3pos or IMP3neg cells. Given the known influence of IMP3 on migration and hypothesizing an oncosoppressive role for IMP3neg EXOs, we performed miRNA profiling on IMP3neg and IMP3pos EXOs. We identified 62 differentially expressed miRNAs, among them miR‐196a, let‐7e and miR‐146b. GO enrichment analysis performed on their predicted targets revealed involvement in positive regulation of cell migration (GO:0030335). Hence, we exposed IMP3pos cells to IMP3neg EXOs and observed impaired migration in presence of IGF1 and CXCL12 gradient. Trying to unveil the underlying mechanism, we found that IMP3neg EXOs carry lower levels of IMP3 targets IGF1R, CXCR4 and CD164. Exposure of IMP3pos cells to IMP3neg EXOs resulted in modulation of IGF1R, CXCR4 and CD164 protein levels.

**Summary/Conclusion**: IMP3neg EXOs carry a specific cargo that dampens the migration of EWS cells, thus conveying the same less‐invasive phenotype observed in IMP3neg cells.

### PS19.05 Extracellular Vesicle‐mediated Lenalidomide Resistance in Multiple Myeloma Cells

Tomofumi Yamamoto, Department of Molecular and Cellular Medicine, Tokyo Medical University

Jun Nakayama, Division of Cellular Signaling, National Cancer Center Research Institute

Yusuke Yamamoto, Division of Cellular Signaling, National Cancer Center Research Institute

Yutaka Hattori, Clinical Physiology and Therapeutics, Keio University Faculty of Pharmacy

Takahiro Ochiya, PhD, Department of Molecular and Cellular Medicine, Tokyo Medical University

**Introduction**: Multiple myeloma (MM) is a hematological tumor. Almost all of the MM therapy contained immunomodulatory drugs (IMiDs); however, long‐term exposure of these drugs caused multi‐drug resistance. Interaction between MM cells and mesenchymal stromal cells (MSCs) in the bone marrow is considered to be critical for drug resistance. Although extracellular vesicles (EVs) are key players for intercellular communications, the mechanism of drug resistance via EVs has not been elucidated.

**Methods**: In order to understand the mechanism of drug resistance in MM, lenalidomide resistant cell lines were established. To identify the genes which involved in drug resistance, RNA sequence among the drug‐resistant cell lines and their parental cell lines was performed. Functional analysis of identified genes was performed using gene silencing.

**Results**: Established lenalidomide resistant cell lines produced much more EVs compared with parental cell lines and enhanced cell adhesion ability on MSCs. Coculture analysis of resistant and parental cells revealed that lenalidomide resistant cell‐derived EVs influenced drug susceptibility in parental cells. In addition, after cocultured with lenalidomide resistant cell lines, parental cell lines phenotypically changed from non‐adherent to adherent. RNA sequencing revealed that the pathways associated with EV biosynthesis were enriched in the lenalidomide resistant cell lines by gene set enrichment analysis. Among the highly expressed genes in lenalidomide resistant cell lines, we found SORT1 and LAMP2 genes, which increased EVs production and cell adhesion ability. Interestingly, the silencing of SORT1 or LAMP2 decreased EV secretion and recovered lenalidomide sensitivity similar to that of parental cells.

**Summary/Conclusion**: Our data showed that EV secretion via SORT1 or LAMP2 could induce cell adhesion, leading to the acquisition of lenalidomide resistance in MM.

### PS19.06 Extracellular Vesicles Derived from Cancer Demonstrate Autophagosome Properties

Daivik Siddhi, Oakton High School

**Introduction**: Exosomes, or extracellular vesicles (EVs), are membrane‐bound vesicles released under both normal and cancerous conditions by cells. EVs released have a diversity of antigenic characteristics that scientists aim to further characterize and study. In particular, EV release is pronounced during cancer growth and invasion. Classifying profiles of EVs released into the lymphatic drainage may provide a direct biomarker predictive of the tumor state, response to therapy, and other microenvironmental conditions linked to disease progression, such as hypoxia. I aim to monitor the breast cancer‐derived EVs of different size classes under normal growth conditions and under an autophagy inhibited environment. Furthermore, I aim to provide a deeper understanding of autophagy within the tumor microenvironment, to identify novel diagnostic markers to inform new therapeutic strategies.

**Methods**: In order to do this, GFP‐4T1 cells, a mouse breast cancer cell line, were cultured in a 6 well cell plate with three containing regular media for growth and the other three containing a 50 millimolar concentration of chloroquine, a known autophagy inhibitor. These medias were pulled off and the exosomes were centrifuged into 2K, 10K, and 100K size classes at 6, 24, and 48 hr time intervals and analyzed for various autophagy and exosome markers using western and immunoblot methods.

**Results**: The double membrane is a hallmark example of an autophagy‐derived autophagosome. The data for the cell experiment is quantified in bar graphs and the concentration in LC3B, an autophagy marker, has gone down as well as the amount of p62, another autophagy marker, which establishes a direct relationship between the decrease in autophagy and decrease in exosomes. To prove this relationship I conducted a quantitative analysis in the form of T‐tests which showed that the difference in amounts of LC3B and p62 are statistically significant and we can accept our hypothesis. The final piece of data are images are of a tube formation assay that further proves that the addition of the larger 2K exosomes is more malignant due to the greater prevalence of greater vascular sprouting over 6 days.

**Summary/Conclusion**: In conclusion the data proved my hypothesis and I found that EVs are released by cancer cells to potentially aid in their malignant progression. Additionally, these exosomes contain markers of autophagy therefore the inner processes and material within the cell can be monitored as new biomarker sources. Some further steps include characterizing these EVs over longer periods of time and analyzing EVs from human cancer cell lines. By establishing the simple yet novel connection between the inhibition of autophagy and decreased exosome release this research has the potential to change the way the medical community approaches breast cancer detection and treatment and takes a solid step towards the fight for the cure.

### PS19.07 Extracellular vesicles secreted by bone tropic renal cell carcinoma induce angiogenesis in bone marrow with potential to facilitate bone metastasis

Masashi Takeda, Kyoto University

Shusuke Akamatsu, Kyoto University

Toshinari Yamasaki, Kyoto University

Tomohiro Fukui, Kyoto University

Takao Haitani, Kyoto University

Hiromasa Sakamoto, Kyoto University

Takayuki Goto, Kyoto University

Atsuro Sawada, Kyoto University

Koji Ueda, Japanese Foundation for Cancer Research

Takashi Kobayashi,Kyoto University

Osamu Ogawa, Kyoto University

**Introduction**: Bone metastasis of renal cell carcinoma (RCC) is associated with poor prognosis. However, the mechanisms underlying RCC bone metastasis remain unclear. Recently, extracellular vesicle (EV) has been shown to play crucial roles in premetastatic niche formation through mediating interactions between cancer cells and stromal cells. The objective of this study is. We aimed to explore the function of EVs secreted by bone tropic RCC cells in premetastatic niche formation.

**Methods**: First, we established a clear cell RCC cell line with enhanced bone tropism (786‐O BT) by in vivo selection of clear cell RCC cell line 786‐O and collected EVs from conditioned medium using ultracentrifugation. Then, to investigate the function of 786‐O derived EVs, we administered either parent 786‐O derived EV (P‐EV) or 786‐O BT derived EV (B‐EV) to nude mice for 2 weeks, collected hind limbs and compared the histological findings. We also performed tube formation assay for in vitro functional analysis of 786‐O derived EVs.

Next, we performed proteomic analysis to identify the protein enriched in B‐EV that could cause histological changes in bone. After selecting a target protein, we established a knockdown cell, and then, performed tube formation assay.

Finally, we injected luciferase expressing 786‐O cells intracardially to EV treated mice and monitored bone metastasis formation using In Vivo Imaging System.

**Results**: Histologic examination revealed B‐EV lead to increased blood vessel formation in bone marrow. (Figure.1a)

Treatment with B‐EV also showed increased tube formation in vitro.

Proteomic analysis data identified 157 proteins enriched in B‐EV compared to P‐EV. Among them, we focused on aminopeptidase N(APN) as a target protein that could be associated with angiogenesis. EVs secreted by APN knockdown cells showed reduced angiogenesis in tube formation assay compared to control EVs.

B‐EV treated mice showed bone metastases more frequently than P‐EV treated mice in an intracardiac injection model. (Figure.1b)

**Summary/Conclusion**: EVs secreted by bone tropic RCC cells induced angiogenesis in bone marrow through APN potentially facilitating bone metastasis.

### PS19.08 IgM as a diagnostic tool: traveling ganglioside enriched large oncosomes are enough to predict cancer prognosis

Madhusudhan Bobbili, Ludwig Boltzmann Institute for Experimental and Clinical Traumatology

Johanna Gamauf, Institute of Molecular Biotechnology, Department of Biotechnology, University of Natural resources and Life Sciences Vienna, Vienna, Austria

Markus Schosserer, Institute of Molecular Biotechnology, Department of Biotechnology, University of Natural resources and Life Sciences Vienna, Vienna, Austria

Hermann Katinger, Polymun Scientific Immunobiologische Forschung GmbH, Klosterneuburg, Austria

Johannes Grillari, Ludwig Boltzmann Institute for Experimental and Clinical Traumatology

**Introduction**: Gangliosides are strongly enriched in human tumors such as melanomas, lung and prostate cancers. Many studies have shown that gangliosides promote cancer development and progression [1]. Recent evidence show that gangliosides are enriched in tumor‐cell derived extracellular vesicles (EVs) or large oncosomes (LO) promoting cancer cell migration [2].

**Methods**: In our study, to determine the cancer prognosis we employed inhouse produced IgM monoclonal antibody specific to class of gangliosides such as GM3, GM2 and GD3 for detecting large oncosomes and small EVs (sEV). LO and sEV derived from different cancer cell types were isolated using differential ultracentifugation. The evaluation of IgM detecting ganglioside enrichment on LO was carried out by fluorescent triggered flow cytometry. Additionally, we performed bead‐capture assay for detection of gangliosides by IgM on sEV.

**Results**: Our preliminary results demonstrate the enrichment of gangliosides on LO derived from melanomas, prostate and colorectal cancer but not on sEV. Additionally, enrichment of gangliosides on large EVs/microvesicles (lEV or MV) ‐derived from different human primary cell types is relatively low to undetectable compared to tumor‐derived LO. Furthermore, LO/MVs and sEV are characterized for surface markers such as CD9, CD63, CD81 and CD147 by multiplex labelling using flow cytometry and for EV specific markers by immunoblotting. Nanoparticle tracking analysis (NTA) was performed for size and concentration of particles and BCA assay for EV protein content.

**Summary/Conclusion**: Our preliminary findings suggest that large oncosomes derived from different cancer cell types are enriched in gangliosides, and that our inhouse IgM provides a diagnostic platform for detecting circulating large oncosomes in melanomas, colorectal and prostate cancers. However, further studies with patient tumor samples are required to validate the potential use of IgM as a diagnostic tool for cancer prognosis.

### PS19.09 Loss of hepatocarcinoma cells‐derived exosomal micoRNA‐622 promotes lipolysis of adipocytes

Xuan Deng, Department of Laboratory Medicine, Huashan Hospital, Shanghai Medical College, Fudan University

Ming Guan, Department of Laboratory Medicine, Huashan Hospital, Shanghai Medical College, Fudan University

**Introduction**: Cancer‐associated cachexia (CAC), characterized by body weight loss, usually accompanying the end stage of cancer, not only increases patient mortality but also reduces the efficacy of treatment. This study was aimed to find microRNAs that are related to disease‐associated malnutrition reflected by body composition in advanced stage HCC patients.

**Methods**: Plasma microRNAs of stage III‐IV HCC patients HCC were sequenced. Body composition measurements were conducted on CT images of HCC patients prior to tumor resection or therapies. Correlation between plasma miR‐622 levels and SFMI (subcutaneous fat mass index) or VFMI (visceral fat mass index) was analyzed by SPSS. Primary human hepatocytes (PHH), commercial HCC cell line (HepG2, Hep3B, PLC and huh7), human mature adipocytes (AD) and mouse 3T3‐L1 adipocytes were used as model systems. Exosome from cells were isolated using differential centrifugation coupled with ultracentrifugation. The isolated exosomes were quantified based on nanoparticle tracking analysis (NTA) and total protein amount. Glycerol and free fatty acids (FFA) levels in media were measured using colorimetric assay kits.

**Results**: We found plasma miR‐622 levels to be positively correlated with fat mass indexes in HCC patients. MiR‐622 levels were drastically downregulated in exosomes from HCC cell lines, whereas upregulated by miR‐622 overexpression. Plasma exosomal miR‐622 were also significantly decreased in HCC patients. As compared to those incubated with PHH‐derived exosomes, adipocytes cultivated with HepG2‐exosomes showed decreased miR‐622 expression and released more FFAs and glycerol. These effects were reversed by exosomes from miR‐622 overexpressing HepG2. The pro‐lipolytic role by loss of exosomal miR‐622 were further confirmed by plasma exosomes. HCC patient‐derived exosomes downregulated miR‐622 and activated lipolysis in adipocytes compared with exosomes from non‐HCC control subjects. Collectively, these results support that exosomal miR‐622 from HCC could be incorporated by adipocytes and at least partially inhibit lipolytic process of adipocytes.

**Summary/Conclusion**: Our study explored the role of HCC‐derived exosomal miR‐622 in attenuating lipolysis using in vitro models. Recent findings suggest cancer cells/tissues can utilize lipolysis to acquire fatty acids that, in turn, providing energy for their proliferation and survival. Thus, promoting the transfer of exosomal miR‐622 might be a potential treatment option to alleviate cancer‐related lipolysis and suppress HCC progression.

### PS19.11 Oxygen tension regulates the secretion and bioactivity of small extracellular vesicles from a heterogenic panel of ovarian cancer cells which results in metabolic switch with an enrichment of glycolysis‐pathway proteins

Nihar Godbole, University of Queensland Centre for Clinical Research

Andrew Lai, PhD, Exosome Biology Laboratory, Centre for Clinical Diagnostics, UQ Centre for Clinical Research, The University of Queensland, Australia

Shayna Sharma, University of Queensland Centre for Clinical Research

America Campos, Exosome Biology Laboratory, Centre for Clinical Diagnostics, UQ Centre for Clinical Research, The University of Queensland, Australia

Priyakshi Kalita‐de Croft, University of Queensland Centre for Clinical Research

Carlos Salomon, MSc, DMedSc, PhD, The University of Queensland

**Introduction**: Ovarian cancer (OVCA) is a broad term, encompassing a heterogenous population of tumours, with both ovarian, and related origins. Recent studies highlight a clear link between hypoxia and changes in the function of small extracellular vesicles (sEVs) in a wide range of cancers. However, progress in the OVCA field is hindered by a lack of studies addressing the heterogeneity of the disease. Even across subtypes, there is significant heterogeneity within cells, which is a major reason for treatment failure. Here, we evaluate the effect of hypoxia on the secretion and bioactivity of sEVs from ovarian cancer cell lines, and their association with glycolysis‐pathway proteins.

**Methods**: A panel of ovarian cancer cell lines were used in this study: low‐grade serous (HEY), high‐grade serous (SKOV‐3, OV90, and CAOV‐3), clear cell (TOV‐II2D) and endometrioid (OVTOKO). Cells were cultured at 370C (5% CO2‐balanced N2 to obtain 1% or 8% O2). Cell‐conditioned media (CCM) was collected and different populations of EVs were isolated by differential centrifugation, and characterized using nanoparticle tracking analysis (NanoSight NS500). To better understand the glycolytic changes associated with hypoxia, we designed a liquid chromatography multiple reaction monitoring (LC‐MRM) assay targeting the glycolysis pathway proteins in sEVs and OVCA cells.

**Results**: Hypoxia induced the secretion of sEVs from all OVCA cell lines studied. Interestingly, high‐grade serous cells increased the secretion of sEVs by 3‐fold, 2,5‐fold and 19‐fold (p < 0.001), in SKOV‐3, OV90, and CAOV‐3, respectively. In low‐grade serous (HEY), clear cell (TOV‐II2D), and endometrioid (OVTOKO), hypoxia increased the secretion of sEVs by 3‐fold, 6‐fold and 4‐fold (p < 0.001), respectively. Interestingly, a higher concentration of sEVs under hypoxia, and normoxia, was observed from OV90 cells, and the lower concentration was identified in CAOV‐3 cells, under 8% oxygen, and in HEY cells under 1% oxygen. Using targeted proteomics, we identified that glycolysis‐associated proteins and detoxification enzymes were enriched in sEVs from 1% when compared with 8% oxygen, and this effect was specific to origin of the OVCA cell lines. Moreover, a specific set of proteins was selectively enriched within the sEVs when compared to their cells of origin at 8% and 1% O2, indicating the specific packaging of proteins into sEVs.

**Summary/Conclusion**: We identified a specific pattern of secretion of sEVs from OVCA cells in response to hypoxia. Changes in the glycolysis‐pathway proteins within sEVs in response to hypoxia suggest a potential role in sEVs in OVCA metabolism.

### PS19.12 Small extracellular vesicles represent different pattern of proteins and metabolites in response to radiotherapy of colorectal cancer patients

Urszula Strybel, Institute of Bioorganic Chemistry Polish Academy of Sciences

Lukasz Marczak, Institute of Bioorganic Chemistry Polish Academy of Sciences

Marcin Zeman, Maria Sklodowska‐Curie National Research Institute of Oncology, Gliwice Branch

Krzysztof Polanski, Wellcome Sanger Institute, Wellcome Genome Campus

Łukasz Mielańczyk, Medical University of Silesia

Monika Pietrowska, PhD, Maria Sklodowska‐Curie National Research Institute of Oncology

Anna Wojakowska, Institute of Bioorganic Chemistry Polish Academy of Sciences

**Introduction**: The major benefit of preoperative radiotherapy (RT) in colorectal cancer (CRC) is downsizing the tumor to reduce radicalness of surgery, or in some cases, to complete omission of the surgery. Serum/plasma proteomics and metabolomics of CRC patients could provide valuable insight into the response to RT. Though small extracellular vesicles (sEVs) are an emerging type of liquid biopsy, metabolomic and proteomic changes in sEVs of cancer patients after RT have not been given as much attention. This study aimed to describe the correlation between specific molecular components of serum/plasma as well as sEVs and CRC patient's response to RT.

**Methods**: Plasma and serum samples were collected from 40 CRC patients treated with RT. Samples were classified into two groups, depending on the response to the treatment: group A " patients with good response (sensitive) to RT and group B " patients with a poor response (resistant) to RT. Small EVs were isolated from serum/plasma using size‐exclusion chromatography (SEC). LC‐MS/MS and GC/MS‐based approaches were used for proteomic and metabolomic profiling.

**Results**: LC‐MS/MS‐based approach allowed the identification of 225 and 332 proteins, of which 29 and 71 differentiating sensitive and resistant serum and serum‐derived sEVs samples, respectively. Serum differentiating proteins were associated mainly with transport and metabolism of lipids. Serum‐derived sEVs distinguished proteins were connected with immune system and inflammatory response. An untargeted GC"MS‐based approach allowed the identification of 116 and 53 metabolites in plasma and plasma‐derived sEVs, respectively, of which 32 metabolites overlapped. Metabolites that differentiated sensitive and resistant plasma samples were associated with lipid and amino acid metabolism. Metabolites differentiating plasma‐derived sEV were associated with energy metabolism. Integration of metabolomic and proteomic data showed common overrepresented pathways connected with response to RT of CRC, including complement and coagulation cascade, ECM‐receptor interaction, fatty acid biosynthesis, and cholesterol metabolism.

**Summary/Conclusion**: This study revealed a specific pattern of proteins and metabolites in serum/plasma and sEVs, which could distinguish CRC patients with different response to preoperative radiotherapy. Moreover, response to RT‐related changes observed in the molecular pattern of sEVs was more significant than response‐related changes detected in serum/plasma molecules. Our results confirmed the feasibility of cancer predictive biomarkers based on exosome proteins and metabolites. Besides, integration of metabolomic and proteomic data reveals novel insights into the analysis of global response to cancer treatment.

### PS19.13 The TGF‐β/smads signaling pathway and epithelial‐mesenchymal transition in invasive pituitary tumors by serum exosomes

Guoge li, Capital Medical University

Guojun Zhang, Capital Medical University

**Introduction**: Assessing the invasiveness of pituitary adenomas (PAs) is critical to making the best surgical and treatment plan. However, it is difficult to reflect the invasiveness of pituitary adenomas based on the current clinical method, such as the imaging or the histological methods. The present article is aimed at using non‐invasive methods to find viable biomarkers for invasive pituitary adenomas and providing basis for early intervention of pituitary adenomas.

**Methods**: E‐Cadherin, N‐Cadherin, Epcam, TGF‐β, smad3, smad7 were detected in the tissues and exosomes of 10 cases of invasive PAs and 10 cases of noninvasive PAs by real‐time quantitative reverse transcription polymerase chain reaction (qRT‐PCR), Western blot, and immunohistochemical analysis.

**Results**: In exosomes, compared with the non‐invasive group, the expression of N‐cad in the invasive group was significantly increased and the expressions of E‐cad and Epcam were reduced. In the invasive group, the expression levels of TGF‐β1 and smad3 were reduced. These results are consistent across exosomes and organizations. In further cell experiments, the EMT ratio in the SIS3 treatment group, especially in the TGF‐β1 and SIS3 treatment groups (P < 0.001) was significantly increased, and the EMT ratio was significantly lower when half the dose of TGF‐β and SIS3 was given.

**Summary/Conclusion**: The results indicate that EMT related biomaker in serum exosome can be used as a potential biomarker for assessing th invasiveness of pituitary adenoma.

### PS19.14 Tumor‐derived microRNA‐378a‐3p‐containing extracellular vesicles induce osteoclastogenesis by activating Dyrk1a/Nfatc1/Angptl2 axis to promote the metastasis of prostate cancer

Jialing Wang, State Key Laboratory of Oncogenes and Related Genes, Renji‐Med X Clinical Stem Cell Research Center, Ren Ji Hospital, School of Medicine, Shanghai Jiao Tong University

Yanqing Wang, Department of Urology, Renji Hospital, School of Medicine, Shanghai Jiao Tong University

Baijun Dong, Department of Urology, Renji Hospital, School of Medicine, Shanghai Jiao Tong University

Wei Xue, Department of Urology, Renji Hospital, School of Medicine, Shanghai Jiao Tong University

Wei‐Qiang Gao, State Key Laboratory of Oncogenes and Related Genes, Renji‐Med X Clinical Stem Cell Research Center, Ren Ji Hospital, School of Medicine, Shanghai Jiao Tong University

Yu‐Xiang Fang, State Key Laboratory of Oncogenes and Related Genes, Renji‐Med X Clinical Stem Cell Research Center, Ren Ji Hospital, School of Medicine, Shanghai Jiao Tong University

**Introduction**: The majority of the deaths of prostate cancer (PCa) are caused by progression to bone metastatic PCa. The importance of extracellular vesicles (EVs) in the formation of the pre‐metastatic niche has been demonstrated in recent years. However, whether and how tumor‐derived EVs interact with pre‐osteoclasts to release EV‐delivered microRNAs to activate pre‐metastatic niche formation and prostate cancer bone metastasis remain unclear.

**Methods**: Bioinformatics and qRT‐PCR analyses were used to screen and identify the expression of miR‐378a‐3p in both serum and tissue derived EVs from primary or metastatic PCa patients. Biological function assay studies in vitro and in vivo were implemented to identify the functions of miR‐378a‐3p during PCa progression. Dual‐luciferase reporter assay, co‐IP assay, western blot assay, IF staining, RIP and ChIP assays were conducted to investigate the underlying mechanism.

**Results**: We found that EV‐mediated release of miR‐378a‐3p from tumor cells is upregulated in bone‐metastatic PCa for maintenance of a low intercellular concentration of miR‐378a‐3p to promote proliferation and anti‐apoptosis of PCa cells and a MAOA‐mediated epithelial‐to‐mesenchymal transition (EMT) for migration as well. Furthermore, we demonstrated that the enrichment of miR‐378a‐3p in tumor delivered EVs was induced by overexpression of hnRNPA2B1, a RNA binding protein, as a transfer chaperone. After miR‐378a‐3p enriched EVs was absorbed by pre‐osteoclasts, elevated intercellular concentration of miR‐378a‐3p in pre‐osteoclasts promotes osteoclastogenesis by targeting Dyrk1a/Nfatc1 signaling pathway. Moreover, inhibition of Dyrk1a by miR‐378a‐3p attenuated its sequestration to Nfatc1 so that the nuclear translocation of Nfatc1 was increased to promote expression of the downstream target gene Angptl2. As a feedback, increased secret of Angptl2 into the environment was found to be capable to improve PCa progression.

**Summary/Conclusion**: Our findings indicate that tumor‐derived miR‐378a‐3p‐containing EVs plays a significant role in promoting prostate cancer bone metastasis by activating Dyrk1a/Nfatc1/Angptl2 axis in pre‐osteoclasts to induce osteoclastogenesis, which implicates that miR‐378a‐3p may be a potential predictor of metastatic PCa. Moreover, reducing the release of miR‐378a‐3p‐containing EVs or blocking the function of miR‐378a‐3p in pre‐osteoclasts might be a potential therapeutic strategy for PCa metastasis.

## PS20 EVs and Our Non‐Human Friends

Chair: Kwang Pyo Kim, Department of Applied Chemistry, Institute of Natural Science, Global Center for Pharmaceutical Ingredient Materials, Kyung Hee University, Yongin, South Korea; Department of Biomedical Science and Technology, Kyung Hee Medical Science Research Institute, Kyung Hee University, Seoul, South Korea

Chair: Zheng Lei, Department of Laboratory Medicine, Nanfang Hospital, Southern Medical University, China (People's Republic)

### PS20.01 Comparative proteome profiling in exosomes derived from porcine colostrum versus mature milk

Rafaela Furioso Ferreira, Institute of Animal Science, Physiology Unit, University of Bonn

Thomas Blees, Institute of Animal Science & Physiology Unit, University of Bonn

Farhad Shakeri, Institute for Medical Biometry, Informatics and Epidemiology, Medical Faculty, University of Bonn

Andreas Buness, Institute for Medical Biometry, Informatics and Epidemiology, Medical Faculty, University of Bonn

Marc Sylvester, Institute of Biochemistry and Molecular Biology, Core Facility Mass Spectrometry, Medical Faculty, University of Bonn

Alessandro Agazzi, Department of Health, Animal Science and Food Safety ‘Carlo Cantoni’ (VESPA), University of Milan

Giovanni Savoini, Department of Health, Animal Science and Food Safety ‘Carlo Cantoni’ (VESPA), University of Milan

Vladimir Mrljak, Faculty of Veterinary Medicine, University of Zagreb

Helga Sauerwein, Institute of Animal Science & Physiology Unit, University of Bonn

**Introduction**: Colostrum and milk have high nutritional value and provide a complete diet for neonates, along with bioactive substances which modulate various functions such as immune defense. The mechanisms by which milk components can prime the infant's active immunity are not entirely clear, and EVs are suggested to be essential for the infant's physiological development.

**Methods**: We assessed the exosomal proteome profile from milk samples obtained from 10 healthy sows, at day 0 (colostrum), day 7, and 14 post partum. Exosomes were isolated by ultracentrifugation coupled with size exclusion chromatography, and were characterized by nanoparticle tracking analysis, transmission electron microscopy and Western blotting for exosome markers. Isolated exosomes were in‐gel digested and after TMT‐labelling of the peptides, they were subjected to LC‐MS/MS. The statistical analyses were performed in R using an in‐house developed workflow. Non‐unique peptides, single‐hit proteins, and fractions with low intensity per protein were filtered out. The data were transformed and normalized using the VSN package and then aggregated to protein‐level by Tukey's median polish procedure. The P‐values were adjusted for multiple testing by Benjamini‐Hochberg method.

**Results**: After exclusion criteria were applied, a total of 319 proteins in each timepoint were statistically analyzed. Exosomes from colostrum presented 162 differentially abundant proteins (DAP) (82 increased and 80 decreased) as compared to exosomes from milk at day 7, and 170 DAP (81 increased and 89 decreased) from milk at day 14, respectively. Comparison between milk exosomes at day 7 and day 14 showed no DAP. The DAP identified were related to biological functions such as uptake of metabolites, regulation of hemostasis and cellular development.

**Summary/Conclusion**: Colostral exosomes have different proteome profiles than exosomes from mature milk, with significance not only for the physiological understanding of EV's impact on the interaction between mother and newborn, but also for the prospective use of supplements with high economic potential such as milk and colostrum replacers.

### PS20.02 Comparison of extracellular vesicles and proteins as a source of small RNA biomarkers in canine urine

Jenni Karttunen, PhD, Department of Veterinary Medicine, University of Cambridge

Lajos Kalmar, PhD, Department of Veterinary Medicine, University of Cambridge, UK

Sarah E. Stewart, Department of Biochemistry and Genetics, La Trobe Institute for Molecular Science, La Trobe University, Melbourne, VIC 3083, Australia

Andrew Grant, PhD, Department of Veterinary Medicine, University of Cambridge, UK

Fiona Karet Frankl, PhD, FRCP, FMedSci, Department of Medical Genetics, University of Cambridge, UK

Tim Williams, PhD, Department of Veterinary Medicine, University of Cambridge, UK

**Introduction**: Urinary extracellular vesicles (EVs) and their RNA cargo are a novel source of biomarkers, however non‐vesicular RNA is also present within urine. Here, we compared the small RNA profiles of the EV and protein fractions of canine urine, to determine their potential as a source of small RNA biomarkers.

**Methods**: EV and protein fractions were obtained by size‐exclusion chromatography (SEC) of 0.22μm filtered urine from five healthy control (HC) dogs and five dogs with urinary tract infections (UTI). RNA from both fractions was analysed using the Agilent Bioanalyzer small RNA chip and small RNA sequencing. The raw sequences were compared to examine the fraction‐specific profiles. EV and protein fractions from HC and UTI samples were compared to identify differentially expressed sequences.

**Results**: In HC samples, the protein fractions included more small RNA compared to EV fractions (30.4‐307.2 vs 2.6‐20.3 ng/ml of starting urine, respectively). After sequencing, 11000–20000 and 17000–73000 different raw sequences (>5 copies) were identified from the HC protein and EV fractions respectively, 700–5000 of which were shared. When UTI samples were compared to HC, no sequences were differentially expressed in the EV fractions (adjusted p‐value < 0.05) and 172 were differentially expressed in protein fractions. Principle component analysis separated the UTI and HC groups based on the small RNA profiles of the protein fractions, but not based on the small RNA profiles of EV fractions.

**Summary/Conclusion**: EV and protein fractions obtained after SEC of 0.22 um filtered dog urine have distinct small RNA profiles. When HC and UTI samples were compared, differentially expressed RNA sequences were identified in the protein fractions, but no significant differences in the EV fractions were demonstrated. These results suggest that UTI does not lead to changes in the small RNA profile of small EVs in urine.

### PS20.03 Extracellular vesicles from Bothrops jararaca venom gland: The possible role in cross communication with mammalian cells and in the venom processing

Larissa G. Machado, Universidade Federal do Rio de Janeiro (UFRJ) ‐ Brazil

Brunno Verçoza, Universidade Federal do Rio de Janeiro (UFRJ)

Fabio Nogueira, Universidade Federal do Rio de Janeiro (UFRJ)

Rafael Melani, Northwestern University

Gilberto Domont, Universidade Federal do Rio de Janeiro (UFRJ)

Juliany Rodrigues, Universidade Federal do Rio de Janeiro (UFRJ)

Silas rodrigues, Universidade Federal do Rio de Janeiro (UFRJ)

Russolina Zingali, Universidade Federal do Rio de Janeiro (UFRJ)

**Introduction**: The snake venom extracellular vesicles (SVEVs) is a very new topic inside and outside of the toxinology community. To date, few articles approached the presence of EVs in snake venoms, and they did not deepen in the biological roles. In the Viperidae family of snakes, the venom is produced and stored in the lumen of the venom gland, where it is processed through unknown mechanisms. The involvement of SVEVs in venom processing and envenomation, and their possible roles in cross‐organism communication are also still unknown. In this study, we used the well characterized B. jararaca venom as a tool to explore the SVEVs.

**Methods**: Fresh venom was fractionated by sequential centrifugation, resulting in two populations of vesicles (Bj‐EVs). Purified Bj‐EVs were analyzed by electron microscopy, NTA and proteomics. The interaction of Bj‐EVs with mammalian cells was accessed by fluorescence and electron microscopy.

**Results**: Bj‐EVs possess typical size range and morphology. We identified by proteomic analysis conserved EVs markers such as Ras‐related proteins, flotilins, annexins and syntenin, which have not been related to venoms so far. Interestingly, we identified important enzyme families (e.g. aminopeptidases) suggesting the contribution of the EVs in the processing of the B. jararaca venom. One minor component in snake venoms but the most abundant in the Bj‐EVs was the 5’‐nucleotidase, characterized as important for cell‐cell adhesion and function. Moreover, we are demonstrating for the first time that mammalian cells efficiently internalize Bj‐EVs and that the commercial antivenom can partially recognize Bj‐EVs.

**Summary/Conclusion**: In the context of the poorly explored EVs from reptiles, in general, this study is crucial for the comprehension of the EVs as a potential mediator in cross‐talk among organisms. It also supports the hypothesis of EVs involvement in venom production, processing and envenoming.

### PS20.04 Nanoalgosomes: microalgae‐derived extracellular vesicles in in vitro and in vivo cellular uptake studies

Giorgia Adamo, Institute for Research and Biomedical Innovation (IRIB) ‐ National Research Council of Italy (CNR), Palermo, Italy

Pamela Santonicola, Institute of Biosciences and BioResources (IBBR) ‐ National Research Council of Italy (CNR), Naples, Italy

Daniele Paolo Romancino, Institute for Research and Biomedical Innovation (IRIB) ‐ National Research Council of Italy (CNR), Palermo, Italy

Sabrina Picciotto, Institute for Research and Biomedical Innovation (IRIB) ‐ National Research Council of Italy (CNR), Palermo, Italy

Estella Rao, Institute of Biophysics (IBF) ‐ National Research Council of Italy (CNR), Palermo, Italy

Angela Paterna, Institute of Biophysics (IBF) ‐ National Research Council of Italy (CNR), Palermo, Italy

Samuele Raccosta, Institute of Biophysics (IBF) ‐ National Research Council of Italy (CNR), Palermo, Italy

Antonella Cusimano, Institute for Research and Biomedical Innovation (IRIB) ‐ National Research Council of Italy (CNR), Palermo, Italy

Rosina Noto, Institute of Biophysics (IBF) ‐ National Research Council of Italy (CNR), Palermo, Italy

Nicolas Touzet,Centre for Environmental Research Innovation and Sustainability Institute of Technology Sligo

Mauro Manno, Institute of Biophysics, National Research Council of Italy

Elia Di SchiaviInstitute of Biosciences and BioResources (IBBR) ‐ National Research Council of Italy (CNR), Naples, Italy

Antonella Bongiovanni, PhD, Institute for Biomedical Research and Innovation, National Research Council of Italy

**Introduction**: Nanoalgosomes are newly characterised subtype of small extracellular vesicles (EVs) derived from microalgae, which we are exploiting as novel and scalable biogenic nanotechnology for the delivery of bioactive compound in different theranostic applications. Here, we focus on the cellular uptake of nanoalgosomes derived from the microalgal species Tetraselmis chuii and their characterization using multiple orthogonal techniques.

**Methods**: Nanoalgosomes were separated from a suspension of Tetraselmis chuii cells using differential ultracentrifugation (dUC) or tangential flow filtration (TFF). After the physicochemical characterization (according to MISEV‐2018 guidelines), they were labelled with membrane‐specific fluorescent dyes and validated by F‐NTA and infrared‐fluorescence assay. The uptake of the nanoalgosomes was monitored by epifluorescence and confocal microscopy in normal and tumoral mammalian cells and in vivo using the animal model Caenorhabditis elegans.

**Results**: The studies performed on normal and tumour mammalian cells and in C. elegans demonstrated that nanoalgosomes are uptaken in a dose and a time dependent manner, through an energy‐dependent mechanism.

**Summary/Conclusion**: Nanoalgosomes are small EVs that can be efficiently taken up by mammalian cells, confirming the cross kingdom communication potential of EVs. These data were also corroborated by in vivo studies performed with C. elegans in which intestinal cells internalized the labelled EVs. Nanoalgosomes will be further explored as novel and natural delivery systems of high‐value microalgal substances (such as antioxidants, pigments, lipids and complex carbohydrates), bioactive biomolecules (e.g.,proteins, miRNA, siRNA, mRNA, lncRNA, peptides) and/or synthetic drugs.

### PS20.05 Nanoparticle Tracking Analysis reveals differences in mode size distribution of exosomes isolated from High‐ and Low‐Fertility Dairy Cows

Natalie P. Turner, Queensland University of Technology (QUT)

**Introduction**: The reproductive status of dairy cows continues to pose challenges to dairy farmers worldwide, with a decline in fertility associated with increased costs, impaired immunity, poor metabolic transition around the time of calving, and longer interval until return to oestrous. Early biomarkers of fertility in dairy cows are yet to be established, however recent developments regarding exosome particle analysis as biomarkers of health and disease may provide valuable information to dairy farmers early on in the life of the cow.

**Methods**: Dairy cows (n = 30) were divided into six groups based on genetic merit and physical attributes (Fertility Breeding Value (FBV)), and duration of post‐partum anoestrous interval (PPAI). Exosomes were isolated from 10mL blood plasma via sequential centrifugation and size exclusion chromatography (SEC). Exosomal fractions were subjected to nanoparticle tracking analysis (NTA) to determine particle concentration and size distribution.

**Results**: The mode sizes of exosomes isolated from high‐FBV cattle were significantly larger than the mode sizes of exosomes isolated from dairy cows in the low‐FBV groups (High FBV long PPAI vs Low FBV long PPAI: p = 0.0215; High FBV short PPAI vs Low FBV short PPAI: p = 0.012; High FBV short PPAI vs Low FBV long PPAI: p = 0.0018). Exosome mode sizes were similar within the two high‐FBV groups, and also within the four low‐FBV groups (p > 0.05).

**Summary/Conclusion**: NTA and mode size comparison of exosomes derived from the blood plasma of dairy cows may assist farmers in identifying cows at‐risk of poor reproductive outcomes.

### PS20.06 OMIC characterization of cow, donkey and goat milk extracellular vesicles reveals their anti‐inflammatory and immune‐modulatory potential

Samanta Mecocci, Department of Veterinary Medicine, University of Perugia

Stefano capomaccio, Department of Veterinary Medicine, University of Perugia

Daniele Pietrucci, Department for Innovation in Biological, Agro‐food and Forest systems, University of Tuscia

Marco Milanesi, Department for Innovation in Biological, Agro‐food and Forest systems, University of Tuscia

Luisa Pascucci, Department of Veterinary Medicine, University of Perugia

Giovanni Chillemi, Department for Innovation in Biological, Agro‐food and Forest systems, University of Tuscia

Katia Cappelli, Department of Veterinary Medicine, University of Perugia

**Introduction**: Other than being a valuable nutrition source, milk represents a sophisticated signaling system that delivers maternal messages. This property seems to be mostly mediated by Extracellular Vesicles (EVs) that act as signal mediators between distant cells and/or tissues, exerting many biological effects. Moreover, milk is among the most promising scalable and reliable source of EVs. Our aim is to characterize the molecular content of cow, donkey and goat milk EVs (MEVs) through RNA and metabolites omic analysis in view of prospective applications as a nutraceutical in inflammatory conditions.

**Methods**: RNA sequencing of MEVs was carried out highlighting over 10,000 transcripts in each species. To compare MEV cargos, orthologous genes were selected and ranked by relative expression level.

**Results**: Within the 10% of the most expressed orthologous genes in all three species (1223), 110 were shared. Donkey and goat were the most similar species with 335 shared genes while cow had only 170 genes in common with donkey and 155 with goat. Functional analysis on the 110 core genes revealed as among the main enriched GO terms those relative to protein formation. Moreover, “negative regulation to oxidative stress” and “IL12‐mediated signaling pathway” emerged, indicating potential involvement in innate and acquired immunity. These terms were also confirmed in analysis on species pairwise shared genes where “ATP metabolic process” and “generation of metabolite and energy precursors” suggested also communication on energy metabolism. Concerning the most abundant genes for each species, donkey and goat MEVs displayed additional terms relative to the immune system and amino acid metabolism.

**Summary/Conclusion**: These results are in accordance, with some peculiarities, with our previous metabolomic analysis where common pathways among the three species involving metabolites with immunomodulating effects were identified, such as arginine, asparagine, glutathione and lysine.

### PS20.07 The characterisation of equine synovial fluid and plasma extracellular vesicles

Emily J. Clarke, BSc (Hons) MRes, University of Liverpool, Institute of Life Course and Medical Sciences

James R. Anderson, BSc(Hons) BVetMed MRes PhD AFHEA MRCVS, University of Liverpool

Alexandra P. Shephard, NanoView Biosciences

Agnieszka Turlo, PhD DVM MRCVS, University of Liverpool

Peter Clegg, MA Vet MB PhD CertEO DipECVS FRCVS, University of Liverpool

Victoria James, University of Nottingham

Mandy J. Peffers, BSc MPhil PhD BVetMed FRCVS, University of Liverpool

**Introduction**: Extracellular vesicles (EVs) are nanoparticles involved in intracellular communication, contributing to osteoarthritis development, serving as potential disease biomarkers and therapeutic targets. EVs should be characterised to confirm isolation; MISEV2018 minimal information for studies guidelines, including surface marker identification; CD9, CD81 and/or CD63. However, until now the identification of antibodies for equine EV marker proteins has been problematic.

**Methods**: In order to characterise equine EVs in accordance with MISEV 2018 guidelines a preliminary experimental study was conducted. Equine synovial fluid (SF) from the metacarpophalangeal joint and plasma was utilised EVs were isolated using size exclusion chromatography and subject to nanoparticle tracking analysis (NTA), and transmission electron microscopy (TEM). Isolated EVs were analysed on the ExoView platform using human and mouse tetraspanin chips and fluorescently stained for CD9, CD63 and CD81.

**Results**: NTA determined the plasma sample concentration to be 2.02 × 109 particles/ml and the SF 1.16 × 108 particles/ml. TEM confirmed the size of EVs. The ExoView system demonstrated human antibody cross‐reactivity for CD9 (1.4 × 108 particles/ml) CD63 (6 × 107 particles/ml) and CD81 (7 × 107 particles/ml). Most EVs detected were < 100nm, and plasma EVs were 5–10% larger than SF EVs. Colocalization analysis found the majority of exosomes were CD9+ (SF = 84%, plasma = 91%) or CD9+/CD81+ (SF = 6%, plasma = 3%).

**Summary/Conclusion**: The ExoView human tetraspanin chip is compatible with equine EVs, enabling the identification of equine exosome surface markers for the first time to our knowledge. Equine EVs can now be characterised according to MISEV2018 guidelines enabling the expansion of EV research. This is fundamental to studying the role of EVs in pathophysiology, and musculoskeletal regenerative therapeutics to be used in equine clinical practice.

### PS20.08 The ratio of ω6:ω3 fatty acids in the maternal diet affects the composition of microRNA contained in exosomes from blood plasma of piglets

Rafaela Furioso Ferreira, Institute of Animal Science, Physiology Unit, University of Bonn

Raffaele Calogero, Department of Molecular Biotechnology and Health Sciences, University of Torino

Maddalena Arigoni, Department of Molecular Biotechnology and Health Sciences, University of Torino

Fabrizio Ceciliani, Department of Veterinary Medicine, University of Milan

Alessandro Agazzi, Department of Health, Animal Science and Food Safety ‘Carlo Cantoni’ (VESPA), University of Milan

Giovanni Savoini, Department of Health, Animal Science and Food Safety ‘Carlo Cantoni’ (VESPA), University of Milan

Vladimir Mrljak, Faculty of Veterinary Medicine, University of Zagreb

Helga Sauerwein, Institute of Animal Science & Physiology Unit, University of Bonn

**Introduction**: Lowering the ratio of ω6:ω3 polyunsaturated fatty acids (PUFA) in maternal diets may promote the health and performance of the sows and their piglets. Yet, the optimal ratio of PUFA in the diet and the mode of transmitting the effects from mother to offspring are largely unknown. The effects of two different ω6:ω3 ratios in the sows’ diet on the microRNA profile in the piglets’ plasma exosomes were tested.

**Methods**: Sows were randomly allocated to either the control group (CR, n = 8) receiving a standard diet (ω6:ω3 = 10:1), or the treatment group (LR, n = 8) fed at a lower dietary ω6:ω3 ratio (4:1) from day 28 of gestation until the end of lactation. Plasma samples were collected at weaning (day 26 of life) from 12 piglets (6 per maternal diet group, all from different mothers). Exosomes were isolated using Exoquick™ Precipitation Solution and characterized by Nanoparticle Tracking Analysis. RNAs were purified and small RNA libraries were prepared for miRNA and short non‐coding RNAs. The miRNA sequencing was performed using the NextSeq 500 sequencer (Illumina). Data analysis was carried out using the docker4seq package in R studio.

**Results**: In total, 193 differently expressed miRNA, 145 up‐regulated and 48 down‐regulated, were identified when comparing the plasma exosomes from piglets born to sows from the CR versus the LR group. These results indicate that exosomes may play a significant role in the transmission of cargo from dietary sources from mother to offspring, which may finally alter gene expression in the infant.

**Summary/Conclusion**: Different dietary ω6:ω3 ratios in the maternal diet affected the exosomal miRNA composition of their piglets’ plasma exosomes. This information may improve the understanding of the mother‐to‐offspring crosstalk, with impacts on newborn health and future biotechnological perspectives.

### PS20.09 Comparative proteomics of extracellular vesicles subsets isolated from pig seminal plasma

Isabel Barranco, Department of Veterinary Medical Sciences, University of Bologna, Ozzano dell ' Emilia, Bologna, Italy

Christian M. Sánchez‐López, Area de Parasitologia, Departamento de Farmacia y Tecnología Farmacéutica y Parasitología, Universitat de València, Burjassot Valencia, Spain; Joint Research Unit on Endocrinology, Nutrition and Clinical Dietetics, Health Research Institute La Fe, Univers

Antonio Marcilla, PhD, Area de Parasitologia, Departamento de Farmacia y Tecnología Farmacéutica y Parasitología, Universitat de València, Burjassot Valencia, Spain; Joint Research Unit on Endocrinology, Nutrition and Clinical Dietetics, Health Research Institute La Fe, Univers

Jordi Roca, Department of Medicine and Animal Surgery, Faculty of Veterinary Science, University of Murcia, Spain; Institute for Biomedical Research of Murcia (IMIB‐Arrixaca), Murcia, Spain

**Introduction**: Extracellular vesicles (EVs) of seminal plasma (SP) play a key role in sperm functionality, even fertility. Little is known about the seminal EVs composition. This study evaluated the proteomic profile of EV subsets of pig SP.

**Methods**: Three pools of SP were analyzed. Each pool contained SP from 3 ejaculates from boars used in artificial insemination. The pools were sequentially centrifuged (3,200g/15 min and 20,000g/30 min at 4°C), and the pellets (washed and solubilized in PBS), and supernatants (0.22μm‐filtered and concentrated with Amicon(R)‐10K), and were subjected to size‐exclusion chromatography for EVs isolation. The fractions 7–9 were selected and mixed. The isolated EVs were analyzed by nanoparticle tracking analysis (NTA) and transmission electron microscopy (TEM). Quantitative proteomics was performed using a SWATH‐MS strategy. Proteins were considered quantitatively different with a p ˂ 0.05 and a Log2 fold‐change > ±2. Gene Ontology (GO) enrichment analysis was performed using Cytoscape plug‐in ClueGO.

**Results**: The concentration (mean±sd) of EVs was 1.4 × 1012 ± 1.1 × 1012 and 3.7 × 1011 ± 2.2 × 1011 particles/mL in samples from supernatants and pellets, respectively. NTA showed differences in EVs size‐distribution between pellet (peaks between 75 and 194 nm) and supernatant samples (more polydisperse, showing peaks between 167 and 354 nm). TEM confirmed the above differences in size. Accordingly, pellets contained larger EVs (enriched in microvesicles, MVs) than supernatant (enriched in exosomes, EXOs). A total of 737 proteins were identified and quantified. Differential quantification analysis revealed that 151 proteins were upregulated and 25 downregulated in MV samples compared to EXO samples. GO enrichment for biological processes revealed that EXO samples were enriched in proteins related to exopeptidase activity, whereas MV samples in those related to cell redox homeostasis and intrinsic apoptosis signaling pathway in response to hydrogen peroxide.

**Summary/Conclusion**: This study showed clear differences in the proteomic profile between the large and small EV subsets of pig SP.

### PS20.10 Identification of anti‐inflammatory vesicle‐like nanoparticles in honey

Jiujiu Yu, University of Nebraska Lincoln

**Introduction**: Honey has been used as a nutrient, an ointment, and medicine worldwide for many centuries. Modern research has demonstrated that honey has many medicinal properties, reflected in its anti‐microbial, anti‐oxidant, and anti‐inflammatory bioactivities. Honey is composed of sugars, water, and a myriad of minor components, including minerals, vitamins, proteins, and polyphenols.

**Methods**: In the current study, we investigate whether honey contains vesicle‐like nanoparticles (VLNs). We employ ultracentrifugation to extract VLNs from honey (H‐VLNs) and characterize such nanoparticles in detail. We also use cell culture and animal models to investigate whether these H‐VLNs have any anti‐inflammatory functions.

**Results**: We have found that VLNs are present in honey. These H‐VLNs are membrane‐bound nano‐scale particles that contain lipids, proteins, and small‐sized RNAs. The presence of plant‐originated plasma transmembrane proteins and plasma membrane‐associated proteins suggests the potential vesicle‐like nature of these particles. H‐VLNs impede the formation and activation of the nucleotide‐binding domain and leucine‐rich repeat related (NLR) family, pyrin domain containing 3 (NLRP3) inflammasome, which is a crucial inflammatory signaling platform in the innate immune system. Intraperitoneal administration of H‐VLNs in mice alleviates inflammation and liver damage in the experimentally induced acute liver injury. miR‐4057 in H‐VLNs was identified in inhibiting NLRP3 inflammasome activation.

**Summary/Conclusion**: Together, our studies have identified anti‐inflammatory VLNs as a new bioactive agent in honey.

### PS20.11 Molecular and Immunological characterization of strawberry‐derived nanovesicles: A study of allergen transporting

Christopher Stanly, EVs&MS Laboratory, Institute of Biosciences and BioResources, National Research Council of Italy

Immacolata Fiume, EVs&MS Laboratory, Institute of Biosciences and BioResources, National Research Council of Italy

Giuseppe Antonucci, EVs&MS Laboratory, Institute of Biosciences and BioResources, National Research Council of Italy

Michele Guescini, University of Urbino Carlo Bo

Kwang Pyo Kim, Department of Applied Chemistry, Institute of Natural Science, Global Center for Pharmaceutical Ingredient Materials, Kyung Hee University, Yongin, South Korea; Department of Biomedical Science and Technology, Kyung Hee Medical Science Research Institute,

Hyoseon Kim, Department of Applied Chemistry, Institute of Natural Science, Global Center for Pharmaceutical Ingredient Materials, Kyung Hee University, Yongin, South Korea; Department of Biomedical Science and Technology, Kyung Hee Medical Science Research Institute,

Yeong Eun Cho, Department of Applied Chemistry, Institute of Natural Science, Global Center for Pharmaceutical Ingredient Materials, Kyung Hee University, Yongin, South Korea

Claudia Rafaiani, Allergy Data Laboratories (ADL), Latina, Italy; Associated Centers for Molecular Allergology (CAAM), Rome, Italy

Maria Antonietta Ciardiello, Institute of Biosciences and BioResources, National Research Council of Italy, Naples, Italy

Adriano Mari,Allergy Data Laboratories (ADL), Latina, Italy; Associated Centers for Molecular Allergology (CAAM), Rome, Italy

Gabriella Pocsfalvi, EVs&MS Laboratory, Institute of Biosciences and BioResources, National Research Council of Italy

**Introduction**: The delicious strawberries (Fragaria x ananassa) are a symbol of perfect goodness. However, due to the presence of various allergenic proteins, it can cause allergic reactions. Here we used a multidisciplinary approach to investigate the role of nanovesicles (NVs) isolated from strawberry in allergenicity. The strategy combines proteomics‐based protein identification, immunological detection, data mining and bioinformatics to gain biological insights useful to evaluate the potential allergens in vesicles.

**Methods**: NVs from strawberry were isolated by differential ultracentrifugation and separated based on density using sucrose density gradient ultracentrifugation. The total yields in terms of protein concentration were determined by BCA assay. The size‐distribution and the vesicle concentration were determined by NTA. Protein profile was obtained by SDS‐PAGE analysis. LC‐ESI‐MS/MS analyses were performed on an Orbitrap instrument. Proteins were identified against the proteome of Fragaria vesca extracted from the NCBI database and quantified using the MaxQuant software package. The allergen profiles in NVs were investigated by IgE inhibition test performed with the single point highest inhibition achievable assay (SPHIAa) in combination with multiplex biochip‐based immunoassay on the FABER(R) system containing 244 allergens, including all of the most important allergy markers.

**Results**: We have successfully isolated NVs from strawberry with a yield of 18 mg/kg of fruit and proved the vesicle characteristics. Proteomics identified 1416 and 1559 proteins in total tissue lysate and NVs, including the frequently identified proteins in edible plant‐derived NV preparations, like ATPase, HSPs, actin, myosin, clathrin, 14‐3‐3 like proteins. Moreover, a BLAST search against the allergen database highlighted a set of potential allergens within the identified protein set. Immunological tests confirmed that the proteins of NVs from strawberry compete for IgE binding with some allergens spotted on the FABER biochip. This includes the major strawberry's allergens Fra a 1, Fra a 3 and Fra a 4, but also other IgE‐binding proteins not yet described in this food, like gibberellin‐regulated proteins, 2S albumin, pectate lyase and trypsin inhibitor.

**Summary/Conclusion**: A combined proteomic and immunological characterization of strawberry‐derived NVs reveals the presence of potential allergens, several of which are recognized by specific IgE contained in the sera of allergic patients. It is worth noting that in addition to the already known allergens, this study reveals possible new allergens not yet reported in strawberry.

### PS20.12 Divergence of gut bacteria through the selection of genetic variations by extracellular vesicles in milk

Janos Zempleni, Ph.D., University of Nebraska‐Lincoln

Fang Zhou, Ph.D., University of Nebraska‐Lincoln

Haluk Dogan, University of Nebraska‐Lincoln

Juan Cui, Ph.D., University of Nebraska‐Lincoln

**Introduction**: EVs facilitate cell‐to‐cell communication and are present in most body fluids including milk. Gram‐positive and Gram‐negative bacteria communicate with their environment through outer membrane vesicles. We demonstrated that mouse pups absorb EVs from maternal milk and a fraction of milk EVs (MEVs) escapes absorption and reaches the large intestine. MEVs altered the composition of bacterial communities in murine ceca.

We hypothesized that MEVs select genetic variations in gut bacteria, thereby contributing to the divergence of bacterial populations.

**Methods**: Ceca content was collected from three mice, age 7 weeks, suspended in minimal salts media and divided into two aliquots. One aliquot was cultured in media containing a nutritionally relevant concentration of MEVs (1.7 × 1010/mL; denoted MEV‐supplemented, MEVS) under anaerobic conditions for 7 days; the other aliquot was cultured in MEV‐free media (MEVF). DNA was sequenced using a 75‐bp single end protocol (estimated coverage 150x). Genetic variations were assessed by using the MIDAS and StrainPhlAn pipelines.

**Results**: Bioinformatics analyses were performed by using 127,935,309±30,104,915 and 138,253,606±25,740,862 reads per sample in MEVS and MEVF cultures, respectively (Nj). MIDAS: More than 200 and 190 million sequencing reads were mapped to 11 and 19 bacterial species in MEVS and MEVF cultures, respectively. In MEVS cultures, 278 and 28,594 strain‐level genetic variations were detected by high stringency (detected in all 3 cultures) and low stringency (detected in 2 out of 3 cultures) analyses, respectively. Ninety‐five genes in 11 bacterial species carried non‐synonymous SNPs in MEVS cultures in the high stringency dataset. In MEVF cultures, 92 and 26,382 strain‐level genetic variations were detected by high and low stringency analyses, respectively. Forty‐two genes in 19 bacterial species carried non‐synonymous SNPs in MEVF cultures in the high stringency dataset. Genetic variations were detected in enzymes catalyzing essential steps in the metabolism of tryptophan, glutamate and purines, i.e., pathways implicated in neurotransmitter synthesis in the host. StrainPhlAn: We detected 6,715 genetic variations across all loci in E. faecalis, C. sporogenes and L. johnsonii for both MEVS and MEVF combined, including 6,694 variations in protein coding regions: 5,182 non‐synonymous (77%) and 1,512 synonymous (23%) variations. We detected 62 insertions and 75 deletions among the non‐synonymous variations.

**Summary/Conclusion**: MEVs contribute to the divergence of gut bacteria through the selection of genetic variations, which might affect neuronal signaling in the host.

## PS21 EVs and Brain: From Drug Abuse to Aging

Chair: Guillaume Van Niel, IPNP INSERM U1266, France

Chair: Tanina Arab, Department of Molecular and Comparative Pathobiology, Johns Hopkins School of Medicine, Johns Hopkins University, Baltimore, MD, USA

### PS21.02 Mitogenic growth factors facilitate astrocyte modulation of NSC proliferation & diffrentiation via exosome‐mediated communication: Role of miRNA transport

Chen xingming, Unverisity of Electronic science and technology of China

lU YANG, University of Electronic Science and Technology of China

**Introduction**: Astrocytes are increasingly recognized as essential regulators of adult neurogenesis through control of neural stem cells (NSCs) proliferation and fate determination. microRNAs are part of the complex molecular cargo found in astrocyte‐derived exosome (ADEs), which have been implicated as primary facilitators of astrocyte mediated cellular regulation. Mitogenic growth factors bFGF and EGF has been used to promote endogenous neurogenesis after brain injury, however, whether astrocytes regulate NSCs proliferation and differatiation in response to bFGF and EGF via ADEs were largely unknown. In current study, we carefully exploited the contributions of ADEs to the growth of EGF and bFGF‐dependent NSCs in vitro and dissect the role of miRNA cargo in regulating NSCs proliefartion and differentiation. We also explored the role of those ADEs in regulating endougenou neurogenesis in the MPTP impaired PD mouse model.

**Methods**: Exosome were collected via ultracentrifugation. NSC proliferation assay was determined by CyQUANT assay and cell counting. The miRNA array followed with bioinformatic analysis were used in this study. Genetic approach was used to remove the Dicer‐dependent miRNA cargo in ADEs. Stereotactic injection was used in in vivo study. Endogenous neurogenesis was evaluated by BrdU labeling assay.

**Results**: In this study, we show that astrocytes exhibited a neurotropic phenotype in the presence of bFGF and EGF maintained co‐culture system, and demonstrated that ADEs contributed to enhanced NSC proliferation. We detected altered miRNA cargo in MGF‐ADEs compared with ADEs continuously released from non‐stimulated control astrocytes (CTRL‐ADEs). Following bioinformatic analysis, the miRNA changes correlated well with target genes that regulate cell development and neuronal differentiation. Interestingly, upon removing the Dicer‐dependent miRNA cargo in ADEs, we found that miRNA cargo is essential to MGF‐ADE‐mediated NSC neuronal fate specification instead of NSC self‐renewal. We further validated the effect of MGF‐ADEs in increasing endogenous neurogenesis in vivo.

**Summary/Conclusion**: In conclusion, our results collectively led us to postulate that astrocyte‐derived exosomes might serve as essential modulators within the neurogenic niche to regulate the proliferation and/or differentiation of NSCs in the presence of bFGF and EGF.

### PS21.04 Super‐resolution live‐cell imaging reveals the changes of subcellular structure of hypomyelination leukodystrophies diseases caused by TMEM106B mutation

Shijia Xing, Peking University

Shiqun Zhao, Peking university

Xiaolu Zheng, Peking University

Ruoyu Duan, Peking University First Hospital

Jingmin Wang, Peking University First Hospital

Liangyi Chen, Peking University

**Introduction**: Hypomyelination leukodystrophies diseases (HLDs) represent a group of heritable white matter disorders characterized by defects in myelin development. TMEM106B (TM), initially identified as an FTLD risk factor, has been associated with HLD recently for its dominant mutation D252N found in patients with hypomyelination. However, it is still unclear how TM D252N mutation leads to myelin abnormality. Previous research found that TM was a type II transmembrane protein and localized to late endosomes and lysosomes. To explore the pathological mechanism of D252N mutant, we utilized Hessian SIM and SD SIM super‐resolution live‐cell imaging to analyze the subcellular localization and dynamic process of TM Wide type (WT) and mutation in the human oligodendrocyte cell line. In our research, TM was colocalized with the major myelin protein PLP1, while the intracellular vesicle size of the D252N mutant was smaller than WT. Besides, we revealed the novel localization of TM to plasm membrane (PM) and extracellular vesicles (EVs).

**Methods**: TM is a single‐pass transmembrane protein with 5 glycosylation sites among which N4 and N5 are complex glycosylated and important for localization and function. WT, N4mut, N5mut, D252N TM were designed and subcloned into vectors with fluorescent protein tag. After expression of fluorescent protein‐labeled TM in human oligodendrocyte cell line Mo3.13 by plasmids transfection, the subcellular localizations and dynamics of TM could be examined by Hessian SIM and SD SIM super‐resolution live‐cell imaging.

**Results**: TM colocalized with the lysosome/late endosome (Ly/LE) marker Rab7 and LAMP1. TM‐labeled intracellular vesicles of D252N (about 0.51 μm) was smaller than WT and N5mut (about 0.63 μm). After starvation, TM‐labeled intracellular vesicles of D252N had no significant difference while WT and N5mut became larger. By co‐transfection with PLP1, TM and PLP1 were colocalized to both intracellular vesicles and PM, EVs. TM‐labeled EVs were produced by the breakage of retraction fibers during cell migration which depended on actin polymerization.

**Summary/Conclusion**: 1.TMEM106B is localized to Ly/LE as well as PM and EVs, and is colocalized with PLP1.

2.The intracellular vesicle size of D252N mutant is smaller than WT and N5mut.

3.TMEM106B‐labeled EVs will be produced during oligodendrocyte migration.

### PS21.05 Sex effects on brain extracellular vesicles during aging

Yohan Kim, Nathan S. Kline Institute

Michelle Kurz, Center for Dementia Research, Nathan S. Kline Institute, Orangeburg, New York 10962, USA

Adaora Aroh, Center for Dementia Research, Nathan S. Kline Institute, Orangeburg, New York 10962, USA

Efrat Levy, Center for Dementia Research, Nathan S. Kline Institute, Orangeburg, New York 10962, USA

**Introduction**: Sex‐specific differences exist in the brain, including the differential modulation of brain networks. Sex, in addition to aging, is an established risk factor for neurodegenerative diseases, with females at higher risk for Alzheimer's disease and lower risk for Parkinson's disease compared to males. Extracellular vesicles (EVs) in the normal brain play a role in neuronal homeostasis by removing intracellular accumulated material and regulating cell‐to‐cell communication. We have investigated sex‐dependent differences in EV levels and their content in the brain during aging.

**Methods**: EVs were isolated from the brains of 3, 6, 12, 18, and 24‐month‐old female and male C57BL/6 mice. EVs were analyzed by nanoparticle tracking analysis, estimation of total protein content, and Western blot analysis using antibodies to different EV subtype markers.

**Results**: Age‐dependent effects were seen in brain EVs isolated from both sexes, although the differences were found to be more prominent in female mice. Annexin A2‐containing plasma membrane‐derived microvesicles increased with aging in female mice; no significant changes were seen in males. While CD63‐containing exosomes increased with age in both sexes, additional exosomal markers appeared to uniquely increase in the EVs isolated from female brains. VDAC, a marker for mitochondria‐derived mitovesicles, was found to be significantly increased during aging in EVs isolated from both sexes.

**Summary/Conclusion**: Our findings show sex‐independent increases in mitovesicles in the brain during aging, a change that may reflect aging‐dependent alterations in mitochondrial function. While aging alters brain exosomes in both sexes, our data suggest that sex differences in exosomal pathways may manifest during aging. Finally, sex appears to be an important determinant for changes in brain microvesicles during aging. Thus, the composition of brain EVs is sensitive to both sex and age.

### PS21.06 Extracellular vesicles mediate anti‐oxidative response. In‐Vitro study in the ocular drainage system

Elie Beit‐Yannai, Ben‐Gurion University

Natalie Lerner, Ben‐Gurion University

Sofia Schreiber‐Avissar, Ben‐Gurion University

**Introduction**: The importance of EVs as signaling mediators has been emphasized for several pathways with only limited data regarding their role as protective messages during oxidative stress. The ocular drainage system is unique by being continuously exposed to oxidative stress and a having one‐way flow of the aqueous humor carrying EVs taking role in primary open angle glaucoma. Here, we aimed to examine the ability of EVs derived from the aqueous humor producing cells (NPCE) exposed to oxidative stress, to deliver protecting messages to the aqueous humor draining cells (TM), a process with significance to the pathophysiology of glaucoma disease.

**Methods**: EVs extracted from media of NPCE cells by a series of ultracentrifuges, were exposed to non‐lethal oxidative stress and their un‐stressed control were incubated with TM cells. The effects of EVs derived from oxidative stressed cells on the activation of the NRF2‐Keap1, a major oxidative stress pathway and the Wnt pathway, known for its role in primary open angle glaucoma, were evaluated.

**Results**: EVs derived from oxidized NPCE cells significantly protected TM cells from direct oxidative stress. The TM cells uptake of EVs from oxidized NPCE was significantly higher and their cytosolic NRF2 levels were significantly higher at 8h post exposure. EVs derived from oxidized NPCE cells significantly attenuated Wnt protein expression in TM cells and activated major antioxidant genes as measured by qRT‐PCR. The TM cells exposed to EVs derived from oxidized NPCE cells exhibited significantly lower oxidative stress and higher SOD and Catalase activity. Finally, we were able to show that carbonylated proteins, end products of oxidized protein, are presented in significantly higher levels in EVs derived from oxidized NPCE cells, supporting their suggested role in the signaling process.

We hypothesize that these findings may have implications beyond understanding the pathophysiology of glaucoma disease and that transmitting signals that activate the antioxidant system in target cells represent a broad response common to many tissues communication.

**Summary/Conclusion**: We hypothesize that these findings may have implications beyond understanding the pathophysiology of glaucoma disease and that transmitting signals that activate the antioxidant system in target cells represent a broad response common to many tissues communication.

### PS21.07 Isolation and characterisation of human fetal neural stem cell extracellular vesicles

Teena K. Gamage, The University of Auckland

Yohanes Nursalim, The University of Auckland

Mhoyra Fraser, PhD, The University of Auckland

**Introduction**: Regenerative therapies for the treatment of stroke, traumatic brain injury and perinatal brain injuries are heralded as having a great potential to reduce the devastating burden of symptoms, particularly in the young. Traditionally non‐neuronal stem cell‐derived extracellular vesicles (EVs), such as those derived from mesenchymal stem cells are investigated for their regenerative potential. However, they may not be the optimal source of neuroregenerative EVs. The fetal brain is the best source of neural stem cells and thus more likely to produce EVs with a greater capacity to promote neural plasticity and neuroregeneration. To our knowledge, EVs have not yet been isolated from human fetal neural stem cells (hFNSCs) therefore our aim was to establish a method to isolate hFNSC‐EVs as a potential neuroregenerative therapy.

**Methods**: Conditioned media was collected from hFNSCs grown in serum‐free conditions. To isolate hFNSC‐EVs, the conditioned media underwent sequential centrifugation (800xg for 10 min, then 2000xg for 30 min), followed by vacuum filtration (via 0.22μm filter), then size exclusion chromatography (SEC) using qEV10 columns. EV rich fractions were then concentrated using amicon filtration columns, then further characterised using nanoparticle tracking analysis (NTA), BCA assay, transmission electron microscopy (TEM), and western blotting.

**Results**: Purified EVs were successfully isolated. SEC‐fraction NTA and BCA characterisation revealed that fractions 6–9 were EV‐enriched (most EVs were 106nm‐148nm in size) and lacked protein contamination. TEM confirmed both the size and phospholipid bilayer of the EVs. Importantly, isolated EVs expressed positive EV markers such as CD63 and CD81, and the neuronal marker LCAM1, but lacked expression of negative markers such as calnexin and albumin.

**Summary/Conclusion**: hFNSC‐EVs can be successfully isolated from cell culture media. Upscaling future production of hFNSC‐EVs will be key to testing their neuroregenerative potential in animal models of human neurological disorders.

### PS21.08 The influence of exosomal miRNA cargo on the neural differentiation of human adipose‐derived stem cells

Aida Selaru, University of Bucharest, Department of Biochemistry and Molecular Biology

Alexandra Dobranici, University of Bucharest, Department of Biochemistry and Molecular Biology

Marieta Costache, University of Bucharest, Department of Biochemistry and Molecular Biology

Sorina Dinescu, University of Bucharest, Department of Biochemistry and Molecular Biology

**Introduction**: The potential of human adipose‐derived stem cells (hASCs) and their exosomal cargo has been largely studied in the past few years in the context of neuronal differentiation. hASCs‐derived exosomes represent a hot topic and lots of effort is put into exploring their capacity for stimulating the trans‐differentiation of hASCs into neuron‐like cells.Thus, our research focuses on understanding the molecular signal transduction which takes place in the exosomal exchange upon neuronal induction of hASCs.

**Methods**: After seeding of hASCs, cells were induced towards the neural lineage for 14 days, with a commercially available media. Exosomes from hASCs cultures at day 0, 7 and 14 of neural induction were isolated using a specific isolation kit, quantified using a fluorimetric method (495 nm) and then validated as exosomes by cryo‐transmission electron microscopy (TEM). Further, the exosomal content was analyzed and compared to the exosomal content of the genuine hASCs in order to identify the signals activated during the trans‐differentiation process.

**Results**: The molecular analysis of microRNAs (miRNAs) indicated the upregulation of several miRNAs, such as miR‐125b and miR‐1188 which have found to be actively involved in the differentiation process.

**Summary/Conclusion**: Based on this evidence, we aim to further expand our studies in order to correlate these findings with their use in the field of nerve regeneration.

### PS21.09 IL‐1β‐encountered astrocytes release extracellular vesicles that stimulate the expression of neuronal receptors implicated in a morphine‐addiction pathway

Sehmus Tohumeken, Johns Hopkins University

Seung‐Wan Yoo, Johns Hopkins University School of Medicine

Norman J. Haughey, Johns Hopkins University School of Medicine

**Introduction**: Neuroinflammation is known to sensitize the brain to drug addiction and increases the likelihood for relapse following a period of absence. Currently, the mechanism(s) by which inflammation sensitize the brain to drug addiction are unknown. Based on data that astrocyte derived extracellular vesicles (ADEVs) regulate neuronal function, and data that the inflammatory cytokines TNFa and IL‐1b promote the release of ADEVs with modified cargo we sought to determine if inflammation modifies addition circuitry through ADEVs

**Methods**: primary rat astrocytes stimulated with IL‐1β (200 ng/ml) released EVs (ADEV‐IL‐1β) containing 131 miRNAs, with 10 increased and 17 decreased >1.5 fold in abundance compared with constitutively released EVs (ADEV‐CR)

**Results**: We found that primary rat astrocytes stimulated with IL‐1β (200 ng/ml) released EVs (ADEV‐IL‐1β) containing 131 miRNAs, with 10 increased and 17 decreased >1.5 fold in abundance compared with constitutively released EVs (ADEV‐CR). Investigating the 17 decreased miRNAs using the Diana‐miRPath informatic system showed that miR‐106b, miR‐218a and miR‐328a mapped to the morphine addiction pathway. Bioinformatic analyses predicted these three miRNAs to target multiple genes in the morphine addiction pathway including OPRM1, KCNJ3, DRD1 and GABBR2. The targets of these three miRNAs were validated in luciferase reporter assays. Exposing primary cortical neurons to ADEV‐IL‐1b increased the expression of OPRM1, KCNJ3, DRD1 and GABBR2 mRNA and protein levels in dose dependent manner (15‐100 EVs/cell)

**Summary/Conclusion**: Collectively, our findings suggest that inflammation modifies the mRNA cargo of ADEVs to contain reduced levels of miRNA regulating the expression of OPRM1, KCNJ3, DRD1 and GABBR2. Increased expression of the corresponding proteins may be responsible for the increased sensitivity for addiction in the setting of inflammation.

### PS21.10 Extracellular vesicle‐mediated primary ciliogenesis contributes to the development of morphine tolerance

Rong Ma, University of Nebraska Medical Center

Naseer Kutchy, University of Nebraska Medical Center

Guoku Hu, University of Nebraska Medical Center

**Introduction**: Morphine is used extensively in the clinical setting owing to its beneficial effects; its therapeutic utility is limited as the prolonged use of morphine often results in the development of tolerance and addiction. Astrocytes in the brain are a direct target of morphine action and play an essential role in the development of morphine tolerance as inhibition of astrocyte activation attenuates morphine tolerance in rodents. Primary cilia and the cilia‐mediated sonic hedgehog (SHH) signaling pathways have been shown to play a role in drug resistance and morphine tolerance, respectively. Extracellular vesicles (EVs) play important roles as cargo‐carrying vesicles mediating communication among cells and tissues, including the central nervous system (CNS).

**Methods**: C57/B6 mice were administered morphine for 8 days to develop tolerance, which was determined using the tail‐flick and hot plate assays. EVs were isolated using both size exclusion chromatography (SEC) and ultracentrifugation approaches followed by full characterization of EVs using NanoSight for EV size distribution and number, and Western blotting for EV markers as well as electron microscopy for EV morphology. Human primary astrocytes were treated with either control‐ADEVs or morphine‐ADEVs for 24h followed by assessment of primary cilia. Pharmacological and genetic approaches were used to determine the role of EVs in primary ciliogenesis. Primary cilia were assessed by fluorescent immunostaining for primary cilia markers (ARL13B, Acetylated Tubulin and γ‐Tubulin) and scanning electron microscopy.

**Results**: Morphine tolerant mice exhibited an increase in primary cilia length and the percentage of ciliated astrocytes compared with control animals. The levels of SHH protein were upregulated in morphine‐stimulated astrocyte‐derived EVs (morphine‐ADEVs). SHH on morphine‐ADEVs activated SHH signaling in astrocytes through primary cilia. Our in vivo study demonstrated inhibition of either EV release or primary cilia prevents morphine tolerance in mice.

**Summary/Conclusion**: EV‐mediated primary ciliogenesis contributes to the development of morphine tolerance.

### PS21.11 Microglial derived extracellular vesicles activate autophagy to maintain cellular homeostasis

Bram Van den Broek, Hasselt University

Charlotte wuyts, Hasselt University

Isabel Pintelon, University of Antwerp

Jean‐Pierre Timmermans, University of Antwerp

Luc Michiels, Hasselt University

Joy I. Irobi, PhD, Hasselt University

**Introduction**: Microglia, the immunocompetent cells of the CNS play an important role in maintaining cellular homeostasis and are the primary source of inflammatory mediators in the CNS. These cells secrete immunomodulatory factors including membrane‐bound‐vesicles. Furthermore, microglia can remove cellular debris by phagocytosis or by autophagy. Accumulating research evidence points out that secreted membrane‐vesicles, specifically the release and uptake of small extracellular vesicles (EVs), contribute to the different aspects of physiology and disease through intercellular communication.

**Methods**: In this study, we investigated the effect of EVs on autophagy in‐vitro. Two subsets of microglia‐derived EVs (M‐EVs: non‐activated EV, nEV and TNFa‐activated EV, aEV) were produced and characterized. Using fluorescently Dil‐labeled M‐EVs, we investigated whether these EVs are incorporated in different cell types. Finally, since autophagy plays an important role in protecting cells against stress. We investigated the effect of M‐EVs subsets on the microglia autophagy process.

**Results**: Results showed the incorporation of EV into recipient cells and showed consistently that M‐EVs exposure to cells, increased the protein level of the autophagy marker LC3B‐II and promoted autophagic flux in live cells.

**Summary/Conclusion**: We demonstrate that in‐vitro produced microglia nEVs and aEVs are internalized by a recipient cell in a similar way and that microglia derived EVs are able to promote activation of autophagy.

## PS22 EV‐based Therapeutics & Delivery Vehicles

Chair: Émilie Velot, Molecular Engineering and Articular Pathophysiology/CNRS‐Université de Lorraine, Nancy, France

Chair: Olivier Blanc‐Brude, Université de Paris, Paris Cardiovascular Research Center, Inserm, Paris, France

### PS22.01 DfE‐loaded extracellular vesicles from mast cell suppress allergic asthma via allergen immunotherapy

liao huanjin, shanghai general hospital

li li, shanghai general hospital

**Introduction**: Allergen immunotherapy (AIT) is the most widely used treatment for allergic diseases that directly targets the T helper 2 (Th2) bias underlying allergy. However, the most widespread clinical applications of AIT require a long period of dose escalation with soluble antigen and carry a significant risk of adverse reactions. Thus, the development of safer, more efficient methods to reduce inflammation is critical to advancing allergy treatment. We hypothesized that extracellular vesicles (EVs) derived from the crude extract of Df (DfE) pulsed BMMCs (BMMC‐EVs‐DfE) would also be effective for the treatment of asthma disease

**Methods**: BMMC‐EVs and BMMC‐EVs‐DfE were isolated from BMMCs cultured alone (BMMC‐EVs) or from BMMCs co‐cultured with DfE (BMMC‐EVs‐DfE), and analyzed by ELISA for the presence and concentration of DfE. The allergic asthma model was established by intraperitoneal injection of DfE and Al(OH)3 in BALB/c mice. Mice were immunized with BMMC‐EVs and BMMC‐EVs‐DfE, which compared with traditional DfE immunotherapy. Then, mice were challenged with DfE after treatment. The levels of DfE specific IgG1, IgG2a, Ig2b, and IgG3 antibodies were determined using ELISA. Levels of cytokines including IFN‐γ, IL‐1β, IL‐4, IL‐5, IL‐6, IL‐17A, and IL‐33, were quantitated by murine Luminex multiplex assay. H&E and AB‐PAS staining was used to observe the histopathology of lung. Muc5ac intracellular levels were detected using IHC.

**Results**: There were no deaths or signs of systemic toxicity noted in association with the BMMC‐EVs‐DfE in mice. DfE and BMMC‐EVs‐DfE treatment could decrease the total cells macrophages and eosinophils number in BALF, as well as attenuated goblet cell hyperplasia and MUC5AC expression. DfE specific IgE antibody, IgG3 and histamine levels were significantly suppressed by DfE and BMMC‐EVs‐DfE treatments. While increase the DfE specific IgG1 and IgG2a level. DfE and BMMC‐EVs‐DfE treated mice can trigger the polarization of T cells to the Th1 type via increase the IFN‐γ and decrease the IL‐4 levels

**Summary/Conclusion**: We demonstrate here that BMMC‐EVs‐DfE could efficiently prevent allergic inflammation, down‐regulate Th2 immune response, reduce goblet hyperplasia and mucus production. The effect of BMMC‐EVs‐DfE loaded with low dose DfE is similar to traditional AIT therapy which requires high doses of allergen. Thus, we illustrate progression toward BMMC‐EVs‐DfE as a antigen carrier platform for the safe and effective inhibition of asthma airway inflammation

### PS22.02 Dynamics of hepatic extracellular vesicle release and uptake under normolipemia and hyperlipidemia

Krisztina Németh, Semmelweis University, Department of Genetics, Cell‐ and Immunobiology

Dorina Lenzinger, Semmelweis University, Department of Genetics, Cell‐ and Immunobiology

Tamás Visnovitz, Semmelweis University, Department of Genetics, Cell‐ and Immunobiology

Anna Koncz, Semmelweis University, Department of Genetics, Cell‐ and Immunobiology

Zoltán Varga, PhD, Research Centre for Natural Sciences, Institute of Materials and Environmental Chemistry

Nikolett Hegedűs, Semmelweis University, Department of Biophysics and Radiation Biology

Ildikó Horváth, Semmelweis University, Department of Biophysics and Radiation Biology

Agnes Kittel, Hungarian Academy of Sciences, Institute of Experimental Medicine

Péter Lőrincz, Eötvös Loránd University, Department of Anatomy, Cell and Developmental Biology

Viola Tamási,Semmelweis University, Department of Genetics, Cell‐ and Immunbiology

Edit Buzás, Semmelweis University, Department of Genetics, Cell‐ and Immunobiology

**Introduction**: Liver plays a central role in elimination of circulating extracellular vesicles (EVs), and it also significantly contributes to EV release. However, the involvement of the different liver cell populations remains unknown.

**Methods**: Here, we investigated EV uptake and release both in normolipemia and hyperlipidemia. C57BL/6 mice were kept on high fat diet for 20–30 weeks before circulating EV profiles were determined. In addition, mice were injected intravenously with fluorescent EVs, and an hour later, liver cell types were isolated and analysed. In vitro, liver cell types were tested for EV release and uptake with/without prior fatty acid treatment.

**Results**: We detected an elevated circulating EV number after the high fat diet. To clarify the differential liver cell involvement, we carried out in vitro experiments. We found an increased release of EVs by primary hepatocytes at concentrations of fatty acids comparable to what is characteristic for hyperlipidemia. When investigating EV uptake, upon injection of medium EVs (326.3±19.8 nm) intravenously to mice, we detected their presence primarily in isolated Kupffer cells. In vitro, we found that medium sized and small sized (130.5±5.8 nm) EVs were preferentially taken up by Kupffer cells, and liver sinusoidal endothelial cells, respectively. Finally, we demonstrated that in hyperlipidemia, there was a decreased EV uptake both by Kupffer cells and liver sinusoidal endothelial cells.

**Summary/Conclusion**: Our data suggest that hyperlipidema increases the release and reduces the uptake of EVs by liver cells. We also provide evidence for size‐dependent differential EV uptake by the different cell types of the liver.

### PS22.03 Exosomal VEGF‐A derived from hypoxia tubular epithelial cells mediated the injury repair response of peritubular endothelial cell in ischemic kidney injury

Xin Zhong, Institute of Nephrology, Zhongda Hospital, Southeast University School of Medicine, Nanjing, China

**Introduction**: Capillary rarefaction is one of the striking features of post‐acute kidney injury (AKI) kidneys. However, the injury repair responses of the endothelial cells of peritubular capillaries (PTCs) are poorly understood. In this study, proliferation of endothelial cells of PTCs as well as redistribution of VEGF‐A from cytoplasmic to the basolateral part of renal tubular cell in ischemia‐reperfusion injury (IRI) mice was observed.

**Methods**: Ischemia‐reperfusion (I/R) injury mice was established and sacrificed at Day 1, 3, 7. In vitro, exosomes released by HK‐2 were applied to HUVEC or a transwell co‐culture system was conducted. Exosome secretion was inhibited by knockdown of Rab27a in vivo through lentiviral shRNA administration in I/R mice.

**Results**: Interestingly, exosome secretion was increased in hypoxic tubular epithelial cells (TECs) and ischemic kidney, meanwhile significant amount of VEGF‐A protein was secreted as cargoes of exosomes. Further studies showed that exosomes from hypoxic TECs could be internalized by HUVECs and induced proliferation and inflammation response which was significantly attenuated by knockdown of VEGF‐A in the exosome producing cells. Importantly, flow cytometry showed that internalization of exosomes significantly decreased when VEGFR2 was knock down in endothelial cells. Finally, inflammation and proliferation of PTCs were repressed when exosome secretion was inhibited by knockdown of Rab27a in vivo through lentiviral shRNA administration in IRI mice.

**Summary/Conclusion**: Taken together, our studies revealed a novel mechanism of tubule and PTCs cross‐talk via exosomal VEGF‐A which may indicate a critical repair response of peritubular endothelial cell after AKI.

### PS22.04 Exosomes derived from L‐PGDS modified huc‐MSCs restrict cancer progression: A new approach for drug delivery

Benshuai You, jiangsu university

Zixuan Sun, jiangsu university

Can Jin, jiangsu university

Wenrong Xu, Jiangsu University

Jiaxin Zhang, jiangsu university

Hui Qian, jiangsu university

**Introduction**: Exosomes serving as a delivery system have attracted extensive research interest, especially in cancer therapy. Human umbilical cord mesenchymal stem cells (huc‐MSCs)‐derived exosomes are alternative in multiple disease models treatment due to their excellent biological characteristics. In our previous study, lipocalin‐type Prostaglandin D2 Synthase(L‐PGDS) showed inhibition effects on gastric cancer growth. In this study, we aimed to explore whether exosomes overexpressing L‐PGDS could restrict gastric cancer progression.

**Methods**: L‐PGDS‐overexpressing exosomes (EX‐L‐PGDS) were generated through adenovirus encoding L‐PGDS transfected MSCs. Migration, invasion, colony‐forming, and flow cytometry assay were used to detect the effect of exosomes on SGC‐7901 cells in vitro. Nude mice subcutaneous tumor‐bearing model was performed to evaluate the inhibitory effect of EX‐L‐PGDS on tumor growth in vivo.

**Results**: In vitro, EX‐L‐PGDS could be internalized and inhibit the colony‐forming, migration and invasion ability of SGC‐7901 cells. Besides, more apoptotic cells were found after EX‐L‐PGDS treatment. As expected, EX‐L‐PGDS restricted the tumor growth in nude mice subcutaneous tumor‐bearing model. More apoptotic cells and L‐PGDS expression were detected in tumor tissue of EX‐L‐PGDS treatment group. Furthermore, we showed that EX‐L‐PGDS reduced the expression of p‐stat3.

**Summary/Conclusion**: Our results imply that MSCs‐derived exosomes could be used as an effective nano‐vehicle to deliver L‐PGDS. In short, we established an approach for exosomes modification and provided a novel idea for the exosomes‐based cancer therapy.

### PS22.05 Extracellular vesicles derived from human liver stem cells attenuate chronic kidney disease development in an in vivo experimental model of renal ischemia and reperfusion injury

Stefania Bruno, sbruno, Department of Medical Sciences

Massimo Cedrino, Molecular Biothecnology Center

Giulia Chiabotto, Department of Medical Sciences, University of Torino

Elena Ceccotti, Department fo Medical Sciences, University of Torino

Chiara Pasquino, Molecular Biothecnology Center

Stefania Tritta, Molecular Biothecnology Center

Giovanni Camussi, University of Turin

**Introduction**: In order to test the potential therapeutic effect of extracellular vesicles (EVs) derived from human liver stem cells (HLSCs) in acute kidney injury (AKI), with subsequent development of chronic kidney disease (CKD), we set up a model of renal ischemia reperfusion injury (IRI).

**Methods**: EVs were obtained by ultracentrifugation and characterized in accordance with ISEV guidelines. Mice were subjected to 30 minutes IRI and EVs (1 × 109) were iv administered immediately after the surgery and three days after. To evaluate AKI, mice were sacrificed 2 days after the surgery. Renal function (creatinine and BUN plasma levels), histology and molecular analyses of specific markers of AKI (lipocalin‐2/NGAL, kidney injury molecule 1 KIM1) have been evaluated using specific kits, immunohistochemical staining and real time PCR analyses. To evaluate development of CKD, mice were sacrificed 2 months after the injury. Renal function, histology and molecular analyses of specific markers of fibrosis development (alpha‐SMA, collagen I, transforming growth factor beta 1) and of inflammation (TNF‐alpha, IL1‐beta and IL‐6) have been quantified using the same methods described above.

**Results**: In AKI experiments, we found that EV‐treatment induced an amelioration of renal histology (tubular necrosis), an increase in tubular cell proliferation and a slight reduction of lipocalin‐2/NGAL expression, considered as a biomarker of renal proximal tubular injury. In CKD experiments, we found that treatment of IRI‐mice with HLSC‐EVs, limited the development of interstitial fibrosis at histological and molecular levels. Furthermore, the expression levels of pro‐inflammatory genes TNF‐alpha, IL1‐beta and IL‐6 were significantly reduced by EV‐treatment.

**Summary/Conclusion**: Treatment of mice with renal IRI with HLSC‐EVs, induces AKI amelioration and interferes with the development of subsequent interstitial fibrosis in CKD.

### PS22.06 High payloading efficiency and functional intracellular delivery of diverse macromolecules using ARRDC1 mediated microvesicles (ARMMs)

Alysia Bryant, Vesigen Therapeutics

Leah Gens, Vesigen Therapeutics

Lucy Sun, Vesigen Therapeutics

Aditi Jhaveri, Vesigen Therapeutics

Qiyu Wang, Vesigen Therapeutics

Nedyalka Valkov, Vesigen Therapeutics

Kristin Luther, Vesigen Therapeutics

Joseph Nabhan, Vesigen Therapeutics

**Introduction**: Major limitations of large molecule extracellular vesicle (EV) delivery platforms include efficient payloading and retention of payload activity or function upon uptake by recipient cells. Systems with the ability to overcome these hurdles can improve and advance EV‐mediated delivery of therapeutic payloads to yield the desirable pharmacology in target cells. We present data showing payloading efficiency and the corresponding activity of multiple payloads and types of cargo in a class of EVs called ARMMs (Arrestin domain‐containing protein 1 microvesicles).

**Methods**: We engineered ARMMs to be intraluminally loaded with reporters, functional proteins, or large biomolecules. We conducted a comprehensive characterization of ARMMs for payloading efficiency using orthogonal methodologies and investigated payload activity in situ and in vitro upon uptake in recipient cells. To evaluate activity of payloads in recipient cells, treatment was carried out at multiple concentrations of ARMMs followed by characterization of payload activity or function.

**Results**: Efficiencies of payloading of ARMMs with enzymatic or functional proteins, or with novel biomolecules, were markedly higher than those achieved by other EV systems, and the activity of the payloads in ARMMs was not affected by the payloading process. Uptake assays demonstrated fusion of ARMMs with the recipient cells and release of payloads into the cytosolic space without endosomal involvement. Functional measurements of payloads delivered to recipient cells showed unperturbed payload activity.

**Summary/Conclusion**: We provide evidence for a robust approach to payload EVs (∼300 protein molecules/EV) using the ARMMs system with little impact on payload properties. Further, our data show direct cellular uptake of ARMMs as a first step toward efficient functional delivery of therapeutic biomolecules.

### PS22.07 Mammal extracellular vesicles as natural delivery systems for controlled drug delivery applications

Mònica Guarro, Institut Químic de Sarrià (IQS) ‐ Universitat Ramon Llull (URL)

Martí Lecina, Institut Químic de Sarrià (IQS) ‐ Universitat. Ramon Llull (URL)

Salvador Borrós, Institut Químic de Sarrià (IQS) ‐ Universitat. Ramon Llull (URL)

Cristina Fornaguera, Institut Químic de Sarrià (IQS) ‐ Universitat Ramon Llull (URL)

**Introduction**: Exosomes are extracellular vesicles of 50–180nm size excreted mostly in cell stress conditions, which carry nucleic acids inside and contain surface proteins that give them the ability to be reintegrated into the same cell linage for cell‐to‐cell communication. This phenomenon, results in the consideration of exosomes as efficient controlled drug and gene delivery systems for therapeutic purposes in cancer patients, for example.

**Methods**: In the current work, we present our technology for the efficient production, purification, stabilization and loading of mammal extracellular vesicles. To achieve this objective, the efficient isolation and purification of exosomes from model tumor cell cultures through serial high‐speed centrifugation was firstly set up, as well as their further physicochemical characterization (i.e. size and concentration by nanoparticle tracking analysis). Further, we set up a methodology for the extracellular vesicles freeze‐drying, a bottleneck step in most nanoformulations. Finally, we performed a loading and in vitro safety and efficacy study as a proof of concept.

**Results**: We were able to efficiently isolate high amounts of extracellular vesicles, which were characterized and demonstrated to be more than 80% pure, spheric and with sizes below 250 nm. We further demonstrated that they do not loose structural properties after lyophilization (submitted for publication). Next, as a proof of concept, we loaded a fluorescent dye, achieving high rates of encapsulation efficiency. Going a step further, we also demonstrated to be able to gene loading of exosomes with a reporter plasmid has been tested comparing two strategies. On the one hand, a pre‐loading (endogenous loading) of exosome‐donor cells has been achieved by the efficient transfection of exosome‐donor cells with poly(beta aminoester) nanoparticles carrying the gene material. On the other hand, a post‐loading strategy (exogenous loading) has been set up to load previously isolated exosomes with nucleic acids, taking advantage of sucrose gradients. Using loaded‐exosomes, engineered following both strategies, a promising performance in in vitro culture models, in terms of good biocompatibility, transfection efficiency of a reporter gene and cell type specificity has been achieved.

**Summary/Conclusion**: Therefore, it can be concluded that we have a versatile platform for the efficient engineering of exosomes loading to further use them as controlled drug and/or gene delivery systems.

### PS22.08 Mesenchymal Stem Cells‐Derived Extracellular Vesicles, via miR‐210, Improve Infarcted Cardiac Function by Promotion of Neoangiogenesis

na wang, Department of Cardiology, Daping Hospital, Army Medical University, Chongqing, China

**Introduction**: Mesenchymal stem cells (MSCs) hold a great promise for reverse acute myocardial infarction. However, the underlying mechanisms remain controversial. Extracellular vesicles (EVs), secreted from MSCs, play an important role in the process of angiogenesis. Due to the role of microRNAs in the angiogenesis and existence of microRNAs in EVs, we hypothesized that MSC‐derived EVs, via microRNAs, improve infarcted cardiac function by promotion of neoangiogenesis.

**Methods**: Our present study found that MSC‐EVs could be taken by the human umbilical vein endothelial cells (HUVECs), consequently increase the angiogenesis, determined by matrigel plug assay and capillary‐like tube formation assay, by facilitation of HUVEC migration and proliferation. After screening, miR‐210 was found to be one candidate, which was supported by the siRNA study. After down‐regulation of miR‐210 in EVs from MSCs, the MSC‐EVs mediated angiogenesis was reduced. Bioinformatics analysis showed that EFNA3 was the target gene of miR‐210, the EFNA3 expression was decreased when HUVECs were co‐cultured with MSC‐EVs. The above‐mentioned experiments were of pathophysiological significance, because treatment of mice with myocardial infarction with MSC‐EVs, by intravenously injection, improved cardiac function, reduced infarcted area, accompanied with increased neoangiogenesis in infarcted border 4 weeks after cardiac infarction. Knockdown of miR‐210 would remarkably reduce the above‐mentioned protective effect of MSC‐EVs.

**Results**: These results collectively demonstrate that miR‐210 in MSC‐EVs could protect cardiac function by promotion of neoangiogenesis in infarcted heart.

**Summary/Conclusion**: Mesenchymal Stem Cells‐Derived Extracellular Vesicles, via miR‐210, Improve Infarcted Cardiac Function by Promotion of Neoangiogenesis

### PS22.09 Molecular details for non‐cellular elements at wound sites: Interactions between extracellular vesicles and host defense peptides

Priyanka Singh, Research Centre for Natural Sciences

Imola Szigyártó, Research Centre for Natural Sciences

Maria Ricci, Research Centre for Natural Sciences

Mayra Quemé, Research Centre for Natural Sciences

Anikó Gaál, Research Centre for Natural Sciences

Diána Kitka, Research Centre for Natural Sciences

Zoltán Varga, PhD, Research Centre for Natural Sciences, Institute of Materials and Environmental Chemistry

Tamás Beke‐Somfai , Research Centre for Natural Sciences, Budapest

**Introduction**: Host defense peptides (HDPs) are highly membrane active, most often cationic, species exerting not only antimicrobial, antiviral, antifungal and anticancer effects, but also a diverse set of roles in immunomodulation. They are present at high concentrations on infection sites and in both external and intestinal wounds. In this respect, extracellular vesicles (EVs), are highly mobile lipid bilayer bodies, have negatively charged phostphatidylserines in their external leaflet, and have recently also been shown to play important roles as non‐cellular elements in wound healing. However, the latter is a complicated process, where molecular level details are far from understood. Here we demonstrate that red blood cell derived EVs, REVs, and HDPs, selected from e.g. intestines and skin, directly interact with each other, which can be highly beneficial for tissue repair. Proteomics data revealed, that REVs carry numerous molecular elements as part of their adsorbed protein corona, such as keratin and haemoglobin that can be vital during wound healing. The biophysical studies revealed, that membrane active HDPs can remove these protein corona members from REVs, but they can also disrupt the vesicles at higher concentration, allowing spread of both lipid content and also internal elements.

**Methods**: To study the action mechanism of membrane‐active peptides (MAPs) on protein corona we used red blood cell‐derived extracellular vesicles (REV) as a model membrane system. We selected several well‐known MAPs like LL‐37, Buforin IIb, Buforin II, Polybia‐MPI, and Magainin II and their interaction was investigated using several biophysical techniques such as polarized light spectroscopy1, microfluidic resistive pulse sensing measurements, and freeze‐fraction transmission electron microscopy.

**Results**: In line with our previous results on EV‐peptide interactions2, we have sown that the selected HDPs can remove and redistribute REV elements efficiently, by both removing the surface proteins of vesicles at lower concentrations, but also by disrupting the vesicles and forming larger, lamellar bilayer structures, that can be beneficial during tissue repair.

**Summary/Conclusion**: The studies provided molecular level insight to processes during tissue repair and wound healing. The combination of REVs and HDPs may also offer therapeutic strategies for these areas.

### PS22.10 Preconditioned versus non preconditioned Extracellular Vesicles from bone marrow derived mesenchymal stem cells for the treatment of tendinitis ‐ an in vitro study

Robert Soukup, University of Veterinary Medicine, Vienna, Austria

Iris ribitsch, University of Veterinary Medicine, Vienna

Florien Jenner, University of Veterinary Medicine, Vienna

Johannes Grillari, Ludwig Boltzmann Institute for Experimental and Clinical Traumatology

**Introduction**: As stimulation of MSCs (mesenchymal stem cells) influences the contents and biological activities of subsequent MSC‐derived EVs (Extracellular Vesicles), we compared the therapeutic effect of EVs from preconditioned (pc) versus non preconditioned (npc) equine bone marrow (bm) derived MSCs.

**Methods**: Equine bmMSCs and tenocytes (n = 3 biol. replicates) were cultured in serum free media (npc MSC) or chemically inflamed serum free media with 10ng/ml IL1β and 10ng/ml TNFα (pc MSC). EVs were isolated from equine bmMSC conditioned medium by size exclusion chromatography (SEC), characterized via Nano Tracking Analysis (NTA), FACS and Western Blot (WB). 1 × 10^9 particles/ml of autologous pc or npc MSC derived EVs was applied on 400000 inflamed tenocytes/ml.

Control groups consisted out of chemically inflamed tenocytes and healthy tenocytes without any treatment.

RNA‐Sequencing (RNA‐Seq) was performed on EVs and treated tenocytes as well as a wound healing assay. FDR was set at 0.05.

**Results**: EV characterization via WB and FACS proved successful isolation of EVs positive for CD9, CD63 and CD81. NTA showed the desired size range (35‐350nm) of the EVs.

Application of pc as well as npc MSC derived EVs significantly reduced expression of inflammation markers like CXCL6 (‐1.4 and ‐1.6 logFC), CSF3 (‐3.2 and‐3.9 logFC), CXCL8 (‐2.2 and ‐2.2 logFC), CXCL6 (‐1.4 and ‐1.6 logFC), CXCL1 (‐1.2 and ‐1.4 logFC), TNFAIP6 (‐1.4 and ‐1.4 logFC), SOD2 (‐0.85 and ‐0.66 logFC) as compared to untreated control samples.

miRNA‐Seq of the EVs showed significant up‐regulation of miR‐146a (1.4 logFC) and miR‐505 (1.1 logFC) in pc EVs. Both factors are known to reduce immune response.

The wound healing assay showed no significant difference between the two treatments.

**Summary/Conclusion**: The obtained data indicates that the cargo of pc and npc EVs reduce the inflammation in tenocytes and may have an immunomodulatory effect on the recipient cells.

### PS22.11 Small extracellular vesicles of mesenchymal stem cells carring Fibrinogen Like Protein1/Programmed Death Ligand‐1 for suppressing immune rejection in organ transplantation

WU YINGYI, sysu

CHEN Hongbo, SYSU

**Introduction**: Serious side effects generated by the long‐term use of immunosuppressive agents, such as FK506, CsA, necessitate the development of novel immunosuppressive agents in organ transplantation. After comprehensive analysis of samples from clinical kidney transplant patients and allogeneic heart transplantation mice, we found that LAG3 and PD‐1 significantly increased in a series of immune checkpoints, while their corresponding ligands did not accordingly. Therefore, we proposed the concept of "re‐establish immunity tolerance” to regulate the immune balance via mimicking the ligand molecule FGL1/PD‐L1(FP) to combine with LAG3/PD‐1 to enhance the immunosuppressive signal.

**Methods**: First, the mesenchymal stem cells were modified to obtain the dual‐target exosomes, and then the characterization of exosomes and the analysis of their inclusiveness were carried out.Its targeting function and inhibition of T cell proliferation were verified in vitro, and the mechanism was further explored.The function of immune suppression in vivo was further verified in allogeneic heart transplantation mice model.

**Results**: Our research found that the modified FP exosomes can effectively inhibit the proliferation of T cells and effectively prolong the survival time of allograft mouse hearts. Effectively inhibit the expression of CD8+ T cells in the heart and spleen and promote the proliferation of CD4+CD25+Foxp3+ Treg cells. FP EVs encapsulated with FK506 can effectively prolong the survival time of transplanted hearts and improve the toxic and side effects of FK506. Therefore, dual‐target FP EVs combined with low‐dose FK506 may become an effective treatment for organ transplant rejection.

**Summary/Conclusion**: Enrichment of FGL1/PD‐L1 on exosomes to rebuild the immune tolerance can be served as an effective strategy to inhibit excessive immune rejection after organ transplantation, implying remarkable function of immune checkpoints in excessive immunity. Furthermore, MSC‐derived exosomes have unique advantages and thus become a promising biomaterial. Our innovative achievements not only provide an effective approach to suppress excessive immune rejection generated from organ transplantation, but open up prologue of immune checkpoint applying in immunosuppression.

### PS22.12 P53 loaded natural nanovesicles of xenogenic origin as highly effective oncology therapy demonstrated in syngeneic murine model

Alex Tendler, ExoProTher Medical Ltd

Lana Volokh, ExoProTher Medical Ltd

Evleen Galouk, Bar Ilan university

Yevgeny Tendler, ExoProTher Medical Ltd

**Introduction**: TP53 mutation is a hallmark of a majority of cancer cases. It is a natural target for cancer therapy. Unfortunately, multiple attempts to introduce exogenous p53 to restore its activity in cancer cells and induce their apoptosis have been failed.

The main challenges are:

1. Efficient delivery of p53‐expressing construct to cancer cells.
2. Inhibition of WT‐p53 via the dominant negative effect of mutant‐p53

It has been only recently discovered by us that corneal epithelial cells actively secrete nanovesicles containing p53 into the extracellular space. They are re‐captured by neighboring epithelial cells and play role in local corneal anti‐cancer defense. Being an immune‐privileged tissue, corneal epithelium lacks mechanisms of innate and adaptive immunity and has to fully rely on local anti‐oncogenic functions. We established a novel therapeutic approach based on this principle.

**Methods**: We use nanovesicles containing WT‐p53 harvested from corneal epithelial cells. In order to prevent inhibition of exogenous WT protein via the dominant negative effect of mutant‐p53, we use non‐human p53 whose C‐terminal domain responsible for oligomerization is different. This way exogenous WT‐p53 is not suppressed even at high concentrations of mutant‐p53.

**Results**: Our nanovesicles obtained from chicken corneal epithelial cells have average size of 150 nm. We demonstrate their anti‐cancer activity in‐vitro in multiple malignant cell lines. Cell lines with mutant‐p53 are significantly more sensitive to our treatment than those with WT‐p53. Moreover, UV irradiation of corneal epithelial cells significantly increased anti‐cancer effect. Using mice syngeneic model, we show that our nanovesicles injected systemically, significantly prolong survival, reduce metastasis and tumor volume with no adverse effects.

**Summary/Conclusion**: Translation of ancient local anti‐oncogenic defense mechanism to modern systemic anti‐cancer treatment has been demonstrated.

## PS23 Biogenesis and Subtypes of EVs

Chair: Carolina Soekmadji, QIMR Berghofer Medical Research Institute, Australia

Chair: Migara Jayasinghe, Department of Pharmacology, Yong Loo Lin School of Medicine, National University of Singapore, Singapore

### PS23.01 Flow Cytometry based approach to study the stress induced modulation of Bacterial Membrane vesicles

Parul Shishpal, ICMR‐ National Institute for Research in Reproductive Health

Vainav Patel, ICMR‐ National Institute for Research in Reproductive Health

Vikrant Bhor, ICMR‐ National Institute for Research in Reproductive Health

**Introduction**: Pathogenic bacteria are exposed to numerous factors such as commensals and other competing bacteria, antibiotics as well as the hostile environment of host which threaten their survival. To thrive under these conditions, pathogens have devised several offense and defence mechanisms, including the release of membrane vesicles (MVs). However, the sub‐micron size of MVs as well as the complex and variable composition makes their analysis challenging. We applied flow cytometry approach to study the stress induced modulation of bacterial membrane vesicles from Gardnerella vaginalis, an anaerobe predominantly associated with bacterial vaginosis (BV) a common reproductive tract infection.

**Methods**: FITC labelled submicron beads were used to define gates to analyze dual fluorescence labelled (protein and lipid) MVs isolated under stressed and non‐stressed condition.

**Results**: Comparative analysis of MVs revealed altered biogenesis of MVs under pH stress compared to non‐stressed conditions, indicated by quantitative differences in MVs of varying sizes as well as presence of two different sub‐populations of similar sized MVs which differed in protein and lipid content.

**Summary/Conclusion**: These findings indicate that flow cytometric analysis can supplement existing approaches including proteomics to evaluate stress induced modulation of MVs in a variety of conditions.

### PS23.02 Protein N‐glycosylation modulates the secretion of small extracellular vesicles

Kazuki Nakajima, Fujita Health University

Naoyuki Taniguchi, Osaka International Cancer Institute

Yoichiro Harada, Osaka International Cancer Institute

**Introduction**: Cells secrete various types of small extracellular vesicles (sEVs), including exosomes and microvesicles. sEVs are known to be enriched with glycoproteins that are modified with asparagine‐linked glycans (N‐glycans), yet little is known about whether N‐glycans play a role in the biogenesis of sEVs and their secretion.

**Methods**: Human amelanotic melanoma A375, human breast cancer MDA‐MB‐231‐luc‐D3H2LN (D3H2LN), mouse melanoma B16‐F10, mouse mammary gland cancer 4T1, mouse neuroblastoma Neuro‐2a, and African green monkey kidney fibroblast‐like COS‐7 were treated with or without N‐glycosylation inhibitor 1 (NGI‐1) in media containing exosome‐free fetal bovine serum. The cells were removed from the conditioned media by low speed centrifugation and the supernatants were subjected to sequential centrifugation at 10,000 ‐ g for 20 min, followed by 100,000 ‐ g for 70 min. The pellets, which contain sEVs, were washed once and the phospholipid contents were analyzed by focused lipidomics.

**Results**: NGI‐1 treatment of B16‐F10 dramatically decreased the amounts of phosphatidylcholine (PC), phosphatidylethanol amine (PE), phosphatidylserine (PS) and shingomyelin (SM) of sEVs. Proteomics analysis of B16‐F10‐derived sEVs suggested that NGI‐1 suppressed the secretion of sEVs whose protein contents were dissimilar to those of exosomes and microvesicles. In contrast, NGI‐1 treatment of Neuro‐2a increased the amount of PC, PE, PS and SM of sEVs. Surprisingly, the NGI‐1‐treated Neuro‐2a secreted a large amount of Cd81. NGI‐1 treatment of the other 4 cell lines showed moderate effects on the phospholipid contents of sEVs.

**Summary/Conclusion**: Our findings raise the possibility that N‐glycosylation‐dependent regulation of the biogenesis/secretion of sEVs operates in several cell lines but the dependency and mode of regulation differ between these cell lines.

### PS23.03 Endoplasmic Reticulum Membrane Contact Sites tune exosome secretion by modulating multivesicular body maturation and transport

Frederik J. Verweij, IPNP INSERM U1266

Maarten Bebelman, MPI‐CBG

Roberta palmulli, University of Cambridge

Anaïs Bécot, IPNP INSERM U1266

Mickael Couty, IPNP INSERM U1266

Xavier Heiligenstein, Institut Curie

Graça Raposo, PhD, Institut Curie, CNRS UMR144, Structure et Compartiments Membranaires, Université Paris Sciences et Lettres, Paris, France. Institut Curie, CNRS UMR144, Plateforme d'imagerie cellulaire et tissulaire (PICT‐IBiSA), Université Paris Sciences et Lettres, Pari

Michiel Pegtel, Amsterdam UMC ‐ Cancer Center Amsterdam

Guillaume Van Niel, PhD, IPNP INSERM U1266

**Introduction**: The secretion of exosomes results from the fusion of multivesicular compartments with the plasma membrane. Yet the precise nature of these compartments and the molecular mechanisms leading to this final secretory step remain ill‐defined.

**Methods**: Using a CD63‐based quantitative dual‐color TIRF live‐imaging approach, we characterized the compartments secreting exosomes.

**Results**: We show that exosomes are released from a subclass of non‐proteolytic, late‐endosomal multivesicular bodies (LE/MVB) that undergo a GTPase cascade, from Rab7 to Rab27, to fuse with the plasma membrane (PM). We identified Membrane Contact Sites (MCS) existing between endoplasmic reticulum (ER) and LE/MVB as key regulators of this cascade leading to exosome secretion. LE/MVB can form various ER MCS, depending on distinct tethering partners such as ORP1L and Protrudin. We show that each tethering partner differently modulates MVB/PM fusion probability and subsequent exosome release by acting on LE/MVB motility and maturation.

**Summary/Conclusion**: Altogether, our results demonstrate that exosome secretion is the result of a complex, multi‐step process that primes LE/MVB for secretion, highlighting the ER as a new player in exosome‐mediated intercellular communication.

### PS23.04 The effect of Wnt activity on extracellular vesicle release in pancreatic ductal adenocarcinoma

András Áron Á Soós, Semmelweis University, Department of Genetics, Cell‐ and Immunobiology

Anikó Zeöld, Semmelweis University, Department of Genetics, Cell‐ and Immunobiology, Molecular Cancer Biology Research Group

Zoltán Wiener, Semmelweis University, Department of Genetics, Cell and Immunobiology, Budapest, Hungary

**Introduction**: Pancreatic ductal adenocarcinoma (PDAC) is one of the deadliest cancers (5‐year survival rate is less than 8%). PDAC cells can harbour various mutations (e.g. KRAS, TP53) that leads to intratumoral cellular heterogeneity and it also makes therapy difficult. Patient‐derived organoids maintain both the genetic and cellular heterogeneity of the original tissues and have proven to be one of the best models of human cancers. A recently established PDAC organoid library highlighted the importance of Wnt proteins produced by tumor or stromal cells. Although extracellular vesicles (EV) hold a great promise for early cancer diagnostics, however, factors modifying their release are not well known. Since we found that increased Wnt activation and cell proliferation resulted in a higher EV secretion in colorectal cancer (CRC), here we tested this mechanism in PDAC.

**Methods**: We cultured PDAC patient‐derived organoids in Matrigel as 3D matrix. The Medical Research Council of Hungary approved the experiments with human samples and informed consent was obtained from patients. We detected gene expression changes with RT‐qPCR, whole‐mount immunostaining and flow cytometry. EVs were detected with Nanoparticle Tracking Analysis (NTA).

**Results**: All of our PDAC organoid lines were independent of external Wnt proteins and they expressed PORCN, an enzyme necessary for Wnt secretion. Organoids expressed epithelial Wnt genes, such as Wnt7a, Wnt10a, Wnt11, but we could not detect the stromal Wnt5a. PORCN inhibitors resulted in a decreased expression of the Wnt target genes AXIN2, LGR5 and TROY in organoids, suggesting the responsiveness of PDAC cells for Wnt proteins. Surprisingly, we found no change in the percentage of KI67+ proliferating cells and EV secretion when blocking Wnt secretion. In addition, organoid cells sorted for different expression level of CD44 and CD133, two Wnt target genes, did not maintain this differential expression pattern in cultures.

**Summary/Conclusion**: Unlike CRC, we found that Wnt activity, cell proliferation and EV release are uncoupled in PDAC that can be explained by the different typical mutational spectrum of the two tumor types. Furthermore, we found a high placticity of PDAC tumor cells in their CD44 and CD133 expression patterns.

### PS23.05 Proposed ancient evolutionary origin of exosome EVs that provide unappreciated carrier effects to assist transfers of their miRNA to targeted cells

Phil W. Askenase, yale university school of medicine

**Introduction**: We postulate that some subsets of current day exosome EVs may be descendants of ancient Protocells proposed as the earliest living forms arising near to the origin of life.

**Methods**: The Protocells are formed in the noxious primordial seas consisting of lipid bubbles evolving into bilamellar membrane bound vesicles able to resist noxious conditions and come to retain spontaneously formed RNA polymers that evolve into self‐replenishing enzymatic ribozyme polyribonucleotides.

**Results**: Properties of present day “activated exosomes” that seem related to the ancient Protocells include ability to survive noxious conditions like strong acidity combined with digestive enzymes in the stomach and perhaps in intracellular phagolysosomes. This permits oral administration of such exosome EVs in mothers’ milk to guide development of organs and systems in neonatal recipients as well as optimal function in hypoxic environments like at necrotic sites of growing tumors or tissue injury. Additionally, there is survival of such modern EVs for optimal oral administration of systemically immunosuppressive dual antigen and gene specific T and B cell‐derived immunoregulatory exosomes. Particularly, the unusual membrane lipid composition of mediating such resistance allows surface binding of antibody free light chains rendering such exosomes antigen‐specific in cell targeting and also able to associate with selected miRNAs generating a simultaneous dual antigen and gene specificity for mediating epigenetic effects in recipient acceptor cells.The unusual ability of such exosomes to transfer minute femtomolar quantities of miRNAs is beyond conventional concepts based on non‐physiological mere addition of miRNAs alone or alternatively further non‐quantitative genetic expression

**Summary/Conclusion**: Non‐canonical minute miRNA transfers are proposed due to both exosome carrier‐delivering and carrier‐acting properties. These carrier effects include surface activation of acceptor cells and three quantitative concentrating effects. These are exosome carriage in minute attoliter volumes substantially raising molar concentrations, transfers that actually are due to only an extreme minority of the applied vesicles and effects on only a small minority of the total targeted cells.

### PS23.06 Utility of extracellular vesicles as a biological indicator of resilience

Meaghan E. Beckner, MS, University of Pittsburgh

William R. Conkright, MS, RD, CSSD, CSCS, University of Pittsburgh

Qi Mi, PhD, Neuromuscular Research Lab / Warrior Human Performance Research Center, University of Pittsburgh, Pittsburgh, PA, USA

Amrita Sahu, PhD, McGowan Institute for Regenerative Medicine, University of Pittsburgh, Pittsburgh, PA, USA

Zachary J. Clemens, MS, McGowan Institute for Regenerative Medicine, University of Pittsburgh, Pittsburgh, PA, USA

Brian J. Martin, PhD, Neuromuscular Research Laboratory / Warrior Human Performance Research Center, University of Pittsburgh, Pittsburgh, PA, USA

Shawn D. Flanagan, PhD, Neuromuscular Research Lab / Warrior Human Performance Research Center, University of Pittsburgh, Pittsburgh, PA, USA

Fabrisia Ambrosio, PhD, McGowan Institute for Regenerative Medicine, University of Pittsburgh, Pittsburgh, PA, USA

Bradley C. Nindl, PhD, Neuromuscular Research Lab / Warrior Human Performance Research Center, University of Pittsburgh, Pittsburgh, PA, USA

**Introduction**: Resilience is described as the capacity to overcome stress and adversity while maintaining normal physiological and psychological functioning. Objective quantification of resilience is challenging as the current standard is self‐report questionnaires. Indeed, circulating free‐floating neuroendocrine biomarkers have been examined as potential indicators of resilience, however, extracellular vesicles (EVs) have not yet been explored as means to reflect this resilience. As such, apoptotic bodies (ABs), a subpopulation of EVs that are formed only during cell death, may serve as an important early indicator of cellular stress. Hence, we hypothesized that ABs would be less prevalent in high resilient individuals.

**Methods**: Nineteen men (27.5 ± 5.9 years, 177.0 ± 6.7 cm, 86.9 ± 17.9 kg, 22.2 ± 6.9 % body fat) completed the Connor Davidson Resilience questionnaire and were exposed to daily rigorous exercise accompanied by 48‐hr of sleep and caloric restriction. Informed consent was obtained from all subjects, and study was approved by the Institutional Review Board. Oxytocin, NPY, IGF‐I, BDNF, and klotho were analyzed using ELISAs. EV concentration and cargoes were characterized using nanoparticle tracking analysis and imaging flow cytometry, respectively. EVs were stained for THSD1 as a marker of ABs. EV structural features were extracted and EVs were stratified by area (small < 0.031; medium 0.031 " 0.785; large > 0.785 μm2), then subsequently analyzed using two‐way mixed ANOVA with Sidak correction to determine differences between high (H‐RES) and low (L‐RES) resilient individuals.

**Results**: Total EV concentration decreased 51% (p < 0.001, η_p^2 = 0.581) from baseline to peak stress, with no significant difference between H‐RES and L‐RES. In contrast, intensity and median pixel of medium sized THSD1+ events increased from baseline to peak stress (p = 0.027 and p = 0.002, respectively). Moreover, these features were significantly greater among L‐RES compared to H‐RES (intensity: 15.9 vs. 7.5, p = 0.010, η_p^2 = 0.328; median pixel: 0.191 vs. 0.098, p = 0.007, η_p^2 = 0.358), indicating greater AB genesis in the L‐RES group during stress. None of the neuroendocrine biomarkers were significantly different between the two groups.

**Summary/Conclusion**: EVs may provide a novel approach to characterizing biological indicators of resilience, with greater sensitivity than neuroendocrine biomarkers. This preliminary analysis suggest low resilient individuals may be more susceptible to stress at a cellular level.

### PS23.07 Caveolin‐1‐overexpression induces deep plasma membrane alteration and secretion of extracellular vesicles in a model of rhabdomyosarcoma

Serena Maggio, University of Urbino

Emanuela Polidori, Department of Biomolecular Sciences, University of Urbino Carlo Bo, Urbino, Italy.

Paola Ceccaroli, Department of Biomolecular Sciences, University of Urbino Carlo Bo, Urbino, Italy.

Laura Graciotti, Department of Clinical and Molecular Sciences, Università Politecnica delle Marche, Ancona, Italy.

Andrea Cioccoloni, Department of Biomolecular Sciences, University of Urbino Carlo Bo, Urbino, Italy.

Michela Battistelli, Department of Biomolecular Sciences, University of Urbino Carlo Bo, Urbino, Italy.

Silvia Codenotti, Department of Molecular and Translational Medicine, University of Brescia, Brescia, Italy.

Gabriella Pocsfalvi, EVs&MS Laboratory, Institute of Biosciences and BioResources, National Research Council of Italy

Fabiola Olivieri, Department of Clinical and Molecular Sciences, Università Politecnica delle Marche, Ancona, Italy.

Massimiliano Bonafè,Department of Experimental, Diagnostic, and Specialty Medicine, University of Bologna, Bologna, Italy.

Alessandro Fanzani, Department of Molecular and Translational Medicine, University of Brescia, Brescia, Italy.

Michele Guescini, University of Urbino Carlo Bo

**Introduction**: Caveolin‐1 (CAV1) is an integral membrane protein required to generate caveolae and cholesterol‐enriched lipid rafts of the plasma membrane. Moreover, CAV1 binds to many other proteins, controls cholesterol homeostasis, and regulates various cell functions. CAV1 has a controversial role in cancer, it is widely accepted that loss of CAV1 correlates with early‐stage tumor progression, while its re‐expression and phosphorylation are associated with recurrence and metastatic disease. For example, it has been shown that CAV1 cooperates to tumor growth and metastatic potential in rhabdomyosarcoma (RD).

A considerable body of evidence suggests the possibility that extracellular CAV1 may be relevant in cancer cell metastasis.

The present work aims to investigate if the increased aggressiveness of RD cells overexpressing CAV‐1 correlates with an altered extracellular vesicle release.

**Methods**: Vesicles were isolated by differential ultracentrifugation and density gradient separation methods from conditioned media of control and metastatic RD lines overexpressing CAV1 (RD‐CAV1). Collected small (sEVs) and large (lEVs) extracellular vesicles were characterized by Nanoparticle Tracking Analysis (NTA), transmission electron microscopy (TEM) and western blot analysis.

**Results**: Scanning and transmission electron micrograph analysis of RD cells showed that the overexpression of CAV1 induced deep plasma membrane reorganization demonstrated by the development of a great number of membrane protrusions emerging from the cell surface of RD‐CAV1 compared to RD‐Control cells. Furthermore, TEM analysis revealed the presence in RD‐CAV1 of higher quantities of intracellular organelles, resembling multivesicular bodies, compared with RD‐Ctrl cells. Physical characterization with Nanosight technology and transmission electron microscopy showed that the applied separation protocols allowed the isolation of two EV subpopulations of about 93 nm (sEVs) and 170 nm (lEVs) in diameter, respectively. Quantification data showed that RD‐CAV1 releases 3–4 fold more EVs compared to RD‐Ctrl; furthermore, NTA revealed that CAV1 overexpression seems not to affect the sEV versus lEV production ratio with sEVs about 10 times more abundant than lEVs.

Analysis of key vesicular markers' protein expression by western blotting confirmed the efficient separation of supernatant‐derived EV sub‐populations: sEVs were positive for the exosomal markers Alix, Flot‐1, Syntenin‐1, and TSG101; whereas lEVs were positive for the endoplasmic reticulum marker calnexin.

**Summary/Conclusion**: The reported data suggest that, in addition to the well‐established structural role, CAV1 could be a key regulatory factor promoting tumor microenvironment remodelling by modulating EV release.

### PS23.08 Evaluation of culture conditions on the presence of Ago2 and miRNAs in small extracellular vesicles

Lizandra Jimenez, Vanderbilt University

Bahnisikha Barman, Vanderbilt University

Roxanne Pelletier, Harvard University Medical School

Alissa Weaver, Department of Cell and Developmental Biology, Vanderbilt University School of Medicine

**Introduction**: Argonaute 2 (Ago2) is the essential component of the RNA‐Induced Silencing Complex (RISC) that binds miRNAs and promotes mRNA degradation. Extracellular vesicle (EV)‐carried miRNAs have been shown to influence gene expression and functional phenotypes in recipient cells. While Ago2 has been shown to be sorted into EVs and to affect sorting of miRNAs, some groups have reported extensive contamination of EV preparations with non‐vesicular Ago2 and miRNAs. We set out to evaluate the effect of growth factor signaling and serum contamination on the detection of Ago2 in SEVs.

**Methods**: Wildtype KRAS colorectal cancer cells, DKs‐8, were conditioned with three different culture media (Serum‐free DMEM, EV‐depleted FBS in DMEM, and Opti‐MEM). EVs were purified from conditioned media by cushion‐density gradient ultracentrifugation. Western blot analysis of DKs‐8 total cell lysates, large EVs and density gradient fractions was performed, probing for Ago2 and EV marker proteins. The size and concentration of the EVs were determined by nanoparticle tracking analysis. To assess whether selected miRNAs are on the inside or outside of SEVs, a RNase protection assay in the absence or presence of Triton X‐100 was performed on the SEVs, followed by RNA extraction and qRT‐PCR for miRNAs.

**Results**: In all conditions, we found the highest abundance of SEVs in fractions 6 and 7, as assessed by Western blot analysis. Ago2 was detected in the same fractions as SEVs in both the Serum‐free DMEM and Opti‐MEM conditions. In contrast, Ago2 was present in both vesicular and non‐vesicular fractions in the EV‐depleted FBS in DMEM condition. No significant differences were observed in the size and number of EVs collected in the three conditioning methods. The selected miRNAs are on the inside of DKs‐8 Serum‐free DMEM and Opti‐MEM SEVs, as they only were sensitive to the RNase treatment in the presence of Triton X‐100. In contrast, the miRNAs appear to be predominantly on the outside of SEVs in the EV dep. FBS condition, as the RNase treatment in the absence of Triton X‐100 abolished the detection of select miRNAs.

**Summary/Conclusion**: Multiple factors may affect the ability to detect vesicular Ago2 and miRNAs, including serum in the conditioned media that may provide large amounts of contaminating extravesicular Ago2 and miRNAs.

### PS23.09 Human neoplastic mast cells secrete distinct subpopulations of KIT receptor‐containing extracellular vesicles

Annika Pfeiffer, National Institutes of Health (NIH)

Jennifer D. D. Petersen, PhD, Section on Integrative Biophysics, Division of Basic and Translational Biophysics, Eunice‐Kennedy‐Shriver National Institute of Child Health and Human Development, National Institutes of Health, Bethesda, Maryland, USA

Joshua Zimmerberg, Section on Integrative Biophysics, Division of Basic and Translational Biophysics, Eunice‐Kennedy‐Shriver National Institute of Child Health and Human Development, National Institutes of Health, Bethesda, Maryland, USA

Dean D. Metcalfe, Mast Cell Biology Section, Laboratory of Allergic Diseases, National Institute of Allergy and Infectious Diseases, National Institutes of Health, Bethesda, Maryland, USA

Ana Olivera, Mast Cell Biology Section, Laboratory of Allergic Diseases, National Institute of Allergy and Infectious Diseases, National Institutes of Health, Bethesda, Maryland, USA

**Introduction**: Activating gene mutations in the transmembrane receptor KIT can lead to the dysregulated proliferation of human mast cells. The resulting neoplastic mast cells constitutively secrete high quantities of extracellular vesicles (EVs), which can transfer KIT and nucleic acids into target cells affecting their function. Despite their potential contributions to disease pathology, KIT‐containing EVs secreted by mast cells have not been thoroughly characterized.

**Methods**: EVs released into the culture supernatant by the human neoplastic mast cell line HMC‐1.1 were isolated by polymer precipitation or differential ultracentrifugation and concentration and size were measured by nanoparticle tracking. EV subpopulations were separated by immunoaffinity capture using canonical EV tetraspanins or KIT. Subpopulations were characterized by immunoblots and density gradients. KIT‐containing EVs were also analyzed by mass spectrometry and morphology was assessed by electron microscopy using KIT‐affinity grids.

**Results**: EVs shed by HMC‐1.1 cells contain KIT and canonical EV markers. We verified the topology of KIT within EV membranes and demonstrated immunoaffinity capture can successfully isolate KIT‐EV populations. Characterizations of the subpopulations revealed that KIT‐EVs contain exosome‐ and microvesicle‐like protein markers and a distinct protein profile from KIT‐depleted EVs. Further analysis showed KIT‐EVs can be separated by ultracentrifugation into P15 and P120 EV subpopulations that are enriched with microvesicle and multivesicular body/exosome markers, respectively.

**Summary/Conclusion**: Neoplastic mast cells secrete KIT‐EVs as heterogenous microvesicle‐ and exosome‐like subpopulations, suggesting these subgroups could have different biological functions or targets. Isolating KIT‐EVs by immunoaffinity will aid in investigating the possibility of enriching in vivo mast cell EVs within complex biological samples, with the focus on developing diagnostic biomarkers.

### PS23.10 Universal extracellular vesicle marker candidates reveal vesicle size‐ and tetraspanin expression‐dependent differences in staining efficiency and profile

George Daaboul, MDPhD, NanoView Biosciences

Dennis Zimmermann, NanoView Biosciences

**Introduction**: Nanoview Biosciences’ ExoViewer platform currently enables the user to capture extracellular vesicles (EV) by means of surface antigen‐specific antibodies (e.g. targeting tetraspanins), making possible the enumeration of individual particles using single‐particle interferometric as well as fluorescence imaging. Currently, interferometric imaging is limited to particles larger than 50 nm, while fluorescently stained EV smaller than 50 nm can be well resolved. Further, it is conceivable that small EV contain antigen numbers in the single digits, making antigen‐specific immunostaining a challenge. To further characterize EV populations of different sizes irrespective of surface marker composition, the next step will be to target the vesicular nature of the detected particles linked to a fluorescence readout.

In a large‐panel screening effort designed to identify a fluorescent probe that is ubiquitously distributed across the surface (or lumen) of EV and at the same time complementary to canonical EV surface markers, we have revealed yet unknown staining characteristics of two entirely distinct popular fluorescence staining probes, namely Annexin‐V and CFDA‐SE (herein referred to as CFSE).

**Methods**: CFSE and Annexin‐V have been widely used in cell biology to monitor cell proliferation and cell viability, respectively, and only more recently have been applied to stain extracellular vesicles mostly in combination with flow cytometry‐based applications. While CFSE staining relies on the passive diffusion across the lipid bilayer into the vesicle lumen and its subsequent esterase‐dependent activation and coupling to luminal proteins, in the presence of Ca2+ Annexin‐V binds with high affinity to phosphatidylserine in the outer lipid bilayer leaflet of many EV as has been recently shown.

**Results**: Here, we are highlighting two key observations: (1) CFSE staining efficiency depends on the enrichment of certain Tetraspanins over others, while Annexin‐V staining shows a largely homogenous staining pattern across different EV sub‐populations of the same sample. (2) For vesicles larger than 50 nm depending on the sample type Annexin‐V and CFSE stain vesicles with efficiencies as high as 90–97%, respectively. However, for vesicles smaller than 50 nm only 50–70% of the detected vesicles are Annexin‐V‐ or CFSE‐positive.

**Summary/Conclusion**: This is suggesting that these dyes are well‐suited to report larger particles while further optimization will be required to enable reliable detection of EV smaller than 50 nm.

### PS23.11 SOX10 downregulation in tumor cells affects extracellular vesicle production and function in the tumor microenvironment

Peng Yuan, German Cancer Research Center

Ka‐Hou Man, German Cancer Research Center

Magdalena Schlotter, German Cancer Research Center

Yonghe Wu, German Cancer Research Center

Bernhard Radlwimmer, German Cancer Research Center

**Introduction**: SOX10 is a transcription factor that is highly expressed in the majority of melanomas and a subtype of glioblastoma. Previously, we showed that knockdown of SOX10 induces increased cytokine and chemokine production and infiltration of tumors by macrophages in glioblastoma cell line and syngraft models. Extracellular vesicles (EVs) may modulate the function of myeloid cells in tumor microenvironment; however, it is unknown whether downregulating SOX10 can affect the production or function of EVs in tumors. In our study, we investigated the impact of SOX10 knockdown (KD) on EVs production and function in melanoma and glioblastoma cell lines.

**Methods**: We used short hairpin RNA to knock down SOX10 in tumor cells. Ultracentrifugation was used to separate EVs from conditioned media (CM) and the sizes and quantities of EVs were determined by Nanosight and MicroBCA assay. RT‐qPCR was used to detect the gene expression changes of pro‐inflammatory genes in macrophages after EVs or CM treatment. In addition, the effect of CM from EV‐treated macrophages on tumor cell migration was characterized by transwell‐migration assay. Finally, Toll‐like receptor 8 (TLR8) inhibitor CU‐CPT8M was used to investigate the mechanism of the phenotypic transition in macrophages after EV treatment.

**Results**: SOX10 KD enhanced the production of exosome‐like EVs in melanoma cell lines A375 and HT144 and glioblastoma cell line LN229. CM from HT144‐SOX10‐KD cells induced stronger upregulation of CXCL8, CCL4 and PD‐L1 in macrophages at mRNA level than control CM. Importantly, depletion of EVs from CM of HT144‐SOX10‐ KD cells reduced the expression level of these pro‐inflammatory genes relative to CM from control cells. Similarly, purified EVs from SOX10 KD cell lines also showed a stronger effect on inducing pro‐inflammatory phenotypes on macrophages, suggesting that EVs from SOX10 KD cells play an important role in macrophages phenotypic transition. Furthermore, CM of SOX10 KD EVs treated macrophages promoted the migration of melanoma cells. At last, we found that TLR8 inhibitor CU‐CPT8M could inhibit the pro‐inflammatory phenotype of macrophages induced by SOX10 KD EVs.

**Summary/Conclusion**: SOX10 KD enhanced EV production in melanoma and glioblastoma cells. SOX10 KD EVs induced pro‐inflammatory gene expression in human macrophages and the CM of these macrophages augmented the migration of tumor cells. Targeting TLR8 pathway may inhibit the phenotypes induced by SOX10 KD EVs.

### PS23.12 The Necroptosis Effector MLKL drives Small Extracellular Vesicle Release and Tumour Growth in Glioblastoma

Gwennan ANDRE‐GREGOIRE, CRCINA UMR1232 and Integrated Center for Cancerology (ICO)

Tiphaine DOUANNE, CRCINA UMR1232

An THYS, CRCINA UMR1232

Clément MAGHE, CRCINA UMR1232

Kathryn JACOBS, CRCINA UMR1232

Cyndie BALLU, CRCINA UMR1232

Kilian TRILLET, CRCINA UMR1232

Ignacio BUSNELLI, INSERM UMR_S 1109

Vincent Hyenne, INSERM / CNRS

Nicolas BIDERE,CRCINA UMR1232

Julie Gavard, INSERM, CNRS, Université de Nantes, Institut de Cancérologie de l'Ouest

**Introduction**: Extracellular vesicles (EVs) play key roles in tumour progression, notably in glioblastoma in which the subpopulation of Glioblastoma Stem‐like Cells (GSCs) might represent a meaningful source of tumour‐derived EVs. However, the mechanisms involved in the production and release of EVs by glioblastoma cells are still poorly understood. Here, we report the identification of MLKL, a crucial effector of cell death by necroptosis, as a regulator of the constitutive secretion of small EVs from GSCs.

**Methods**: Human public databases for EV biogenesis proteins were interrogated. The functional role of MLKL was assessed by either siRNA, inhibitor and direct “protein knock‐down”, so called trim‐away in vitro. In vivo, GBM model of nude mice implanted with patient‐derived GSC were treated with MLKL inhibitor NSA.

**Results**: The targeting of MLKL by genetic, protein depletion or chemical approaches alters endosomal trafficking and EV release and reduces GSC expansion in vitro. This function ascribed to MLKL appears independent of its role during necroptosis. In vivo, pharmacological inhibition of MLKL triggers a reduction of both the tumour burden in xenografted mice and of the level of plasmatic EVs.

**Summary/Conclusion**: This work reinforces the idea of a non‐deadly role for MLKL in endosomal trafficking and suggests that interfering with EV biogenesis is a promising therapeutic option to sensitise glioblastoma cells to death.

### PS23.13 Deciphering the role of neutral sphingomyelinase 2 dependent extracellular vesicle production in systemic inflammation

Marlies Burgelman, VIB ‐ Ghent University

Charysse Vandendriessche, VIB ‐ Ghent University

Roosmarijn E. Vandenbroucke, VIB ‐ Ghent University

**Introduction**: Previously, we showed that extracellular vesicle (EV) production by choroid plexus epithelial (CPE) cells at the interface between blood and cerebrospinal fluid (CSF) plays an important role in a lipopolysaccharide (LPS‐) induced mouse model of systemic inflammation. Interestingly, interfering with EV biogenesis via inhibition of neutral sphingomyelinase 2 (nSMase2) altered EV release into the CSF, pointing towards a role for nSMase2 mediated EV‐release in systemic inflammation (Balusu et al., EMBO Mol Med, 2016).

**Methods**: Recently, our lab generated nSMase2 fl/fl mice via CRISPR/Cas9 technology to develop cell‐ and tissue‐specific knock‐out mice for nSMase2, assuming that these mice will produce less EVs in the targeted cells or tissues.

**Results**: Our experiments revealed that the nSMase2 fl/fl LysM Cre mice, in which nSMase2 is deleted in the myeloid blood cells, show protection against LPS‐induced systemic inflammation. On the contrary, we did not observe such protection in the cecal ligation and puncture model for sepsis.

**Summary/Conclusion**: Myeloid cell specific nSMase 2 deficient mice are protected against LPS‐ but not CLP‐ induced lethality. Further research is ongoing to unravel the role of myeloid cell‐derived EVs in systemic inflammation and to explore the importance of nSMase2‐dependent EV release from other sources.

## PS24 Separation and Concentration II

Chair: Meadhbh Brennan, NUIG, Galway, Ireland

Chair: Steffi Bosch, IECM, ONIRIS, INRAE, USC1383, Nantes, France

### PS24.01 Characterisation and comparison of two isolation methods of Extracellular Vesicels from bone marrow derived equine Mesenchymal Stem Cells

Robert Soukup, University of Veterinary Medicine, Vienna, Austria

Iris ribitsch, University of Veterinary Medicine, Vienna

Johannes Grillari, Ludwig Boltzmann Institute for Experimental and Clinical Traumatology

Florien Jenner, University of Veterinary Medicine, Vienna

**Introduction**: The efficacy of Mesenchymal Stem Cells (MSCs) is attributed to their paracrine effects and MSC secreted extracellular vesicles (EVs) have shown equivalent therapeutic potential to their parent cells. Unfortunately, non‐standardized EV isolation methods impede comparability and reproducibility of results obtained by different laboratories. Hence, we compared two different isolation methods: The broadly used ultracentrifugation (UC) method and size exclusion chromatography (SEC).

**Methods**: EVs were obtained from equine bmMSCs (n = 3 biol. replicates) seeded at 1 × 10^6 cultured in standard media (DMEM + 10% FCS) for 24 hours and 48 hours in serum free media. The vesicles were isolated from 10 ml MSC conditioned medium (MSC‐CM) by UC and by SEC using pre‐casted columns and characterized following the ISEV guidelines. Differences in surface marker stability, size uniformity and number of particles were assessed by Western Blot, Nanotracking Analysis (NTA) and FACS.

**Results**: Successful isolation of EVs was confirmed for both methods using Western Blot (CD9, CD81) and FACS (CD81). 10 ml of MSC‐CM yielded 1569 particles/ml with a size distribution of < 200nm: 87.55 Frequency of parents (FOP), >200‐500nm: 8755 FOP, >200‐500nm: 8.755 FOP using UC (4h processing time) and 795729 particles/ml with a size distribution of 200nm: 81.5 FOP, >200‐500nm: 10.1 FOP, >200‐500nm: 6.52 FOP using SEC (1 h processing time). In order to obtain 1 × 10^6 particles/ml 6373 ml of MSC‐CM and 4 hours are needed using UC and 12.5 ml of MSC‐CM and 1 hour using SEC.

**Summary/Conclusion**: Both methods led to convincing and reproducible EV isolation. SEC isolates particles of the desired size range (35nm‐350nm) more precisely than UC and the yield is 507 times higher. Furthermore, the SEC columns are easier to handle, require less cells and are less time consuming (UC 4h versus SEC 1h). The risk of losing EVs during the isolation process (no washing step required) is reduced.

### PS24.02 More Valid Detection of Potential Biomarkers on Extracellular Vesicles Isolated from Plasma of Rheumatoid Arthritis Patients by Using Optimized Size Exclusion Chromatography

Onno Arntz, Radboudumc

Peter van der Kraan, Radboud University Medical Centre

Marije Koenders, Radboud University Medical Centre

Fons van de Loo, Radboud University Medical Centre

**Introduction**: Isolation of pure extracellular vesicles (EVs) is still a challenge especially from plasma. Size exclusion chromatography (SEC) has become the standard technique of isolating EVs from human plasma. We have shown that only when using optimized SEC (eSEC) EVs can be separated from therapeutic antibodies RA patients are treated with (1). In this study we compared conventional SEC with eSEC and analyzed the protein composition of plasma EVs from RA patients and healthy controls (HC).

**Methods**: EVs were obtained from plasma by either SEC or eSEC. Particle and protein concentrations were detected by NTA and micro‐BCA, respectively. Protein profiles of EVs determined by proteomics from both SEC and eSEC were compared. Thereafter EVs were isolated by eSEC from plasma of 10 RA patients (sex 6F/4M, mean age 56) and 9 healthy controls HC (sex 6F/3M, mean age 59) and compared by mass spectrometry proteomic array. By using Funrich pathways of upregulated and unique RA bounded EV proteins were analyzed.

**Results**: Protein content was 90% lower, while particle concentration was equal in eSEC‐isolated EVs. Only in eSEC isolated EVs the presence of exosome markers (CD9, CD81, CD63, Alix, HSP‐70 and TSG101) could be detected. No differences were observed in particle size and concentration between RA (118 nm, 9.89 × 109 particles/ml) and HC (114 nm, 9.65 × 109 particles/ml) and also the total protein content was equal (RA; 0.30fg/particle, HC; 0.39fg/particle) using eSEC. Proteomic data analysis showed 37 proteins that were significantly enriched, 5 proteins diminished, 11 unique proteins and 1 protein absent in RA‐EVs as compared to HC‐EVs (table 1). In proteomic data of SEC isolated RA and HC EVs only one of these protein was detected. The unique and upregulated proteins in RA EVs showed signaling pathways like Glypican, IL‐3, IL‐5, IFNg which were already described as enriched in RA patients (2).

**Summary/Conclusion**: This study shows that using an optimized SEC column provides purer plasma EVs. The different protein profile we found in eSEC isolated RA‐EVs compared to HC could serve as a fingerprint for this disease, but further research is warranted.

1. Arntz O.J. PlosOne 2020
2. Dolcino M. Cells 2019

### PS24.03 Establishment of a simplified dichotomic size‐exclusion chromatography for purification of extracellular vesicles toward clinical applications

Jiahui Guo, Jinan University

Caihong Wu, Jinan University

Xinyi Lin, Jinan University

Wenting zheng, Jinan University

Tong Wang, Jinan University

Yizhi Cui, Jinan University

**Introduction**: Size‐exclusion chromatography (SEC) is a widely adopted method for the isolation of extracellular vesicles (EVs) from complex samples. SEC can efficiently remove high‐abundant proteins, while often requires multiple fractionation operation using diversified column settings. In this study, we aim to establish a simplified SEC method to acquire high quality EVs that fit the characteristics recommended by MISEV2018.

**Methods**: Stepwise optimizations were performed by comparing the FBS‐derived EVs separation performance of all three cross‐linked Sepharose resins (CL‐2B, CL‐4B and CL‐6B) and different bed volumes of the columns (10 mL and 20 mL) using multiple particle (NTA, nFCM and EM) and protein (BCA, WB and proteomic) characterization techniques. Superior performed column setting was subjected to strategic optimization for further simplify the EVs isolation protocol. Optimized method was then verified in the EVs isolation from human serum and SW620 cell culture supernatant.

**Results**: In comparison of the traditional 10 mL columns, CL‐6B and CL‐4B showed superior performance to CL‐2B with significantly narrower sEVs and protein peaks. By increasing their bed volumes to 20 mL, the resolutions of CL‐6B and CL‐4B columns could be significantly improved, while CL‐6B column had the best performance. With such a column, we further established a dichotomic SEC method that only requires two bulk elution to acquire EVs in Eluate 1 and proteins in Eluate 2. This method can recover ∼80% EVs and is applicable to the sEVs isolation from serum and cell culture supernatant samples that meet the classic characteristics recommended by MISEV2018. We further justified that the CL‐6B columns could be reused for over 10 times.

**Summary/Conclusion**: With its outstanding advantages on simplicity, reproducibility and error‐tolerance, such a method has its intriguing potential to be used for EVs preparation toward clinical testing and/or basic research.

### PS24.04 Isolation and characterization of salivary extracellular vesicles from the MISEV 2018 perspective. A systematic review

Fernanda Jadue, Universidad de Los Andes

Cristián Rojas, Universidad de Los Andes

Felipe Martí, Universidad de Los Andes

José Tomás Rivera, Universidad de Los Andes

**Introduction**: There is consensus among the scientific community about a lack of standardization in the development and reporting of extracellular vesicle (EV) research. This makes studies uncomparable, irreproducible, and affects the validity of the reported conclusions. To address this, the ISEV on 2018 published an update of the Minimal Information for Studies of Extracellular Vesicles (MISEV) guidelines. The aim of this systematic review was to describe the degree of compliance the published methods for the isolation and characterization of salivary extracellular vesicles have with the MISEV 2018 requirements.

**Methods**: A systematic review using MEDLINE, EMBASE, BIREME and EV‐TRACK databases was carried out until august 20th 2020. Only published experimental studies in English or Spanish were human salivary EVs were obtained were included. The primary outcome was the degree of compliance with the mandatory requirements of the following MISEV 2018 domains: nomenclature, collection and pre‐processing, EV separation and concentration, EV characterization and reporting. The functional studies domain was not included. When all the mandatory requirements of the domain were followed the domain was classified as in compliance. A narrative qualitative synthesis was done.

**Results**: Sixty‐three studies were included. None of the studies complied with all the analyzed MISEV domains. Compliance with the nomenclature domain was achieved by 10 (16%) studies, with the separation and concentration domain by 14 (22%) studies. Compliance with the collection and pre‐processing, characterization and reporting domains was not achieved by any study.

**Summary/Conclusion**: None of the included studies complies with all the MISEV 2018 requirements. The degree of compliance with each of the analyzed domains is low. Hence, there is a need to redirect efforts to the standardization of experimental protocols of salivary EV research, as well as improving the quality of reporting.

Review protocol was registered on Open Science Framework.

### PS24.06 Empowering the therapeutic potential of clinically expired platelet concentrates: optimization of platelet‐derived extracellular vesicles isolation process

Daniela Salvador, iBET, ITQB‐NOVA

Ana Meliciano, iBET, ITQB‐NOVA

Ana Filipa F. Louro, iBET; ITQB‐NOVA

Pedro Mendonça, IPST, Instituto Português do Sangue e da Transplantação, Lisboa, Portugal

Ana Paula Sousa, IPST, Instituto Português do Sangue e da Transplantação, Lisboa, Portugal

Margarida Serra, iBET; ITQB‐NOVA

**Introduction**: Platelets have become key players in tissue regeneration due to their enriched content in bioactive growth factors and extracellular vesicles (EV), which can be used in several biomedical applications. However, platelet concentrates (PC) derived from whole blood are regularly produced in blood centers with relative short shelf‐life for clinical applications. In this work, we used clinically expired PC with no therapeutic value, as a promising source of functional EV with high therapeutic potential. In particular, we evaluated different strategies for EV isolation from PC aiming at improving EV yields and purity.

**Methods**: Three EV isolation strategies were evaluated: i) iodixanol‐based gradient ultracentrifugation (DGUC), ii) size exclusion chromatography (SEC), and iii) the combination of these two methodologies (DGUC+SEC). Briefly, PC supernatants were ultracentrifuged and then processed either by DGUC or SEC. DGUC fractions with highest EV contents were further purified by SEC. All EV groups were characterized by nanoparticle tracking analysis, transmission electron microscopy and western blot (WB).

**Results**: Cup‐shaped EV with an intact lipid bilayer were obtained in all three strategies. The highest EV yields were attained by DGUC (4.48‐1011 particle/mL) when compared to other strategies (9.82‐1010 and 1.62‐1010 particle/mL for SEC and DGUC+SEC, respectively). WB analysis showed contamination with Apolipoprotein A1 and Argonaute‐2 in all EV groups, despite the presence of specific EV markers CD63 and TSG101 which were enriched in the DGUC and SEC samples

**Summary/Conclusion**: This work provides important insights towards the valorization of PC with no therapeutic value. We show the potential of DGUC methodology to isolate EV with high efficiency and purity from clinically expired PC.

### PS24.07 Optimized purification of EVs released by blood eating parasites: mini‐gradients for EVs from limited sources

Marije Kuipers, MSc, Leiden University Medical Center

Cornelis H. Hokke, Prof.dr., Leiden University Medical Center

Hermelijn H. Smits, Dr., Leiden University Medical Center

Esther N.M Nolte – ‘t Hoen, Dr., Utrecht University

**Introduction**: Isolation of extracellular vesicles (EVs) from blood eating parasites, such as Schistosoma mansoni, is challenging. The complex life cycle and the necessity to use animal hosts for maintaining the parasite limit the available material for EV isolation. Moreover, the biophysical and molecular properties of EVs differ between the parasite's life cycle stages and the immunogenic and toxic byproduct of hemoglobin digestion, hemozoin, contaminates adult worm EV populations enriched by ultracentrifugation. Therefore, we designed optimized protocols for isolation of Schistosoma‐derived EVs.

**Methods**: Medium of ex vivo cultured adult worms or in vitro transformed larvae was pre‐processed by differential (ultra)centrifugation. We compared SEC and density gradients with sucrose or iodixanol for separation of EVs from non‐EV products. Conventional gradients in SW55 (4.5 mL, 16 hours spin) were compared to mini‐gradients in TLS55 (1.8 mL, 2 hours spin). Hemozoin was verified visually or by cryo EM. EVs were detected by western blot analysis for Tetraspanin 2.

**Results**: EVs could be separated from non‐EV products, including hemozoin, by density gradient centrifugation but not by ultracentrifugation or SEC. Larval EVs that contain filamentous structures on their surface were able to float efficiently into iodixanol but not into sucrose gradients, whereas adult worm EVs floated into both types of gradients. With the mini‐gradients, pure EVs could be recovered in substantially reduced volumes, abolishing the need for additional EV concentration steps that decrease EV yield.

**Summary/Conclusion**: Our data indicate that specific parasite EV subsets can display differential migration behavior in density gradients prepared with sucrose or iodixanol. We propose the use of mini‐gradients for purifying EVs from limited sources, such as parasites, to increase EV yield while reducing handling time.

### PS24.08 Scalable Production of ARRDC1 Mediated Microvesicles (ARMMs) as Non‐Viral Vehicles for the Delivery of Therapeutic Payloads

Kristin Luther, Vesigen Therapeutics

Alysia Bryant, Vesigen Therapeutics

Leah Gens, Vesigen Therapeutics

Aditi Jhaveri, Vesigen Therapeutics

Qiyu Wang, Vesigen Therapeutics

Lucy Sun, Vesigen Therapeutics

Nedyalka Valkov, Vesigen Therapeutics

Joseph Nabhan, Vesigen Therapeutics

**Introduction**: Arrestin domain containing protein 1 (ARRDC1) mediates plasma membrane budding to produce extracellular vesicles (EVs) called ARMMs (ARRDC1 Mediated Microvesicles). This class of EVs was shown to package and deliver complex protein payloads in vivo for the purpose of intercellular communication and signaling. Recently, ARMMs were adapted for use as a versatile tool for the packaging and delivery of a range of cargos such as proteins and RNA. Here, we present the development of a scalable and GMP‐compliant process for payloading ARMMs with protein cargos.

**Methods**: We used transient transfection or stable cell lines to produce ARMMs containing various protein payloads. Clarified conditioned media (CM) were concentrated either by ultracentrifugation or tangential flow filtration (TFF) to produce materials suitable for in vitro and in vivo studies. The stability of ARMMs was measured over time and after multiple freeze‐thaw cycles. Cargo loading and stability were assessed by NTA, Western blot, and ELISA.

**Results**: Cells transfected with ARMMs payloading constructs consistently showed 5–10 fold increases in EV/ARMMs production over baseline. Following purification, yields of 90% were consistently obtained using TFF and our process removed 78% of non‐EV protein. Active protein payloading in ARMMs was consistent and efficient. ARMMs remained stable with unaltered properties after storage at ‐80C for over two months and after up to 5 freeze thaw cycles.

**Summary/Conclusion**: Production and purification of ARMMs from suspension cells represent a significant step forward in establishing the ARMMs system as a scalable platform for non‐viral delivery of macromolecules. ARMMs are readily payloaded with effector proteins during biogenesis from cells and this system can be co‐opted to payload proteins of interest. Together with the demonstrated stability of ARMMs, this platform is a promising new vehicle for therapeutic delivery.

### PS24.09 Free flow electrophoresis allows quick preparation of extracellular vesicles from cell culture supernatants and human plasma

Simon Staubach, Institute for Transfusion Medicine, University Hospital Essen, University of Duisburg‐Essen, Essen, Germany

Tobias Tertel, Institute for Transfusion Medicine, University Hospital Essen, Germany

Verena Börger, Institute for Transfusion Medicine, University Hospital Essen, University of Duisburg Essen, Essen, Germany

Christian Grätz, Technische Universität München

Michael W. Pfaffl, PhD, Chair of Animal Physiology & Immunology

Oliver Drews, University Hospital Mannheim, Department of Chemistry

Gerhard Weber, FEE Service

Bernd Giebel, Prof, Institute for Transfusion Medicine, University Hospital Essen, Germany

**Introduction**: Despite increasing interests in extracellular vesicles (EVs), it remains a long procedure to prepare extracellular vesicles (EVs) to high purity. Neither fractionation by density nor by size alone is sufficient to separate EVs from most contaminants including lipoproteins. For now, a time‐consuming combination of two methods (density and size separation) is required to enrich EV to high purity at the expense of their recovery. During the recent years, we qualified Free Flow Electrophoresis (FFE) as efficient method for EV separation.

**Methods**: FFE is a well‐established (semi‐) preparative method to separate analytes with inherent difference in charge density and/or their pI‐values into up to 96 different fractions. Upon applying imaging flow cytometry analyses to identify EV containing fractions, we have optimized FFE protocols for the preparation of bona fide EVs from conditioned cell culture media and demonstrated the reproducibility of the method.

**Results**: Applying a comparable strategy, we now have improved FFE protocols for the preparation of EVs from human plasma samples. Notably, EVs from plasma show a much higher EV complexity than of cell culture supernatants. Specifically, plasma EVs are recovered in more than three FFE fractions. Several of these fractions also contain relatively high protein contents which could effectively be reduced by subsequent ultra‐filtration (UF). The whole procedure takes approximately 40 min per plasma sample and compared to other technologies can be considered as relative quick. Currently, we are characterizing the EVs obtained from different pooled FFE and ultra‐filtrated fractions by RNA and proteome analyses.

**Summary/Conclusion**: The whole procedure takes approximately 40 min per plasma sample and compared to other technologies can be considered as relative quick. Currently, we are characterizing the EVs obtained from different pooled FFE and ultra‐filtrated fractions by RNA and proteome analyses.

## PS25 EVs in Cardiovascular Diseases and Vascular Disorders

Chair: Uta Erdbruegger, University of Virginia Health System, United States

Chair: Xavier Loyer, INSERM U970‐PARCC, France

### PS25.01 Circulating microRNA‐122‐5p correlates with improvement in left‐ventricular function after transcatheter aortic valve replacement and regulates viability of cardiomyocytes via extracellular vesicles

Mohammed Rabiul Hosen, University of Bonn

**Introduction**: Transcatheter aortic valve replacement is a well‐established treatment option for high‐ and intermediate‐risk patients with severe symptomatic aortic valve stenosis. The majority of patients experience an improvement of the left ventricular ejection fraction after TAVR in response to TAVR‐associated afterload reduction. However, a specific role for circulating microRNAs in the improvement of cardiac function for patients after TAVR has not yet been investigated. Herein, we generally explored the differential expression of miRNAs in circulating extracellular vesicles in patients after TAVR and, in particular, the novel role of circulating miR‐122‐5p in cardiomyocytes.

**Methods**: Circulating EV‐associated miRNAs were investigated by using an unbiased Taqman‐based human miR array. Several EV‐miRNAs (miR‐122‐5p, miR‐26a, miR‐192, miR‐483‐5p, miR‐720, miR‐885‐5p, and miR‐1274) were significantly deregulated in aortic stenosis patients at day seven after TAVR in comparison to the pre‐procedural levels in patients without LVEF‐improvement. The higher levels of miR‐122 were negatively correlated with LVEF improvement at both day seven (r = ‐0.264 and p = 0.015) and at six months (r = ‐0.328 and p = 0.0018) after TAVR. At the three‐year follow‐up, patients with a higher level of miR‐122‐5p displayed significantly increased cardiovascular mortality (p = 0.03). By utilization of patient‐derived samples and a murine aortic‐stenosis model, we observed that the expression of miR‐122‐5p correlates negatively with cardiac function, which is associated with LVEF. Graded wire‐injury‐induced aortic‐valve‐stenotic mice demonstrated a higher level of miR‐122‐5p, which was related to cardiomyocyte dysfunction.

**Results**: Gain‐ and loss‐of‐function experiments suggested that EV‐mediated shuttling of miR‐122‐5p increases the level of miR‐122‐5p in recipient cardiomyocytes and regulates viability of the cardiomyocytes. In silico prediction and mass spectrometry were used to search for binding partners of miR‐122‐5p. Mechanistically, miR pulldown, electrophoretic mobility shift assay, and RNA immunoprecipitation confirmed that miR‐122‐5p interacts with an RNA‐binding protein, hnRNPU, in a sequence‐specific manner to encapsulate miR‐122‐5p into large EVs. Upon shuttling into recipient cells, miR‐122‐5p negatively regulates the expression of the anti‐apoptotic gene BCL2, by binding to its 3' untranslated region to regulate its translation, and thereby decreasing the viability of target cardiomyocytes.

**Summary/Conclusion**: Increased levels of circulating pro‐apoptotic EV‐incorporated miR‐122‐5p is associated with reduced LVEF after TAVR. Extracellular vesicular shuttling of miR‐122‐5p regulates the viability and apoptosis of cardiomyocytes in a Bcl2‐dependent manner.

### PS25.02 High Glucose Macrophage Exosomes Enhance Atherosclerosis by Driving Cellular Proliferation & Hematopoiesis

Robert Raffai, PhD, University of California San Francisco

Laura Bouchareychas, UCSF

Phat Duong, NCIRE

Tuan Anh Phu, NCIRE

Eric Alsop, TGen

Bessie Meechoovet, TGen

Rebecca Reiman, TGen

Martin Ng, NCIRE

Ryo Yamamoto, Stanford University

Hiromitsu Nakauchi,Stanford University

Warren Gasper, UCSF

Kendall Van Keuren‐JensenTranslational Genomics Research Institute

**Introduction**: Hyperglycemia is a recognized risk factor for cardiovascular complications in diabetes including atherosclerosis. While hyperglycemia is known to cause microRNA dysregulation that contributes to cellular activation, whether it can do so through intercellular communication via extracellular vesicles (EVs) is not known.

**Methods**: We investigated whether EVs produced under hyperglycemic conditions could communicate signaling to drive atherosclerosis. We did so by treating Apoe'/'mice with exosomes produced by bone marrow‐derived macrophages (BMDM) exposed to high glucose (BMDM"HG‐exo) or control.

**Results**: Repeated infusion of BMDM “HG‐exo led to a robust increase in hematopoiesis leading to augmented circulating myeloid cell numbers. Four weeks of BMDM” HG‐exo infusions increased atherosclerotic lesion sizes with an accumulation of macrophage and apoptotic cells in the aortic root. Transcriptome‐wide analysis of naïve cultured macrophages treated with BMDM “HG‐exo showed an upregulation of genes associated with cell proliferation. Furthermore, BMDM” HG‐exo reprogramed energy metabolism in recipient macrophages with an upregulation of glycolytic activity. Plasma EVs isolated from human subjects with type II diabetes exerted similar cell signaling when incubated with cultured human macrophages. Lastly, profiling microRNA in BMDM"HG‐exo and human diabetic plasma EVs converged on miR‐486 as commonly enriched.

**Summary/Conclusion**: In conclusion, our findings show that macrophage EVs serve to communicate detrimental properties of hyperglycemia to accelerate atherosclerosis. Furthermore, our findings identify miR‐486 in human plasma EVs as a possible biomarker of advanced peripheral atherosclerosis.

### PS25.03 Extracellular Vesicles Derived from Intermittent Hypoxia–Treated Red Blood Cells Impair Endothelial Function Through Regulating eNOS Phosphorylation and ET‐1 Expression

Lu Peng, Key Laboratory of Upper Airway Dysfunction‐related Cardiovascular Diseases, Beijing Institute of Heart, Lung and Blood Vessel Disease, Beijing Anzhen Hospital, Capital Medical University.

Yongxiang Wei, Department of Otolaryngology‐Head and Neck Surgery, Beijing Anzhen Hospital, Capital Medical University.

Huina Zhang, Key Laboratory of Upper Airway Dysfunction‐related Cardiovascular Diseases, Beijing Institute of Heart, Lung and Blood Vessel Disease, Beijing Anzhen Hospital, Capital Medical University.

**Introduction**: Intermittent hypoxia (IH), a main characteristic of obstructive sleep apnea (OSA) syndrome, has been known as a dominant cause of OSA‐related endothelial dysfunction and hypertension. However, the underlying mechanism still remains unclear. Extracellular vesicles (EVs), small vesicles secreted by various cells, can be absorbed by endothelial cells and then influence vascular function. The aim of this research is to clarify whether and how EVs shedding from red blood cells (RBCs) are involved in IH‐induced endothelial dysfunction.

**Methods**: EVs were extracted by ultracentrifugation. After the identification of property and purity, EVs from IH‐exposed RBCs (IH REVs) and normoxia‐exposed RBCs (NOR REVs) or from OSA and non‐OSA patient RBCs were utilized to treat C57BL/6 mouse aortas or human umbilical vein endothelial cells (HUVECs) for mechanistic exploration.

**Results**: Functional results demonstrated that REVs from OSA patients dramatically impaired endothelium‐dependent relaxations (EDRs). Similarly, in vivo and ex vivo studies showed that IH REVs caused significant endothelial dysfunction compared to control group. Further results presented that IH REVs blocked endothelial nitric oxide synthase (eNOS) phosphorylation through inhibiting PI3K/Akt pathway and enhanced endothelin‐1 (ET‐1) expression through activating Erk1/2 pathway in endothelial cells. Meanwhile, endothelial dysfunction caused by IH REVs was reversed by Akt activator SC79 as well as Erk kinase inhibitor PD98059, suggesting that PI3K/Akt/eNOS and Erk1/2/ET‐1 pathways were implicated in IH REV‐induced impaired EDRs.

**Summary/Conclusion**: This study reveals a novel role of REVs in endothelial dysfunction under IH and dissects the relevant mechanism involved in this process, which will help to establish a comprehensive understanding of OSA or IH‐related endothelial dysfunction from a new scope.

### PS25.04 Extracellular vesicle microRNA cargoes from intermittent hypoxia‐exposed cardiomyocytes and their effect on endothelium

Yu Li, Beijing Anzhen Hospital, Capital Medical University

xinliang Ma, Professor

Huina Zhang, Key Laboratory of Upper Airway Dysfunction‐related Cardiovascular Diseases, Beijing Institute of Heart, Lung and Blood Vessel Disease, Beijing Anzhen Hospital, Capital Medical University.

**Introduction**: Intermittent hypoxia (IH), as the crucial pathophysiological feature of obstructive sleep apnea syndrome (OSAS), is an independent risk factor initiating the progression of cardiovascular complications. Extracellular vesicles (EVs) are involved in multiple pathological process, including cardiovascular complications via delivering various molecule cargos to recipient cells. Uncovering the miRNA components in EVs derived from myocardium will help understanding the mechanism of IH‐induced cardiovasculopathy. However, the information of EV miRNA cargos from IH‐exposed cardiomyocyte remains unclear. This study aims to reveal the differential miRNA expression of cardiomyocytes‐derived EVs after IH and normoxia treatment.

**Methods**: EVs were isolated from primary cardiomyocytes with normoxia and intermittent hypoxia ‐treatment. An unbiased miRNA array assay was used to identify cardiac EV miRNA. The expression of target miRNAs was validated by qPCR assay and the enriched function of the target miRNAs were predicted by bioinformatics analysis.

**Results**: 63 differentially expressed miRNAs, including 32 up‐regulated miRNAs and 31 downregulated miRNAs were identified in EVs from IH‐exposed cardiomyocytes. Among them, 16 miRNAs with homologous sequence in mouse and human were selected and verified by qPCR assay and 11 miRNAs were proved with the same tendency as miRNA array identified. KEGG pathway analysis showed the main enriched pathway of differentially expressed miRNA is associated with PI3K/Akt signaling pathway.

**Summary/Conclusion**: This study revealed characteristic microRNAs with altered expression in EVs from intermittent hypoxia‐treated cardiomyocytes, which might provide the potential molecular targets for further deepening investigation on clinical or basic research about the EV function in IH‐induced cardiomyopathy.

PS25 EVs in Cardiovascular Diseases and Vascular Disorders

### PS25.05 Extracellular vesicles from Acute Coronary Syndrome patients and myocardial ischemia‐reperfusion injury

Saveria Femminò, University of Turin

Fabrizio D'Ascenzo, University of Turin

Francesco Ravera, University of Turin

Filippo Angelini, University of Turin

Andrea Caccioppo, University of Turin

Luca Franchin, University of Turin

Alberto Grosso, University of Turin

Stefano Comità, University of Turin

Claudia Cavallari, University of Turin

Claudia Penna,University of Turin

Gaetano Maria De Ferrari, University of Turin

Giovanni CamussiUniversity of Turin

Pasquale Pagliaro, University of Turin

Maria Felice MF Brizzi,University of Turin

**Introduction**: The Remote Ischemic PreConditioning (RIPC) approach fails to reduce Ischemia/Reperfusion (I/R) injury in Acute Coronary Syndrome (ACS) patients that have undergone Percutaneous Coronary Intervention (PCI). Extracellular vesicles (EV) have attracted interest as mediators of tissue damage/recovery upon I/R injury. In the present study we investigated the effect of serum‐derived EV recovered from ACS patients (Non‐ST Elevation Myocardial Ischemia, Unstable Angina), treated with PCI and subjected to the RIPC procedure or otherwise, in preventing I/R injury.

**Methods**: A double‐blind, randomized, sham‐controlled study (NCT02195726) has been extended, and EV recovered from 30 patients who were randomly assigned (1:1) to undergo the RIPC‐ (EV‐RIPC) or sham‐procedures (EV‐naive) before PCI.

**Results**: We found that troponin (TnT) was enriched in EV, compared to healthy subjects, regardless of diagnosis and the magnitude of its circulating level. Moreover, we demonstrated that EV‐naive, unlike EV‐RIPC, induced protection against I/R, both in‐vitro and in the whole heart. Mechanistically, we noticed that STAT‐3 was a crucial mediator of EV‐naive protection. Interestingly, Erk‐1/2 targeting rescued STAT‐3 activation and protection upon EV‐RIPC treatment. EV were screened for 84 Human Cardiovascular Disease mRNAs. DUSP6 mRNA was significantly enriched in EV‐naïve from most patients (n = 13/15). DUSP6 silencing in EV‐naive prevented STAT‐3 phosphorylation and their cardio‐protective properties.

**Summary/Conclusion**: This study first demonstrates the cardio‐protective activity and mechanism of action of EV‐naive, and validates a potency test to predict biological activity ex‐vivo. Moreover, it provides insight on the unsuccessfulness of the RIPC‐procedure in humans. Finally, we propose EV‐TnT content as a sensitive marker to estimate I/R damage in clinics.

### PS25.06 Plasma exosomes in Obstructive Sleep Apnea patients promote cardiomyocytes fibrosis and hypertrophy

Hui Gong, The Second Xiangya Hospital, Central South University

Xing Lyu, The Second Xiangya Hospital, Central South University

Xiangyu Zhang, The Second Xiangya Hospital, Central South University

**Introduction**: OSA has been particularly implicated as mechanistically contributing to the prevalence and severity of cardiovascular diseases (CVD). The fibrosis and hypertrophy of cardiomyocytes are important contributor to cardiac end‐organ dysfunction. It is essential to develop novel pharmacological agents to counteract the pathophysiological mechanisms responsible for OSA‐related adverse consequences. Exosomes are released ubiquitously into the circulation, and transfer their cargo to target cells facilitating physiological and pathological processes.

**Methods**: Exosomes from fasting morning plasma samples from untreated OSA (OSA‐Exos) and healthy controls(non OSA‐Exos) were applied on cardiomyocytes H9C2. Expression of fibrotic and hypertrophic gene markers including Col1A1, ANP, and BNP and immunofluorescence staining for Col1A1 were performed. In addition, equivalent OSA‐Exos amounts were added to H9C2 in culture, after which autophagy and Akt/mTOR expression changes were assessed by western blotting.

**Results**: Exosmes from untreated OSA induced significant increases in Col1A1, ANP, and BNP compared to controls. Futhermore, we found that the expression of LC3B and beclin1, indicators of autophagosome accumulation, were decreased in the OSA‐Exos groups. p‐Akt/Akt and p‐mTOR/mTOR displayed a higher level in OSA‐Exos treated groups than in the control group. OSA‐Exos inhibited autophagy and Akt/mTOR signaling pathway was involved in the regulation autophagy by OSA‐Exos.

**Summary/Conclusion**: OSA derived exosomes contributed to cardiomyocytes fibrosis and hypertrophy and inhibited autophagy via Akt/mTOR signaling pathway.

### PS25.07 Polyphosphate Expression by Extracellular Vesicles Mediates Binding of Contact Factors and Contact Activation

Young Jun Shim, Cleveland Clinic

Shadi Swaidani, Cleveland Clinic

Ravi K. Alluri, Cleveland Clinic

Suman Kundu, Cleveland Clinic

John Barnard, Cleveland Clinic

Alvin Schmaier, Case Western Reserve University

Alok Khorana, Cleveland Clinic

Keith McCrae, Cleveland Clinic

**Introduction**: Extracellular vesicles (EV) are elevated in cancer. Studies suggest that EV may stimulate thrombosis in cancer patients through expression of tissue factor. However, limited data implicates EV in activation of the contact pathway of coagulation through support of factor XII (FXII) autoactivation to factor XIIa (FXIIa). To better define the ability of EV to initiate contact activation, we compared the ability of EV derived from different cancer cell lines to support FXII autoactivation.

**Methods**: EVs derived from cancer cell lines and plasma of cancer patients were isolated using qEV size exclusion column (IZON) and pan‐exosome isolation kit (MACS Miltenyi Biotech), respectively. Isolated EVs were analyzed by ZetaView and electron microscopy. FXII autoactivation in normal human plasma (NHP) incubated with EVs was analyzed by measuring S‐2302 hydrolysis in the presence or absence of DNase, RNase, and CIP (calf intestinal alkaline phosphatase). To examine the role of FXII, corn trypsin inhibitor (FXIIa inhibitor) or immunodepleted FXII‐deficient plasma was used. EV‐associated polyphosphate (polyP) and its interaction with FXII was detected using alexaFluor488‐labelled mutant exopolyphosphatase (PPX∆12) and FXII, respectively by measuring EV in fluorescence mode. To assess in vivo thrombosis, wild‐type, f12‐/‐, klkb1‐/‐, or kgn1‐/‐ mice were injected with cancer cell‐derived EV with or without CIP pretreatment and pulmonary emboli was analyzed by immunostaining of fibrin and platelet deposition in lung. Cleaved high molecular weight kininogen (cHK) in NHP was evaluated by Li‐COR immunoblotting. cHK levels in plasma from patients with cancer were evaluated using WES capillary immunoassay (ProetinSimple).

**Results**: EV provided a platform for FXII autoactivation in a manner proportionate to the clinical thrombotic risk of the tumors from which these cell lines were derived. Concordant with autoactivation of FXII, EV induced the cleavage of high molecular weight kininogen (HK) to cHK. Additionally, EV from cancer patients stimulated FXII autoactivation and HK cleavage. EV treated with CIP or E. Coli exopolyphosphatase degraded polyphosphate so that blocked binding of FXII to EV and the ability of EV to support FXII autoactivation. In vivo, EV induced pulmonary thrombosis in wild‐type mice, with protection conferred by deficiency of FXII, HK, or prekallikrein. Moreover, pre‐treatment of EV with CIP inhibited their prothrombotic effect.

**Summary/Conclusion**: Polyphosphate in EV supports contact activation and may additionally contribute to the prothrombotic effects of EV in cancer.

### PS25.08 Pro‐inflammatory effect of extracellular vesicles released by senescent‐like C2C12 myocytes

Serena Maggio, University of Urbino

Andrea Cioccoloni, Department of Biomolecular Sciences, University of Urbino Carlo Bo, Urbino, Italy.

Paola Ceccaroli, Department of Biomolecular Sciences, University of Urbino Carlo Bo, Urbino, Italy.

Emanuela Polidori, Department of Biomolecular Sciences, University of Urbino Carlo Bo, Urbino, Italy.

Michela Battistelli, Department of Biomolecular Sciences, University of Urbino Carlo Bo, Urbino, Italy.

Giovanni Piccoli, Department of Biomolecular Sciences, University of Urbino Carlo Bo, Urbino, Italy

Vilberto Stocchi, Department of Biomolecular Sciences, University of Urbino Carlo Bo, Urbino, Italy.

Michele Guescini, University of Urbino Carlo Bo

**Introduction**: Sarcopenia is a geriatric syndrome involving progressive and generalized loss of skeletal muscle mass and strength. Moreover, aged muscles also show an altered mitochondrial function, increased protein degradation, inflammation, and loss of satellite cell (SC) number and function. It has been suggested that an altered secretory pattern involving inflammatory, growth‐promoting, and remodeling factors, called senescence‐associated secretory phenotype (SASP), could induce sarcopenia. Although the SASP has long been considered composed of soluble factors, growing evidence has recently emerged for the role of extracellular vesicles (EVs) as key players within the senescent cells secretome.

This study aimed to investigate whether the EVs released by senescent‐like myocytes could affect the behavior of surrounding myoblasts and macrophages, critical players in the muscle niche.

**Methods**: Two models of senescent‐like C2C12 cells were used: C2C12 cells, an in vitro model of myogenic differentiation, were cultured for more than 40 passages (high passages, HP) or subjected to oxidative stress (OX). EVs were obtained using serial ultracentrifugation and density gradient, quantified through Nanoparticles Tracking Assay (NTA), characterized by Western Blot (WB), and used to treat the recipient cells. Expression analyses were performed using RT‐qPCR and WB.

**Results**: HP and OX myocytes displayed senescence‐associated characteristics such as defective myogenic differentiation process and a significantly inflamed status compared to C2C12 cells cultured in standard condition at low passages (LP).

The EV quantification showed that HP myocytes release more EVs compared to LP cells. Besides, EVs secreted by HP and OX cells carried a higher amount of nucleic acids than controls, especially DNA.

Furthermore, the effect of HP‐ and OX‐derived EVs on LP C2C12 and RAW264.7 cells was tested by RT‐qPCR using inflammatory mRNA markers. The obtained results revealed that senescent‐like EVs induce IL‐6 and IL‐1b expression.

**Summary/Conclusion**: The reported data suggest that EVs released by senescent‐like myocytes induce an inflamed status in recipient cells. Of note, the observed inflammatory response seems to underlie the derangement of the myogenic process found in LP differentiating myocytes.

### PS25.09 Chemically modified myocytes‐derived EVs for the treatment of cardiac fibrosis

Marta Prieto‐Vila, Tokyo Medical University

Asao Muranaka, Tokyo Medical University

Takahiro Ochiya, PhD, Department of Molecular and Cellular Medicine, Tokyo Medical University

**Introduction**: Cardiac fibrosis is a disorder that oftentimes occurs after a cardiac injure. Fibroblasts are erroneously activated, causing an excessive accumulation of extracellular matrix (ECM), decreasing cardiac function, and eventually leading to death. It is known that adult cardiomyocytes communicate with the surrounding cells, including fibroblasts, by extracellular vesicles (EVs). The loss of this communication is thought to play a central role in cardiac fibrosis. Therefore, cardiomyocyte‐derived EVs may be a promising cell‐free system for the treatment of fibrosis inhibition.

**Methods**: By the use of a defined conjunct of chemicals, mature cardiomyocytes culture was highly improved to ensure a high collection of EVs. Terminal differentiation, as well as senesce markers apparition, was delayed in comparison to the predetermined culture medium. EVs were isolated by ultracentrifugation, and their effect on fibrosis was tested. For that, prior to any treatment, and to mimic fibrosis, primary cardiac fibroblast were activated overnight with TGFβ.

**Results**: Chemically‐treated myocytes secreted a rather large amount of EVs, which expressed the common EVs membrane markers. TGFβ‐treated cardiac fibroblasts co‐cultured with myocytes showed a decrease of fibroblast activation markers both at mRNA and protein levels. Furthermore, ECM secretion was also reduced. Similar results were found when activated fibroblasts were treated with EVs. Next generation sequencing of EVs showed multiple myocyte‐specific and antifibrotic‐related microRNAs. The effect of those microRNAs is currently under validation.

**Summary/Conclusion**: Thanks to the large amount of cardiac‐specific and anti‐fibrotic microRNAs encapsulated within adult myocytes‐derived EVs, fibrosis is significantly reduced in activated cardiac fibroblasts. Our findings indicate that the use of EVs derived from chemically modified myocytes is a promising treatment for ischemic myocardial fibrosis.

### PS25.10 Detection of systemic arteriosclerosis by miRNA transcriptional analysis of EV enriched serum samples

Alex Hildebrandt, Technical University of Munich

Benedikt Kirchner, MSc, Technical University of Munich

Agnes S. Meidert, University Hospital, Ludwig‐Maximilians‐University Munich

Florian Brandes, Ludwig‐Maximilians‐University Munich

Dominik Buschmann, Technical University of Munich

Anja Lindemann, University Hospital, Ludwig‐Maximilians‐Universität München

Alexander Doege, Ludwig‐Maximilians‐University Munich

Rolf Weidenhagen, Muenchen Klinik Neuperlach

Marlene Reithmair,University Hospital, Ludwig‐Maximilians‐Universität München

Gustav Schelling, University Hospital, Ludwig‐Maximilians‐Universität München

Michael W. Pfaffl, PhDChair of Animal Physiology & Immunology

**Introduction**: Atherosclerosis is a slowly progressive disorder leading to arterial hypoperfusion of heart, brain, other important organ systems and the peripheral extremities. Severe cases can result in aneurysma formation, organ failures and loss of extremities. The success of therapy depends largely on the early identification of individuals at risk and the timely intervention with behavioural modification, pharmacologic treatment and surgery. Early detection of arteriosclerosis requires sensitive biomarkers as indicators of endothelial damage, plaque formation and inflammation. Most cells of the vascular endothelium and ischemic organs can shed extracellular vesicles (EVs) of different type and RNA content. Our primary aim was the identification of EV‐related miRNAs which allow an early detection of atherosclerosis. In a second approach, we tested whether the EV miRNA profile was able to identify organ‐specific manifestations of arteriosclerosis.

**Methods**: We studied 117 patients with clinically confirmed arteriosclerosis; 33 had coronary heart disease, 25 carotid stenosis, 30 aortic aneurysm and 29 peripheral artery disease. 28 healthy individuals served as controls, EVs were purified from serum by precipitation, total RNA was isolated and small‐RNA was profiled by NGS. Differential gene expression (DGE) was performed, and significant regulated miRNAs were analyzed by multivariate analyses via PCA and PLS‐DA. Differentially regulated miRNAs were analyzed by Ingenuity Pathway Analysis (IPA) to characterize endothelial and organ specific signaling pathways.

**Results**: Expression values of 9 miRNAs were found to discriminate between atherosclerosis and the healthy state (p < 0.005) and can be linked to phenotypical processes. The data set from RNA‐seq was unable to differentiate between organ and vascular manifestations of the disorder due to high group variations. Based on 12 differentially expressed miRNAs, IPA revealed a fully activated network of targeted cytokines (IL‐10), receptors (IGF1R, IL2R) and adhesion molecules (ALCAM) indicating ongoing inflammation and endothelial activation.

**Summary/Conclusion**: A stable miRNA signature was characterized which could be useful for the early detection of systemic atherosclerosis.

### PS25.11 Non‐valvular atrial fibrillation patients anticoagulated with rivaroxaban compared with warfarin exhibit reduced circulating extracellular vesicles with attenuated pro‐inflammatory protein signatures

Luisa Weiss, School of Biomolecular and Biomedical Science, University College Dublin, Dublin, Ireland

John Keaney, Department of Cardiology, Mater Misericordiae University Hospital, Dublin, Ireland

Paulina Szklanna, School of Biomolecular and Biomedical Science, University College Dublin, Dublin, Ireland

Tadhg Prendiville, Department of Haematology, Mater Misericordiae University Hospital, Dublin, Ireland

Sarah Kelliher, Department of Haematology, Mater Misericordiae University Hospital, Dublin, Ireland

Karl Ewins, Department of Haematology, Mater Misericordiae University Hospital, Dublin, Ireland

Shane comer, School of Biomolecular and Biomedical Science, University College Dublin, Dublin, Ireland

Ellen O'Rourke, Department of Haematology, Mater Misericordiae University Hospital, Dublin, Ireland

Áine Lennon, Department of Haematology, Mater Misericordiae University Hospital, Dublin, Ireland

Alfonso Blanco,Flow Cytometry Core, Conway Institute, University College Dublin, Dublin, Ireland

Barry Kevane, Department of Haematology, Mater Misericordiae University Hospital, Dublin, Ireland

Séan MurphyDepartment of Stroke Medicine, Mater Misericordiae University Hospital, Dublin, Ireland

Fionnuala Ní Áinle, Department of Haematology, Mater Misericordiae University Hospital, Dublin, Ireland

Patricia Maguire,School of Biomolecular and Biomedical Science, University College Dublin, Dublin, Ireland

**Introduction**: Rivaroxaban, a direct oral factor Xa inhibitor, mediates anti‐inflammatory and cardiovascular‐protective effects besides its well‐established anticoagulant properties, however, these remain poorly characterized. Extracellular vesicles (EVs) are important circulating messengers regulating a myriad of biological and pathological processes and may be highly relevant to the pathophysiology of atrial fibrillation as they reflect alterations in platelet and endothelial biology. However, the effects of rivaroxaban on circulating pro‐inflammatory EVs remain unknown. We hypothesized that rivaroxaban's anti‐inflammatory properties are reflected upon differential molecular profiles of circulating EVs.

**Methods**: Patients were recruited following informed written consent according to the declaration of Helsinki. Plasma EV size and concentration were characterised by Nanoparticle Tracking Analysis and flow cytometry. To investigate differences in protein expression, EVs were enriched 3 times by ultracentrifugation at 110,000xg and subjected to label‐free quantification mass spectrometry.

**Results**: Rivaroxaban‐treated non‐valvular atrial fibrillation (NVAF) patients (n = 8) exhibit significantly reduced levels of circulating small EVs (< 200 nm) compared with matched warfarin controls (n = 15, p = 0.003), while large EV levels were unaffected. Quantitative proteomic analysis of enriched plasma EVs from six pooled biological donors per treatment group revealed a profound decrease in highly pro‐inflammatory protein expression and complement factors, together with increased expression of negative regulators of inflammatory pathways. Crucially, a reduction in circulating levels of platelet activation marker soluble P‐selectin was observed in rivaroxaban‐treated patients (compared with warfarin controls), which negatively correlated with the patient's time on treatment.

**Summary/Conclusion**: Collectively, these data demonstrate that NVAF patients anticoagulated with rivaroxaban (compared with warfarin) exhibit both a reduced pro‐inflammatory state and evidence of reduced platelet activation. These findings are of translational relevance towards characterizing the anti‐inflammatory and cardiovascular‐protective mechanisms associated with rivaroxaban therapy.

### PS25.12 Characterising extracellular vesicle release from human coronary artery vascular smooth muscle cells exposed to oxidised LDL

Eleni Charla, University of GlasgoInstitute of Cardiovascular & Medical Sciences, University of Glasgow, Glasgow, UK

John Mercer, Institute of Cardiovascular & Medical Sciences, University of Glasgow, Glasgow, UK

Pasquale Maffia, University of GlasgoInstitute of Infection, Immunity and Inflammation, University of Glasgow, Glasgow, UK

Stuart A A. Nicklin, Institute of Cardiovascular & Medical Sciences, University of Glasgow, Glasgow, UK

**Introduction**: Low‐density lipoprotein (LDL) accumulation and its oxidation to oxidized LDL (oxLDL) in coronary arteries lead to the development of atherosclerotic plaques and subsequent plaque rupture which is a leading cause of death worldwide. Extracellular vesicles (EVs) are secreted from a range of cells that contribute to atherosclerotic plaque development, including endothelial cells (ECs), vascular smooth muscle cells (VSMC), and immune cells. Researching EV signaling in atherosclerosis is currently of great interest.

**Methods**: Primary human coronary artery SMC (HCASMC) were incubated ± 10mg/mL oxLDL for 24h, media removed, cells washed, and then replaced with fresh serum‐free media. After 48h, EVs were purified from the media by size exclusion chromatography. EVs were characterized with Nanosight tracking analysis (NTA), transmission electron microscopy (TEM), and western immunoblotting. EVs were treated ± Proteinase K (PK), lysed, and investigated for the presence of oxLDL by ELISA. Viability of HCASMC exposed to control and oxLDL‐EVs was measured by MTT assay where quiescent HCASMC were treated for 24h with control or oxLDL‐EVs.

**Results**: EVs were successfully purified and NTA revealed no significant differences in concentration or size between control or oxLDL‐EVs (9.9 × 1011±2.8 × 1011 vs 8.9 × 1011±1.6 × 1011 particles/mL; mean sizes of 116.8±4.46nm vs 126.9±5.7nm, respectively (n = 4)). TEM analysis confirmed the presence of rounded particles and the EV‐associated markers, CD81, Annexin A2, and CD63 were detected by western immunoblotting. OxLDL was not detected in purified control or ox‐LDL‐ EVs (n = 2). In HCASMC exposed to either control‐EVs or oxLDL‐EVs no differences in viability were observed via MTT assay (n = 3).

**Summary/Conclusion**: These data suggest that oxLDL treatment does not alter EV release in HCASMCs. Future work will investigate whether EVs from control and ox‐LDL‐stimulated HCASMC trigger other phenotype changes. These studies will contribute to the understanding of EV signaling in cells relevant to atherosclerosis development.

### PS25.13 Extracellular vesicles carrying β2‐adrenergic receptor and tissue plasminogen activator are trapped in coronary circulation during stimulation of cardiac sympathetic nerves in pigs

Trude Aspelin, Oslo University Hospital, Ullevaal

Hans Christian D. Aass, The Blood Cell Research Group, Section for Research, Development and Innovation, Department of Medical Biochemistry, Oslo University Hospital, Ullevål, Norway

Lilly Alice Steffensen, Oslo University Hospital, The Blood Cell Research Group

Morten Eriksen, Oslo University Hospital, Institute for Experimental Medical Research

Anne‐Marie Siebke Trøseid, The Blood Cell Research Group, Section for Research, Development and Innovation, Department of Medical Biochemistry, Oslo University Hospital, Ullevål, Norway

Tonje Bjørnetrø, Akershus University Hospital

Kari Bente Foss Haug, The Blood Cell Research Group, Section for Research, Development and Innovation, Department of Medical Biochemistry, Oslo University Hospital, Ullevål, Norway

Torstein Lyberg, Oslo University Hospital, Department of Medical Biochemistry

Reidun Øvstebø, The Blood Cell Research Group, Section for Research, Development and Innovation, Department of Medical Biochemistry, Oslo University Hospital, Ullevål, Norway

**Introduction**: Acute coronary syndrome (ACS) induces increased levels of circulating extracellular vesicles (EVs). ACS is associated with sympathetic nerve stimulation and increased norepinephrine (NE) levels in blood. The main activator of fibrinolysis tissue plasminogen activator (tPA), has been detected on EVs. Our aim was to investigate transcoronary trapping of EVs during electrical stimulation of cardiac sympathetic nerves (SS) and examine if these EVs carry both β2‐adrenergic receptor (β2AR, NE receptor) and tPA.

**Methods**: In 6 pigs, blood was collected from a femoral artery (A) and a shunt draining coronary venous blood (V) at baseline, 3 min of SS, and 30 min after SS. NE was measured in plasma. EVs were isolated by size exclusion chromatography, characterized by western immunoblot (CD63, Flotillin‐1, Calnexin, tPA and β2AR) and electron microscopy, and concentration measured with nanoparticle tracking analysis. tPA‐positive EVs were pulled down with anti‐tPA‐coated magnetic beads and stained with anti‐β2AR prior to flow cytometric analysis. Coronary trapping of NE, EVs or tPA‐β2AR‐EVs are defined by a positive A‐V difference corrected for coronary local blood flow, whereas a negative value indicates coronary release.

**Results**: SS‐induced mobilization of A‐EVs and simultaneous reduction in V‐EVs indicate coronary trapping of EVs, possibly caused by simultaneously released NE. Pull‐down experiments demonstrated concurrent β2AR and tPA on EVs. Although a tendency during SS, 30 min after SS all 6 pigs demonstrated trapping of tPA‐β2AR‐EVs in the coronary circulation.

**Summary/Conclusion**: Pigs subjected to cardiac sympathetic nerve stimulation demonstrated local NE release and trapping of EVs in the coronary circulation. Selected EVs carried both tPA and β2AR and may thus form a sandwich between EV‐β2AR, NE and β2AR on endothelial cells. EVs carrying tPA and β2AR may therefore represent a new principle to secure local coronary fibrinolysis.

